# Regioselective, catalytic 1,1-difluorination of enynes

**DOI:** 10.1038/s41557-023-01344-5

**Published:** 2023-10-16

**Authors:** Zi-Xuan Wang, Keith Livingstone, Carla Hümpel, Constantin G. Daniliuc, Christian Mück-Lichtenfeld, Ryan Gilmour

**Affiliations:** 1 Institute for Organic Chemistry, Westfälische Wilhelms-Universität (WWU) Münster, Münster, Germany; 2 Cells in Motion (CiM) Interfaculty Center, Westfälische Wilhelms-Universität (WWU) Münster, Münster, Germany

**Keywords:** Organocatalysis, Synthetic chemistry methodology, Synthetic chemistry methodology

## Abstract

Fluorinated small molecules are prevalent across the functional small-molecule spectrum, but the scarcity of naturally occurring sources creates an opportunity for creative endeavour in developing routes to access these important materials. Iodine(I)/iodine(III) catalysis has proven to be particularly well-suited to this task, enabling abundant alkene substrates to be readily intercepted by in situ-generated *λ*^3^-iodanes and processed to high-value (di)fluorinated products. These organocatalysis paradigms often emulate metal-based processes by engaging the *π* bond and, in the case of styrenes, facilitating fluorinative phenonium-ion rearrangements to generate difluoromethylene units. Here we demonstrate that enynes are competent proxies for styrenes, thereby mitigating the recurrent need for aryl substituents, and enabling highly versatile homopropargylic difluorides to be generated in an operationally simple manner. The scope of the method is disclosed, together with application in target synthesis (>30 examples, up to >90% yield).

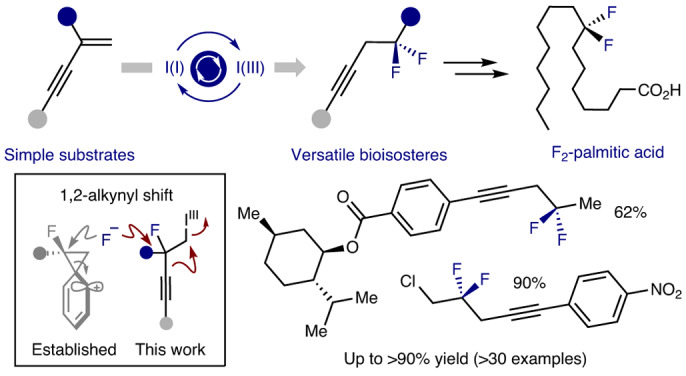

## Abstract (web summary)

Hypervalent iodine catalysis remains a powerful method to enable geminal difluoromethylenation of alkenes. However, the scope is mainly limited to styrene derivatives. Now, enynes have been validated as competent substrates where a formal 1,2-shift of the alkyne occurs, thereby enabling highly versatile homopropargylic difluorides to be generated.

## Main

The synergistic interplay of precision synthesis^1,2^ and functional small-molecule design^3^ continues to be a major driver of innovation in both disciplines. In the vanguard of enabling technologies, fluorination has a venerable history in tailoring the physiochemical traits of promising active pharmaceutical ingredients (APIs), and the societal impact of Fried’s seminal work on fluorinated steroids is a compelling exemplar^4^. Function-driven synthesis thus continues to provide a powerful incentive to expand the current methodological arsenal under the auspices of atom and step efficiency^5^. In particular, the success of the geminal-difluoromethylene group in leading pharmaceuticals has stimulated much interest in the development of main group catalysis-based strategies to facilitate installation from readily available precursors^6–8^.

**Fig. 1:**
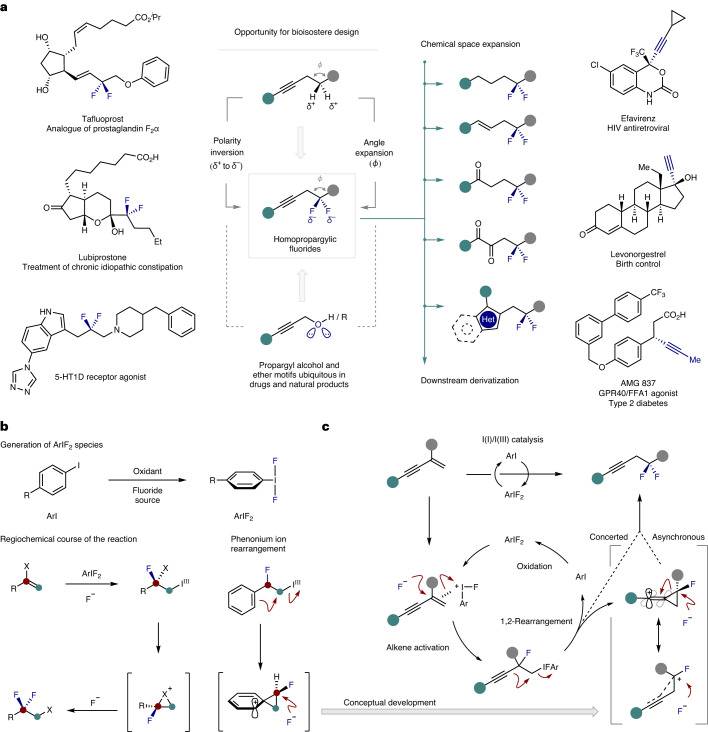
Development of a catalytic *gem*-difluorination of enynes. **a**, Bioisostere design and examples of bioactive molecules containing a CF_2_ or alkyne moiety. **b**, Hypervalent iodine-catalysed *gem*-difluorination of alkenes and the phenonium-ion rearrangement. **c**, Reaction blueprint to enable catalysis-based fluorinative alkyne-migration. The electron-rich alkyne is envisaged to be a competent proxy for phenyl, enabling the phenonium-ion rearrangement to be replaced by a formal 1,2-alkynyl shift via a stabilized vinyl cation.

The prominence of fluorination patterns in contemporary drug discovery^9–14^ disguises the comparative scarcity of naturally occurring organofluorines in marine and terrestrial environments^15,16^. Although more than 5,000 halogen-containing natural products have been described so far^17,18^, and fluorine sources are accessible, it is manifest that nature has not been compelled to evolved fluorine biochemistry to any substantial degree^19,20^. This fluorous juxtaposition between natural and synthetic functional small molecules continues to provide opportunities for the conception and development of new molecular entities with geometries and physiochemistries that are not encountered in biology^21–23^, and it logically follows that this continues to expand the chemical space available for function-driven synthesis. A compelling exemplar is the bond-angle distortion that results from CH_2_ to CF_2_ replacement^24^, which renders the difluoromethylene group a validated bioisostere of oxygen in phosphate mimics^25,26^. This motif is finding increasing application in the small-molecule drug repertoire, with prominent examples including lubiprostone (Amitiza), tafluprost (Taflotan) and various 5-HT1D agonists (Fig. 1a)^27^. Motivated by the demand for new fluorinated modules for medicinal chemistry^12,14^, and cognizant of the emergent importance of alkyne-containing APIs such as efavirenz (Estiva) and levonorgestrel (PlanB One-Step), it was envisaged that a route to homopropargylic difluorides would address a gap in the discovery portfolio: this would provide isosteric surrogates of propargylic ethers and alcohols in which the electronegativity of the fluorine atoms would emulate the non-bonding electron pairs^28^.

Of the many enabling innovations that enable direct, geminal difluoromethylenation of alkenes, hypervalent iodine (I/III) catalysis has proven particularly powerful^29–39^. However, a precondition of this strategy is the requirement for substrates that undergo skeletal rearrangement to ensure that the desired 1,1-regioselectivity of the fluorination is reached (Fig. 1b)^40^. This restraint continues to limit the scope of the transformation to styrene derivatives in which a phenonium-ion rearrangement occurs^41–45^. Although the introduction of heteroatom substituents partially circumvents this limitation^35,36,46–48^, geminal difluorination in the presence of carbon-based groups, in the absence of aryl substituents^43^, remain conspicuously challenging. To address this, it was envisaged that enynes would be attractive substrates in which the electron-rich alkyne would serve as a phenyl proxy. This would enable the regiochemical paradigm predicated on the phenonium-ion rearrangement to be replaced by a formal 1,2-alkynyl shift via a stabilized vinyl cation (Fig. 1c). Homopropargylic fluorides would also enable direct access to homoallylic and alkyl difluorides, thereby expanding the impact of catalytic difluorinations enabled by I(I)/I(III) catalysis.

A catalytic cycle was conceived based on the in situ generation of an ArIF_2_ species, via a process of ligand exchange, that would promote an alkene-activation and fluorination sequence. Should the key rearrangement be successful, then the product cation would benefit from fluorine as a stabilizing auxiliary^49^. This would provide a facile route to homopropargylic difluorides, in which the alkyne handle would facilitate downstream functionalization.

## Results and discussion

To validate the working hypothesis delineated in Fig. 1c, enyne **S1** was prepared and exposed to catalysis conditions using various inexpensive aryl iodides, oxidants and HF sources (Table 1). Initially, *p*-TolI was combined with Selectfluor and amine•HF (1:7 ratio) in chloroform at ambient temperature. This enabled the desired homopropargylic fluoride **1** to be generated in 88% yield. Importantly, the *vicinal* regioisomer was not formed under these conditions, as determined by ^19^F NMR (<5%). However, in the absence of the catalyst, the *vicinal* difluoride was formed in 13% yield. A screen of electronically modulated catalysts confirmed the superiority of *p*-TolI, and revealed the following trend: *p*-Me > *p*-H > *p*-CO_2_Me > *p*-OMe. Modifying the amine:HF ratio or the oxidant were found to have a detrimental effect on the reaction outcome (Table 1).

**Table 1:** Reaction optimization


Entry	Modified conditions	Yield (%)
1	Amine•HF 1:4.5	55
2	Amine•HF 1:9.2	41
3	Oxone as oxidant	51
4	*m*-CPBA as oxidant^a^	74
5	10 mol% *p*-TolI	78
6	Reaction at 0 °C	24
7^b^	No catalyst	<5
8	No oxidant	<5

Standard reaction conditions: enyne **S1** (0.1 mmol), catalyst (20 mol%), amine•HF 1:7.0 (0.25 ml), CHCl_3_ (0.25 ml) and Selectfluor (0.15 mmol). Yields were determined by ^19^F NMR using ethyl fluoroacetate as an internal standard.

^a^meta-Chloroperoxybenzoic. ^b^Amine•HF ratio changed to 1:7.5.

Having identified optimized conditions for the title reaction, the scope and limitations of the transformation were investigated. In the course of this process, reactivity differences were noted in response to subtle changes in the amine:HF ratio. This is in line with early observations related to the impact of trifluoroacetic acid on the reactivity of iodobenzene dichloride^50^. For that purpose, a gradient of amine:HF ratios was considered starting from 1:4.5 and increasing to 1:7.0 (denoted A–F). For simplicity, only the most effective conditions are indicated in Table 2. Initially, the impact of modifying the capping aryl group was investigated while keeping the alkene substituent constant (R = Me). This enabled a series of *gem*-difluorides to be generated, and demonstrated functional-group compatibility with electron-withdrawing groups, halogens and small alkyl fragments (**1**–**10**, up to 83%). In the case of product **2**, it was possible to rigorously establish the molecular connectivity by single-crystal X-ray diffraction (Table 2; CCDC 2256836). Gratifyingly, the method also proved to be compatible with medicinally relevant heterocycles such as pyridines, quinolines and morpholines (**11**–**13**, up to 56%). Furthermore, it was possible to replace R = Me with R = CH_2_X (X = Br and Cl) to create linchpins that could be functionalized at the proximal C(*sp*^3^) position (**15** and **16**, up to 91%). Finally, the compatibility of the method with more complex natural-product-derived scaffolds was validated (**17**–**19**).

**Table 2:**
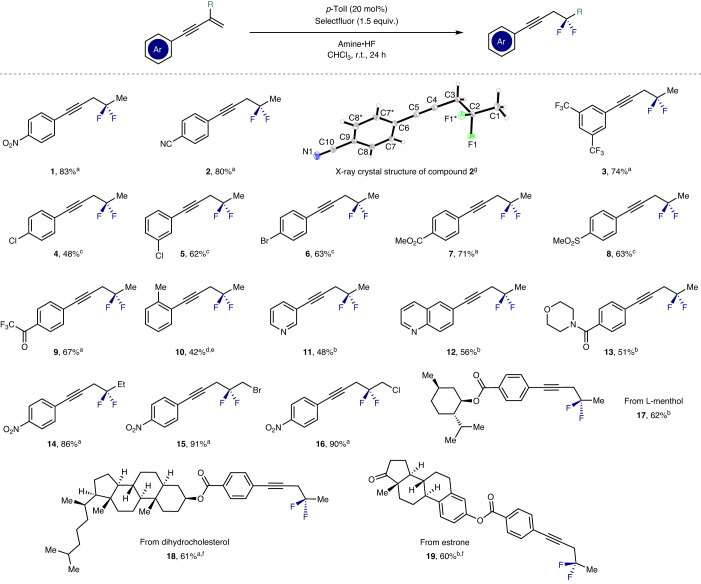
Establishing the scope of aryl alkynes

Reaction conditions: enyne (0.2 mmol), catalyst (20 mol%), amine•HF (0.5 ml), CHCl_3_ (0.5 ml) and Selectfluor (0.3 mmol). Isolated yields are given.

^a^Amine:HF = 1:7.0.

^b^Amine:HF = 1:6.0.

^c^Amine:HF = 1:5.0.

^d^Amine:HF = 1:4.5.

^e19^F NMR yield using ethyl fluoroacetate as an internal standard. 23% of the vicinal difluorination product was formed due to an uncatalysed background reaction (details are provided in Supplementary section 1.3).

^f^Reaction performed on a 0.10 mmol scale.

^g^Thermal ellipsoids are shown at 50% probability. Care should be exercised during isolation due to the volatility of many of the products.

To advance the scope of the transformation beyond aryl-substituted enynes, aliphatic derivatives were then explored with a view to applying the method to target synthesis (Table 3). Simple alkyl and cycloalkyl derivatives were tolerated (**20** and **21**, up to 63%) and it was possible to introduce functionality in the form of phthalimides (**22**, 50%) and ethers (**23**, 71%). Substrates with potentially challenging benzylic/propargylic positions such as **24** were smoothly converted to the desired product. The transformation was found to be chemoselective for the enyne versus cinnamoyl motifs (**25**, 64%), and alkynoic esters (**26**, 63%), tosylates (**27**, 56%) and alcohols (**28**, 41%) were compatible. Modifying the alkyl substituent was possible (**29**, **30**) and enabled the 1,2,2-trifluoro motif to be generated in a facile manner. The introduction of more complex heterocycles, such as in febuxostat (Adenuric) derivative **32**, is an encouraging validation of the method in a drug-discovery setting. With a view to accessing the parent motif derived from the unsubstituted enyne, the triisopropylsilyl (TIPS)-acetylene **33** was prepared in 67% yield.

**Table 3:**
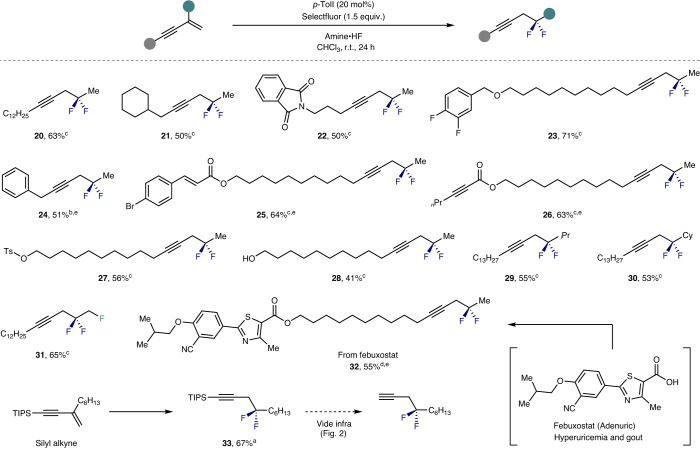
Expanding the scope to alkyl and silyl alkynes

Reaction conditions: enyne (0.2 mmol), catalyst (20 mol%), amine•HF (0.5 ml), CHCl_3_ (0.5 ml) and Selectfluor (0.3 mmol). Isolated yields are reported.

^a^Amine:HF = 1:7.0.

^b^Amine:HF = 1:6.5.

^c^Amine:HF = 1:6.0.

^d^Amine:HF = 1:5.5.

^e^Reaction performed on a 0.10 mmol scale.

**Fig. 2:**
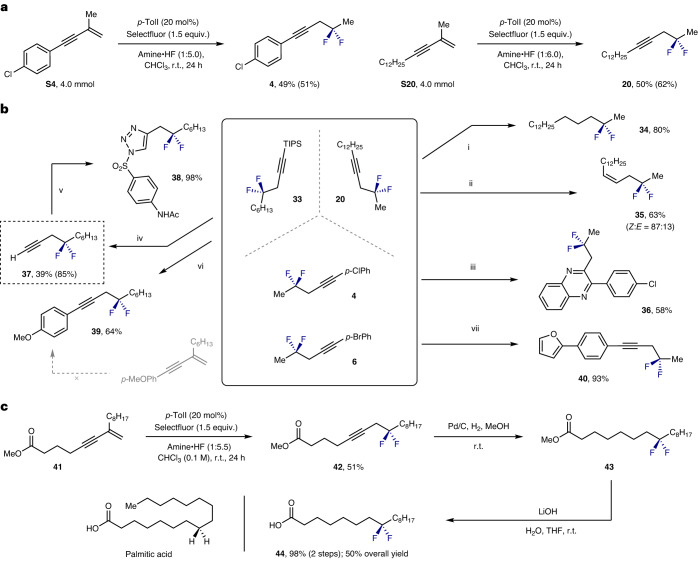
Synthetic applications. **a**, Scale-up experiments. **b**, Product derivatization. Conditions. (i) Conversion of **20** to form **34:** Pd/C (10 mol%), H_2_, MeOH (0.1 M), r.t., 24 h. (ii) Semi-reduction to generate the *Z*-alkene **35**: TiCl_2_Cp_2_ (10 mol%), LiAlH_4_ (2.0 equiv.), tetrahydrofuran (THF) (0.2 M), r.t., overnight. (iii) Generation of quinoxaline **36**: (1) RuCl_3_ (1 mol%), PhI(OAc)_2_ (3.0 equiv.), DCM, H_2_O (4:1, 0.2 M), r.t., 3 h; (2) saccharin (5 mol%), 1,2-phenylendiamine (1.1 equiv.), MeOH (0.2 M), r.t., 12 h. (iv) TIPS deprotection to generate terminal alkyne **37**: TBAF (2.0 equiv.), THF (0.4 M), r.t., 2 h. (v) Formation of triazole **38** via a copper-catalysed click reaction: CuTc (10 mol%), 4-acetamidobenzenesulfonyl azide (1.2 equiv.), toluene (0.2 M), r.t., 12 h. (vi) In situ deprotection of **33** and subsequent Sonogashira cross-coupling: Pd(PPh_3_)_2_Cl_2_ (4.5 mol%), CuI (5 mol%), 4-iodoanisole (1.2 equiv.), NEt_3_ (7.0 equiv.), TBAF (2.0 equiv.), THF (0.2 M), 45 °C, 12 h. (vii) Suzuki-coupling to generate compound **40**: Pd(PPh_3_)_4_ (10 mol%), 2-furanboronic acid (2.5 equiv.), K_2_CO_3_ (2.5 equiv., 2 M in H_2_O), dimethoxyethane (DME) (0.2 M), 85 °C, overnight. **c**, Synthesis of CF_2_-modified palmitic acid. Isolated yields are given. ^19^F NMR yields are given in parentheses and determined by ^19^F NMR using ethyl fluoroacetate as an internal standard.

To demonstrate the synthetic utility of this geminal difluorination of enynes, two representative experiments were validated on a 4.0 mmol scale (Fig. 2a), and a series of product derivatization reactions were conducted (Fig. 2b). Initially, alkyne **20** was fully and partially reduced^51^ to the alkane and alkene products **34** and **35**, respectively. To demonstrate the value of the homopropargylic fluorides in heterocycle formation, compound **4** was converted to the quinoxaline **36** through Ru-catalysed oxidation of the alkyne and concomitant condensation with 1,2-phenylendiamine^52,53^. Desilylation of compound **33** with tetrabutylammonium fluoride (TBAF) furnished the terminal alkyne **37** in 85% yield: this could then be processed further to triazole **38** via a copper-catalysed click reaction^54^. In situ deprotection of **33** and subsequent Sonogashira cross-coupling proved facile, enabling the electron-rich aryl alkyne **39** to be generated in 64% yield. Because electron-rich enynes undergo uncatalysed side reactions with the Selectfluor^55^, this approach enables the geminal difluorination products to be generated by an alternative route. Finally, the compatibility of the motif under Suzuki–Miyaura conditions was demonstrated through the generation of compound **40** (93% yield)^56^.

A concise route to the CF_2_-modified palmitic acid **44** was conceived to validate the method in target synthesis (Fig. 2c). Initially reported by O’Hagan and co-workers in the context of a wider study of the conformational preferences of palmitic acids and nonadecane containing CF_2_ groups^57^, this molecule remains a benchmark in difluorination method development^58^. With the aim of complementing the existing reagent-based approaches, enyne **41** was exposed to the catalytic geminal-difluorination conditions: this furnished the key intermediate **42** in 51% yield. Chemoselective reduction of the alkyne and saponification of the methyl ester enabled the desired compound **44** to be generated in 98% yield over two steps.

**Fig. 3:**
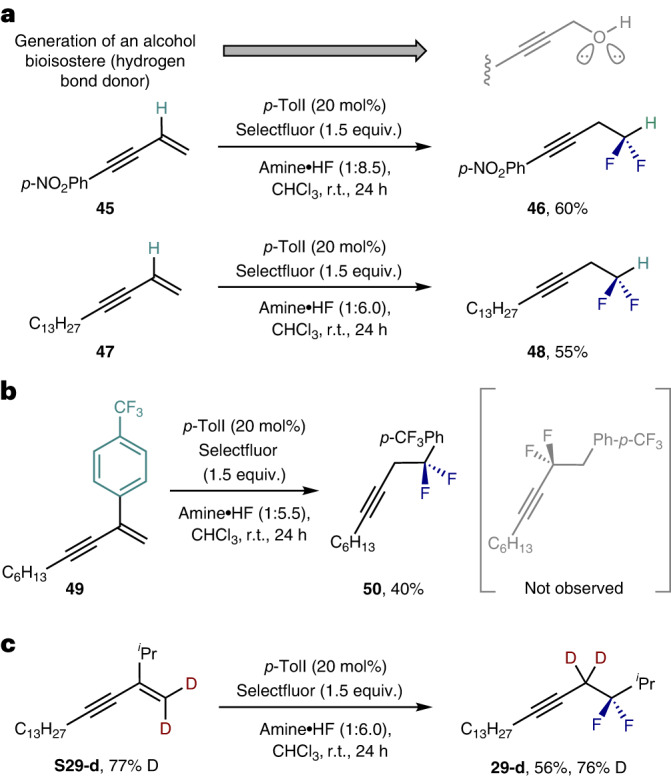
Control experiments. **a**, Removal of the alkyl group. Enynes **45** and **47** could be converted to **46** and **48**, containing the difluoromethyl group, respectively. **b**, Investigation of regioselectivity. Compound **50** was obtained as the sole product. **c**, Deuterium labelling experiment. Deuterium atom incorporated exclusively at the propargylic position, unambiguously demonstrating that 1,2-alkynyl migration took place. Isolated yields are given.

Finally, control reactions were performed to establish that deletion of the alkyl substituent was tolerated (Fig. 3). Interest in the difluoromethyl group as a surrogate of primary alcohols^28^ renders such products appealing in the wider context of molecular design on account of their hydrogen-donor character^59^. Pleasingly, both the aryl- and alkyl-substituted enynes **45** and **47** could be processed to their respective homopropargyl difluoride products **46** and **48**, respectively. Replacing the substituent with an aryl group (**49** Ar = *p*-CF_3_) was then explored to identify which regioisomer was predominantly formed. The isolation of compound **50** as the sole product of the reaction (40% yield) is consistent with the 1,2-shift out-competing phenonium-ion rearrangement. The skeletal rearrangement that is central to the working hypothesis was supported by deuterium labelling to generate **29**-**d** (56%, 76% D incorporation; Fig. 3c).

## Conclusions

The direct, geminal difluorination of alkenes under the auspices of hypervalent iodine catalysis remains a powerful paradigm to expand organofluorine chemical space for contemporary drug discovery. In situ-generated *λ*^3^-iodanes regulate regiocontrol by inducing C(*sp*^3^)–F bond-forming/rearrangement sequences with exquisite efficiency: the latter step is conditional on substrates that are predisposed to undergo a phenonium-ion rearrangement. To circumvent this limitation, enynes have been validated as competent substrates that deliver the desired 1,1-selectivity, where the phenonium-ion rearrangement can be replaced by a formal 1,2-shift of the alkyne. Computational support for the tentative mechanism outlined in Fig. 1 is available in Supplementary section 1.7. Utilizing the alkyne as a phenyl proxy, it has been possible to achieve the title reaction and deliver homopropargylic difluorides that are highly amenable to downstream functionalization. A broad substrate scope is demonstrated (>30 examples) together with selected derivatization protocols, as well as a short, catalysis-based synthesis of CF_2_-modified palmitic acid. It is envisaged that this enabling method will find application in the conception of new drug-discovery modules.

## Methods

### General procedure for 1,1-difluorination of enynes

Unless otherwise stated, a Teflon vial was equipped with a 1-cm stirring bar followed by the addition of enyne (0.2 mmol, 1.0 equiv.), *p*-iodotoluene (9 mg, 0.04 mmol, 20 mol%) and CHCl_3_ (0.5 ml). The stated amine:HF mixture was added (0.5 ml) via syringe. After stirring for 1 min, Selectfluor (106 mg, 0.3 mmol, 1.5 equiv.) was added in one portion. The reaction vessel was then sealed with a Teflon screw cap. After stirring (350 r.p.m.) at ambient temperature for 24 h, the reaction mixture was poured into 100 ml of a saturated solution of NaHCO_3_ (caution! generation of CO_2_!). The Teflon vial was rinsed with dichloromethane (DCM) and dropped into another flask of saturated aqueous solution of NaHCO_3_ to guarantee the removal of excess HF. The organics were extracted with DCM (3 × 30 ml), the combined organic layers were dried over Na_2_SO_4_, filtered, and the solvent was carefully removed under reduced pressure. An internal standard (ethyl fluoroacetate) was added to the crude residue and the NMR yield was analysed by ^19^F NMR spectroscopy against the internal standard. The NMR sample was recombined with the crude residue and purification by column chromatography or preparative thin-layer chromatography yielded the desired product.

## Online content

Any methods, additional references, Nature Portfolio reporting summaries, source data, extended data, supplementary information, acknowledgements, peer review information; details of author contributions and competing interests; and statements of data and code availability are available at 10.1038/s41557-023-01344-5.

### Supplementary information

Supplementary Information Supplementary Figs. 1–278, Methods and references.

Supplementary Data 1 Computational data.

Supplementary Data 2 Crystallographic data for compound **2**; CCDC reference 2256836.

## Data Availability

Crystallographic data for the structure reported in this Article have been deposited at the Cambridge Crystallographic Data Centre, under deposition nos. CCDC 2256836 (**2**). Copies of the data can be obtained free of charge via https://www.ccdc.cam.ac.uk/structures/. All data are available in the main text or the Supplementary Information.
