# Supplementary material for: Regioselective, catalytic 1,1-difluorination of enynes
Source: Nat Chem. 2023 Oct 16;15(11):1515–22. doi: 10.1038/s41557-023-01344-5 (PMC10624631; doi:10.1038/s41557-023-01344-5)
Supplement: Supplementary file 1 — Supplementary Figs. 1–278, Methods and references. [file 41557_2023_1344_MOESM1_ESM.pdf]

nature chemistry

Article

https://doi.org/10.1038/s41557-023-01344-5

Regioselective, catalytic 1,1-difluorination of enynes

In the format provided by the authors and unedited

Table of Contents

1. Supplementary Methods 2
1.1 General information 2
1.2 Preparation and characterisation of enynes 4
1.3 Preparation and characterisation of homopropargylic fluorides 34
1.4 Synthetic applications 64
1.4.1 Product derivatisation 64
1.4.2 Synthesis of O´Hagan´s CF_2-modified palmitic acid 71
1.5 Control experiments 74
1.6 X-ray crystallographic data 80
1.7 DFT calculations 82
1.8 NMR-Spectra of key compounds 91
2. Supplementary References 230

1. Supplementary Methods

1.1 General information

All commercially available reagents were purchased as reagent grade from *Sigma Aldrich, Merck*, *Alfa Aesar, TCI, Fluorochem* or *abcr* and were used without further purification unless otherwise stated. Solvents for extractions or chromatographic purifications were bought as technical grade and distilled on a rotary evaporator prior to use. All reactions with HF were run in Teflon® vials. For analytical thin layer chromatography, glass plates coated with SiO_2-60 F254 were used from *Merck*. They were visualized with UV-light (254 nm) or with $KMnO_4$ or CAM solution. Column chromatography was performed using silica gel (40-63 μm, *VWR Chemicals*). For preparative thin layer chromatography, glass plates coated with SiO_2-60 F254 and 2 mm thickness were used from *Merck.* The obtained products are often volatile and care must be taken in the isolation. The NMR measurements were performed on a *Bruker AV300, AV400, Agilent DD2 500* or an *Agilent DD2 600* by the NMR service department of the Organisch-Chemisches Institut, Westfälische Wilhelms-Universität Münster. The chemical shifts were referenced to the residual solvent peak as the internal standard (7.26 ppm for $CDCl_3$, 2.50 ppm for DMSO-d_6 for ^{1}H-NMR, 77.16 ppm for $CDCl_3$, 39.52 ppm for DMSO-d_6 for ^{13}C-NMR). The multiplicity is abbreviated as follows: s (singlet), d (doublet), t (triplet), q (quartet), p (pentet), h (heptet), m (multiplet) and br (broad). The given assignments are supported by additional 1D and 2D NMR experiments. The melting points were determined on a *Büchi* B-545 melting point apparatus with open glass capillaries. The IR measurements were performed on a *Perkin-Elmer 100 FT-IR* spectrometer and the intensities of the bands are assigned as follows: w (weak), m (medium), s (strong). High resolution mass spectrometry was performed by the MS service of the Organisch-Chemisches Institut, Westfälische Wilhelms-Universität Münster on a Bruker Daltonics MicroTof (HRMS-ESI), a Triplequad TSQ 7000 (MS-EI), Triplequad Quattro Micro GC (GC-EI-MS), a Qp5050 Single Quad (GC-EI-MS) or a LTQ Orbitap LTQ XL (HRMS-APCI).

Preparation of various amine·HF mixtures

Amine·HF sources:

$NEt_3 \cdot 3HF$; Supplier: abcr; (MW: 161.21g/mol, ρ = 0.990 g/mL)

Olah's Reagent (70wt% HF: Py·9.23HF); Supplier: Sigma Aldrich; (MW: 263.79 g/mol, ρ = 1.1 g/mL)

Procedure for calculating compositions of amine·HF mixtures:

The amine·HF mixtures based on $NEt_3 \cdot 3HF$ and Olah´s reagent were prepared following the procedure previously described by this group.[1]

1:4.5 (0.5 mL): 0.34 mL NEt_3•3HF and 0.16 mL Olah's reagent

1:5.0 (0.5 mL): 0.29 mL NEt_3•3HF and 0.21 mL Olah's reagent

1:5.5 (0.5 mL): 0.25 mL NEt_3•3HF and 0.25 mL Olah's reagent

1:6.0 (0.5 mL): 0.21 mL NEt_3•3HF and 0.29 mL Olah's reagent

1:6.5 (0.5 mL): 0.17 mL NEt_3•3HF and 0.33 mL Olah's reagent

1:7.0 (0.5 mL): 0.14 mL NEt_3•3HF and 0.36 mL Olah's reagent

1:8.5 (0.5 mL): 0.04 mL NEt_3•3HF and 0.46 mL Olah's reagent

1.2 Preparation and characterisation of enynes

General Procedure A

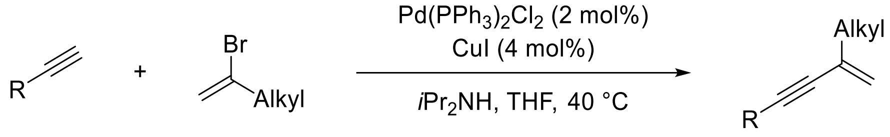

According to a modified literature procedure,[2] to a three-neck flask was added $Pd(PPh_3)_2Cl_2$ (2 mol%), CuI (4 mol%), *i*Pr_2NH (3.0 eq.), THF (0.50 M), alkenyl bromide (1.2 eq.), and alkyne (1.0 eq.) sequentially under Ar atmosphere. The reaction mixture was heated to 40 °C and stirred overnight. After completion of the reaction, the solvent was removed under reduced pressure, a saturated NH_4Cl solution was added to the resulting residue and the aqueous layer was extracted three times with DCM. The combined organic layers were dried over anhydrous Na_2SO_4, filtered and concentrated under reduced pressure. The crude product was purified by column chromatography (SiO_2, specified combination of solvents).

General Procedure B

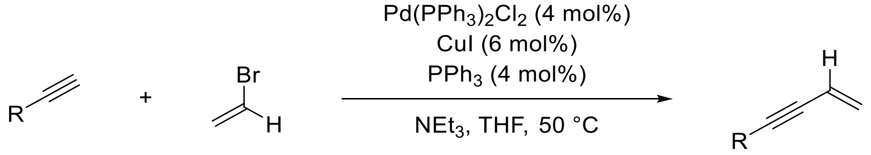

According to a modified literature procedure,[3] $Pd(PPh_3)_2Cl_2$ (4 mol%), CuI (6 mol%), and PPh_3 (4 mol%) were added to a dry flask under an Ar atmosphere. THF (0.50 M), NEt_3 (10.0 eq.), the corresponding alkyne (1.0 eq.), and vinylbromide (1 M in THF, 2.0 eq.) were subsequently added. The reaction mixture was heated to 50 °C and stirred overnight. After completion of the reaction, the solvent was removed under reduced pressure, a saturated NH_4Cl solution was added to the resulting residue and the aqueous layer was extracted three times with DCM. The combined organic layers were dried over anhydrous Na_2SO_4, filtered and concentrated under reduced pressure. The crude product was purified by column chromatography (SiO_2, specified combination of solvents).

General Procedure C

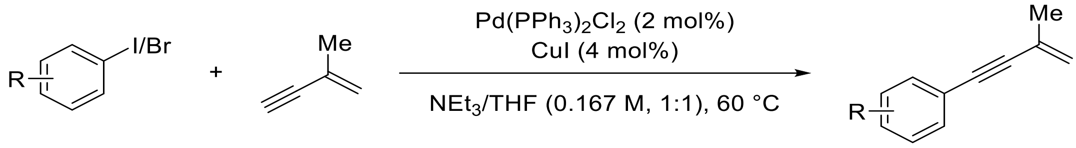

According to a modified literature procedure,[4] a three-necked flask charged with $Pd(PPh_3)_2Cl_2$ (2 mol%) and CuI (4 mol%) was evacuated and backfilled with Ar 3 times, THF and NEt_3 were added at room temperature under argon. Then, aryl bromide or aryl iodide (1.0 eq.) and 2-methylbut-1-en-3-yne (1.1 eq.) were added. The reaction mixture was heated to 60 °C and stirred overnight. After completion of the reaction, the solvent was removed under reduced pressure, a saturated NH_4Cl solution was added to the resulting residue and the aqueous layer was extracted three times with DCM. The combined organic layers were dried over anhydrous Na_2SO_4, filtered and concentrated under reduced pressure. The crude product was purified by column chromatography (SiO_2, specified combination of solvents).

General Procedure D

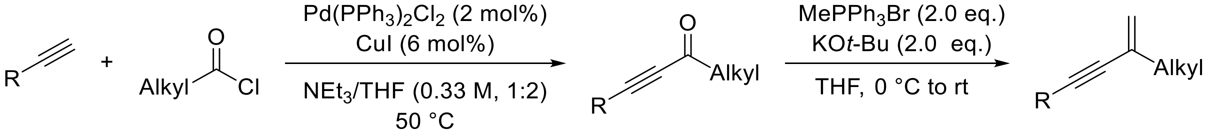

According to a modified literature procedure,[5] a three-necked flask charged with $Pd(PPh_3)_2Cl_2$ (2 mol%) and CuI (6 mol%) was evacuated and backfilled with Ar 3 times, THF and NEt_3 were added at room temperature under argon. Then alkyne (1.0 eq.) and acyl chloride (1.5 eq.) were subsequently added to the resulting suspension. The reaction mixture was heated to 50 °C and stirred for 24 h. After completion of the reaction, the reaction mixture was filtrated through a small pad of silica gel (pentane:Et_2O = 10:1). The solvent was removed under reduced pressure with the aid of a rotary evaporator to give the crude product ketone, which was used without any further purification for the next step.

A flame-dried Schlenk-flask charged with methyltriphenylphosphonium bromide (2.0 eq.) was evacuated and backfilled with Ar 3 times before dry THF (0.167 M) was added. Potassium *tert*-butoxide (2.0 eq.) was added to the suspension at 0 °C (ice bath) and a bright yellow color was observed. The mixture was stirred at 0 °C for 30 min. The crude product ketone (1.0 eq.) was subsequently added. The reaction mixture was stirred at 0 °C for 3 h then allowed to warm up

to room temperature. The stirring was continued for an additional 15 h. After the reaction was complete, the reaction mixture was diluted with Et_2O and filtered through a small pad of silica gel. The solvent was removed under reduced pressure with the aid of a rotary evaporator and the crude residue was purified by column chromatography (SiO_2, specified combination of solvents).

1-(3-Methylbut-3-en-1-yn-1-yl)-4-nitrobenzene (S1)

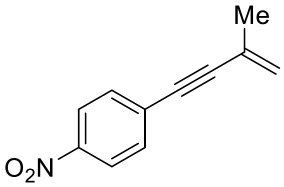

Compound **S1** was prepared according to General Procedure **A** using (4-nitrophenyl)acetylene (2.21 g, 15.00 mmol, 1.0 eq.). The crude residue was purified by column chromatography (*n*-pentane: EtOAc 20:1) to yield the title compound as a yellow oil (1.16 g, 6.21 mmol, 41%).

R*f* = 0.45 (*n*-pentane:EtOAc 20:1).

^{1}H NMR (400 MHz, $CDCl_3$) δ 8.18 (d, *J* = 8.9 Hz, 2H), 7.57 (d, *J* = 8.9 Hz, 2H), 5.52 – 5.46 (m, 1H), 5.45 – 5.39 (m, 1H), 2.01 (t, *J* = 1.3 Hz, 3H).

^{13}C NMR (101 MHz, $CDCl_3$) δ 147.07, 132.38 (*overlapping*), 130.39, 126.28, 124.14, 123.69 (*overlapping*), 95.92, 86.60, 23.24.

GC-EI-MS: (*m/z*) requires: [($C_{11}H_9NO_2$)] = 187.06, (*m/z*) found: [($C_{11}H_9NO_2$)] = 187.10.

Analytical data is in agreement with literature values.[6]

4-(3-Methylbut-3-en-1-yn-1-yl)benzonitrile (S2)

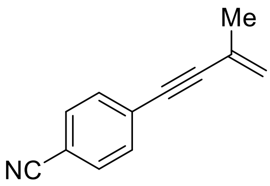

Compound **S2** was prepared according to General Procedure **A** using 4-cyanophenylacetylene (254.3 mg, 2.00 mmol, 1.0 eq.). The crude residue was purified by column chromatography (*n*-pentane: Et_2O 20:1) to yield the title compound as a white solid (116.5 mg, 0.70 mmol, 35%).

R*f* = 0.60 (*n*-pentane:Et_2O 20:1).

^{1}H NMR (400 MHz, $CDCl_3$) δ 7.60 (d, *J* = 8.6 Hz, 2H), 7.51 (d, *J* = 8.7 Hz, 2H), 5.48 – 5.45

(m, 1H), 5.41 – 5.37 (m, 1H), 1.99 (dd, J = 1.6, 1.0 Hz, 3H).

^{13}C NMR (101 MHz, $CDCl_3$) δ 132.15 (*overlapping*), 132.08 (*overlapping*), 128.35, 126.30, 123.80, 118.61, 111.51, 95.00, 86.78, 23.26.

GC-EI-MS: (*m/z*) requires: [($C_{12}H_9N$)] = 167.07, (*m/z*) found: [($C_{12}H_9N$)] = 167.09.

Analytical data is in agreement with literature values.[6]

1-(3-Methylbut-3-en-1-yn-1-yl)-3,5-bis(trifluoromethyl)benzene (S3)

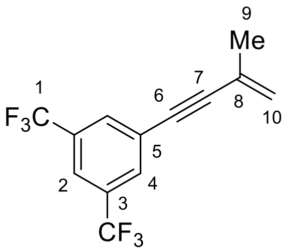

Compound **S3** was prepared according to General Procedure **C** using 3,5-*bis*(trifluoromethyl)iodobenzene (1.36 g, 4.00 mmol, 1.0 eq.). The crude residue was purified by column chromatography (*n*-pentane) to yield the title compound as a colorless oil (0.60 g, 2.16 mmol, 54%).

R$_f$ = 0.80 (*n*-pentane).

^{1}H NMR (500 MHz, $CDCl_3$) δ 7.86 (s, 2H, H-C4), 7.78 (s, 1H, H-C2), 5.50 – 5.47 (m, 1H, H^a-C10), 5.42 – 5.39 (m, 1H, H^b-C10), 2.02 – 1.99 (m, 3H, H-C9).

^{13}C NMR (126 MHz, $CDCl_3$) δ 132.0 (q, $^2J_{CF}$ = 33.7 Hz, C3), 131.6 (m, C4), 126.1 (C8), 125.8 (C5), 124.2 (C10), 123.1 (q, $^1J_{CF}$ = 273.2 Hz, C1), 121.6 (m, C2), 94.1 (C7), 85.4 (C6), 23.2 (C9).

^{19}F NMR (470 MHz, $CDCl_3$) δ -63.20 (s, 3F, F-C1).

^{19}F{^{1}H} NMR (470 MHz, $CDCl_3$) δ -63.20 (s, 3F, F-C1).

GC-EI-MS: (*m/z*) requires: [($C_{13}H_8F_6$)] = 278.0525, (*m/z*) found: [($C_{13}H_8F_6$)] = 278.0518.

FT-IR ($\tilde{\nu}$ = cm^{-1}): 2216 (w), 1617 (w), 1393 (m), 1383 (m), 1372 (m), 1274 (s), 1172 (s), 1128 (s), 1106 (s), 1043 (w), 989 (m), 895 (s), 848 (m), 699 (m), 683 (s).

1-Chloro-4-(3-methylbut-3-en-1-yn-1-yl)benzene (S4)

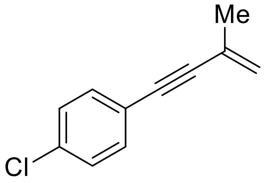

Compound **S4** was prepared according to General Procedure **C** using 4-chloroiodobenzene (2.00 g, 8.41 mmol, 1.0 eq.). The crude residue was purified by column chromatography (*n*-pentane) to yield the title compound as a colorless oil (900.0 mg, 5.11 mmol, 61%).

$\mathbf{R}_f$ = 0.58 (*n*-pentane).

^{1}**H NMR** (400 MHz, $CDCl_3$) δ 7.40 – 7.34 (m, 2H), 7.31 – 7.27 (m, 2H), 5.40 (dq, *J* = 2.0, 1.1 Hz, 1H), 5.32 (app. p, *J* = 1.6 Hz, 1H), 1.99 (dd, *J* = 1.6, 1.0 Hz, 3H).

GC-EI-MS (*m/z*) requires: [($C_{11}H_9Cl$)] = 176.04, (*m/z*) found: [($C_{11}H_9Cl$)] = 176.07.

Analytical data is in agreement with literature values.[2]

1-Chloro-3-(3-methylbut-3-en-1-yn-1-yl)benzene (S5)

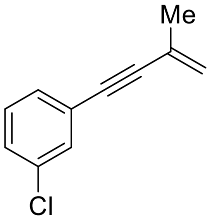

Compound **S5** was prepared according to General Procedure **A** using *m*-chlorophenylacetylene (273.2 mg, 2.00 mmol, 1.0 eq.). The crude residue was purified by column chromatography (*n*-pentane) to yield the title compound as a colorless oil (128.4 mg, 0.73 mmol, 36%).

$\mathbf{R}_f$ = 0.78 (*n*-pentane).

^{1}**H NMR** (400 MHz, $CDCl_3$) δ 7.43 (t, *J* = 1.5 Hz, 1H), 7.34 – 7.21 (m, 3H), 5.43 – 5.39 (m, 1H), 5.35 – 5.30 (m, 1H), 1.98 (dd, *J* = 1.6, 1.0 Hz, 3H).

^{13}**C NMR** (101 MHz, $CDCl_3$) δ 134.26, 131.58, 129.82, 129.63, 128.53, 126.65, 125.16, 122.82, 91.86, 87.05, 23.47.

GC-EI-MS: (*m/z*) requires: [($C_{11}H_9Cl$)] = 176.04, (*m/z*) found: [($C_{11}H_9Cl$)] = 176.07.

Analytical data is in agreement with literature values.[2]

1-Bromo-4-(3-methylbut-3-en-1-yn-1-yl)benzene (S6)

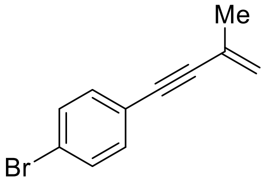

Compound **S6** was prepared according to General Procedure **A** using 4-bromo-1-ethynylbenzene (543.1 mg, 3.00 mmol, 1.0 eq.). The crude residue was purified by column chromatography (*n*-pentane) to yield the title compound as a white solid (421.9 mg, 1.91 mmol, 64%).

R*f* = 0.75 (*n*-pentane).

^{1}H NMR (400 MHz, $CDCl_3$) δ 7.44 (d, *J* = 8.6 Hz, 2H), 7.30 (d, *J* = 8.6 Hz, 2H), 5.43 – 5.37 (m, 1H), 5.34 – 5.30 (m, 1H), 1.98 (dd, *J* = 1.6, 1.0 Hz, 3H).

^{13}C NMR (101 MHz, $CDCl_3$) δ 133.13 (*overlapping*), 131.68 (*overlapping*), 126.76, 122.55, 122.49, 122.39, 91.82, 87.43, 23.49.

GC-EI-MS: (*m/z*) requires: [($C_{11}H_9{}^{79}Br$)] = 219.9882, (*m/z*) found: [($C_{11}H_9{}^{79}Br$)] = 219.9884.

Analytical data is in agreement with literature values.[7]

Methyl 4-(3-methylbut-3-en-1-yn-1-yl)benzoate (S7)

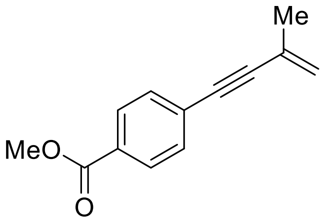

Compound **S7** was prepared according to General Procedure **C** using methyl 4-iodobenzoate (2.10 g, 8.00 mmol, 1.0 eq.). The crude residue was purified by column chromatography (*n*-pentane: Et_2O 30:1) to yield the title compound as a pale yellow solid (1.51 g, 7.56 mmol, 94%).

R*f* = 0.51 (*n*-pentane:Et_2O 20:1).

^{1}H NMR (400 MHz, $CDCl_3$) δ 7.98 (d, *J* = 8.7 Hz, 2H), 7.49 (d, *J* = 8.6 Hz, 2H), 5.47 – 5.41 (m, 1H), 5.38 – 5.33 (m, 1H), 3.92 (s, 3H), 2.00 (dd, *J* = 1.6, 1.0 Hz, 3H).

^{13}C NMR (101 MHz, $CDCl_3$) δ 166.68, 131.61 (*overlapping*), 129.58 (*overlapping*), 129.52, 128.15, 126.65, 123.11, 93.63, 87.72, 52.32, 23.43.

GC-EI-MS: (*m/z*) requires: [($C_{13}H_{12}O_2$)] = 200.0832, (*m/z*) found: [($C_{13}H_{12}O_2$)] = 200.0829.

Analytical data is in agreement with literature values.[8]

1-(3-Methylbut-3-en-1-yn-1-yl)-4-(methylsulfonyl)benzene (S8)

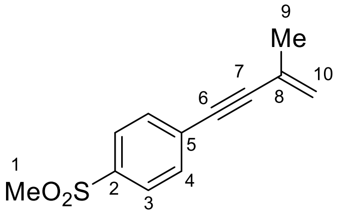

Compound **S8** was prepared according to General Procedure **C** using 4-bromophenyl methyl sulfone (470.2 mg, 2.00 mmol, 1.0 eq.). The crude residue was purified by column chromatography (*n*-pentane: Et_2O 1.5:1) to yield the title compound as a yellow solid (136.4 mg, 0.62 mmol, 31%).

R*f* = 0.51 (*n*-pentane:Et_2O 20:1).

^{1}H NMR (400 MHz, $CDCl_3$) δ 7.89 (d, $^3J_{HH}$ = 8.5 Hz, 2H, H-C3), 7.61 (d, $^3J_{HH}$ = 8.5 Hz, 2H, H-C4), 5.49 – 5.46 (m, 1H, H^a-C10), 5.41 – 5.37 (m, 1H, H^b-C10), 3.06 (s, 3H, H-C1), 2.00 (dd, $^4J_{HH}$ = 1.6, $^4J_{HH}$ = 1.0 Hz, 3H, H-C9).

^{13}C NMR (126 MHz, $CDCl_3$) δ 139.7 (C2), 132.4 (C4), 129.4 (C5), 127.5 (C3), 126.4 (C8), 123.9 (C10), 94.7 (C7), 86.7 (C6), 44.6 (C1), 23.3 (C9).

ESI-MS: (*m/z*) requires: [$(C_{12}H_{12}O_2SNa)^+$] = 243.0450, (*m/z*) found: [$(C_{12}H_{12}O_2SNa)^+$] = 243.0447.

FT-IR ($\tilde{\nu}$ = cm^{-1}): 3016 (w), 2920 (w), 1591 (w), 1574 (m), 1387 (w), 1304 (s), 1277 (m), 1182 (w), 1143 (s), 1086 (s), 1066 (m), 1010 (m), 967 (s), 904 (m), 837 (m), 825 (m), 769 (s), 722 (m), 713 (m).

Melting Point: 62-64 ˚C.

2,2,2-Trifluoro-1-(4-(3-methylbut-3-en-1-yn-1-yl)phenyl)ethan-1-one (S9)

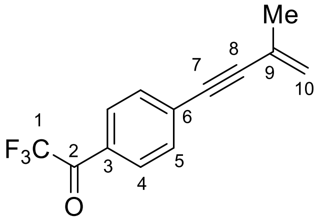

Compound **S9** was prepared according to General Procedure **C** using 4-bromo-2,2,2-trifluoroacetophenone (506.0 mg, 2.00 mmol, 1.0 eq.). The crude residue was purified by column chromatography (*n*-pentane: Et_2O 30:1-2:1) to yield the title compound as a yellow oil (315.6 mg, 1.32 mmol, 66%).

R*f* = 0.49 (*n*-pentane:Et_2O 4:1).

^{1}H NMR (500 MHz, $CDCl_3$) δ 8.02 (d, $^3J_{HH}$ = 7.9 Hz, 2H, H-C4), 7.58 (d, $^3J_{HH}$ = 8.7 Hz, 2H,

H-C5), 5.51 – 5.47 (m, 1H, H^a-C10), 5.43 – 5.39 (m, 1H, H^b-C10), 2.03 – 1.98 (m, 3H, H-C11).

^{13}C NMR (126 MHz, $CDCl_3$) δ 179.9 (t, $^2J_{CF}$= 35.2 Hz, C2), 132.2 (C5), 131.1 (C3), 130.1 (q, $^4J_{CF}$ = 2.2 Hz, C4), 129.0 (C6), 126.4 (C9), 124.1 (C10), 116.8 (q, $^1J_{CF}$ = 291.2 Hz, C1), 96.1 (C8), 87.2 (C7), 23.3 (C11).

^{19}F NMR (470 MHz, $CDCl_3$) δ -71.43 (s, 3F, F-C1).

^{19}F{^{1}H} NMR (470 MHz, $CDCl_3$) δ -71.43 (s, 3F, F-C1).

GC-EI-MS: (*m/z*) requires: [($C_{13}H_9OF_3$)] = 238.0600, (*m/z*) found: [($C_{13}H_9OF_3$)] = 238.0601.

FT-IR ($\tilde{\nu}$ = cm^{-1}): 2210 (w), 1715 (s), 1611 (w), 1598 (m), 1553 (w), 1412 (w), 1375 (w), 1336 (w), 1319 (w), 1284 (w), 1194 (s), 1169 (s), 1139 (s), 938 (s), 901 (m), 851 (m), 765 (s), 753 (m), 695 (m).

1-Methyl-2-(3-methylbut-3-en-1-yn-1-yl)benzene (S10)

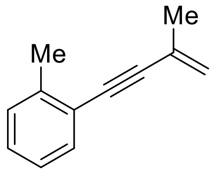

Compound **S10** was prepared according to General Procedure **A** using 1-ethynyl-2-methylbenzene (464.6 mg, 4.00 mmol, 1.0 eq.). The crude residue was purified by column chromatography (*n*-pentane) to yield the title compound as a colorless oil (343.7 mg, 2.20 mmol, 55%).

R*f* = 0.80 (*n*-pentane).

^{1}H NMR (400 MHz, $CDCl_3$) δ 7.40 (d, *J* = 7.5 Hz, 1H), 7.23 – 7.17 (m, 2H), 7.17 – 7.10 (m, 1H), 5.41 – 5.36 (m, 1H), 5.31 – 5.26 (m, 1H), 2.44 (s, 3H), 2.01 (t, *J* = 1.3 Hz, 3H).

^{13}C NMR (101 MHz, $CDCl_3$) δ 140.24, 131.93, 129.52, 128.32, 127.20, 125.64, 123.15, 121.61, 94.72, 87.51, 77.48, 20.76.

GC-EI-MS: (*m/z*) requires: [($C_{12}H_{12}$)] = 156.0934, (*m/z*) found: [($C_{12}H_{12}$)] = 156.0933.

Analytical data is in agreement with literature values.[6]

3-(3-Methylbut-3-en-1-yn-1-yl)pyridine (S11)

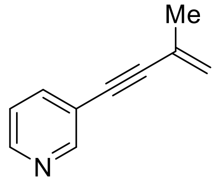

Compound **S11** was prepared according to General Procedure **C** using 3-iodopyridine (410.0 mg, 2.00 mmol, 1.0 eq.). The crude residue was purified by column chromatography (*n*-pentane: Et_2O 5:1-4:1) to yield the title compound as a brownish-red oil (256.0 mg, 1.79 mmol, 89%).

$\mathbf{R}_f$ = 0.39 (*n*-pentane:Et_2O 4:1).

^{1}H NMR (400 MHz, $CDCl_3$) δ 8.67 (d, *J* = 2.2 Hz, 1H), 8.52 (dd, *J* = 4.9, 1.7 Hz, 1H), 7.72 (dt, *J* = 7.9, 1.9 Hz, 1H), 7.26 – 7.22 (m, 1H), 5.47 – 5.42 (m, 1H), 5.36 (p, *J* = 1.7 Hz, 1H), 2.00 (t, *J* = 1.3 Hz, 3H).

^{13}C NMR (101 MHz, $CDCl_3$) δ 152.26, 148.49, 138.52, 126.43, 123.15, 123.08, 120.58, 93.93, 84.98, 23.36.

ESI-MS: (*m/z*) requires: $[(C_{10}H_9NH)^+]$ = 144.0808, (*m/z*) found: $[(C_{10}H_9NH)^+]$ = 144.0804.

Analytical data is in agreement with literature values.[9]

6-(3-Methylbut-3-en-1-yn-1-yl)quinoline (S12)

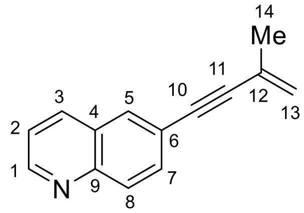

Compound **S12** was prepared according to General Procedure **C** using 6-iodoquinoline (765.2 mg, 3.00 mmol, 1.0 eq.). The crude residue was purified by column chromatography (*n*-pentane: Et_2O 1.5:1-1:1) to yield the title compound as a brownish-red oil (574.4 mg, 2.97 mmol, 99%).

$\mathbf{R}_f$ = 0.31 (*n*-pentane:Et_2O 1.5:1).

^{1}H NMR (599 MHz, $CDCl_3$) δ 8.90 (dd, $^3J_{HH}$ = 4.3 Hz, $^4J_{HH}$ = 1.7 Hz, 1H, H-C1), 8.13 – 8.07 (m, 1H, H-C3), 8.03 (d, $^3J_{HH}$ = 8.7 Hz, 1H, H-C8), 7.93 (d, $^4J_{HH}$ = 1.8 Hz, 1H, H-C5), 7.73 (dd, $^3J_{HH}$ = 8.7 Hz, $^4J_{HH}$ = 1.9 Hz, 1H, H-C7), 7.40 (dd, $^3J_{HH}$ = 8.3, 4.2 Hz, 1H, H-C2), 5.48 – 5.45 (m, 1H, H^a-C13), 5.37 – 5.35 (m, 1H, H^b-C13), 2.03 (dd, $^4J_{HH}$ = 1.6, 1.0 Hz, 3H).

^{13}C NMR (151 MHz, $CDCl_3$) δ 151.0 (C1), 147.8 (C9), 135.8 (C3), 132.3 (C7), 131.2 (C5), 129.7 (C8), 128.2 (C4), 126.8 (C12), 122.8 (C13), 121.9 (C2), 121.8 (C6), 92.0 (C11), 88.1

(C10), 23.6 (C14).

GC-EI-MS: (*m/z*) requires: [($C_{14}H_{11}N$)] = 193.0886, (*m/z*) found: [($C_{14}H_{11}N$)] = 193.0882.

FT-IR ($\tilde{\nu}$ = cm^{-1}): 2973 (w), 2921 (w), 1607 (w), 1591 (m), 1567 (w), 1494 (s), 1459 (w), 1432 (w), 1373 (m), 1350 (w), 1332 (m), 1296 (m), 1158 (w), 1118 (w), 888 (s), 837 (s), 795 (m), 768 (m), 631 (w), 607 (w).

(4-(3-Methylbut-3-en-1-yn-1-yl)phenyl)(morpholino)methanone (S13)

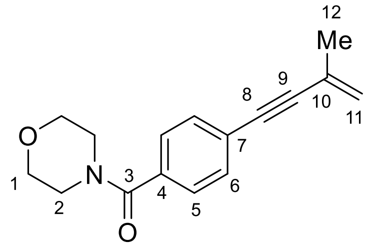

A mixture of methyl 4-(3-methylbut-3-en-1-yn-1-yl)benzoate (**S7**) (600.7 mg, 3.00 mmol, 1.0 eq.) and LiOH (431.1 mg, 18.00 mmol, 6.0 eq.) in THF (9 mL) and H_2O (9 mL) was vigorously stirred at 50 °C for 8 h. After the reaction was quenched with saturated aqueous NH_4Cl, the water layer was acidified with concentrated HCl (pH = 3) and extracted with DCM. The organic layer was dried with Na_2SO_4, concentrated and the crude 4-(3-methylbut-3-en-1-yn-1-yl)benzoic acid was directly used in next step without further purification.

To a solution of crude 4-(3-methylbut-3-en-1-yn-1-yl)benzoic acid (186.2 mg, 1.00 mmol, 1.0 equiv.) in DCM (2 mL) was added 1-ethyl-3-(3-(dimethylamino)propyl)-carbodiimide hydrochloride (EDCI) (287.6 mg, 1.50 mmol, 1.5 eq.), *N,N*-dimethylaminopyridine (12.2 mg, 0.10 mmol, 10 mol%) and then morpholine (130.7 mg, 1.50 mmol, 1.5 eq.). The reaction mixture was stirred at room temperature for 12 h, before DCM was added. The mixture was washed with brine and dried over Na_2SO_4. After removal of the solvent, the residue was purified by column chromatography (*n*-pentane:EtOAc 1.5:1) to yield the title compound as a white solid (213.0 mg, 0.83 mmol, 83%).

$\mathbf{R}_f$ = 0.35 (*n*-pentane:EtOAc 1.5:1).

^{1}H NMR (500 MHz, $CDCl_3$) δ 7.49 – 7.45 (m, 2H, H-C6), 7.38 – 7.33 (m, 2H, H-C5), 5.44 – 5.39 (m, 1H, H^a-C11), 5.35 – 5.32 (m, 1H, H^b-C11), 3.98 – 3.19 (m, 8H, H-C1, H-C2), 1.99 (dd, $^4J_{HH}$ = 1.6, 1.0 Hz, 3H, H-C12).

^{13}**C NMR** (126 MHz, $CDCl_3$) δ 170.0 (C3), 134.8 (C7), 131.8 (C6), 127.3 (C5), 126.7 (C10), 125.3 (C4), 122.8 (C11), 92.3 (C9), 87.6 (C8), 67.0 (C1, C2), 23.5 (C12).

ESI-MS: (*m/z*) requires: $[(C_{16}H_{17}NO_2Na)^+]$ = 278.1152, (*m/z*) found: $[(C_{16}H_{17}NO_2Na)^+]$ = 278.1149.

FT-IR ($\tilde{\nu}$ = cm^{-1}): 2970 (w), 2919 (w), 2855 (w), 1633 (s), 1455 (m), 1428 (s), 1300 (w), 1279 (s), 1257 (m), 1155 (w), 1115 (s), 1066 (w), 1025 (m), 1012 (m), 894 (m), 857 (w), 837 (m), 762 (w).

Melting Point: 81-83 °C.

1-(3-Methylenepent-1-yn-1-yl)-4-nitrobenzene (S14)

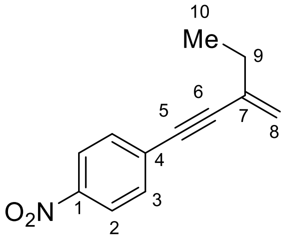

Compound **S14** was prepared according to General Procedure **A** using (4-nitrophenyl)acetylene (441.4 mg, 3.00 mmol, 1.0 eq.). The crude residue was purified by column chromatography (*n*-pentane:Et_2O 30:1) to yield the title compound as a yellow oil (216.0 mg, 1.07 mmol, 36%).

$\mathbf{R}_f$ = 0.61 (*n*-pentane:Et_2O 20:1).

^{1}**H NMR** (500 MHz, $CDCl_3$) δ 8.21 – 8.15 (m, 2H, H-C2), 7.60 – 7.54 (m, 2H, H-C3), 5.49 (dt, $^2J_{HH}$ = 1.6, $^4J_{HH}$ = 0.9 Hz, 1H, H^a-C8), 5.41 (q, $^2J_{HH}$ = 1.5 Hz, $^4J_{HH}$ = 1.5 Hz, 1H, H^b-C8), 2.29 (qdd, $^3J_{HH}$ = 7.4, $^4J_{HH}$ = 1.5, $^4J_{HH}$ = 0.9 Hz, 2H, H-C9), 1.17 (t, $^3J_{HH}$ = 7.5 Hz, 3H, H-C10).

^{13}**C NMR** (126 MHz, $CDCl_3$) δ 147.1 (C4), 132.7 (C7), 132.4 (C3), 130.5 (C1), 123.7 (C2), 122.3 (C8), 95.3 (C6), 87.4 (C5), 30.2 (C9), 13.0 (C10).

GC-EI-MS: (*m/z*) requires: $[(C_{12}H_{11}NO_2)]$ = 201.0784, (*m/z*) found: $[(C_{12}H_{11}NO_2)]$ = 201.0785.

FT-IR ($\tilde{\nu}$ = cm^{-1}): 2970 (w), 2936 (w), 2203 (w), 1591 (m), 1515 (s), 1491 (w), 1462 (w), 1438 (w), 1403 (w), 1376 (w), 1337 (s), 1306 (m), 1284 (w), 1174 (w), 1106 (m), 1058 (w), 1013 (w), 904 (m), 852 (s), 818 (w), 796 (w), 749 (s), 687 (m).

2-Methylene-4-(4-nitrophenyl)but-3-yn-1-ol (Int 1)

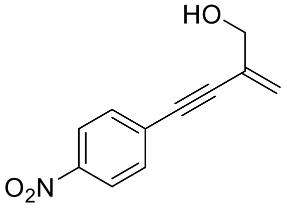

Sodium iodide (6.00 g, 40.00 mmol, 2.0 eq.) and water (432.0 mg, 24.00 mmol, 1.2 eq.) were dissolved in acetonitrile (32 mL). Then TMS-Cl (4.35 g, 40.00 mmol, 2.0 eq.) was added into the mixture at room temperature. After 10 minutes, propargylic alcohol (1.12 g, 20 mmol, 1.0 equiv.) was injected in one portion, followed by stirring at room temperature for another 90 minutes. After that, the reaction mixture was diluted with water and aqueous $Na_2S_2O_3$ (5 wt%). The aqueous phase was extracted with Et_2O, and the combined organic layers were dried over Na_2SO_4, filtered through a small pad of silica gel. The solvent was removed under reduced pressure with the aid of a rotary evaporator to give the crude product 2-iodoprop-2-en-1-ol, which was used without any further purification for the next step.

A three-necked flask charged with $Pd(PPh_3)_2Cl_2$ (56.2 mg, 0.08 mmol, 2 mol%) and CuI (38.1 mg, 0.20 mmol, 5 mol%) was evacuated and backfilled with Ar 3 times, THF and NEt_3 (0.25 M, 1:1) were added at room temperature under argon. Then crude 2-iodoprop-2-en-1-ol (735.9 mg, 4.00 mmol, 1.0 eq.) and (4-nitrophenyl)acetylene (706.2 mg, 4.80 mmol, 1.2 eq.) were added. The reaction mixture was heated to 50 °C and stirred overnight. After completion of the reaction, the solvent was removed under reduced pressure, a saturated NH_4Cl solution was added to the resulting residue and the aqueous layer was extracted three times with DCM. The combined organic layers were dried over anhydrous Na_2SO_4, filtered and concentrated under reduced pressure. The crude product was purified by column chromatography (*n*-pentane:Et_2O 1.5:1-1:1) to yield the title compound as a yellow solid (525.0 mg, 2.58 mmol, 65%).

$\mathbf{R_f}$ = 0.40 (*n*-pentane:Et_2O 1:1).

^{1}H NMR (400 MHz, $CDCl_3$) δ 8.28 – 8.08 (m, 2H), 7.68 – 7.52 (m, 2H), 5.73 (q, *J* = 1.6 Hz, 1H), 5.68 (q, *J* = 1.3 Hz, 1H), 4.27 (d, *J* = 6.3 Hz, 2H), 1.71 (t, *J* = 6.4 Hz, 1H).

^{13}C NMR (101 MHz, $CDCl_3$) δ 147.24, 132.50 (*overlapping*), 130.66, 129.84, 123.71 (*overlapping*), 122.77, 92.37, 88.86, 65.14.

ESI-MS: (*m/z*) requires: $[(C_{11}H_9NO_3Na)^+]$ = 226.0475, (*m/z*) found: $[(C_{11}H_9NO_3Na)^+]$ = 226.0474.

Analytical data is in agreement with literature values.[10]

1-(3-(Bromomethyl)but-3-en-1-yn-1-yl)-4-nitrobenzene (S15)

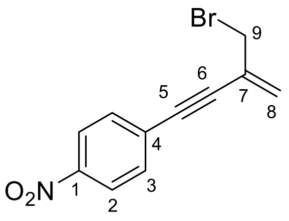

To a solution of 2-methylene-4-(4-nitrophenyl)but-3-yn-1-ol (**Int 1**) (304.8 mg, 1.50 mmol, 1.0 eq.) in Et_2O (0.30 M) at 0 °C was added PBr_3 (203.0 mg, 0.75 mmol, 50 mol%). The solution was warmed to room temperature and stirred for 16 hours. The reaction was cooled to 0 °C and water (1.5 mL) and 5% aq. K_2CO_3 (1.5 mL) were added. The organic phase was isolated, washed with brine, dried over Na_2SO_4 and concentrated in vacuo. The crude residue was purified by column chromatography (*n*-pentane:Et_2O 20:1) to yield the title compound as a pale yellow solid (158.3 mg, 0.59 mmol, 40%).

$\mathbf{R}_{\boldsymbol{f}}$ = 0.49 (*n*-pentane:Et_2O 20:1).

^{1}H NMR (500 MHz, $CDCl_3$) δ 8.23 – 8.18 (m, 2H, H-C2), 7.65 – 7.60 (m, 2H, H-C3), 5.76 (q, $^2J_{HH}$ = 1.0 Hz, $^4J_{HH}$ = 1.0 Hz, 1H, H^a-C8), 5.69 (d, $^2J_{HH}$ = 0.9 Hz, 1H, H^b-C8), 4.07 (d, $^4J_{HH}$ = 1.0 Hz, 2H, H-C9).

^{13}C NMR (126 MHz, $CDCl_3$) δ 132.7 (C3), 129.6 (C1), 127.9 (C7), 126.7 (C8), 123.8 (C2), 92.1 (C6), 88.8 (C5), 34.0 (C9).

GC-EI-MS: (*m/z*) requires: [($C_{11}H_8NO_2{}^{79}Br$)] = 264.9733, (*m/z*) found: [($C_{11}H_8NO_2{}^{79}Br$)] = 264.9734.

FT-IR ($\tilde{\nu}$ = cm^{-1}): 2972 (w), 1596 (m), 1518 (s), 1345 (s), 1286 (w), 1218 (w), 1108 (w), 855 (m), 750 (m), 687 (w).

Melting Point: 67-68 ˚C.

1-(3-(Chloromethyl)but-3-en-1-yn-1-yl)-4-nitrobenzene (S16)

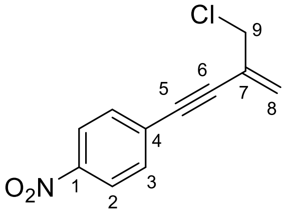

To a solution of 2-methylene-4-(4-nitrophenyl)but-3-yn-1-ol (**Int 1**) (304.8 mg, 1.50 mmol, 1.0 eq.) in MeCN (1 mL) at 0 °C was added PPh_3 (511.5 mg, 1.95 mmol, 1.3 eq.) and CCl_4 (1 mL). The solution was warmed to room temperature and stirred for 2 hours. The reaction mixture was directly purified by column chromatography (*n*-pentane:Et_2O 20:1) to yield the title compound as a yellow solid (265.9 mg, 1.20 mmol, 80%).

R*f* = 0.45 (*n*-pentane:Et_2O 20:1).

^{1}H NMR (500 MHz, $CDCl_3$) δ 8.23 – 8.18 (m, 2H, H-C2), 7.64 – 7.59 (m, 2H, H-C3), 5.79 – 5.77 (m, 1H, H^a-C8), 5.74 – 5.72 (m, 1H, H^b-C8), 4.19 – 4.16 (m, 2H, H-C9).

^{13}C NMR (126 MHz, $CDCl_3$) δ 132.6 (C3), 129.6 (C1), 127.5 (C7), 126.3 (C8), 123.8 (C2), 91.8 (C6), 88.9 (C5), 46.6 (C9).

GC-EI-MS: (*m/z*) requires: [($C_{11}H_8NO_2Cl$)] = 221.0238, (*m/z*) found: [($C_{11}H_8NO_2Cl$)] = 221.0239.

FT-IR ($\tilde{\nu}$ = cm^{-1}): 2972 (w), 1607 (w), 1591 (m), 1508 (s), 1491 (w), 1405 (w), 1373 (w), 1339 (s), 1326 (m), 1307 (m), 1286 (m), 1267 (m), 1108 (m), 924 (w), 875 (w), 847 (s), 749 (s), 715 (w), 696 (w), 686 (w).

Melting Point: 63-64 ˚C.

(1*R*,2*S*,5*R*)-2-Isopropyl-5-methylcyclohexyl 4-(3-methylbut-3-en-1-yn-1-yl)benzoate (S17)

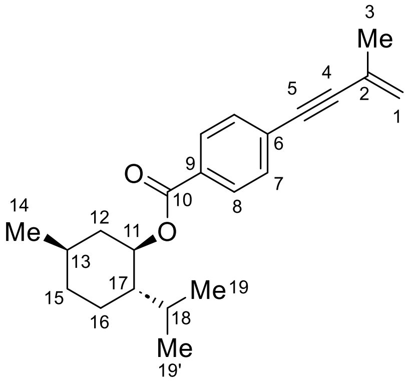

A mixture of methyl 4-(3-methylbut-3-en-1-yn-1-yl)benzoate (**S7**) (600.7 mg, 3.00 mmol, 1.0 eq.) and LiOH (431.1 mg, 18.00 mmol, 6.0 eq.) in THF (9 mL) and H_2O (9 mL) was vigorously stirred at 50 °C for 8 h. After the reaction was quenched with saturated aqueous NH_4Cl, the water layer was acidified with concentrated HCl (pH = 3) and extracted with DCM. The organic layer was dried with Na_2SO_4,

concentrated and the crude 4-(3-methylbut-3-en-1-yn-1-yl)benzoic acid was directly used in next step without further purification.

To a solution of crude 4-(3-methylbut-3-en-1-yn-1-yl)benzoic acid (186.2 mg, 1.00 mmol, 1.0 eq.) in DCM (5 mL) was added 1-ethyl-3-(3-(dimethylamino)propyl)-carbodiimide hydrochloride (EDCI) (287.6 mg, 1.50 mmol, 1.5 eq.), *N*,*N*-dimethylaminopyridine (12.2 mg, 0.10 mmol, 10 mol %) and then *L*-menthol (187.5 mg, 1.20 mmol, 1.2 eq.). The reaction mixture was stirred at room temperature for 12 h, before DCM was added. The mixture was washed with brine and dried over Na_2SO_4. After removal of the solvent, the residue was purified by column chromatography (*n*-pentane:Et_2O 40:1-30:1) to yield the title compound as a colorless oil (247.6 mg, 0.76 mmol, 76%).

R_f = 0.38 (*n*-pentane:Et_2O 40:1).

^{1}H NMR (500 MHz, $CDCl_3$) δ 8.00 – 7.96 (m, 2H, H-C8), 7.51 – 7.47 (m, 2H, H-C7), 5.44 (dq, $^2J_{HH}$ = 1.9, $^4J_{HH}$ = 1.0 Hz, 1H, H^a-C1), 5.35 (p, $^2J_{HH}$ = 1.7 Hz, $^4J_{HH}$ = 1.7 Hz, 1H, H^b-C1), 4.93 (td, $^3J_{HH}$ = 10.9, 4.4 Hz, 1H, H-C11), 2.17 – 2.08 (m, 1H, H-C12), 2.00 (dd, $^4J_{HH}$ = 1.6, 1.0 Hz, 3H, H-C3), 1.98 – 1.91 (m, 1H, H-C13), 1.77 – 1.73 (m, 1H, H-C16), 1.73 – 1.69 (m, 1H, H-C15), 1.61 – 1.56 (m, 1H, H-C18), 1.55 – 1.51 (m, 1H, H-C17), 1.18 – 1.05 (m, 2H, H-C12, H-C16), 0.97 – 0.88 (m, 7H, H-C15, H-C19, H-C19'), 0.79 (d, $^3J_{HH}$ = 7.0 Hz, 3H, H-C14).

^{13}C NMR (126 MHz, $CDCl_3$) δ 165.7 (C10), 131.6 (C7), 130.3 (C6), 129.6 (C8), 127.9 (C9), 126.7 (C2), 123.1 (C1), 93.5 (C4), 87.9 (C5), 75.2 (C11), 47.4 (C17), 41.1 (C12), 34.5 (C15), 31.6 (C18), 26.7 (C13), 23.8 (C16), 23.5 (C3), 22.2 (C19), 20.9 (C19'), 16.7 (C14).

GC-EI-MS: (*m/z*) requires: [($C_{22}H_{28}O_2$)] = 324.2084, (*m/z*) found: [($C_{22}H_{28}O_2$)] = 324.2084.

FT-IR ($\tilde{\nu}$ = cm^{-1}): 2956 (m), 2924 (m), 2870 (w), 1713 (s), 1614 (w), 1604 (w), 1455 (w), 1405 (w), 1372 (w), 1306 (m), 1287 (m), 1267 (s), 1241 (m), 1174 (m), 1115 (m), 1106 (m), 1095 (m), 1039 (w), 1017 (m), 981 (w), 961 (m), 905 (m), 857 (m), 768 (m), 732 (m), 695 (w).

(3*S*,5*S*,8*R*,9*S*,10*S*,13*R*,14*S*,17*R*)-10,13-Dimethyl-17-((*R*)-6-methylheptan-2-yl)hexadecahydro-1H-cyclopenta[a]phenanthren-3-yl 4-(3-methylbut-3-en-1-yn-1-yl)benzoate (S18)

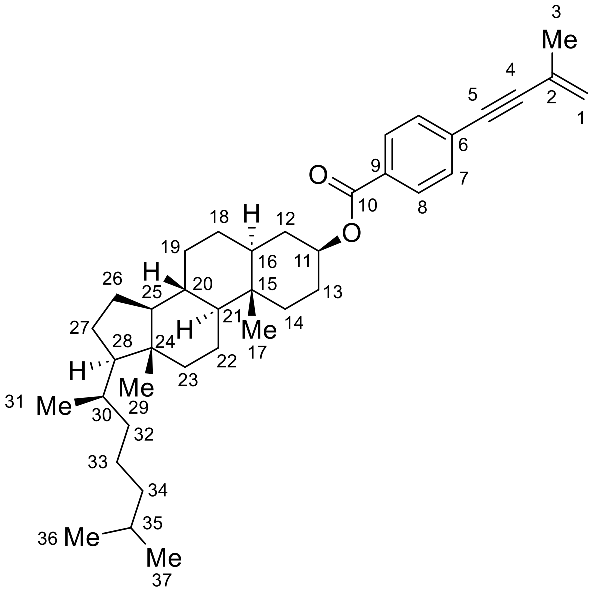

A mixture of methyl 4-(3-methylbut-3-en-1-yn-1-yl)benzoate (**S7**) (600.7 mg, 3.00 mmol, 1.0 eq.) and LiOH (431.1 mg, 18.00 mmol, 6.0 eq.) in THF (9 mL) and H_2O (9 mL) was vigorously stirred at 50 °C for 8 h. After the reaction was quenched with saturated aqueous NH_4Cl, the aqueous layer was acidified with concentrated HCl (pH = 3) and extracted with DCM. The organic layer was dried with Na_2SO_4, concentrated and the crude 4-(3-methylbut-3-en-1-yn-1-yl)benzoic acid was directly used in next step without further purification.

To a solution of crude 4-(3-methylbut-3-en-1-yn-1-yl)benzoic acid (186.2 mg, 1.00 mmol, 1.0 eq.) in DCM (5 mL) were added 1-ethyl-3-(3-(dimethylamino)propyl)-carbodiimide hydrochloride (EDCI) (287.6 mg, 1.50 mmol, 1.5 eq.), *N*,*N*-dimethylaminopyridine (12.2 mg, 0.10 mmol, 10 mol %) and then dihydrocholesterol (427.5 mg, 1.10 mmol, 1.1 eq.). The reaction mixture was stirred at room temperature for 12 h, before DCM was added. The mixture was washed with brine and dried over Na_2SO_4. After removal of the solvent, the residue was purified by column chromatography (*n*-pentane:Et_2O 30:1) to yield the title compound as a white solid (379.6 mg, 0.68 mmol, 68%).

R*f* = 0.48 (*n*-pentane:Et_2O 30:1).

^{1}H NMR (400 MHz, $CDCl_3$) δ 8.00 – 7.94 (m, 2H, H-C8), 7.53 – 7.43 (m, 2H, H-C7), 5.47 – 5.40 (m, 1H, H^a-C1), 5.38 – 5.31 (m, 1H, H^b-C1), 4.93 (tt, $^3J_{HH}$ = 11.3, 4.9 Hz, 1H, H-C11), 2.03 – 1.90 (m, 5H), 1.86 – 1.61 (m, 5H), 1.55 – 1.45 (m, 4H), 1.41 – 1.20 (m, 10H), 1.18 – 0.95 (m, 9H), 0.91 (d, J = 6.5 Hz, 3H), 0.89 – 0.84 (m, 9H), 0.72 – 0.63 (m, 4H).

^{13}C NMR (151 MHz, $CDCl_3$) δ 165.8 (C10), 131.5 (C7), 130.4 (C6), 129.5 (C8), 127.9 (C9),

126.7 (C2), 123.1 (C1), 93.4 (C4), 87.9 (C5), 74.8 (C11), 56.6 (*C*H), 56.4 (*C*H), 54.4 (*C*H), 44.9 (*C*H), 42.8 (*C*), 40.2 (CH_2), 39.7 (CH_2), 37.0 (CH_2), 36.3 (CH_2), 36.0 (*C*H), 35.7 (*C*), 35.7 (*C*H), 34.3 (CH_2), 32.2 (CH_2), 28.8 (CH_2), 28.4 (CH_2), 28.2 (*C*H), 27.7 (CH_2), 24.4 (CH_2), 24.0 (CH_2), 23.5 (CH_3), 23.0 (CH_3), 22.7 (CH_3), 21.4 (CH_2), 18.8 (CH_3), 12.5 (CH_3), 12.2 (CH_3).

ESI-MS: (*m/z*) requires: [$(C_{39}H_{56}O_2Na)^+$] = 579.4173, (*m/z*) found: [$(C_{39}H_{56}O_2Na)^+$] = 579.4177.

FT-IR ($\tilde{\nu}$ = cm^{-1}): 3671 (w), 2927 (s), 1706 (s), 1449 (w), 1406 (m), 1393 (m), 1382 (m), 1332 (w), 1280 (m), 1174 (w), 1066 (s), 1027 (m), 893 (w), 857 (w), 771 (m), 700 (w).

Melting Point: 177-179 ˚C.

(8*R*,9*S*,13*S*)-13-Methyl-17-oxo-7,8,9,11,12,13,14,15,16,17-decahydro-6H-cyclopenta[a]phenanthren-3-yl 4-(3-methylbut-3-en-1-yn-1-yl)benzoate (S19)

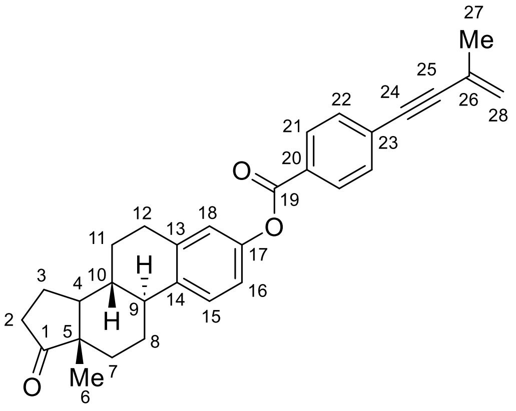

A mixture of methyl 4-(3-methylbut-3-en-1-yn-1-yl)benzoate (**S7**) (600.7 mg, 3.00 mmol, 1.0 eq.) and LiOH (431.1 mg, 18.00 mmol, 6.0 eq.) in THF (9 mL) and H_2O (9 mL) was vigorously stirred at 50 °C for 8 h. After the reaction was quenched with saturated aqueous NH_4Cl, the water layer was acidified with concentrated HCl (pH = 3) and extracted with DCM. The organic layer was dried with Na_2SO_4, concentrated and the crude 4-(3-methylbut-3-en-1-yn-1-yl)benzoic acid was directly used in next step without further purification.

To a solution of crude 4-(3-methylbut-3-en-1-yn-1-yl)benzoic acid (186.2 mg, 1.00 mmol, 1.0 eq.) in DCM (5 mL) was added 1-ethyl-3-(3-(dimethylamino)propyl)-carbodiimide hydrochloride (EDCI) (287.6 mg, 1.50 mmol, 1.5 eq.), *N*,*N*-dimethylaminopyridine (12.2 mg, 0.10 mmol, 10 mol %) and then estrone (297.4 mg, 1.10 mmol, 1.1 eq.). The reaction mixture was stirred at room temperature for 12 h, before DCM was added. The mixture was washed with brine and dried over Na_2SO_4. After removal of the solvent, the residue was purified by column chromatography (*n*-pentane:Et_2O 2.5:1-2:1) to yield the title compound as a white solid

(167.8 mg, 0.38 mmol, 38%).

R*f* = 0.49 (*n*-pentane:Et_2O 2:1).

^{1}H NMR (500 MHz, $CDCl_3$) δ 8.17 – 8.08 (m, 2H, H-C21), 7.59 – 7.51 (m, 2H, H-C22), 7.34 (d, $^3J_{HH}$ = 8.6 Hz, 1H, H-C15), 6.98 (dd, $^3J_{HH}$ = 8.5 Hz, $^4J_{HH}$ = 2.6 Hz, 1H, H-C16), 6.95 (d, $^4J_{HH}$ = 2.4 Hz, 1H, H-C18), 5.49 – 5.44 (m, 1H, H^a-C28), 5.39 – 5.36 (m, 1H, H^b-C28), 2.98 – 2.91 (m, 2H, H-C12), 2.56 – 2.47 (m, 1H, H^a-C2), 2.47 – 2.39 (m, 1H, H^a-C8), 2.32 (td, $^3J_{HH}$ = 10.9, 4.3 Hz, 1H, H-C9), 2.23 – 2.11 (m, 1H, H^b-C2), 2.11 – 1.94 (m, 6H, H-C27, H^a-C7, H^a-C11, H^a-C3), 1.67 – 1.47 (m, 6H, H-C4, H-C10, H^b-C8, H^b-C7, H^b-C11, H^b-C3), 0.93 (s, 3H, H-C6).

^{13}C NMR (126 MHz, $CDCl_3$) δ 220.9 (C1), 165.1 (C19), 148.9 (C17), 138.3 (C14), 137.7 (C13), 131.8 (C22), 130.1 (C21), 129.0 (C23), 128.8 (C20), 126.6 (C15), 126.6 (C26), 123.3 (C28), 121.8 (C18), 119.0 (C16), 94.1 (C25), 87.7 (C24), 50.6 (C4), 48.1 (C5), 44.3 (C9), 38.2 (C10), 36.0 (C2), 31.7 (C7), 29.6 (C12), 26.5 (C11), 25.9 (C8), 23.4 (C27), 21.8 (C3), 14.0 (C6).

ESI-MS: (*m/z*) requires: [$(C_{30}H_{30}O_3Na)^+$] = 461.2087, (*m/z*) found: [$(C_{30}H_{30}O_3Na)^+$] = 461.2085.

FT-IR ($\tilde{\nu}$ = cm^{-1}): 2954 (w), 2927 (w), 1729 (s), 1601 (m), 1494 (m), 1452 (w), 1405 (w), 1373 (w), 1263 (s), 1221 (m), 1210 (m), 1171 (m), 1152 (m), 1102 (w), 1072 (s), 1016 (w), 1007 (w), 908 (m), 858 (w), 765 (m), 732 (m), 693 (w).

Melting Point: 188-190 ˚C.

2-Methylhexadec-1-en-3-yne (S20)

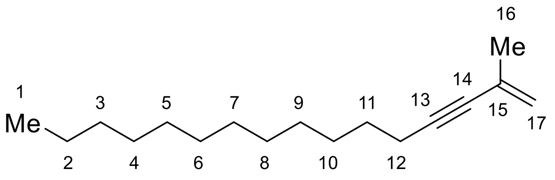

Compound **S20** was prepared according to General Procedure **A** using 1-tetradecyne (388.7 mg, 2.00 mmol, 1.0 eq.). The crude residue was purified by column chromatography (*n*-pentane) to yield the title compound as a colorless oil (448.5 mg, 1.91 mmol, 96%).

R*f* = 0.88 (*n*-pentane).

^{1}H NMR (599 MHz, $CDCl_3$) δ 5.21 – 5.18 (m, 1H, H^a-C17), 5.15 – 5.12 (m, 1H, H^b-C17), 2.29 (t, $^3J_{HH}$ = 7.2 Hz, 2H, H-C12), 1.90 – 1.85 (m, 3H, H-C16), 1.52 (p, $^3J_{HH}$ = 6.9 Hz, 2H, H-C11), 1.38 (p, $^3J_{HH}$ = 6.9 Hz, 2H, H-C10), 1.32 – 1.21 (m, 16H, H-C2~C9), 0.88 (t, $^3J_{HH}$ = 7.0 Hz, 3H, H-C1).

^{13}C NMR (151 MHz, $CDCl_3$) δ 127.5 (C15), 120.4 (C17), 89.7 (C13), 82.0 (C14), 32.1 (CH$_2$), 29.8 (CH$_2$), 29.8 (CH$_2$), 29.8 (CH$_2$), 29.7 (CH$_2$), 29.5 (CH$_2$), 29.3 (CH$_2$), 29.0 (C10), 28.9 (C11), 24.0 (C16), 22.8 (CH$_2$), 19.4 (C12), 14.3 (C1).

GC-EI-MS: (*m/z*) requires: [($C_{16}H_{27}$)] = 219.2107 [M-Me], (*m/z*) found: [($C_{16}H_{27}$)] = 219.2107.

FT-IR ($\tilde{\nu}$ = cm^{-1}): 2923 (s), 2854 (m), 1616 (w), 1466 (w), 1456 (w), 1372 (w), 1330 (w), 1289 (w), 890 (m), 722 (w).

(4-Methylpent-4-en-2-yn-1-yl)cyclohexane (S21)

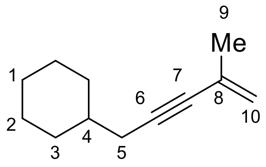

Compound **S21** was prepared according to General Procedure **A** using prop-2-ynylcyclohexane (488.9 mg, 4.00 mmol, 1.0 eq.). The crude residue was purified by column chromatography (*n*-pentane) to yield the title compound as a colorless oil (312.4 mg, 1.93 mmol, 48%).

R*f* = 0.87 (*n*-pentane).

^{1}H NMR (500 MHz, $CDCl_3$) δ 5.22 – 5.16 (m, 1H, H^a-C10), 5.15 – 5.10 (m, 1H, H^b-C10), 2.18 (d, $^3J_{HH}$ = 6.7 Hz, 2H, H-C5), 1.87 (dd, $^4J_{HH}$ = 1.6, 1.0 Hz, 3H, H-C9), 1.83 – 1.78 (m, 2H, H-C3), 1.74 – 1.69 (m, 2H, H-C2), 1.68 – 1.62 (m, 1H, H-C1), 1.50 – 1.44 (m, 1H, H-C4), 1.30 – 1.20 (m, 2H, H-C2), 1.19 – 1.09 (m, 1H, H-C1), 1.04 – 0.95 (m, 2H, H-C3).

^{13}C NMR (126 MHz, $CDCl_3$) δ 127.6 (C8), 120.3 (C10), 88.5 (C6), 82.9 (C7), 37.6 (C4), 32.9 (C3), 27.2 (C5), 26.5 (C1), 26.3 (C2), 24.1 (C9).

GC-EI-MS: (*m/z*) requires: [($C_{12}H_{18}$)] = 162.1403, (*m/z*) found: [($C_{12}H_{18}$)] = 162.1404.

FT-IR ($\tilde{\nu}$ = cm^{-1}): 2921 (s), 2851 (m), 2224 (w), 1679 (w), 1614 (w), 1449 (m), 1426 (w), 1372 (w), 1326 (w), 1291 (w), 1225 (w), 1032 (w), 890 (s), 844 (w).

2-(6-Methylhept-6-en-4-yn-1-yl)isoindoline-1,3-dione (S22)

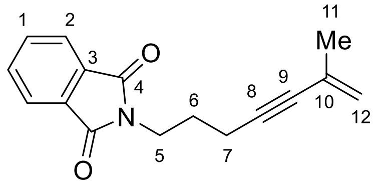

Compound **S22** was prepared according to General Procedure **A** using *N*-(pent-4-ynyl)phthalimide (426.5 mg, 2.00 mmol, 1.0 eq.). The crude residue was purified by column chromatography (*n*-pentane:Et_2O 4:1) to yield the title compound as a white solid (397.9 mg, 1.57 mmol, 79%).

R*f* = 0.30 (*n*-pentane:Et_2O 4:1).

^{1}H NMR (500 MHz, $CDCl_3$) δ 7.87 – 7.80 (m, 2H, H-C1), 7.73 – 7.67 (m, 2H, H-C2), 5.14 – 5.02 (m, 2H, H-C12), 3.80 (t, $^3J_{HH}$ = 7.0 Hz, 2H, H-C5), 2.38 (t, $^3J_{HH}$ = 7.0 Hz, 2H, H-C7), 1.94 (p, $^3J_{H\text{-}H}$ = 7.0 Hz, 2H, H-C6), 1.78 (t, $^4J_{HH}$ = 1.3 Hz, 3H, H-C11).

^{13}C NMR (126 MHz, $CDCl_3$) δ 168.5 (C4), 134.0 (C1), 132.3 (C3), 127.1 (C2), 123.4 (C10), 120.8 (C12), 87.8 (C8), 82.7 (C9), 37.5 (C5), 27.6 (C11), 23.7 (C7), 17.3 (C6).

GC-EI-MS: (*m/z*) requires: [($C_{16}H_{15}NO_2$)] = 253.1097, (*m/z*) found: [($C_{16}H_{15}NO_2$)] = 253.1103.

FT-IR ($\tilde{\nu}$ = cm^{-1}): 1769 (w), 1709 (m), 1438 (w), 1398 (m), 1373 (m), 1188 (w), 1115 (w), 1035 (m), 903 (m), 868 (w), 844 (w), 796 (w), 722 (s), 712 (s), 692 (w), 624 (w).

Melting Point: 72-74 ˚C.

13-Methyltetradec-13-en-11-yn-1-ol (S28)

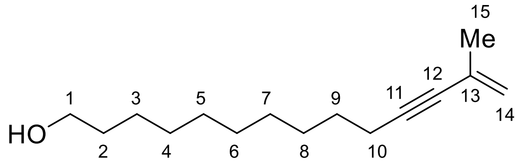

Compound **S28** was prepared according to General Procedure **A** using 11-dodecyn-1-ol (1.09 g, 6.00 mmol, 1.0 eq.). The crude residue was purified by column chromatography (*n*-pentane:Et_2O 2:1) to yield the title compound as a pale yellow oil (1.02 g, 4.59 mmol, 76%).

R*f* = 0.31 (*n*-pentane:Et_2O 2:1).

^{1}H NMR (500 MHz, $CDCl_3$) δ 5.20-5.17 (m, 1H, H^a-C14), 5.13-5.11 (m, 1H, H^b-C14), 3.63 (t, $^3J_{HH}$ = 7.0 Hz, 2H, H-C1), 2.28 (t, $^3J_{HH}$ = 7.0 Hz, 2H, H-C10), 1.86 (t, $^4J_{HH}$ = 1.3 Hz, 3H, H-

C15), 1.60 – 1.46 (m, 4H, H-C2, H-C9), 1.42 – 1.23 (m, 13H, H-C3~C8, OH).

^{13}C NMR (126 MHz, $CDCl_3$) δ 127.5 (C13), 120.4 (C14), 89.7 (C11), 82.0 (C12), 63.2 (C1), 33.0 (C2), 29.7 (C10), 29.6 (C3), 29.5 (C9), 29.2 (C4), 29.0 (C5), 28.9 (C6), 25.9 (C7), 24.0 (C8), 19.4 (C15).

ESI-MS: (*m/z*) requires: [$(C_{15}H_{26}ONa)^+$] = 245.1876, (*m/z*) found: [$(C_{15}H_{26}ONa)^+$] = 245.1875.

FT-IR ($\tilde{\nu}$ = cm^{-1}): 3333 (w), 2926 (s), 2854 (m), 1616 (w), 1456 (w), 1435 (w), 1372 (w), 1330 (w), 1289 (w), 1058 (w), 890 (m), 723 (w).

1,2-Difluoro-4-(((13-methyltetradec-13-en-11-yn-1-yl)oxy)methyl)benzene (S23)

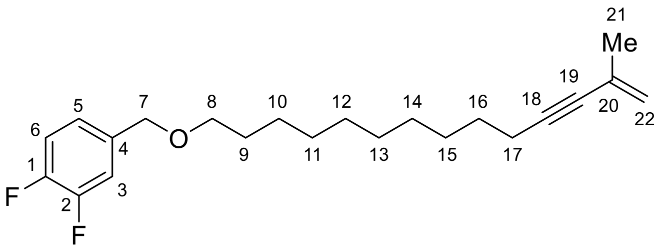

Under argon atmosphere, to a flame-dried Schlenk flask were added NaH (160.0 mg, 60% in mineral oil, 4.00 mmol, 2.0 eq.) and THF (4 mL). The mixture was cooled to 0 °C. 13-methyltetradec-13-en-11-yn-1-ol (**S28**) (444.7 mg, 2.00 mmol, dissolved in 1 mL THF, 1.0 eq.) was added dropwise. The reaction mixture was then stirred for 80 minutes. Tetrabutylammonium iodide (36.9 mg, 0.10 mmol, 5 mol%) and α-bromo-3,4-difluorotoluene (455.4 mg, 2.20 mmol, 1.1 eq.) were added. The cooling bath was removed and the reaction mixture was heated to 40 °C and stirred for 20 h. The reaction was cooled to room temperature. A saturated aqueous solution of NH_4Cl was added to quench the reaction. The aqueous layer was then extracted with diethyl ether and the combined organic layer was dried over anhydrous Na_2SO_4, filtered, and concentrated in vacuo. The residue was purified by column chromatography (*n*-pentane:Et_2O 80:1-60:1) to yield the title compound as a colorless oil (424.9 mg, 1.22 mmol, 61%).

R_f = 0.44 (*n*-pentane:Et_2O 90:1).

^{1}H NMR (599 MHz, $CDCl_3$) δ 7.16 (ddd, $^3J_{HF}$ = 11.1, $^4J_{HF}$ = 7.7, $^4J_{HH}$ = 2.1 Hz, 1H, H-C3), 7.11 (ddd, $^3J_{HF}$ = 10.2, $^3J_{HH}$ = 8.2 Hz, $^4J_{HF}$ = 8.2 Hz, 1H, H-C6), 7.05 – 7.01 (m, 1H, H-C5), 5.20 – 5.17 (m, 1H, H^a-C22), 5.14 – 5.11 (m, 1H, H^b-C22), 4.43 (s, 2H, H-C7), 3.45 (t, $^3J_{HH}$ =

6.6 Hz, 2H, H-C8), 2.28 (t, $^3J_{HH}$ = 7.2 Hz, 2H, H-C17), 1.87 (t, $^4J_{HH}$ = 1.3 Hz, 3H, H-C21), 1.61 (p, $^3J_{HH}$ = 6.9 Hz, 2H, H-C9), 1.54 – 1.49 (m, 2H, H-C16), 1.42 – 1.33 (m, 4H, H-C10, H-C15), 1.32 – 1.25 (m, 8H, H-C11~C14).

^{13}C NMR (151 MHz, $CDCl_3$) δ 150.5 (dd, $^1J_{CF}$ = 98.7 Hz, $^2J_{CF}$ = 12.7 Hz, *C*F), 149.8 (dd, $^1J_{CF}$ = 97.9 Hz, $^2J_{CF}$ = 12.7 Hz, *C*F), 136.0 (dd, $^3J_{CF}$ = 5.4 Hz, $^4J_{CF}$ = 3.9 Hz, C4), 127.5 (C20), 123.4 (dd, $^3J_{CF}$ = 6.2 Hz, $^4J_{CF}$ = 3.6 Hz, C5), 120.4 (C22), 117.2 (d, $^2J_{CF}$ = 17.2 Hz, C6), 116.5 (d, $^2J_{CF}$ = 17.4 Hz, C3), 89.7 (C18), 81.97 (C19), 71.8 (d, $^4J_{CF}$ = 1.4 Hz, C7), 70.9 (C8), 29.6 (C9), 29.7 (*C*H_2), 29.6 (*C*H_2), 29.6 (*C*H_2), 29.3 (*C*H_2), 29.0 (C15), 28.9 (C16), 26.3 (C10), 24.0 (C21), 19.4 (C17).

^{19}F NMR (564 MHz, $CDCl_3$) δ -138.10 (dddd, $^3J_{FF}$ = 20.6, $^3J_{HF}$ = 11.1, $^4J_{HF}$ = 8.0, $^5J_{HF}$ = 1.3 Hz, 1F, F-C2), -139.89 – -140.06 (m, 1F, F-C1).

^{19}F{^{1}H} NMR (564 MHz, $CDCl_3$) δ -138.10 (d, $^3J_{FF}$ = 21.1 Hz, F-C2), -139.97 (d, $^3J_{FF}$ = 21.1 Hz, F-C1).

GC-EI-MS: (*m/z*) requires: [($C_{22}H_{30}OF_2$)] = 348.2259, (*m/z*) found: [($C_{22}H_{30}OF_2$)] = 348.2251.

FT-IR ($\tilde{\nu}$ = cm^{-1}): 2927 (m), 2855 (m), 1611 (w), 1518 (s), 1456 (w), 1433 (m), 1360 (w), 1286 (s), 1208 (w), 1145 (m), 1113 (s), 1099 (s), 954 (w), 941 (w), 891 (m), 872 (w), 816 (m), 779 (m), 748 (w).

(4-Methylpent-4-en-2-yn-1-yl)benzene (S24)

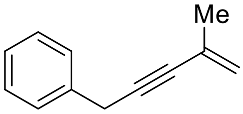

Compound **S24** was prepared according to General Procedure **A** using propargyl benzene (464.6 mg, 4.00 mmol, 1.0 eq.). The crude residue was purified by column chromatography (*n*-pentane) to yield the title compound as a colorless oil (463.0 mg, 2.96 mmol, 74%).

R*f* = 0.33 (*n*-pentane).

^{1}H NMR (400 MHz, $CDCl_3$) δ 7.38 – 7.29 (m, 4H), 7.25 – 7.20 (m, 1H), 5.29 – 5.26 (m, 1H), 5.22 – 5.17 (m, 1H), 3.73 (s, 2H), 1.91 (t, *J* = 1.3 Hz, 3H).

GC-EI-MS: (*m/z*) requires: [($C_{12}H_{18}$)] = 156.0934, (*m/z*) found: [($C_{12}H_{18}$)] = 156.0934.

Analytical data is in agreement with literature values.[11]

13-Methyltetradec-13-en-11-yn-1-yl (*E*)-3-(4-bromophenyl)acrylate (S25)

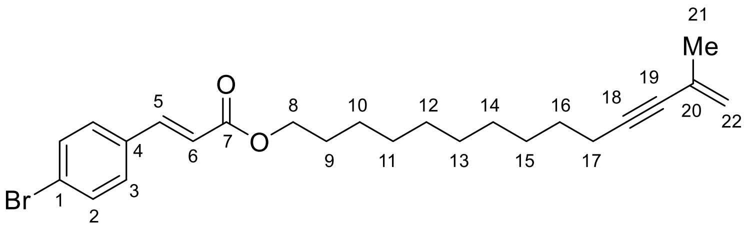

To a solution of 4-bromocinnamic acid (249.8 mg, 1.10 mmol, 1.1 eq.) in DCM (2 mL) were added 1-ethyl-3-(3-(dimethylamino)propyl)-carbodiimide hydrochloride (EDCI) (287.6 mg, 1.50 mmol, 1.5 eq.), *N*,*N*-dimethylaminopyridine (12.2 mg, 0.10 mmol, 10 mol %) and then 13-methyltetradec-13-en-11-yn-1-ol (**S28**) (227.4 mg, 1.00 mmol, 1.0 eq.). The reaction mixture was stirred at room temperature for 12 h. DCM was added. The mixture was washed with brine and dried over Na_2SO_4. After removal of the solvent, the residue was purified by column chromatography (*n*-pentane:Et_2O 20:1) to yield the title compound as a colorless oil (393.6 mg, 0.91 mmol, 91%).

$\mathbf{R}_f$ = 0.40 (*n*-pentane:Et_2O 20:1).

^{1}H NMR (400 MHz, $CDCl_3$) δ 7.61 (d, $^3J_{HH}$ = 16.0 Hz, 1H, H-C5), 7.52 (d, $^3J_{HH}$ = 8.5 Hz, 2H, H-C2), 7.39 (d, $^3J_{HH}$ = 8.5 Hz, 2H, H-C3), 6.42 (d, $^3J_{HH}$ = 16.0 Hz, 1H, H-C6), 5.22 – 5.16 (m, 1H, H^a-C22), 5.15 – 5.10 (m, 1H, H^b-C22), 4.20 (t, $^3J_{HH}$ = 6.7 Hz, 2H, H-C8), 2.28 (t, $^3J_{HH}$ = 7.1 Hz, 2H, H-C17), 1.87 (t, $^4J_{HH}$ = 1.3 Hz, 3H), 1.76 – 1.64 (m, 2H, H-C9), 1.55 – 1.47 (m, 2H, H-C16), 1.44 – 1.25 (m, 12H, H-C10~C15).

^{13}C NMR (126 MHz, $CDCl_3$) δ 167.0 (C7), 143.3 (C5), 133.6 (C1), 132.3 (C2), 129.6 (C3), 127.5 (C20), 124.6 (C4), 120.4 (C22), 119.2 (C6), 89.6 (C18), 82.0 (C19), 65.0 (C8), 29.6 (CH_2), 30.0 (CH_2), 29.4 (CH_2), 29.2 (CH_2), 29.0 (C15), 28.9 (C16), 28.9 (C9), 26.1 (C10), 24.0 (C21), 19.4 (C17).

ESI-MS: (*m/z*) requires: [$(C_{24}H_{31}O_2{}^{79}BrNa)^+$] = 453.1400, (*m/z*) found: [$(C_{24}H_{31}O_2{}^{79}BrNa)^+$] = 453.1398.

FT-IR ($\tilde{\nu}$ = cm^{-1}): 2924 (m), 2854 (w), 1713 (s), 1637 (m), 1587 (w), 1488 (m), 1402 (w), 1309 (m), 1268 (m), 1201 (m), 1181 (m), 1168 (s), 1108 (w), 1072 (s), 1009 (m), 981 (m), 891 (w), 818 (s), 723 (w).

13-Methyltetradec-13-en-11-yn-1-yl hex-2-ynoate (S26)

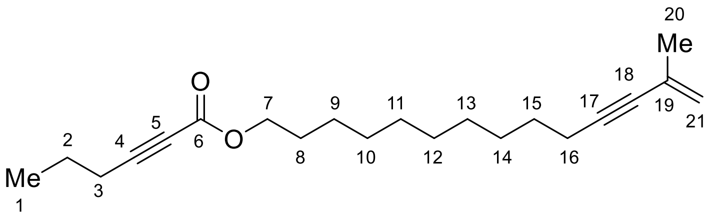

To a solution of hex-2-ynoic acid (185.0 mg, 1.65 mmol, 1.1 eq.) in DCM (3 mL) were added 1-ethyl-3-(3-(dimethylamino)propyl)-carbodiimide hydrochloride (EDCI) (431.3 mg, 2.25 mmol, 1.5 eq.), *N*,*N*-dimethylaminopyridine (18.3 mg, 0.15 mmol, 10 mol%) and then 13-methyltetradec-13-en-11-yn-1-ol (**S28**) (333.6 mg, 1.50 mmol, 1.0 eq.). The reaction mixture was stirred at room temperature for 12 h. DCM was added. The mixture was washed with brine and dried over Na_2SO_4. After removal of the solvent, the residue was purified by column chromatography (*n*-pentane:Et_2O 20:1) to yield the title compound as a colorless oil (44.0 mg, 0.14 mmol, 9%).

$\mathbf{R}_{\boldsymbol{f}}$ = 0.48 (*n*-pentane:Et_2O 20:1).

^{1}**H NMR** (500 MHz, $CDCl_3$) δ 5.21 – 5.17 (m, 1H, H^a-C21), 5.14 – 5.11 (m, 1H, H^b-C21), 4.14 (t, $^{3}J_{HH}$ = 6.8 Hz, 2H, H-C7), 2.35 – 2.24 (m, 4H, H-C3, H-C16), 1.87 (dd, $^{4}J_{HH}$ = 1.5, 1.0 Hz, 3H, H-C20), 1.69 – 1.57 (m, 4H, H-C8, H-C2), 1.55 – 1.48 (m, 2H, H-C15), 1.41 – 1.26 (m, 12H, H-C9~C14), 1.01 (t, $^{3}J_{HH}$ = 7.4 Hz, 3H, H-C1).

^{13}**C NMR** (126 MHz, $CDCl_3$) δ 154.2 (C6), 127.5 (C19), 120.4 (C21), 89.6 (C17), 89.4 (C4), 82.0 (C18), 73.5 (C5), 66.1 (C7), 29.6 (*C*H_2), 29.5 (*C*H_2), 29.3 (*C*H_2), 29.2 (*C*H_2), 29.0 (C14), 28.9 (C15), 28.6 (C8), 26.0 (*C*H_2), 24.0 (C20), 21.2 (C2), 20.8 (C3), 19.4 (C16), 13.6 (C1).

GC-EI-MS: (*m/z*) requires: [($C_{20}H_{29}O_2$)] = 301.2168 [M-Me], (*m/z*) found: [($C_{20}H_{29}O_2$)] = 301.2164.

FT-IR ($\tilde{\nu}$ = cm^{-1}): 2929 (m), 2855 (w), 2234 (w), 1709 (s), 1616 (w), 1372 (w), 1327 (w), 1246 (s), 1082 (m), 1025 (w), 891 (w), 804 (w), 752 (m), 723 (w).

13-Methyltetradec-13-en-11-yn-1-yl 4-methylbenzenesulfonate (S27)

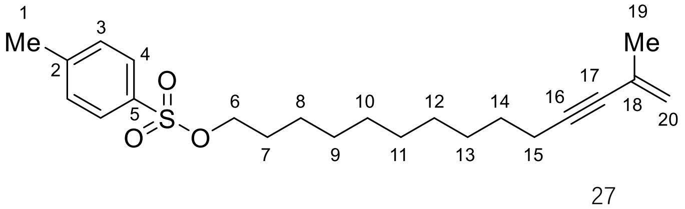

Under argon atmosphere, to a flame-dried Schlenk flask was added 13-methyltetradec-13-en-11-yn-1-ol (**S28**)

(333.6 mg, 1.50 mmol, 1.0 eq.) and DCM (3 mL). The mixture was cooled to 0 °C. DMAP (18.3 mg, 0.15 mmol, 10 mol%), NEt_3 (227.7 mg, 2.25 mmol, 1.5 eq.) and TsCl (429.0 mg, 2.25 mmol, 1.5 eq.) were added successively. The reaction mixture was warmed to room temperature and stirred for 24 h. After removal of the solvent, the residue was directly purified by column chromatography (*n*-pentane:Et_2O 10:1) to yield the title compound as a colorless oil (453.8 mg, 1.21 mmol, 80%).

R*f* = 0.34 (*n*-pentane:Et_2O 10:1).

^{1}H NMR (599 MHz, $CDCl_3$) δ 7.79 (d, $^3J_{HH}$ = 8.3 Hz, 2H, H-C4), 7.34 (d, $^3J_{HH}$ = 7.9 Hz, 2H, H-C3), 5.21 – 5.17 (m, 1H, H^a-C20), 5.14 – 5.11 (m, 1H, H^b-C20), 4.02 (t, $^3J_{HH}$ = 6.5 Hz, 2H, H-C6), 2.45 (s, 3H, H-C1), 2.28 (t, $^3J_{HH}$ = 7.1 Hz, 2H, H-C15), 1.87 (t, $^4J_{HH}$ = 1.2 Hz, 3H, H-C19), 1.66 – 1.60 (m, 2H, H-C7), 1.51 (p, *J* = 7.2 Hz, 2H, H-C14), 1.37 (p, *J* = 6.8 Hz, 2H, H-C13), 1.32 – 1.19 (m, 10H, H-C8~C12).

^{13}C NMR (151 MHz, $CDCl_3$) δ 144.7 (C5), 133.5 (C2), 129.9 (C3), 128.0 (C4), 127.5 (C18), 120.4 (C20), 89.6 (C16), 82.0 (C17), 70.8 (C6), 29.5 (CH_2), 29.5 (CH_2), 29.2 (CH_2), 29.0 (CH_2), 29.0 (CH_2), 29.0 (CH_2), 28.9 (C14), 25.5 (CH_2), 24.0 (C19), 21.8 (C1), 19.4 (C15).

ESI-MS: (*m/z*) requires: [$(C_{22}H_{32}O_3SNa)^+$] = 399.1964, (*m/z*) found: [$(C_{22}H_{32}O_3SNa)^+$] = 399.1972.

FT-IR ($\tilde{\nu}$ = cm^{-1}): 2926 (m), 2855 (w), 1614 (w), 1598 (w), 1456 (w), 1360 (m), 1290 (w), 1188 (m), 1175 (s), 1098 (w), 1020 (w), 957 (m), 924 (m), 893 (m), 814 (m), 772 (w), 723 (w), 689 (w), 663 (s).

2-Methyl-3-methyleneoctadec-4-yne (S29)

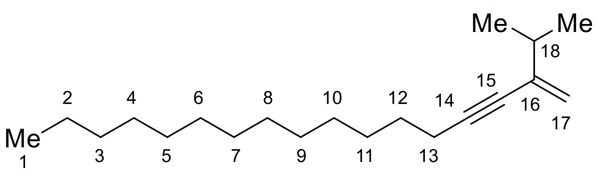

Compound **S29** was prepared according to General Procedure **D** using pentadec-1-yne (1.04 g, 5.00 mmol, 1.0 eq.). The crude residue was purified by column chromatography (*n*-pentane) to yield the title compound as a colorless oil (462.2 mg, 1.67 mmol, 33%).

R$_f$ = 0.89 (*n*-pentane).

^{1}H NMR (500 MHz, $CDCl_3$) δ 5.16 (d, $^2J_{HH}$ = 2.0 Hz, 1H, H^a-C17), 5.13 (dd, $^2J_{HH}$ = 2.0, $^4J_{HH}$ = 1.1 Hz, 1H, H^b-C17), 2.42 – 2.34 (m, 1H, H-C18), 2.31 (t, $^3J_{HH}$ = 7.1 Hz, 2H, H-C13), 1.56 – 1.51 (m, 2H, H-C12), 1.45 – 1.36 (m, 2H, H-C11), 1.32 – 1.24 (m, 18H, H-C2~C10), 1.08 (d, $^3J_{HH}$ = 6.8 Hz, 6H, H-C19), 0.88 (t, $^3J_{HH}$ = 7.0 Hz, 3H, H-C1).

^{13}C NMR (126 MHz, $CDCl_3$) δ 139.0 (C16), 117.2 (C17), 91.1 (C14), 79.9 (C15), 35.7 (C18), 32.1 (CH_2), 29.8 (CH_2), 29.8 (CH_2), 29.8 (CH_2), 29.8 (CH_2), 29.7 (CH_2), 29.5 (CH_2), 29.3 (CH_2), 29.0 (C11), 29.0 (C12), 22.9 (CH_2), 21.8 (C19), 19.5 (C13), 14.3 (C1).

GC-EI-MS: (*m/z*) requires: [($C_{20}H_{36}$)] = 276.2812, (*m/z*) found: [($C_{20}H_{36}$)] = 276.2815.

FT-IR ($\tilde{\nu}$ = cm^{-1}): 2962 (m), 2923 (s), 2854 (m), 1610 (w), 1466 (w), 1393 (w), 1379 (w), 1362 (w), 1066 (w), 891 (m), 722 (w).

Heptadec-1-en-3-yn-2-ylcyclohexane (S30)

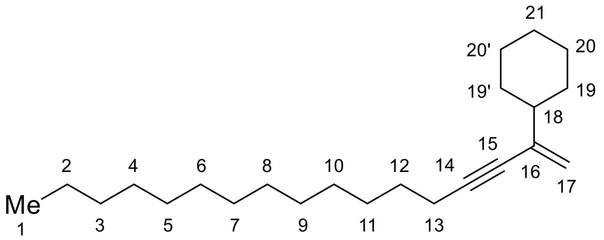

Compound **S30** was prepared according to General Procedure **D** using pentadec-1-yne (1.04 g, 5.00 mmol, 1.0 eq.). The crude residue was purified by column chromatography (*n*-pentane) to yield the title compound as a colorless oil (492.9 mg, 1.56 mmol, 31%).

R$_f$ = 0.64 (*n*-pentane).

^{1}H NMR (500 MHz, $CDCl_3$) δ 5.19 – 5.16 (m, 1H, H^a-C17), 5.13 – 5.10 (m, 1H, H^b-C17), 2.31 (t, $^3J_{HH}$ = 7.0 Hz, 2H, H-C13), 2.05 – 1.96 (m, 1H, H-C18), 1.81 – 1.72 (m, 4H, H-C19, H-C20), 1.70 – 1.63 (m, 1H, H^a-C21), 1.57 – 1.50 (m, 2H, H-C12), 1.41 (p, $^3J_{HH}$ = 6.9 Hz, 2H, H-C11), 1.33 – 1.22 (m, 22H, H-C2~C10, H-C19', H-C20'), 1.21 – 1.11 (m, 1H, H^b-C21), 0.88 (t, $^3J_{HH}$ = 6.9 Hz, 3H, H-C1).

^{13}C NMR (126 MHz, $CDCl_3$) δ 138.2 (C16), 117.5 (C17), 90.8 (C14), 80.5 (C15), 45.4 (C18), 32.1 (C19, C19'), 32.1 (CH_2), 29.8 (CH_2), 29.8 (CH_2), 29.8 (CH_2), 29.7 (CH_2), 29.5 (CH_2), 29.3

(CH_2), 29.0 (C11), 29.0 (C12), 26.4 (C20, C20'), 26.2 (C21), 22.9 (CH_2), 19.5 (C13), 14.3 (C1).

GC-EI-MS: (*m/z*) requires: [($C_{23}H_{40}$)] = 316.3125, (*m/z*) found: [($C_{23}H_{40}$)] = 316.3124.

FT-IR ($\tilde{\nu}$ = cm^{-1}): 2923 (s), 2853 (s), 1609 (w), 1466 (w), 1451 (w), 1066 (w), 888 (m), 722 (w).

2-(Fluoromethyl)hexadec-1-en-3-yne (S31)

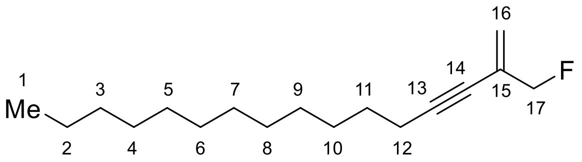

To a solution of 1-tetradecyne (971.8 mg, 5.00 mmol, 1.0 eq.) in anhydrous THF (0.25 M) at –78 °C, *n*-BuLi (1.6 M in *n*-hexane, 1.2 eq.) was added dropwise. The reaction mixture was stirred at –78 °C for 3 h. Then, ethyl 2-fluoroacetate (636.6 mg, 6.00 mmol, 1.2 eq.) and boron trifluoride etherate (851.6 mg, 6.00 mmol, 1.2 eq.) were slowly added, and the obtained reaction mixture was stirred at the same temperature for 2 h. After quenching with saturated aqueous ammonium chloride, the mixture was extracted with Et_2O, and the combined organic layer was washed with brine, then dried over anhydrous Na_2SO_4, filtrated through a small pad of silica gel. The solvent was removed under reduced pressure with the aid of a rotary evaporator to give the crude product 1-fluorohexadec-3-yn-2-one, which was used without any further purification for the next step.

A flame-dried Schlenk-flask charged with methyltriphenylphosphonium bromide (346.5 mg, 0.97 mmol, 2.0 eq.) was evacuated and backfilled with Ar 3 times before dry THF (5 mL) was added. Potassium *tert*-butoxide (108.8 mg, 0.97 mmol, 2.0 eq.) was added to the suspension at 0 °C (ice bath) and a bright yellow color was observed. The mixture was stirred at 0 °C for 30 min. The crude product 1-fluorohexadec-3-yn-2-one (123.2 mg, 0.48 mmol, 1.0 eq.) was subsequently added. The reaction mixture was stirred at 0 °C for 3 h then allowed to warm up to room temperature. The stirring was continued for an additional 15 h. After the reaction was complete, the reaction mixture was diluted with Et_2O and filtered through a small pad of silica gel. The solvent was removed under reduced pressure with the aid of a rotary evaporator and the crude residue was purified by column chromatography to yield the title compound as a colorless oil (71.3 mg, 0.28 mmol, 58%).

$\mathbf{R}_f$ = 0.66 (*n*-pentane).

^{1}H NMR (500 MHz, $CDCl_3$) δ 5.50 – 5.48 (m, 1H, H^a-C16), 5.47 – 5.45 (m, 1H, H^b-C16), 4.78 (dt, $^2J_{HF}$ = 46.9 Hz, $^4J_{HH}$ = 1.4 Hz, 2H, H-C17), 2.30 (t, $^3J_{HH}$ = 7.2 Hz, 2H, H-C12), 1.54 – 1.50 (m, 2H, H-C11), 1.42 – 1.34 (m, 2H, H-C10), 1.32 – 1.25 (m, 16H, H-C2~C9), 0.88 (t, $^3J_{HH}$ = 6.9 Hz, 3H, H-C1).

^{13}C NMR (126 MHz, $CDCl_3$) δ 127.6 (d, $^2J_{CF}$ = 18.9 Hz, C15), 120.6 (d, $^3J_{CF}$ = 8.2 Hz, C16), 110.2 (C14), 92.9 (C13), 84.0 (d, $^1J_{CF}$ = 174.8 Hz, C17), 32.1 (CH_2), 29.8 (CH_2), 29.8 (CH_2), 29.8 (CH_2), 29.7 (CH_2), 29.5 (CH_2), 29.3 (CH_2), 29.0 (C10), 28.7 (C11), 22.8 (CH_2), 19.4 (C12), 14.3 (C1).

^{19}F NMR (470 MHz, $CDCl_3$) δ -214.63 (tt, $^2J_{HF}$ = 46.8 Hz, $^4J_{HF}$ = 1.9 Hz, 1F, F-C17).

^{19}F{^{1}H} NMR (470 MHz, $CDCl_3$) δ -214.63 (s, 1F, F-C17).

ESI-MS: (*m/z*) requires: [$(C_{17}H_{29}FAg)^+$] = 359.1299, (*m/z*) found: [$(C_{17}H_{29}FAg)^+$] = 359.1308.

FT-IR ($\tilde{\nu}$ = cm^{-1}): 2924 (s), 2854 (m), 1466 (w), 1393 (w), 1379 (w), 1231 (w), 1066 (m), 904 (m).

13-Methyltetradec-13-en-11-yn-1-yl-2-(3-cyano-4-isobutoxyphenyl)-4-methylthiazole-5-carboxylate (S32)

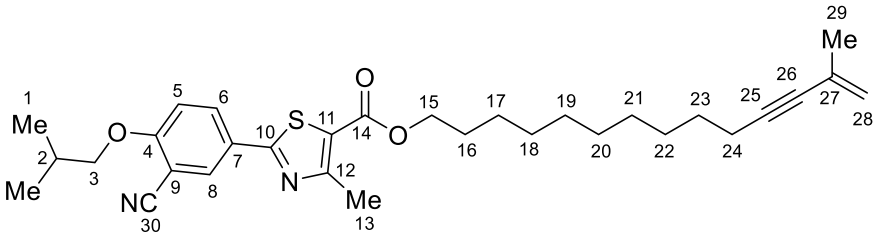

To a solution of Febuxostat (348.0 mg, 1.10 mmol, 1.1 eq.) in DCM (2 mL) was added 1-ethyl-3-(3-(dimethylamino)propyl)-carbodiimide hydrochloride (EDCI) (287.6 mg, 1.50 mmol, 1.5 eq.), *N,N*-dimethylaminopyridine (12.2 mg, 0.10 mmol, 10 mol %) and then 13-methyltetradec-13-en-11-yn-1-ol (**S28**) (227.4 mg, 1.00 mmol, 1.0 eq.). The reaction mixture was stirred at room temperature for 12 h. DCM was added. The mixture was washed with brine and dried over Na_2SO_4. After removal of the solvent, the residue was purified by column chromatography (*n*-

pentane:Et_2O 3:1) to yield the title compound as a waxy solid (332.8 mg, 0.64 mmol, 64%).

R*f* = 0.45 (*n*-pentane:Et_2O 3:1).

^{1}H NMR (500 MHz, $CDCl_3$) δ 8.17 (d, $^4J_{HH}$ = 2.3 Hz, 1H, H-C8), 8.09 (dd, $^3J_{HH}$ = 8.8 Hz, $^4J_{HH}$ = 2.3 Hz, 1H, H-C6), 7.00 (d, $^3J_{HH}$ = 8.9 Hz, 1H, H-C5), 5.20 – 5.17 (m, 1H, H^a-C28), 5.13 – 5.10 (m, 1H, H^b-C28), 4.28 (t, $^3J_{HH}$ = 6.6 Hz, 2H, H-C15), 3.89 (d, $^3J_{HH}$ = 6.5 Hz, 2H, H-C3), 2.76 (s, 3H, H-C13), 2.28 (t, $^3J_{HH}$ = 7.1 Hz, 2H, H-C24), 2.25 – 2.14 (m, 1H, H-C2), 1.86 (t, $^4J_{HH}$ = 1.3 Hz, 3H, H-C29), 1.79 – 1.69 (m, 2H, H-C16), 1.52 (p, $^3J_{HH}$ = 7.2 Hz, 2H, H-C23), 1.46 – 1.27 (m, 12H, H-C17~C22), 1.09 (d, $^3J_{HH}$ = 6.7 Hz, 6H, H-C1).

^{13}C NMR (126 MHz, $CDCl_3$) δ 167.3 (C10), 162.6 (C4), 162.3 (C14), 161.2 (C12), 132.7 (C6), 132.2 (C8), 127.5 (C27), 126.2 (C7), 122.2 (C11), 120.4 (C28), 115.5 (C30), 112.8 (C5), 103.1 (C9), 89.6 (C25), 82.0 (C26), 75.8 (C3), 65.6 (C15), 29.6 (CH_2), 29.6 (CH_2), 29.3 (CH_2), 29.2 (CH_2), 29.0 (CH_2), 28.9 (C23), 28.8 (C16), 28.3 (C2), 26.1 (CH_2), 24.0 (C29), 19.4 (C24), 19.2 (C1), 17.6 (C13).

ESI-MS: (*m/z*) requires: [$(C_{31}H_{40}N_2O_3SNa)^+$] = 543.2652, (*m/z*) found: [$(C_{31}H_{40}N_2O_3SNa)^+$] = 543.2651.

FT-IR ($\tilde{\nu}$ = cm^{-1}): 2927 (m), 2855 (w), 2228 (w), 1710 (m), 1607 (m), 1508 (m), 1468 (w), 1451 (m), 1432 (m), 1389 (m), 1372 (m), 1329 (m), 1296 (s), 1258 (s), 1171 (w), 1129 (m), 1092 (s), 1045 (m), 1013 (m), 960 (w), 890 (m), 828 (w), 761 (w), 733 (w), 725 (w), 659 (w), 640 (w).

2-bromooct-1-ene (Int 2)

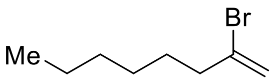

1-Octyne (1.48 mL, 10.00 mmol, 1.0 eq.) was added dropwise to a solution of boron tribromide (1M in DCM, 5.0 mL, 5.00 mmol, 0.5 eq.) at -78 °C under an argon atmosphere. The mixture was stirred at this temperature for one hour and then at room temperature for a further two hours. After this time, acetic acid (17.2 mL, 300 mmol, 30 eq.) was slowly added and the solution was stirred for one hour at room temperature. The reaction was quenched through slow addition to a stirred solution of excess sodium bicarbonate in water (caution, rapid generation of CO_2). The mixture was extracted using DCM,

and the aqueous layer was back-washed with two additional portions. The combined organic layers were washed with brine, dried over sodium sulfate, filtered and concentrated under reduced pressure. Purification of the crude material by column chromatography (*n*-pentane) afforded the title compound as a yellow oil (1.42 g, 7.45 mmol, 75%).

R*f* = 0.81 (*n*-pentane).

^{1}H NMR (400 MHz, $CDCl_3$) δ 5.55 (dd, *J* = 1.5, 1.3 Hz, 1H), 5.38 (d, *J* = 1.5 Hz, 1H), 2.41 (td, *J* = 7.4, 1.2 Hz, 2H), 1.61 – 1.51 (m, 2H), 1.37 – 1.23 (m, 6H), 0.96 – 0.85 (m, 3H).

^{13}C NMR (101 MHz, $CDCl_3$) δ 135.12, 116.32, 41.58, 31.67, 28.23, 28.01, 22.70, 14.19.

EI MS (*m/z*) requires: [($C_8H_{15}{}^{79}Br$)] = 190.04, (*m/z*) found: [($C_8H_{15}{}^{79}Br$)] = 190.05.

Analytical data is in agreement with literature values.[12]

Triisopropyl(3-methylenenon-1-yn-1-yl)silane (S33)

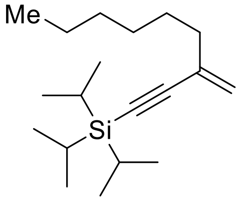

Compound **S33** was prepared according to General Procedure **A** using 2-bromooct-1-ene (**Int 2**) (1.90 g, 10.0 mmol, 1.0 equiv.) and triisopropylacetylene (2.69 mL, 12.0 mmol, 1.2 equiv.). The crude residue was purified by column chromatography (*n*-pentane) to afford the title compound as a yellow liquid (1.97 g, 6.73 mmol, 67%).

R*f* = 0.76 (*n*-pentane).

^{1}H NMR (400 MHz, $CDCl_3$) δ 5.34 (d, *J* = 2.0 Hz, 1H), 5.22 (dd, *J* = 2.2, 1.3 Hz, 1H), 2.15 (t, *J* = 7.5 Hz, 2H), 1.62 – 1.49 (m, 2H), 1.38 – 1.22 (m, 6H), 1.14 – 1.04 (m, 21H), 0.91 – 0.85 (m, 3H).

GC-EI-MS (*m/z*) requires: [($C_{19}H_{36}Si$)] = 292.26, (*m/z*) found: [($C_{11}H_{18}O$)] = 292.27.

Analytical data is in agreement with literature values.[13]

1.3 Preparation and characterisation of homopropargylic fluorides

General Procedure E for 1,1-difluorination of enynes

Unless otherwise stated, a Teflon® vial was equipped with a 1 cm stirring bar followed by the addition of enyne (0.2 mmol, 1.0 eq.), *p*-iodotoluene (9 mg, 0.04 mmol, 20 mol%) and $CHCl_3$ (0.5 mL). The stated amine:HF mixture was added (0.5 mL) via syringe. After stirring for 1 min, Selectfluor® (106 mg, 0.3 mmol, 1.5 eq.) was added in one portion. The reaction vessel was then sealed with a Teflon® screw cap. After stirring (350 rpm) at ambient temperature for 24 h, the reaction mixture was poured into 100 mL of a saturated solution of $NaHCO_3$ (CAUTION, generation of CO_2!). The Teflon® vial was rinsed with DCM and dropped into another flask of saturated aqueous solution of $NaHCO_3$ to guarantee the removal of excess HF. The organics were extracted with DCM (3x 30 mL), the combined organic layers were dried over Na_2SO_4, filtered and the solvent was carefully removed under reduced pressure. An internal standard (ethyl fluoroacetate) was added to the crude residue and the NMR yield was analysed by ^{19}F NMR spectroscopy against the internal standard. The NMR sample was recombined with the crude residue and purification by column chromatography or preparative thin layer chromatography yielded the desired product.

1-(4,4-Difluoropent-1-yn-1-yl)-4-nitrobenzene (1)

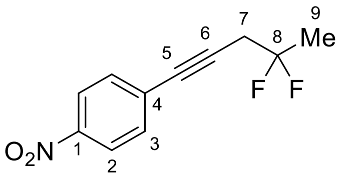

Compound **1** was prepared according to General Procedure **E** with an amine:HF ratio of 1:7.0 using 1-(3-methylbut-3-en-1-yn-1-yl)-4-nitrobenzene (**S1**) (37.4 mg, 0.2 mmol, 1.0 eq.). The crude product was purified by column chromatography (*n*-pentane:Et_2O 20:1) to yield the title compound as a yellow oil (32.2 mg, 0.17 mmol, 83%).

R*f* = 0.45 (*n*-pentane:Et_2O 20:1).

^{1}H NMR (400 MHz, $CDCl_3$) δ 8.25 – 8.13 (m, 2H, H-C2), 7.63 – 7.52 (m, 2H, H-C3), 3.05 (t, $^3J_{HF}$ = 13.3 Hz, 2H, H-C7), 1.79 (t, $^3J_{HF}$ = 18.1 Hz, 3H, H-C9).

^{13}C NMR (126 MHz, $CDCl_3$) δ 147.3 (C4), 132.7 (C3), 129.8 (C1), 123.7 (C2), 121.8 (t, $^1J_{CF}$ = 241.1 Hz, C8), 87.5 (t, $^3J_{CF}$ = 8.6 Hz, C6), 82.0 (t, $^4J_{CF}$ = 1.2 Hz, C5), 30.4 (t, $^2J_{CF}$ = 32.1 Hz, C7), 22.9 (t, $^2J_{CF}$ = 26.6 Hz, C9).

^{19}**F NMR** (470 MHz, $CDCl_3$) δ -89.40 (qt, $^3J_{HF}$ = 18.1, 13.3 Hz, 2F, F-C8).

^{19}**F{^{1}H} NMR** (470 MHz, $CDCl_3$) δ -89.40 (s, 2F, F-C8).

GC-EI-MS: (*m/z*) requires: [($C_{11}H_9NO_2F_2$)] = 225.0596, (*m/z*) found: [($C_{11}H_9NO_2F_2$)] = 225.0597.

FT-IR ($\tilde{\nu}$ = cm^{-1}): 1594 (m), 1517 (s), 1492 (w), 1393 (m), 1340 (s), 1309 (w), 1286 (m), 1260 (w), 1234 (m), 1175 (m), 1144 (m), 1116 (m), 1108 (m), 1014 (w), 963 (m), 936 (m), 877 (m), 852 (s), 796 (w), 783 (w), 749 (m), 687 (m), 634 (w), 613 (w).

4-(4,4-Difluoropent-1-yn-1-yl)benzonitrile (2)

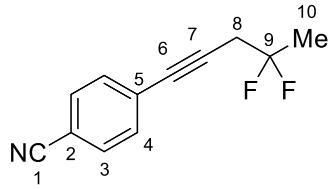

Compound **2** was prepared according to the General Procedure **E** with an amine:HF ratio of 1:7.0 using 4-(3-methylbut-3-en-1-yn-1-yl)benzonitrile (**S2**) (33.4 mg, 0.2 mmol, 1.0 eq.). The crude product was purified by column chromatography (*n*-pentane:Et_2O 10:1) to yield the title compound as a colorless solid (32.7 mg, 0.16 mmol, 80%).

$\mathbf{R}_f$ = 0.40 (*n*-pentane:Et_2O 10:1).

^{1}**H NMR** (500 MHz, $CDCl_3$) δ 7.62 – 7.57 (m, 2H, H-C3), 7.53 – 7.47 (m, 2H, H-C4), 3.03 (t, $^3J_{HF}$ = 13.3 Hz, 2H, H-C8), 1.78 (t, $^3J_{HF}$ = 18.2 Hz, 3H, H-C10).

^{13}**C NMR** (126 MHz, $CDCl_3$) δ 132.4 (C4), 132.1 (C3), 127.8 (C5), 121.7 (t, $^1J_{CF}$ = 241.9 Hz, C9), 118.5 (C1), 111.9 (C2), 86.6 (t, $^3J_{CF}$ = 8.8 Hz, C7), 82.2 (t, $^4J_{CF}$ = 1.2 Hz, C6), 30.4 (t, $^2J_{CF}$ = 31.5 Hz, C8), 22.9 (t, $^2J_{CF}$ = 26.5 Hz, C10).

^{19}**F NMR** (470 MHz, $CDCl_3$) δ -89.45 (qt, $^3J_{HF}$ = 18.2, 13.3 Hz, 2F, F-C9).

^{19}**F{^{1}H} NMR** (470 MHz, $CDCl_3$) δ -89.45 (s, 2F, F-C9).

GC-EI-MS: (*m/z*) requires: [($C_{12}H_9NF_2$)] = 205.0698, (*m/z*) found: [($C_{12}H_9NF_2$)] = 205.0698.

FT-IR ($\tilde{\nu}$ = cm^{-1}) 2223 (s), 1606 (m), 1518 (w), 1502 (m), 1446 (w), 1418 (m), 1390 (s), 1356 (w), 1261 (s), 1227 (s), 1194 (w), 1180 (m), 1164 (m), 1099 (s), 946 (s), 930 (w), 870 (s), 837 (s), 792 (s), 657 (w).

Melting Point: 72-74 ˚C.

1-(4,4-Difluoropent-1-yn-1-yl)-3,5-*bis*(trifluoromethyl)benzene (3)

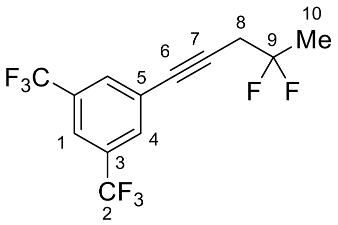

Compound **3** was prepared according to the General Procedure **E** with an amine:HF ratio of 1:7.0 using 1-(3-methylbut-3-en-1-yn-1-yl)-3,5-*bis*(trifluoromethyl)benzene (**S3**) (55.6 mg, 0.2 mmol, 1.0 eq.). The crude product was purified by column chromatography (*n*-pentane) to yield the title compound as a colorless oil (46.7 mg, 0.15 mmol, 74%).

R*f* = 0.33 (*n*-pentane).

^{1}H NMR (500 MHz, $CDCl_3$) δ 7.87 – 7.84 (m, 2H, H-C4), 7.82 – 7.79 (m, 1H, H-C1), 3.04 (t, $^3J_{HF}$ = 13.3 Hz, 2H, H-C8), 1.79 (t, $^3J_{HF}$ = 18.1 Hz, 3H, H-C10).

^{13}C NMR (126 MHz, $CDCl_3$) δ 132.1 (q, $^2J_{CF}$ = 33.7 Hz, C3), 131.9 (m, C4), 125.3 (C5), 123.1 (q, $^1J_{CF}$ = 273.1 Hz, C2), 122.0 (m, C1), 121.7 (t, $^1J_{CF}$ = 241.2 Hz, C9), 85.8 (t, $^3J_{CF}$ = 8.6 Hz, C7), 80.9 (C6), 30.3 (t, $^2J_{CF}$ = 32.1 Hz, C8), 22.9 (t, $^2J_{CF}$ = 26.7 Hz, C10).

^{19}F NMR (470 MHz, $CDCl_3$) δ -63.25 (s, 6F, F-C2), -89.48 (qt, $^3J_{HF}$ = 18.2, 13.3 Hz, 2F, F-C9).

^{19}F{^{1}H} NMR (470 MHz, $CDCl_3$) δ -63.25 (s, 6F, F-C2), -89.48 (s, 2F, F-C9).

GC-EI-MS: (*m/z*) requires: [($C_{13}H_8F_8$)] = 316.0493, (*m/z*) found: [($C_{13}H_8F_8$)] = 316.0488.

FT-IR ($\tilde{\nu}$ = cm^{-1}) 1464 (w), 1385 (m), 1350 (w), 1333 (w), 1276 (s), 1234 (m), 1174 (m), 1129 (s), 1106 (s), 1027 (m), 964 (m), 940 (m), 897 (m), 880 (m), 848 (m), 796 (w), 783 (w), 720 (m), 700 (m), 683 (s), 622 (w).

1-Chloro-4-(4,4-difluoropent-1-yn-1-yl)benzene (4)

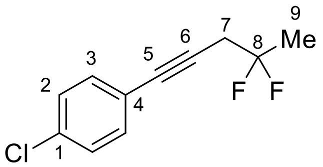

Compound **4** was prepared according to the General Procedure **E** with an amine:HF ratio of 1:5.0 using 1-chloro-4-(3-methylbut-3-en-1-yn-1-yl)benzene (**S4**) (35.2 mg, 0.2 mmol, 1.0 eq.). The crude product was purified by column chromatography (*n*-pentane) to yield the title compound as a colorless oil (20.6 mg, 0.10 mmol, 48%).

R*f* = 0.36 (*n*-pentane).

^{1}H NMR (500 MHz, $CDCl_3$) δ 7.38 – 7.34 (m, 2H, H-C3), 7.30 – 7.26 (m, 2H, H-C2), 3.00 (t, $^{3}J_{HF}$ = 13.3 Hz, 2H, H-C7), 1.78 (t, $^{3}J_{HF}$ = 18.1 Hz, 3H, H-C9).

^{13}C NMR (126 MHz, $CDCl_3$) δ 134.5 (C1), 133.1 (C3), 128.8 (C2), 122.1 (t, $^{1}J_{CF}$ = 240.8 Hz, C8), 121.5 (C4), 82.9 (t, $^{3}J_{CF}$ = 8.9 Hz, C6), 82.6 (t, $^{4}J_{CF}$ = 1.2 Hz, C5), 30.3 (t, $^{2}J_{CF}$ = 32.2 Hz, C7), 22.8 (t, $^{2}J_{CF}$ = 26.7 Hz, C9).

^{19}F NMR (470 MHz, $CDCl_3$) δ -89.53 (qt, $^{3}J_{HF}$ = 18.2, 13.3 Hz, F-C8).

^{19}F{^{1}H} NMR (470 MHz, $CDCl_3$) δ -89.53 (s, 2F, F-C8).

GC-EI-MS (*m/z*) requires: [($C_{11}H_9ClF_2$)] = 214.03554, (*m/z*) found: [($C_{11}H_9ClF_2$)] = 214.03530.

FT-IR ($\tilde{\nu}$ = cm^{-1}) 3008 (w), 1594 (w), 1489 (s), 1446 (w), 1421 (w), 1392 (s), 1339 (w), 1280 (w), 1261 (m), 1235 (s), 1175 (m), 1144 (s), 1116 (s), 1091 (s), 1056 (w), 1016 (s), 963 (s), 936 (s), 878 (s), 827 (s), 796 (m), 783 (m) 759 (s), 707 (w), 641 (m), 618 (w).

1-Chloro-3-(4,4-difluoropent-1-yn-1-yl)benzene (5)

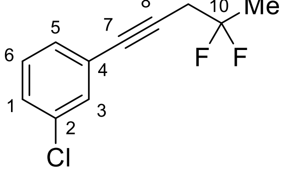

Compound **5** was prepared according to the General Procedure **E** with an amine:HF ratio of 1:5.0 using 1-chloro-3-(3-methylbut-3-en-1-yn-1-yl)benzene (**S5**) (35.3 mg, 0.2 mmol, 1.0 eq.). The crude product was purified by column chromatography (*n*-pentane) to yield the title compound as a colorless oil (26.6 mg, 0.12 mmol, 62%).

R*f* = 0.35 (*n*-pentane).

^{1}**H NMR** (600 MHz, $CDCl_3$) δ 7.43 – 7.41 (m, 1H, H-C3), 7.32 – 7.28 (m, 2H, H-C1, H-C5), 7.25 – 7.21 (m, 1H, H-C6), 3.00 (t, $^{3}J_{HF}$ = 13.2 Hz, 2H, H-C9), 1.78 (t, $^{3}J_{HF}$ = 18.2 Hz, 3H, H-C11).

^{13}**C NMR** (151 MHz, $CDCl_3$) δ 134.3 (C2), 131.8 (C3), 130.0 (C6), 129.7 (C5), 128.8 (C1), 124.7 (C4), 122.1 (t, $^{1}J_{CF}$ = 201.6 Hz, C10), 83.2 (t, $^{3}J_{CF}$ = 8.9 Hz, C8), 82.3 (t, $^{4}J_{CF}$ = 1.3 Hz, C7), 30.3 (t, $^{2}J_{CF}$ = 32.2 Hz, C9), 22.8 (t, $^{2}J_{CF}$ = 26.7 Hz, C11).

^{19}**F NMR** (470 MHz, $CDCl_3$) δ -89.51 (qt, $^{3}J_{HF}$ = 18.2, 13.3 Hz, 2F, F-C10).

^{19}**F{^{1}H} NMR** (470 MHz, $CDCl_3$) δ -89.51 (s, 2F, F-C10).

GC-EI-MS: (*m/z*) requires: [($C_{11}H_9ClF_2$)] = 214.0355, (*m/z*) found: [($C_{11}H_9ClF_2$)] = 214.0355.

FT-IR ($\tilde{\nu}$ = cm^{-1}) 1594 (w), 1563 (w), 1475 (m), 1408 (w), 1392 (m), 1336 (w), 1279 (w), 1233 (m), 1175 (m), 1144 (m), 1118 (s), 1096 (m), 1080 (m), 1010 (w), 963 (m), 937 (m), 878 (s), 781 (s), 680 (s), 617 (w).

1-Bromo-4-(4,4-difluoropent-1-yn-1-yl)benzene (6)

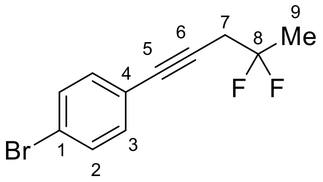

Compound **6** was prepared according to the General Procedure **E** with an amine:HF ratio of 1:5.0 using 1-bromo-4-(3-methylbut-3-en-1-yn-1-yl)benzene (**S6**) (44.2 mg, 0.2 mmol, 1.0 eq.). The crude product was purified by column chromatography (*n*-pentane) to yield the title compound as a colorless oil (32.7 mg, 0.13 mmol, 63%).

R$_f$ = 0.34 (*n*-pentane).

^{1}**H NMR** (500 MHz, $CDCl_3$) δ 7.47 – 7.42 (m, 2H, H-C2), 7.32 – 7.27 (m, 2H, H-C3), 2.99 (t, $^{3}J_{HF}$ = 13.3 Hz, 2H, H-C7), 1.77 (t, $^{3}J_{HF}$ = 18.2 Hz, 3H, H-C9).

^{13}**C NMR** (126 MHz, $CDCl_3$) δ 133.3 (C3), 131.7 (C2), 122.7 (C1), 122.1 (t, $^{1}J_{CF}$ = 201.6 Hz, C8), 121.9 (C4), 83.1 (t, $^{3}J_{CF}$ = 8.9 Hz, C6), 82.6 (t, $^{4}J_{CF}$ = 1.3 Hz, C5), 30.3 (t, $^{2}J_{CF}$ = 32.2 Hz, C7), 22.8 (t, $^{2}J_{CF}$ = 26.7 Hz, C9).

^{19}**F NMR** (470 MHz, $CDCl_3$) δ -89.54 (qt, $^{3}J_{HF}$ = 18.2, 13.3 Hz, 2F, F-C8).

^{19}F{^{1}H} NMR (470 MHz, $CDCl_3$) δ -89.54 (s, 2F, F-C8).

GC-EI-MS: (*m/z*) requires: [($C_{11}H_9{}^{79}BrF_2$)] = 257.9850, (*m/z*) found: [($C_{11}H_9{}^{79}BrF_2$)] = 257.9847.

FT-IR ($\tilde{\nu}$ = cm^{-1}) 2921 (w), 1487 (m), 1392 (m), 1336 (w), 1279 (w), 1258 (m), 1233 (s), 1177 (w), 1144 (s), 1116 (s), 1096 (w), 1070 (s), 1012 (s), 963 (m), 936 (s), 878 (s), 822 (s), 796 (w), 783 (m), 746 (m), 705 (w), 628 (w), 606 (w).

Methyl 4-(4,4-difluoropent-1-yn-1-yl)benzoate (7)

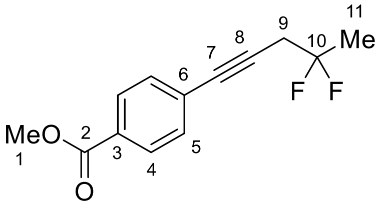

Compound **7** was prepared according to the General Procedure **E** with an amine:HF ratio of 1:7.0 using methyl 4-(3-methylbut-3-en-1-yn-1-yl)benzoate (**S7**) (40.1 mg, 0.2 mmol, 1.0 eq.). The crude product was purified by column chromatography (*n*-pentane:Et_2O 20:1) to yield the title compound as a colorless oil (33.7 mg, 0.14 mmol, 71%).

R*f* = 0.34 (*n*-pentane:Et_2O 20:1).

^{1}H NMR (600 MHz, $CDCl_3$) δ 8.01 – 7.91 (m, 2H, H-C4), 7.52 – 7.43 (m, 2H, H-C5), 3.91 (s, 3H, H-C1), 3.03 (t, $^{3}J_{HF}$ = 13.2 Hz, 2H, H-C9), 1.78 (t, $^{3}J_{HF}$ = 18.2 Hz, 3H, H-C11).

^{13}C NMR (151 MHz, $CDCl_3$) δ 166.6 (C2), 131.8 (C5), 129.8 (C3), 129.6 (C4), 127.6 (C6), 122.0 (t, $^{1}J_{CF}$ = 201.6 Hz, C10), 85.0 (t, $^{3}J_{CF}$ = 8.8 Hz, C8), 83.0 (t, $^{4}J_{CF}$ = 1.2 Hz, C7), 52.4 (C1), 30.4 (t, $^{2}J_{CF}$ = 32.2 Hz, C9), 22.8 (t, $^{2}J_{CF}$ = 26.7 Hz, C11).

^{19}F NMR (470 MHz, $CDCl_3$) δ -89.47 (qt, $^{3}J_{HF}$ = 18.2, 13.3 Hz, 2F, F-C10).

^{19}F{^{1}H} NMR (470 MHz, $CDCl_3$) δ -89.47 (s, 2F, F-C10).

GC-EI-MS: (*m/z*) requires: [($C_{13}H_{12}O_2F_2$)] = 238.0800, (*m/z*) found: [($C_{13}H_{12}O_2F_2$)] = 238.0801.

FT-IR ($\tilde{\nu}$ = cm^{-1}) 2956 (w), 1720 (m), 1607 (m), 1436 (m), 1405 (w), 1393 (m), 1309 (m), 1274 (s), 1234 (m), 1192 (w), 1177 (m), 1144 (m), 1115 (s), 1108 (s), 1066 (w), 1020 (m), 963 (m),

937 (m), 910 (m), 878 (m), 858 (m), 829 (w), 796 (w), 783 (w), 768 (s), 758 (m), 732 (m), 696 (m), 669 (w), 649 (w), 631 (w), 610 (w).

1-(4,4-Difluoropent-1-yn-1-yl)-4-(methylsulfonyl)benzene (8)

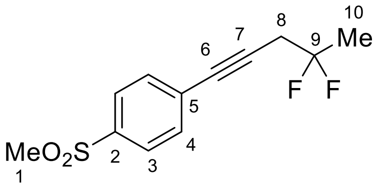

Compound **8** was prepared according to the General Procedure **E** with an amine:HF ratio of 1:5.0 using 1-(3-methylbut-3-en-1-yn-1-yl)-4-(methylsulfonyl)benzene (**S8**) (44.1 mg, 0.2 mmol, 1.0 eq.). The crude product was purified by column chromatography (*n*-pentane:Et_2O 1:1) to yield the title compound as a colorless oil (32.6 mg, 0.13 mmol, 63%).

R_f = 0.30 (*n*-pentane:Et_2O 1:1).

^{1}H NMR (500 MHz, $CDCl_3$) δ 7.90 – 7.85 (m, 2H, H-C4), 7.62 – 7.58 (m, 2H, H-C3), 3.04 (s, 3H, H-C1), 3.03 (t, $^3J_{HF}$ = 13.2 Hz, 2H, H-C8), 1.78 (t, $^3J_{HF}$ = 18.2 Hz, 3H, H-C10).

^{13}C NMR (126 MHz, $CDCl_3$) δ 140.0 (C2), 132.6 (C4), 128.8 (C5), 127.5 (C3), 121.8 (t, $^1J_{CF}$ = 241.9 Hz, C9), 86.3 (t, $^3J_{CF}$ = 8.7 Hz, C7), 82.1 (t, $^4J_{CF}$ = 1.2 Hz, C6), 44.6 (C1), 30.3 (t, $^2J_{CF}$ = 32.1 Hz, C8), 22.9 (t, $^2J_{CF}$ = 26.7 Hz, C10).

^{19}F NMR (470 MHz, $CDCl_3$) δ -89.42 (qt, $^3J_{HF}$ = 18.2, 13.3 Hz, 2F, F-C9).

^{19}F{^{1}H} NMR (470 MHz, $CDCl_3$) δ -89.42 (s, 2F, F-C9).

GC-EI-MS: (*m/z*) requires: [($C_{12}H_{12}O_2SF_2$)] = 258.0521, (*m/z*) found: [($C_{12}H_{12}O_2SF_2$)] = 258.0520.

FT-IR ($\tilde{\nu}$ = cm^{-1}) 3668 (w), 2972 (s), 2901 (m), 1393 (m), 1310 (m), 1280 (w), 1233 (m), 1149 (s), 1066 (s), 1058 (s), 1027 (m), 957 (w), 936 (w), 878 (w), 839 (w), 778 (m), 746 (w), 618 (w).

1-(4-(4,4-Difluoropent-1-yn-1-yl)phenyl)-2,2,2-trifluoroethan-1-one (9)

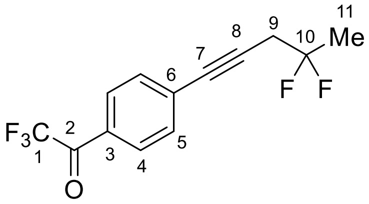

Compound **9** was prepared according to the General Procedure **E** with an amine:HF ratio of 1:7.0 using 2,2,2-trifluoro-1-(4-(3-methylbut-3-en-1-yn-1-yl)phenyl)ethan-1-one (**S9**) (47.6 mg, 0.2 mmol, 1.0 eq.). The crude product was purified by column chromatography (*n*-pentane:Et_2O 1.5:1) to yield the title compound as a yellow oil (37.0 mg, 0.13 mmol, 67%).

R*f* = 0.45 (*n*-pentane:Et_2O 1.5:1).

^{1}H NMR (400 MHz, $CDCl_3$) δ 8.04 – 7.98 (m, 2H, H-C4), 7.60 – 7.55 (m, 2H, H-C5), 3.06 (t, $^3J_{HF}$ = 13.3 Hz, 2H, H-C9), 1.79 (t, $^3J_{HF}$ = 18.1 Hz, 3H, H-C11).

^{13}C NMR (126 MHz, $CDCl_3$) δ 179.9 (app. q, $^2J_{CF}$ = 35.3 Hz, C2), 132.4 (C4), 130.49(C5), 130.1 (q, $^3J_{CF}$ = 2.2 Hz, C3), 129.2 (C6),121.8 (t, $^1J_{CF}$ = 241.9 Hz, C10), 116.7 (q, $^1J_{CF}$ = 292.3Hz, C1), 87.6 (t, $^3J_{CF}$ = 8.7 Hz, C8), 82.6 (app. t, $^4J_{CF}$ = 1.2 Hz, C7), 30.5 (t, $^2J_{CF}$ = 32.2 Hz, C9), 22.9 (t, $^2J_{CF}$ = 26.7 Hz, C11).

^{19}F NMR (470 MHz, $CDCl_3$) δ -71.52 (s, 3F, F-C1), -89.40 (qt, $^3J_{HF}$ = 18.2, 13.3 Hz, 2F, F-C10).

^{19}F{^{1}H} NMR (470 MHz, $CDCl_3$) δ -71.52 (s, 3F, F-C1), -89.40 (s, 2F, F-C10).

GC-EI-MS: (*m/z*) requires: [($C_{13}H_9OF_5$)] = 276.0568, (*m/z*) found: [($C_{13}H_9OF_5$)] = 276.0558.

FT-IR ($\tilde{\nu}$ = cm^{-1}) 2980 (w), 1718 (m), 1603 (m), 1555 (w), 1412 (w), 1393 (m), 1336 (w), 1281 (w), 1261 (w), 1233 (m), 1205 (m), 1195 (m), 1174 (s), 1139 (s), 1116 (s), 1066 (w), 1058 (w), 1027 (w), 963 (m), 937 (s), 878 (m), 852 (m), 796 (w), 783 (w), 766 (m), 722 (m), 702 (w), 631 (w), 616 (w).

1-(4,4-Difluoropent-1-yn-1-yl)-3-methylbenzene (10)

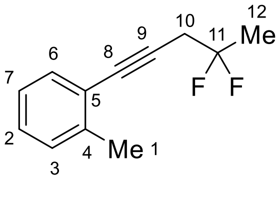

Compound **10** was prepared according to the General Procedure **E** with an amine:HF ratio of 1:4.5 using 1-methyl-2-(3-methylbut-3-en-1-yn-1-yl)benzene (**S10**) (31.2 mg, 0.2 mmol, 1.0 eq.). After workup, the

crude mixture was analysed by ^{19}F NMR (42% *gem*-difluoride and 23% *vic*-difluoride). Purification by column chromatography (*n*-pentane) yielded the title compound **10** as a colorless volatile liquid (6.4 mg, 0.03 mmol, 16%).

R*f* = 0.30 (*n*-pentane).

^{1}H NMR (500 MHz, $CDCl_3$) δ 7.41 – 7.37 (m, 1H, H-C6), 7.24 – 7.17 (m, 2H, H-C2, H-C3), 7.14 – 7.10 (m, 1H, H-C7), 3.05 (t, $^{3}J_{HF}$ = 13.3 Hz, 2H, H-C10), 2.43 (s, 3H, H-C1), 1.80 (t, $^{3}J_{HF}$ = 18.2 Hz, 3H, H-C12).

^{13}C NMR (126 MHz, $CDCl_3$) δ 140.5 (C4), 132.1 (C6), 129.6 (C3), 128.4 (C2), 125.7 (C7), 122.8 (C5), 122.3 (t, $^{1}J_{CF}$ = 201.6 Hz, C11), 85.7 (t, $^{3}J_{CF}$ = 9.1 Hz, C9), 82.6 (C8), 30.5 (t, $^{2}J_{CF}$ = 32.2 Hz, C10), 22.8 (t, $^{2}J_{CF}$ = 26.7 Hz, C12), 20.8 (C1).

^{19}F NMR (470 MHz, $CDCl_3$) δ -89.66 (qt, $^{3}J_{HF}$ = 18.2, 13.3 Hz, 2F, F-C11).

^{19}F{^{1}H} NMR (470 MHz, $CDCl_3$) δ -89.66 (s, 2F, F-C11).

GC-EI-MS: (*m/z*) requires: [($C_{12}H_{12}F_2$)] = 194.0902, (*m/z*) found: [($C_{12}H_{12}F_2$)] = 194.0902.

FT-IR ($\tilde{\nu}$ = cm^{-1}) 2921 (w), 1485 (w), 1456 (w), 1392 (m), 1337 (w), 1280 (w), 1254 (w), 1233 (m), 1175 (w), 1142 (s), 1116 (s), 1045 (w), 963 (m), 936 (m), 878 (m), 795 (w), 782 (w), 756 (s), 716 (m), 620 (w).

1-(3,4-difluoro-3-methylbut-1-yn-1-yl)-3-methylbenzene (10')

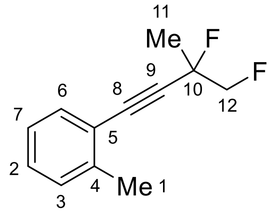

Compound **10'** was prepared according to the General Procedure **E** in the absence of a catalyst with an amine:HF ratio of 1:4.5 using 1-methyl-2-(3-methylbut-3-en-1-yn-1-yl)benzene (**S10**) (62.5 mg, 0.4 mmol, 1.0 eq.). After workup, the crude mixture was analysed by ^{19}F NMR (<5% *gem*-difluoride and 53% *vic*-difluoride). Purification by column chromatography (*n*-pentane) yielded the title compound **10'** as a colorless volatile liquid (32.8 mg, 0.17 mmol, 42%).

R*f* = 0.25 (*n*-pentane).

^{1}H NMR (500 MHz, $CDCl_3$) δ 7.30 – 7.25 (m, 1H, H-C6), 7.30 – 7.25 (m, 1H, H-C2), 7.24 – 7.20 (m, 1H, H-C3), 7.19 – 7.13 (m, 1H, H-C7), 4.72 – 4.39 (m, 2H, H-C12), 2.44 (s, 3H, H-C1), 1.79 (dd, $^3J_{HF}$ = 20.1 Hz, $^4J_{HF}$ = 2.0 Hz, 3H, H-C11).

^{13}C NMR (126 MHz, $CDCl_3$) δ 140.8 (d, $^5J_{CF}$ = 2.3 Hz, C4), 132.3 (d, $^5J_{CF}$ = 2.6 Hz, C6), 129.7 (C3), 129.4 (d, $^7J_{CF}$ = 0.7 Hz, C2), 125.8 (C7), 121.2 (d, $^4J_{CF}$ = 3.3 Hz, C5), 88.4 (dd, $^2J_{CF}$ = 28.6 Hz, $^3J_{CF}$ = 7.7 Hz, C9), 88.3 (dd, $^1J_{CF}$ = 172.8 Hz, $^2J_{CF}$ = 21.8 Hz, C10), 87.2 (dd, $^3J_{CF}$ = 8.8 Hz, $^4J_{CF}$ = 1.6 Hz, C8), 86.0 (dd, $^1J_{CF}$ = 184.6 Hz, $^2J_{CF}$ = 27.4 Hz, C12), 23.2 (dd, $^2J_{CF}$ = 26.6 Hz, $^3J_{CF}$ = 3.5 Hz, C9), 20.6 (C1).

^{19}F NMR (470 MHz, $CDCl_3$) δ -143.0 (m, 1F, F-C10), -224.0 (m, 1F, F-C12).

^{19}F{^{1}H} NMR (470 MHz, $CDCl_3$) δ -143.0 (d, $^3J_{HF}$ = 15.8, 1F, F-C10), -224.0 (d, $^3J_{HF}$ = 15.8, 1F, F-C12).

GC-EI-MS: (*m/z*) requires: [($C_{12}H_{12}F_2$)] = 194.0902, (*m/z*) found: [($C_{12}H_{12}F_2$)] = 194.0902.

FT-IR ($\tilde{\nu}$ = cm^{-1}) 2993 (w), 2227 (w), 1487 (m), 1456 (w), 1310 (m), 1268 (w), 1202 (m), 1162 (w), 1135 (m), 1106 (w), 1040 (s), 943 (w), 898 (s), 831 (m), 824 (m), 756 (s), 716 (s), 616 (m).

3-(4,4-Difluoropent-1-yn-1-yl)pyridine (11)

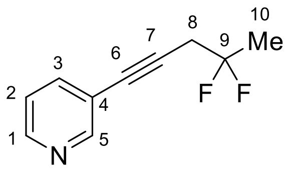

Compound **11** was prepared according to the General Procedure **E** with an amine:HF ratio of 1:6.0 using 3-(3-methylbut-3-en-1-yn-1-yl)pyridine (**S11**) (28.6 mg, 0.2 mmol, 1.0 eq.). The crude product was purified by preparative thin layer chromatography (*n*-pentane:Et_2O 1.5:1) to yield the title compound as a yellow oil (17.4 mg, 0.10 mmol, 48%).

R*f* = 0.30 (*n*-pentane:Et_2O 2:1).

^{1}H NMR (400 MHz, $CDCl_3$) δ 8.66 (d, $^4J_{HH}$ = 1.3 Hz, 1H, H-C5), 8.53 (dd, $^3J_{HH}$ = 4.9 Hz, $^4J_{HH}$ = 1.7 Hz, 1H, H-C1), 7.71 (dt, $^3J_{HH}$ = 7.9 Hz, $^4J_{HH}$ = 1.9 Hz, 1H, H-C3), 7.24 (ddd, $^3J_{HH}$ = 7.9, 4.9 Hz, $^4J_{HH}$ = 0.9 Hz, 1H, H-C2), 3.03 (t, $^3J_{HF}$ = 13.2 Hz, 2H, H-C8), 1.78 (t, $^3J_{HF}$ = 18.1 Hz, 3H, H-C10).

^{13}**C NMR** (126 MHz, $CDCl_3$) δ 152.6 (C5), 148.9 (C1), 138.8 (C3), 125.7 (C4), 123.1 (C2), 122.0 (t, $^1J_{CF}$ = 241.0 Hz, C9), 85.4 (t, $^3J_{CF}$ = 8.8 Hz, C7), 80.5 (C6), 30.4 (t, $^2J_{CF}$ = 32.2 Hz, C8), 22.9 (t, $^2J_{CF}$ = 26.7 Hz, C10).

^{19}**F NMR** (470 MHz, $CDCl_3$) δ -89.50 (qt, $^3J_{HF}$ = 18.2, 13.3 Hz, 2F, F-C9).

^{19}**F{^{1}H} NMR** (470 MHz, $CDCl_3$) δ -89.50 (s, 2F, F-C9).

GC-EI-MS: (*m/z*) requires: [($C_{10}H_9NF_2$)] = 181.0698, (*m/z*) found: [($C_{10}H_9NF_2$)] = 181.0693.

FT-IR ($\tilde{\nu}$ = cm^{-1}): 2924 (w), 1564 (w), 1477 (m), 1409 (m), 1392 (m), 1280 (w), 1266 (w), 1233 (s), 1177 (m), 1144 (s), 1118 (s), 1025 (m), 963 (m), 937 (s), 878 (m), 805 (m), 783 (m), 756 (m), 735 (w), 705 (s), 627 (m), 617 (w).

6-(4,4-Difluoropent-1-yn-1-yl)quinoline (12)

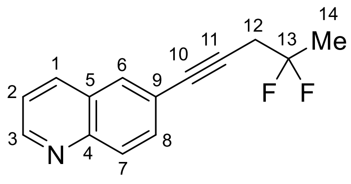

Compound **12** was prepared according to the General Procedure **E** with an amine:HF ratio of 1:6.0 using 6-(3-methylbut-3-en-1-yn-1-yl)quinoline (**S12**) (38.7 mg, 0.2 mmol, 1.0 eq.). The crude product was purified by column chromatography (*n*-pentane:Et_2O 1:1) to yield the title compound as a colorless oil (25.9 mg, 0.11 mmol, 56%).

R*f* = 0.32 (*n*-pentane:Et_2O 1:1).

^{1}**H NMR** (600 MHz, $CDCl_3$) δ 8.90 (dd, $^3J_{HH}$ = 4.2 Hz, $^4J_{HH}$ =1.7 Hz, 1H, H-C3), 8.11-8.07 (m, 1H, H-C1), 8.06 – 8.00 (m, 1H, H-C7), 7.91 (d, $^4J_{HH}$ = 1.8 Hz, 1H, H-C6), 7.70 (dd, $^3J_{HH}$ = 8.7 Hz, $^4J_{HH}$ = 1.8 Hz, 1H, C8), 7.40 (dd, $^3J_{HH}$ = 8.3, 4.2 Hz, 1H, C2), 3.06 (t, $^3J_{HF}$ = 13.3 Hz, 2H, H-C12), 1.81 (t, $^3J_{HF}$ = 18.2 Hz, 3H, H-C14).

^{13}**C NMR** (151 MHz, $CDCl_3$) δ 151.1 (C3), 147.8 (C4), 135.8 (C1), 132.3 (C8), 131.5 (C6), 129.7 (C7), 128.1 (C5), 122.1 (t, $^1J_{CF}$ = 241.6 Hz, C13), 121.9 (C2), 121.2 (C9), 83.2 (t, $^4J_{CF}$ = 1.3 Hz, C10), 83.2 (t, $^3J_{CF}$ = 8.8 Hz, C11), 30.4 (t, $^2J_{CF}$ = 32.1 Hz, C12), 22.9 (t, $^2J_{CF}$ = 26.7 Hz, C14).

^{19}**F NMR** (564 MHz, $CDCl_3$) δ -89.46 (qt, $^3J_{HF}$ = 18.2 Hz, $^3J_{HF}$ =13.3 Hz, 2F, F-C13).

^{19}F{^{1}H} NMR (564 MHz, $CDCl_3$) δ-89.46 (s, 2F, F-C13).

GC-EI-MS: (*m/z*) requires: [($C_{14}H_{11}NF_2$)] = 231.0854, (*m/z*) found: [($C_{14}H_{11}NF_2$)] = 231.0854.

FT-IR ($\tilde{\nu}$ = cm^{-1}): 2972 (w), 1591 (w), 1568 (w), 1495 (m), 1393 (m), 1320 (w), 1233 (m), 1175 (w), 1144 (m), 1118 (m), 1076 (w), 964 (m), 937 (m), 880 (m), 838 (m), 796 (m), 772 (s).

(4-(4,4-Difluoropent-1-yn-1-yl)phenyl)(morpholino)methanone (13)

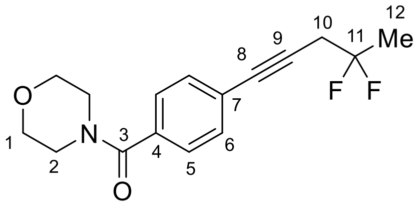

Compound **13** was prepared according to the General Procedure **E** with an amine:HF ratio of 1:6.0 using (4-(3-methylbut-3-en-1-yn-1-yl)phenyl)(morpholino)methanone (**S13**) (51.1 mg, 0.2 mmol, 1.0 eq.). The crude product was purified by column chromatography (*n*-pentane:EtOAc 1:2) to yield the title compound as a colorless oil (30.0 mg, 0.10 mmol, 51%).

R*f* = 0.35 (*n*-pentane:EtOAc 1:2).

^{1}H NMR (599 MHz, $CDCl_3$) δ 7.49 – 7.45 (m, 2H, H-C6), 7.37 – 7.33 (m, 2H, H-C7), 3.87 – 3.42 (m, 8H, H-C1, H-C2), 3.01 (t, $^{3}J_{HF}$ = 13.3 Hz, 2H, H-C10), 1.78 (t, $^{3}J_{HF}$ = 18.3 Hz, 2H, H-C10).

^{13}C NMR (151 MHz, $CDCl_3$) δ 169.9 (C3), 135.1 (C7), 132.0 (C6), 127.3 (C5), 124.7 (C4), 122.1 (t, $^{1}J_{CF}$ = 240.9 Hz, C17), 83.6 (t, $^{3}J_{CF}$ = 8.9 Hz, C9), 82.8 (C8), 67.0 (C1, C2), 30.3 (t, $^{2}J_{CF}$ = 32.2 Hz, C10), 22.8 (t, $^{2}J_{CF}$ = 26.7 Hz, C12).

^{19}F NMR (564 MHz, $CDCl_3$) δ -89.51 (qt, $^{3}J_{HF}$ = 18.3, 13.3 Hz, 2F, F-C11).

^{19}F{^{1}H} NMR (564 MHz, $CDCl_3$) δ -89.51 (s, 2F, F-C11).

ESI-MS: (*m/z*) requires: [($C_{16}H_{17}NO_2F_2Na$)$^{+}$] = 316.1120, (*m/z*) found: [($C_{16}H_{17}NO_2F_2Na$)$^{+}$] = 316.1117.

FT-IR ($\tilde{\nu}$ = cm^{-1}): 2980 (m), 2901 (m), 1633 (w), 1393 (m), 1279 (w), 1258 (w), 1220 (m), 1145 (w), 1113 (m), 1066 (m), 1058 (m), 1027 (m), 964 (w), 936 (w), 880 (w), 839 (w), 772 (s).

1-(4,4-Difluorohex-1-yn-1-yl)-4-nitrobenzene (14)

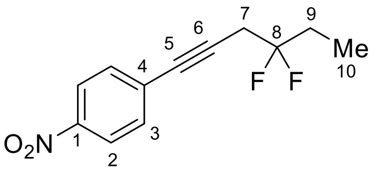

Compound **14** was prepared according to the General Procedure **E** with an amine:HF ratio of 1:7.0 using 1-(3-methylenepent-1-yn-1-yl)-4-nitrobenzene (**S14**) (40.2 mg, 0.2 mmol, 1.0 eq.). The crude product was purified by column chromatography (*n*-pentane:Et_2O 20:1) to yield the title compound as a yellow oil (41.1 mg, 0.17 mmol, 86%).

$\mathbf{R}_f$ = 0.37 (*n*-pentane:Et_2O 20:1).

^{1}H NMR (500 MHz, $CDCl_3$) δ 8.30 – 8.02 (m, 2H, H-C2), 7.71 – 7.36 (m, 2H, H-C3), 3.05 (t, $^3J_{HF}$ = 13.6 Hz, 2H, H-C7), 2.07 (tq, $^3J_{HF}$ = 16.4 Hz, $^3J_{HH}$ = 7.5 Hz, 2H, H-C9), 1.10 (t, $^3J_{HH}$ = 7.5 Hz, 3H, H-C10).

^{13}C NMR (126 MHz, $CDCl_3$) δ 147.3 (C1), 132.7 (C3), 129.9 (C4), 123.7 (C2), 123.0 (t, $^1J_{CF}$ = 243.2 Hz, C8), 87.5 (t, $^3J_{CF}$ = 9.5 Hz, C6), 81.9 (t, $^4J_{CF}$ = 1.2 Hz, C5), 29.1 (t, $^2J_{CF}$ = 25.2 Hz, C7), 28.6 (t, $^2J_{CF}$ = 31.5 Hz, C9), 6.6 (t, $^3J_{CF}$ = 5.5 Hz, C10).

^{19}F NMR (470 MHz, $CDCl_3$) δ -98.42 (tt,$^3J_{HF}$ = 16.5 Hz, $^3J_{HF}$ =13.7 Hz, 2F, F-C8).

^{19}F{^{1}H} NMR (470 MHz, $CDCl_3$) δ -98.42 (s, 2F, F-C8).

GC-EI-MS: (*m/z*) requires: [($C_{12}H_{11}NO_2F_2$)] = 239.0752, (*m/z*) found: [($C_{12}H_{11}NO_2F_2$)] = 239.0752.

FT-IR ($\tilde{\nu}$ = cm^{-1}): 2986 (w), 2946 (w), 1594 (m), 1517 (s), 1492 (w), 1466 (w), 1372 (w), 1340 (s), 1309 (w), 1286 (w), 1174 (w), 1147 (m), 1106 (m), 1046 (w), 1004 (m), 987 (m), 878 (m), 852 (s), 794 (w), 749 (s), 687 (m), 662 (w), 643 (w), 623 (w).

1-(5-Bromo-4,4-difluoropent-1-yn-1-yl)-4-nitrobenzene (15)

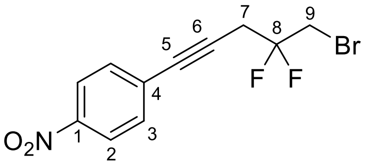

Compound **15** was prepared according to the General Procedure **E** with an amine:HF ratio of 1:7.0 using 1-(3-(bromomethyl)but-3-en-1-yn-1-yl)-4-nitrobenzene (**S15**) (53.2 mg, 0.2 mmol, 1.0 eq.). The crude product was purified by column chromatography (*n*-pentane:Et_2O 14:1) to yield the title compound as a yellow oil (55.6 mg, 0.18 mmol, 91%).

R*f* = 0.45 (*n*-pentane:Et_2O 14:1).

^{1}H NMR (600 MHz, $CDCl_3$) δ 8.21 – 8.16 (m, 2H, H-C2), 7.61 – 7.55 (m, 2H, H-C3), 3.73 (t, $^3J_{HF}$ = 12.6 Hz, 2H, H-C7), 3.31 (t, $^3J_{HF}$ = 13.3 Hz, 2H, H-C9).

^{13}C NMR (151 MHz, $CDCl_3$) δ 147.5 (C1), 132.8 (C3), 129.4 (C4), 123.7 (C2), 119.4 (t, $^1J_{CF}$ = 246.0 Hz, C8), 85.8 (t, $^3J_{CF}$ = 8.4 Hz, C6), 82.6 (C5), 30.2 (t, $^2J_{CF}$ = 32.5 Hz, C7), 27.5 (t, $^2J_{CF}$ = 30.6 Hz, C9).

^{19}F NMR (564 MHz, $CDCl_3$) δ -97.39 (tt,$^3J_{HF}$ = 11.3 Hz, $^3J_{HF}$ =11.1 Hz, 2F, F-C8).

^{19}F{^{1}H} NMR (564 MHz, $CDCl_3$) δ -97.39 (s, 2F, F-C8).

GC-EI-MS: (*m/z*) requires: [($C_{11}H_8NO_2{}^{79}BrF_2$)] = 302.9701, (*m/z*) found: [($C_{11}H_8NO_2{}^{79}BrF_2$)] = 302.9700.

FT-IR ($\tilde{\nu}$ = cm^{-1}): 2972 (w), 1594 (m), 1515 (m), 1492 (w), 1423 (w), 1340 (s), 1309 (w), 1301 (w), 1286 (m), 1246 (w), 1220 (m), 1175 (w), 1157 (w), 1142 (m), 1108 (m), 1080 (m), 1039 (m), 1014 (m), 888 (w), 852 (s), 798 (m), 772 (s), 749 (m), 697 (m), 687 (m), 667 (w), 631 (w).

1-(5-Chloro-4,4-difluoropent-1-yn-1-yl)-4-nitrobenzene (16)

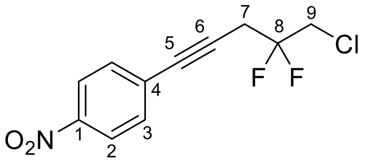

Compound **16** was prepared according to the General Procedure **E** with an amine:HF ratio of 1:7.0 using 1-(3-(chloromethyl)but-3-en-1-yn-1-yl)-4-nitrobenzene (**S16**) (44.3 mg, 0.2 mmol, 1.0 eq.). The crude product was purified by column chromatography (*n*-pentane:Et_2O 15:1) to yield the title compound as a yellow oil (46.6 mg, 0.18 mmol, 90%).

R*f* = 0.44 (*n*-pentane:Et_2O 15:1).

^{1}H NMR (500 MHz, $CDCl_3$) δ 8.22 – 8.15 (m, 2H, H-C2), 7.62 – 7.52 (m, 2H, H-C3), 3.87 (t, $^3J_{HF}$ = 11.8 Hz, 2H, H-C7), 3.26 (t, $^3J_{HF}$ = 13.5 Hz, 2H, H-C9).

^{13}C NMR (126 MHz, $CDCl_3$) δ 147.5 (C1), 132.8 (C3), 129.4 (C4), 123.7 (C2), 119.9 (t, $^1J_{CF}$ = 246.6 Hz, C8), 85.7 (t, $^3J_{CF}$ = 8.4 Hz, C6), 82.5 (t, $^4J_{CF}$ = 1.1 Hz, C5), 43.4 (t, $^2J_{CF}$ = 33.6 Hz, C7), 27.8 (t, $^2J_{CF}$ = 30.2 Hz, C9).

^{19}F NMR (470 MHz, $CDCl_3$) δ -100.20 (tt,$^3J_{HF}$ = 13.6 Hz, $^3J_{HF}$ =11.8 Hz, 2F, F-C8).

^{19}F{^{1}H} NMR (470 MHz, $CDCl_3$) δ-100.20 (s, 2F, F-C8).

GC-EI-MS: (*m/z*) requires: [($C_{11}H_8NO_2ClF_2$)] = 259.0206, (*m/z*) found: [($C_{11}H_8NO_2ClF_2$)] = 259.0206.

FT-IR ($\tilde{\nu}$ = cm^{-1}): 2972 (w), 1594 (m), 1517 (m), 1492 (w), 1429 (w), 1340 (s), 1322 (m), 1309 (m), 1286 (m), 1264 (m), 1220 (m), 1175 (w), 1151 (m), 1099 (m), 1059 (s), 1014 (w), 917 (w), 881 (w), 854 (s), 815 (m), 772 (s), 749 (m), 687(m), 680 (w), 637 (w), 621 (w).

(1*R*,2*S*,5*R*)-2-Isopropyl-5-methylcyclohexyl 4-(4,4-difluoropent-1-yn-1-yl)benzoate (17)

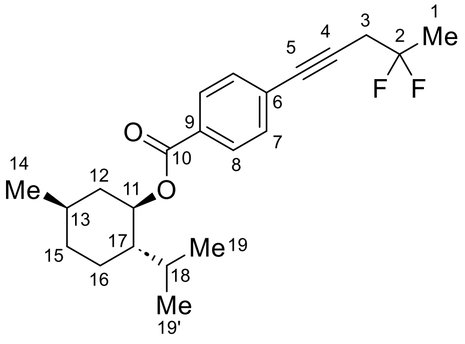

Compound **17** was prepared according to the General Procedure **E** with an amine:HF ratio of 1:6.0 using (1*R*,2*S*,5*R*)-2-isopropyl-5-methylcyclohexyl 4-(3-methylbut-3-en-1-yn-1-yl)benzoate (**S17**) (64.9 mg, 0.2 mmol, 1.0 eq.). The crude product was purified by column chromatography (*n*-pentane:DCM 3:1-2:1) to yield the title compound as a colorless oil (44.7 mg, 0.12 mmol, 62%).

R*f* = 0.41 (*n*-pentane:Et_2O 30:1).

^{1}H NMR (400 MHz, $CDCl_3$) δ 8.03 – 7.93 (m, 2H, H-C8), 7.52 – 7.44 (m, 2H, H-C7), 4.93 (td, $^3J_{HH}$ = 10.9, 4.4 Hz, 1H, H-C11), 3.03 (t, $^3J_{HF}$ = 13.2 Hz, 2H, H-C3), 2.16 – 2.08 (m, 1H, H-C12), 1.99 – 1.88 (m, 1H, H-C13), 1.85 – 1.69 (m, 5H, H-C1, H-C16, H-C15), 1.60 – 1.50 (m, 2H, H-C18, H-C17), 1.18 – 1.04 (m, 2H, H-C12, H-C16), 0.98 – 0.85 (m, 7H, H-C15, H-C19, H-C19'), 0.79 (d, $^3J_{HH}$ = 6.9 Hz, 3H, H-C14).

^{13}C NMR (126 MHz, $CDCl_3$) δ 165.6 (C10), 131.8 (C7), 130.5 (C6), 129.6 (C8), 127.4 (C9), 122.1 (t, $^1J_{CF}$ = 241.0 Hz, C2), 84.8 (t, $^3J_{CF}$ = 8.9 Hz, C4), 83.1 (app. t, $^4J_{CF}$ = 1.3 Hz, C5), 75.3 (C11), 47.4 (C17), 41.1 (C12), 34.5 (C15), 31.6 (C18), 30.4 (t, $^2J_{CF}$ = 32.2 Hz, C3), 26.7 (C13), 23.8 (C16), 22.8 (t, $^2J_{CF}$ = 26.8 Hz, C1), 22.2 (C19), 20.9 (C19'), 16.7 (C14).

^{19}F NMR (470 MHz, $CDCl_3$) δ -89.44 (qt, $^3J_{HF}$ = 18.2, 13.2 Hz, 2F, F-C2).

$^{19}F\{^1H\}$ NMR (470 MHz, $CDCl_3$) δ -89.44 (s, 2F, F-C2).

ESI-MS: (*m/z*) requires: $[(C_{22}H_{28}O_2F_2Na)^+]$ = 385.1950, (*m/z*) found: $[(C_{22}H_{28}O_2F_2Na)^+]$ = 385.1951.

FT-IR ($\tilde{\nu}$ = cm^{-1}): 2959 (w), 1716 (w), 1289 (w), 1273 (m), 1220 (m), 1175 (w), 1145 (w), 1118 (w), 772 (s).

(3*S*,5*S*,8*R*,9*S*,10*S*,13*R*,14*S*,17*R*)-10,13-Dimethyl-17-((*R*)-6-methylheptan-2-yl)hexadecahydro-1H-cyclopenta[a]phenanthren-3-yl 4-(4,4-difluoropent-1-yn-1-yl)benzoate (18)

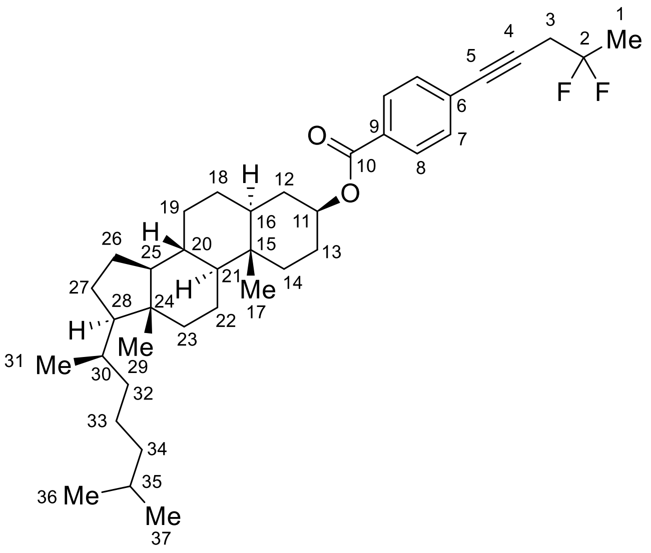

Compound **18** was prepared according to the General Procedure **E** with an amine:HF ratio of 1:7.0 using (3*S*,5*S*,8*R*,9*S*,10*S*,13*R*,14*S*,17*R*)-10,13-dimethyl-17-((*R*)-6-methylheptan-2-yl)hexadecahydro-1H-cyclopenta[a]phenanthren-3-yl 4-(3-methylbut-3-en-1-yn-1-yl)benzoate (**S18**) (55.7 mg, 0.1 mmol, 1.0 eq.). The crude product was purified by column chromatography (*n*-pentane:Et_2O 20:1) to yield the title compound as a white solid (36.5 mg, 0.06 mmol, 61%).

R*f* = 0.40 (*n*-pentane:Et_2O 20:1).

^1H NMR (599 MHz, $CDCl_3$) δ 7.99 – 7.94 (m, 2H, H-C8), 7.49 – 7.44 (m, 2H, H-C7), 4.93 (tt, $^3J_{HH}$ = 11.3, 4.9 Hz, 1H, H-C11), 3.03 (t, $^3J_{HF}$ = 13.2 Hz, 2H, H-C3), 2.00 – 1.91 (m, 2H), 1.86 – 1.75 (m, 5H), 1.74 – 1.59 (m, 3H), 1.59 – 1.55 (m, 1H), 1.54 – 1.46 (m, 3H), 1.43 – 1.18 (m, 10H), 1.17 – 0.95 (m, 9H), 0.91 (d, *J* = 6.6 Hz, 3H), 0.88 – 0.85 (m, 9H), 0.66 (m, 4H).

^{13}C NMR (151 MHz, $CDCl_3$) δ 165.7 (C10), 131.7 (C7), 130.7 (C6), 129.6 (C8), 127.3 (C9), 122.1 (t, $^1J_{CF}$ = 240.9 Hz, C2), 84.8 (t, $^3J_{CF}$ = 8.9 Hz, C4), 83.1 (C5), 74.8 (C11), 56.6 (*C*H),

56.4 (*C*H), 54.4 (*C*H), 44.9 (*C*H), 42.8 (*C*), 40.2 (*C*H_2), 39.7 (*C*H_2), 37.0 (*C*H_2), 36.3 (*C*H_2), 36.0 (*C*H), 35.7 (*C*), 35.7 (*C*H), 34.3 (*C*H_2), 32.2 (*C*H_2), 30.4 (t, $^2J_{CF}$ = 32.2 Hz, C3), 28.8 (*C*H_2), 28.4 (*C*H_2), 28.2 (*C*H), 27.7 (*C*H_2), 24.4 (*C*H_2), 24.0 (*C*H_2), 23.0 (*C*H_3), 22.9 (t, $^2J_{CF}$ = 26.7 Hz, C1), 22.7 (*C*H_3), 21.4 (*C*H_2), 18.8 (*C*H_3), 12.5 (*C*H_3), 12.2 (*C*H_3).

^{19}F NMR (564 MHz, $CDCl_3$) δ -89.46 (qt, $^3J_{HF}$ = 18.1, 13.2 Hz, 2F, F-C2).

^{19}F{^{1}H} NMR (564 MHz, $CDCl_3$) δ -89.46 (s, 2F, F-C2).

ESI-MS: (*m/z*) requires: [$(C_{39}H_{56}O_2F_2Na)^+$] = 617.4141, (*m/z*) found: [$(C_{39}H_{56}O_2F_2Na)^+$] = 617.4140.

FT-IR ($\tilde{\nu}$ = cm^{-1}): 2929 (w), 2868 (w), 1705 (m), 1607 (w), 1468 (w), 1449 (w), 1405 (w), 1392 (w), 1332 (w), 1307 (w), 1277 (m), 1235 (w), 1175 (w), 1147 (w), 1118 (m), 1019 (w), 964 (w), 938 (w), 907 (s), 880 (w), 857 (w), 771 (m), 732 (s), 700 (w), 650 (w).

Melting Point: 168-170 ˚C.

(8*R*,9*S*,13*S*)-13-Methyl-17-oxo-7,8,9,11,12,13,14,15,16,17-decahydro-6H-cyclopenta[a]phenanthren-3-yl 4-(4,4-difluoropent-1-yn-1-yl)benzoate (19)

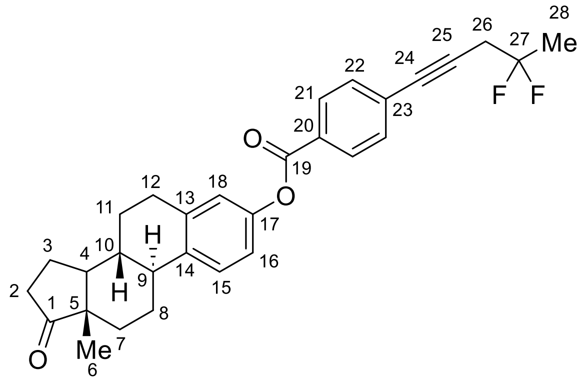

Compound **19** was prepared according to the General Procedure **E** with an amine:HF ratio of 1:6.0 using (8*R*,9*S*,13*S*)-13-methyl-17-oxo-7,8,9,11,12,13,14,15,16,17-decahydro-6H-cyclopenta[a]phenanthren-3-yl 4-(3-methylbut-3-en-1-yn-1-yl)benzoate (**S19**) (43.9 mg, 0.1 mmol, 1.0 eq.). The crude product was purified by column chromatography (*n*-pentane:Et_2O 1.5:1) to yield the title compound as a white solid (28.7 mg, 0.06 mmol, 60%).

R*f* = 0.34 (*n*-pentane:Et_2O 1.5:1).

^{1}H NMR (599 MHz, $CDCl_3$) δ 8.16 – 8.10 (m, 2H, H-C21), 7.58 – 7.52 (m, 2H, H-C22), 7.34 (d, $^3J_{HH}$ = 8.7 Hz, 1H, H-C15), 6.98 (dd, $^3J_{HH}$ = 8.5 Hz, $^4J_{HH}$ = 2.6 Hz, 1H, H-C16), 6.94 (d,

$^4J_{HH}$ = 2.5 Hz, 1H, H-C18), 3.05 (t, $^3J_{HF}$ = 13.2 Hz, 2H, H-C26), 2.98 – 2.91 (m, 2H, H-C12), 2.55 – 2.48 (m, 1H, H^a-C2), 2.47 – 2.40 (m, 1H, H^a-C8), 2.32 (td, *J* = 11.1, 4.3 Hz, 1H, H-C9), 2.21 – 2.11 (m, 1H, H^b-C2), 2.11 – 2.01 (m, 2H, H^a-C7, H^a-C11), 2.00 – 1.95 (m, 1H, H^a-C7), 1.80 (t, $^3J_{HF}$ = 18.1 Hz, 3H, H-C28), 1.67 – 1.45 (m, 6H, H-C4, H-C10, H^b-C8, H^b-C7, H^b-C11, H^b-C3), 0.93 (s, 3H, H-C6).

^{13}C NMR (151 MHz, $CDCl_3$) δ 220.9 (C1), 165.0 (C19), 148.9 (C17), 138.3 (C14), 137.7 (C13), 132.0 (C22), 130.1 (C21), 129.3 (C23), 128.3 (C20), 126.6 (C15), 122.0 (t, $^1J_{CF}$ = 241.0 Hz, C27), 121.8 (C18), 118.9 (C16), 85.5 (t, $^3J_{CF}$ = 8.8 Hz, C25), 82.9 (C24), 50.6 (C4), 48.1 (C5), 44.3 (C9), 38.2 (C10), 36.0 (C2), 31.7 (C7), 30.4 (t, $^2J_{CF}$ = 32.2 Hz, C26), 29.6 (C12), 26.5 (C11), 25.9 (C8), 22.9 (t, $^2J_{CF}$ = 26.7 Hz, C28), 21.7 (C3), 14.0 (C6).

^{19}F NMR (564 MHz, $CDCl_3$) δ -89.43 (qt, $^3J_{HF}$ = 18.3, 13.3 Hz, 2F, F-C27).

^{19}F{^{1}H} NMR (564 MHz, $CDCl_3$) δ -89.43 (s, 2F, F-C27).

ESI-MS: (*m/z*) requires: [($C_{30}H_{30}O_3F_2Na)^+$] = 499.2055, (*m/z*) found: [($C_{30}H_{30}O_3F_2Na)^+$] = 499.2053.

FT-IR ($\tilde{\nu}$ = cm^{-1}): 2930 (w), 1732 (s), 1606 (w), 1494 (w), 1454 (w), 1406 (w), 1393 (w), 1258 (s), 1223 (m), 1210 (m), 1175 (m), 1149 (m), 1118 (m), 1102 (w), 1072 (s), 1017 (m), 1009 (w), 963 (w), 937 (w), 911 (m), 878 (w), 858 (w), 818 (w), 782 (w), 765 (m), 732 (m), 692 (w), 649 (w).

Melting Point: 176-178 °C.

2,2-Difluoroheptadec-4-yne (20)

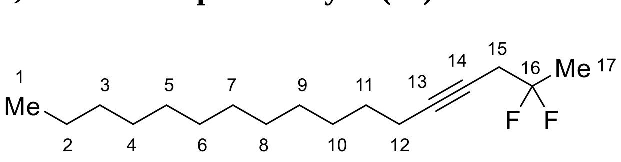

Compound **20** was prepared according to the General Procedure **E** with an amine:HF ratio of 1:6.0 using 2-methylhexadec-1-en-3-yne (**S20**) (46.9 mg, 0.2 mmol, 1.0 eq.). The crude product was purified by column chromatography (*n*-pentane) to yield the title compound as a colorless oil (34.3 mg, 0.13 mmol, 63%).

R$_f$ = 0.70 (*n*-pentane).

^{1}H NMR (500 MHz, $CDCl_3$) δ 2.73 (tt, $^3J_{HF}$ = 13.4 Hz, $^5J_{HH}$ = 2.5 Hz, 2H, H-C15), 2.16 (tt, $^3J_{HH}$ = 7.1 Hz, $^5J_{HH}$ = 2.4 Hz, 2H, H-C12), 1.73 (t, $^3J_{HF}$ = 18.2 Hz, 3H, H-C17), 1.53 – 1.45 (m, 2H, H-C11), 1.41 – 1.33 (m, 2H, H-C10), 1.32 – 1.30 (m, 16H, H-C2~C9), 0.88 (t, $^3J_{HH}$ = 6.9 Hz, 3H, H-C1).

^{13}C NMR (126 MHz, $CDCl_3$) δ 122.6 (t, $^1J_{CF}$ = 240.2 Hz, C16), 84.0 (t, $^4J_{CF}$ = 1.3 Hz, C13), 72.2 (t, $^3J_{CF}$ = 9.1 Hz, C14), 32.1 (CH_2), 29.8 (CH_2), 29.8 (CH_2), 29.8 (CH_2), 29.7 (t, $^2J_{CF}$ = 32.0 Hz, C15), 29.7 (CH_2), 29.5 (CH_2), 29.3 (CH_2), 29.0 (C10), 28.8 (C11), 22.8 (CH_2), 22.6 (t, $^2J_{CF}$ = 26.9 Hz, C17), 18.8 (C12), 14.3 (C1).

^{19}F NMR (470 MHz, $CDCl_3$) δ -90.14 (qt, $^3J_{HF}$ = 18.2, 13.4 Hz, 2F, F-C16).

^{19}F{^{1}H} NMR (470 MHz, $CDCl_3$) δ -90.14 (s, 2F, F-C16).

Analysis by mass spectrometry was inconclusive.

FT-IR ($\tilde{\nu}$ = cm^{-1}): 2924 (s), 2854 (m), 1466 (w), 1390 (m), 1329 (w), 1280 (w), 1237 (s), 1174 (w), 1151 (s), 1138 (s), 1119 (s), 964 (m), 938 (s), 881 (s), 782 (w), 722 (w).

(5,5-Difluorohex-2-yn-1-yl)cyclohexane (21)

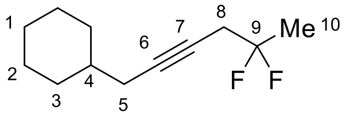

Compound **21** was prepared according to the General Procedure **E** with an amine:HF ratio of 1:6.0 using (4-methylpent-4-en-2-yn-1-yl)cyclohexane (**S21**) (32.5 mg, 0.2 mmol, 1.0 eq.). The crude product was purified by column chromatography (*n*-pentane) to yield the title compound as a colorless oil (19.8 mg, 0.10 mmol, 50%).

R$_f$ = 0.70 (*n*-pentane).

^{1}H NMR (500 MHz, $CDCl_3$) δ 2.74 (tt, $^3J_{HF}$ = 13.4 Hz, $^5J_{HH}$ = 2.5 Hz, 2H, H-C8), 2.07 (dt, $^3J_{HH}$ = 6.6 Hz, $^5J_{HH}$ = 2.4 Hz, 2H, H-C5), 1.81 – 1.62 (m, 8H, H-C10, H-C3, H-C2, H-C1), 1.50 – 1.39 (m, 1H, H-C4), 1.30 – 1.08 (m, 3H, H-C2, H-C1), 1.04 – 0.93 (m, 2H, H-C3).

^{13}C NMR (126 MHz, $CDCl_3$) δ 122.6 (t, $^{1}J_{CF}$ = 240.1 Hz, C9), 82.8 (t, $^{4}J_{CF}$ = 1.3 Hz, C6), 73.1 (t, $^{3}J_{CF}$ = 9.1 Hz, C7), 37.5 (C4), 32.8 (C3), 29.7 (t, $^{2}J_{CF}$ = 31.9 Hz, C8), 26.6 (C5), 26.4 (C1), 26.3 (C2), 22.6 (t, $^{2}J_{CF}$ = 26.9 Hz, C10).

^{19}F NMR (470 MHz, $CDCl_3$) δ -90.11 (qt, $^{3}J_{HF}$ = 18.2, 13.5 Hz, 2F, F-C9).

^{19}F{^{1}H} NMR (470 MHz, $CDCl_3$) δ -90.11 (s, 2F, F-C9).

GC-EI-MS: (*m/z*) requires: [($C_{11}H_{15}F_2$)] = 185.1136 [M-Me], (*m/z*) found: [($C_{11}H_{15}F_2$)] = 185.1136.

FT-IR ($\tilde{\nu}$ = cm^{-1}): 2924 (m), 2853 (w), 1449 (w), 1390 (m), 1279 (w), 1237 (m), 1151 (m), 1138 (s), 1119 (s), 1068 (w), 966 (m), 937 (s), 894 (w), 880 (s), 795 (w), 781 (w).

2-(7,7-Difluorooct-4-yn-1-yl)isoindoline-1,3-dione (22)

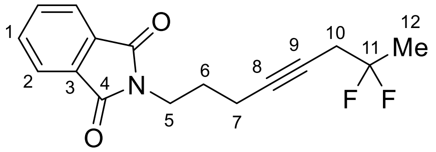

Compound **22** was prepared according to the General Procedure **E** with an amine:HF ratio of 1:6.0 using 2-(6-methylhept-6-en-4-yn-1-yl)isoindoline-1,3-dione (**S22**) (50.7 mg, 0.2 mmol, 1.0 eq.). The crude product was purified by column chromatography (*n*-pentane:Et_2O 3:1) to yield the title compound as a white solid (29.1 mg, 0.10 mmol, 50%).

R*f* = 0.44 (*n*-pentane:Et_2O 2.5:1).

^{1}H NMR (599 MHz, $CDCl_3$) δ 7.85 (dd, $^{3}J_{HH}$ = 5.5 Hz, $^{4}J_{HH}$ = 3.0 Hz, 2H, H-C2), 7.72 (dd, $^{3}J_{HH}$ = 5.5 Hz, $^{4}J_{HH}$ = 3.0 Hz, 2H, H-C1), 3.79 (t, $^{3}J_{HH}$ = 7.1 Hz, 2H, H-C5), 2.63 (tt, $^{3}J_{HF}$ = 13.4 Hz, $^{5}J_{HH}$ = 2.4 Hz, 2H, H-C10), 2.27 (tt, $^{3}J_{HH}$ = 7.0 Hz, $^{5}J_{HH}$ = 2.4 Hz, 2H, H-C7), 1.91 (p, $^{3}J_{HH}$ = 7.1 Hz, 2H, H-C6), 1.69 (t, $^{3}J_{HF}$ = 18.3 Hz, 3H, H-C12).

^{13}C NMR (151 MHz, $CDCl_3$) δ 168.5 (C4), 134.1 (C1), 132.3 (C3), 123.4 (C2), 122.4 (app. t, $^{1}J_{CF}$ = 239.4 Hz, C11), 82.4 (C8), 73.2 (app. t, $^{3}J_{CF}$ = 9.0 Hz, C9), 37.4 (C5), 29.6 (t, $^{2}J_{CF}$ = 32.1 Hz, C10), 27.6 (C6), 22.6 (t, $^{2}J_{CF}$ = 26.7 Hz, C12), 16.7 (C7).

^{19}F NMR (564 MHz, $CDCl_3$) δ -90.01 (qt, $^{3}J_{HF}$ = 18.3, 13.4 Hz, 2F, F-C11).

^{19}F{^{1}H} NMR (564 MHz, $CDCl_3$) δ -90.01 (s, 2F, F-C11).

ESI-MS: (*m/z*) requires: [($C_{16}H_{15}NO_2F_2Na$)$^+$] = 314.0963, (*m/z*) found: [($C_{16}H_{15}NO_2F_2Na$)$^+$] = 314.0963.

FT-IR ($\tilde{\nu}$ = cm^{-1}): 2924 (s), 2854 (m), 1466 (w), 1390 (m), 1329 (w), 1280 (w), 1237 (m), 1174 (w), 1151 (m), 1138 (m), 1119 (s), 964 (m), 938 (s), 881 (m), 782 (w), 722 (w).

Melting Point: 46-48 ˚C.

4-(((14,14-Difluoropentadec-11-yn-1-yl)oxy)methyl)-1,2-difluorobenzene (23)

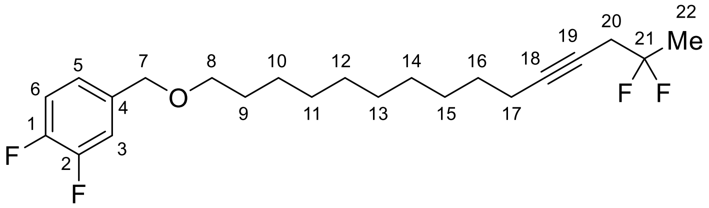

Compound **23** was prepared according to the General Procedure **E** with an amine:HF ratio of 1:6.0 using 1,2-difluoro-4-(((13-methyltetradec-13-en-11-yn-1-yl)oxy)methyl)benzene (**S23**) (69.7 mg, 0.2 mmol, 1.0 eq.). The crude product was purified by column chromatography (*n*-pentane:Et_2O 3:1) to yield the title compound as a colorless oil (54.6 mg, 0.14 mmol, 71%).

R*f* = 0.34 (*n*-pentane:Et_2O 50:1).

^{1}H NMR (599 MHz, $CDCl_3$) δ 7.16 (ddd, $^3J_{HF}$ = 11.1 Hz, $^4J_{HF}$ = 7.7 Hz, $^4J_{HH}$ = 2.1 Hz, 1H, H-C3), 7.11 (ddd, $^3J_{HF}$ = 10.2 Hz, $^3J_{HH}$ = 8.2 Hz, $^4J_{HF}$ = 8.2 Hz, 1H, H-C6), 7.05 – 7.00 (m, 1H, H-C5), 4.43 (s, 2H, H-C7), 3.46 (t, $^3J_{HH}$ = 6.6 Hz, 2H, H-C8), 2.73 (tt, $^3J_{HF}$ = 13.4 Hz, $^5J_{HH}$ = 2.5 Hz, 2H, H-C20), 2.16 (tt, $^3J_{HH}$ = 7.1 Hz, $^5J_{HH}$ = 2.4 Hz, 2H, H-C17), 1.70 (t, $^3J_{HF}$ = 18.3 Hz, 3H, H-C22), 1.61 (p, $^3J_{HH}$ = 6.9 Hz, 2H, H-C9), 1.49 (p, $^3J_{HH}$ = 7.2 Hz, 2H, H-C16), 1.41 – 1.32 (m, 4H, H-C10, H-C15), 1.32 – 1.25 (m, 8H, H-C11~C14).

^{13}C NMR (151 MHz, $CDCl_3$) δ 150.5 (dd, $^1J_{CF}$ = 98.7 Hz, $^2J_{CF}$ = 12.7 Hz, *C*F), 149.8 (dd, $^1J_{CF}$ = 97.9 Hz, $^2J_{CF}$ = 12.7 Hz, *C*F), 136.0 (dd, $^3J_{CF}$ = 5.4 Hz, $^4J_{CF}$ = 3.9 Hz, C4), 123.4 (dd, $^3J_{CF}$ = 6.2 Hz, $^4J_{CF}$ = 3.6 Hz, C5), 122.6 (t, $^1J_{CF}$ = 240.2 Hz, C21), 117.2 (d, $^2J_{CF}$ = 17.2 Hz, C6), 116.5 (d, $^2J_{CF}$ = 17.4 Hz, C3), 84.0 (t, $^4J_{CF}$ = 1.4 Hz, C18), 72.2 (t, $^3J_{CF}$ = 9.0 Hz, C19), 71.7 (d, $^4J_{CF}$ = 1.4 Hz, C7), 70.9 (C8), 29.9 (C9), 29.7 (t, $^2J_{CF}$ = 31.9 Hz, C20), 29.7 (*C*H_2), 29.6 (*C*H_2), 29.6 (*C*H_2), 29.2 (*C*H_2), 28.9 (C15), 28.8 (C16), 26.3 (C10), 22.6 (t, $^2J_{CF}$ = 26.9 Hz, C22), 18.8 (C17).

^{19}**F NMR** (564 MHz, $CDCl_3$) δ -90.15 (qt, $^3J_{HF}$ = 18.3, 13.5 Hz, 2F, F-C21), -138.11 (dddd, $^3J_{FF}$ = 20.8 Hz, $^3J_{HF}$ = 11.2 Hz, $^4J_{HF}$ = 7.9 Hz, $^5J_{HF}$ = 1.4 Hz, 1F, F-C2), -139.98 (dddd, $^3J_{FF}$ = 21.0 Hz, $^3J_{HF}$ = 10.3 Hz, $^4J_{HF}$ = 7.7 Hz, $^4J_{HF}$ = 4.3 Hz, 1F, F-C1).

^{19}**F{^{1}H} NMR** (377 MHz, $CDCl_3$) δ -90.11 (s, 2F, F-C21), -138.08 (d, $^3J_{FF}$ = 21.1 Hz, 1F, F-C2), -139.95 (d, $^3J_{FF}$ = 21.1 Hz, 1F, F-C1).

GC-EI-MS: (*m/z*) requires: [($C_{22}H_{29}OF_4$)] = 386.2183 [M-H], (*m/z*) found: [($C_{22}H_{29}OF_4$)] = 386.2180.

FT-IR ($\tilde{\nu}$ = cm^{-1}): 2929 (m), 2855 (m), 1611 (w), 1520 (s), 1435 (m), 1392 (m), 1360 (w), 1284 (m), 1237 (m), 1208 (m), 1139 (m), 1113 (s), 1101 (s), 964 (m), 937 (m), 880 (m), 816 (m), 781 (m), 749 (w).

(5,5-Difluorohex-2-yn-1-yl)benzene (24)

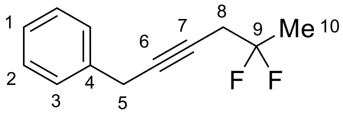

Compound **24** was prepared according to the General Procedure **E** with an amine:HF ratio of 1:6.5 using (4-methylpent-4-en-2-yn-1-yl)benzene (**S24**) (15.6 mg, 0.1 mmol, 1.0 eq.). The crude product was purified by column chromatography (*n*-pentane:Et_2O 30:1) to yield the title compound as a colorless oil (10.0 mg, 0.05 mmol, 51%).

R$_f$ = 0.15 (*n*-pentane).

^{1}**H NMR** (500 MHz, $CDCl_3$) δ 7.35 – 7.31 (m, 4H, H-C2, H-C3), 7.26 – 7.22 (m, 1H, H-C1), 3.61 (t, $^5J_{HH}$ = 2.5 Hz, 2H, H-C5), 2.82 (tt, $^3J_{HF}$ = 13.4 Hz, $^5J_{HH}$ = 2.5 Hz, 2H, H-C8), 1.73 (t, $^3J_{HF}$ = 18.2 Hz, 3H, H-C10).

^{13}**C NMR** (151 MHz, $CDCl_3$) δ 136.8 (C4), 128.7 (C2), 128.0 (C3), 126.8 (C1), 122.4 (t, $^1J_{CF}$ = 240.3 Hz, C9), 81.3 (C6), 74.6 (t, $^3J_{CF}$ = 9.0 Hz, C7), 29.8 (t, $^2J_{CF}$ = 31.9 Hz, C8), 25.2 (C5), 22.7 (t, $^2J_{CF}$ = 26.8 Hz, C10).

^{19}**F NMR** (564 MHz, $CDCl_3$) δ -89.92 (qt, $^3J_{HF}$ = 18.2, 13.4 Hz, 2F, F-C9).

^{19}**F{^{1}H} NMR** (564 MHz, $CDCl_3$) δ -89.92 (s, 2F, F-C9).

GC-EI-MS: (*m/z*) requires: [($C_{12}H_{12}F_2$)] = 194.0902, (*m/z*) found: [($C_{12}H_{12}F_2$)] = 194.0902.

FT-IR ($\tilde{\nu}$ = cm^{-1}): 2924 (w), 1603 (w), 1495 (w), 1454 (w), 1422 (w), 1392 (m), 1333 (w), 1280 (w), 1235 (m), 1175 (w), 1148 (m), 1136 (m), 1118 (m), 1030 (w), 964 (m), 936 (m), 880 (m), 795 (w), 781 (w), 729 (s), 695 (s), 606 (w).

14,14-Difluoropentadec-11-yn-1-yl (*E*)-3-(4-bromophenyl)acrylate (25)

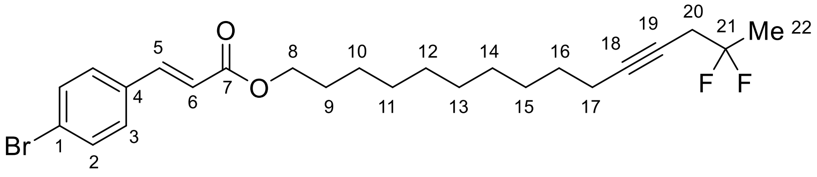

Compound **25** was prepared according to the General Procedure **E** with an amine:HF ratio of 1:6.0 using 13-methyltetradec-13-en-11-yn-1-yl (*E*)-3-(4-bromophenyl)acrylate (**S25**) (43.1 mg, 0.1 mmol, 1.0 eq.). The crude product was purified by column chromatography (*n*-pentane:Et_2O 15:1) to yield the title compound as a colorless oil (29.9 mg, 0.06 mmol, 64%).

R*f* = 0.39 (*n*-pentane:Et_2O 15:1).

^{1}H NMR (599 MHz, $CDCl_3$) δ 7.61 (d, $^3J_{HH}$ = 16.0 Hz, 1H, H-C5), 7.54 – 7.50 (m, 2H, H-C2), 7.41 – 7.36 (m, 2H, H-C3), 6.42 (d, $^3J_{HH}$ = 16.0 Hz, 1H, H-C6), 4.20 (t, $^3J_{HH}$ = 6.7 Hz, 2H, H-C8), 2.73 (tt, $^3J_{HF}$ = 13.5 Hz, $^5J_{HH}$ = 2.5 Hz, 2H, H-C20), 2.16 (tt, $^3J_{HH}$ = 7.1 Hz, $^5J_{HH}$ = 2.4 Hz, 2H, H-C17), 1.74 – 1.66 (m, 5H, H-C22, H-C9), 1.53 – 1.45 (m, 2H, H-C16), 1.42 – 1.25 (m, 12H, H-C10~C15).

^{13}C NMR (151 MHz, $CDCl_3$) δ 167.0 (C7), 143.3 (C5), 133.6 (C1), 132.3 (C2), 129.6 (C3), 124.6 (C4), 122.5 (t, $^1J_{CF}$ = 240.2 Hz, C21), 119.2 (C6), 83.9 (C18), 72.3 (app. t, $^3J_{CF}$ = 9.0 Hz, C19), 65.0 (C8), 29.7 (t, $^2J_{CF}$ = 31.9 Hz, C20), 29.6 (CH_2), 29.6 (CH_2), 29.4 (CH_2), 29.2 (CH_2), 28.9 (C15), 28.6 (C16), 28.8 (C9), 26.1 (C10), 22.6 (t, $^2J_{CF}$ = 26.9 Hz, C22), 18.8 (C17).

^{19}F NMR (564 MHz, $CDCl_3$) δ -90.13 (qt, $^3J_{HF}$ = 18.2, 13.4 Hz, 2F, F-C21).

^{19}F{^{1}H} NMR (564 MHz, $CDCl_3$) δ -90.13 (s, 2F, F-C21).

ESI-MS: (*m/z*) requires: [($C_{24}H_{31}O_2{}^{79}BrF_2Na)^+$] = 491.1368, (*m/z*) found: [($C_{24}H_{31}O_2{}^{79}BrF_2Na)^+$] = 491.1367.

FT-IR ($\tilde{\nu}$ = cm^{-1}): 2927 (m), 2855 (w), 1712 (s), 1637 (m), 1587 (w), 1488 (m), 1466 (w), 1403 (w), 1390 (m), 1309 (m), 1270 (m), 1237 (m), 1201 (m), 1169 (s), 1138 (m), 1119 (m), 1072 (s), 1009 (m), 981 (m), 964 (m), 936 (m), 880 (m), 818 (s), 795 (w), 782 (w), 753 (w), 722 (w).

14,14-Difluoropentadec-11-yn-1-yl hex-2-ynoate (26)

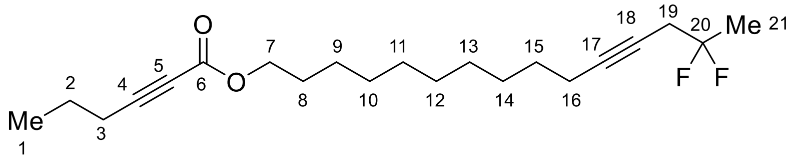

Compound **26** was prepared according to the General Procedure **E** with an amine:HF ratio of 1:6.0 using 13-methyltetradec-13-en-11-yn-1-yl hex-2-ynoate (**S26**) (31.7 mg, 0.1 mmol, 1.0 eq.). The crude product was purified by column chromatography (*n*-pentane:Et_2O 20:1) to yield the title compound as a colorless oil (22.5 mg, 0.06 mmol, 63%).

$\mathbf{R}_f$ = 0.36 (*n*-pentane:Et_2O 20:1).

^{1}H NMR (500 MHz, $CDCl_3$) δ 4.14 (t, $^3J_{HH}$ = 6.8 Hz, 2H, H-C7), 2.73 (tt, $^3J_{HF}$ = 13.4 Hz, $^5J_{HH}$ = 2.6 Hz, 2H, H-C19), 2.31 (t, $^3J_{HH}$ = 7.1 Hz, 2H, H-C3), 2.20 – 2.12 (m, 2H, H-C16), 1.74 – 1.57 (m, 7H, H-C21, H-C8, H-C2), 1.52 – 1.45 (m, 2H, H-C15), 1.40 – 1.25 (m, 12H, H-C9~C14), 1.01 (t, $^3J_{HH}$ = 7.4 Hz, 3H, H-C1).

^{13}C NMR (126 MHz, $CDCl_3$) δ 154.2 (C6), 122.5 (t, $^1J_{CF}$ = 240.1 Hz, C20), 89.4 (C4), 83.9 (t, $^4J_{CF}$ = 1.3 Hz, C17), 73.5 (C5), 72.3 (t, $^3J_{CF}$ = 9.0 Hz, C18), 66.1 (C7), 29.7 (t, $^2J_{CF}$ = 31.8 Hz, C19), 29.5 (*C*H_2, *overlapping*), 29.3 (*C*H_2), 29.2 (*C*H_2), 28.9 (C14), 28.8 (C15), 28.6 (C8), 25.9 (*C*H_2), 22.6 (t, $^2J_{CF}$ = 26.8 Hz, C21), 21.2 (C2), 20.8 (C3), 18.8 (C16), 13.6 (C1).

^{19}F NMR (470 MHz, $CDCl_3$) δ -90.14 (qt, $^3J_{HF}$ = 18.2, 13.4 Hz, 2F, F-C20).

^{19}F{^{1}H} NMR (470 MHz, $CDCl_3$) δ -90.14 (s, 2F, F-C20).

ESI-MS: (*m/z*) requires: [$(C_{21}H_{32}O_2F_2Na)^+$] = 377.2263, (*m/z*) found: [$(C_{21}H_{32}O_2F_2Na)^+$] = 377.2262.

FT-IR ($\tilde{\nu}$ = cm^{-1}): 2929 (m), 2857 (w), 2234 (w), 1709 (s), 1465 (w), 1392 (w), 1327 (w), 1240 (s), 1151 (m), 1138 (m), 1119 (m), 1082 (m), 964 (w), 937 (m), 880 (m), 752 (m).

14,14-Difluoropentadec-11-yn-1-yl 4-methylbenzenesulfonate (27)

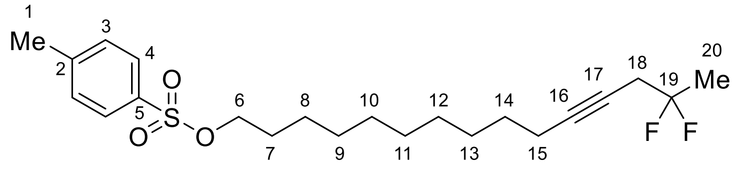

Compound **27** was prepared according to the General Procedure **E** with an amine:HF ratio of 1:6.0 using 13-methyltetradec-13-en-11-yn-1-yl 4-methylbenzenesulfonate (**S27**) (75.3 mg, 0.2 mmol, 1.0 eq.). The crude product was purified by column chromatography (*n*-pentane:Et_2O 5:1) to yield the title compound as a colorless oil (46.8 mg, 0.11 mmol, 56%).

R*f* = 0.37 (*n*-pentane:Et_2O 5:1).

^{1}H NMR (500 MHz, $CDCl_3$) δ 7.81 – 7.76 (m, 2H, H-C4), 7.37 – 7.32 (m, 2H, H-C3), 4.02 (t, $^3J_{HH}$ = 6.5 Hz, 2H, H-C6), 2.73 (tt, $^3J_{HF}$ = 13.8 Hz, $^5J_{HH}$ = 2.5 Hz, 2H, H-C18), 2.45 (s, 3H, H-C1), 2.16 (tt, $^3J_{HH}$ = 7.1 Hz, $^5J_{HH}$ = 2.4 Hz, 2H, H-C15), 1.69 (t, $^3J_{HF}$ = 18.3 Hz, 3H, H-C20), 1.65 – 1.60 (m, 2H, H-C7), 1.52 – 1.44 (m, 2H, H-C14), 1.39 – 1.31 (m, 2H, H-C13), 1.31 – 1.18 (m, 10H, H-C8~C12).

^{13}C NMR (126 MHz, $CDCl_3$) δ 144.8 (C5), 133.4 (C2), 129.9 (C3), 128.0 (C4), 122.5 (t, $^1J_{CF}$ = 240.1 Hz, C19), 83.9 (C16), 72.3 (t, $^3J_{CF}$ = 9.0 Hz, C17), 70.8 (C6), 29.7 (t, $^2J_{CF}$ = 31.9 Hz, C18), 29.5 (CH_2), 29.4 (CH_2), 29.2 (CH_2), 29.0 (CH_2), 29.0 (C7), 28.9 (C13), 28.8 (C14), 25.5 (CH_2), 22.6 (t, $^2J_{CF}$ = 26.8 Hz, C20), 21.8 (C1), 18.8 (C15).

^{19}F NMR (470 MHz, $CDCl_3$) δ -90.15 (qt, $^3J_{HF}$ = 18.2, 13.5 Hz, 2F, F-C19).

^{19}F{^{1}H} NMR (470 MHz, $CDCl_3$) δ -90.15 (s, 2F, F-C19).

ESI-MS: (*m/z*) requires: [$(C_{22}H_{32}O_3SF_2Na)^+$] = 437.1932, (*m/z*) found: [$(C_{22}H_{32}O_3SF_2Na)^+$] = 437.1931.

FT-IR ($\tilde{\nu}$ = cm^{-1}): 2927 (w), 2857 (w), 1598 (w), 1392 (w), 1359 (m), 1237 (m), 1188 (m), 1175 (s), 1151 (m), 1138 (m), 1119 (m), 1098 (m), 1020 (w), 963 (m), 936 (m), 880 (m), 814 (m), 794 (w), 781 (m), 725 (w), 689 (w), 663 (s).

14,14-Difluoropentadec-11-yn-1-ol (28)

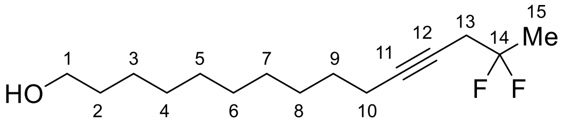

Compound **28** was prepared according to the General Procedure **E** with an amine:HF ratio of 1:6.0 using 13-methyltetradec-13-en-11-yn-1-ol (**S28**) (44.5 mg, 0.2 mmol, 1.0 eq.). The crude product was purified by column chromatography (*n*-pentane:Et_2O 1:1) to yield the title compound as a colorless oil (21.5 mg, 0.08 mmol, 41%).

R*f* = 0.49 (*n*-pentane:Et_2O 1:1).

^{1}H NMR (500 MHz, $CDCl_3$) δ 3.64 (t, $^3J_{HH}$ = 6.7 Hz, 2H, H-C1), 2.73 (tt, $^3J_{HF}$ = 13.5 Hz, $^5J_{HH}$ = 2.5 Hz, 2H, H-C13), 2.16 (tt, $^3J_{HH}$ = 7.2 Hz, $^5J_{HH}$ = 2.4 Hz, 2H, H-C10), 1.70 (t, $^3J_{HF}$ = 18.2 Hz, 3H, H-C15), 1.58 – 1.53 (m, 2H, H-C2), 1.53 – 1.45 (m, 2H, H-C9), 1.40 – 1.22 (m, 13H, H-C3~C8, OH).

^{13}C NMR (126 MHz, $CDCl_3$) δ 122.6 (t, $^1J_{CF}$ = 240.1 Hz, C14), 84.0 (t, $^4J_{CF}$ = 1.3 Hz, C11), 72.2 (t, $^3J_{CF}$ = 9.1 Hz, C12), 63.2 (C1), 33.0 (C2), 29.7 (t, $^2J_{CF}$ = 31.8 Hz, C13), 29.7 (CH_2), 29.6 (CH_2), 29.5 (CH_2), 29.2 (CH_2), 28.9 (CH_2), 28.8 (C9), 25.9 (CH_2), 22.6 (t, $^2J_{CF}$ = 26.9 Hz, C15), 18.8 (C10).

^{19}F NMR (470 MHz, $CDCl_3$) δ -90.13 (qt, $^3J_{HF}$ = 18.2, 13.5 Hz, 2F, F-C14).

^{19}F{^{1}H} NMR (470 MHz, $CDCl_3$) δ -90.13 (s, 2F, F-C14).

ESI-MS: (*m/z*) requires: [$(C_{15}H_{26}OF_2Na)^+$] = 283.1844, (*m/z*) found: [$(C_{15}H_{26}OF_2Na)^+$] = 283.1843.

FT-IR ($\tilde{\nu}$ = cm^{-1}): 3343 (w), 2927 (s), 2855 (s), 1459 (w), 1392 (m), 1237 (m), 1152 (m), 1139 (m), 1119 (m), 1058 (s), 964 (w), 937 (m), 908 (w), 881 (w), 758 (m), 735 (w).

3,3-Difluoro-2-methylnonadec-5-yne (29)

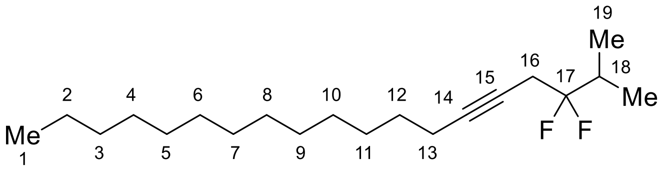

Compound **29** was prepared according to the General Procedure **E** with an amine:HF ratio of 1:6.0 using 2-methyl-3-methyleneoctadec-4-yne (**S29**) (55.3

mg, 0.2 mmol, 1.0 eq.). The crude product was purified by column chromatography (*n*-pentane) to yield the title compound as a colorless oil (34.3 mg, 0.11 mmol, 55%).

R*f* = 0.71 (*n*-pentane).

^{1}H NMR (400 MHz, $CDCl_3$) δ 2.74 (tt, $^3J_{HF}$ = 14.5 Hz, $^5J_{HH}$ = 2.4 Hz, 2H, H-C16), 2.44 – 2.22 (m, 1H, H-C18), 2.16 (tt, $^3J_{HH}$ = 7.1 Hz, $^5J_{HH}$ = 2.5 Hz, 2H, H-C13), 1.48 (p, J = 7.2 Hz, 2H, H-C12), 1.39 – 1.22 (m, 20H, H-C2~C11), 1.04 (d, $^3J_{HH}$ = 6.9 Hz, 6H, H-C19), 1.04 (t, $^3J_{HH}$ = 7.0 Hz, 3H, H-C1).

^{13}C NMR (126 MHz, $CDCl_3$) δ 124.7 (t, $^1J_{CF}$ = 245.3 Hz, C17), 83.8 (C14), 72.0 (t, $^3J_{CF}$ = 8.8 Hz, C15), 33.3 (t, $^2J_{CF}$ = 23.9 Hz, C18), 32.1 (CH_2), 29.8 (CH_2), 29.8 (CH_2), 29.8 (CH_2), 29.8 (CH_2), 29.7 (CH_2), 29.5 (CH_2), 29.3 (CH_2), 29.0 (CH_2), 28.9 (C12), 26.5 (t, $^2J_{CF}$ = 31.9 Hz, C16), 22.8 (CH_2), 18.8 (C13), 15.7 (t, $^3J_{CF}$ = 4.9 Hz, C19), 14.3 (C1).

^{19}F NMR (470 MHz, $CDCl_3$) δ -105.63 (q, $^3J_{HF}$ = 14.4 Hz, 2F, F-C17).

^{19}F{^{1}H} NMR (377 MHz, $CDCl_3$) δ -105.63 (s, 2F, F-C17).

GC-EI-MS: (*m/z*) requires: [($C_{20}H_{36}F_2$)] = 314.2780, (*m/z*) found: [($C_{20}H_{36}F_2$)] = 314.2784.

FT-IR ($\tilde{\nu}$ = cm^{-1}): 2923 (s), 2854 (m), 1466 (w), 1254 (w), 1208 (w), 1169 (m), 1157 (w), 1078 (s), 1004 (s), 903 (w), 874 (m), 722 (w), 634 (w).

(1,1-Difluoroheptadec-3-yn-1-yl)cyclohexane (30)

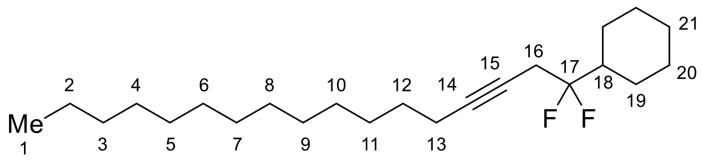

Compound **30** was prepared according to the General Procedure **E** with an amine:HF ratio of 1:6.0 using heptadec-1-en-3-yn-2-ylcyclohexane (**S30**) (63.3 mg, 0.2 mmol, 1.0 eq.). The crude product was purified by column chromatography (*n*-pentane) to yield the title compound as a colorless oil (37.8 mg, 0.11 mmol, 53%).

R*f* = 0.70 (*n*-pentane).

^{1}H NMR (500 MHz, $CDCl_3$) δ 2.73 (tt, $^3J_{HF}$ = 14.6 Hz, $^5J_{HH}$ = 2.4 Hz, 2H, H-C16), 2.16 (tt, $^3J_{HH}$ = 7.1 Hz, $^5J_{HH}$ = 2.4 Hz, 2H, H-C13), 2.07 – 1.93 (m, 1H, H-C18), 1.91 – 1.76 (m, 4H, H-C19, H-C20), 1.73 – 1.65 (m, 1H, H-C21), 1.53 – 1.45 (m, 2H, H-C12), 1.41 – 1.34 (m, 2H, H-C11), 1.32 – 1.18 (m, 23H, H-C2~C10, H-C19~C21), 0.88 (t, $^3J_{HH}$ = 6.9 Hz, 3H, H-C1).

^{13}C NMR (126 MHz, $CDCl_3$) δ 124.2 (t, $^1J_{CF}$ = 245.0 Hz, C17), 83.8 (C14), 72.1 (app. t, $^3J_{CF}$ = 9.0 Hz, C15), 42.7 (t, $^2J_{CF}$ = 22.9 Hz, C18), 32.1 (*C*H_2), 29.8 (*C*H_2), 29.8 (*C*H_2), 29.8 (*C*H_2), 29.8 (*C*H_2), 29.7 (*C*H_2), 29.5 (*C*H_2), 29.3 (*C*H_2), 29.0 (C11), 28.9 (C12), 26.5 (t, $^2J_{CF}$ = 32.1 Hz, C16), 26.1 (C21), 25.9 (C20), 25.6 (t, $^3J_{CF}$ = 4.3 Hz, C19), 22.9 (*C*H_2), 18.8 (C13), 14.3 (C1).

^{19}F NMR (470 MHz, $CDCl_3$) δ -104.14 (q, $^3J_{HF}$ = 14.5 Hz, 2F, F-C17).

^{19}F{^{1}H} NMR (470 MHz, $CDCl_3$) δ -104.14 (s, 2F, F-C17).

GC-EI-MS: (*m/z*) requires: [($C_{23}H_{40}F_2$)] = 354.3093, (*m/z*) found: [($C_{23}H_{40}F_2$)] = 354.3089.

FT-IR ($\tilde{\nu}$ = cm^{-1}): 2923 (s), 2854 (s), 1466 (w), 1454 (m), 1379 (w), 1324 (w), 1260 (w), 1237 (w), 1211 (w), 1154 (m), 1128 (m), 1078 (m), 1043 (s), 992 (m), 897 (w), 872 (m), 860 (w), 831 (w), 772 (w), 722 (w), 634 (m).

1,2,2-Trifluoroheptadec-4-yne (31)

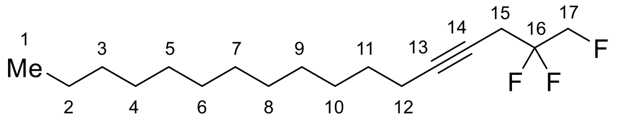

Compound **31** was prepared according to the General Procedure **E** with an amine:HF ratio of 1:6.0 using 2-(fluoromethyl)hexadec-1-en-3-yne (**S31**) (50.5 mg, 0.2 mmol, 1.0 eq.). The crude product was purified by column chromatography (*n*-pentane) to yield the title compound as a colorless oil (38.0 mg, 0.13 mmol, 65%).

R*f* = 0.35 (*n*-pentane).

^{1}H NMR (500 MHz, $CDCl_3$) δ 4.58 (dt, $^2J_{HF}$ = 46.3 Hz, $^3J_{HF}$ = 11.3 Hz, 2H, H-C17), 2.88 (tq, $^3J_{HF}$ = 14.1 Hz, $^4J_{HF}$ = 2.5 Hz, $^5J_{HH}$ = 2.5 Hz, 2H, H-C15), 2.16 (tt, $^3J_{HH}$ = 7.1 Hz, $^5J_{HH}$ = 2.4

Hz, 2H, H-C12), 1.49 (p, $^{3}J_{HH}$ = 7.1 Hz, 2H, H-C11), 1.41 – 1.32 (m, 2H, H-C10), 1.32 – 1.23 (m, 16H, H-C2~C9), 0.88 (t, $^{3}J_{HH}$ = 6.9 Hz, 3H, H-C1).

^{13}C NMR (126 MHz, $CDCl_3$) δ 119.2 (td, $^{1}J_{CF}$ = 244.6 Hz, $^{2}J_{CF}$ = 22.5 Hz, C16), 84.7 (t, $^{4}J_{CF}$ = 1.2 Hz, C13), 80.4 (dt, $^{1}J_{CF}$ = 178.3 Hz, $^{2}J_{CF}$ = 35.2 Hz, C17), 70.5 (t, $^{3}J_{CF}$ = 9.0 Hz, C14), 32.1 (CH_2), 29.8 (CH_2), 29.8 (CH_2), 29.8 (CH_2), 29.7 (CH_2), 29.5 (CH_2), 29.3 (CH_2), 29.0 (C10), 28.7 (C11), 25.2 (td, $^{2}J_{CF}$ = 29.3 Hz, $^{3}J_{CF}$ = 1.2 Hz, C15), 22.8 (CH_2), 18.8 (C12), 14.3 (C1).

^{19}F NMR (470 MHz, $CDCl_3$) δ -108.80 (qt, $^{3}J_{FF}$ = 14.3 Hz, $^{3}J_{HF}$ = 14.3, 11.3 Hz, 2F, F-C16), -235.74 – -236.07 (m, 1F, F-C17).

^{19}F{^{1}H} NMR (470 MHz, $CDCl_3$) δ -108.80 (d, $^{3}J_{FF}$ = 14.7 Hz, 2F, F-C16), -235.89 (t, $^{3}J_{FF}$ = 14.8 Hz, 1F, F-C17).

Analysis by mass spectrometry was inconclusive.

FT-IR ($\tilde{\nu}$ = cm^{-1}): 2924 (s), 2854 (m), 1456 (w), 1290 (w), 1260 (w), 1220 (m), 1152 (m), 1095 (m), 1060 (s), 960 (m), 941 (w), 882 (m), 772 (s).

14,14-Difluoropentadec-11-yn-1-yl-2-(3-cyano-4-isobutoxyphenyl)-4-methylthiazole-5-carboxylate (32)

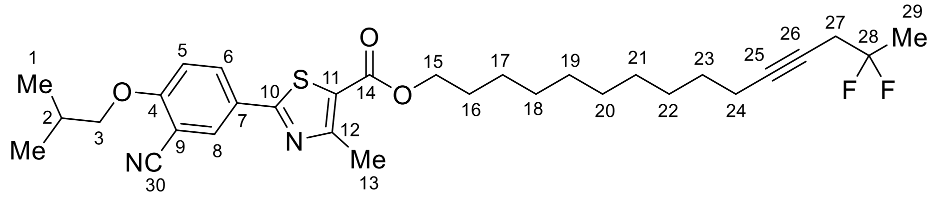

Compound **32** was prepared according to the General Procedure **E** with an amine:HF ratio of 1:6.0 using 13-methyltetradec-13-en-11-yn-1-yl-2-(3-cyano-4-isobutoxyphenyl)-4-methylthiazole-5-carboxylate (**S32**) (52.1 mg, 0.1 mmol, 1.0 eq.). The crude product was purified by column chromatography (*n*-pentane:Et_2O 2:1) to yield the title compound as a white waxy solid (30.8 mg, 0.06 mmol, 55%).

R*f* = 0.34 (*n*-pentane:Et_2O 2:1).

^{1}**H NMR** (599 MHz, $CDCl_3$) δ 8.17 (d, $^{4}J_{HH}$ = 2.3 Hz, 1H, H-C8), 8.09 (dd, $^{3}J_{HH}$ = 8.8 Hz, $^{4}J_{HH}$ = 2.3 Hz, 1H, H-C6), 7.00 (d, $^{3}J_{HH}$ = 8.9 Hz, 1H, H-C5), 4.28 (t, $^{3}J_{HH}$ = 6.7 Hz, 2H, H-C15), 3.90 (d, $^{3}J_{HH}$ = 6.5 Hz, 2H, H-C3), 2.85 – 2.62 (m, 5H, H-C13, H-27), 2.23 – 2.17 (m, 1H, H-C2), 2.16 (tt, $^{3}J_{HH}$ = 7.1 Hz, $^{5}J_{HH}$ = 2.4 Hz, 2H, H-C24), 1.80 – 1.62 (m, 5H, H-C29, H-C16), 1.49 (p, $^{3}J_{HH}$ = 7.1 Hz, 2H, H-C23), 1.45 – 1.39 (m, 2H, H-C17), 1.39 – 1.27 (m, 10H, H-C18~C22), 1.09 (d, $^{3}J_{HH}$ = 6.7 Hz, 6H, H-C1).

^{13}**C NMR** (151 MHz, $CDCl_3$) δ 167.3 (C10), 162.6 (C4), 162.3 (C14), 161.2 (C12), 132.7 (C6), 132.2 (C8), 126.2 (C7), 122.5 (t, $^{1}J_{CF}$ = 240.1 Hz, C28), 122.1 (C11), 115.5 (C30), 112.8 (C5), 103.1 (C9), 83.9 (C25), 75.8 (C3), 72.3 (t, $^{3}J_{CF}$ = 9.0 Hz, C26), 65.6 (C15), 29.7 (t, $^{2}J_{CF}$ = 31.8 Hz, C27), 29.6 (CH_2), 29.6 (CH_2), 29.3 (CH_2), 29.2 (CH_2), 28.9 (C23), 28.8 (C16), 28.3 (C2), 26.1 (C17), 22.6 (t, $^{2}J_{CF}$ = 26.8 Hz, C29), 19.2 (C1), 18.8 (C24), 17.6 (C13).

^{19}**F NMR** (564 MHz, $CDCl_3$) δ -90.13 (qt, $^{3}J_{HF}$ = 18.2, 13.5 Hz, 2F, F-C28).

^{19}**F{^{1}H} NMR** (564 MHz, $CDCl_3$) δ -90.13 (s, 2F, F-C28).

ESI-MS: (*m/z*) requires: $[(C_{31}H_{40}N_2O_3SF_2Na)^+]$ = 581.2620, (*m/z*) found: $[(C_{31}H_{40}N_2O_3SF_2Na)^+]$ = 581.2619.

FT-IR ($\tilde{\nu}$ = cm^{-1}): 2929 (m), 2857 (w), 1713 (m), 1606 (m), 1508 (m), 1433 (m), 1390 (m), 1372 (m), 1329 (m), 1260 (s), 1171 (w), 1093 (s), 1013 (m), 964 (w), 937 (w), 880 (w), 821 (w), 772 (m), 762 (m).

(4,4-Difluorodec-1-yn-1-yl)triisopropylsilane (33)

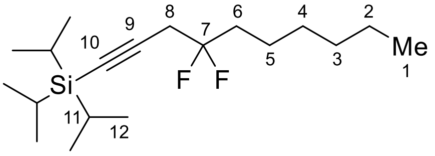

Compound **33** was prepared according to the General Procedure **E** with an amine:HF ratio of 1:7.0 using triisopropyl(3-methylenenon-1-yn-1-yl)silane (**S33**) (58.5 mg, 0.1 mmol, 1.0 eq.). The crude product was purified by column chromatography (*n*-pentane) to yield the title compound as a colorless oil (44.1 mg, 0.13 mmol, 67%).

$\mathbf{R}_f$ = 0.57 (*n*-pentane).

^{1}H NMR (500 MHz, $CDCl_3$) δ 2.83 (t, $^{3}J_{HF}$ = 13.5 Hz, 2H, H-C8), 2.07 – 1.95 (m, 2H, H-C6), 1.52 – 1.45 (m, 2H, H-C5), 1.39 – 1.24 (m, 6H, H-C2~C4), 1.10 – 1.04 (m, 21H, H-C11, H-C12), 0.93 – 0.86 (m, 3H, H-C1).

^{13}C NMR (126 MHz, $CDCl_3$) δ 123.5 (t, $^{1}J_{CF}$ = 243.2 Hz, C7), 100.2 (t, $^{3}J_{CF}$ = 8.7 Hz, C9), 84.6 (C10), 35.5 (t, $^{2}J_{CF}$ = 24.2 Hz, C8), 31.7 (C2/3/4), 29.2 (t, $^{2}J_{CF}$ = 32.4 Hz, C6), 29.1 (C2/3/4), 22.6 (C2/3/4), 22.3 (t, $^{3}J_{CF}$ = 4.6 Hz, C5), 18.7 (C11), 14.2 (C1), 11.3 (C12).

^{19}F NMR (470 MHz, $CDCl_3$) δ -96.09 (tt, $^{3}J_{HF}$ = 16.4, 13.5 Hz, F-C7).

^{19}F{^{1}H} NMR (564 MHz, $CDCl_3$) δ -96.09 (s, 2F, F-C7).

GC-EI-MS: (*m/z*) requires: [($C_{19}H_{36}F_2Si$)] = 330.2549, (*m/z*) found: [($C_{19}H_{36}F_2Si$)] = 330.2550.

FT-IR ($\tilde{\nu}$ = cm^{-1}) 2957 (w), 2866 (s), 2184 (m), 1464 (s), 1419 (w), 1383 (m), 1367 (w), 1270 (w), 1256 (w), 1149 (w), 1075 (m), 1033 (s), 996 (w), 920 (w), 882 (s), 677 (m), 666 (m), 623 (m).

1.4 Synthetic applications

1.4.1 Product derivatisation

2,2-Difluoroheptadecane (34)

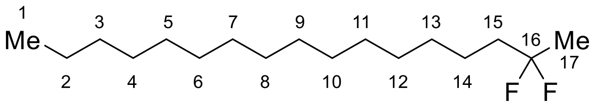

2,2-Difluoroheptadec-4-yne (**20**) (81.7 mg, 0.30 mmol, 1.0 eq.) and palladium on carbon (10% w/w. 31.8 mg, 0.03 mmol, 10 mol%) were placed in a Schlenk flask. The flask was evacuated and refilled with hydrogen through a balloon. After addition of 3.0 mL of methanol, the mixture was stirred at room temperature for 24 h under balloon pressure of hydrogen. The reaction mixture was filtered through celite and washed with Et_2O. After removal of solvent, the residue was purified by column chromatography (*n*-pentane) to yield the title compound as a colorless oil (66.0 mg, 0.24 mmol, 80%).

R*f* = 0.70 (*n*-pentane).

^{1}H NMR (400 MHz, $CDCl_3$) δ 1.92 – 1.71 (m, 2H, H-C15), 1.57 (t, $^{3}J_{HF}$ = 18.4 Hz, 3H, H-C17), 1.51 – 1.40 (m, 2H, H-C14), 1.34 – 1.24 (m, 24H, H-C2~C13), 0.88 (t, $^{3}J_{HH}$ = 6.9 Hz,

3H, H-C1).

^{13}C NMR (151 MHz, $CDCl_3$) δ 124.6 (t, $^1J_{CF}$ = 237.8 Hz, C16), 38.1 (t, $^2J_{CF}$ = 25.3 Hz, C15), 32.1 (*C*H_2), 29.9 (*C*H_2), 29.8 (*C*H_2), 29.8 (*C*H_2), 29.8 (*C*H_2), 29.8 (*C*H_2), 29.8 (*C*H_2), 29.6 (*C*H_2), 29.6 (*C*H_2), 29.5 (*C*H_2), 29.5 (*C*H_2), 23.3 (t, $^2J_{CF}$ = 28.2 Hz, C17), 22.9 (t, $^3J_{CF}$ = 4.7 Hz, C14), 22.6 (*C*H_2), 14.3 (C1).

^{19}F NMR (564 MHz, $CDCl_3$) δ -90.33 (qt, $^3J_{HF}$ = 18.4, 16.0 Hz, 2F, F-C16).

^{19}F{^{1}H} NMR (564 MHz, $CDCl_3$) δ -90.33 (s, 2F, F-C16).

Analysis by mass spectrometry was inconclusive.

FT-IR ($\tilde{\nu}$ = cm^{-1}): 2956 (m), 2923 (s), 2854 (s), 1468 (m), 1390 (m), 1241 (m), 1174 (w), 1139 (m), 1118 (w), 1066 (m), 1058 (m), 1027 (w), 959 (w), 911 (m), 773 (w), 722 (w).

(*Z*)-2,2-Difluoroheptadec-4-ene (35)

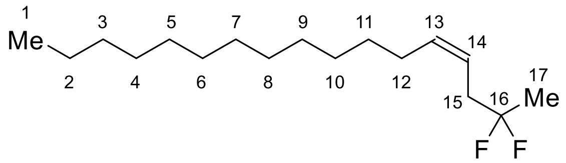

To a solution of titanocene dichloride (2.5 mg, 0.01 mmol, 10 mol%) dissolved in 0.5 mL of THF in a flame-dried flask under argon atmosphere, was added 2,2-difluoroheptadec-4-yne (**20**) (27.2 mg, 0.10 mmol, 1.0 eq.) at room temperature. The resulting solution was allowed to stir for 20 min before cooling down to 0 °C. After that, lithium aluminum hydride (7.6 mg, 0.20 mmol, 2 eq.) was added slowly to the reaction mixture. The resulting mixture was allowed to stir at room temperature overnight before cooling back to 0 °C. The reaction was quenched by the successive addition of water, 5% NaOH (aq), and water. The mixture was filtered and then concentrated under reduced pressure. The residue was purified by column chromatography (*n*-pentane) to yield the title compound as a colorless oil (17.3 mg, 0.06 mmol, 63%, *Z*:*E* = 87:13).

R*f* = 0.70 (*n*-pentane).

^{1}H NMR (599 MHz, $CDCl_3$) δ 5.66 – 5.60 (m, 1H, H-C13, *major*, *Z*-alkene), 5.60 – 5.56 (m, 1H, H-C13, *minor*, *E*-alkene), 5.42 – 5.37 (m, 1H, H-C14), 2.61 (td, $^3J_{HF}$ = 15.4 Hz, $^3J_{HH}$ = 7.5 Hz, 2H, H-C15, *major*, *Z*-alkene), 2.53 (td, $^3J_{HF}$ = 15.3 Hz, $^3J_{HH}$ = 7.2 Hz, 2H, H-C15, *minor*,

E-alkene), 2.04 (q, $^3J_{HH}$ = 7.4 Hz, 2H, H-C12), 1.57 (t, $^3J_{HF}$ = 18.4 Hz, 3H, H-C17), 1.36 (p, $^3J_{HH}$ = 7.4 Hz, 2H, H-C11), 1.30 – 1.24 (m, 18H, H-C2~C10), 0.88 (t, $^3J_{HH}$ = 7.0 Hz, 3H, H-C1).

^{13}C NMR (151 MHz, $CDCl_3$, *Z*-alkene) δ 135.0 (C13), 124.0 (t, $^1J_{CF}$ = 239.0 Hz, C16), 120.5 (t, $^3J_{CF}$ = 5.8 Hz, C14), 36.3 (t, $^2J_{CF}$ = 26.3 Hz, C15), 32.1 (CH_2), 29.8 (CH_2), 29.8 (CH_2), 29.8 (CH_2), 29.8 (CH_2), 29.7 (CH_2), 29.5 (CH_2), 29.5 (CH_2), 29.4 (CH_2), 27.5 (C12), 23.0 (t, $^2J_{CF}$ = 27.6 Hz, C17), 22.6 (CH_2), 14.3 (C1).

^{19}F NMR (564 MHz, $CDCl_3$) δ -89.62 (qtd, $^3J_{HF}$ = 18.4, 15.4 Hz, $^4J_{HF}$ =1.1 Hz, 2F, F-C16, *major*, *Z*-alkene), -90.01 (qt, $^3J_{HF}$ = 18.4, 15.3 Hz, 2F, F-C16, *minor*, *E*-alkene).

^{19}F{^{1}H} NMR (564 MHz, $CDCl_3$) δ -89.62 (s, 2F, F-C16, *major*, *Z*-alkene), -90.01 (s, 2F, F-C16, *minor*, *E*-alkene).

GC-EI-MS: (*m/z*) requires: [($C_{17}H_{32}F_2$)] = 274.2467, (*m/z*) found: [($C_{17}H_{32}F_2$)] = 274.2466.

FT-IR ($\tilde{\nu}$ = cm^{-1}): 3660 (w), 2924 (s), 2854 (s), 1468 (w), 1390 (m), 1319 (w), 1287 (w), 1233 (m), 1138 (m), 1066 (m), 1058 (m), 966 (w), 931 (w), 802 (w), 772 (w), 720 (w).

2-(4-Chlorophenyl)-3-(2,2-difluoropropyl)quinoxaline (36)

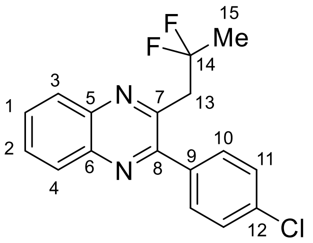

A solution containing 1-chloro-4-(4,4-difluoropent-1-yn-1-yl)benzene (**4**) (21.5 mg, 0.10 mmol, 1.0 eq.), $RuCl_3{\cdot}xH_2O$ (40~49% Ru) (0.5 mg, 0.001 mmol, 0.5 mg) and $PhI(OAc)_2$ (96.6 mg, 0.3 mmol, 3 eq.) in DCM and H_2O (4:1, 0.5 mL) was allowed to stir at room temperature for 3 h. The organics were extracted with DCM and the combined organic layers were dried over Na_2SO_4. The solvent was evaporated under reduced pressure and the crude diketone was directly used in next step without further purification.

Saccharin (0.92 mg, 0.005 mmol, 5 mol%) and 1,2-phenylendiamine (11.9 mg, 0.11 mmol, 1.1 eq.) were successively added to the crude diketone in methanol (0.5 mL). The mixture was stirred for 12 h. Solvent was removed under reduced pressure and the crude product was

purified by column chromatography (*n*-pentane:Et_2O 5:1) to yield the title compound as a white solid (18.6 mg, 0.06 mmol, 58%).

R_f = 0.31 (*n*-pentane:Et_2O 5:1).

^{1}H NMR (500 MHz, $CDCl_3$) δ 8.16 – 8.08 (m, 2H, H-C3~C4), 7.83 – 7.76 (m, 2H, H-C1~C2), 7.60 – 7.54 (m, 2H, H-C10), 7.54 – 7.49 (m, 2H, H-C11), 3.65 (t, $^3J_{HF}$ = 13.2 Hz, 2H, H-C13), 1.75 (t, $^3J_{HF}$ = 18.2 Hz, 3H, H-C15).

^{13}C NMR (126 MHz, $CDCl_3$) δ 154.7 (C8), 148.3 (C7), 141.4 (C5), 141.2 (C6), 137.4 (C12), 135.5 (C9), 130.8 (C10), 130.5 (C1/2), 130.4 (C1/2), 129.4 (C3/4), 129.1 (C3/4), 129.1 (C11), 123.5 (t, $^1J_{CF}$ = 240.9 Hz, C14), 43.4 (t, $^2J_{CF}$ = 27.1 Hz, C13), 24.0 (t, $^2J_{CF}$ = 26.5 Hz, C15).

^{19}F NMR (470 MHz, $CDCl_3$) δ -89.53 (qt,$^3J_{HF}$ = 18.9 Hz, $^3J_{HF}$ =14.3 Hz, 2F, F-C14).

^{19}F{^{1}H} NMR (470 MHz, $CDCl_3$) δ-89.53 (s, 2F, F-C14).

ESI-MS: (*m/z*) requires: [($C_{17}H_{13}N_2ClF_2Na)^+$] = 341.0628, (*m/z*) found: [($C_{17}H_{13}N_2ClF_2Na)^+$] = 341.0626.

FT-IR ($\tilde{\nu}$ = cm^{-1}): 2980 (w), 1611 (w), 1594 (w), 1544 (w), 1494 (w), 1479 (w), 1392 (m), 1359 (m), 1329 (w), 1287 (w), 1223 (m), 1185 (w), 1162 (w), 1122 (m), 1092 (s), 1058 (m), 1012 (m), 1003 (m), 963 (m), 946 (w), 931 (w), 921 (m), 907 (w), 878 (w), 871 (w), 847 (s), 825 (m), 801 (m), 789 (w), 763 (s), 735 (w), 726 (w), 699 (w), 669 (w), 636 (w), 616 (m), 607 (w).

Melting Point: 99-100 ˚C.

4,4-Difluorodec-1-yne (37)

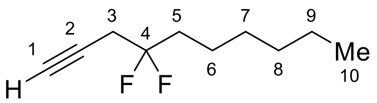

To a solution of (4,4-difluorodec-1-yn-1-yl)triisopropylsilane (**33**) (132.2 mg, 0.40 mmol, 1.0 eq.) in 1.0 mL of THF was added TBAF (0.8 mmol, 1.0 M in THF, 2.0 eq.). The solution was stirred at room temperature for 2 h. After workup, the crude mixture was analysed by ^{19}F NMR (85% NMR yield). Solvent was carefully removed under reduced pressure and the crude product was purified by column chromatography (*n*-pentane) to yield the title compound as a colorless volatile liquid (27.0 mg, 0.15 mmol, 39%).

R$_f$ = 0.37 (*n*-pentane).

^{1}H NMR (500 MHz, $CDCl_3$) δ 2.77 (td, $^3J_{HF}$ = 13.7 Hz, $^4J_{HH}$ = 2.7 Hz, 2H, H-C3), 2.10 (t, $^4J_{HH}$ = 2.7 Hz, 1H, H-C1), 2.04 – 1.92 (m, 2H, H-C5), 1.53 – 1.45 (m, 2H, H-C6), 1.37 – 1.27 (m, 6H, H-C7~C9), 0.90 (t, $^3J_{HH}$ = 6.9 Hz, 3H, H-C10).

^{13}C NMR (126 MHz, $CDCl_3$) δ 123.0 (t, $^1J_{CF}$ = 243.1 Hz, C4), 76.5 (t, $^3J_{CF}$ = 8.8 Hz, C2), 71.7 (C1), 35.4 (t, $^2J_{CF}$ = 24.1 Hz, C5), 31.7 (*C*H$_2$), 29.1 (*C*H$_2$), 28.0 (t, $^2J_{CF}$ = 32.3 Hz, C3), 22.6 (*C*H$_2$), 22.2 (t, $^3J_{CF}$ = 4.4 Hz, C6), 14.2 (C10).

^{19}F NMR (470 MHz, $CDCl_3$) δ -96.68 (tt, $^3J_{HF}$ = 16.6, 13.7 Hz, 2F, F-C4).

^{19}F{^{1}H} NMR (470 MHz, $CDCl_3$) δ -96.68 (s, 2F, F-C4).

GC-EI-MS: (*m/z*) requires: [($C_9H_{13}F_2$)] = 159.0985 [M-Me], (*m/z*) found: [($C_9H_{13}F_2$)] = 159.0983.

FT-IR ($\tilde{\nu}$ = cm^{-1}): 3676 (m), 2980 (s), 2901 (m), 2365 (m), 1406 (m), 1393 (m), 1230 (m), 1066 (s), 1027 (m), 880 (w), 772 (m), 669 (w).

***N*-(4-((4-(2,2-difluorooctyl)-1H-1,2,3-triazol-1-yl)sulfonyl)phenyl)acetamide (38)**

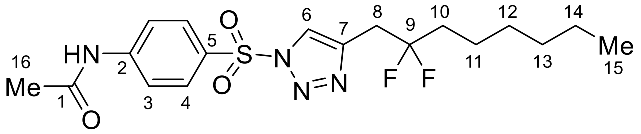

Copper thiophene-2-carboxylate (1.9 mg, 0.01 mmol, 10 mol%) was added to a stirred solution of 4,4-difluorodec-1-yne (**37**) (17.4 mg, 0.10 mmol, 1.0 eq.) in toluene (0.5 mL). The reaction mixture was cooled in an ice-water bath. Subsequently, 4-acetamidobenzenesulfonyl azide (28.8 mg, 0.12 mmol, 1.2 eq.) was added slowly. The reaction mixture was allowed to warm to room temperature and stir for 12 h. After removal of the solvent, the residue was directly purified by column chromatography (*n*-pentane:EtOAc 1:1) to yield the title compound as a white solid (40.8 mg, 0.10 mmol, 98%).

R$_f$ = 0.30 (*n*-pentane:EtOAc 1:1).

^{1}H NMR (500 MHz, $CDCl_3$) δ 8.33 (s, 1H, NH), 8.10 (s, 1H, H-C6), 8.00 – 7.93 (m, 2H, H-C4), 7.76 – 7.69 (m, 2H, H-C3), 3.31 (t, $^3J_{HF}$ = 16.0 Hz, 2H, H-C8), 2.22 (s, 3H, H-C16), 1.91

– 1.74 (m, 2H, H-C10), 1.54 – 1.44 (m, 2H, H-C11), 1.35 – 1.21 (m, 6H, H-C12~C14), 0.87 (t, $^{3}J_{HH}$ = 7.0 Hz, 3H, H-C15).

^{13}C NMR (126 MHz, $CDCl_3$) δ 169.4 (C1), 145.3 (C5), 140.3 (t, $^{3}J_{CF}$ = 4.5 Hz, C7), 130.4 (C4), 129.3 (C2), 123.2 (t, $^{1}J_{CF}$ = 242.2 Hz, C9), 123.1 (C6), 119.7 (C3), 36.3 (t, $^{2}J_{CF}$ = 24.3 Hz, C10), 33.6 (t, $^{2}J_{CF}$ = 28.1 Hz, C8), 31.6 (CH_2), 29.0 (CH_2), 24.9 (C16), 22.6 (CH_2), 22.2 (t, $^{3}J_{CF}$ = 4.4 Hz, C11), 14.1 (C15).

^{19}F NMR (470 MHz, $CDCl_3$) δ -97.20 (p, $^{3}J_{HF}$ = 16.4 Hz, 2F, F-C9).

^{19}F{^{1}H} NMR (470 MHz, $CDCl_3$) δ -97.20 (s, 2F, F-C9).

ESI-MS: (*m/z*) requires: $[(C_{18}H_{24}N_4O_3SF_2Na)^+]$ = 437.1429, (*m/z*) found: $[(C_{18}H_{24}N_4O_3SF_2Na)^+]$ = 437.1430.

FT-IR ($\tilde{\nu}$ = cm^{-1}): 3320 (w), 3115 (w), 2954 (w), 2929 (w), 2128 (w), 1702 (m), 1590 (m), 1528 (m), 1499 (w), 1468 (w), 1392 (m), 1370 (m), 1319 (m), 1261 (m), 1251 (m), 1192 (m), 1174 (s), 1089 (m), 1022 (s), 987 (m), 862 (w), 838 (m), 805 (m), 776 (m), 728 (m), 710 (m), 669 (w), 647 (m), 614 (m), 606 (s).

1-(4,4-Difluorodec-1-yn-1-yl)-4-methoxybenzene (39)

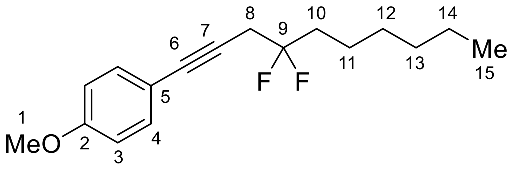

(4,4-Difluorodec-1-yn-1-yl)triisopropylsilane (**33**) (33.1 mg, 0.10 mmol, 1.0 eq.), $PdCl_2(PPh_3)_2$ (3.2 mg, 0.005 mmol, 4.5 mol%), and CuI (1.0 mg, 0.005 mmol, 5 mol%) were placed in a Schlenk flask. The flask was evacuated and refilled with Ar. THF (0.5 mL), NEt_3 (0.1 mL), TBAF (0.2 mL, 1 M in THF, 2 eq.) and *p*-iodoanisole (28.1 mg, 0.12 mmol, 1.2 eq.) were successively added into the flask. The reaction was stirred at 45 °C for 12 h. The solvent was removed on a rotary evaporator. Further purification by column chromatography (*n*-pentane:DCM 6:1) afforded the title compound as a colorless oil (17.9 mg, 0.06 mmol, 64%).

R_f = 0.17 (*n*-pentane:DCM 8:1).

^{1}H NMR (500 MHz, $CDCl_3$) δ 7.39 – 7.34 (m, 2H, H-C4), 6.86 – 6.80 (m, 2H, H-C3), 3.81 (s,

3H, H-C1), 2.97 (t, $^3J_{HF}$ = 13.7 Hz, 2H, H-C8), 2.11 – 1.96 (m, 2H, H-C10), 1.57 – 1.49 (m, 2H, H-C11), 1.41 – 1.28 (m, 6H, H-C12~C14), 0.90 (t, $^3J_{HH}$ = 6.9 Hz, 3H, H-C15).

^{13}C NMR (126 MHz, $CDCl_3$) δ 159.7 (C5), 133.3 (C4), 123.4 (t, $^1J_{CF}$ = 243.1 Hz, C9), 115.2 (C2), 114.0 (C3), 83.3 (C6), 80.3 (t, $^3J_{CF}$ = 9.1 Hz, C7), 55.4 (C1), 35.6 (t, $^2J_{CF}$ = 24.2 Hz, C10), 31.7 (CH_2), 29.1 (CH_2), 28.9 (t, $^2J_{CF}$ = 32.3 Hz, C8), 22.6 (CH_2), 22.3 (t, $^3J_{CF}$ = 4.5 Hz, C11), 14.2 (C15).

^{19}F NMR (470 MHz, $CDCl_3$) δ -96.34 (tt, $^3J_{HF}$ = 16.7, 13.7 Hz, 2F, F-C9).

^{19}F{^{1}H} NMR (470 MHz, $CDCl_3$) δ -96.34 (s, 2F, F-C9).

ESI-MS: (*m/z*) requires: [($C_{17}H_{22}OF_2Na)^+$] = 303.1531, (*m/z*) found: [($C_{17}H_{22}OF_2Na)^+$] = 303.1532.

FT-IR ($\tilde{\nu}$ = cm^{-1}): 2957 (w), 2932 (w), 1607 (m), 1570 (w), 1510 (s), 1465 (w), 1442 (w), 1382 (w), 1290 (m), 1246 (s), 1172 (m), 1148 (m), 1106 (w), 1075 (m), 1033 (s), 977 (w), 874 (w), 831 (s), 802 (m), 773 (w), 726 (w), 677 (w), 627 (w).

2-(4-(4,4-Difluoropent-1-yn-1-yl)phenyl)furan (40)

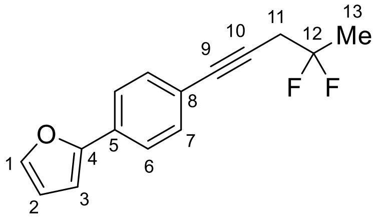

$Pd(PPh_3)_4$ (11.6 mg, 0.01 mmol, 10 mol%) and furan-2-ylboronic acid (28.0 mg, 0.25 mmol, 2.5 eq.) were placed in a Schlenk flask. The flask was evacuated and refilled with Ar. 1-bromo-4-(4,4-difluoropent-1-yn-1-yl)benzene (**6**) (25.9 mg, 0.10 mmol, 1.0 eq.), DME (0.5 mL) and 2 M aq. K_2CO_3 (125 μL, 0.25 mmol, 2.5 eq.) were successively added, and the reaction was stirred at 85 °C overnight. The resulting mixture was cooled to ambient temperature and diluted with H_2O. The organics were extracted with ethyl acetate and the combined organic layers were dried over Na_2SO_4. After removal of the solvent, the residue was purified by column chromatography (*n*-pentane:Et_2O 120:1-100:1) to yield the title compound as a pale yellow solid (22.8 mg, 0.09 mmol, 93%).

R_f = 0.30 (*n*-pentane:Et_2O 120:1).

^{1}H NMR (500 MHz, $CDCl_3$) δ 7.64 – 7.57 (m, 2H, H-C6), 7.48 (dd, $^3J_{HH}$ = 1.8, $^4J_{HH}$ = 0.7 Hz,

1H, H-C1), δ 7.46 – 7.41 (m, 2H, H-C7), 6.68 (dd, $^{3}J_{HH}$ = 1.8, $^{4}J_{HH}$ = 0.7 Hz, 1H, H-C3), 6.48 (dd, $^{3}J_{HH}$ = 3.4, 1.8 Hz, 1H, H-C2),3.03 (t, $^{3}J_{HF}$ = 13.2 Hz, 2H, H-C11), 1.80 (t, $^{3}J_{HF}$ = 18.2 Hz, 3H, H-C13).

^{13}C NMR (126 MHz, $CDCl_3$) δ 153.5 (C4), 142.6 (C1), 132.2 (C7), 130.8 (C5), 123.7 (C6), 122.3 (t, $^{1}J_{CF}$ = 241.9 Hz, C12), 121.6 (C8), 112.0 (C2), 106.1 (C3), 83.6 (C9), 82.6 (t, $^{3}J_{CF}$ = = 8.9 Hz, C10), 30.4 (t, $^{2}J_{CF}$ = 32.2 Hz, C11), 22.8 (t, $^{2}J_{CF}$ = 26.8 Hz, C13).

^{19}F NMR (470 MHz, $CDCl_3$) δ -89.49 (qt,$^{3}J_{HF}$ = 18.2 Hz, $^{3}J_{HF}$ =13.3 Hz, 2F, F-C12).

^{19}F{^{1}H} NMR (470 MHz, $CDCl_3$) δ-89.49 (s, 2F, F-C12).

GC-EI-MS: (*m/z*) requires: [($C_{15}H_{12}OF_2$)] = 246.0851, (*m/z*) found: [($C_{15}H_{12}OF_2$)] = 246.0851.

FT-IR ($\tilde{\nu}$ = cm^{-1}): 2989 (m), 2901 (m), 1515 (w), 1393 (s), 1339 (w), 1280 (m), 1258 (m), 1235 (s), 1220 (m), 1175 (w), 1145 (s), 1116 (s), 1079 (s), 1066 (s), 1057 (s), 1027 (m), 1019 (m), 1009 (s), 964 (m), 936 (m), 904 (m), 878 (m), 842 (s), 805 (m), 783 (w), 772 (m), 738 (s), 664 (w).

Melting Point: 57-59 ˚C.

1.4.2 Synthesis of O´Hagan´s CF_2-modified palmitic acid

Methyl 7-methylenepentadec-5-ynoate (41)

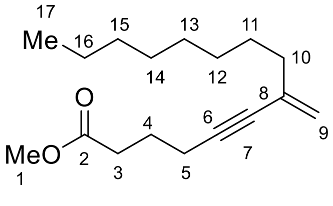

Compound **41** was prepared according to General Procedure **A** using methyl hex-5-ynoate (227.1 mg, 1.80 mmol, 1.0 eq.). The crude residue was purified by column chromatography (*n*-pentane:Et_2O 15:1) to yield the title compound as a colorless oil (345.8 mg, 1.31 mmol, 73%).

R*f* = 0.50 (*n*-pentane:Et_2O 15:1).

^{1}H NMR (599 MHz, $CDCl_3$) δ 5.22 – 5.20 (m, 1H, H^a-C9), 5.14 – 5.12 (m, 1H, H^b-C9), 3.68 (s, 3H, H-C1), 2.45 (t, $^{3}J_{HH}$ = 7.5 Hz, 2H, H-C3), 2.38 (t, $^{3}J_{HH}$ = 6.9 Hz, 2H, H-C5), 2.10 (t, $^{3}J_{HH}$ = 7.6 Hz, 2H, H-C10), 1.86 (p, $^{3}J_{HH}$ = 7.0 Hz, 2H, H-C4), 1.53 – 1.45 (m, 2H, H-C11),

1.33 – 1.22 (m, 10H, H-C12~C16), 0.88 (t, $^3J_{HH}$ = 7.0 Hz, 3H, H-C17).

^{13}C NMR (151 MHz, $CDCl_3$) δ 173.8 (C2), 132.3 (C8), 119.9 (C9), 88.5 (C6), 82.1 (C7), 51.7 (C1), 37.6 (C10), 33.0 (C3), 32.0 (CH_2), 29.6 (CH_2), 29.4 (CH_2), 29.1 (CH_2), 28.3 (C11), 24.1 (C4), 22.8 (CH_2), 18.9 (C5), 14.3 (C17).

ESI-MS: (*m/z*) requires: [$(C_{17}H_{28}O_2Na)^+$] = 287.1982, (*m/z*) found: [$(C_{17}H_{28}O_2Na)^+$] = 287.1982.

FT-IR ($\tilde{\nu}$ = cm^{-1}): 2926 (m), 2855 (w), 1741 (s), 1610 (w), 1456 (w), 1436 (w), 1369 (w), 1314 (w), 1246 (w), 1214 (w), 1161 (m), 893 (w).

Methyl 8,8-difluorohexadec-5-ynoate (42)

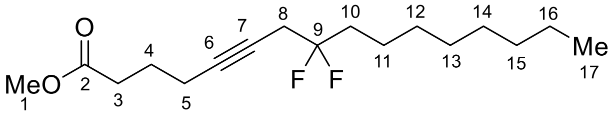

Compound **42** was prepared according to the General Procedure **E** with an amine:HF ratio of 1:5.5 and a concentration of 0.1 M using methyl 7-methylenepentadec-5-ynoate (**41**) (26.4 mg, 0.1 mmol, 1.0 eq.). The crude product was purified by column chromatography (*n*-pentane:Et_2O 15:1) to yield the title compound as a colorless oil (15.3 mg, 0.05 mmol, 51%).

R*f* = 0.35 (*n*-pentane:Et_2O 15:1).

^{1}H NMR (400 MHz, $CDCl_3$) δ 3.68 (s, 3H, H-C1), 2.72 (tt, $^3J_{HF}$ = 13.9 Hz, $^5J_{HH}$ = 2.4 Hz, 2H, H-C8), 2.43 (t, $^3J_{HH}$ = 7.4 Hz, 2H, H-C3), 2.25 (tt, $^3J_{HH}$ = 6.9 Hz, $^5J_{HH}$ = 2.4 Hz, 2H, H-C5), 2.02 – 1.87 (m, 2H, H-C10), 1.82 (p, J = 7.2 Hz, 2H, H-C4), 1.53 – 1.42 (m, 2H, H-C11), 1.39 – 1.22 (m, 10H, H-C12~C16), 0.92 – 0.84 (m, 3H, H-C17).

^{13}C NMR (126 MHz, $CDCl_3$) δ 173.7 (C2), 123.5 (t, $^1J_{CF}$ = 242.7 Hz, C9), 82.4 (C6), 73.2 (t, $^3J_{CF}$ = 8.9 Hz, C7), 51.7 (C1), 35.5 (t, $^2J_{CF}$ = 24.3 Hz, C10), 32.9 (C3), 32.0 (CH_2), 29.5 (CH_2), 29.5 (CH_2), 29.3 (CH_2), 28.2 (t, $^2J_{CF}$ = 31.9 Hz, C8), 24.0 (C4), 22.8 (CH_2), 22.3 (C11), 18.3 (C5), 14.2 (C17).

^{19}F NMR (470 MHz, $CDCl_3$) δ -96.87 (tt, $^3J_{HF}$ = 16.6, 13.9 Hz, 2F, F-C9).

^{19}F{^{1}H} NMR (470 MHz, $CDCl_3$) δ -96.87 (s, 2F, F-C9).

GC-EI-MS: (*m/z*) requires: [($C_{17}H_{27}O_2F_2$)] = 302.2008 [M-H], (*m/z*) found: [($C_{17}H_{27}O_2F_2$)] = 302.2003.

FT-IR ($\tilde{\nu}$ = cm^{-1}): 2954 (m), 2927 (s), 2857 (w), 1739 (s), 1458 (w), 1436 (m), 1314 (w), 1217 (m), 1159 (s), 1058 (m), 999 (m), 884 (m), 723 (w), 633 (w).

8,8-Difluorohexadecanoic acid (44)

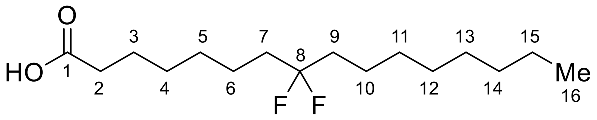

Methyl 8,8-difluorohexadec-5-ynoate (**42**) (30.2 mg, 0.10 mmol, 1.0 eq.) and palladium, on carbon (10% w/w, 10.6 mg, 0.01 mmol, 10 mol%) were placed in a Schlenk flask. The flask was evacuated and refilled with hydrogen through a balloon. After addition of 1.0 mL of methanol, the mixture was stirred at room temperature for 24 h under balloon pressure of hydrogen. The reaction mixture was filtered through celite and washed with Et_2O. After removal of solvent, the crude methyl 8,8-difluorohexadecanoate (**43**) was used in the next step without further purification.

The crude methyl 8,8-difluorohexadecanoate (**43**) was dissolved 1.0 mL H_2O and 2.0 mL THF. Then LiOH (14.4 mg, 0.6 mmol, 6.0 eq.) was added to the mixture. The reaction was stirred at room temperature for 12 h, and then the mixture was concentrated. Water (2 mL) was added to the flask containing the residue, and 1 M hydrochloric acid was used to acidify the solution. The aqueous phase was extracted with DCM (3×10 mL). The organic layer was dried over Na_2SO_4, filtered, and concentrated. The residue was purified by column chromatography (*n*-pentane:EtOAc 5:1) to afford the title compound as a white solid (28.7 mg, 0.10 mmol, 98%).

R*f* = 0.40 (*n*-pentane:EtOAc 5:1).

^{1}H NMR (599 MHz, $CDCl_3$) δ 2.36 (t, $^3J_{HH}$ = 7.5 Hz, 2H, H-C2), 1.87 – 1.70 (m, 4H, H-C7, H-C9), 1.65 (p, $^3J_{HH}$ = 7.4 Hz, 2H, H-C3), 1.52 – 1.40 (m, 4H, H-C6, H-C10), 1.38 – 1.25 (m, 14H, H-C4~C5, H-C11~C15), 0.88 (t, $^3J_{HH}$ = 7.0 Hz, 3H, H-C16).

^{13}C NMR (151 MHz, $CDCl_3$) δ 180.1 (C1), 125.5 (t, $^1J_{CF}$ = 240.0 Hz, C8), 36.5 (t, $^2J_{CF}$ = 25.5 Hz, *C*H_2), 36.4 (t, $^2J_{CF}$ = 25.5 Hz, *C*H_2), 34.1 (C2), 32.0 (*C*H_2), 29.6 (*C*H_2), 29.5 (*C*H_2), 29.3 (*C*H_2), 29.2 (*C*H_2), 29.0 (*C*H_2), 24.6 (C3), 22.8 (*C*H_2), 22.5 (t, $^3J_{CF}$ = 4.6 Hz, *C*H_2), 22.3 (t, $^3J_{CF}$

= 4.6 Hz, *C*H_2), 14.2 (C16).

^{19}F NMR (564 MHz, $CDCl_3$) δ -97.78 (p, $^3J_{HF}$ = 16.6 Hz, 2F, F-C8).

^{19}F{^{1}H} NMR (564 MHz, $CDCl_3$) δ -97.78 (s, 2F, F-C8).

ESI-MS: (*m/z*) requires: [$(C_{16}H_{29}O_2F_2Na_2)^+$] = 337.1926, (*m/z*) found: [$(C_{16}H_{29}O_2F_2Na_2)^+$] = 337.1923.

Analytical data is in agreement with literature values.[15]

1.5 Control experiments

1-(But-3-en-1-yn-1-yl)-4-nitrobenzene (45)

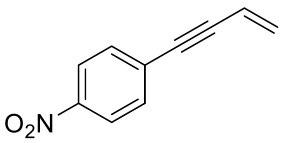

Compound **45** was prepared according to General Procedure **B** using (4-nitrophenyl)acetylene (735.7 mg, 5.00 mmol, 1.0 eq.). The crude residue was purified by column chromatography (*n*-pentane) to yield the title compound as a yellow solid (310.0 mg, 1.79 mmol, 36%).

R*f* = 0.65 (*n*-pentane:Et_2O 20:1).

^{1}H NMR (400 MHz, $CDCl_3$) δ 8.21 – 8.17 (m, 2H), 7.60 – 7.55 (m, 2H), 6.04 (dd, *J* = 17.5, 11.2 Hz, 1H), 5.84 (dd, *J* = 17.6, 1.9 Hz, 1H), 5.67 (dd, *J* = 11.1, 2.0 Hz, 1H).

^{13}C NMR (101 MHz, $CDCl_3$) δ 147.17, 132.39 (*overlapping*), 130.22, 129.19, 123.72 (*overlapping*), 116.61, 93.30, 88.12.

GC-EI-MS: (*m/z*) requires: [$(C_{10}H_7NO_2)$] = 173.0471, (*m/z*) found: [$(C_{10}H_7NO_2)$] = 173.0471.

Analytical data is in agreement with literature values.[16]

1-(4,4-Difluorobut-1-yn-1-yl)-4-nitrobenzene (46)

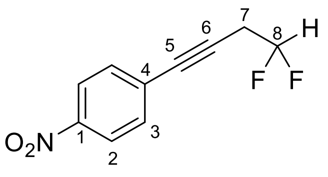

Compound **46** was prepared according to the General Procedure **E** with an amine:HF ratio of 1:8.5 using 1-(but-3-en-1-yn-1-yl)-4-nitrobenzene (**45**) (34.6 mg, 0.2 mmol, 1.0 eq.). The crude product was purified by column chromatography (*n*-pentane:Et_2O 15:1) to

yield the title compound as a colorless oil (25.3 mg, 0.12 mmol, 60%).

R$_f$ = 0.35 (*n*-pentane:Et_2O 15:1).

^{1}H NMR (500 MHz, $CDCl_3$) δ 8.29 – 8.00 (m, 2H, H-C2), 7.67 – 7.43 (m, 2H, H-C3), 5.99 (tt, $^2J_{HF}$ = 56.0 Hz, $^3J_{HH}$ = 4.4 Hz, 1H, H-C8), 3.05 (td, $^3J_{HF}$ = 15.3 Hz, $^3J_{HH}$ = 4.4 Hz, 2H, H-C7).

^{13}C NMR (126 MHz, $CDCl_3$) δ 147.4 (C1), 132.7 (C3), 129.6 (C4), 123.7 (C2), 113.8 (t, $^1J_{CF}$ = 243.1 Hz, C8), 85.8 (t, $^3J_{CF}$ = 9.5 Hz, C6), 82.3 (C5), 26.7 (t, $^2J_{CF}$ = 26.7 Hz, C7).

^{19}F NMR (470 MHz, $CDCl_3$) δ -114.88 (dt,$^2J_{HF}$ = 56.0 Hz, $^3J_{HF}$ =15.3 Hz, 2F, F-C8).

^{19}F{^{1}H} NMR (470 MHz, $CDCl_3$) δ-114.88 (s, 2F, F-C8).

GC-EI-MS: (*m/z*) requires: [($C_{10}H_7NO_2F_2$)] = 211.0439, (*m/z*) found: [($C_{10}H_7NO_2F_2$)] = 211.0437.

FT-IR ($\tilde{\nu}$ = cm^{-1}): 2989 (m), 1594 (m), 1518 (s), 1492 (w), 1395 (m), 1343 (s), 1309 (w), 1286 (w), 1225 (w), 1175 (w), 1109 (s), 855 (s), 768 (w), 750 (s), 710 (w), 689 (m).

Heptadec-1-en-3-yne (47)

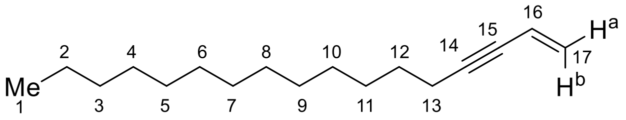

Compound **47** was prepared according to General Procedure **B** using pentadec-1-yne (1.26 g, 6.06 mmol, 1.0 eq.). The crude residue was purified by column chromatography (*n*-pentane) to yield the title compound as a colorless oil (529.0 mg, 2.26 mmol, 37%).

R$_f$ = 0.85 (*n*-pentane).

^{1}H NMR (500 MHz, $CDCl_3$) δ 5.78 (ddt, $^3J_{HH}$ = 17.5, $^3J_{HH}$ = 11.1, $^4J_{HH}$ = 2.2 Hz, 1H, H-C16), 5.54 (dd, $^3J_{HH}$ = 17.5, $^2J_{HH}$ = 2.2 Hz, 1H, H^b-C17), 5.37 (dd, $^3J_{HH}$ = 11.1, $^2J_{HH}$ = 2.2 Hz, 1H, H^a-C17), 2.29 (td, $^3J_{HH}$ = 7.2, $^4J_{HH}$ = 2.2 Hz, 2H, H-C13), 1.53 (p, $^3J_{HH}$ = 7.0 Hz, 2H, H-C12), 1.42 – 1.34 (m, 2H, H-C11), 1.32 – 1.25 (m, 18H, H-C2~C10), 0.88 (t, $^3J_{HH}$ = 6.9 Hz, 3H, H-C1).

^{13}C NMR (126 MHz, $CDCl_3$) δ 125.5 (C17), 117.8 (C16), 91.4 (C14), 79.4 (C15), 32.1 (CH_2),

29.8 (CH_2), 29.8 (CH_2), 29.8 (CH_2), 29.8 (CH_2), 29.7 (CH_2), 29.5 (CH_2), 29.3 (CH_2), 29.1 (C11), 28.8 (C12), 22.9 (CH_2), 19.5 (C13), 14.3 (C1).

Analysis by mass spectrometry was inconclusive.

FT-IR ($\tilde{\nu}$ = cm^{-1}): 2956 (m), 2921 (s), 2853 (s), 1609 (w), 1466 (w), 1411 (w), 1393 (w), 1378 (w), 1066 (w), 971 (w), 911 (m), 722 (w).

1,1-Difluoroheptadec-3-yne (48)

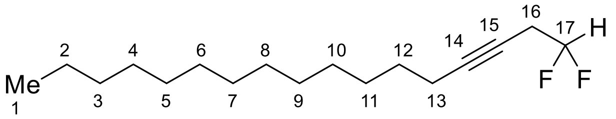

Compound **48** was prepared according to the General Procedure **E** with an amine:HF ratio of 1:6.0 using heptadec-1-en-3-yne (**47**) (46.9 mg, 0.2 mmol, 1.0 eq.). The crude product was purified by column chromatography (*n*-pentane) to yield the title compound as a colorless oil (29.8 mg, 0.11 mmol, 55%).

$\mathbf{R_f}$ = 0.69 (*n*-pentane).

^{1}H NMR (500 MHz, $CDCl_3$) δ 5.83 (tt, $^2J_{HF}$ =56.5 Hz, $^3J_{HH}$ = 4.6 Hz, 1H, H-C17), 2.78-2.66 (m, 2H, H-C16), 2.22 – 2.08 (m, 2H, H-C13), 1.53-1.45 (m, 2H, H-C12), 1.40-1.32 (m, 2H, H-C11), 1.30-1.22 (m, 18H, H-C2~C10), 0.88 (t, $^3J_{HH}$ = 4.3 Hz, 3H, H-C1).

^{13}C NMR (126 MHz, $CDCl_3$) δ 114.8 (t, $^1J_{CF}$ = 242.2 Hz, C17), 84.4 (t, $^4J_{CF}$ = 1.1 Hz, C14), 70.6 (t, $^3J_{CF}$ = 9.9 Hz, C15), 32.1 (C13), 29.8 (C12), 29.8 (C11), 29.8 (C10), 29.8 (C9), 29.7 (C8), 29.5 (C7), 29.3 (C6), 29.0 (C5), 28.8 (C4), 26.0 (t, $^2J_{CF}$ = 26.4 Hz, C16), 22.8 (C3), 18.8 (C2), 14.3 (C1).

^{19}F NMR (470 MHz, $CDCl_3$) δ -115.19 (qt,$^2J_{HF}$ = 56.5 Hz, $^3J_{HF}$ =15.5 Hz, 2F, F-C13).

^{19}F{^{1}H} NMR (470 MHz, $CDCl_3$) δ -115.19 (s, 2F, F-C17).

ESI-MS: (*m/z*) requires: [$(C_{17}H_{30}F_2Ag)^+$] = 379.1361, (*m/z*) found: [$(C_{17}H_{30}F_2Ag)^+$] = 379.1367.

FT-IR ($\tilde{\nu}$ = cm^{-1}): 2923 (s), 2854 (m), 1466 (m), 1393 (m), 1227 (w), 1116 (s), 1059 (s), 864 (m), 722 (w).

1-(Dec-1-en-3-yn-2-yl)-4-(trifluoromethyl)benzene (49)

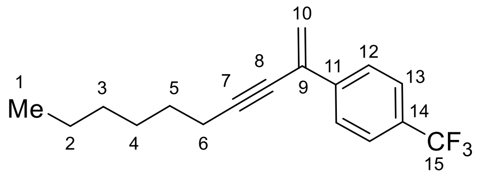

Under an argon atmosphere, a flask was charged with 1-octyne (1.21 g, 11.00 mmol, 1.1 eq.) and 20 mL of dry THF. The solution was cooled to -78 °C and *n*BuLi (1.6 M in *n*-hexane, 7.5 mL, 12.00 mmol, 1.2 eq.) was added. The resulting solution was stirred for 30 minutes at room temperature and then cooled to -78 °C again. 1-(4-trifluoromethylphenyl)ethanone (1.88 g, 10.00 mmol, 1.0 eq.) was added dropwise. The reaction mixture was allowed to warm to room temperature and monitored by TLC for completion. On completion the reaction was quenched with saturated aqueous NH_4Cl. The aqueous layer was extracted with ethyl acetate and the combined organic layers were washed with brine, dried over Na_2SO_4 and filtered. Then, the solution was concentrated under reduced pressure to afford the crude 2-(4-(trifluoromethyl)phenyl)dec-3-yn-2-ol.

The resulting crude 2-(4-(trifluoromethyl)phenyl)dec-3-yn-2-ol (1.23 g, 4.14 mmol, 1.0 eq.) was dissolved in dry DCM (10 mL), and the mixture was cooled to 0 °C with a cooling bath. To this solution was added triethylamine (2.09 g, 20.69 mmol, 5.0 eq.) and methylsulfonyl chloride (1.18 g, 10.34 mmol, 2.5 eq.) sequentially. The reaction was monitored by TLC for completion. On completion the reaction was quenched with saturated aqueous NH_4Cl. The aqueous layer was extracted with ethyl acetate and the combined organic layers were washed with brine, dried over Na_2SO_4 and filtered, and concentrated under reduced pressure. The crude product was purified by column chromatography (*n*-pentane) to yield the title compound as a colorless oil (658.7 mg, 2.35 mmol, 57%).

R_f = 0.72 (*n*-pentane).

^{1}H NMR (500 MHz, $CDCl_3$) δ 7.77 – 7.73 (m, 2H, H-C12), 7.62 – 7.58 (m, 2H, H-C13), 5.91 (s, 1H, H^a-C10), 5.68 (s, 1H, H^b-C10), 2.42 (t, $^3J_{HH}$ = 7.1 Hz, 2H, H-C6), 1.66 – 1.57 (m, 2H, H-C5), 1.50 – 1.42 (m, 2H, H-C4), 1.37 – 1.30 (m, 4H, H-C2, H-C3), 0.94 – 0.89 (m, 3H, H-C1).

^{13}C NMR (126 MHz, $CDCl_3$) δ 141.5 (app. q, $^5J_{CF}$ = 1.4 Hz, C17), 130.2 (C9), 130.2 (app. q, $^2J_{CF}$ = 32.4 Hz, C14), 126.5 (C12), 125.4 (q, $^3J_{CF}$ = 3.8 Hz, C13), 124.3 (app. q, $^1J_{CF}$ = 272.2 Hz, C15), 121.4 (C10), 93.1 (C7), 79.2 (C8), 31.5 (CH_2), 28.8 (CH_2), 28.8 (CH_2), 22.7 (CH_2),

19.5 (C6), 14.2 (C1).

^{19}F NMR (470 MHz, $CDCl_3$) δ -62.61 (s, 3F, F-C15).

^{19}F{^{1}H} NMR (470 MHz, $CDCl_3$) δ -62.61 (s, 3F, F-C15).

GC-EI-MS: (*m/z*) requires: [($C_{17}H_{19}F_3$)] = 280.1433, (*m/z*) found: [($C_{17}H_{19}F_3$)] = 280.1432.

FT-IR ($\tilde{\nu}$ = cm^{-1}): 2957 (w), 2932 (w), 2861 (w), 1718 (w), 1619 (w), 1412 (w), 1323 (s), 1165 (m), 1125 (s), 1111 (s), 1068 (s), 1017 (m), 904 (w), 841 (m), 766 (w), 725 (w), 692 (w), 607 (m).

1-(1,1-Difluorodec-3-yn-1-yl)-4-(trifluoromethyl)benzene (50)

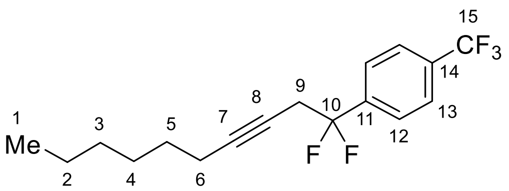

Compound **50** was prepared according to the General Procedure **E** with an amine:HF ratio of 1:5.5 using 1-(dec-1-en-3-yn-2-yl)-4-(trifluoromethyl)benzene (**49**) (56.1 mg, 0.2 mmol, 1.0 eq.). After workup, the crude mixture was analysed by ^{19}F NMR (42% homopropargylic fluoride and <5% propargylic fluoride). Purification by column chromatography (*n*-pentane) yielded the title compound as a colorless volatile liquid (25.5 mg, 0.08 mmol, 40%).

R*f* = 0.49 (*n*-pentane).

^{1}H NMR (400 MHz, $CDCl_3$) δ 7.74 – 7.62 (m, 4H, H-C12, H-C13), 3.05 (tt, $^{3}J_{HF}$ = 13.5 Hz, $^{5}J_{HH}$ = 2.5 Hz, 2H, H-C9), 2.10 (tt, $^{3}J_{HH}$ = 7.0 Hz, $^{5}J_{HH}$ = 2.4 Hz, 2H, H-C6), 1.40 (p, $^{3}J_{HH}$ = 7.5 Hz, 2H, H-C5), 1.33 – 1.19 (m, 6H, H-C2~C4), 0.88 (t, $^{3}J_{HH}$ = 7.0 Hz, 3H, H-C1).

^{13}C NMR (126 MHz, $CDCl_3$) δ 139.8 (t, $^{2}J_{CF}$ = 26.7 Hz, C11), 132.3 (app. q, $^{2}J_{CF}$ = 33.0 Hz, C14), 126.2 (t, $^{3}J_{CF}$ = 6.1 Hz, C12), 125.4 (q, $^{3}J_{CF}$ = 3.8 Hz, C13), 123.9 (app. q, $^{1}J_{CF}$ = 272.3 Hz, C15), 120.1 (t, $^{1}J_{CF}$ = 245.0 Hz, C10), 85.2 (C7), 70.9 (t, $^{3}J_{CF}$ = 8.4 Hz, C8), 31.4 (CH_2), 31.0 (t, $^{2}J_{CF}$ = 33.3 Hz, C9), 28.6 (CH_2), 28.5 (CH_2), 22.7 (CH_2), 18.7 (C6), 14.1 (C1).

^{19}F NMR (470 MHz, $CDCl_3$) δ -62.94 (s, 3F, F-C15), -95.06 (t, $^{3}J_{HF}$ = 13.5 Hz, 2F, F-C10).

^{19}F{^{1}H} NMR (377 MHz, $CDCl_3$) δ -62.94 (s, 3F, F-C15), -95.06 (s, 2F, F-C10).

GC-EI-MS: (*m/z*) requires: [($C_{17}H_{19}F_5$)] = 318.1401, (*m/z*) found: [($C_{17}H_{19}F_5$)] = 318.1393.

FT-IR ($\tilde{\nu}$ = cm^{-1}): 2960 (w), 2932 (w), 2861 (w), 1413 (w), 1324 (s), 1312 (m), 1220 (w), 1168 (m), 1131 (s), 1068 (s), 1014 (m), 996 (w), 891 (m), 842 (s), 772 (s), 753 (w), 719 (m), 640 (m), 607 (w).

(Methyl-d3)triphenylphosphonium bromide (Int 3)

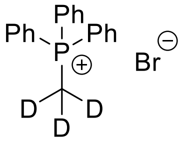

To a flask with a magnetic stir bar was added Ph_3PMeBr (1.61 g, 4.50 mmol, 1.0 eq.), D_2O (6 mL), and NaOH (90mg, 2.25 mmol, 0.5 eq.). The reaction mixture was stirred under Ar atmosphere, and after 24 h, DCM (15 mL) was added to the solution and organic layer was collected and dried with Na_2SO_4. The addition of pentane (30 mL) to the resulting solution and storage at 0 °C for 24 h afforded colorless crystals (1.26 g, 3.50 mmol, 78%)

^{1}H NMR (400 MHz, $CDCl_3$) δ 7.83 – 7.64 (m, 15H).

^{2}H NMR (77 MHz, $CHCl_3$) δ 3.21 (s, 3H).

ESI-MS: (*m/z*) requires: [($C_{19}H_{15}PD_3$)$^+$] = 280.1329, (*m/z*) found: [($C_{19}H_{15}PD_3$)$^+$] = 280.1329.

Analytical data is in agreement with literature values.[17]

2-Methyl-3-methyleneoctadec-4-yne (S29-d)

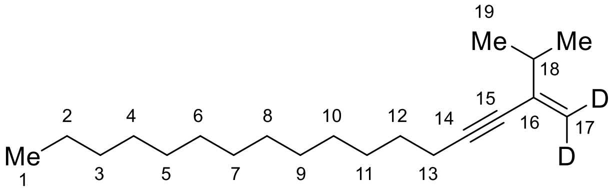

Compound **S29-d** was prepared according to General Procedure **D** using pentadec-1-yne (232.2 mg, 1.11 mmol, 1.0 eq.). The crude residue was purified by column chromatography (*n*-pentane) to yield the title compound as a colorless oil (175.7 mg, 0.63 mmol, 57%, 77% D).

R*f* = 0.89 (*n*-pentane).

^{1}H NMR (500 MHz, $CDCl_3$) δ 5.42 – 4.93 (m, 0.46 H, H-C17), 2.42 – 2.34 (m, 1H, H-C18),

2.31 (t, $^3J_{HH}$ = 7.1 Hz, 2H, H-C13), 1.56 – 1.51 (m, 2H, H-C12), 1.45 – 1.36 (m, 2H, H-C11), 1.32 – 1.24 (m, 18H, H-C2~C10), 1.08 (d, $^3J_{HH}$ = 6.8 Hz, 6H, H-C19), 0.88 (t, $^3J_{HH}$ = 7.0 Hz, 3H, H-C1).

^{2}H NMR (77 MHz, $CHCl_3$) δ 5.18 (s, 1.54 ^{2}H, ^{2}H-C17).

3,3-Difluoro-2-methylnonadec-5-yne (29-d)

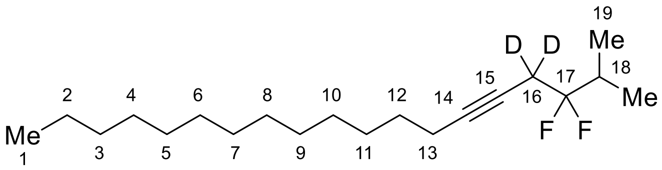

Compound **29-d** was prepared according to the General Procedure **E** with an amine:HF ratio of 1:6.0 using 2-methyl-3-methyleneoctadec-4-yne (**S29-d**) (55.7 mg, 0.2 mmol, 1.0 eq.). The crude product was purified by column chromatography (*n*-pentane) to yield the title compound as a colorless oil (35.2 mg, 0.11 mmol, 56%, 76% D).

R$_f$ = 0.71 (*n*-pentane).

^{1}H NMR (400 MHz, $CDCl_3$) δ 2.79 – 2.68 (m, 0.49 H, H-C16), 2.44 – 2.22 (m, 1H, H-C18), 2.16 (tt, $^3J_{HH}$ = 7.1 Hz, $^5J_{HH}$ = 2.5 Hz, 2H, H-C13), 1.48 (p, J = 7.2 Hz, 2H, H-C12), 1.39 – 1.22 (m, 20H, H-C2~C11), 1.04 (d, $^3J_{HH}$ = 6.9 Hz, 6H, H-C19), 1.04 (t, $^3J_{HH}$ = 7.0 Hz, 3H, H-C1).

^{2}H NMR (77 MHz, $CHCl_3$) δ 2.69 (s, 1.51 ^{2}H, ^{2}H-C16).

1.6 X-ray crystallographic data

X-Ray diffraction: Data sets for compound **2** were collected with a Bruker D8 Venture Photon III Diffractometer. Programs used: data collection: *APEX4* Version 2021.4-0[18] (Bruker AXS Inc., **2021**); cell refinement: *SAINT* Version 8.40B (Bruker AXS Inc., **2021**); data reduction: *SAINT* Version 8.40B (Bruker AXS Inc., **2021**); absorption correction, *SADABS* Version 2016/2 (Bruker AXS Inc., **2021**); structure solution *SHELXT*-Version 2018-3[19] (Sheldrick, G. M. *Acta Cryst.,* **2015**, *A71*, 3-8); structure refinement *SHELXL*- Version 2018-3[20] (Sheldrick, G. M. *Acta Cryst.,* **2015**, *C71* (1), 3-8) and graphics, *XP*[21] (Version 5.1, Bruker AXS Inc., Madison, Wisconsin, USA, **1998**). *R*-values are given for observed reflections, and wR^2 values are given

for all reflections.

X-ray crystal structure analysis of 2 (gil10435): A colorless, plate-like specimen of $C_{12}H_9F_2N$, approximate dimensions 0.082 mm x 0.084 mm x 0.211 mm, was used for the X-ray crystallographic analysis. The X-ray intensity data were measured on a single crystal diffractometer Bruker D8 Venture Photon III system equipped with a micro focus tube Mo ImS (MoKα, λ = 0.71073 Å) and a MX mirror monochromator. A total of 567 frames were collected. The total exposure time was 4.72 hours. The frames were integrated with the Bruker SAINT software package using a narrow-frame algorithm. The integration of the data using a monoclinic unit cell yielded a total of 10539 reflections to a maximum θ angle of 26.78° (0.79 Å resolution), of which 1126 were independent (average redundancy 9.360, completeness = 99.4%, R_{int} = 3.63%, R_{sig} = 1.69%) and 963 (85.52%) were greater than $2\sigma(F^2)$. The final cell constants of a = 4.6655(2) Å, b = 8.1691(4) Å, c = 13.1868(6) Å, β = 98.453(2)°, volume = 497.13(4) $Å^3$, are based upon the refinement of the XYZ-centroids of 4653 reflections above 20 σ(I) with 5.884° < 2θ < 53.55°. Data were corrected for absorption effects using the Multi-Scan method (SADABS). The ratio of minimum to maximum apparent transmission was 0.938. The calculated minimum and maximum transmission coefficients (based on crystal size) are 0.9780 and 0.9910. The structure was solved and refined using the Bruker SHELXTL Software Package, using the space group $P2_1/m$, with Z = 2 for the formula unit, $C_{12}H_9F_2N$. The final anisotropic full-matrix least-squares refinement on F^2 with 90 variables converged at R1 = 3.48%, for the observed data and wR2 = 9.36% for all data. The goodness-of-fit was 1.049. The largest peak in the final difference electron density synthesis was 0.335 $e^-/Å^3$ and the largest hole was -0.268 $e^-/Å^3$ with an RMS deviation of 0.043 $e^-/Å^3$. On the basis of the final model, the calculated density was 1.371 g/cm^3 and F(000), 212 e^-. The hydrogens at C1 and C3 atoms were refined freely but with C-H distance restraints (U-fixed value). CCDC Nr.: 2256836.

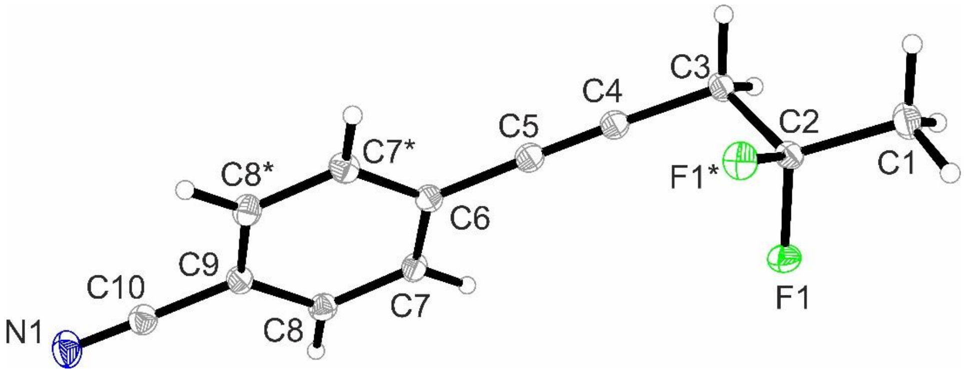

Supplementary Figure 1. Crystal structure of compound **2**. Thermal ellipsoids are shown at 50% probability.

1.7 DFT calculations

Methods

All computations were performed with TURBOMOLE 7.6.[22] The structures were optimized without geometrical constraints using the TPSS meta-GGA functional[23] and an atom-pairwise dispersion correction (D3).[24] A flexible triple zeta basis set (def2-TZVP)[25] was used in all calculations. For the calculation of free energy contributions of translation, rotations and harmonic vibrations (G^{RRHO}_{298}), a rotor approximation was applied for vibrational modes with wave numbers below 100 cm^{-1}.[26] Single point energy DFT calculations were performed with Turbomole using the hybrid functional PW6B95(-D3).[27] Free energies of solvation (G^{solv}_{298}) were obtained with the COSMO-RS model[28] for 298 K using chloroform ($CHCl_3$) as solvent.

The total relative Gibbs free energy ($\Delta G(298)_{solv}$) is calculated with respect to the reference compounds (isolated **S1**, pyridine and $PhIF_2$) as

$$\Delta G(298)_{solv} = \Delta E(\text{PW6B95-D3//TPSS-D3/def2-TZVP}) + \Delta G^{RRHO}_{298} + \Delta G^{solv}_{298} \qquad (1)$$

For each intermediate, the energies of species not depicted explicitely were included in the calculation (Pyridine for **INT1-3b**; Pyridine and $[PhIF]^-$ for **INT2cat/INT3cat**; $[PhIF]^-$ for **INT1py-INT3bpy**). All energies are reported in Supplementary Table 1, DFT-optimized structures of all species are presented in Supplementary Figure 2.

Results

We have investigated possible intermediates of the 1,1-difluorination of enyne **S1** with $PhIF_2$ in the presence of pyridine. An energy diagram summarizing relative free energies is presented in Supplementary Figure 2.

It turns out that primary cation **INT1cat** forms a cyclic cation-π complex **INT3cat** which is more stable than the rearranged, fluorosubstituted carbenium ion **INT2cat**. Given the large rise in ΔG for the formation of dissociated ions, we probed the interaction of the carbocation with $PhIF^-$ or pyridine. It turned out that the latter is significantly more favourable, leading to an exothermic formation of intermediate pyridinium ions.

The initially formed **INT1py** converts exothermically to **INT2py**, possibly through methylene cyclopropanes **INT3apy**/**INT3bpy** as intermediates. Nucleophilic attack of fluoride replacing pyridine leads to the formation of the observed product **1**.

Supplementary Table 1. Energies of molecular species in the reactions of **S1**, calculated with DFT[a].

	E_{el} (TPSS-D3/def2-TZVP) [E_h]	G^{RRHO}_{298} (TPSS-D3) [kcal/mol]	E_{el} (PW6B95-D3/def2-TZVP) [E_h]	G^{solv}_{298}, $CHCl_3$ (COSMO-RS) [kcal/mol]	$\Delta G(298)_{solv}$[b] [kcal/mol]
S1	-630.009944	82.254	-630.662934	-7.678	
PhIF2	-729.135848	36.472	-729.863735	-5.696	
[PhIF]-	-629.330424	34.467	-629.969021	-60.810	
PhI	-529.386914	35.958	-529.934159	-4.634	
PYR	-248.431835	37.507703	-248.698453	-3.03589	
INT1	-1359.145017	133.748	-1360.529448	-19.337	7.32
INT2	-1359.154063	134.826	-1360.537668	-18.192	4.38
INT3a	-1359.145183	134.770	-1360.526621	-18.024	11.43
INT3b	-1359.143018	134.064	-1360.524342	-17.991	12.18
INT2cat	-729.602534	81.839	-730.345072	-52.092	31.45
INT3cat	-729.616437	83.431	-730.357958	-47.618	29.43
INT1py	-978.133774	138.499	-979.147356	-51.056	-10.49
INT2py	-978.134098	138.653	-979.148798	-50.948	-11.13
INT3apy	-978.129511	138.896	-979.142254	-51.357	-7.19
INT3bpy	-978.128494	138.751	-979.141027	-50.795	-6.00
1	-829.826724	85.371	-830.675739	-8.207	-49.09

[a] PW6B95-D3//TPSS-D3/def2-TZVP+COSMO-RS($CHCl_3$)

[b] relative free energy calculated from eq. (1)

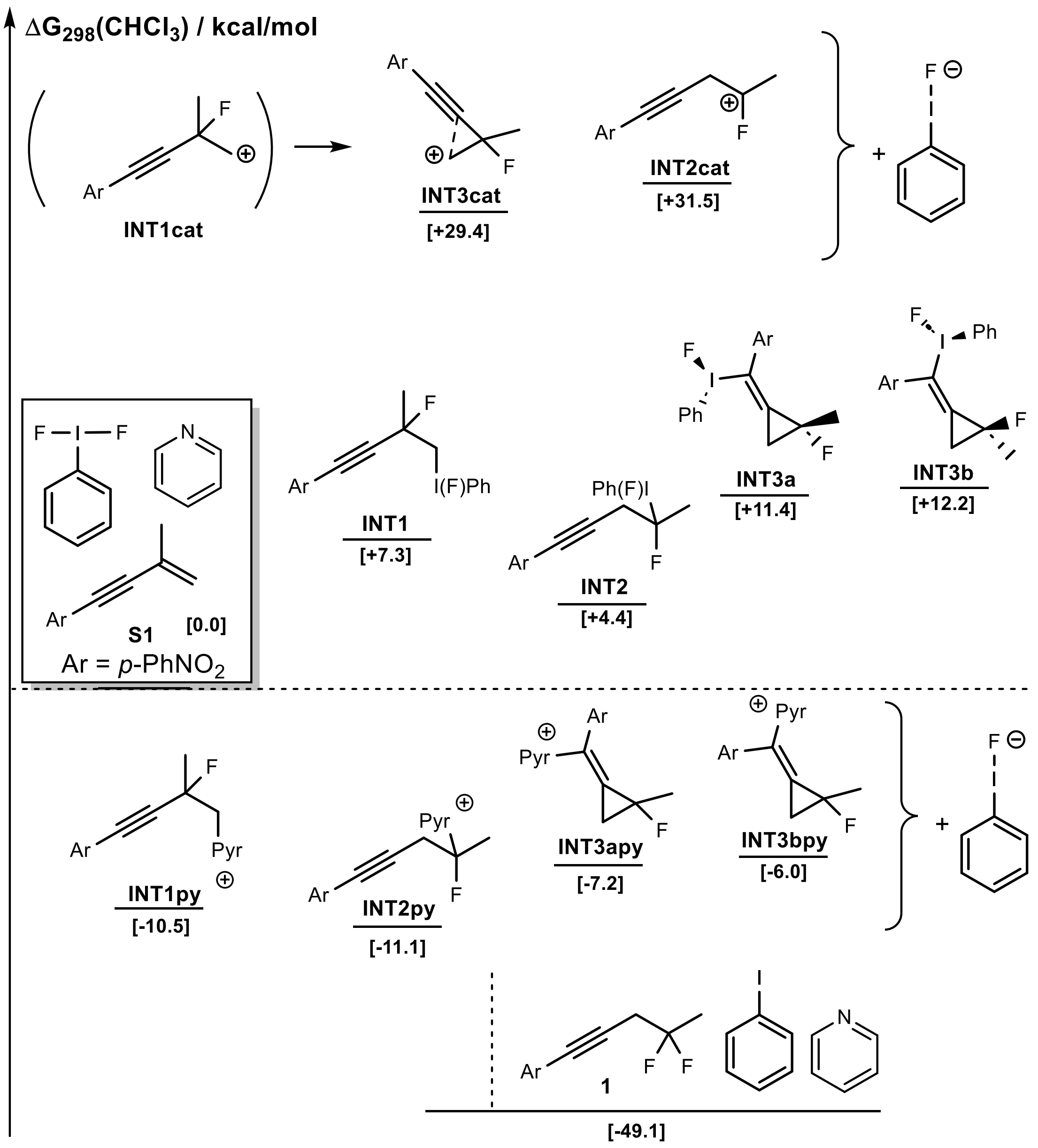

Supplementary Figure 2. Relative free energies of intermediates of the transformation **S1→1**. All values of ΔG_{298} are given relative to isolated **S1**, pyridine and $PhIF_2$ in chloroform.

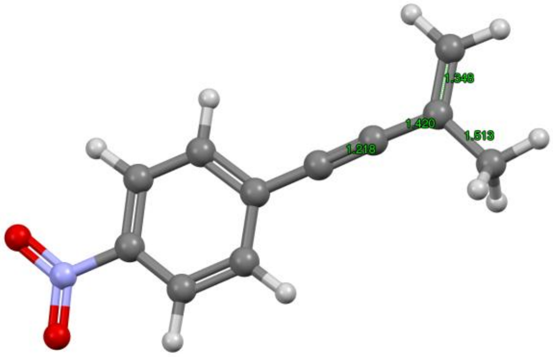

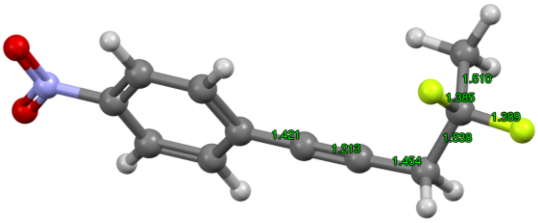

S1 **1**

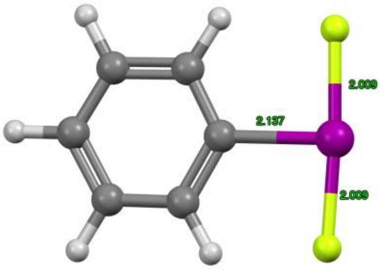

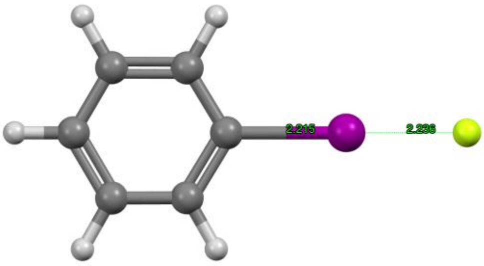

PhIF$_2$ **PhIF$^-$**

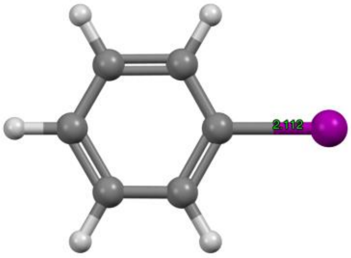

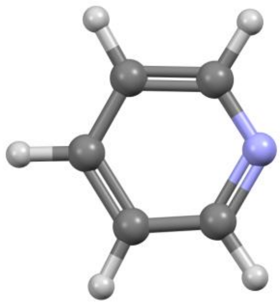

PhI **PYR**

Supplementary Figure 3. Molecular structures of all intermediates optimized with TPSS-D3/def2-TZVP. Distances are given in Å. Element colors are gray (C), white (H), blue (N), red (O), yellow (F), purple (I).

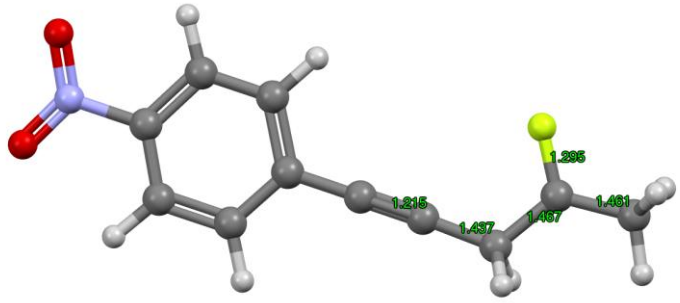

INT2cat

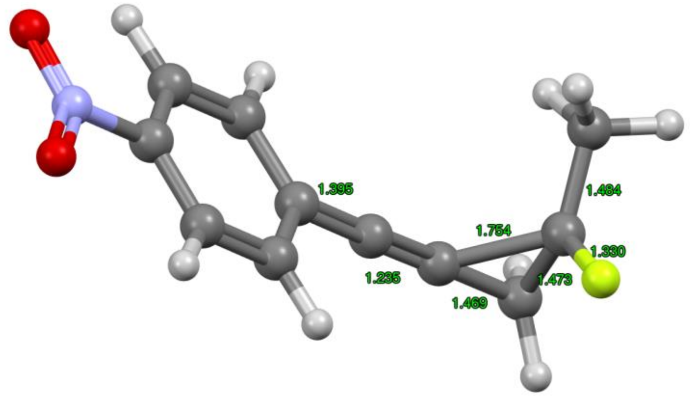

INT3cat

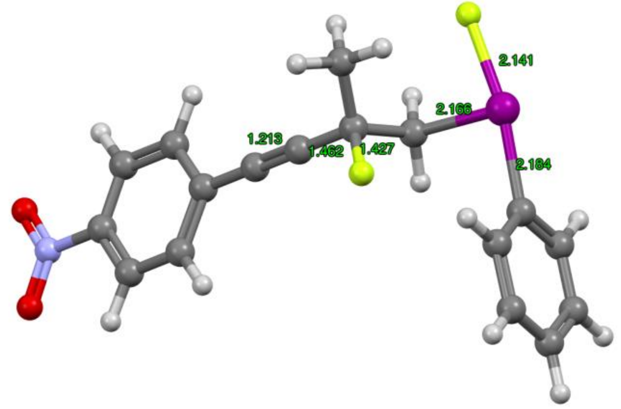

INT1

Supplementary Figure 3 (continued). Molecular structures of all intermediates optimized with TPSS-D3/def2-TZVP. Distances are given in Å. Element colors are gray (C), white (H), blue (N), red (O), yellow (F), purple (I).

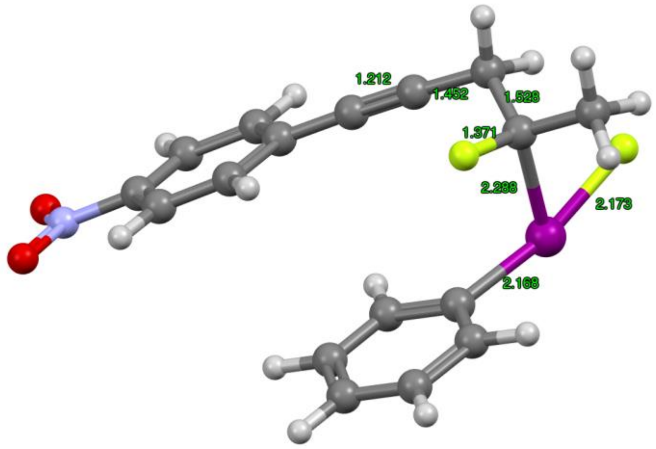

INT2

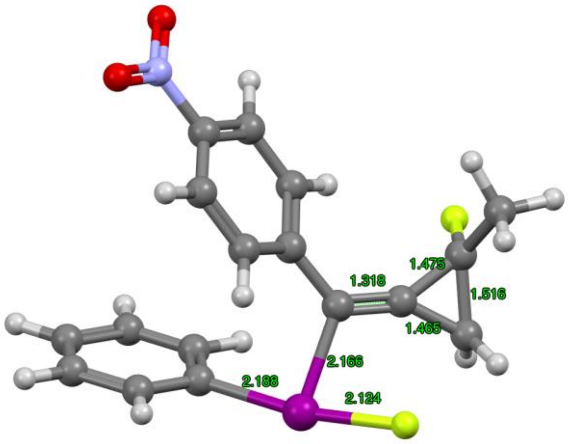

INT3a

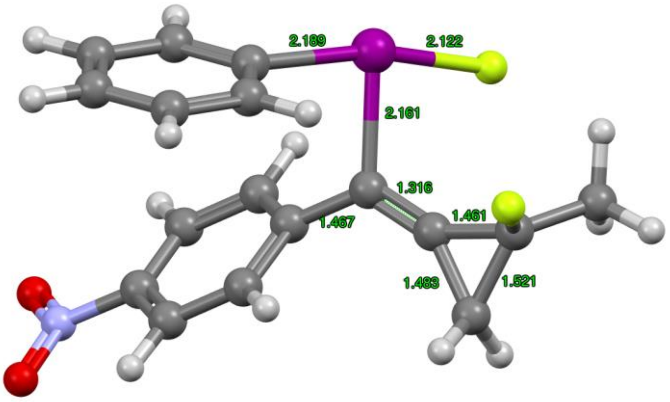

INT3b

Supplementary Figure 3 (continued). Molecular structures of all intermediates optimized with TPSS-D3/def2-TZVP. Distances are given in Å. Element colors are gray (C), white (H), blue (N), red (O), yellow (F), purple (I).

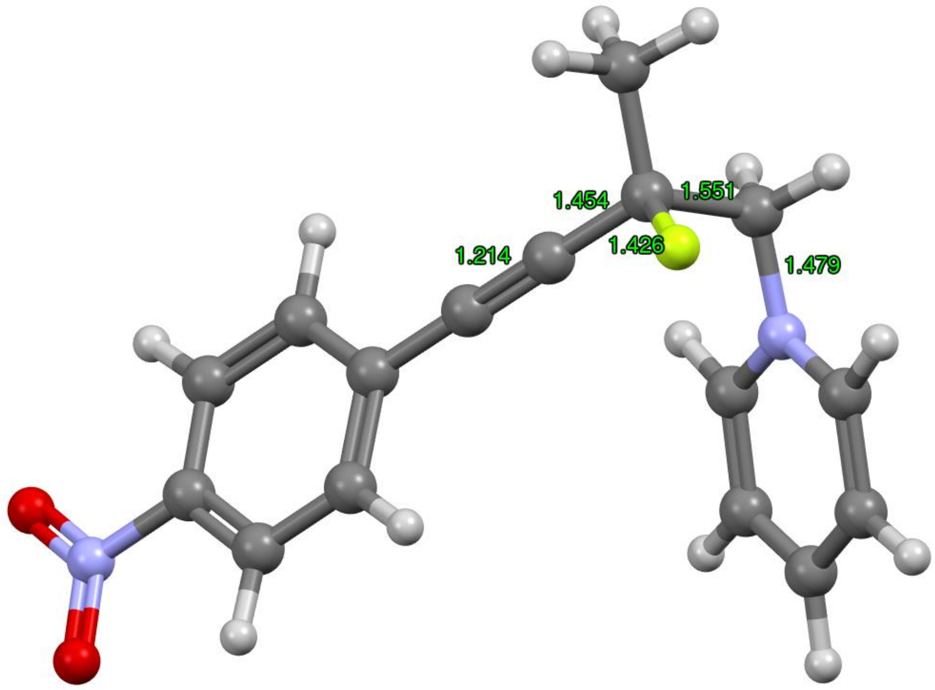

INT1py

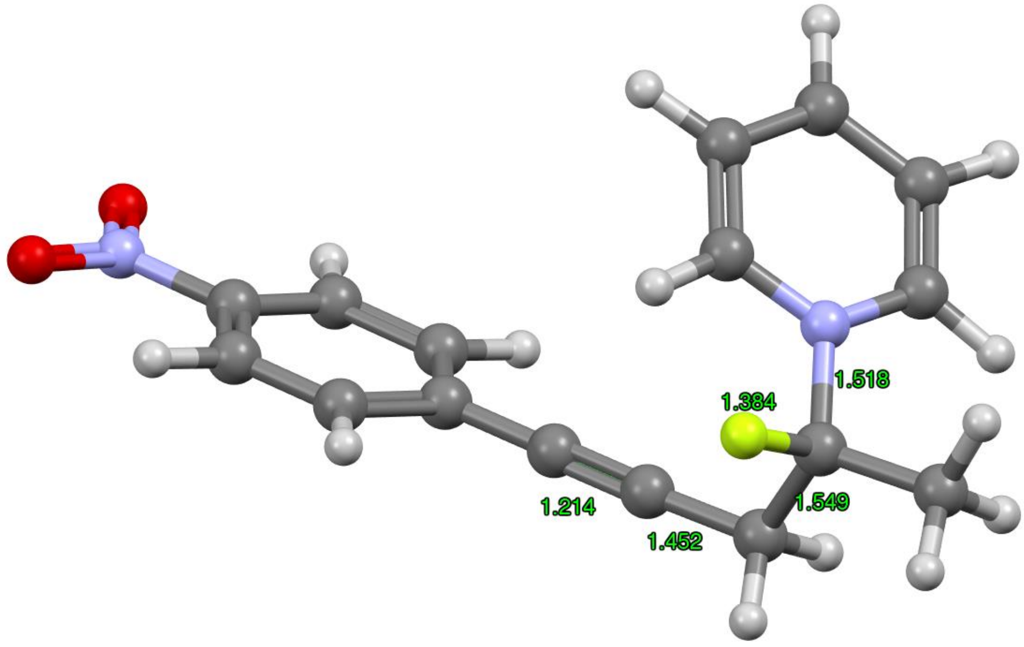

INT2py

Supplementary Figure 3 (continued). Molecular structures of all intermediates optimized with TPSS-D3/def2-TZVP. Distances are given in Å. Element colors are gray (C), white (H), blue (N), red (O), yellow (F), purple (I).

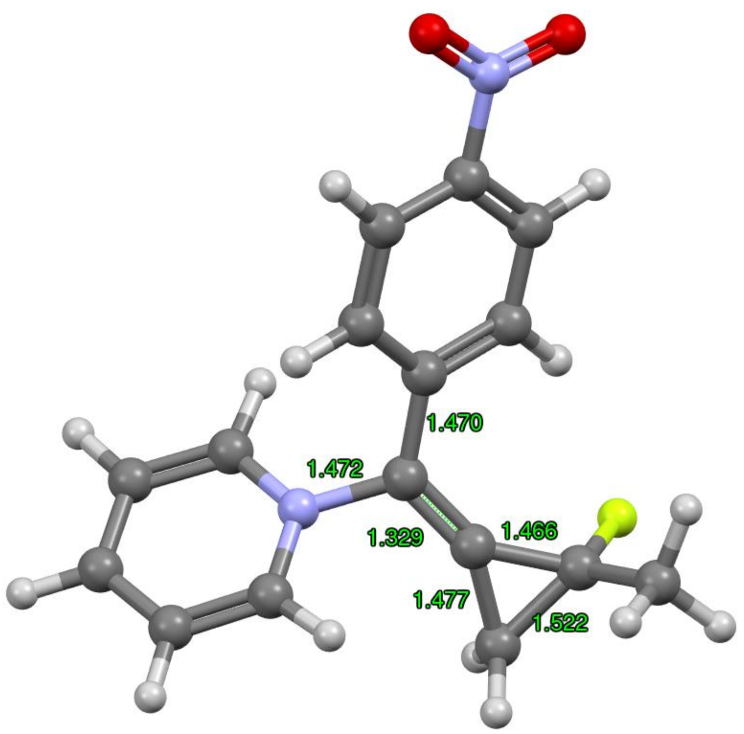

INT3apy

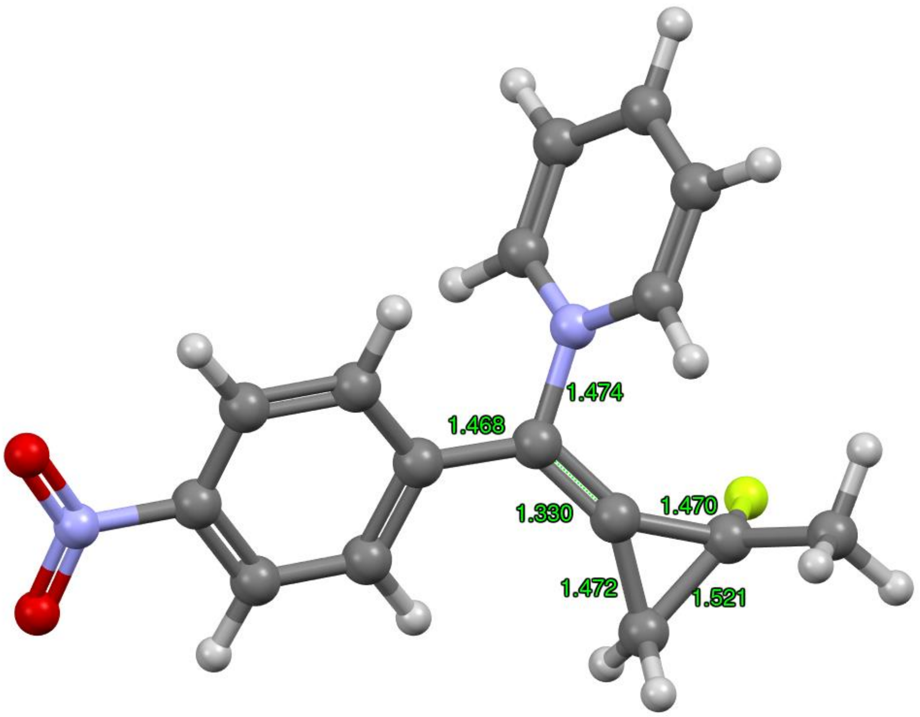

INT3bpy

Supplementary Figure 3 (continued). Molecular structures of all intermediates optimized with TPSS-D3/def2-TZVP. Distances are given in Å. Element colors are gray (C), white (H), blue (N), red (O), yellow (F), purple (I).

1.8 NMR-Spectra of key compounds

1-(3-Methylbut-3-en-1-yn-1-yl)-4-nitrobenzene (S1)

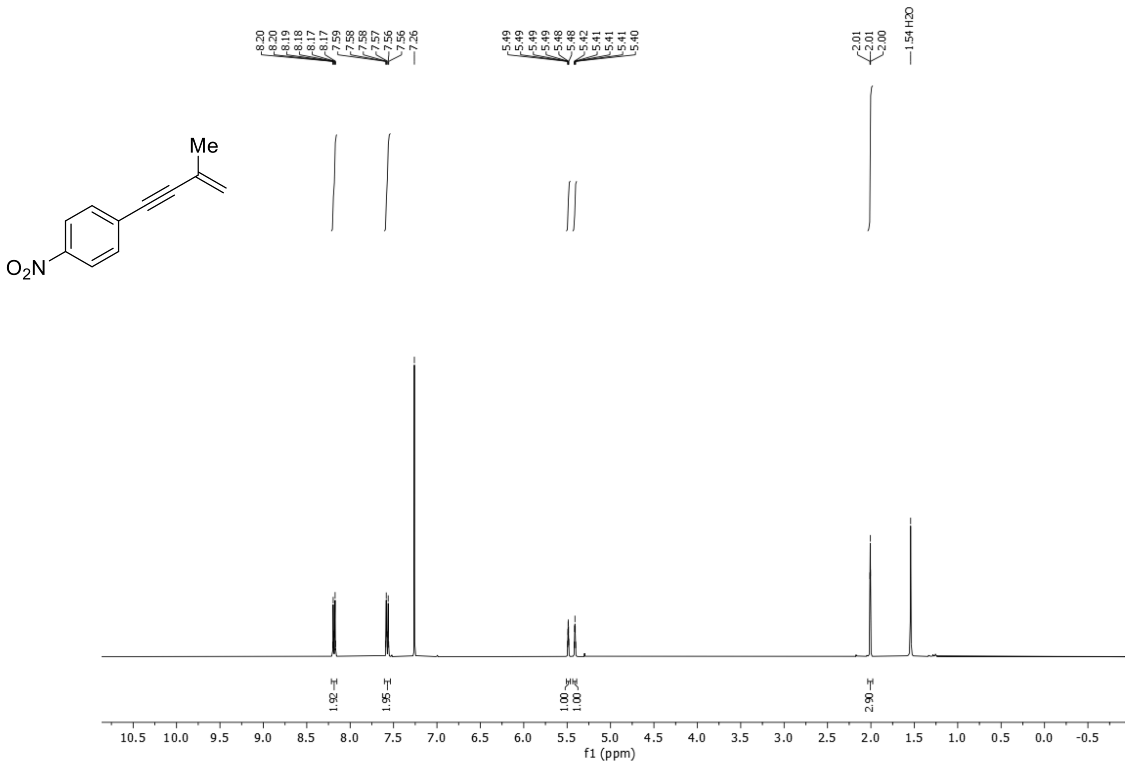

Supplementary Figure 4. ^{1}H NMR of **S1** (400 MHz, 299 K, $CDCl_3$).

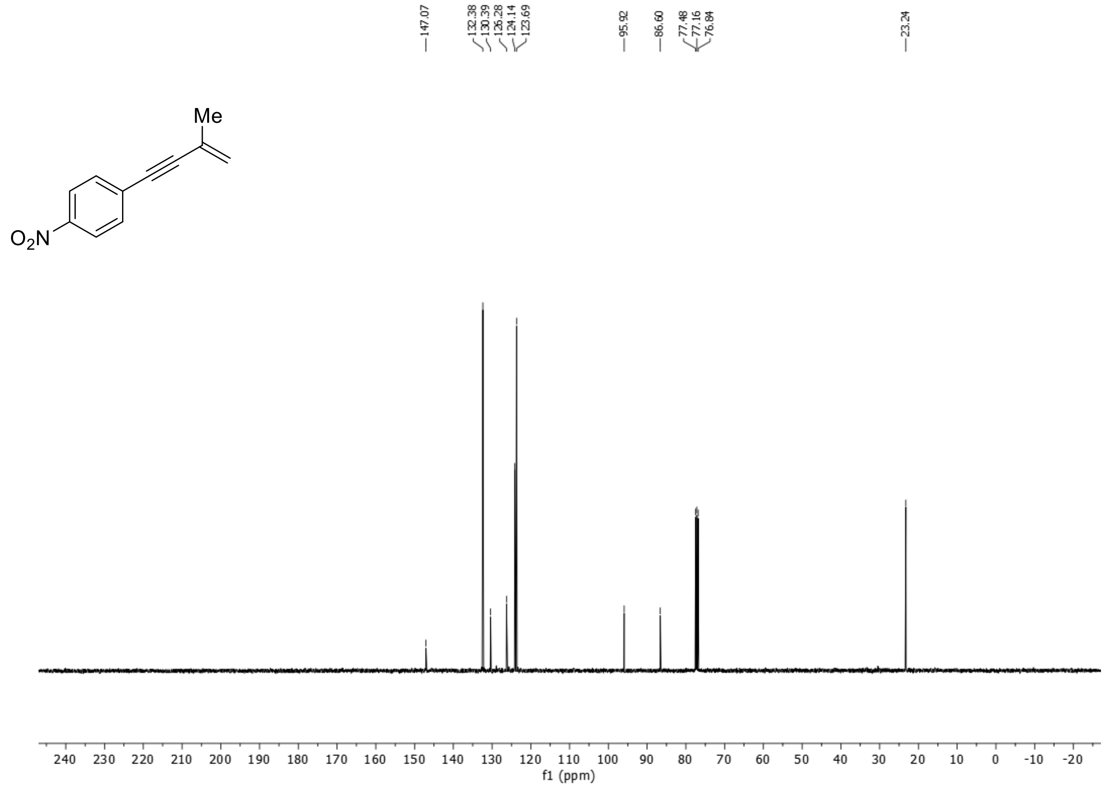

Supplementary Figure 5. $^{13}C\{^{1}H\}$ NMR of **S1** (101 MHz, 299 K, $CDCl_3$).

4-(3-Methylbut-3-en-1-yn-1-yl)benzonitrile (S2)

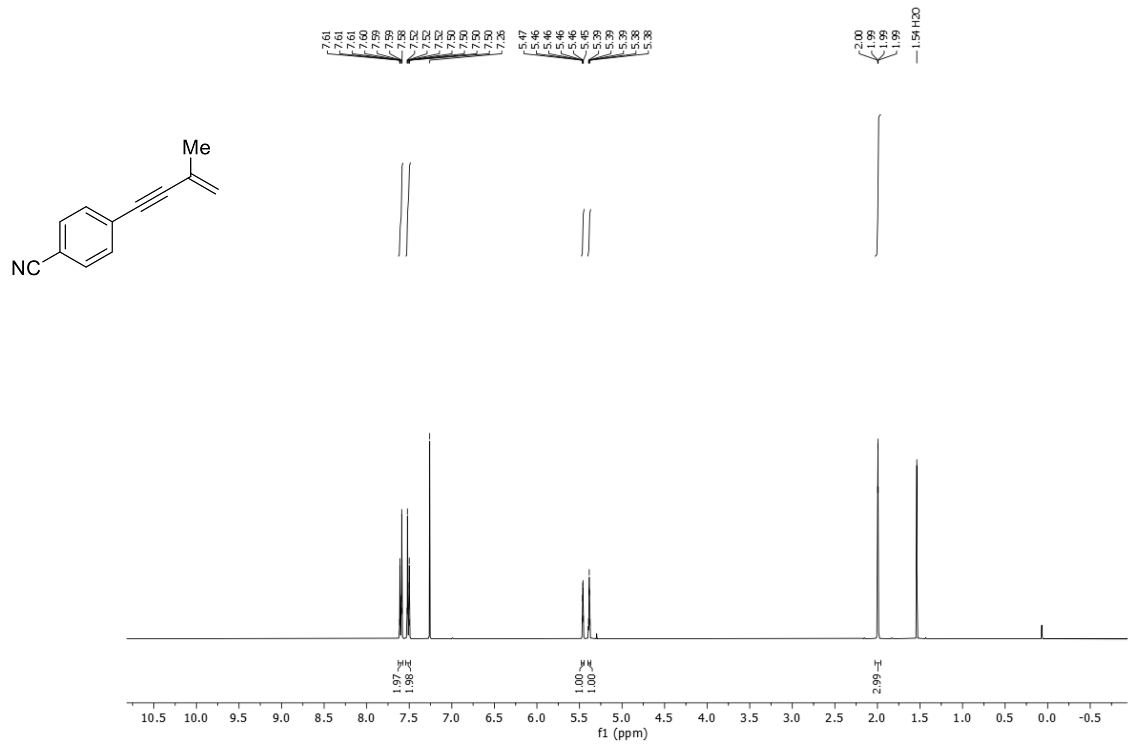

Supplementary Figure 6. ^{1}H NMR of **S2** (400 MHz, 299 K, $CDCl_3$).

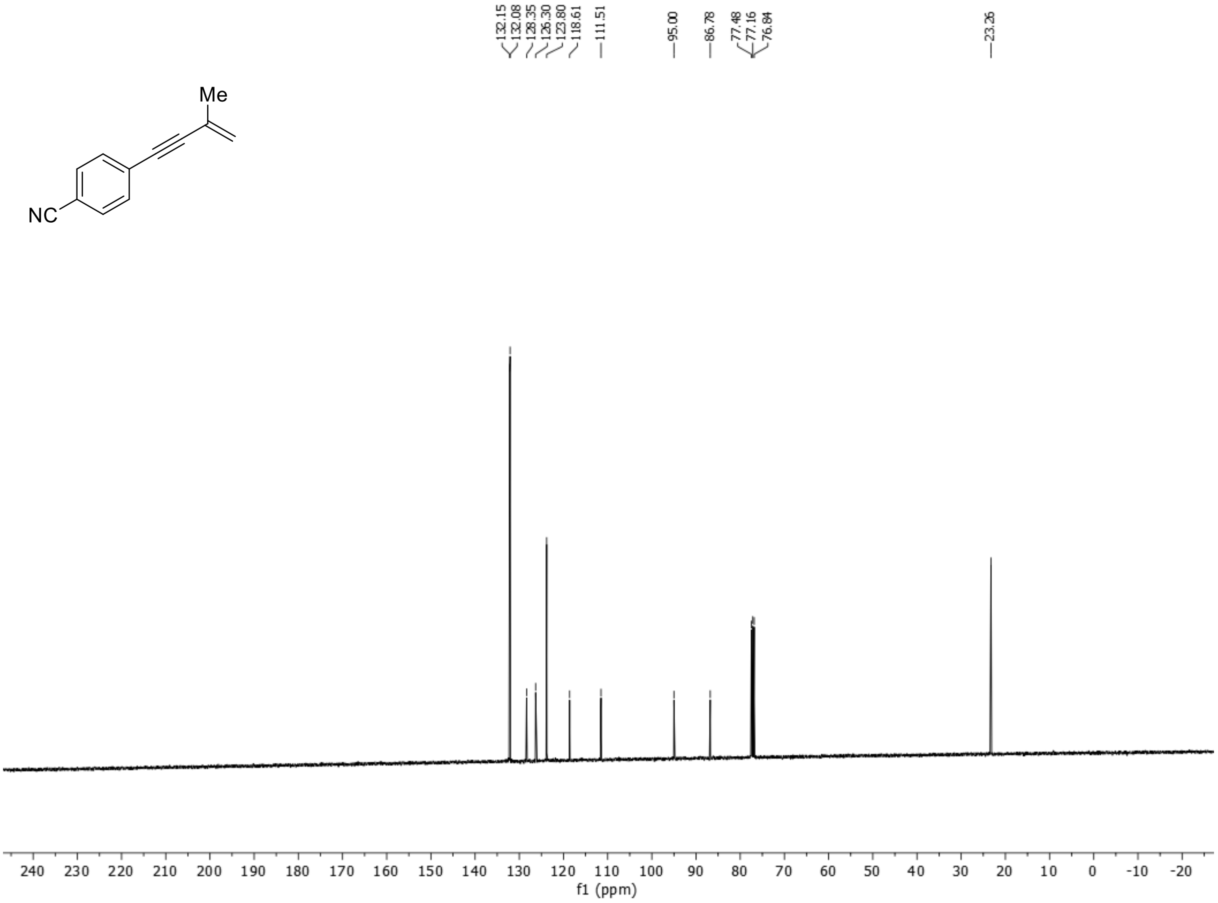

Supplementary Figure 7. $^{13}C\{^{1}H\}$ NMR of **S2** (101 MHz, 299 K, $CDCl_3$).

1-(3-Methylbut-3-en-1-yn-1-yl)-3,5-bis(trifluoromethyl)benzene (S3)

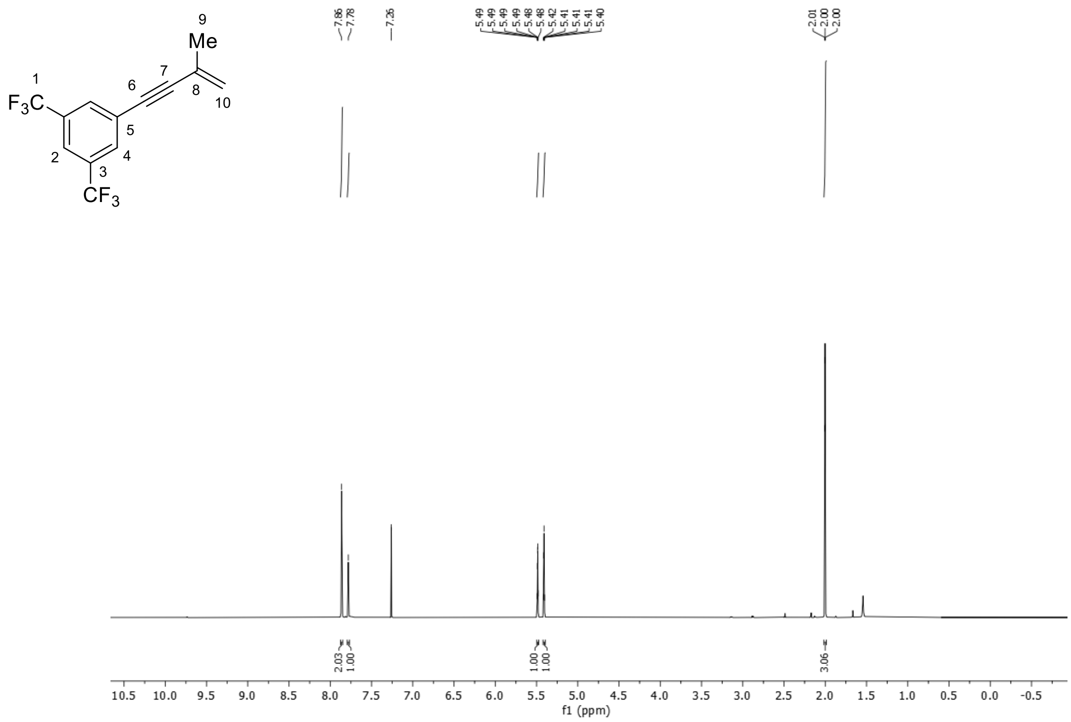

Supplementary Figure 8. ^{1}H NMR of **S3** (500 MHz, 299 K, $CDCl_3$).

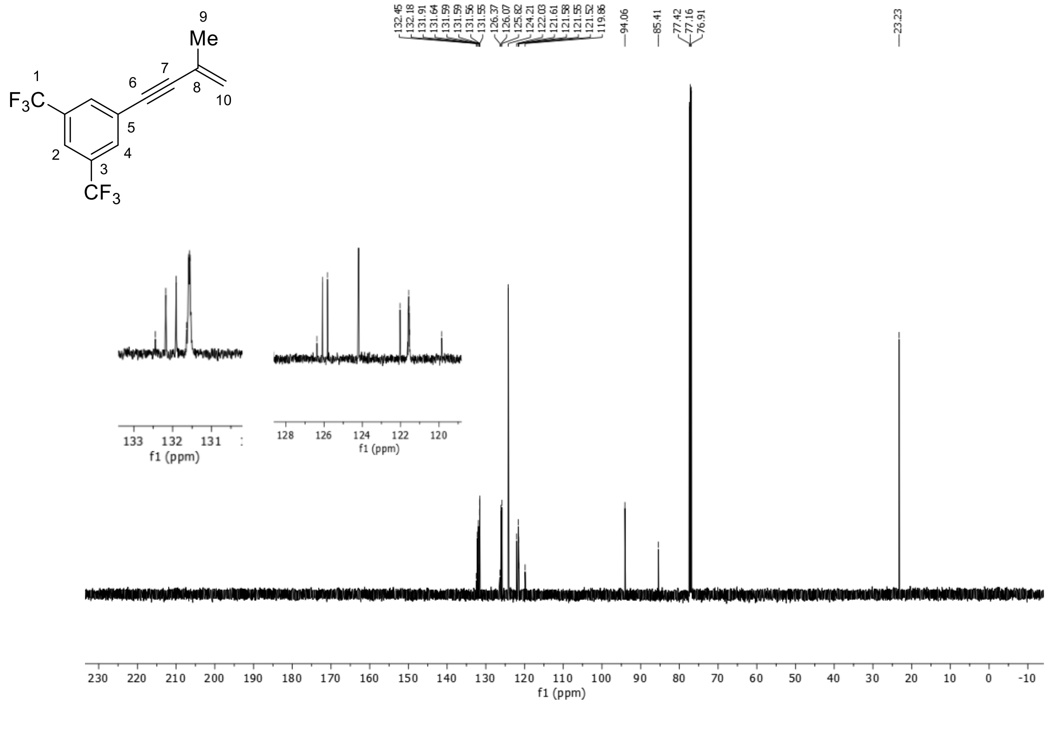

Supplementary Figure 9. $^{13}C\{^{1}H\}$ NMR of **S3** (126 MHz, 299 K, $CDCl_3$).

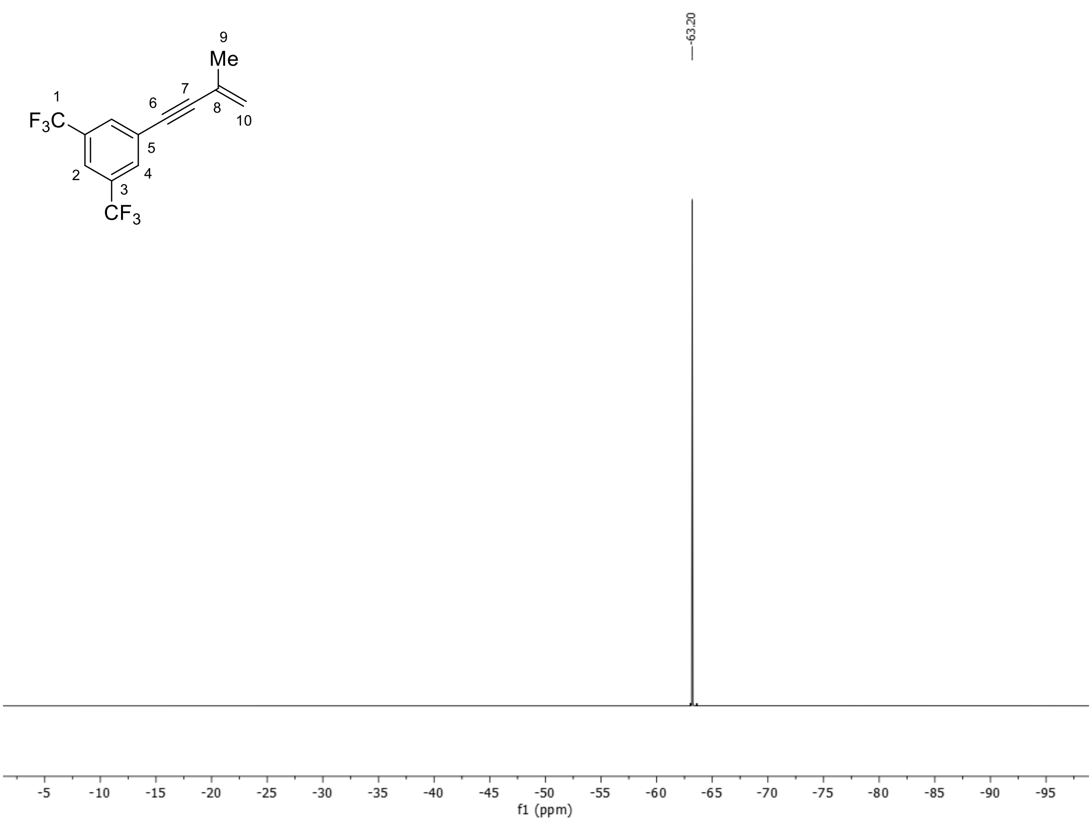

Supplementary Figure 10. ^{19}F NMR of **S3** (470 MHz, 299 K, $CDCl_3$).

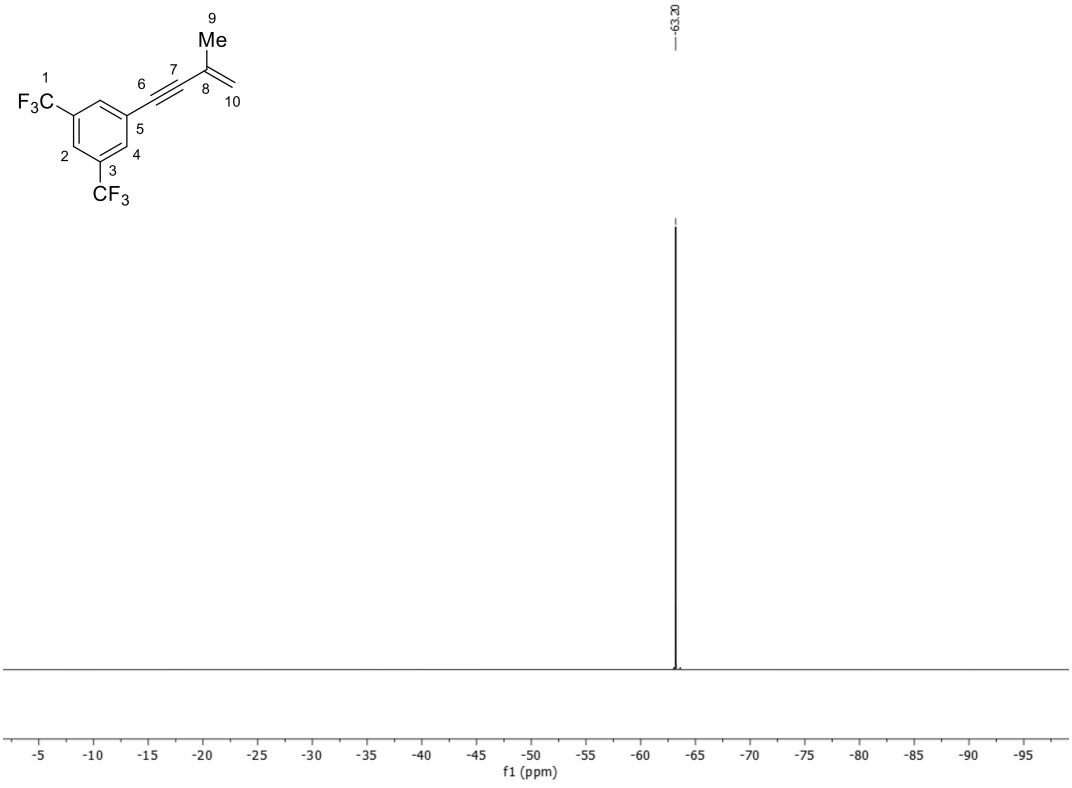

Supplementary Figure 11. $^{19}F\{^1H\}$ NMR of **S3** (470 MHz, 299 K, $CDCl_3$).

1-Chloro-4-(3-methylbut-3-en-1-yn-1-yl)benzene (S4)

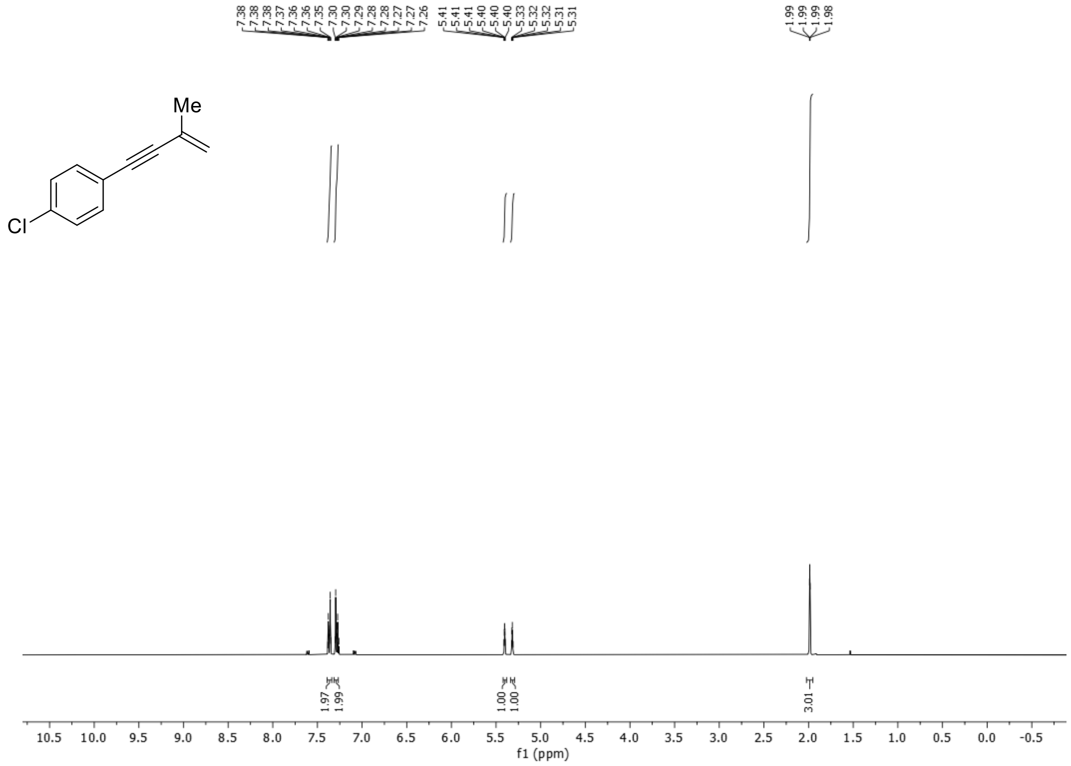

Supplementary Figure 12. ^{1}H NMR of **S4** (400 MHz, 299 K, $CDCl_3$).

1-Chloro-3-(3-methylbut-3-en-1-yn-1-yl)benzene (S5)

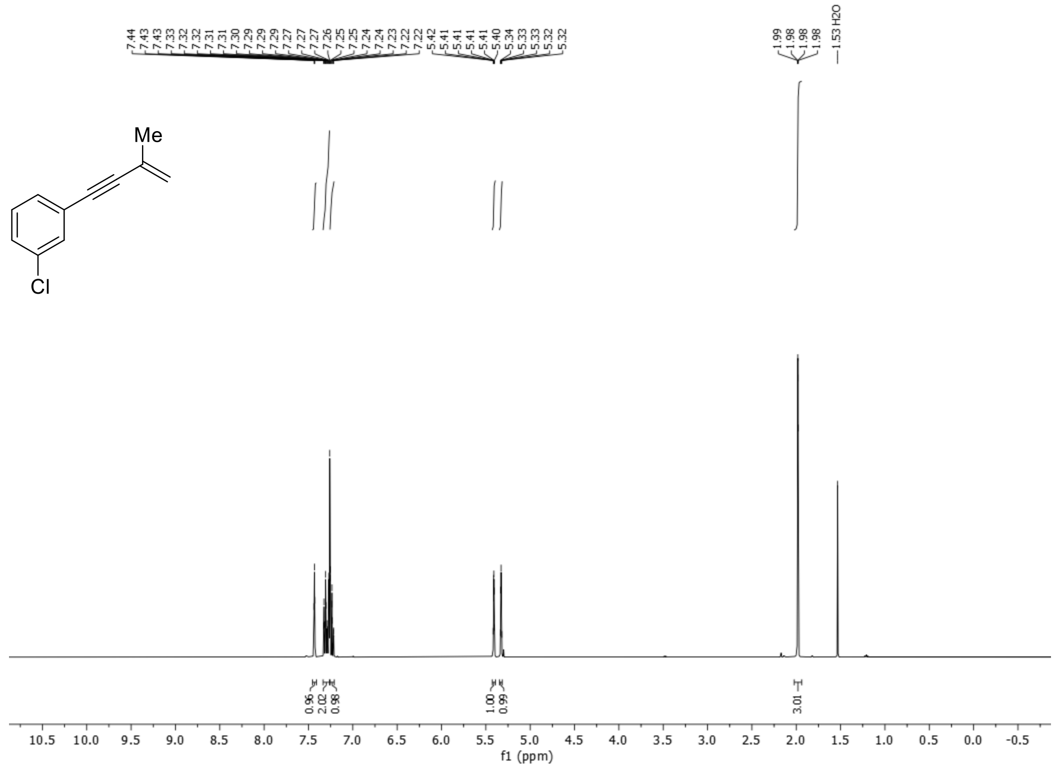

Supplementary Figure 13. ^{1}H NMR of **S5** (400 MHz, 299 K, $CDCl_3$).

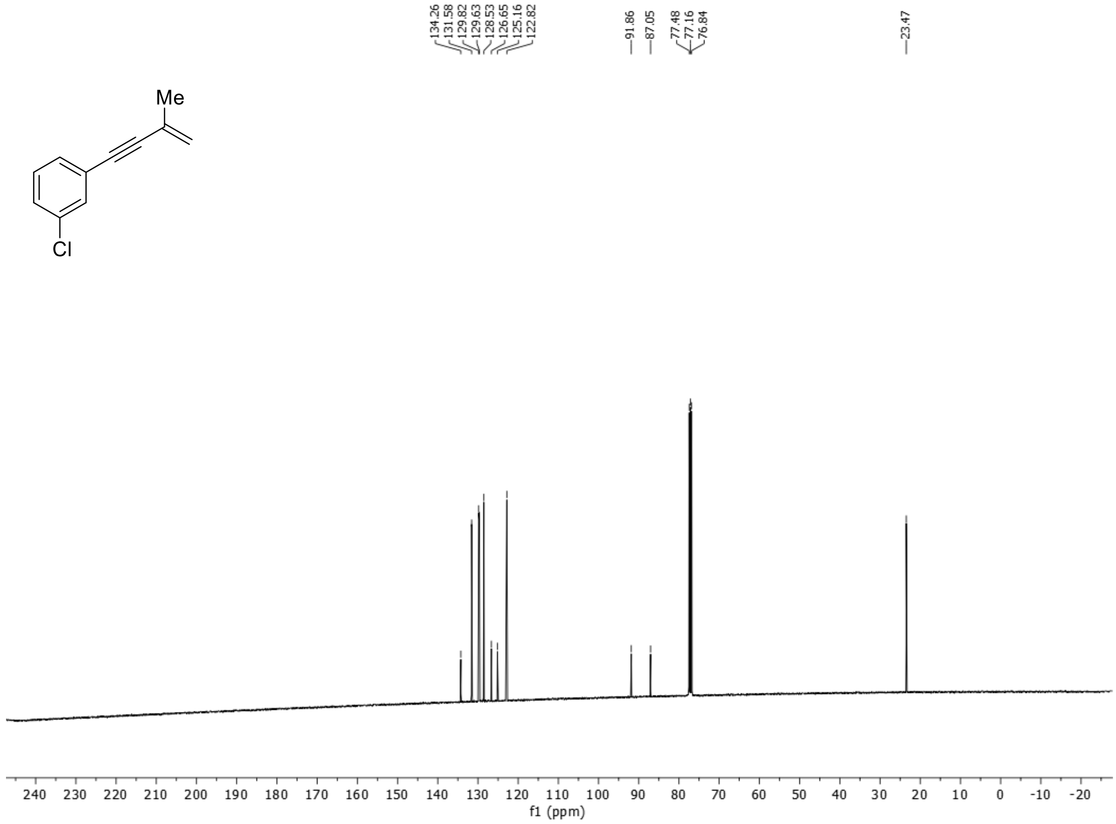

Supplementary Figure 14. $^{13}C\{^{1}H\}$ NMR of **S5** (101 MHz, 299 K, $CDCl_3$).

1-Bromo-4-(3-methylbut-3-en-1-yn-1-yl)benzene (S6)

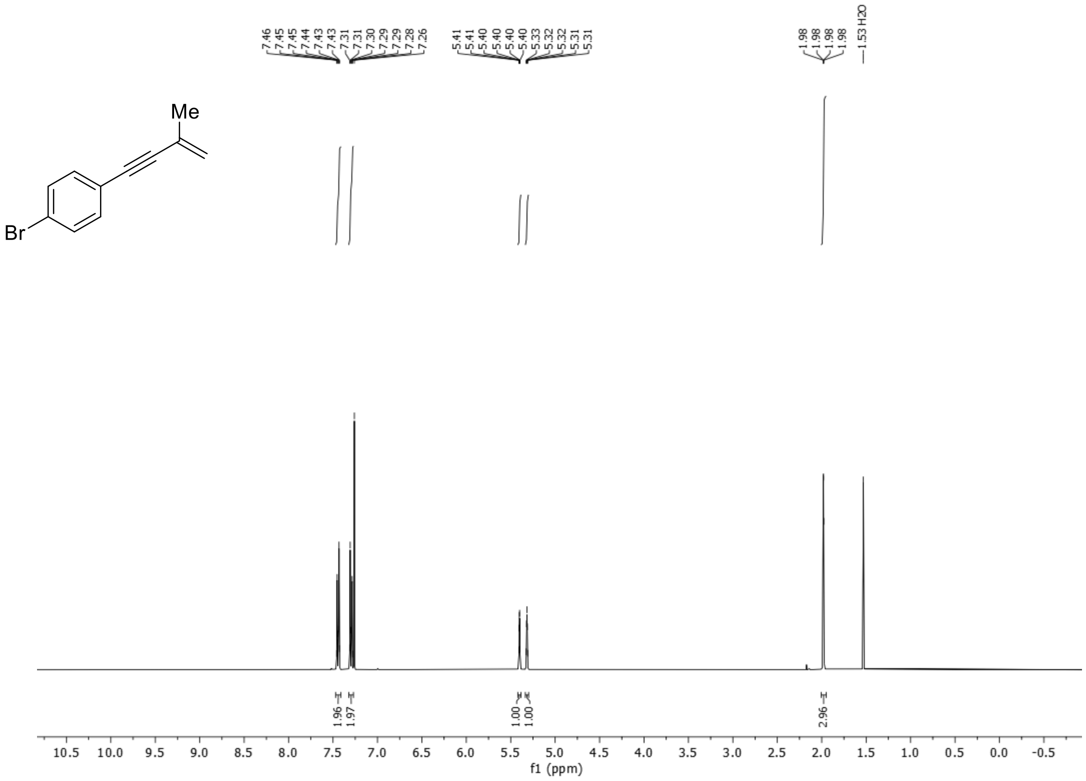

Supplementary Figure 15. ^{1}H NMR of **S6** (400 MHz, 299 K, $CDCl_3$).

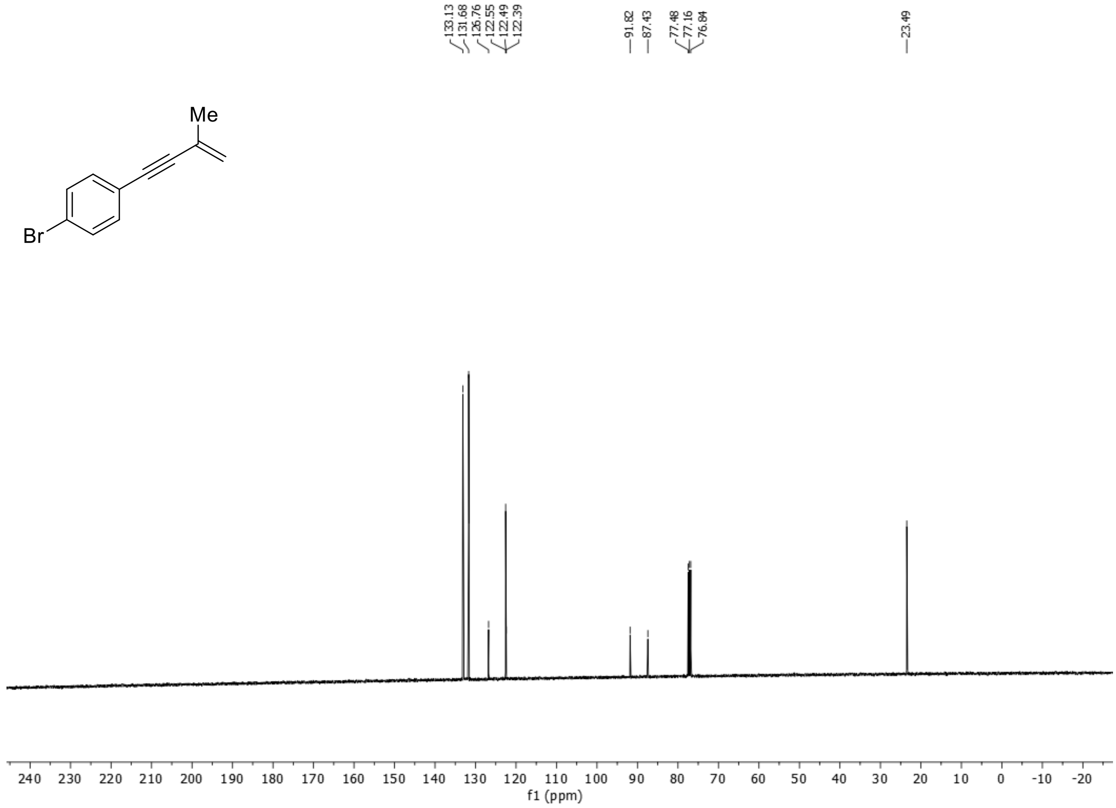

Supplementary Figure 16. $^{13}C\{^{1}H\}$ NMR of **S6** (101 MHz, 299 K, $CDCl_3$).

Methyl 4-(3-methylbut-3-en-1-yn-1-yl)benzoate (S7)

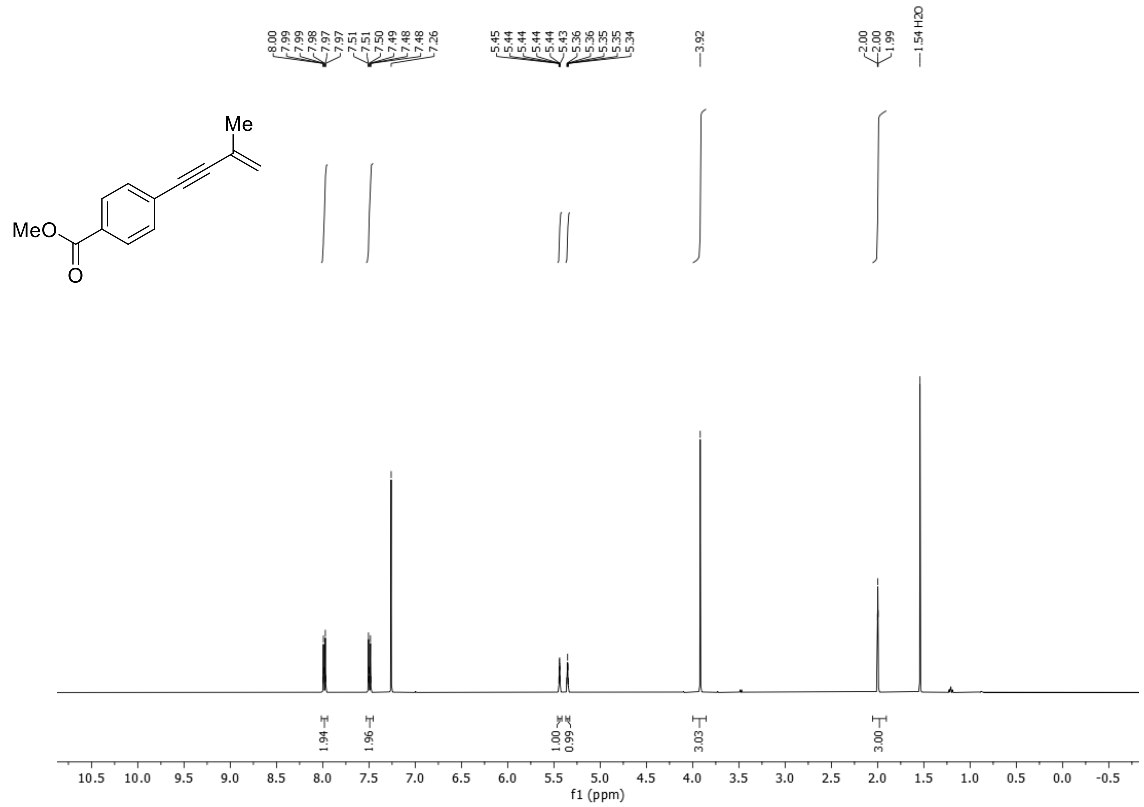

Supplementary Figure 17. ^{1}H NMR of **S7** (400 MHz, 299 K, $CDCl_3$).

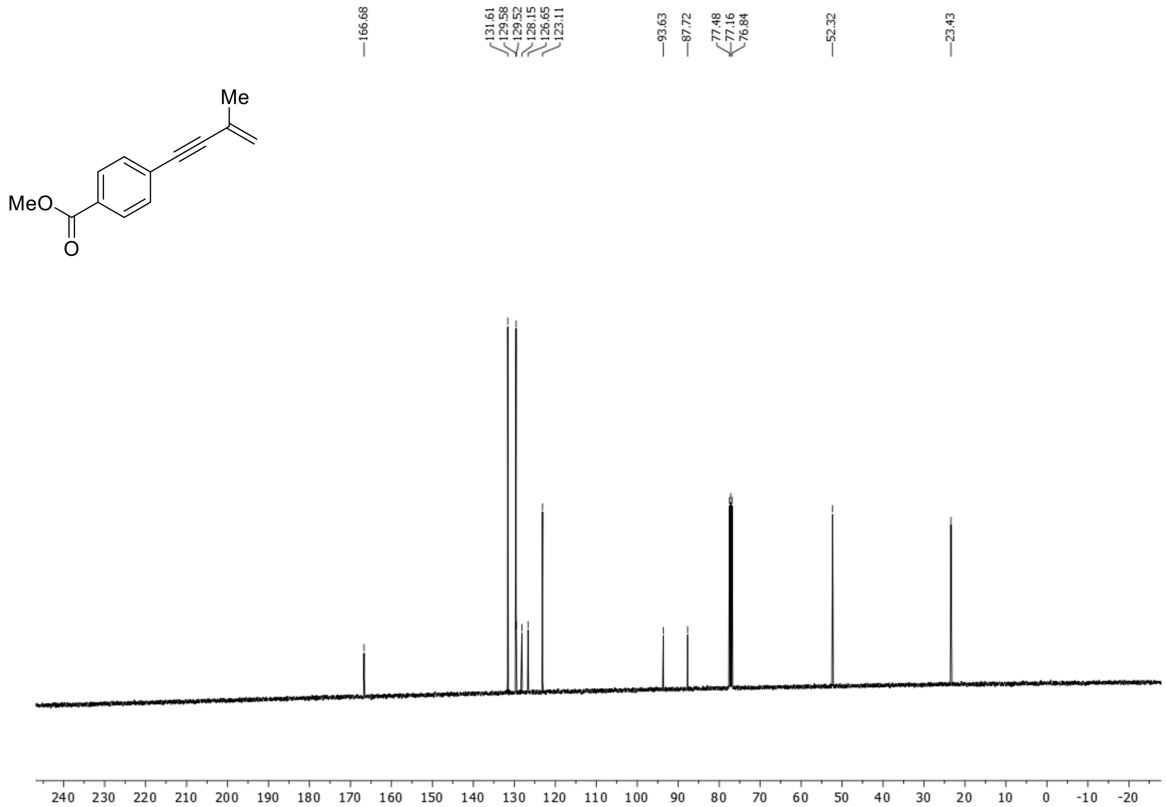

Supplementary Figure 18. $^{13}C\{^{1}H\}$ NMR of **S7** (101 MHz, 299 K, $CDCl_3$).

1-(3-Methylbut-3-en-1-yn-1-yl)-4-(methylsulfonyl)benzene (S8)

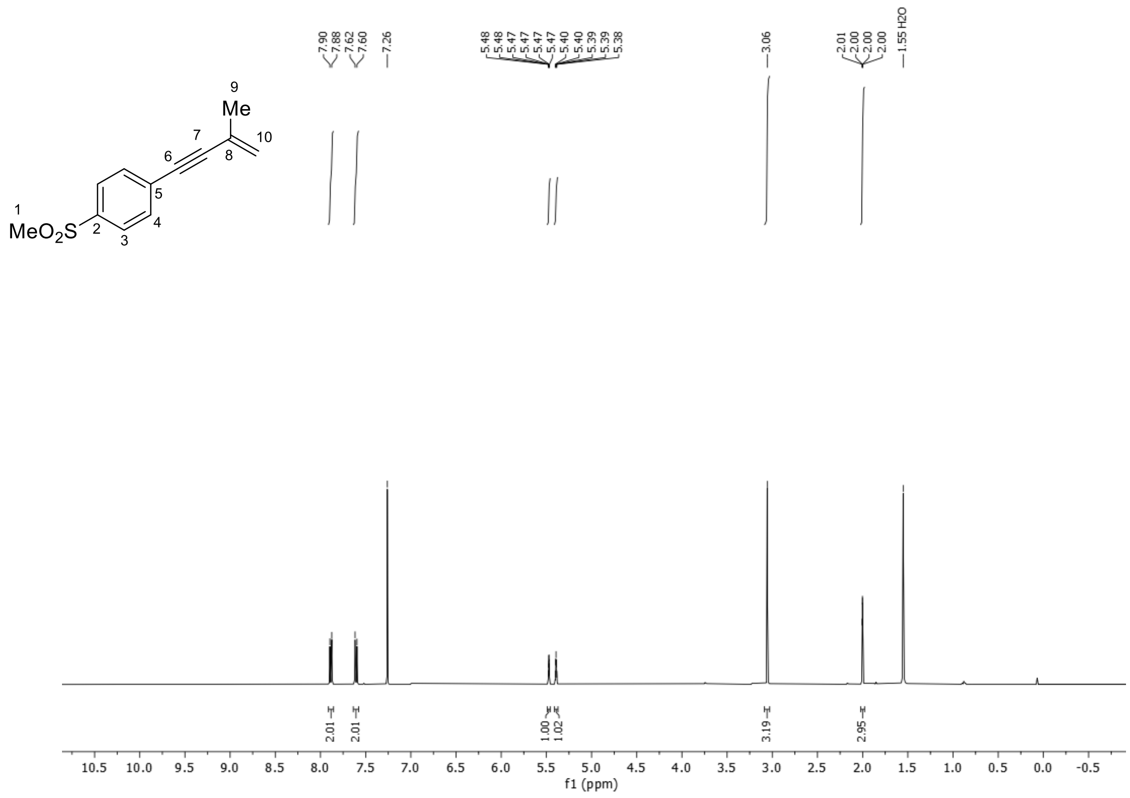

Supplementary Figure 19. ^{1}H NMR of **S8** (400 MHz, 299 K, $CDCl_3$).

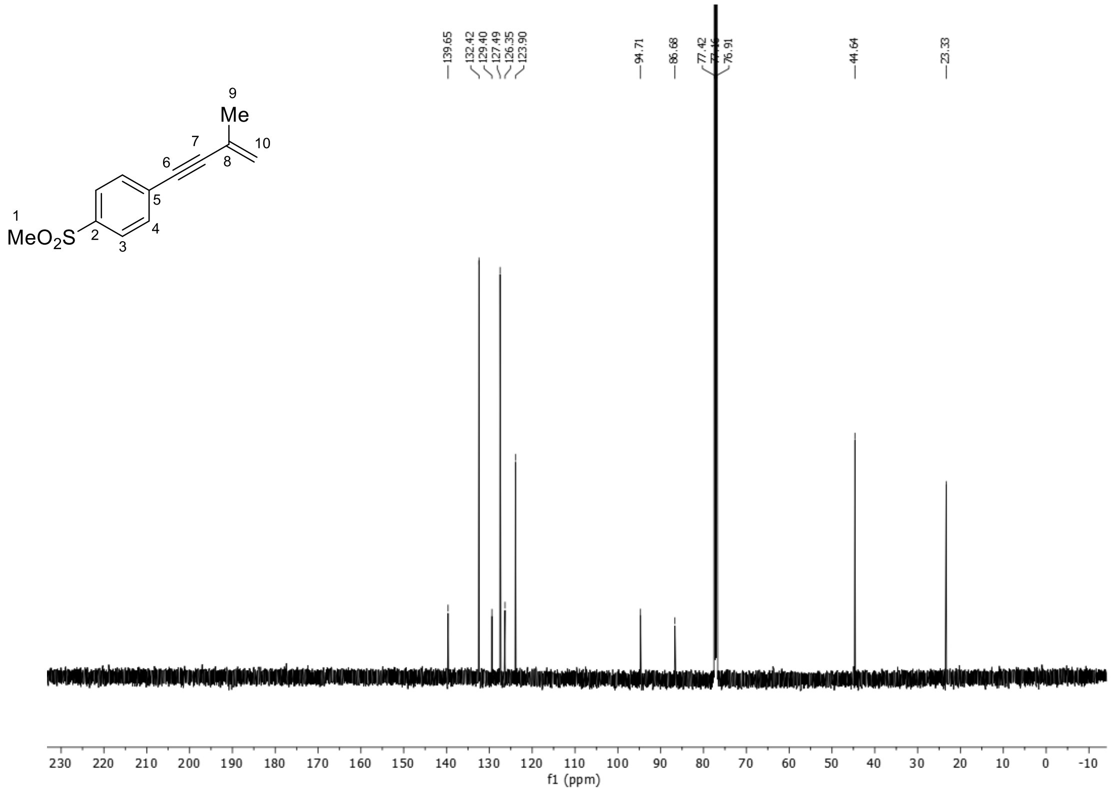

Supplementary Figure 20. $^{13}C\{^{1}H\}$ NMR of **S8** (126 MHz, 299 K, $CDCl_3$).

2,2,2-Trifluoro-1-(4-(3-methylbut-3-en-1-yn-1-yl)phenyl)ethan-1-one (S9)

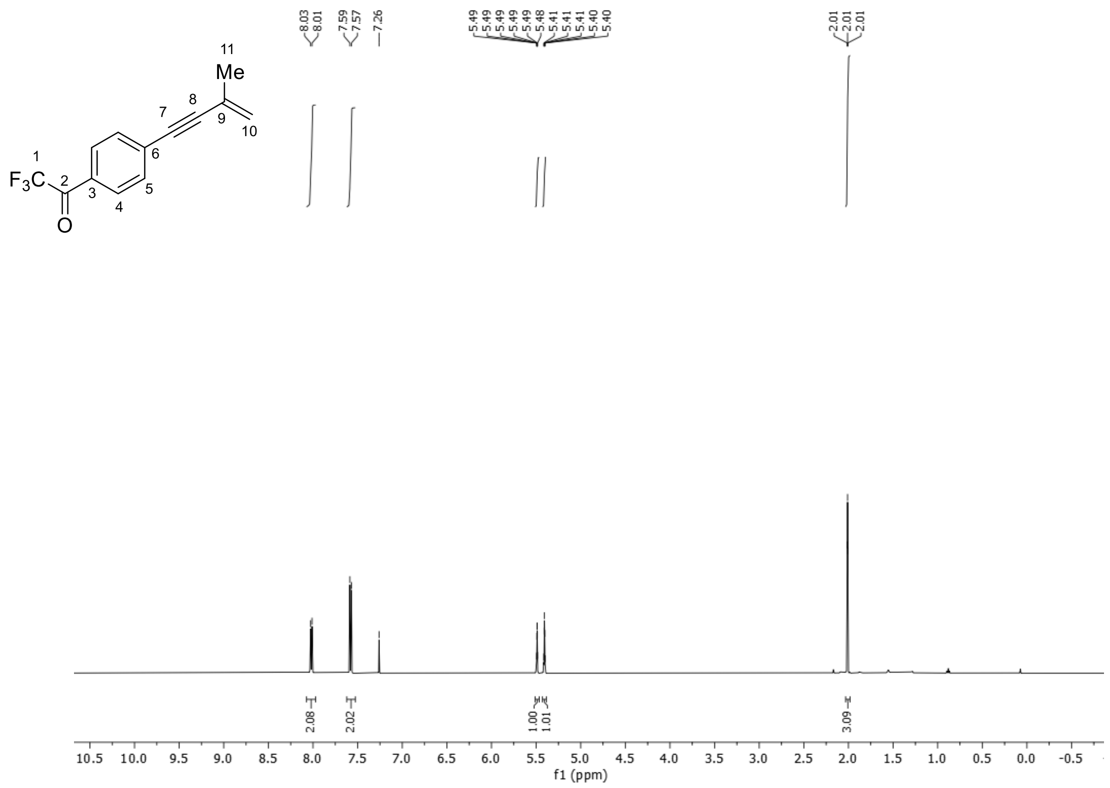

Supplementary Figure 21. ^{1}H NMR of **S9** (500 MHz, 299 K, $CDCl_3$).

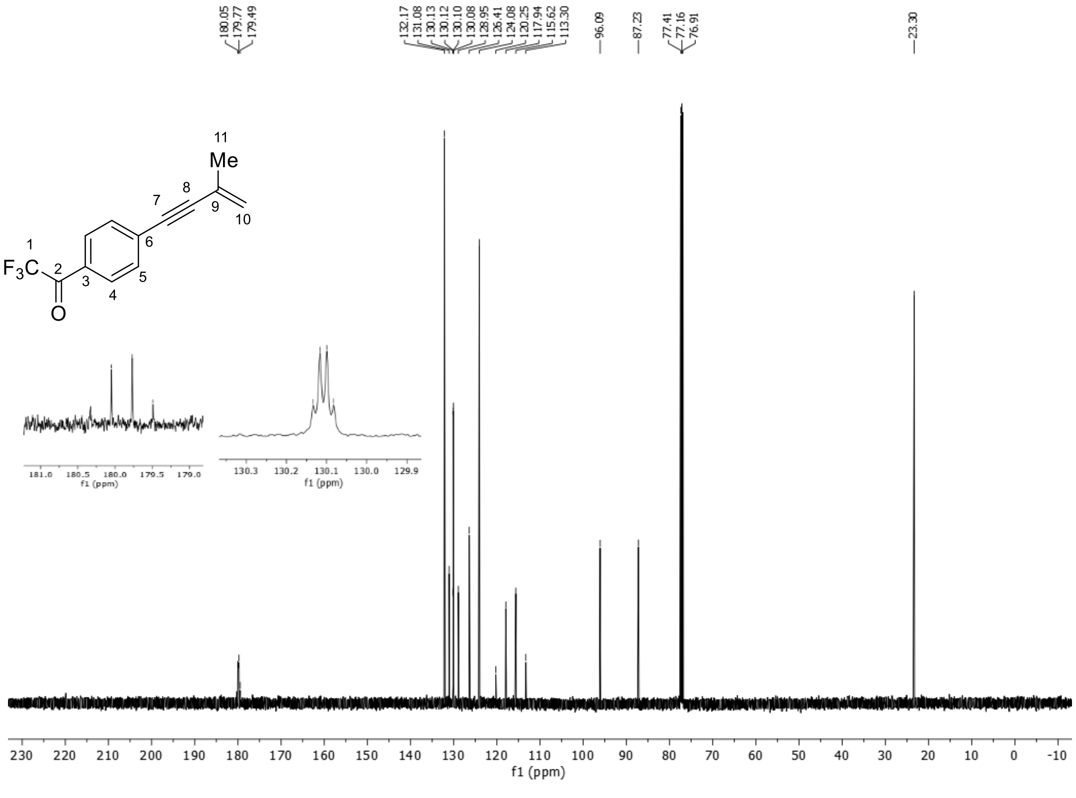

Supplementary Figure 22. $^{13}C\{^1H\}$ NMR of **S9** (126 MHz, 299 K, $CDCl_3$).

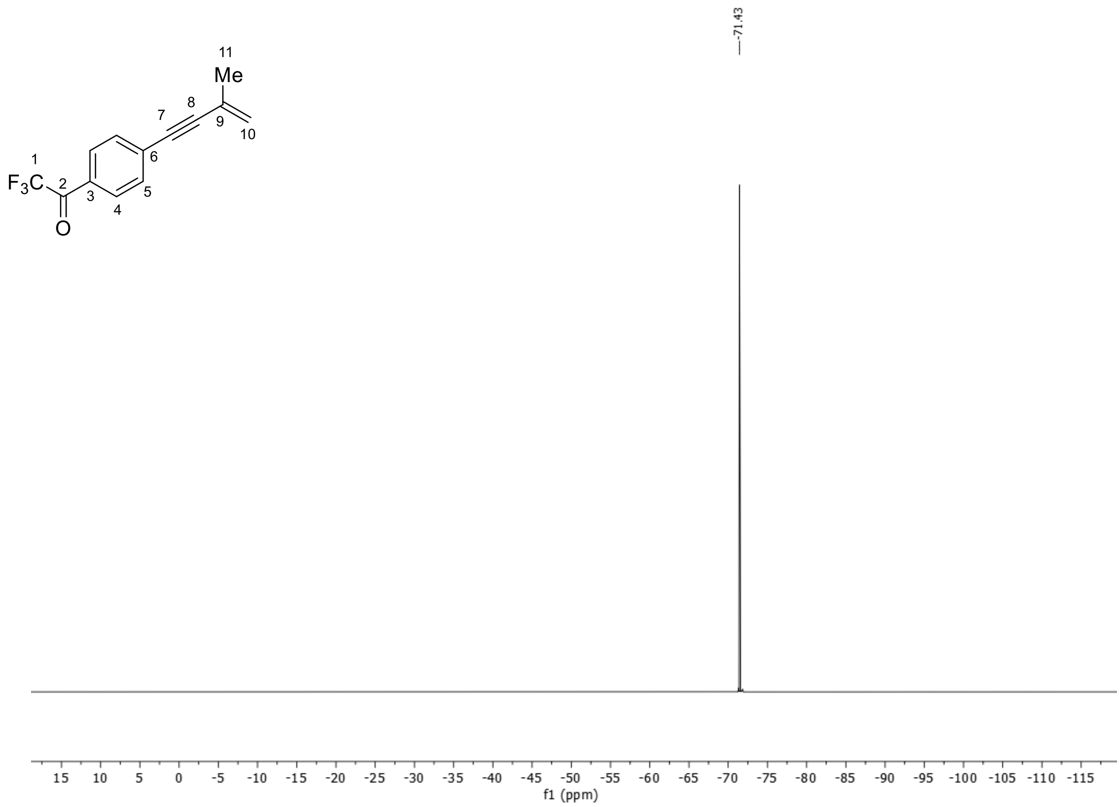

Supplementary Figure 23. ^{19}F NMR of **S9** (470 MHz, 299 K, $CDCl_3$).

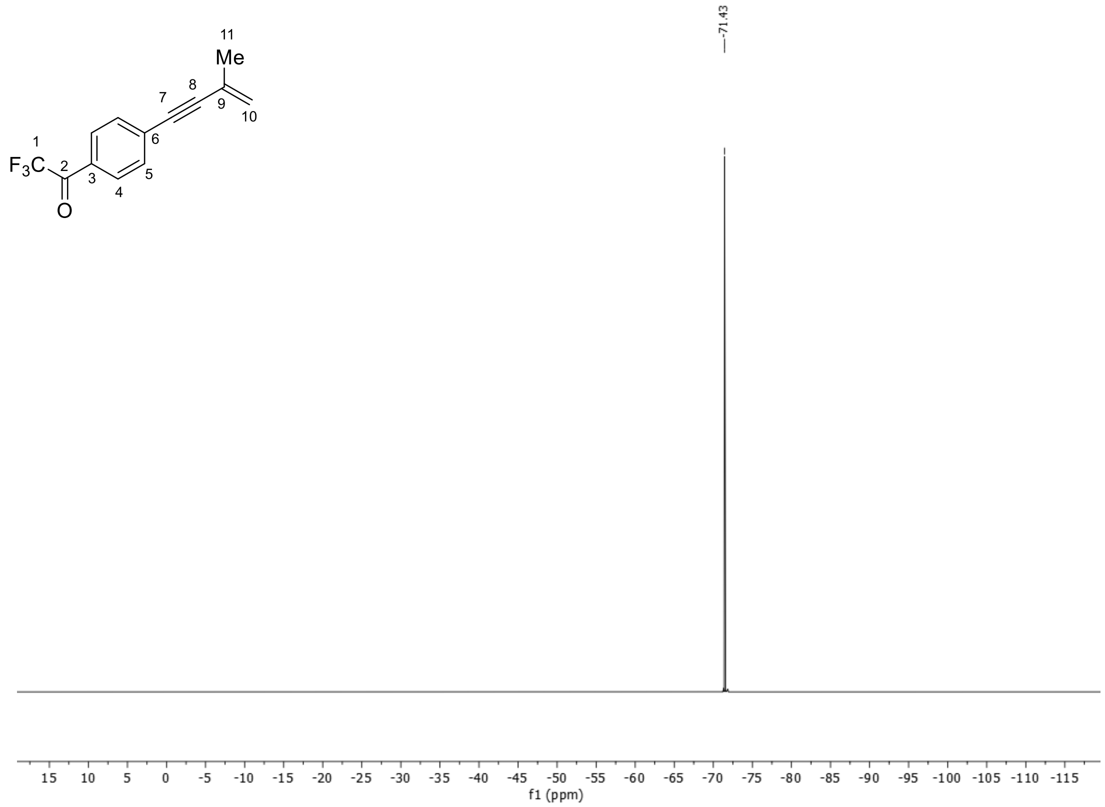

Supplementary Figure 24. ^{19}F{^{1}H} NMR of **S9** (470 MHz, 299 K, $CDCl_3$).

1-Methyl-2-(3-methylbut-3-en-1-yn-1-yl)benzene (S10)

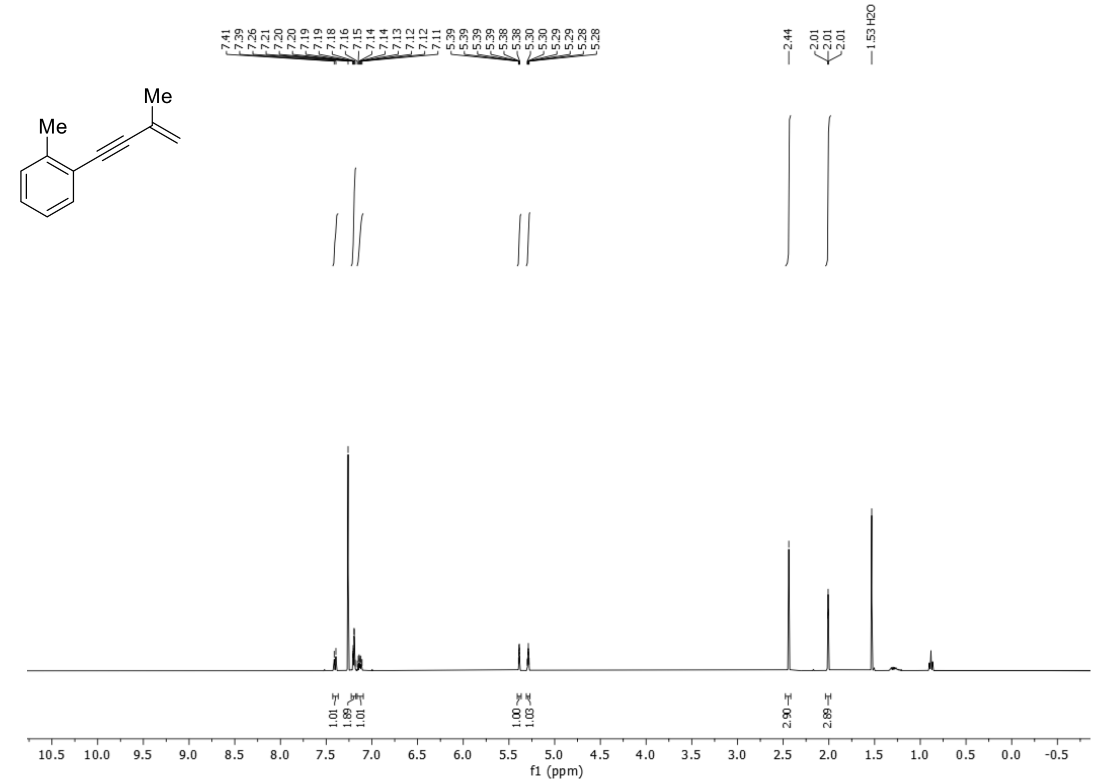

Supplementary Figure 25. ^{1}H NMR of **S10** (400 MHz, 299 K, $CDCl_3$).

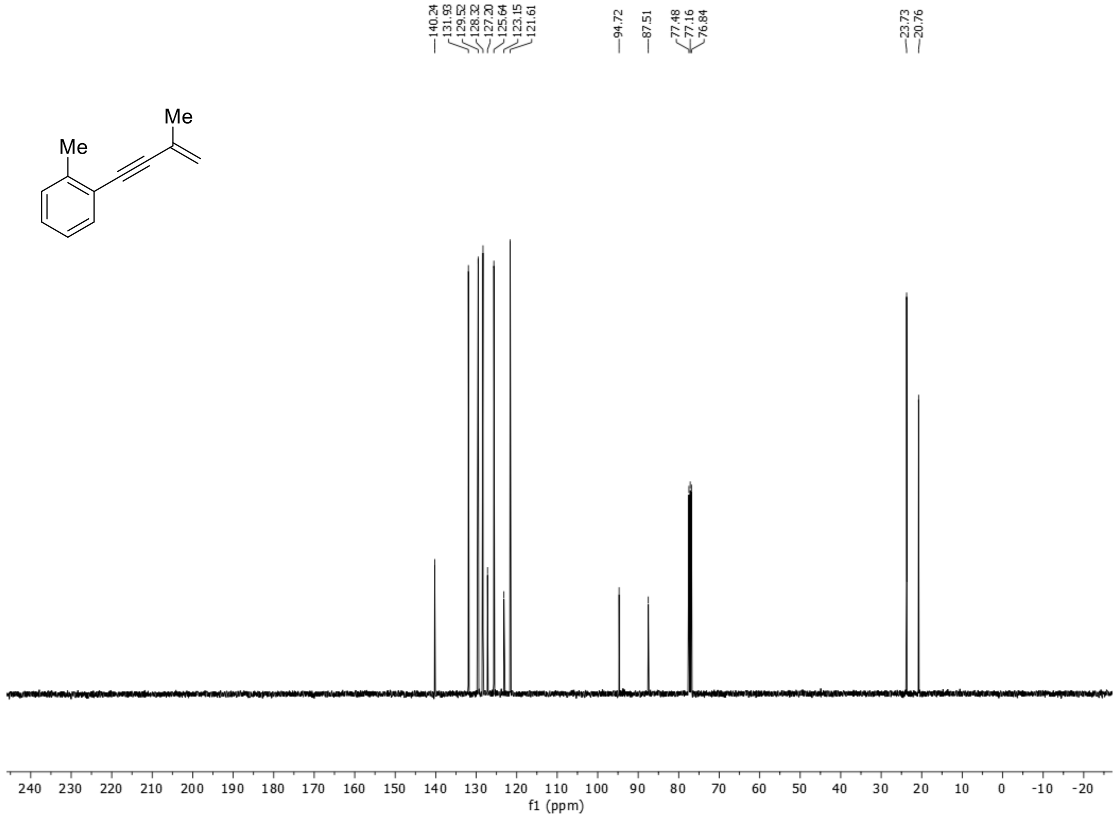

Supplementary Figure 26. $^{13}C\{^{1}H\}$ NMR of **S10** (101 MHz, 299 K, $CDCl_3$).

3-(3-Methylbut-3-en-1-yn-1-yl)pyridine (S11)

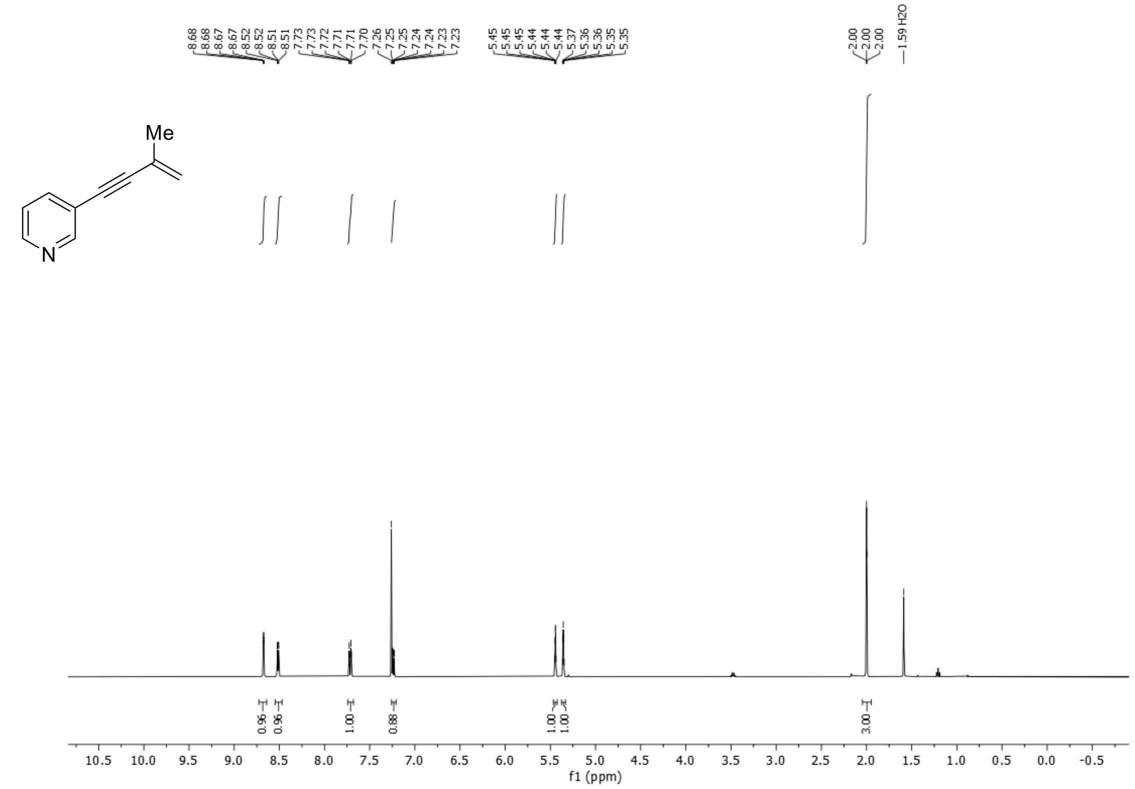

Supplementary Figure 27. ^{1}H NMR of **S11** (400 MHz, 299 K, $CDCl_3$).

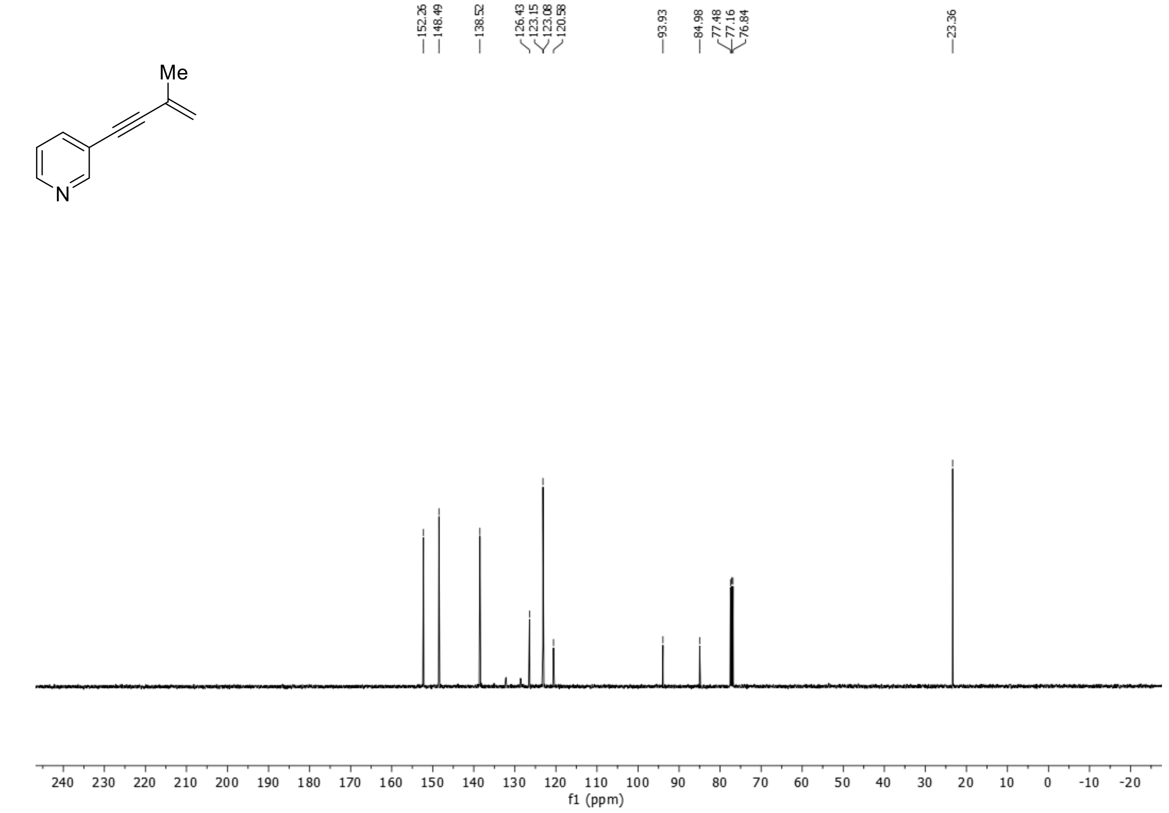

Supplementary Figure 28. $^{13}C\{^{1}H\}$ NMR of **S11** (101 MHz, 299 K, $CDCl_3$).

6-(3-Methylbut-3-en-1-yn-1-yl)quinoline (S12)

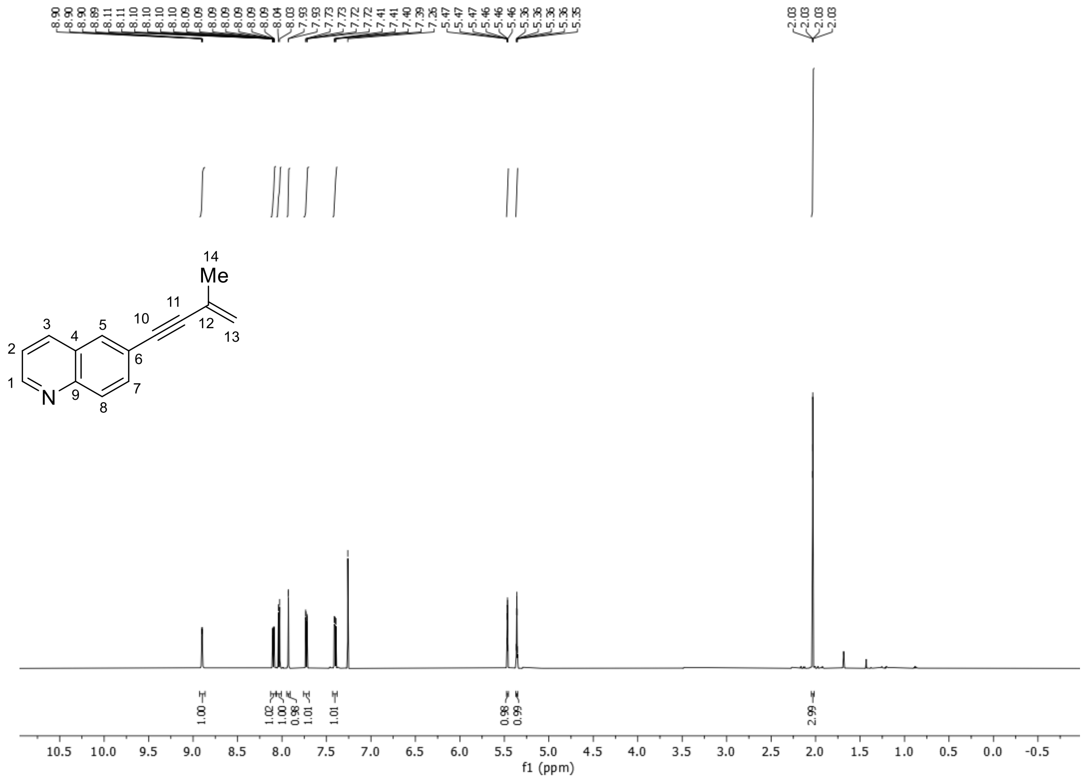

Supplementary Figure 29. ^{1}H NMR of **S12** (599 MHz, 299 K, $CDCl_3$).

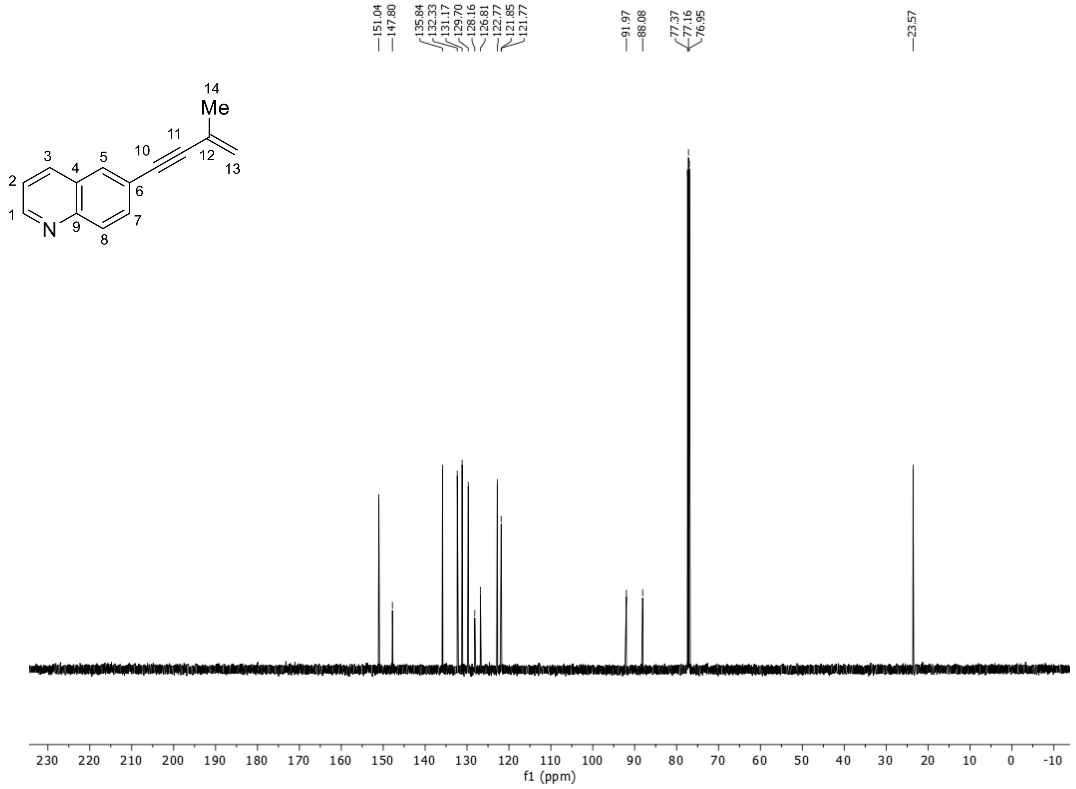

Supplementary Figure 30. $^{13}C\{^{1}H\}$ NMR of **S12** (151 MHz, 299 K, $CDCl_3$).

(4-(3-Methylbut-3-en-1-yn-1-yl)phenyl)(morpholino)methanone (S13)

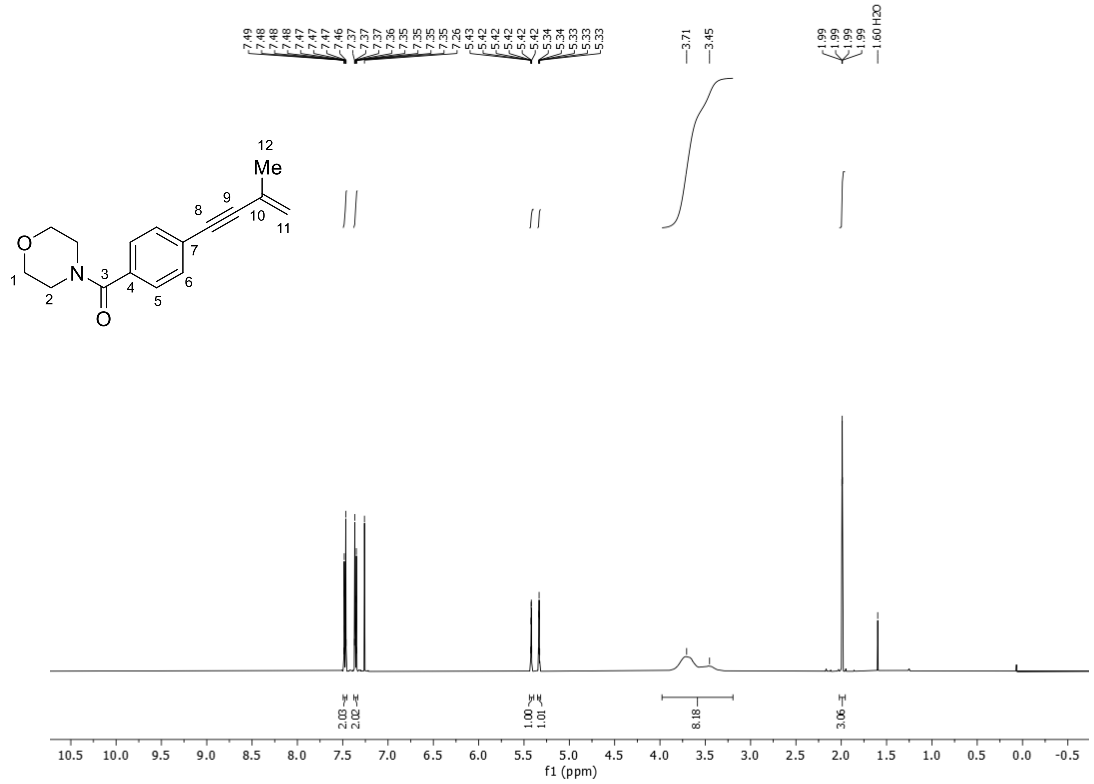

Supplementary Figure 31. ^{1}H NMR of **S13** (500 MHz, 299 K, $CDCl_3$).

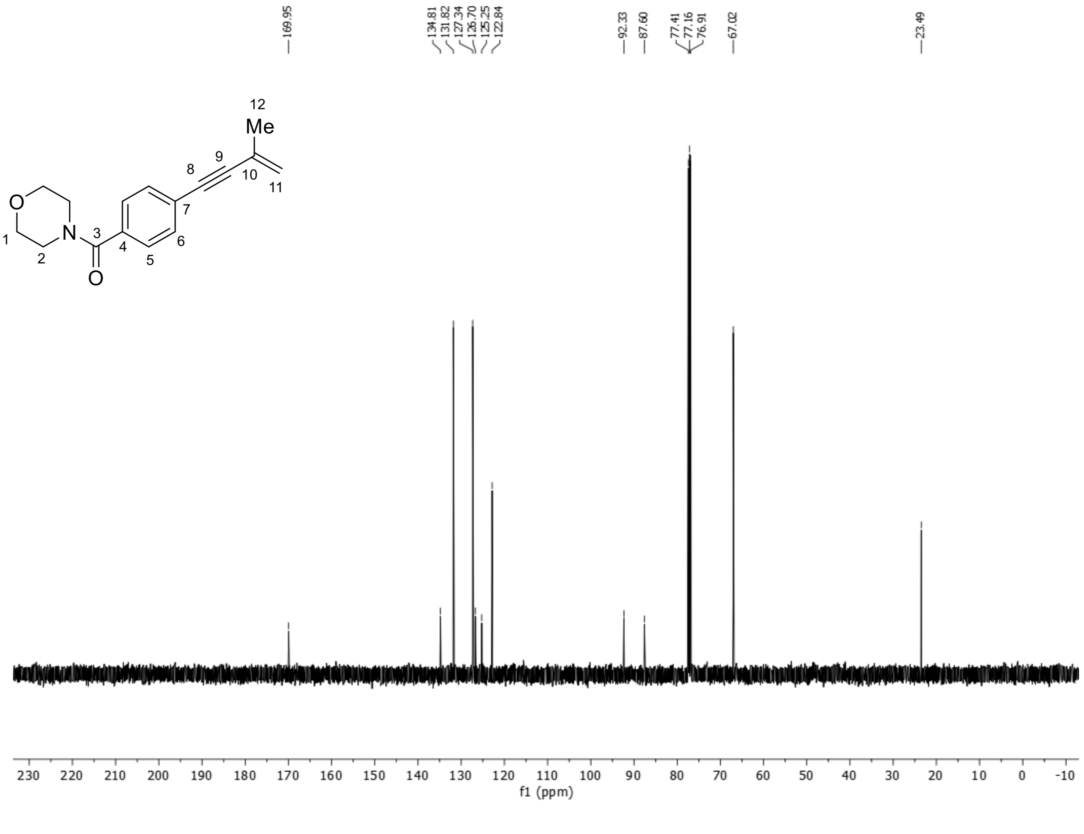

Supplementary Figure 32. $^{13}C\{^{1}H\}$ NMR of **S13** (126 MHz, 299 K, $CDCl_3$).

1-(3-Methylenepent-1-yn-1-yl)-4-nitrobenzene (S14)

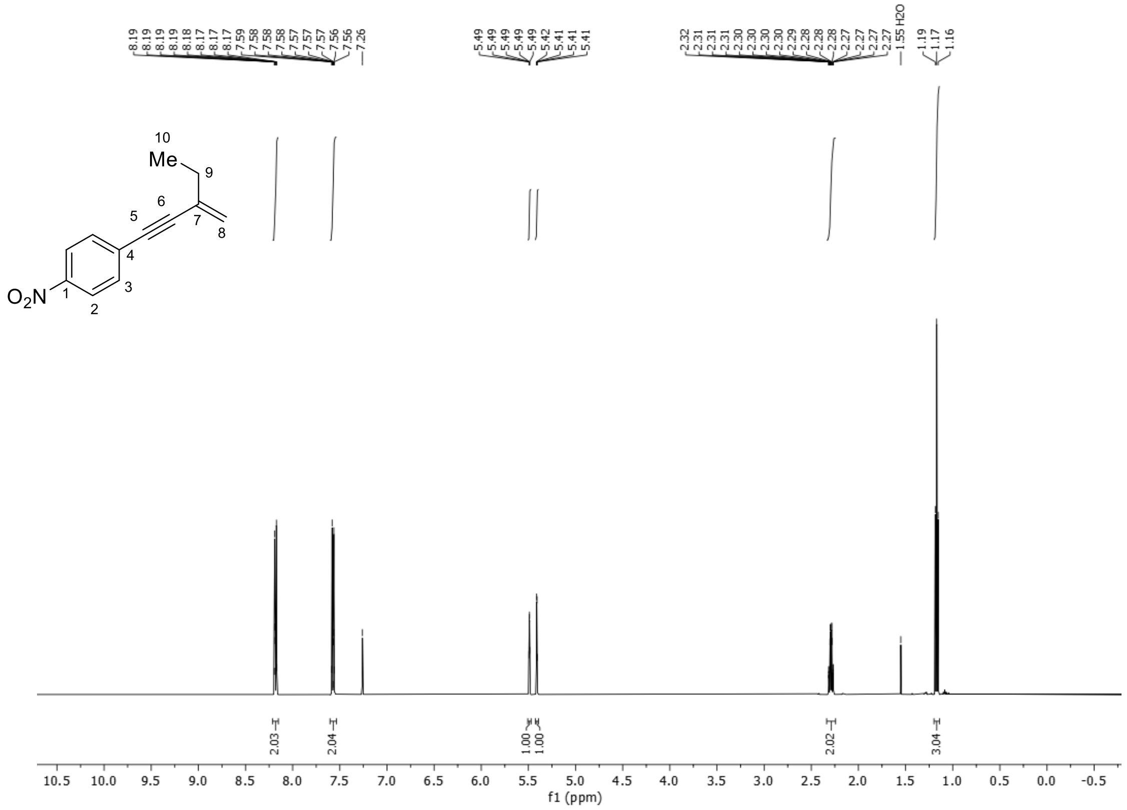

Supplementary Figure 33. ^{1}H NMR of **S14** (500 MHz, 299 K, $CDCl_3$).

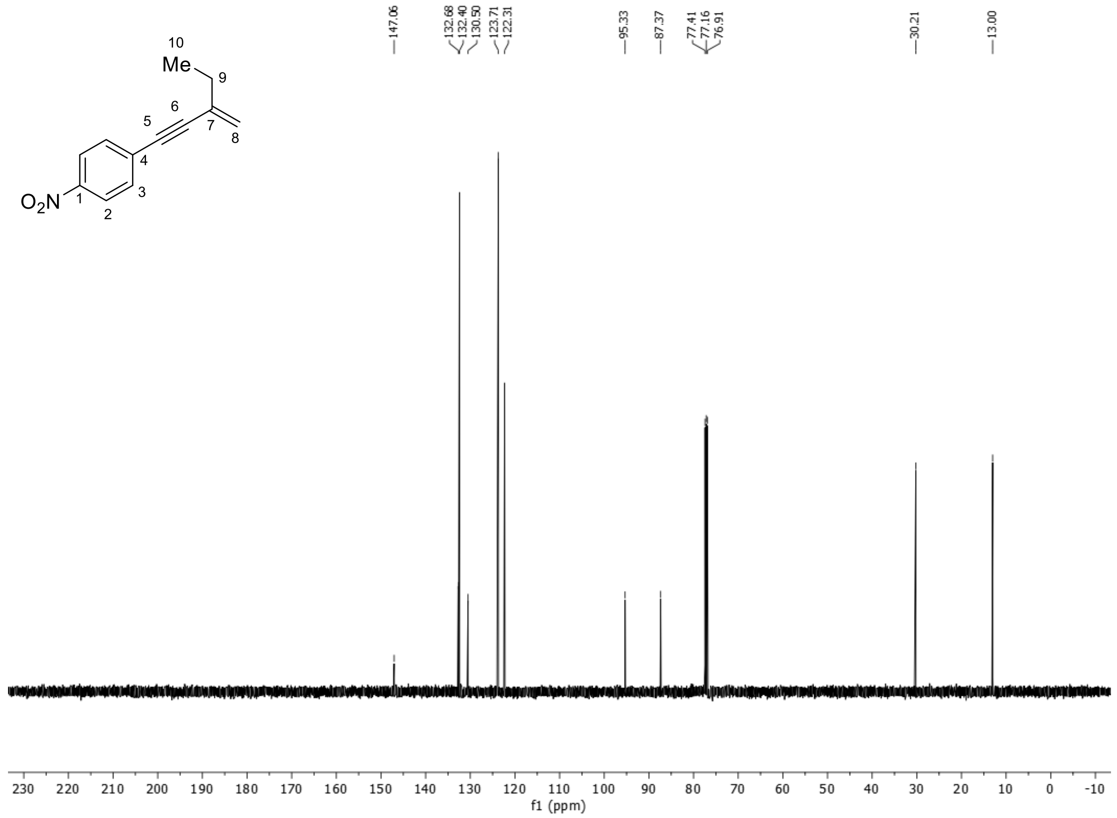

Supplementary Figure 34. $^{13}C\{^{1}H\}$ NMR of **S14** (126 MHz, 299 K, $CDCl_3$).

2-Methylene-4-(4-nitrophenyl)but-3-yn-1-ol (Int 1)

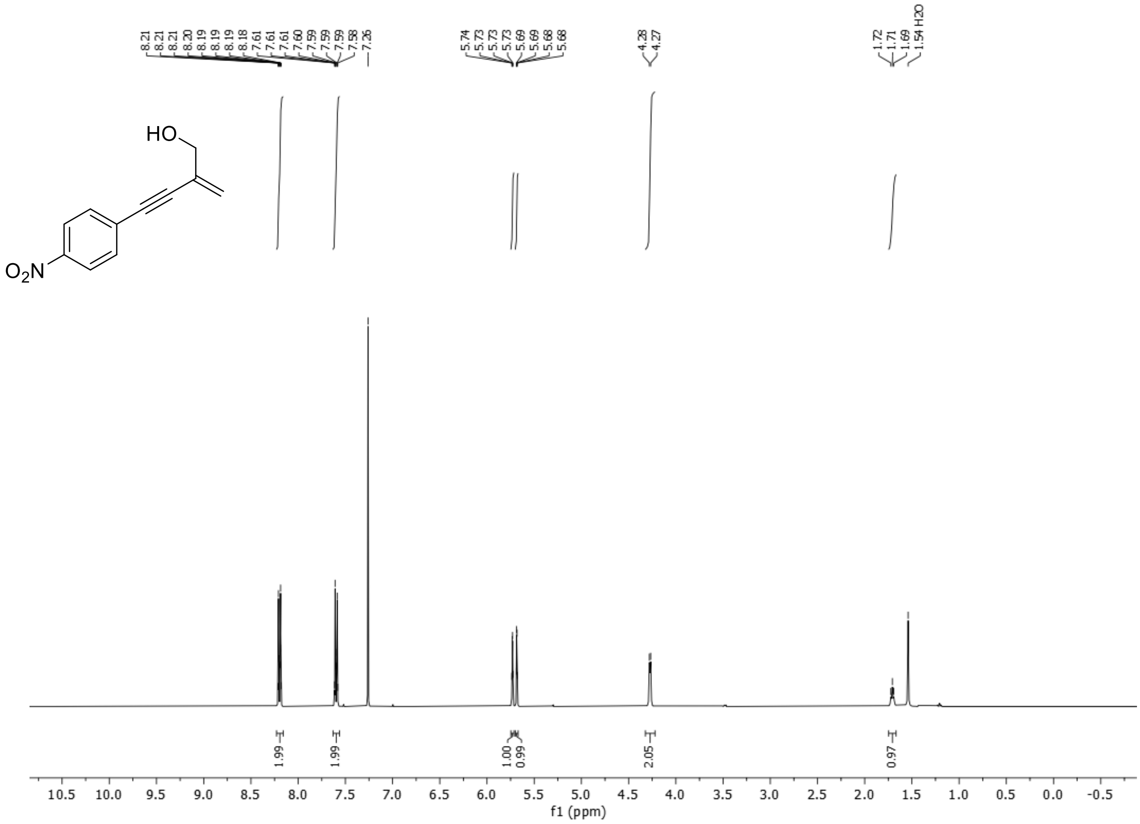

Supplementary Figure 35. ^{1}H NMR of **Int 1** (400 MHz, 299 K, $CDCl_3$).

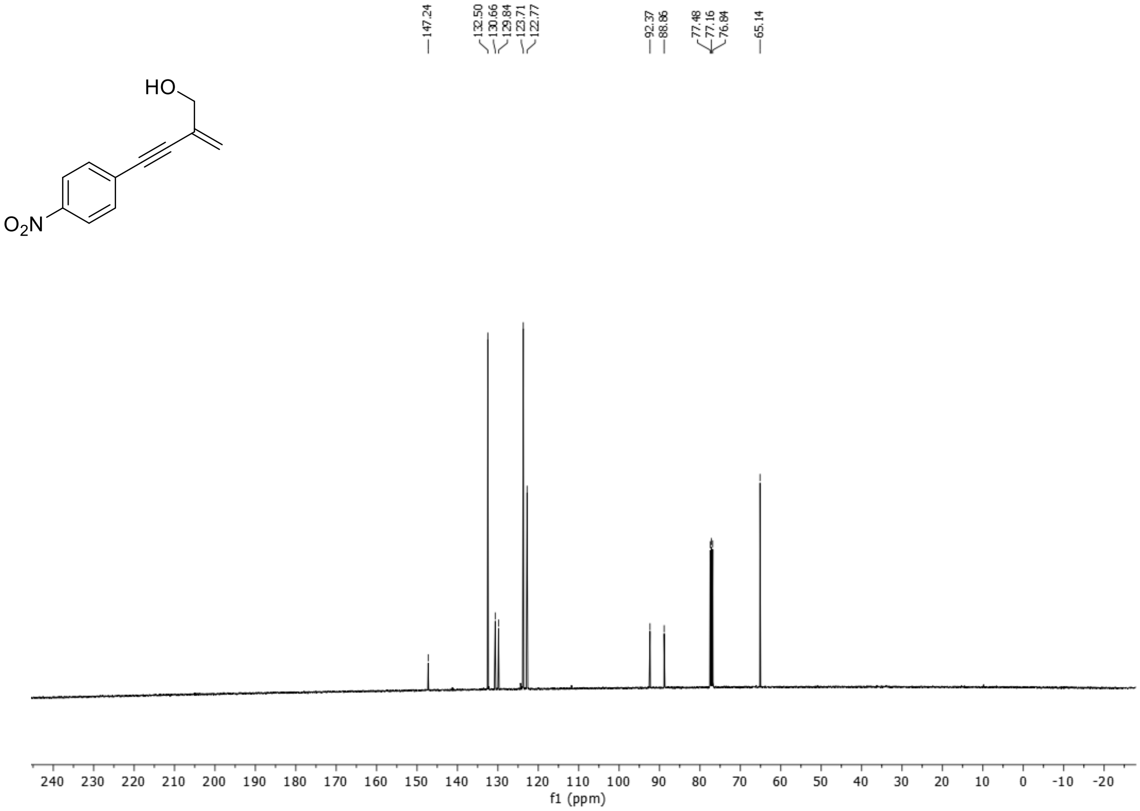

Supplementary Figure 36. $^{13}C\{^{1}H\}$ NMR of **Int 1** (101 MHz, 299 K, $CDCl_3$).

1-(3-(Bromomethyl)but-3-en-1-yn-1-yl)-4-nitrobenzene (S15)

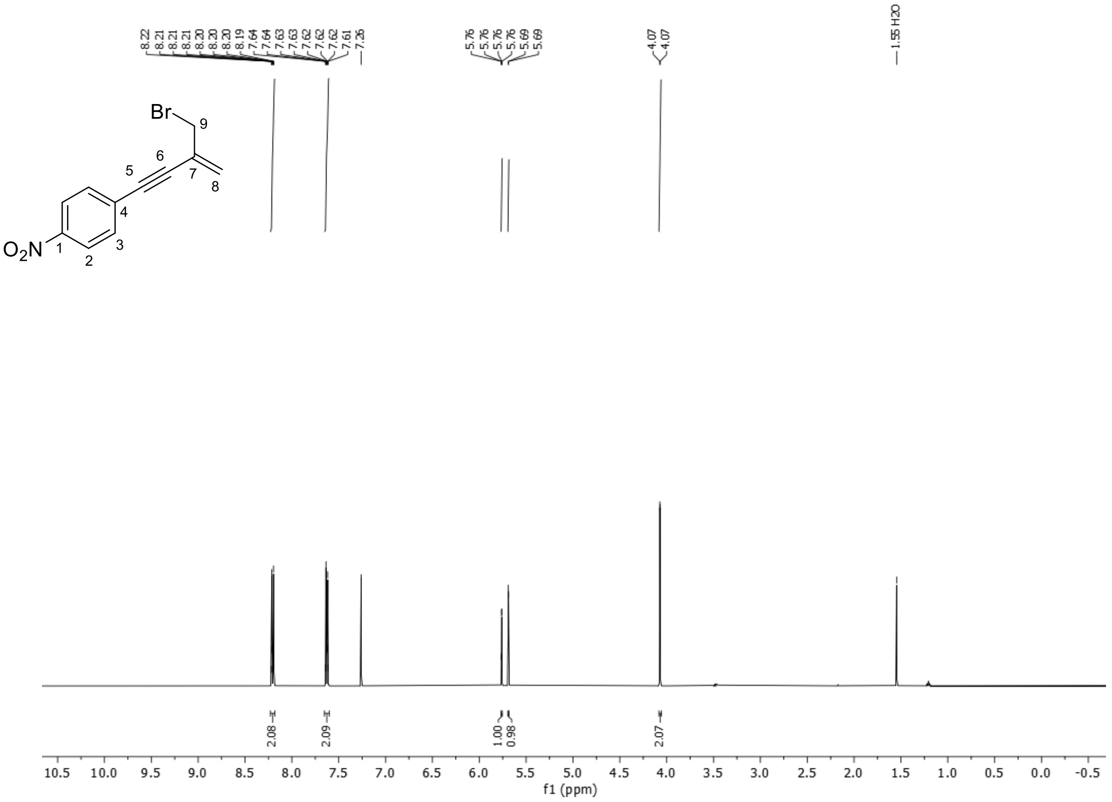

Supplementary Figure 37. ^{1}H NMR of **S15** (500 MHz, 299 K, $CDCl_3$).

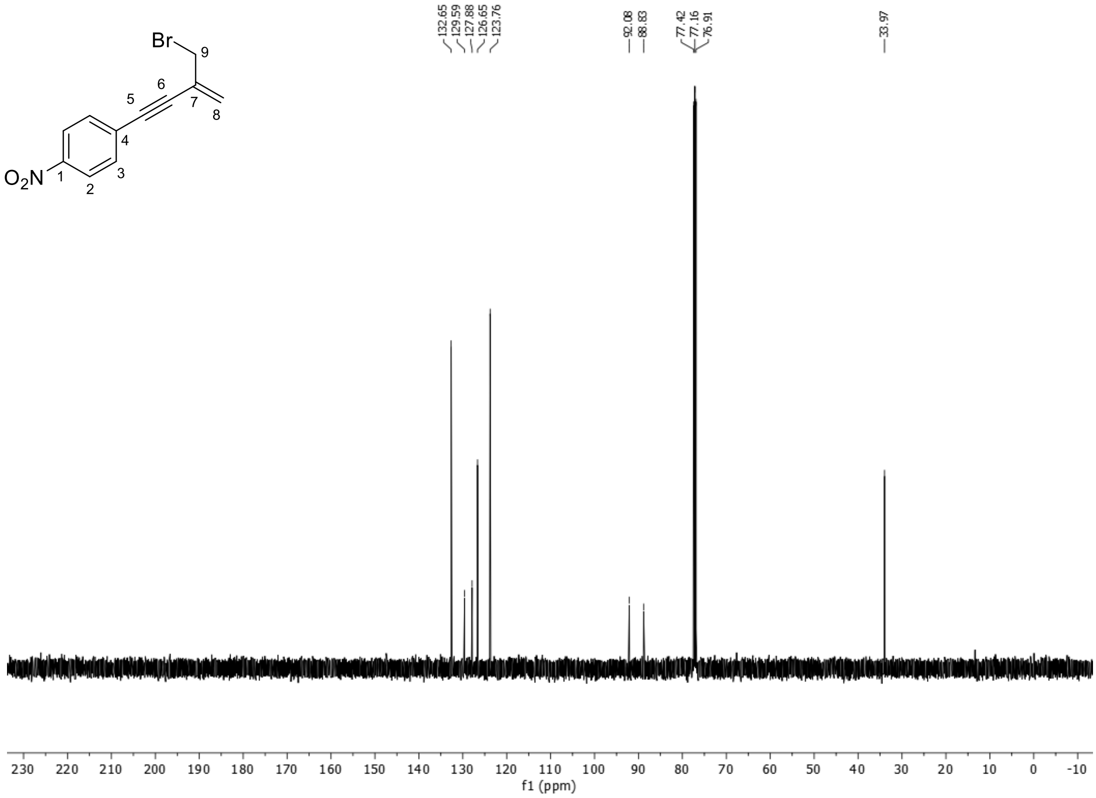

Supplementary Figure 38. $^{13}C\{^{1}H\}$ NMR of **S15** (126 MHz, 299 K, $CDCl_3$).

1-(3-(Chloromethyl)but-3-en-1-yn-1-yl)-4-nitrobenzene (S16)

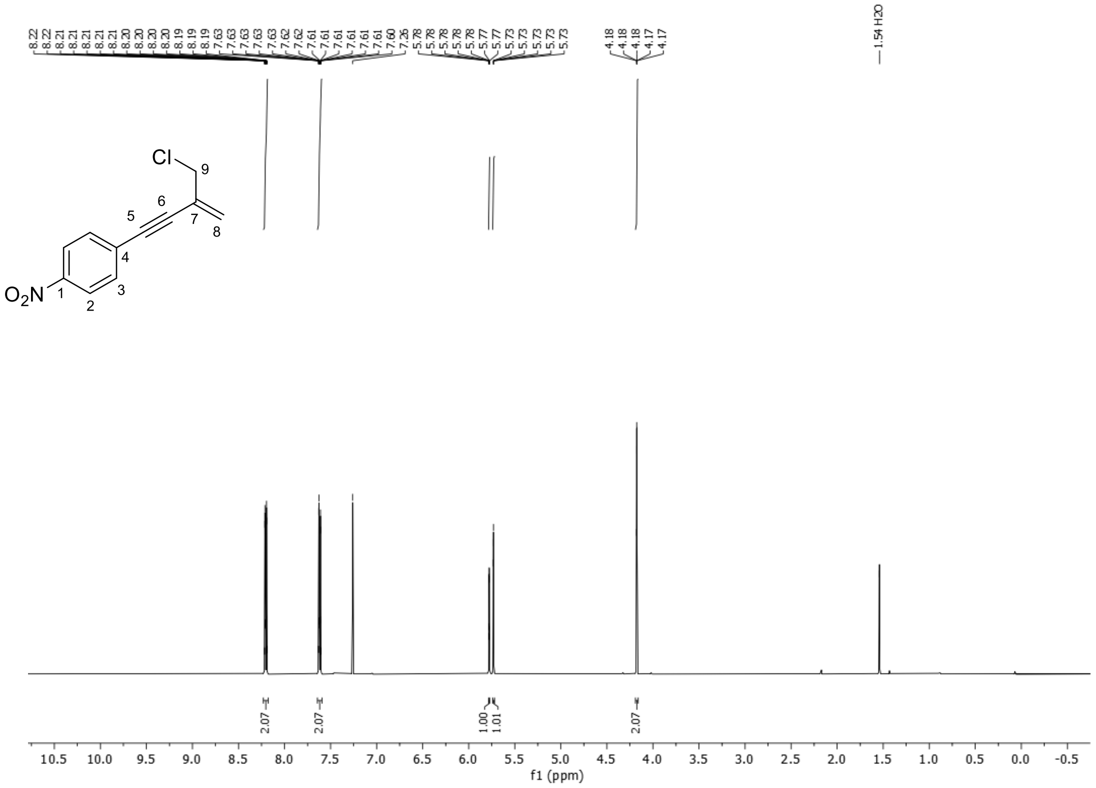

Supplementary Figure 39. ^{1}H NMR of **S16** (500 MHz, 299 K, $CDCl_3$).

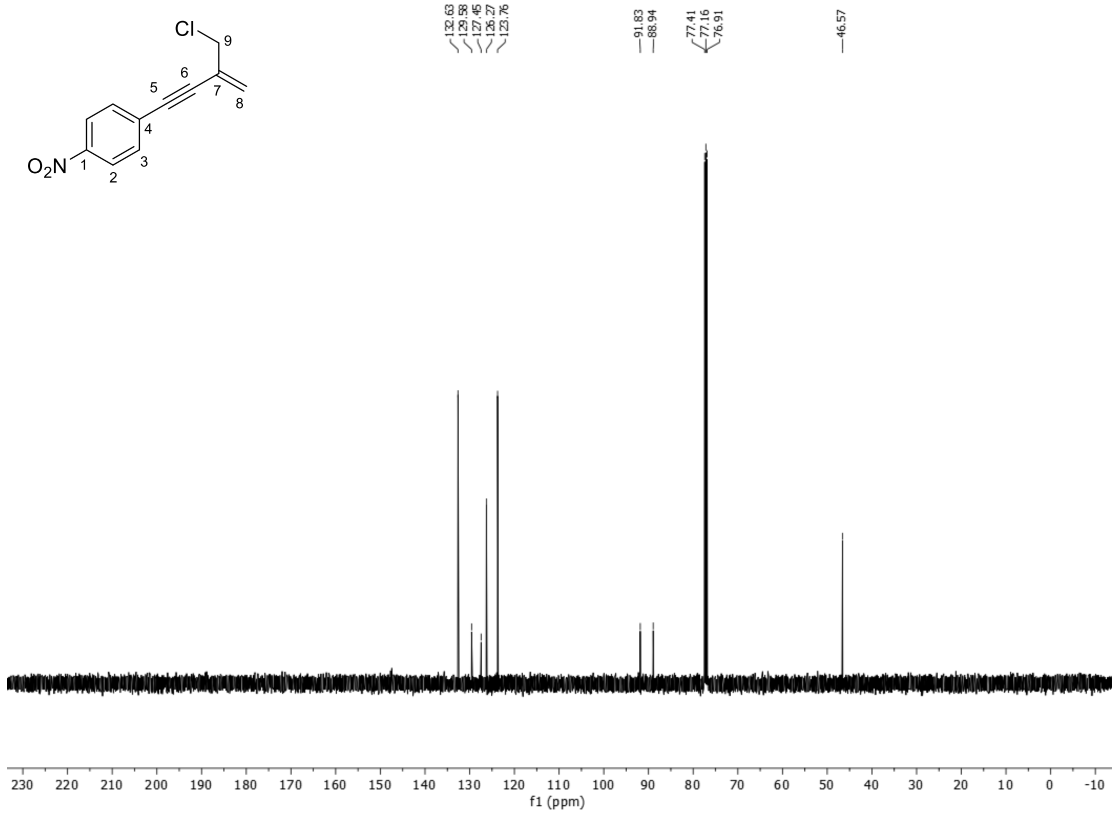

Supplementary Figure 40. $^{13}C\{^{1}H\}$ NMR of **S16** (126 MHz, 299 K, $CDCl_3$).

(1*R*,2*S*,5*R*)-2-Isopropyl-5-methylcyclohexyl 4-(3-methylbut-3-en-1-yn-1-yl)benzoate (S17)

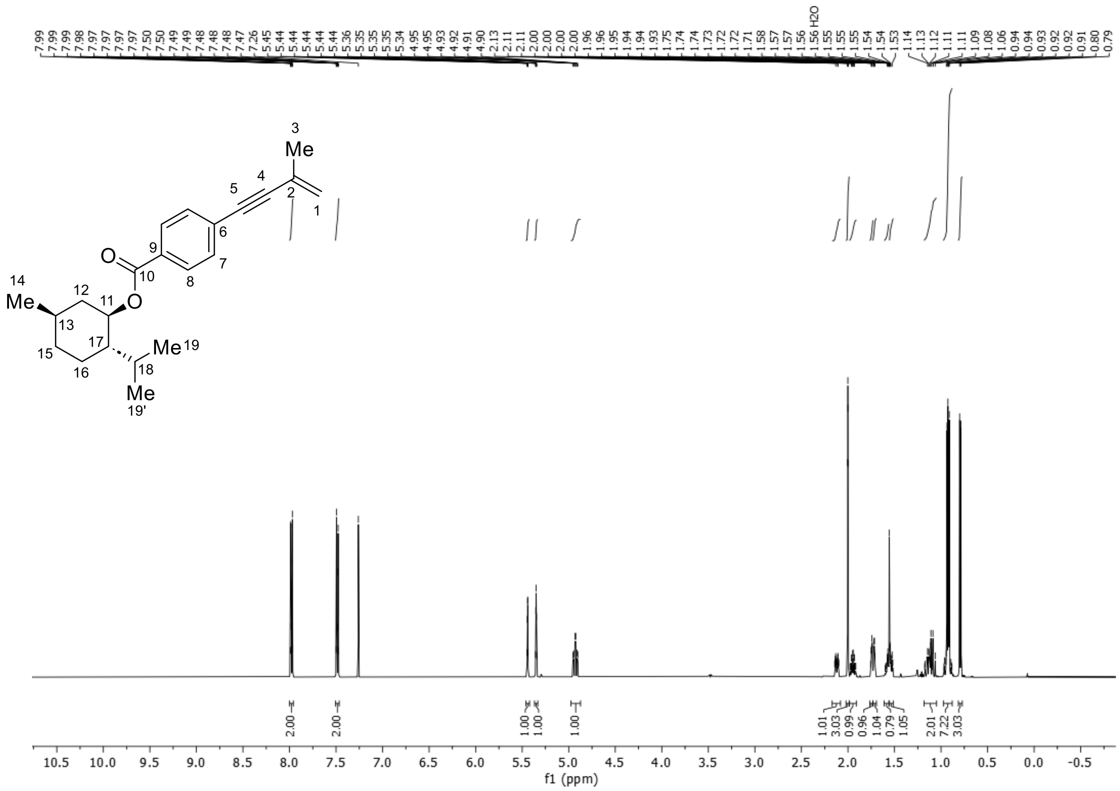

Supplementary Figure 41. ^{1}H NMR of **S17** (500 MHz, 299 K, $CDCl_3$).

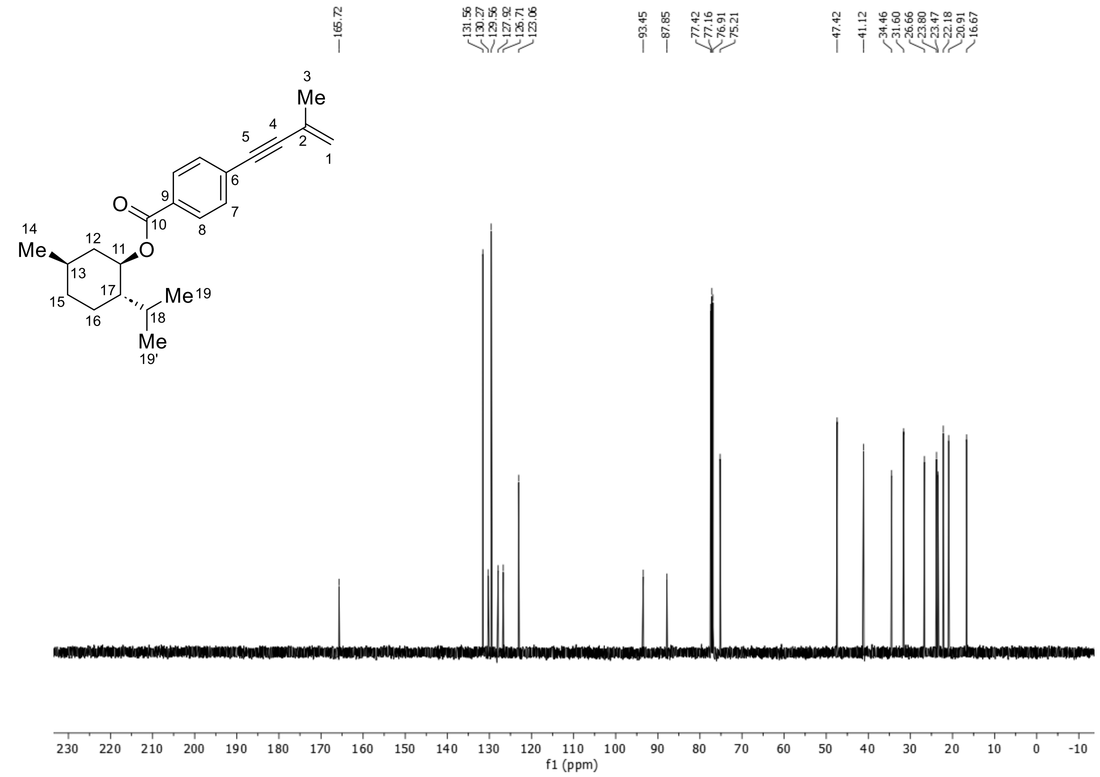

Supplementary Figure 42. $^{13}C\{^{1}H\}$ NMR of **S17** (126 MHz, 299 K, $CDCl_3$).

(3*S*,5*S*,8*R*,9*S*,10*S*,13*R*,14*S*,17*R*)-10,13-Dimethyl-17-((*R*)-6-methylheptan-2-yl)hexadecahydro-1H-cyclopenta[a]phenanthren-3-yl 4-(3-methylbut-3-en-1-yn-1-yl)benzoate (S18)

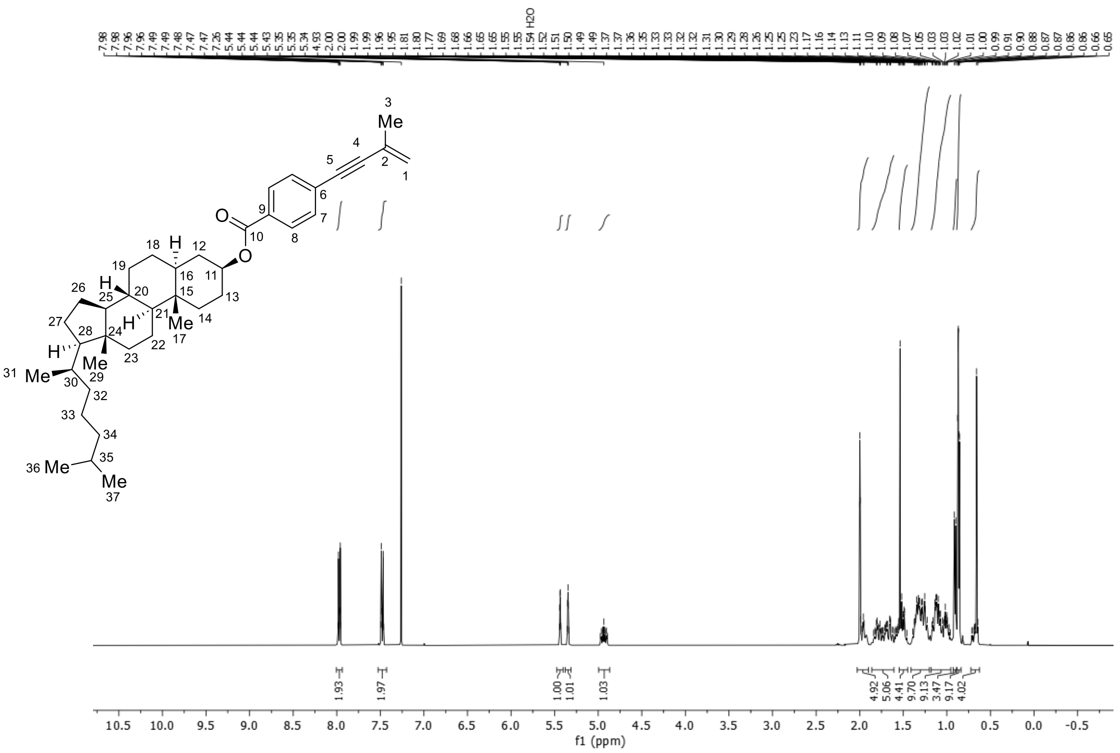

Supplementary Figure 43. ^{1}H NMR of **S18** (400 MHz, 299 K, $CDCl_3$).

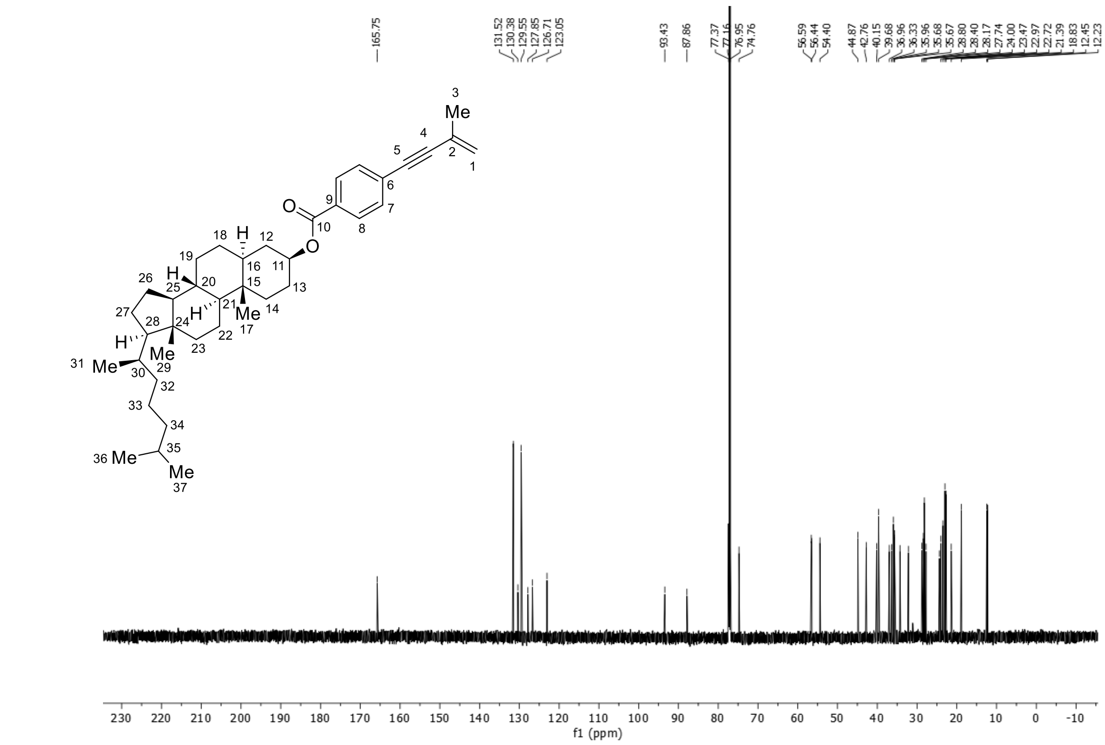

Supplementary Figure 44. $^{13}C\{^{1}H\}$ NMR of **S18** (151 MHz, 299 K, $CDCl_3$).

(8*R*,9*S*,13*S*)-13-Methyl-17-oxo-7,8,9,11,12,13,14,15,16,17-decahydro-6H-cyclopenta[a]phenanthren-3-yl 4-(3-methylbut-3-en-1-yn-1-yl)benzoate (S19)

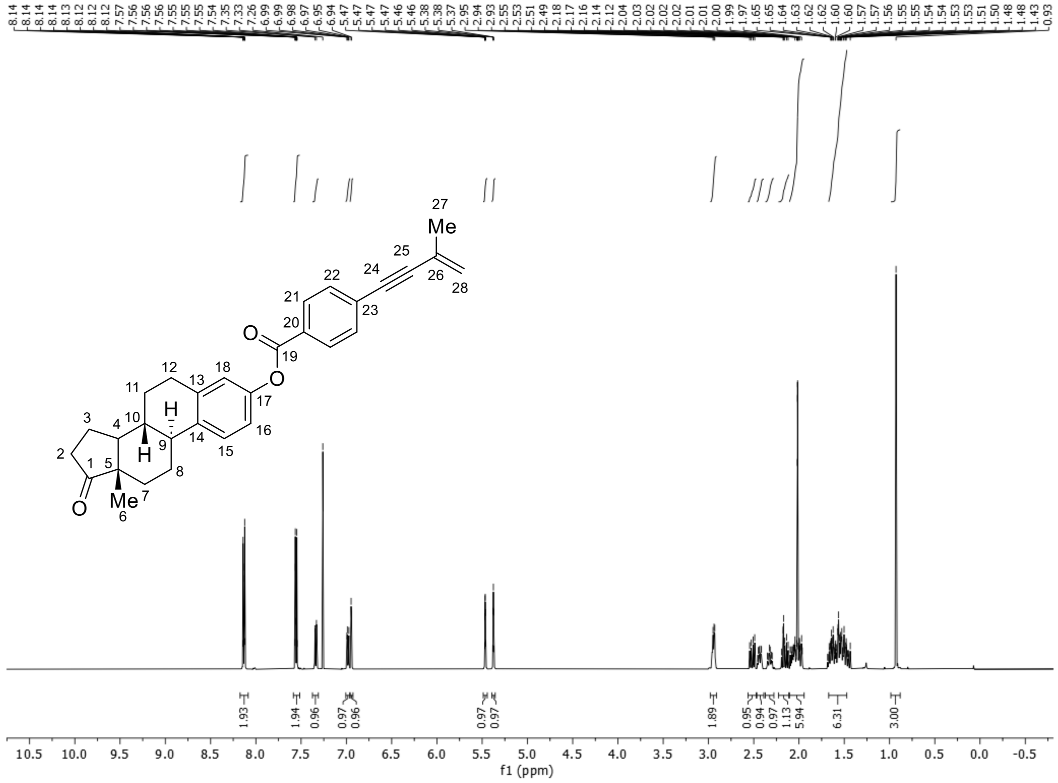

Supplementary Figure 45. ^{1}H NMR of **S19** (500 MHz, 299 K, $CDCl_3$).

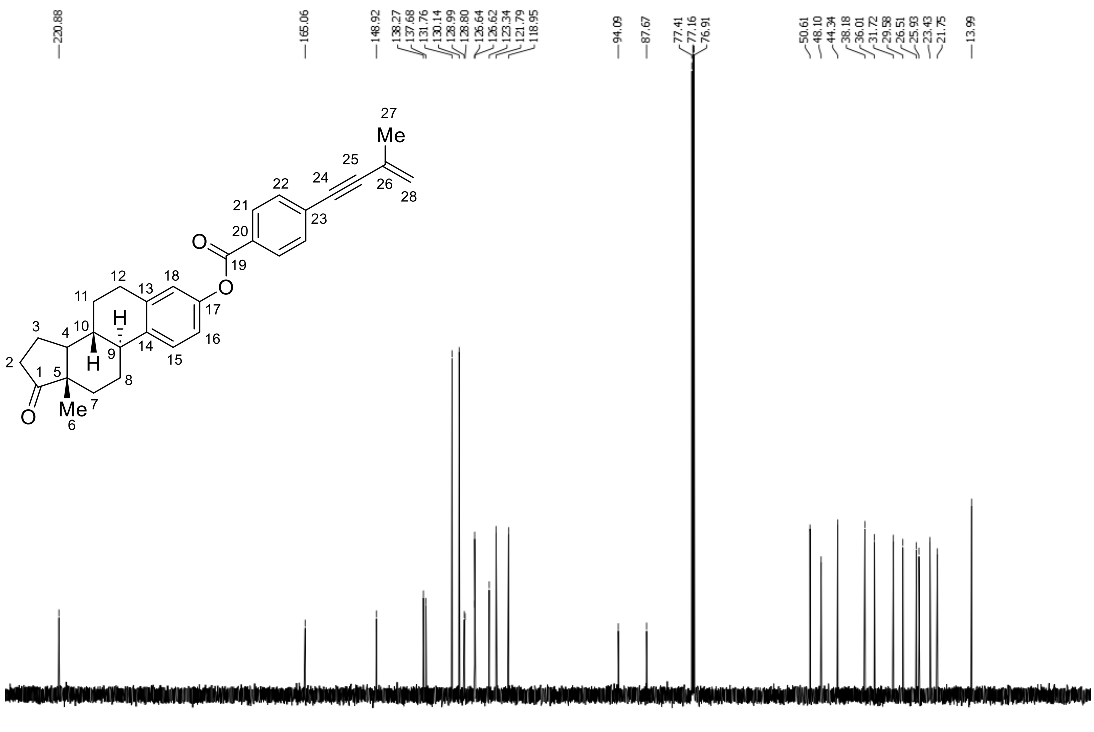

Supplementary Figure 46. $^{13}C\{^{1}H\}$ NMR of **S19** (126 MHz, 299 K, $CDCl_3$).

2-Methylhexadec-1-en-3-yne (S20)

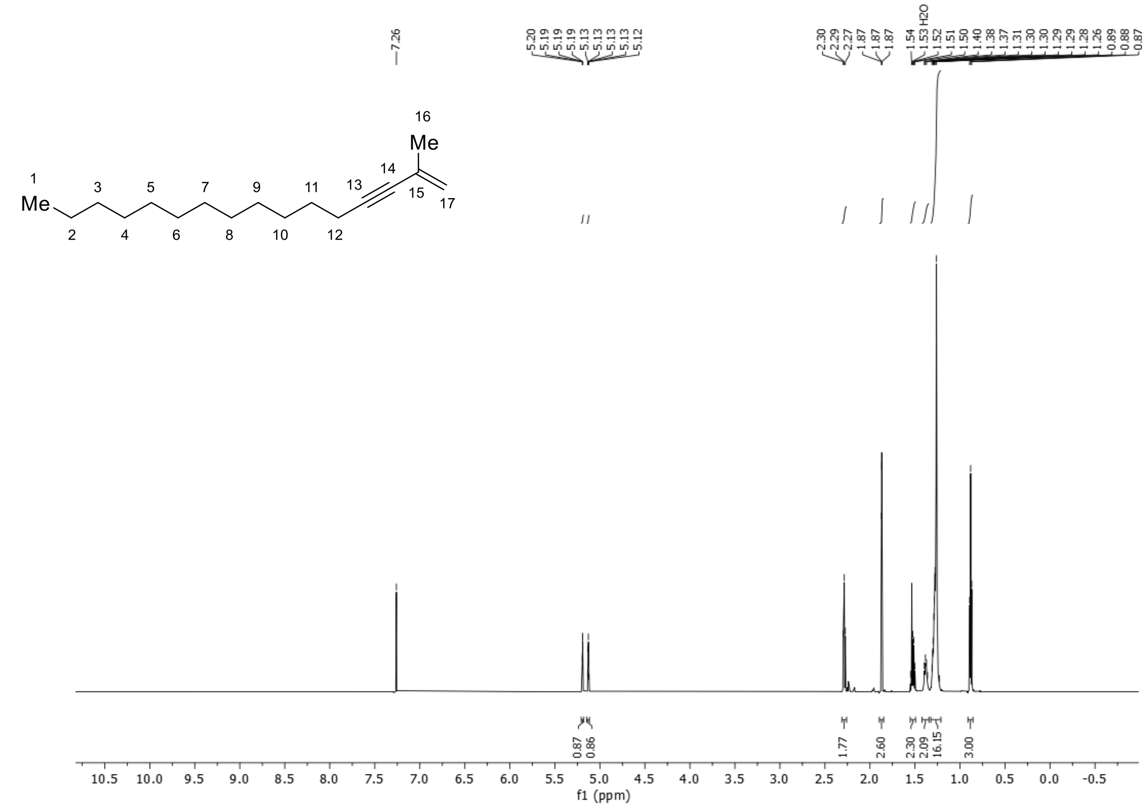

Supplementary Figure 47. ^{1}H NMR of **S20** (599 MHz, 299 K, $CDCl_3$).

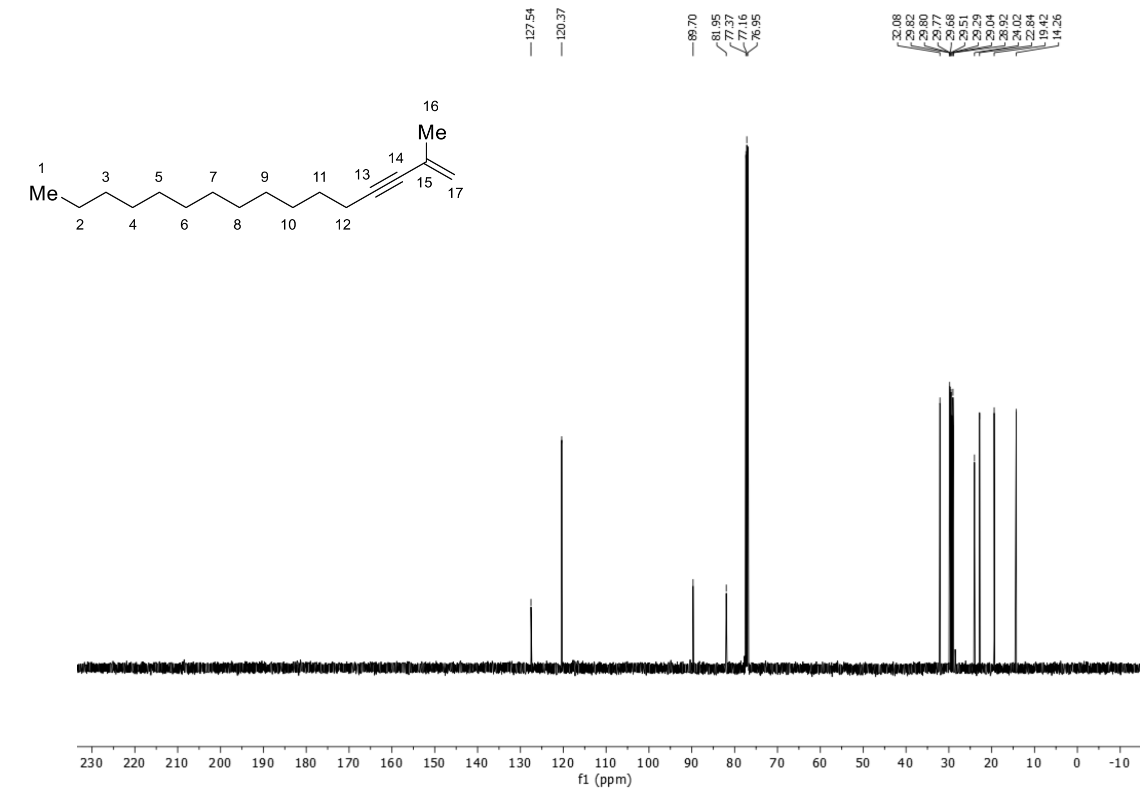

Supplementary Figure 48. $^{13}C\{^{1}H\}$ NMR of **S20** (151 MHz, 299 K, $CDCl_3$).

4-Methylpent-4-en-2-yn-1-yl)cyclohexane (S21)

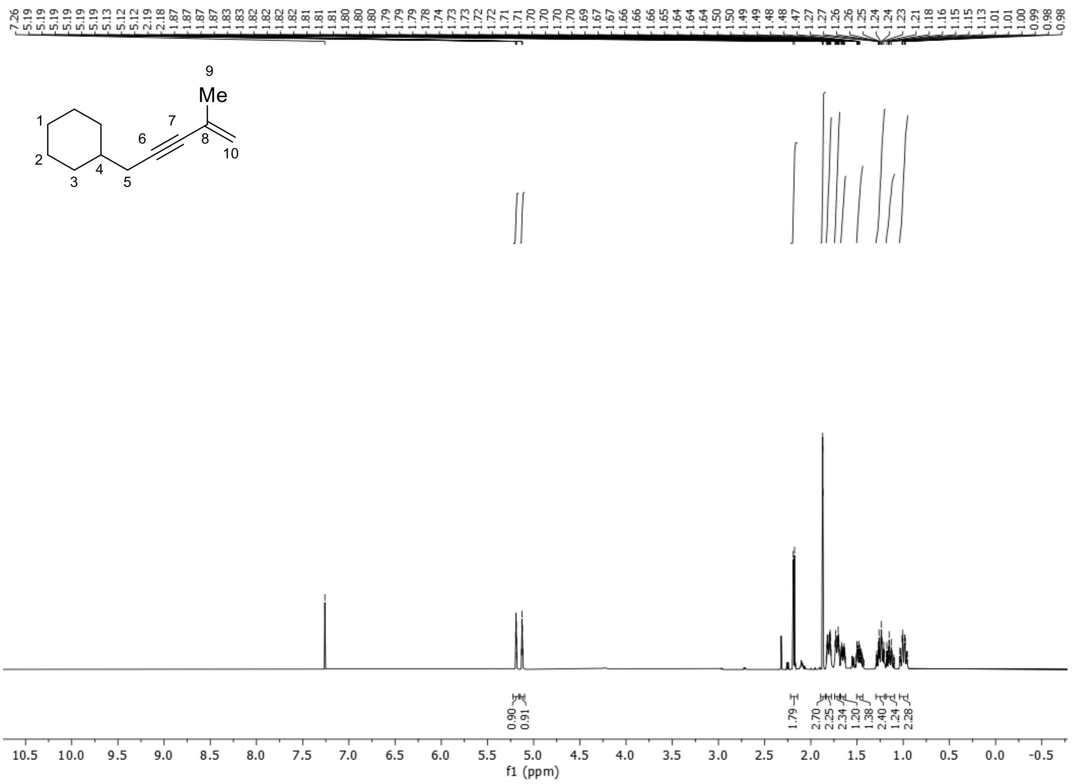

Supplementary Figure 49. ^{1}H NMR of **S21** (500 MHz, 299 K, $CDCl_3$).

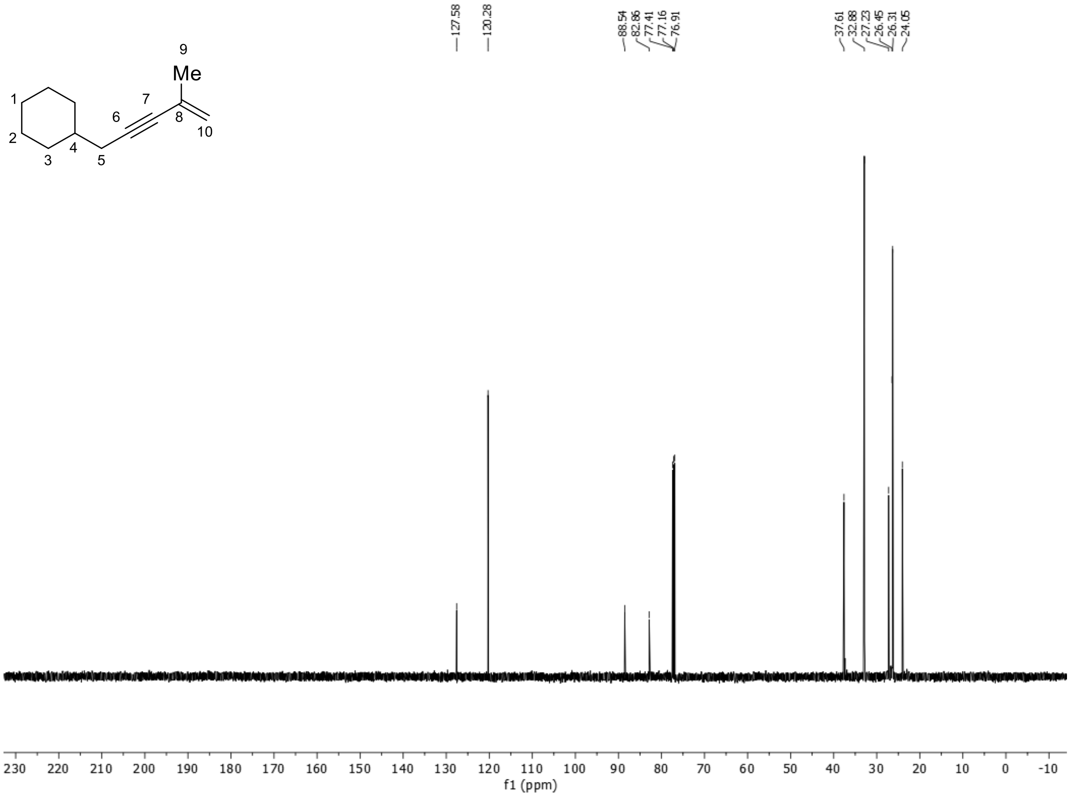

Supplementary Figure 50. $^{13}C\{^{1}H\}$ NMR of **S21** (126 MHz, 299 K, $CDCl_3$).

2-(6-Methylhept-6-en-4-yn-1-yl)isoindoline-1,3-dione (S22)

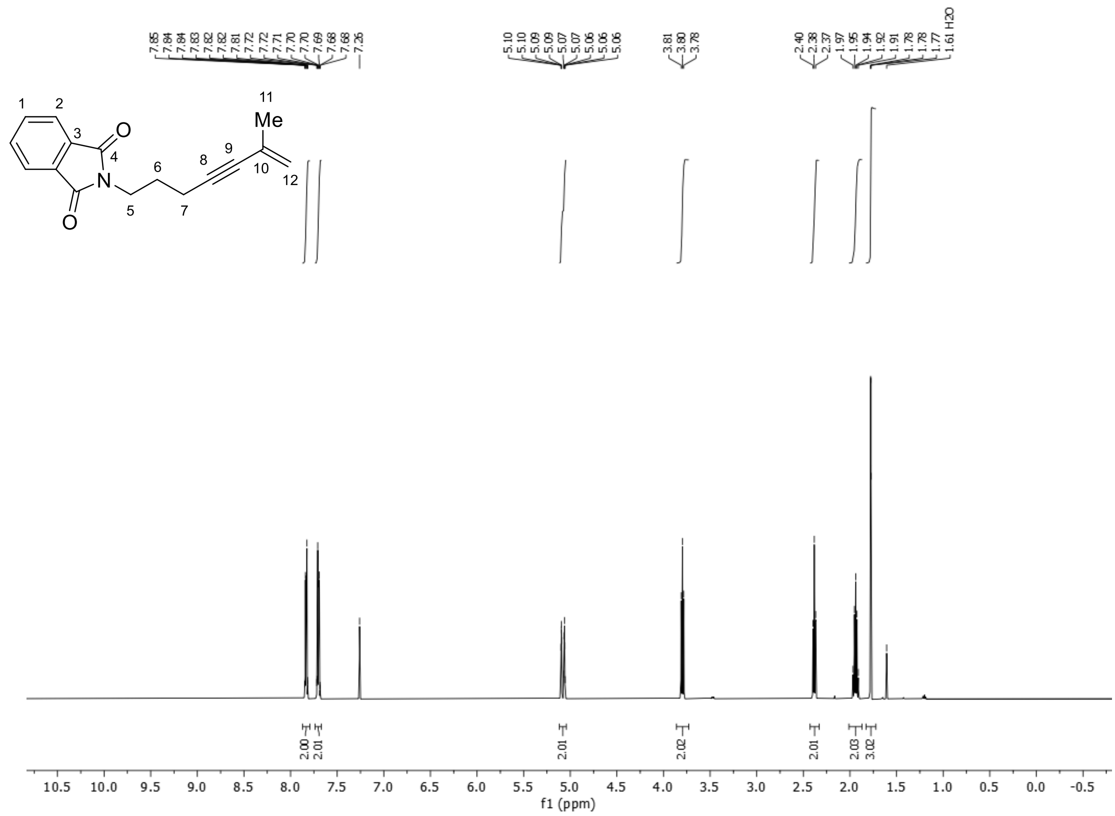

Supplementary Figure 51. ^{1}H NMR of **S22** (500 MHz, 299 K, $CDCl_3$).

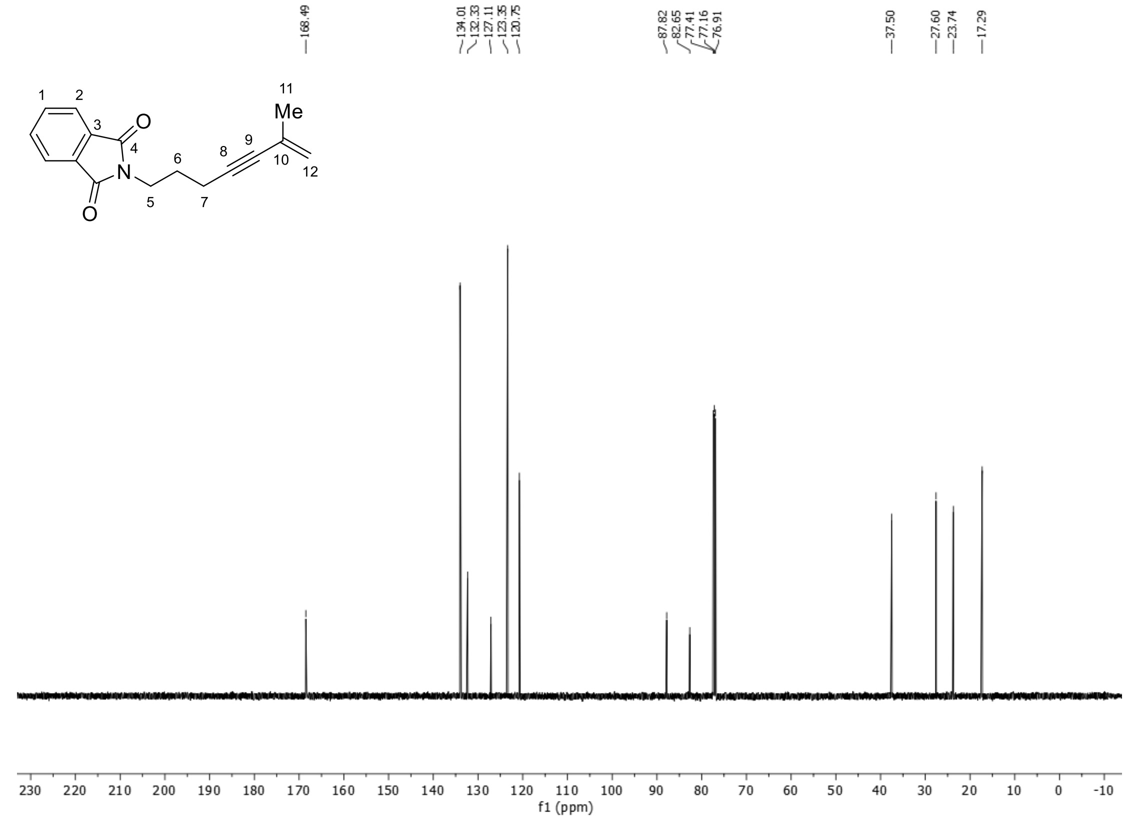

Supplementary Figure 52. $^{13}C\{^{1}H\}$ NMR of **S22** (126 MHz, 299 K, $CDCl_3$).

1,2-Difluoro-4-(((13-methyltetradec-13-en-11-yn-1-yl)oxy)methyl)benzene (S23)

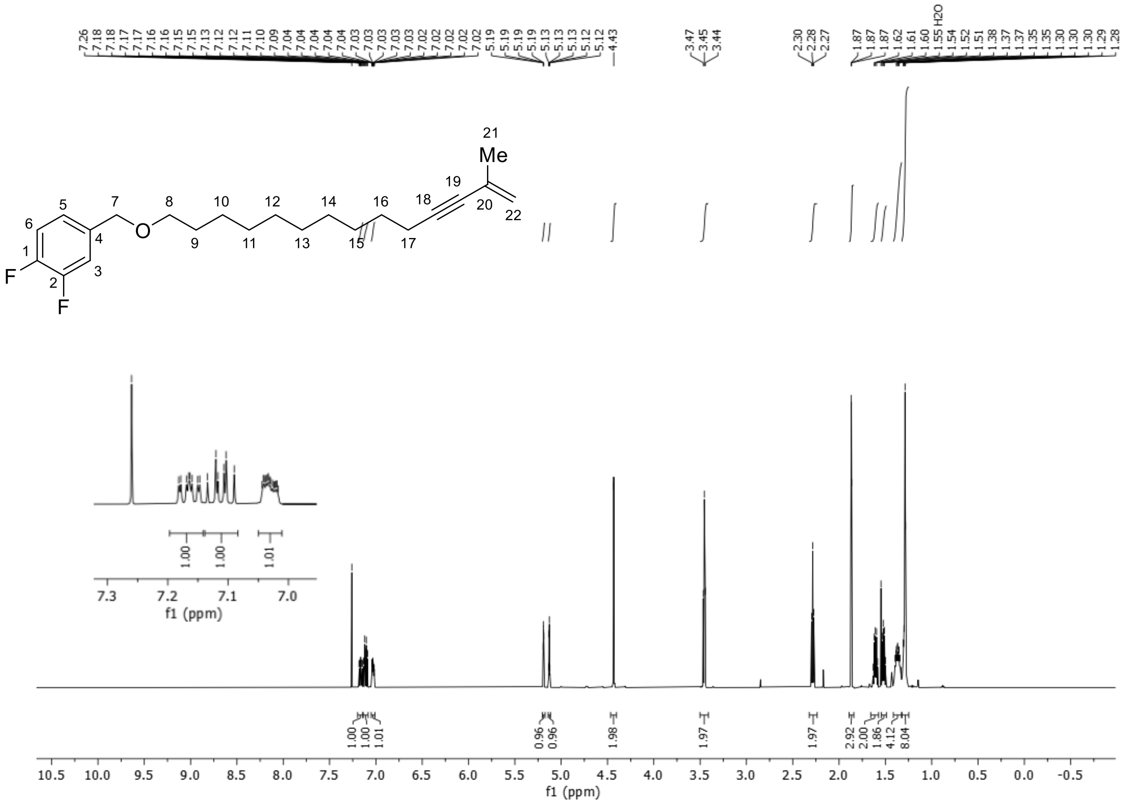

Supplementary Figure 53. ^{1}H NMR of **S23** (599 MHz, 299 K, $CDCl_3$).

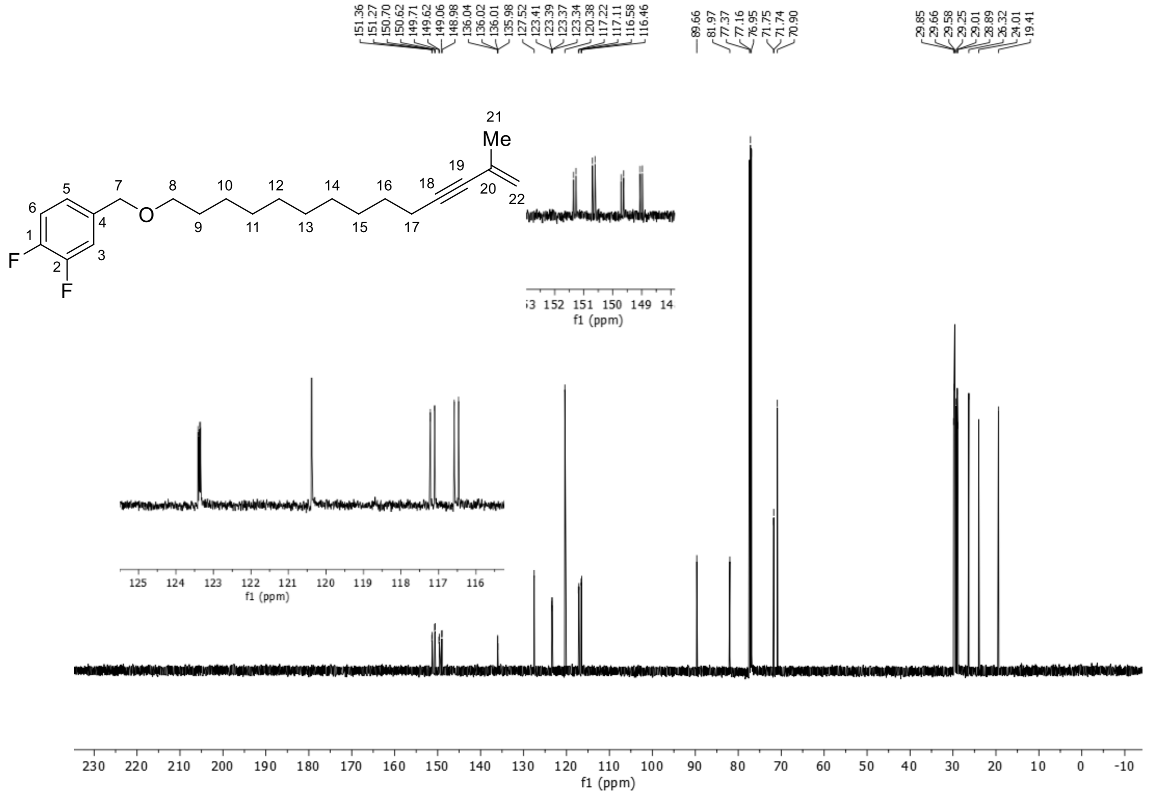

Supplementary Figure 54. $^{13}C\{^{1}H\}$ NMR of **S23** (151 MHz, 299 K, $CDCl_3$).

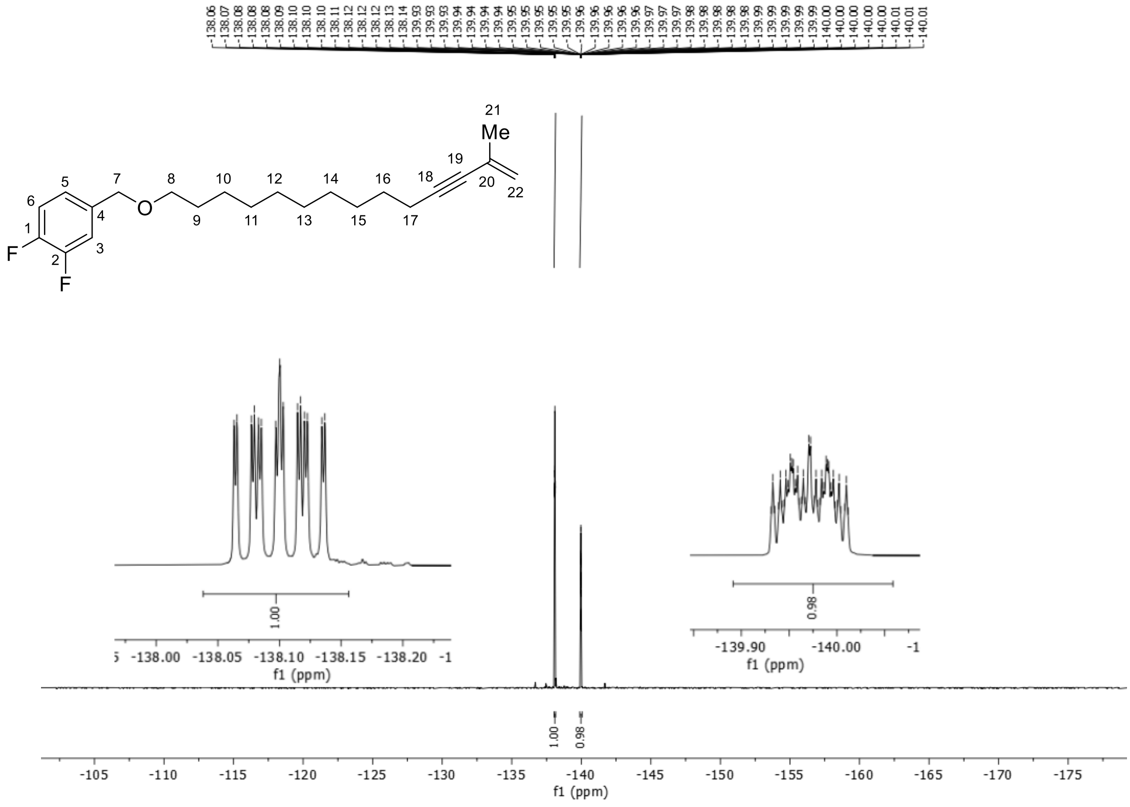

Supplementary Figure 55. ^{19}F NMR of **S23** (564 MHz, 299 K, $CDCl_3$).

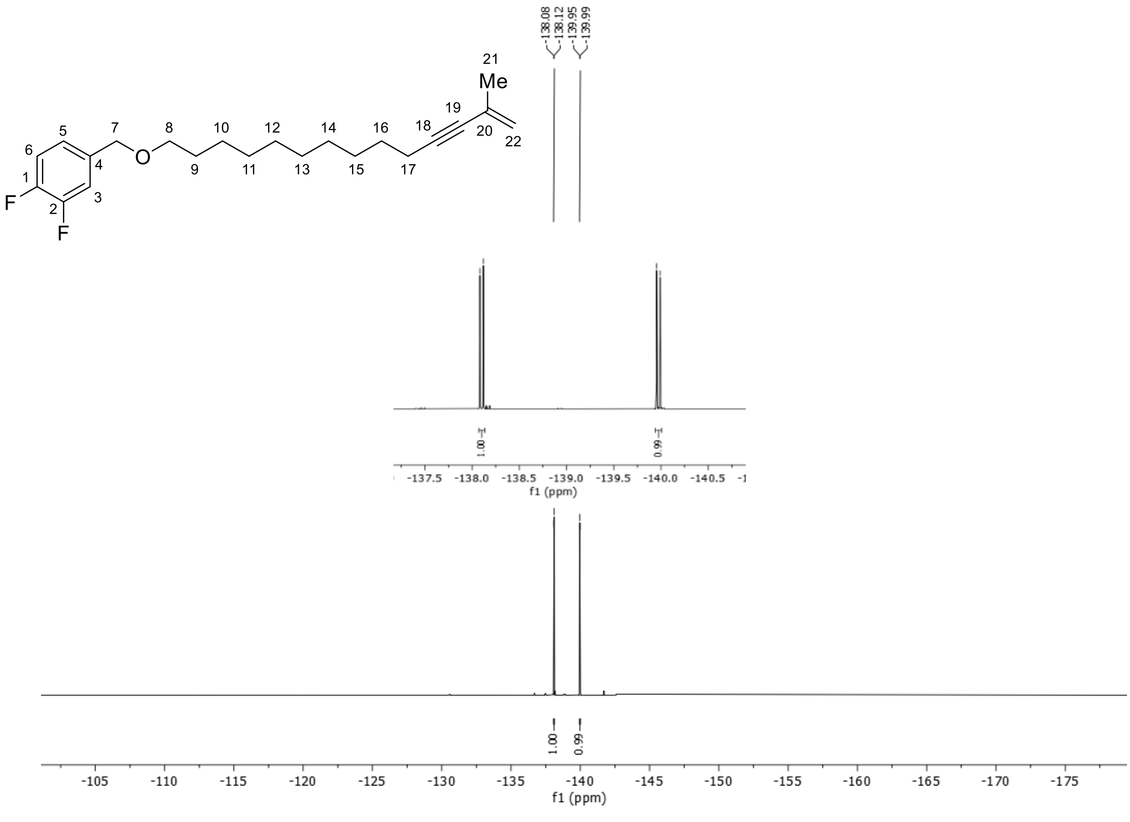

Supplementary Figure 56. $^{19}F\{^1H\}$ NMR of **S23** (564 MHz, 299 K, $CDCl_3$).

(4-Methylpent-4-en-2-yn-1-yl)benzene (S24)

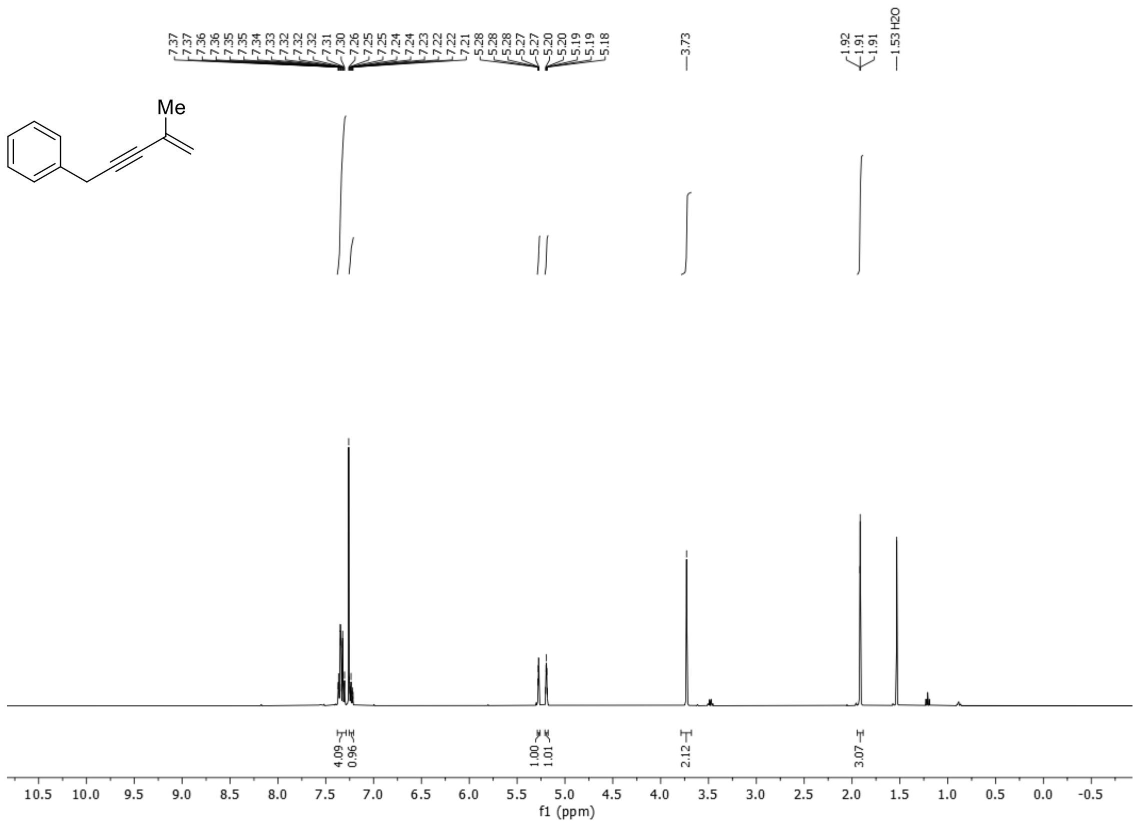

Supplementary Figure 57. ^{1}H NMR of **S24** (400 MHz, 299 K, $CDCl_3$).

13-Methyltetradec-13-en-11-yn-1-yl (*E*)-3-(4-bromophenyl)acrylate (S25)

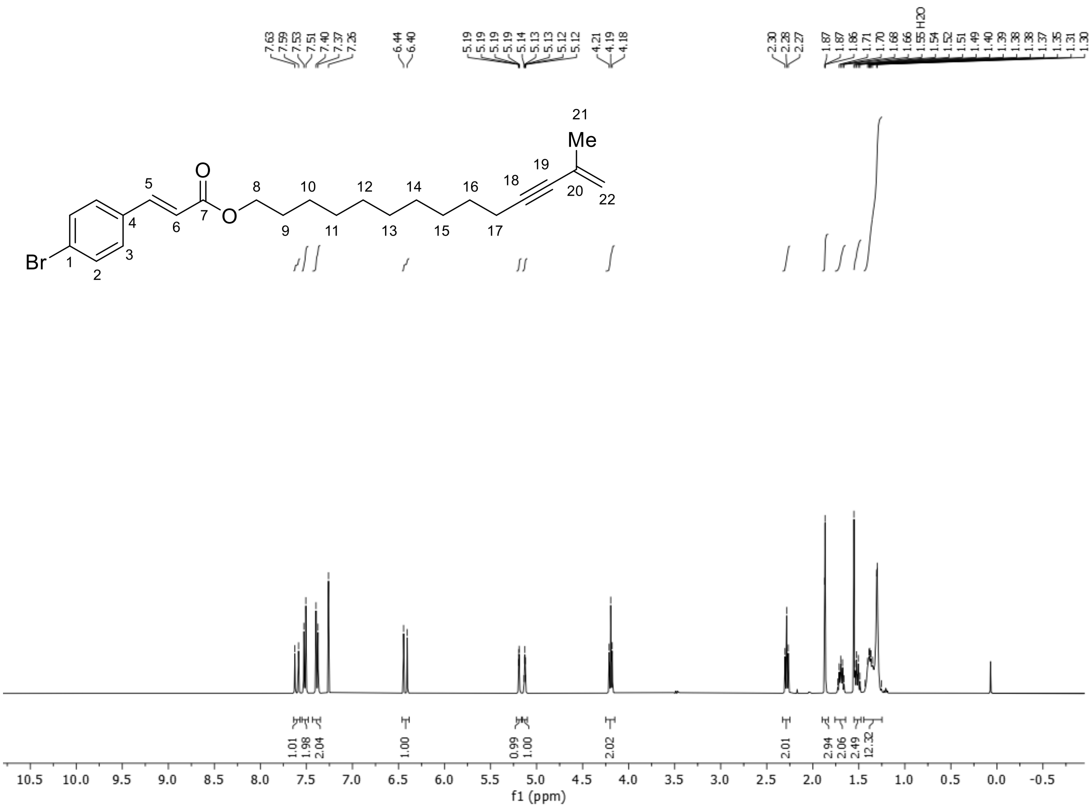

Supplementary Figure 58. ^{1}H NMR of **S25** (400 MHz, 299 K, $CDCl_3$).

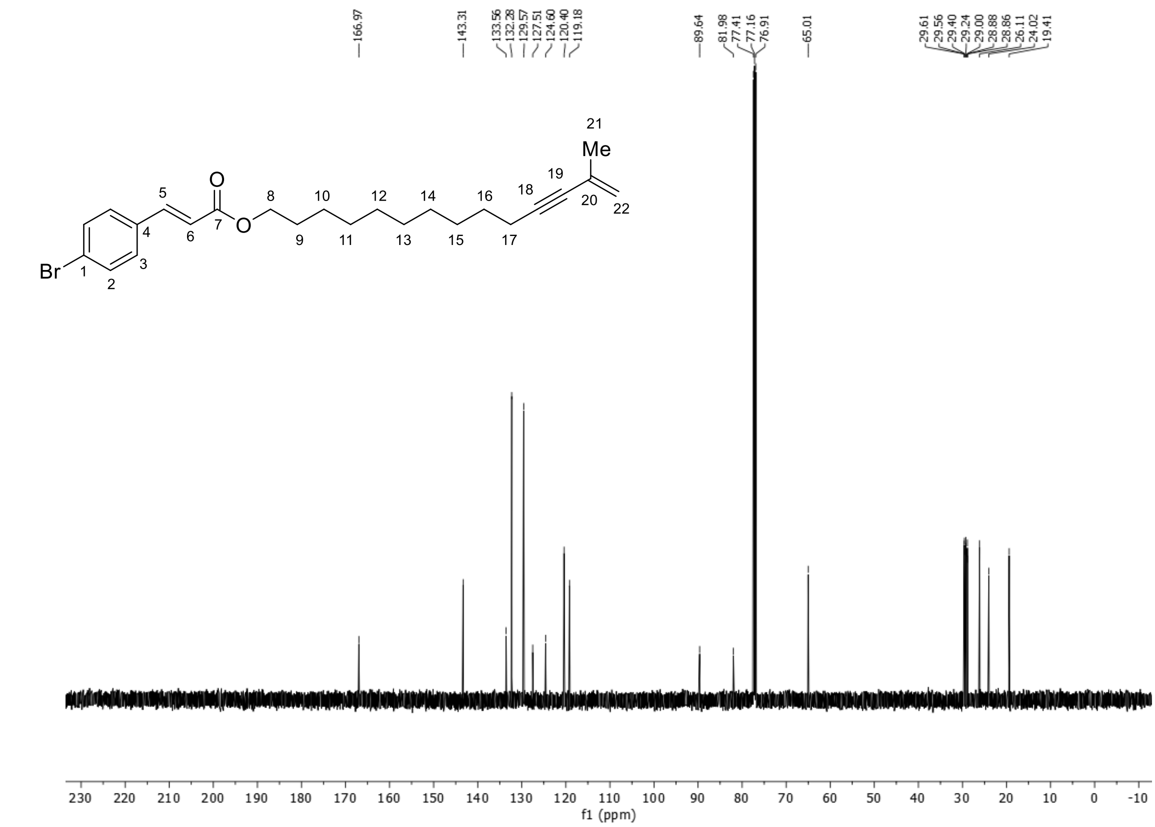

Supplementary Figure 59. $^{13}C\{^{1}H\}$ NMR of **S25** (126 MHz, 299 K, $CDCl_3$).

13-Methyltetradec-13-en-11-yn-1-yl hex-2-ynoate (S26)

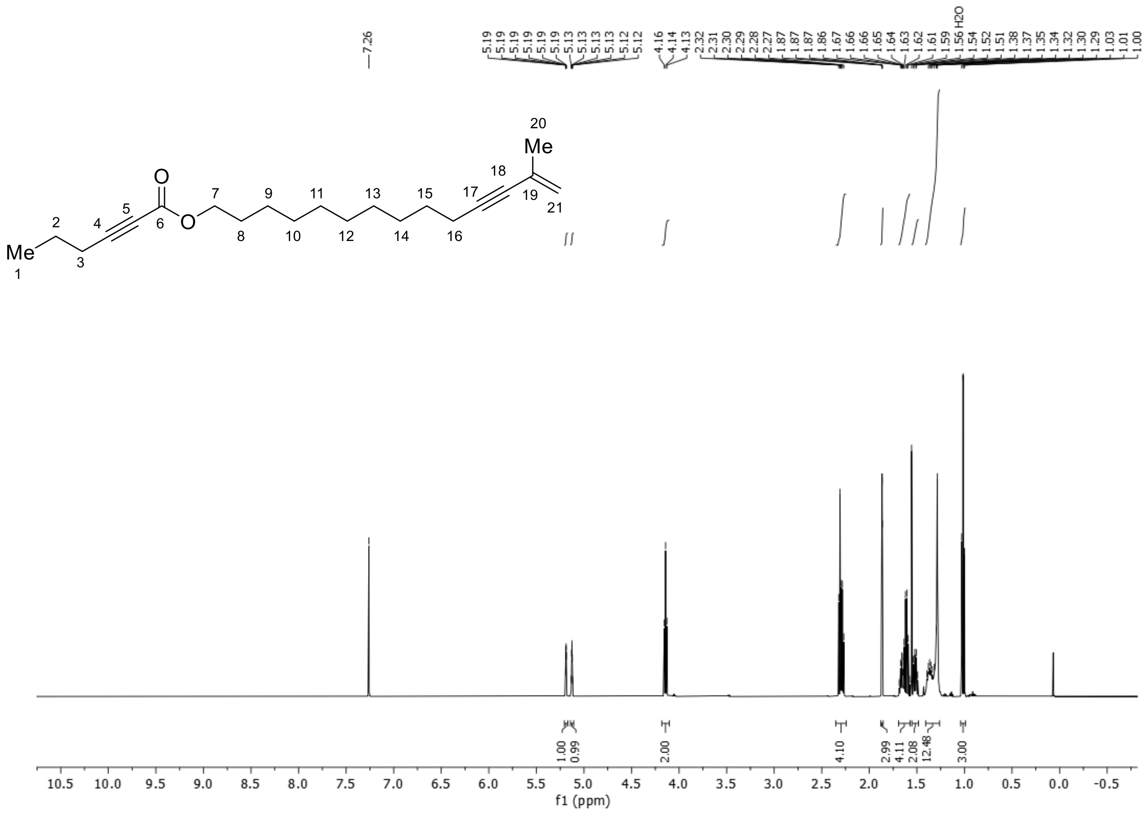

Supplementary Figure 60. ^{1}H NMR of **S26** (500 MHz, 299 K, $CDCl_3$).

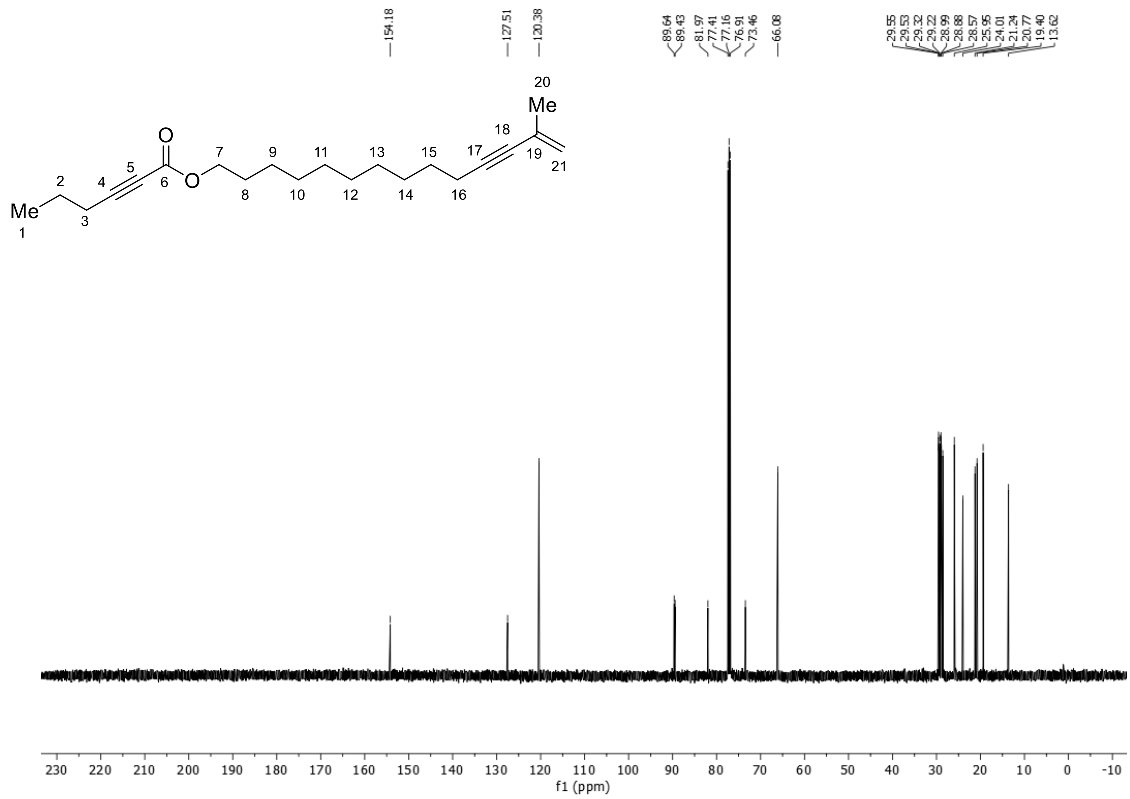

Supplementary Figure 61. $^{13}C\{^{1}H\}$ NMR of **S26** (126 MHz, 299 K, $CDCl_3$).

13-Methyltetradec-13-en-11-yn-1-yl 4-methylbenzenesulfonate (S27)

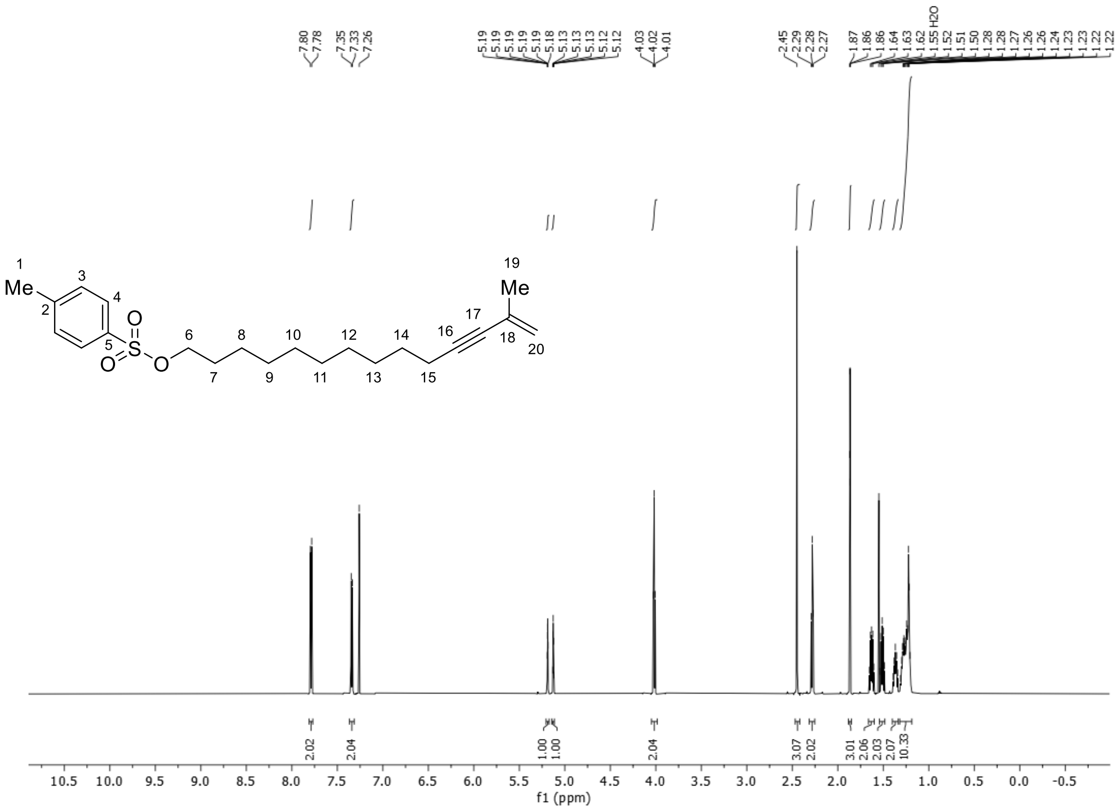

Supplementary Figure 62. ^{1}H NMR of **S27** (599 MHz, 299 K, $CDCl_3$).

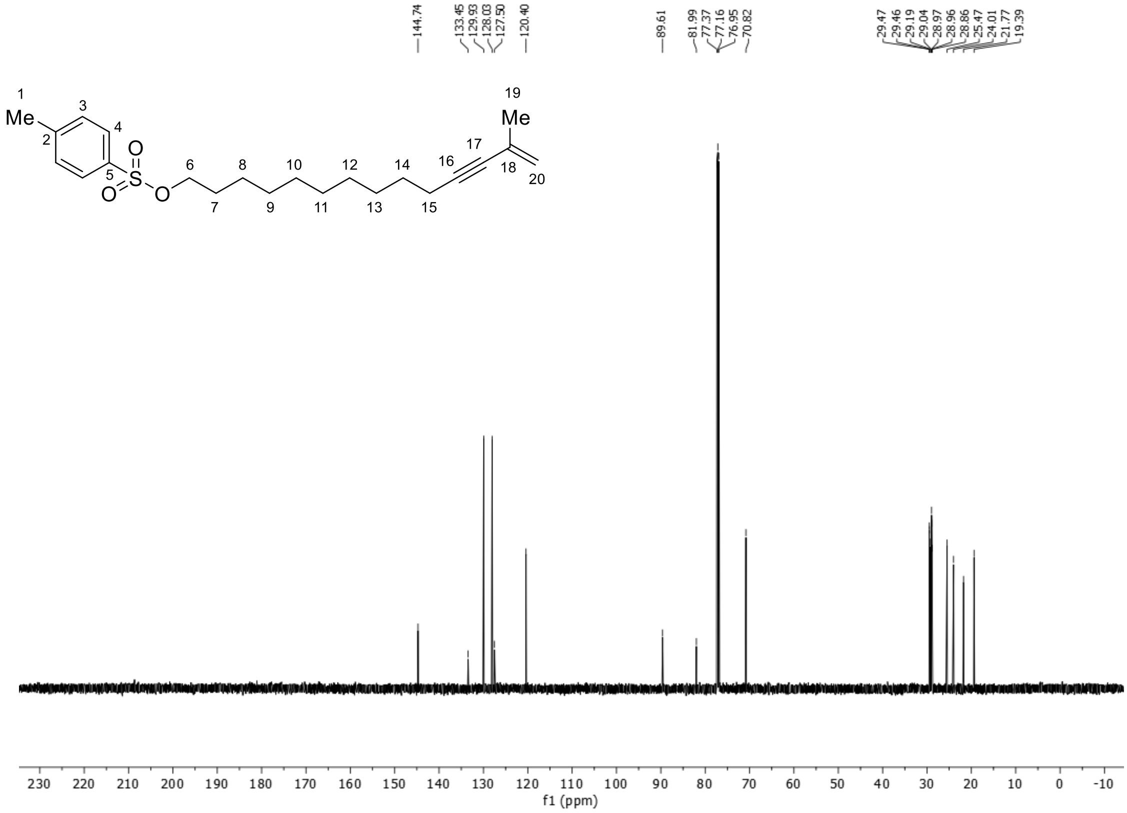

Supplementary Figure 63. $^{13}C\{^{1}H\}$ NMR of **S27** (151 MHz, 299 K, $CDCl_3$).

13-Methyltetradec-13-en-11-yn-1-ol (S28)

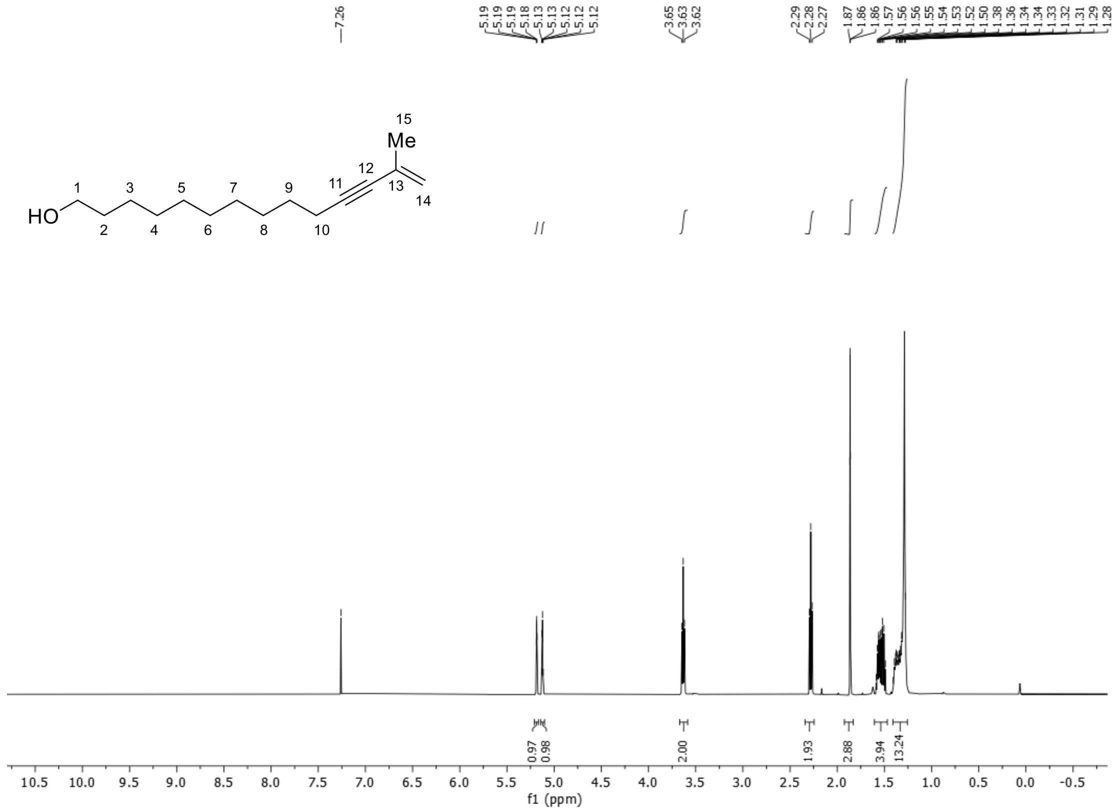

Supplementary Figure 64. ^{1}H NMR of **S28** (500 MHz, 299 K, $CDCl_3$).

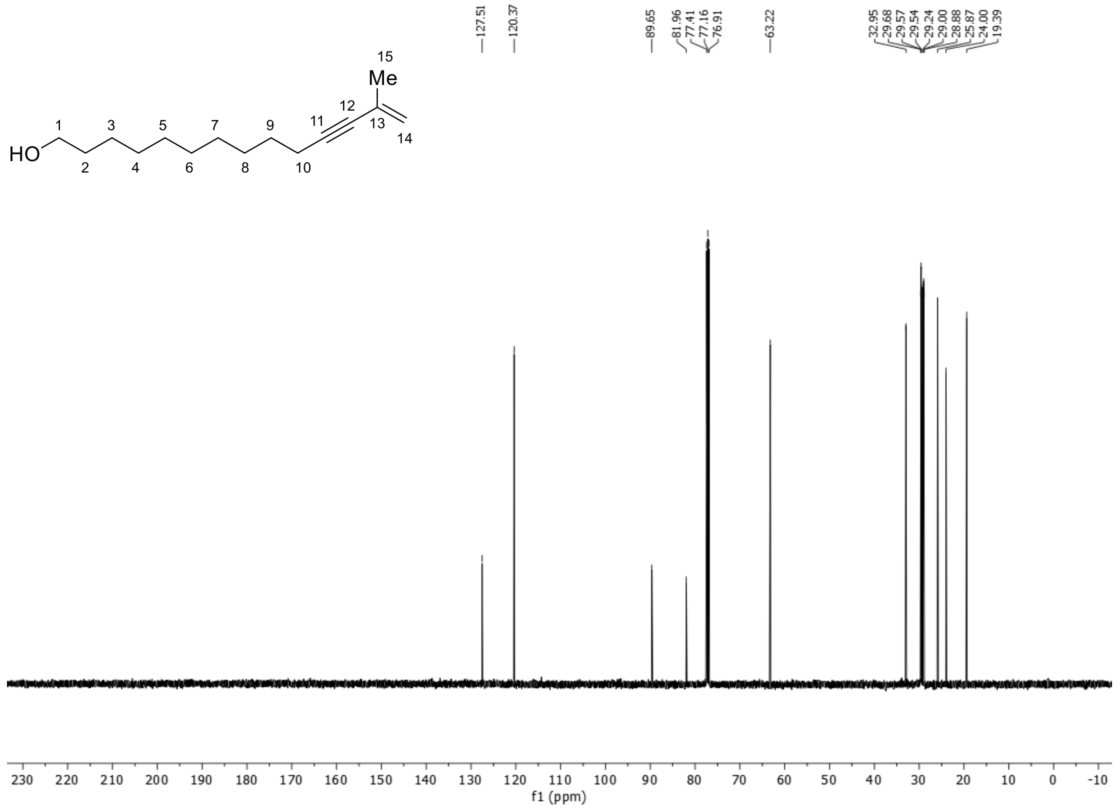

Supplementary Figure 65. $^{13}C\{^1H\}$ NMR of **S28** (126 MHz, 299 K, $CDCl_3$).

2-Methyl-3-methyleneoctadec-4-yne (S29)

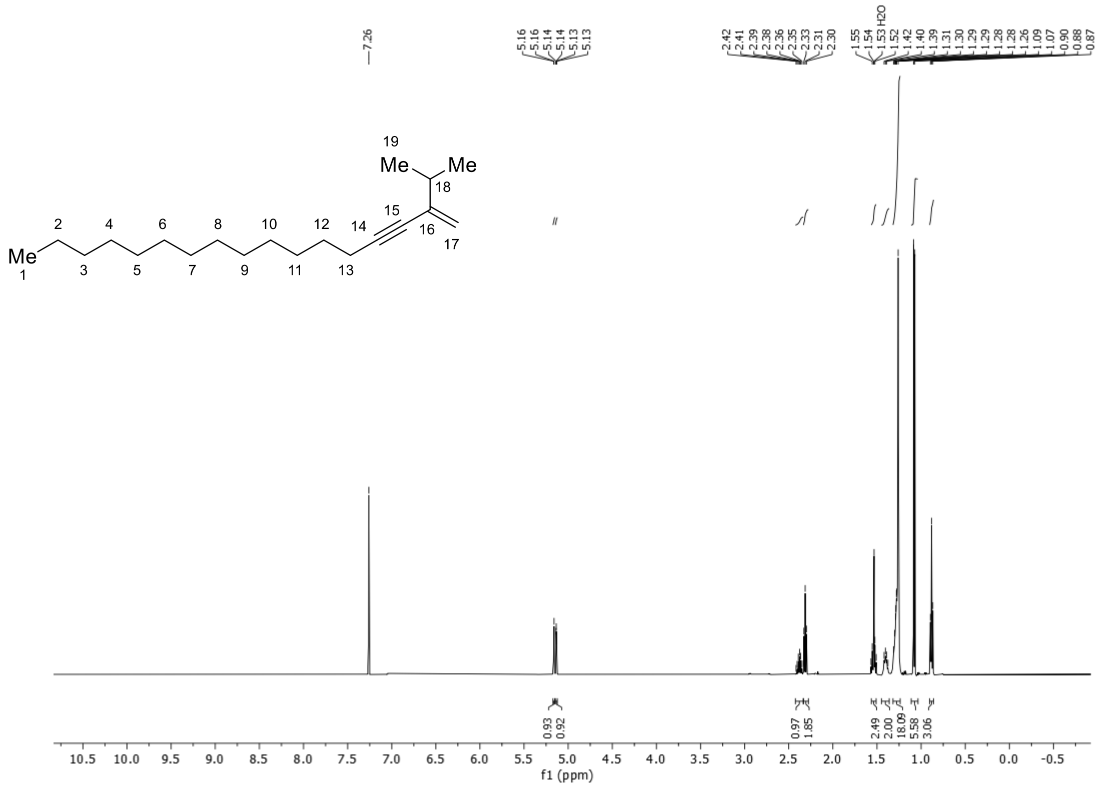

Supplementary Figure 66. ^{1}H NMR of **S29** (500 MHz, 299 K, $CDCl_3$).

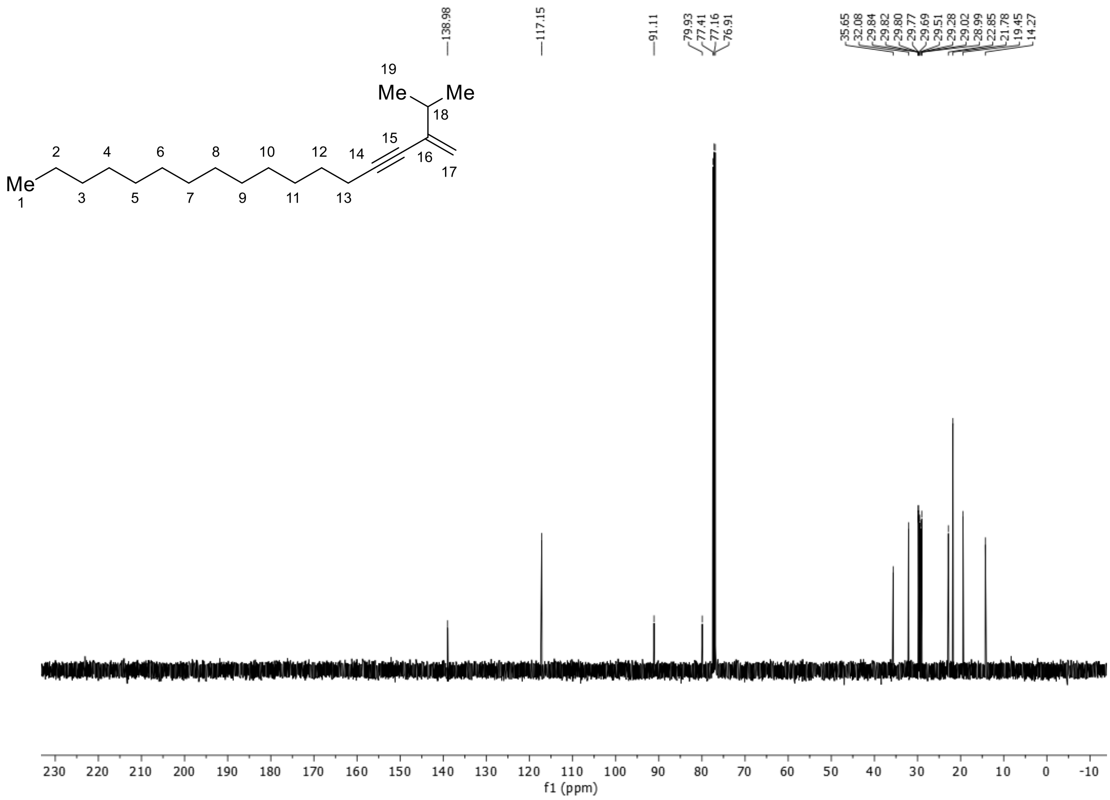

Supplementary Figure 67. $^{13}C\{^{1}H\}$ NMR of **S29** (126 MHz, 299 K, $CDCl_3$).

Heptadec-1-en-3-yn-2-ylcyclohexane (S30)

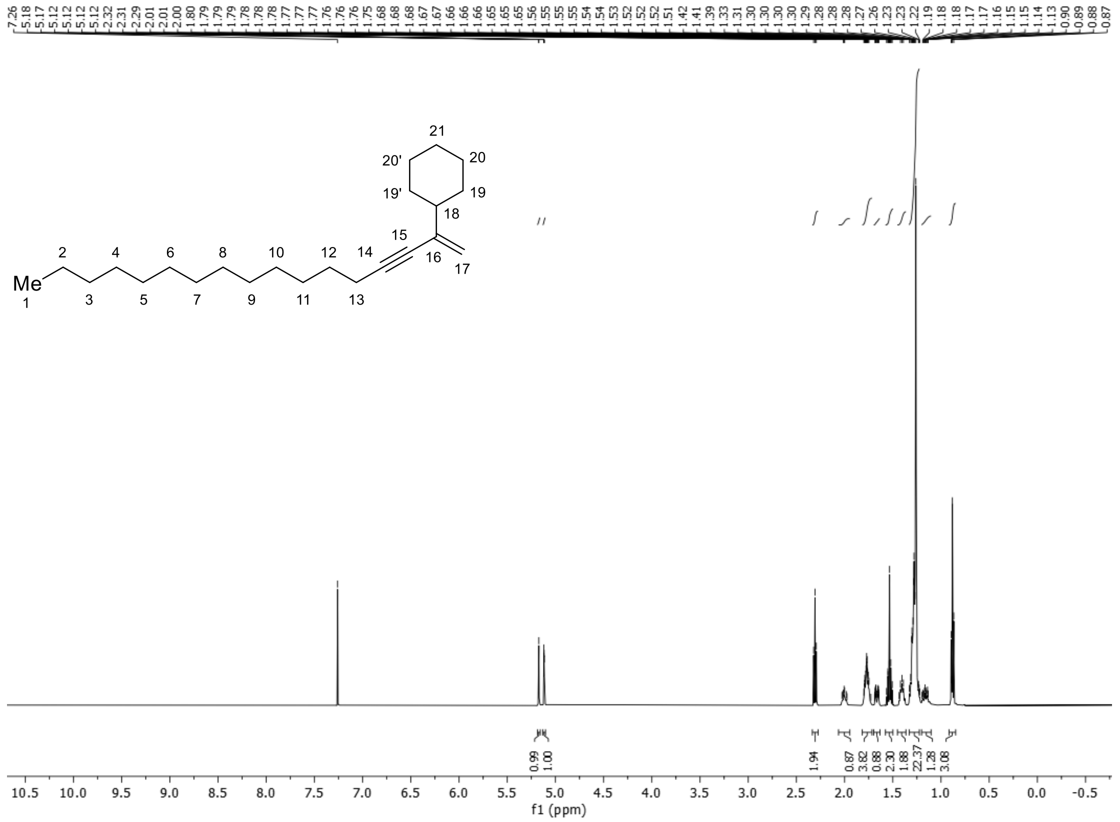

Supplementary Figure 68. ^{1}H NMR of **S30** (500 MHz, 299 K, $CDCl_3$).

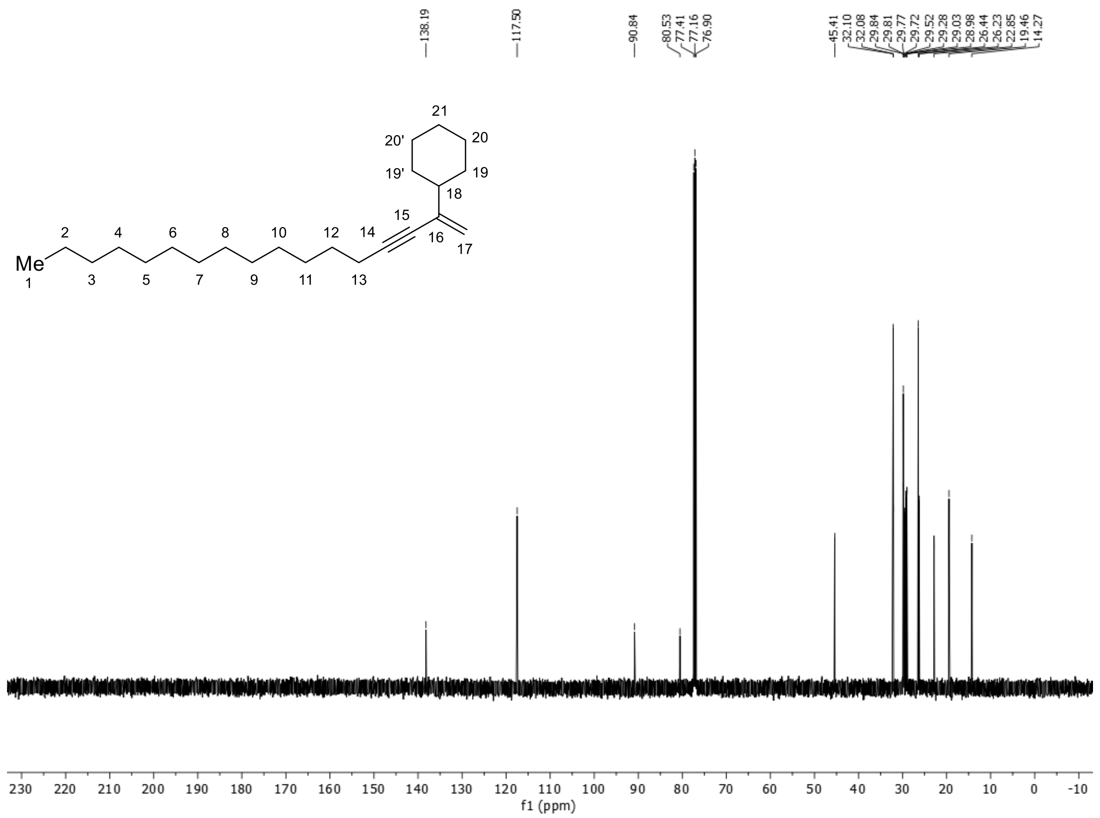

Supplementary Figure 69. $^{13}C\{^{1}H\}$ NMR of **S30** (126 MHz, 299 K, $CDCl_3$).

2-(Fluoromethyl)hexadec-1-en-3-yne (S31)

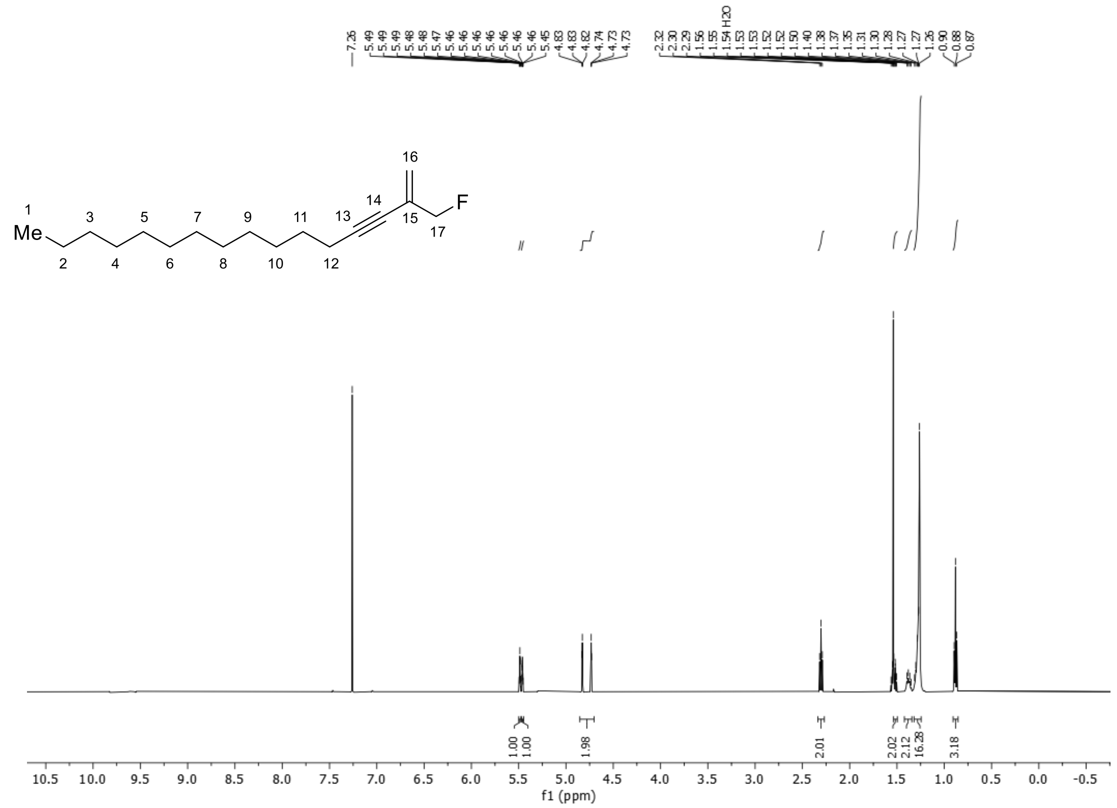

Supplementary Figure 70. ^{1}H NMR of **S31** (500 MHz, 299 K, $CDCl_3$).

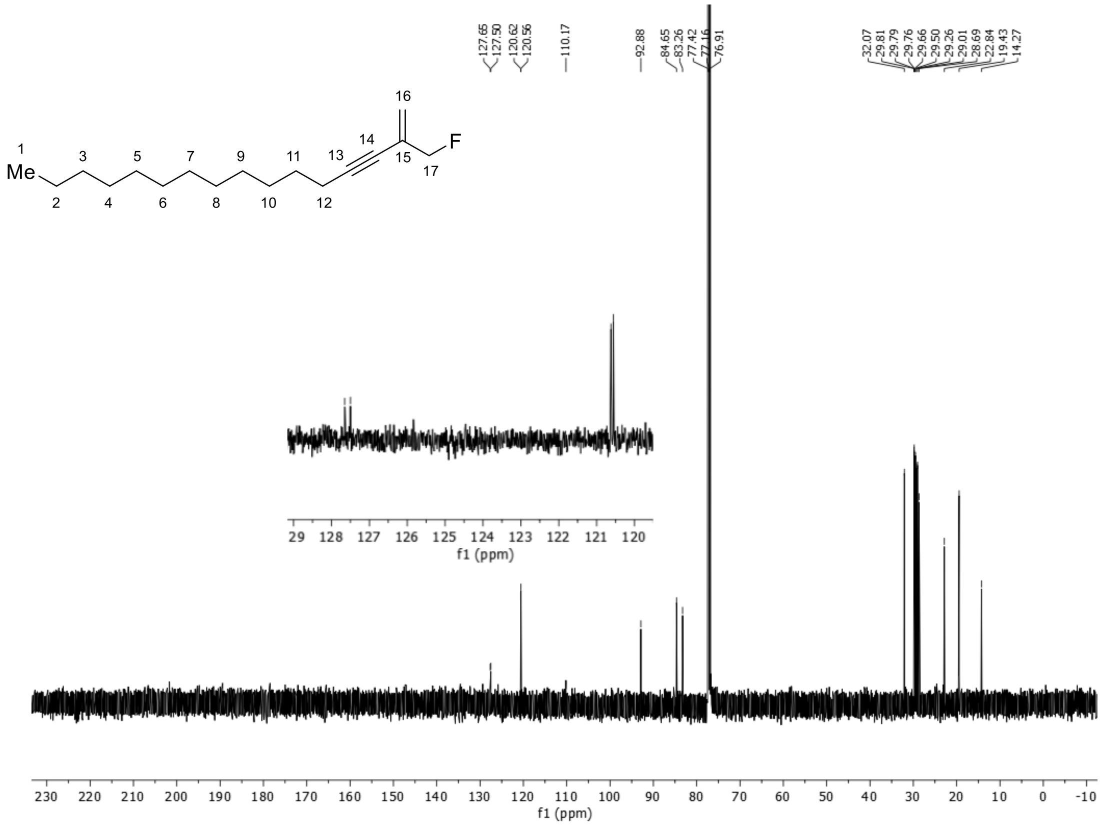

Supplementary Figure 71. $^{13}C\{^{1}H\}$ NMR of **S31** (126 MHz, 299 K, $CDCl_3$).

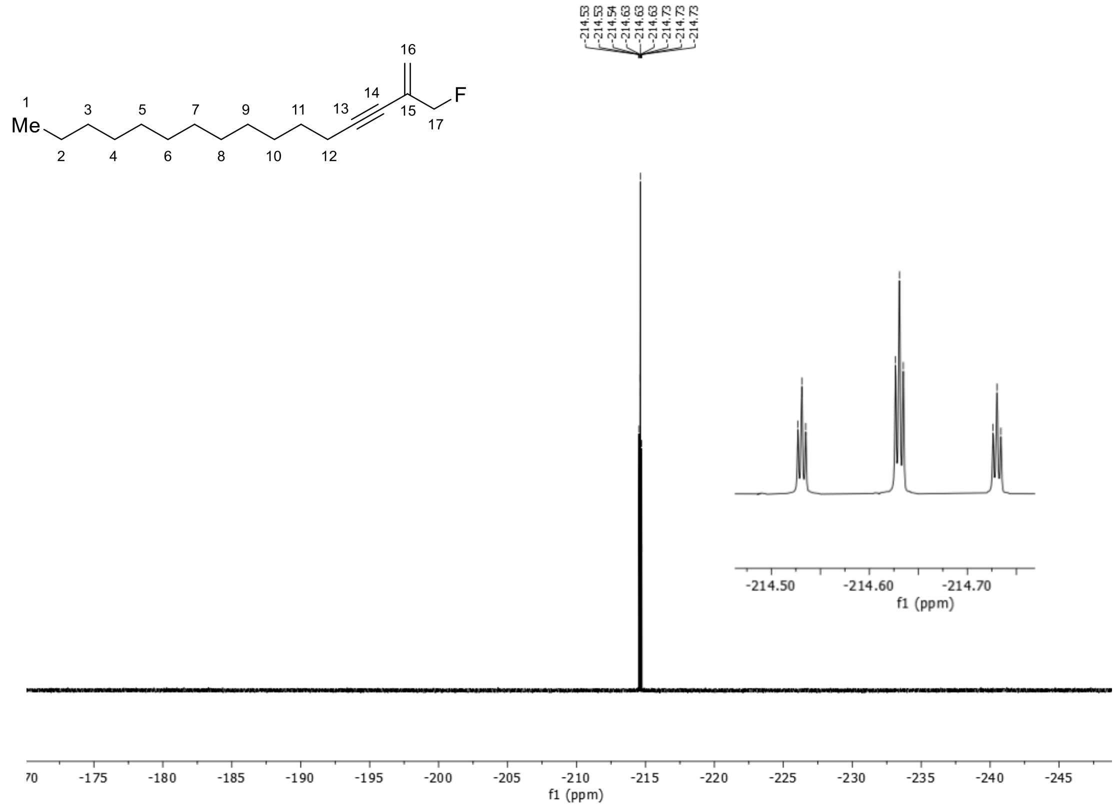

Supplementary Figure 72. ^{19}F NMR of **S31** (470 MHz, 299 K, $CDCl_3$).

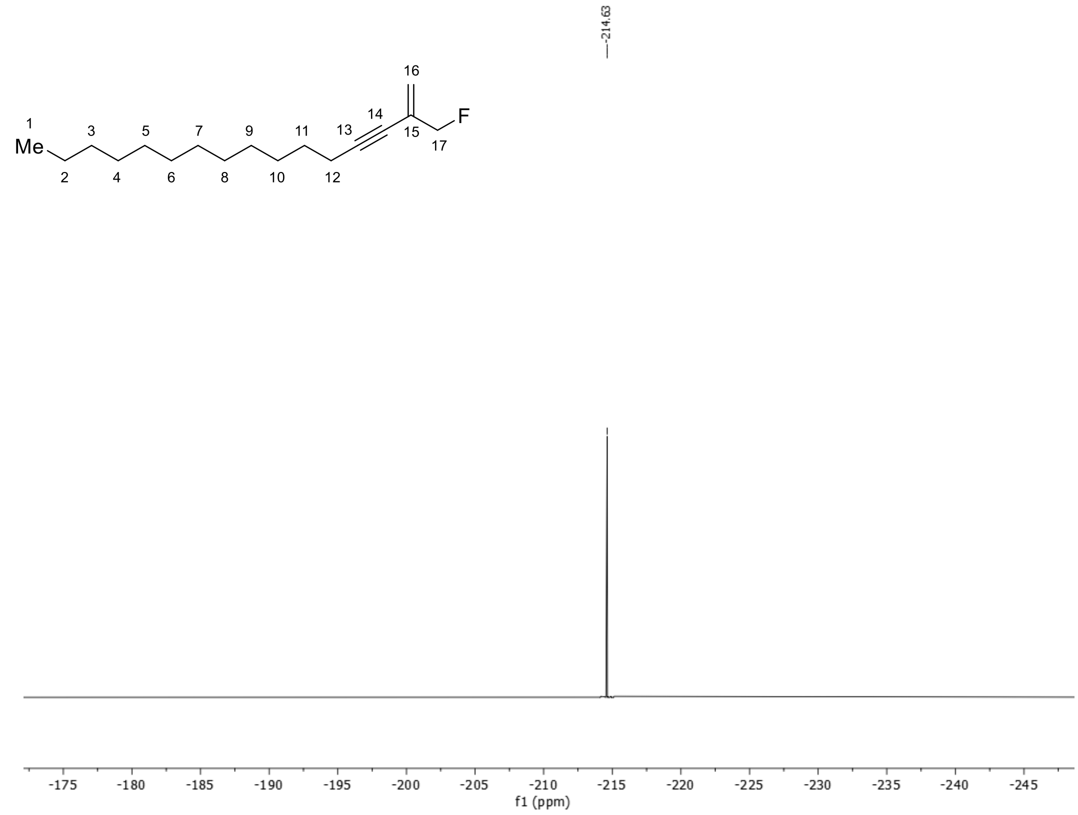

Supplementary Figure 73. $^{19}F\{^1H\}$ NMR of **S31** (470 MHz, 299 K, $CDCl_3$).

13-Methyltetradec-13-en-11-yn-1-yl-2-(3-cyano-4-isobutoxyphenyl)-4-methylthiazole-5-carboxylate (S32)

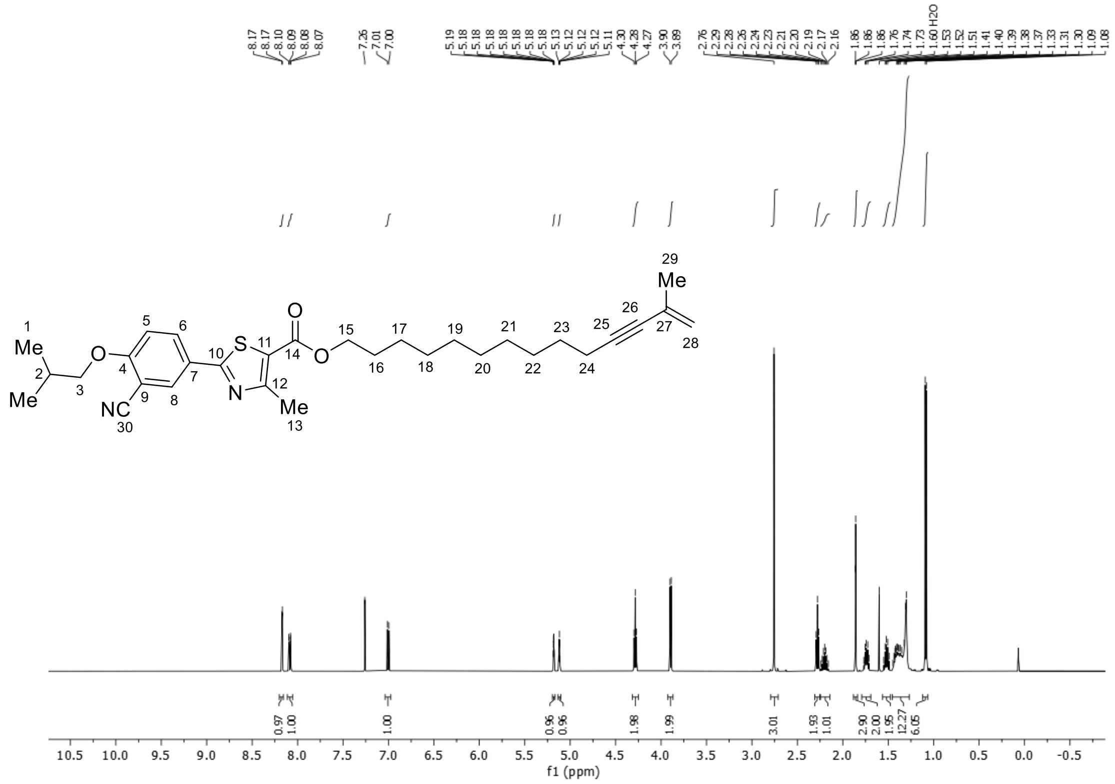

Supplementary Figure 74. ^{1}H NMR of **S32** (500 MHz, 299 K, $CDCl_3$).

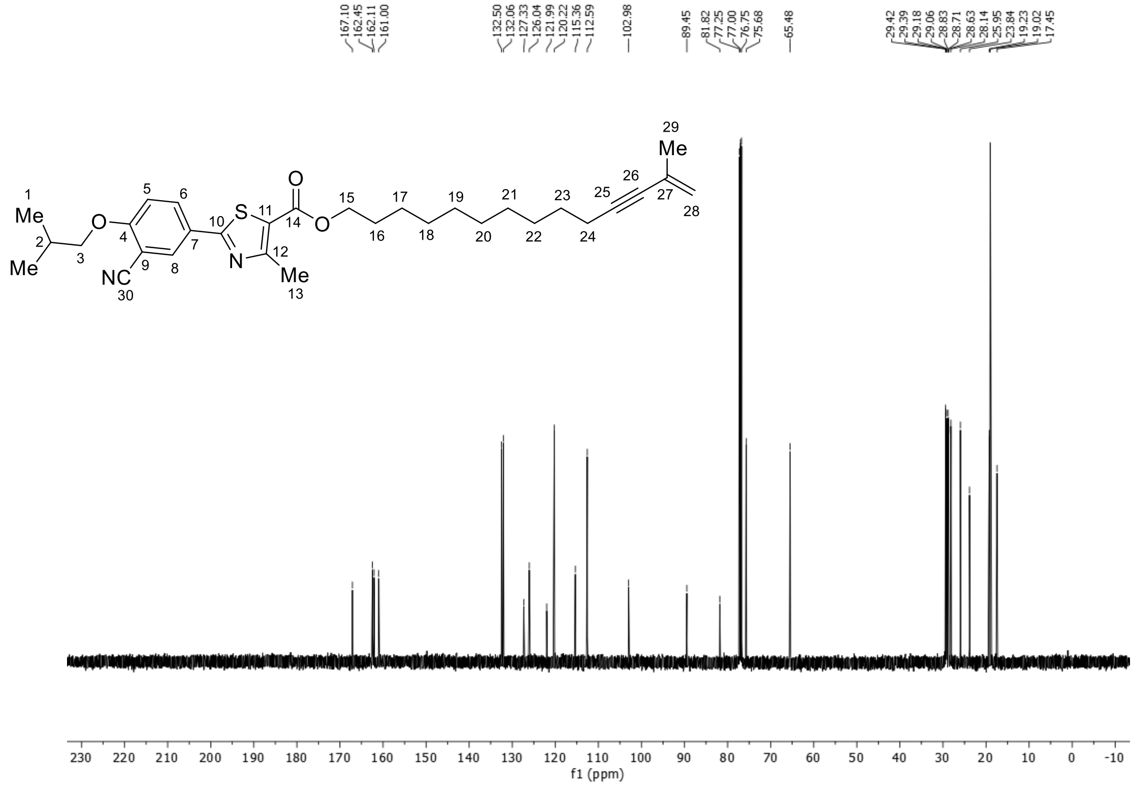

Supplementary Figure 75. $^{13}C\{^{1}H\}$ NMR of **S32** (126 MHz, 299 K, $CDCl_3$).

2-bromooct-1-ene (Int 2)

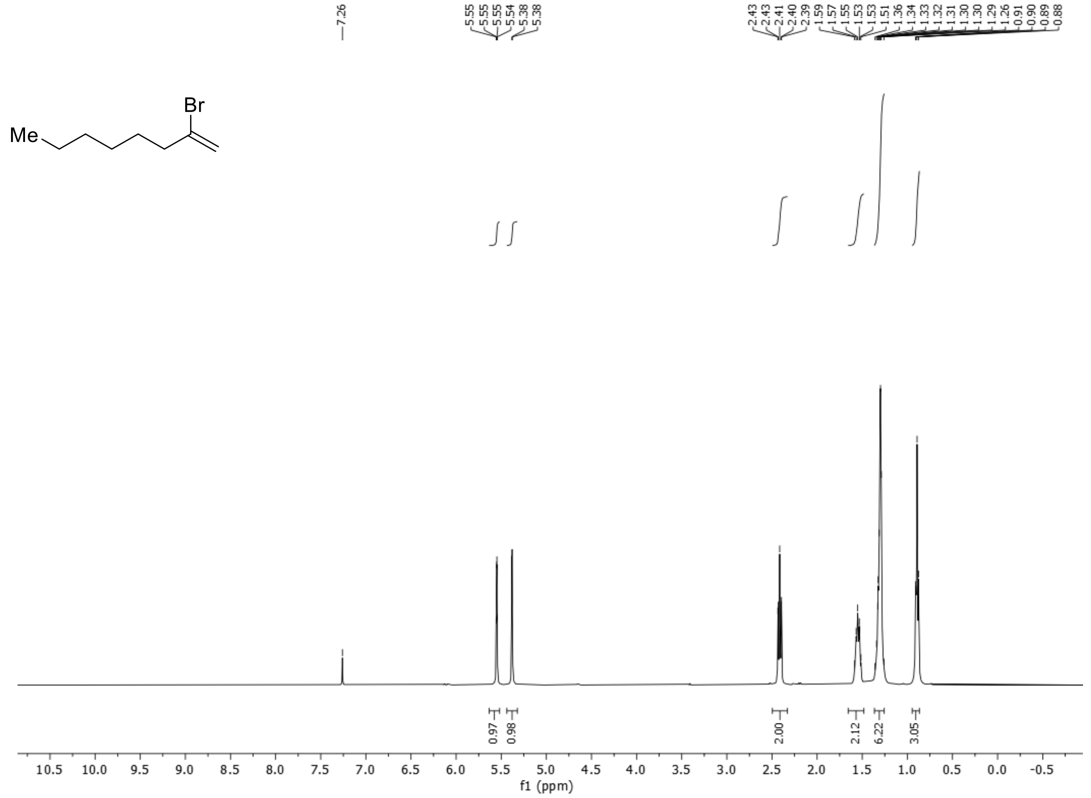

Supplementary Figure 76. ^{1}H NMR of **Int 2** (400 MHz, 299 K, $CDCl_3$).

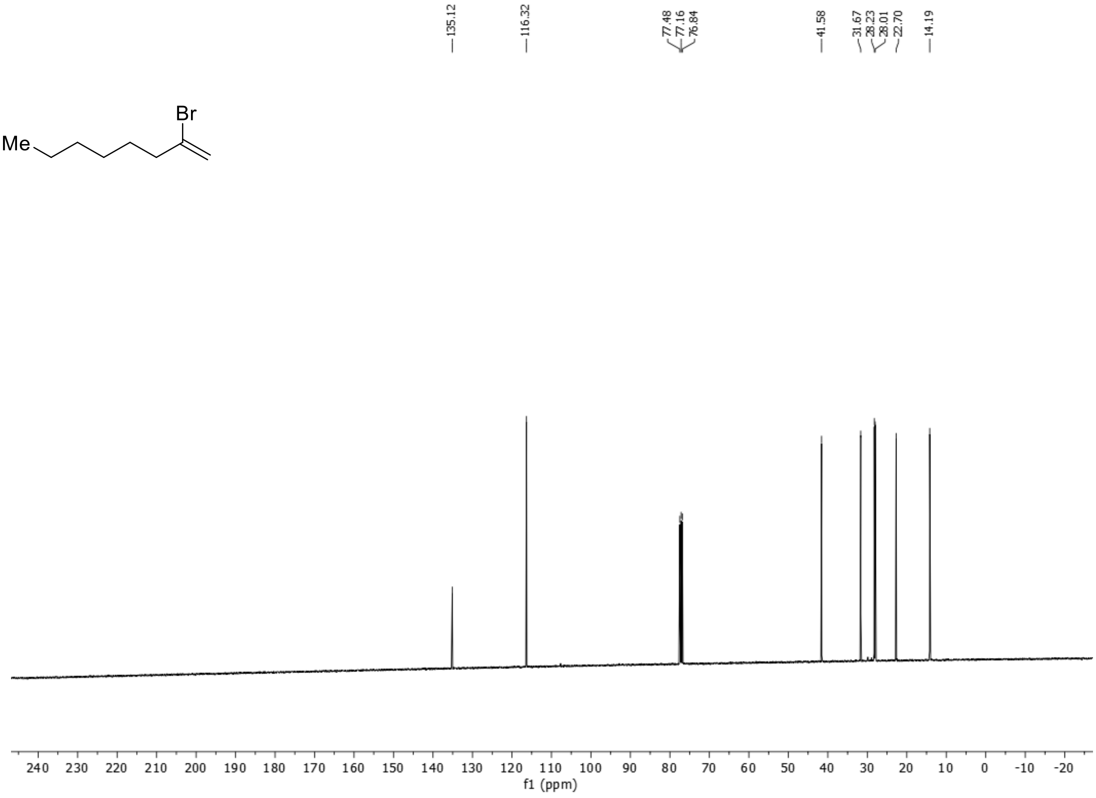

Supplementary Figure 77. $^{13}C\{^{1}H\}$ NMR of **Int 2** (101 MHz, 299 K, $CDCl_3$).

Triisopropyl(3-methylenenon-1-yn-1-yl)silane (S33)

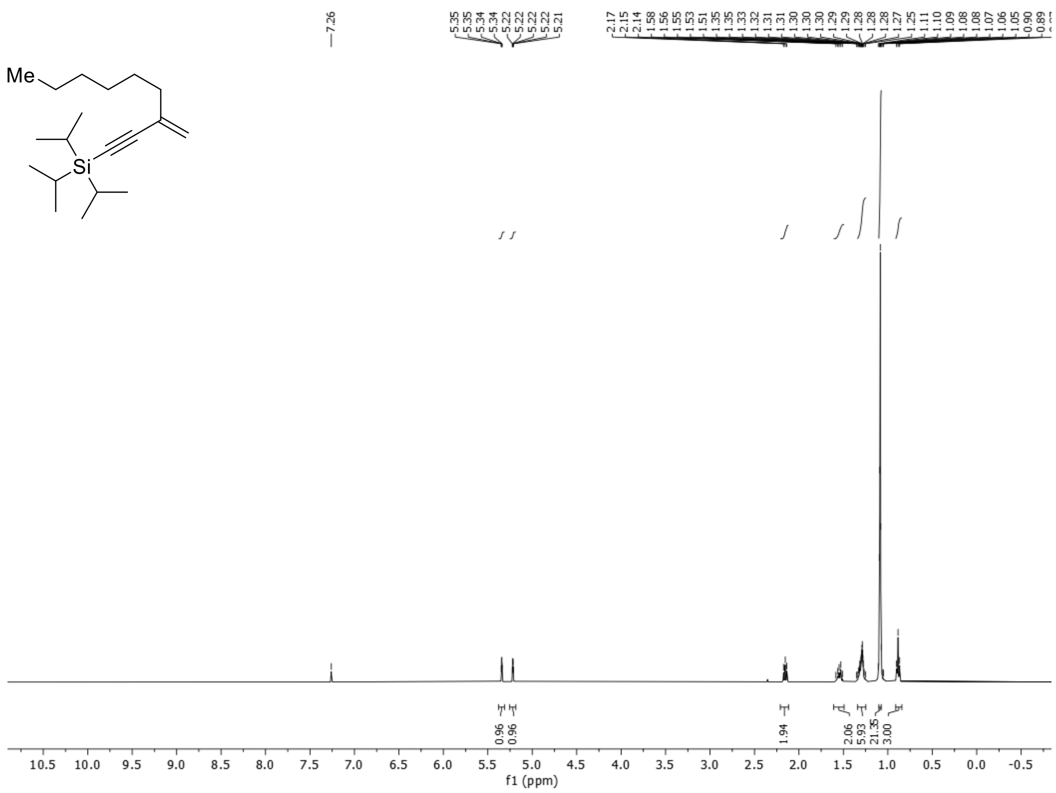

Supplementary Figure 78. ^{1}H NMR of **S33** (400 MHz, 299 K, $CDCl_3$).

1-(4,4-Difluoropent-1-yn-1-yl)-4-nitrobenzene (1)

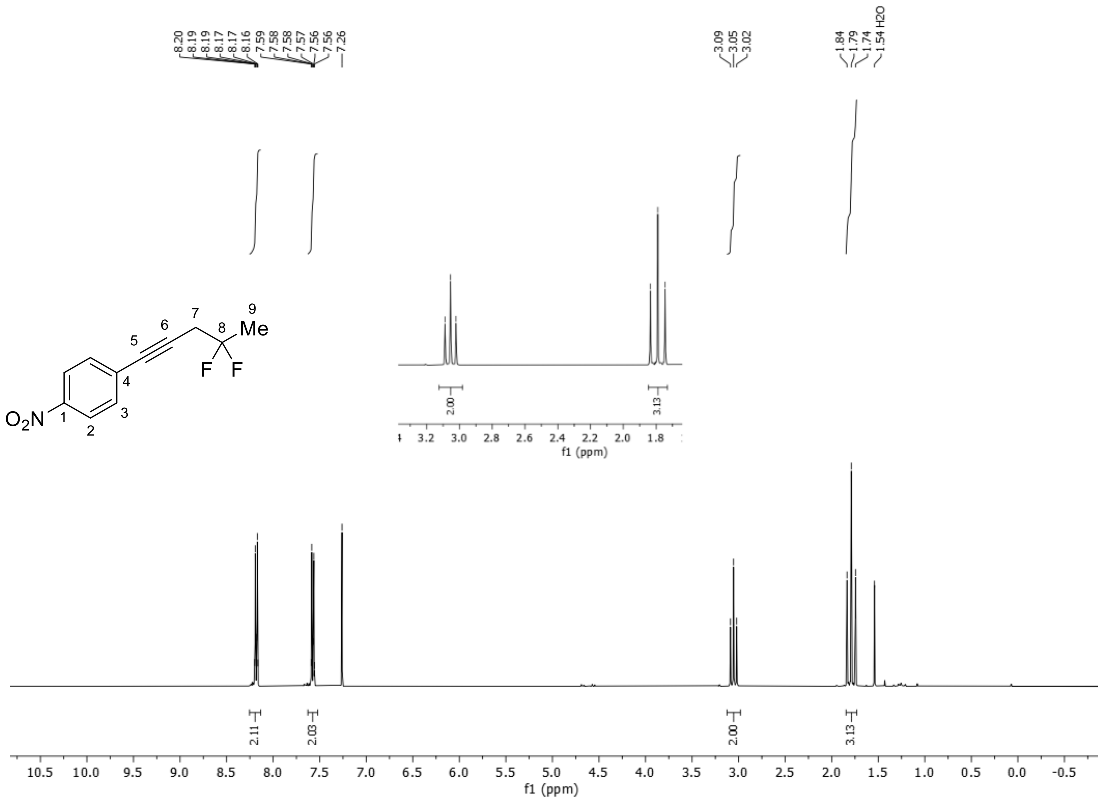

Supplementary Figure 79. ^{1}H NMR of **1** (400 MHz, 299 K, $CDCl_3$).

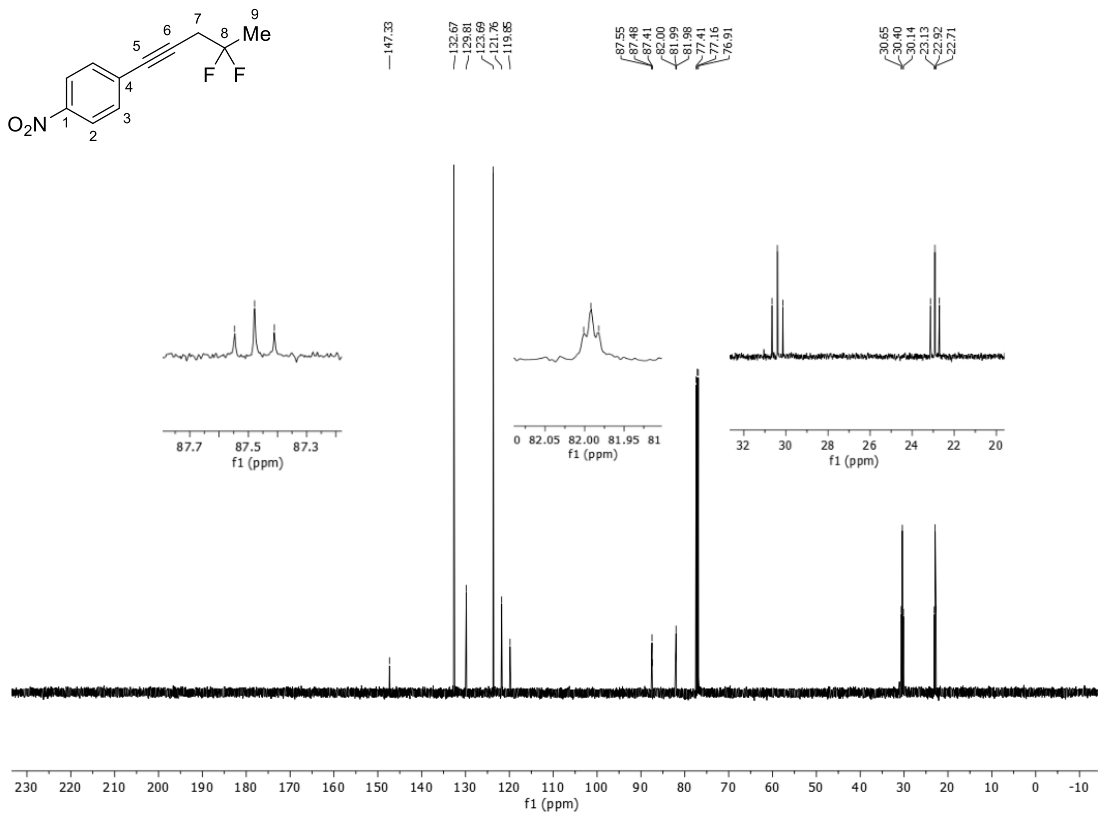

Supplementary Figure 80. $^{13}C\{^{1}H\}$ NMR of **1** (126 MHz, 299 K, $CDCl_3$).

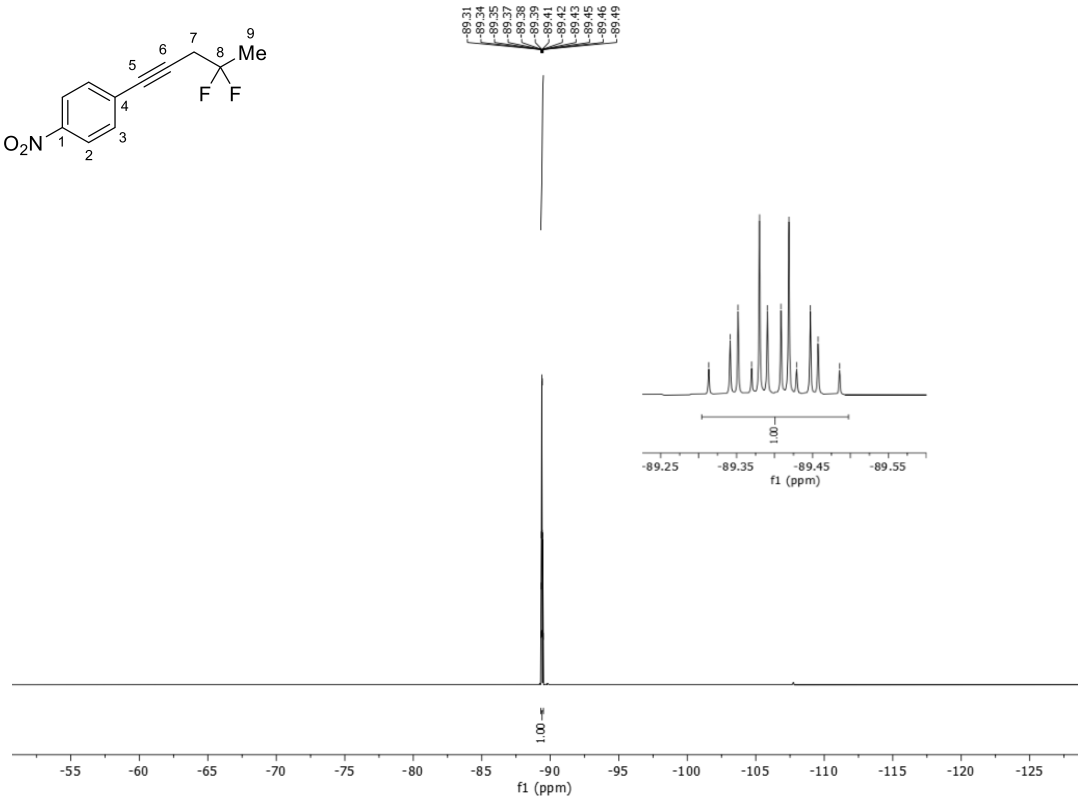

Supplementary Figure 81. ^{19}F NMR of **1** (470 MHz, 299 K, $CDCl_3$).

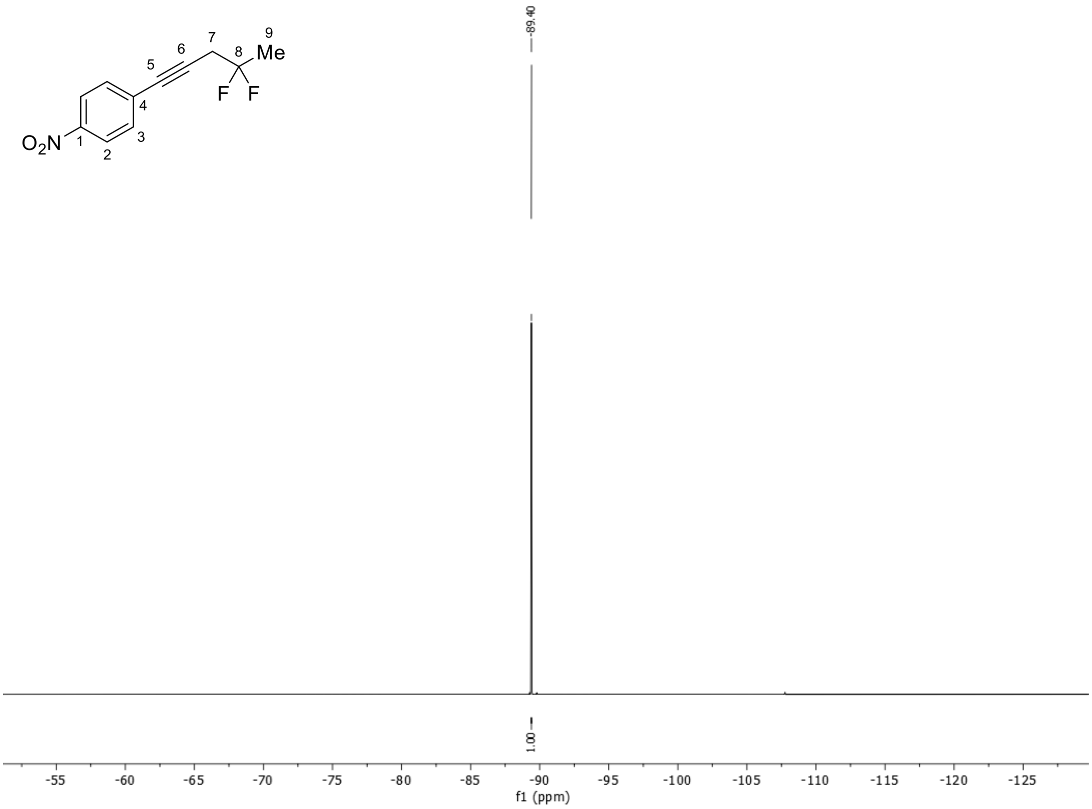

Supplementary Figure 82. $^{19}F\{^1H\}$ NMR of **1** (470 MHz, 299 K, $CDCl_3$).

4-(4,4-Difluoropent-1-yn-1-yl)benzonitrile (2)

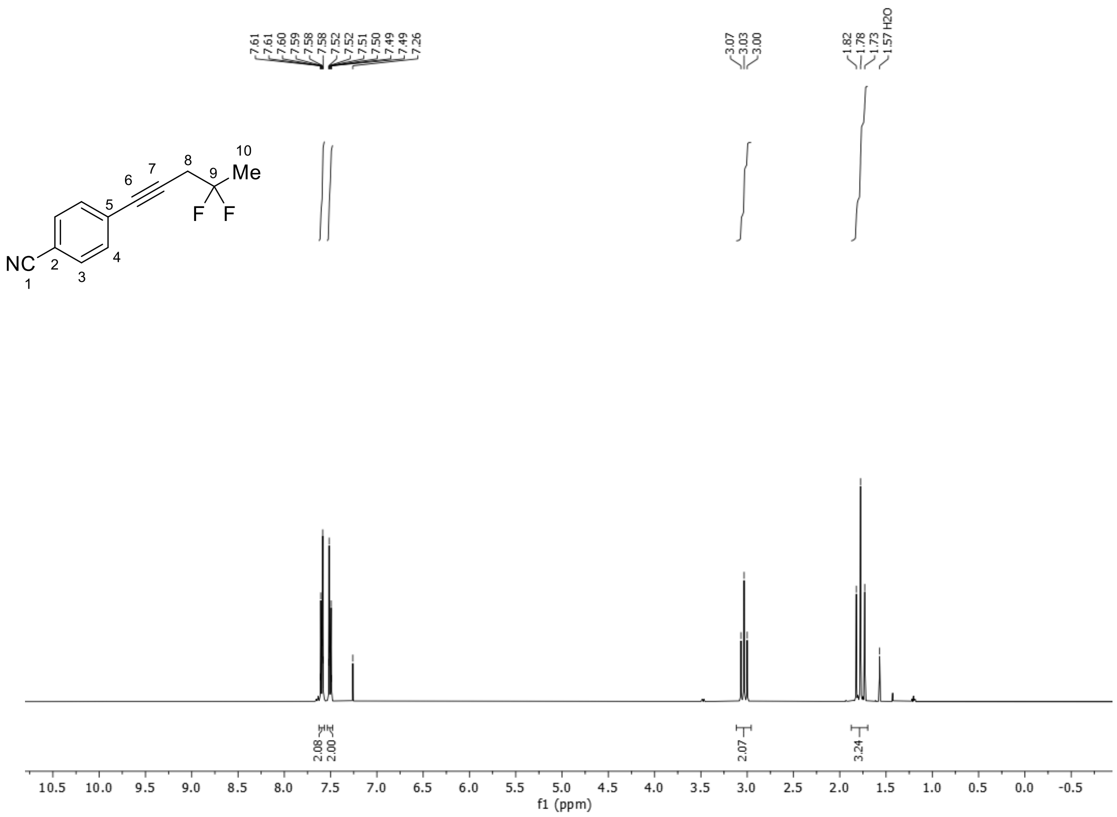

Supplementary Figure 83. ^{1}H NMR of **2** (500 MHz, 299 K, $CDCl_3$).

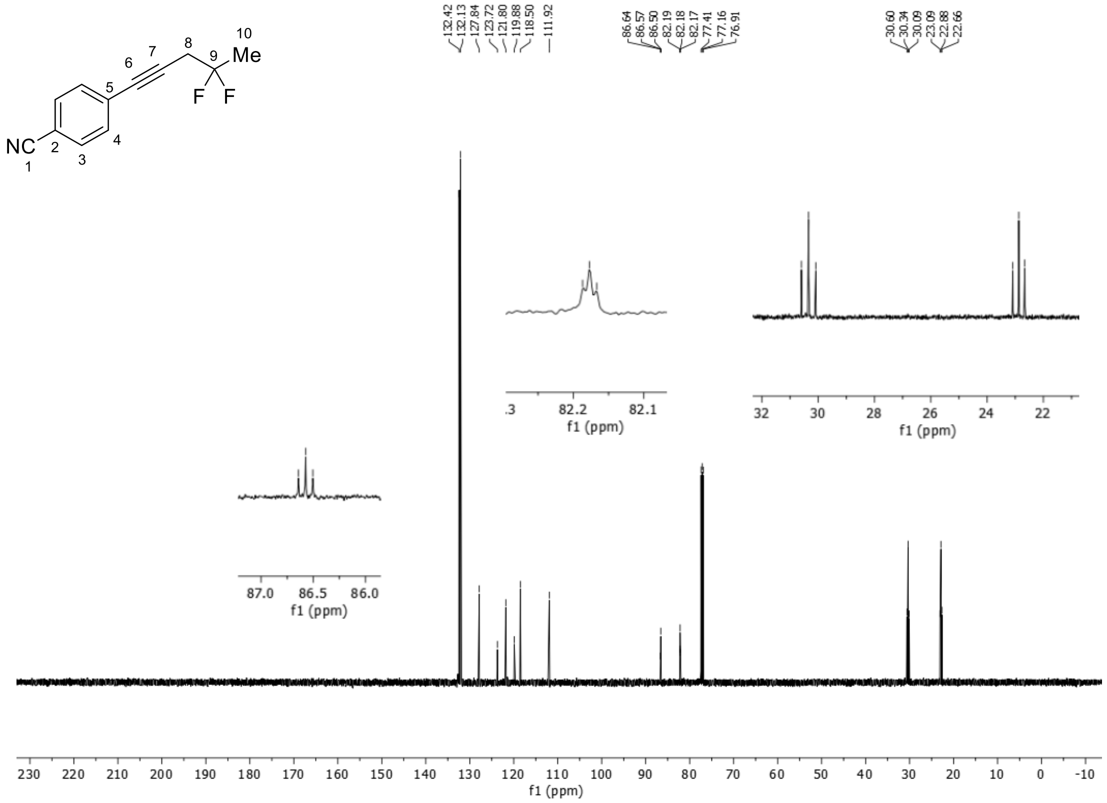

Supplementary Figure 84. $^{13}C\{^{1}H\}$ NMR of **2** (126 MHz, 299 K, $CDCl_3$).

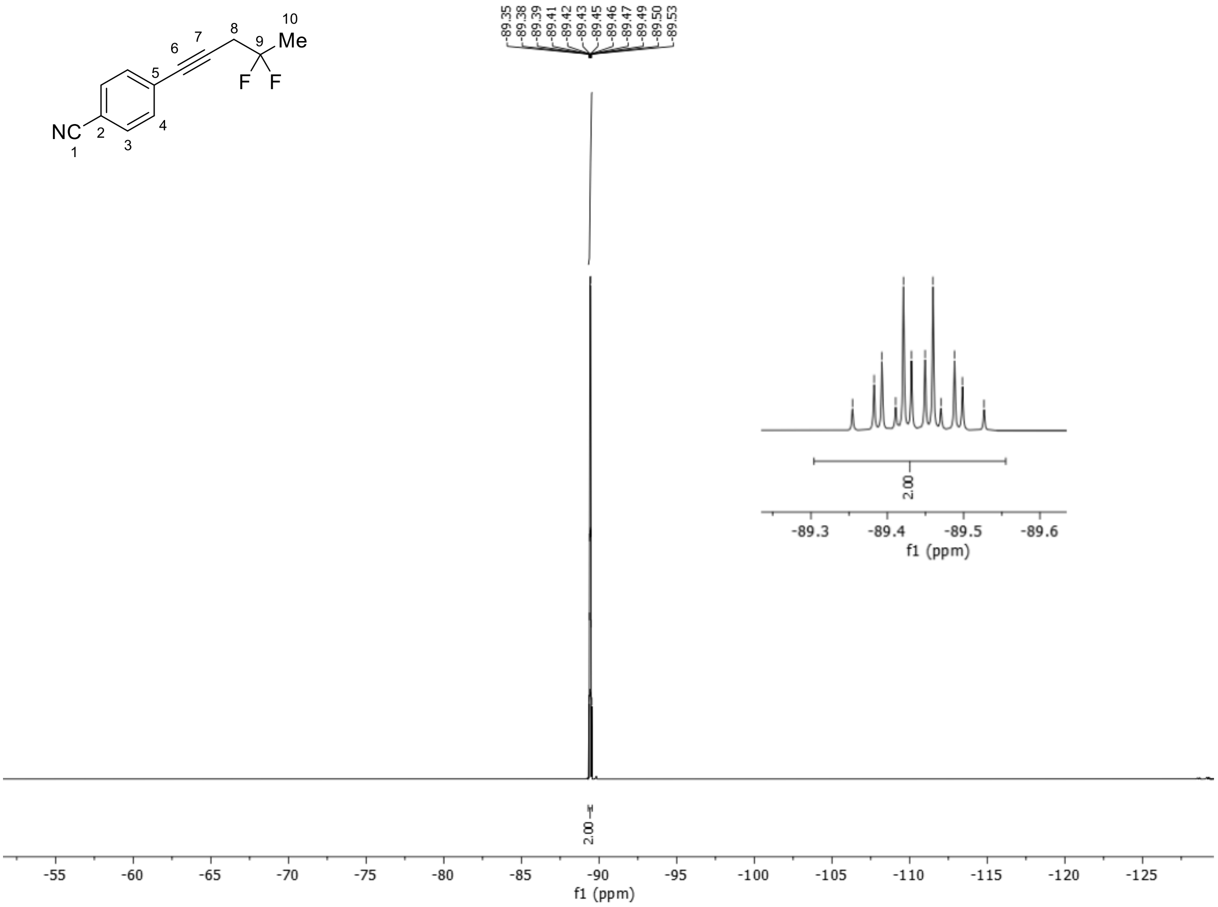

Supplementary Figure 85. ^{19}F NMR of **2** (470 MHz, 299 K, $CDCl_3$).

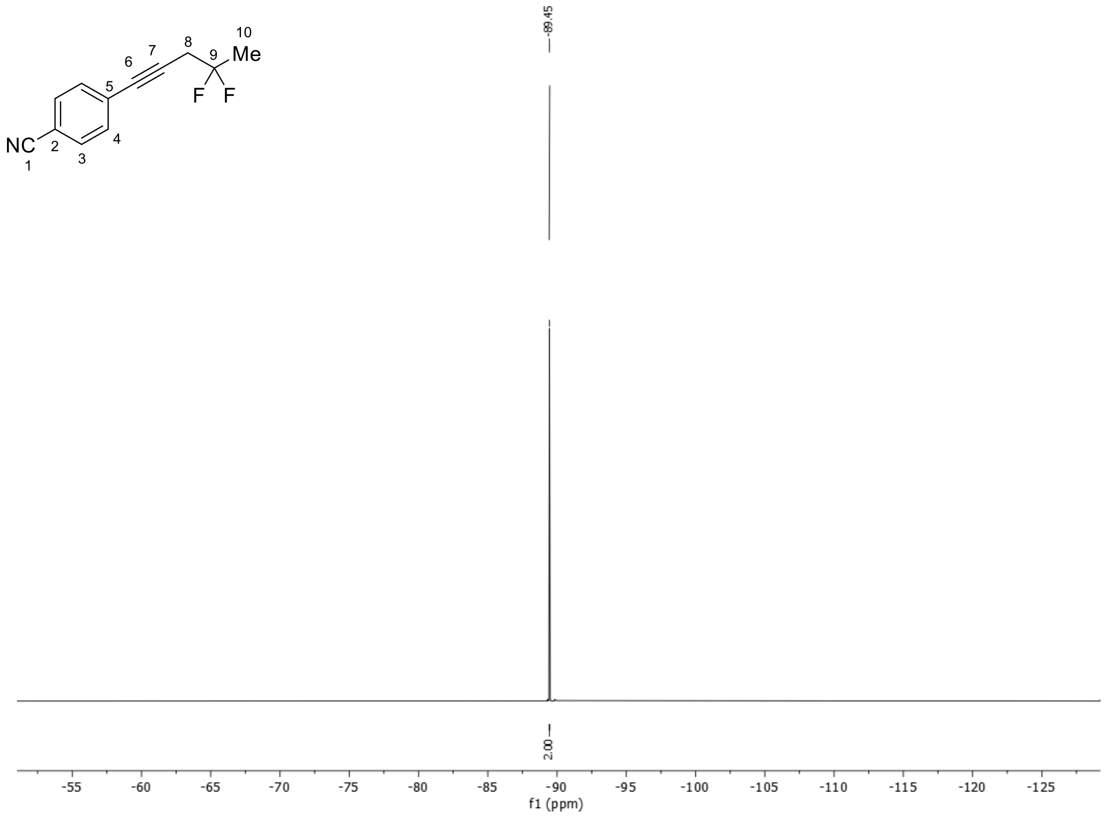

Supplementary Figure 86. $^{19}F\{^{1}H\}$ NMR of **2** (470 MHz, 299 K, $CDCl_3$).

1-(4,4-Difluoropent-1-yn-1-yl)-3,5-bis(trifluoromethyl)benzene (3)

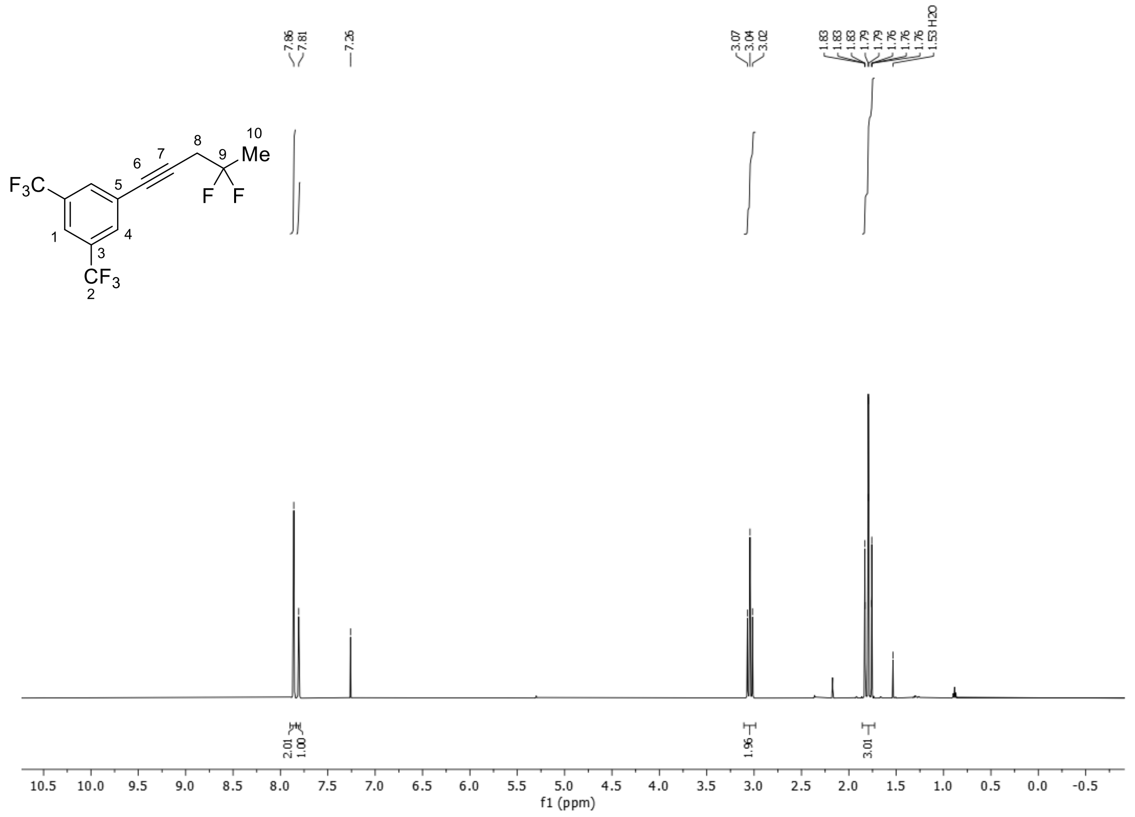

Supplementary Figure 87. ^{1}H NMR of **3** (500 MHz, 299 K, $CDCl_3$).

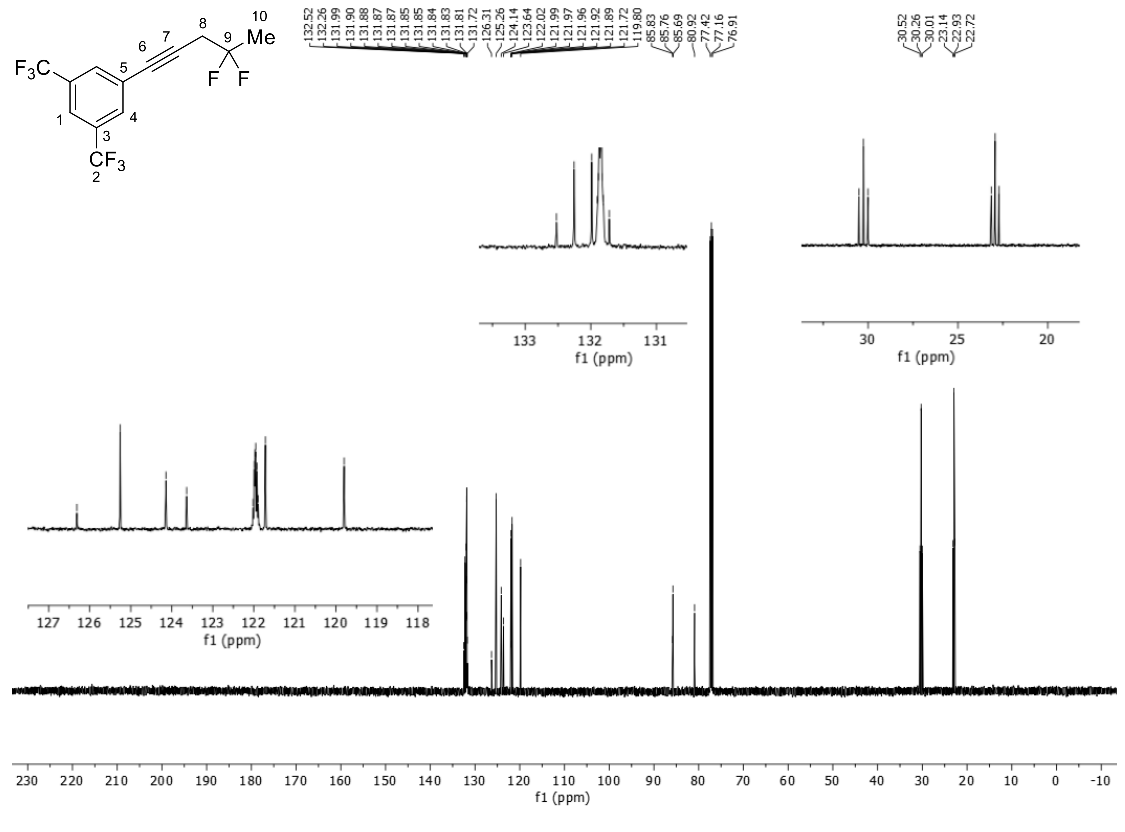

Supplementary Figure 88. $^{13}C\{^{1}H\}$ NMR of **3** (126 MHz, 299 K, $CDCl_3$).

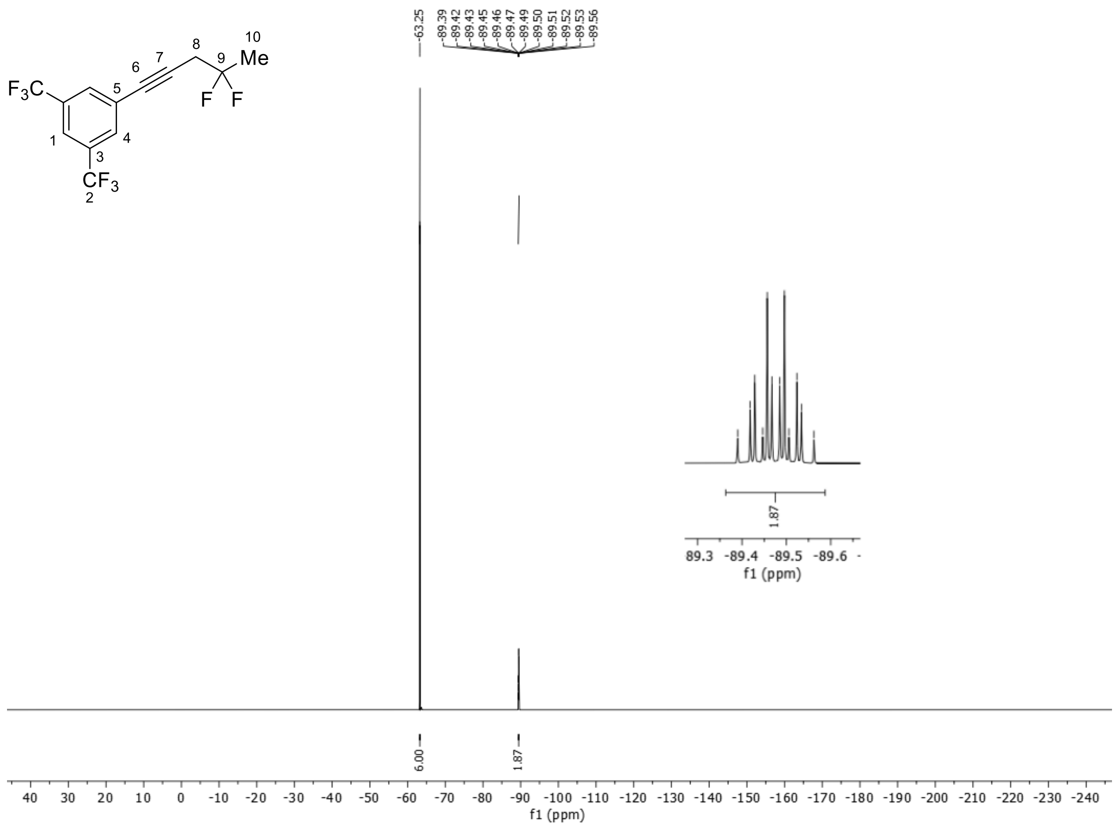

Supplementary Figure 89. ^{19}F NMR of **3** (470 MHz, 299 K, $CDCl_3$).

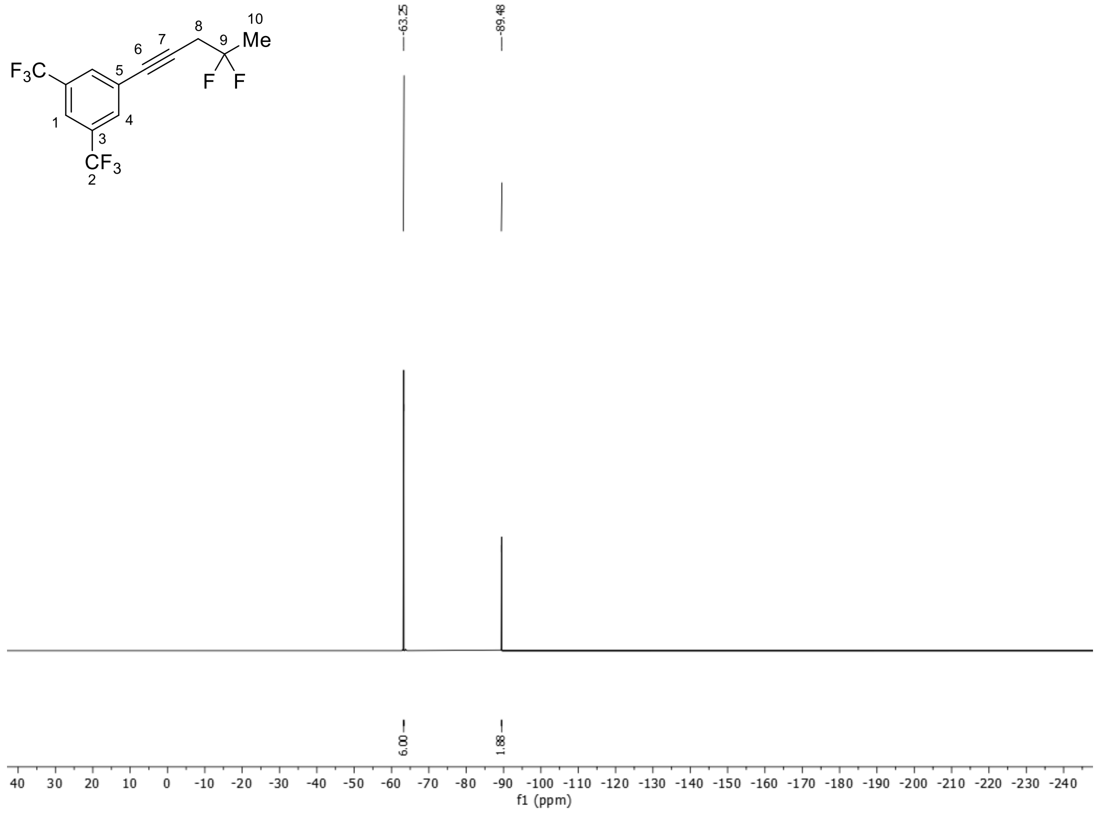

Supplementary Figure 90. $^{19}F\{^1H\}$ NMR of **3** (470 MHz, 299 K, $CDCl_3$).

1-Chloro-4-(4,4-difluoropent-1-yn-1-yl)benzene (4)

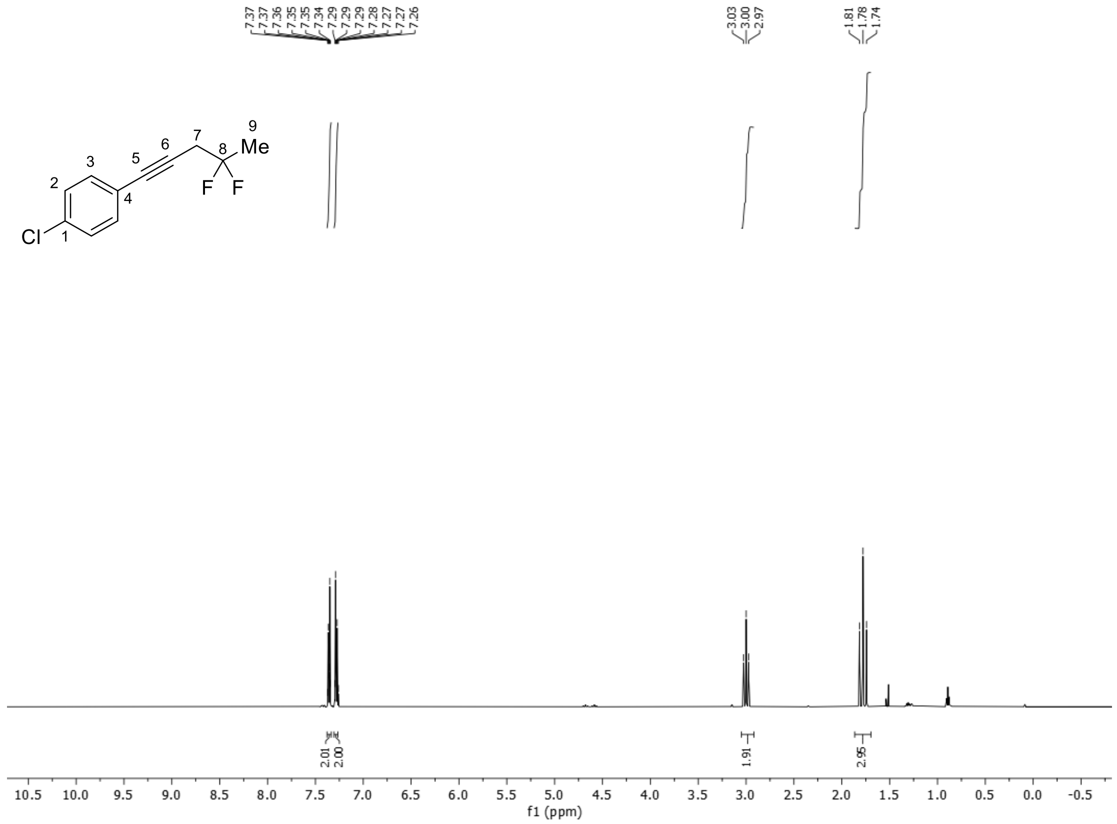

Supplementary Figure 91. ^{1}H NMR of **4** (500 MHz, 299 K, $CDCl_3$).

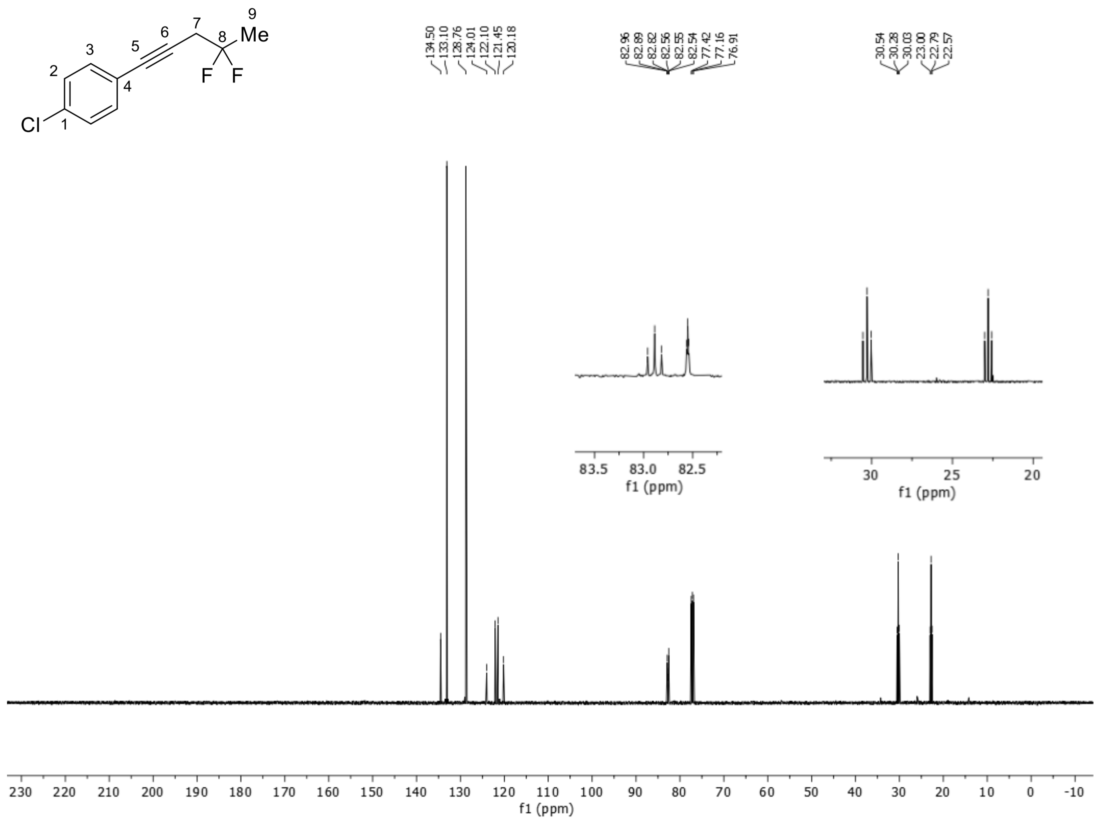

Supplementary Figure 92. $^{13}C\{^{1}H\}$ NMR of **4** (126 MHz, 299 K, $CDCl_3$).

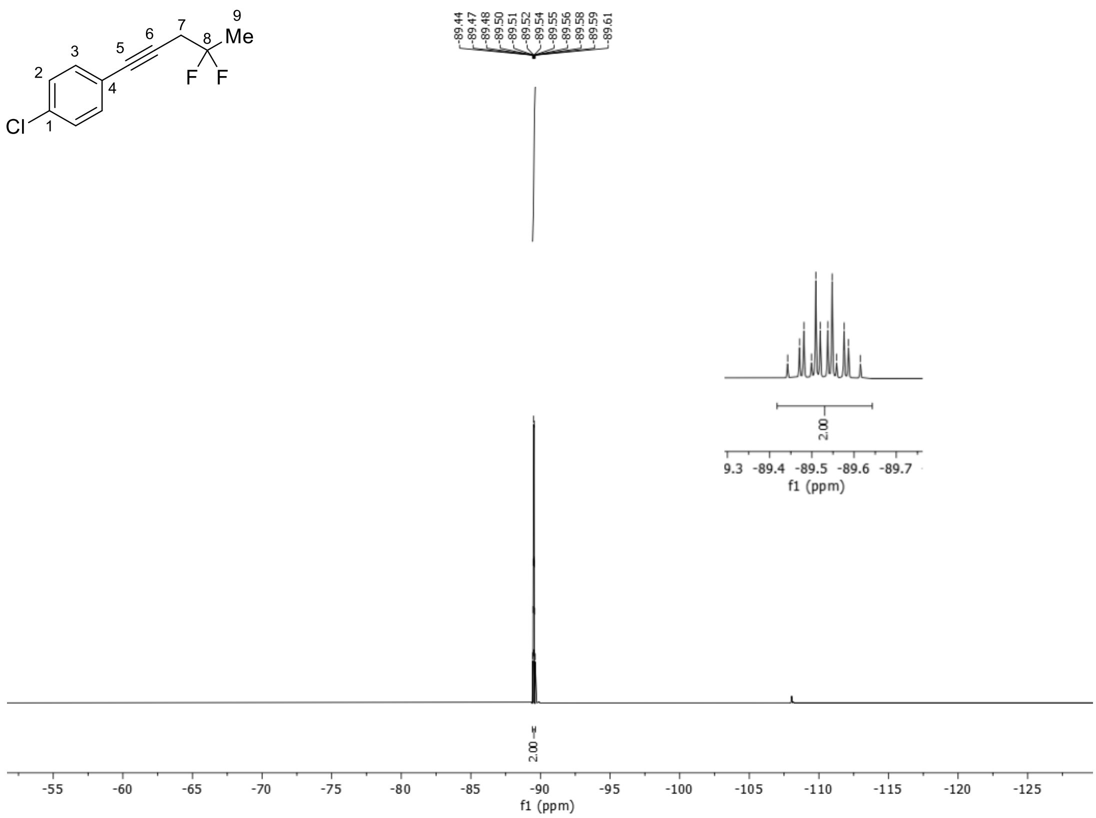

Supplementary Figure 93. ^{19}F NMR of **4** (470 MHz, 299 K, $CDCl_3$).

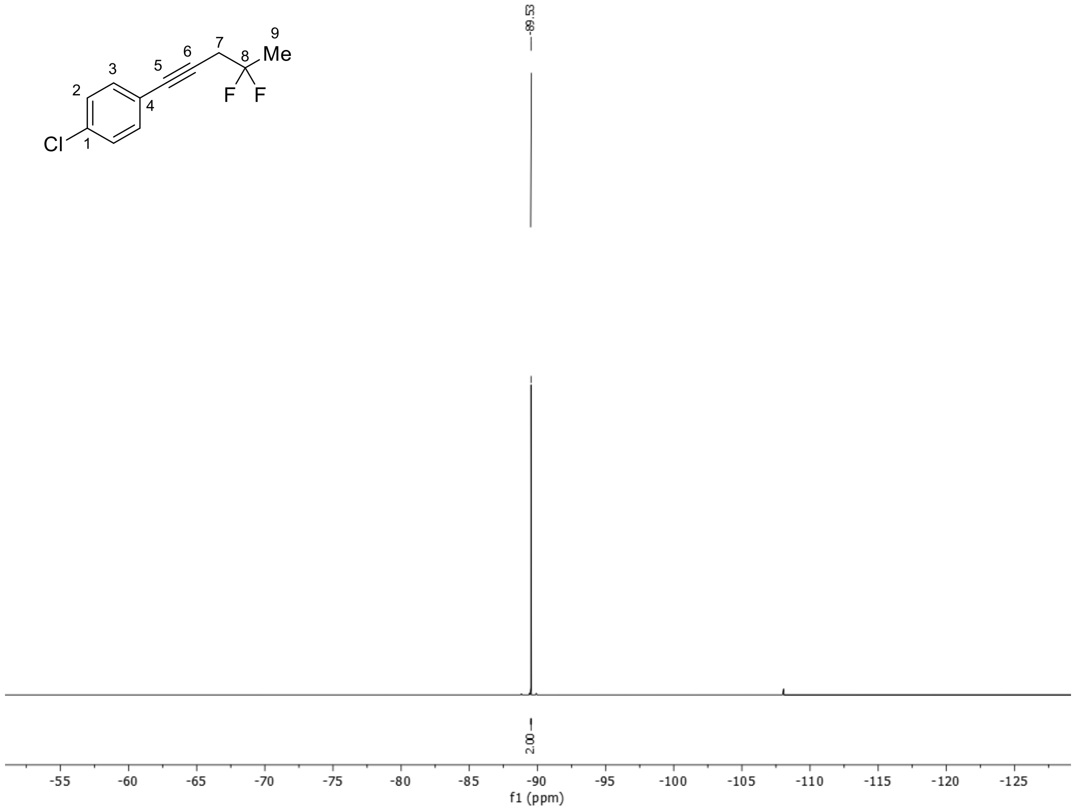

Supplementary Figure 94. $^{19}F\{^1H\}$ NMR of **4** (470 MHz, 299 K, $CDCl_3$).

1-Chloro-3-(4,4-difluoropent-1-yn-1-yl)benzene (5)

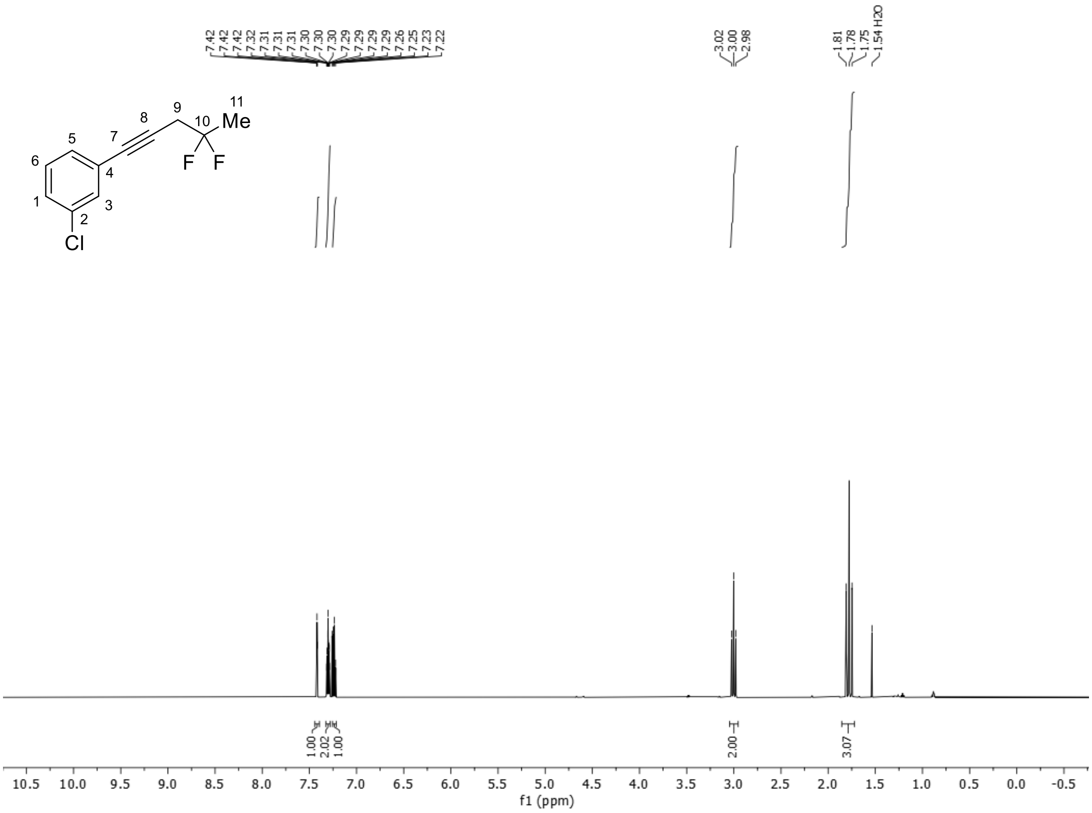

Supplementary Figure 95. ^{1}H NMR of **5** (600 MHz, 299 K, $CDCl_3$).

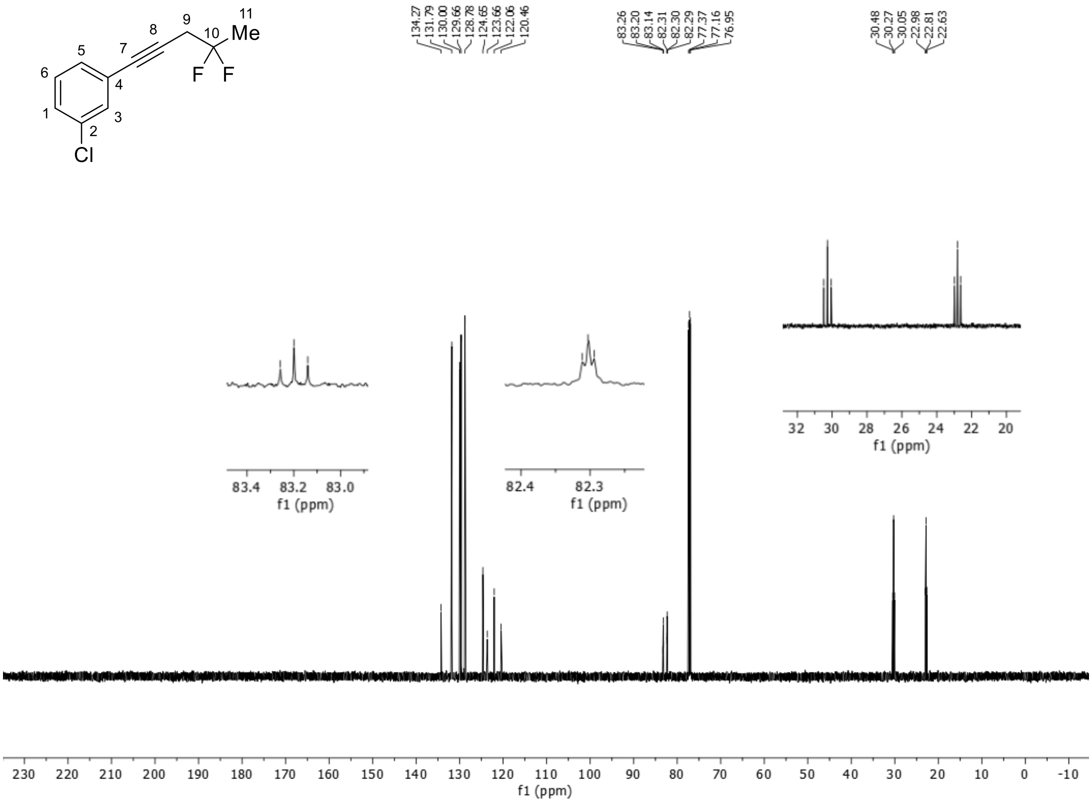

Supplementary Figure 96. $^{13}C\{^{1}H\}$ NMR of **5** (151 MHz, 299 K, $CDCl_3$).

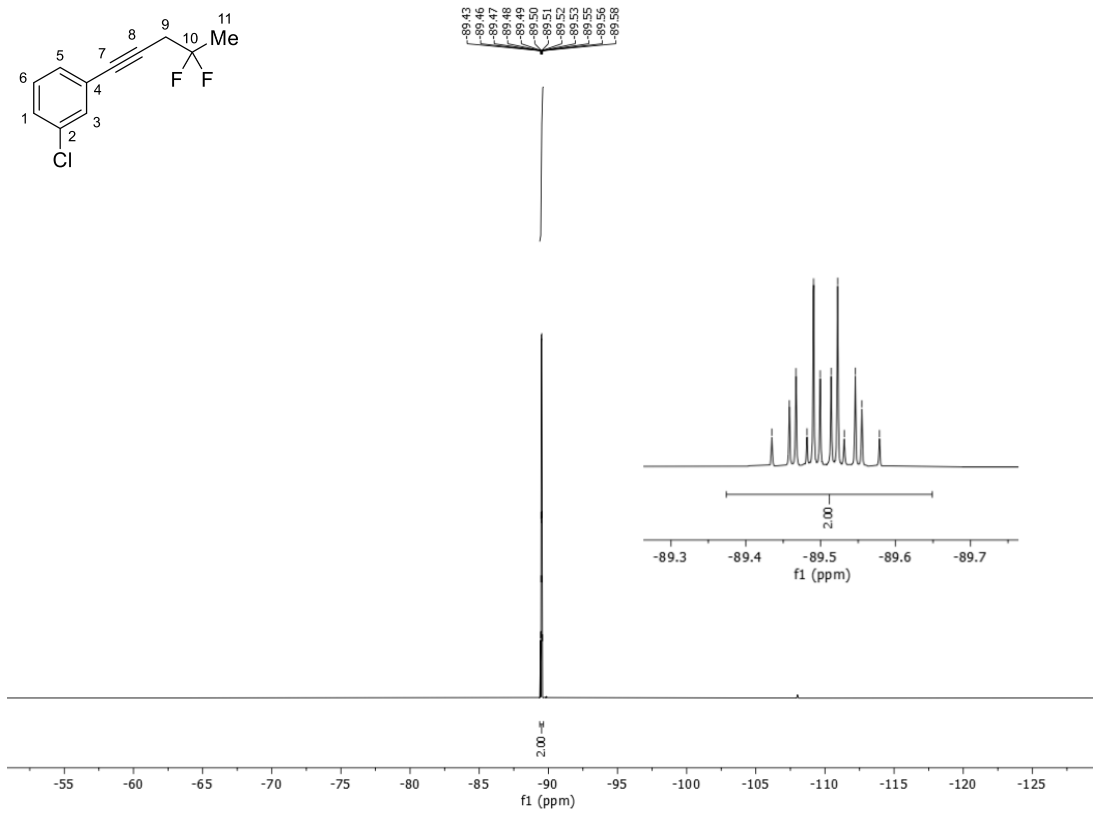

Supplementary Figure 97. ^{19}F NMR of **5** (470 MHz, 299 K, $CDCl_3$).

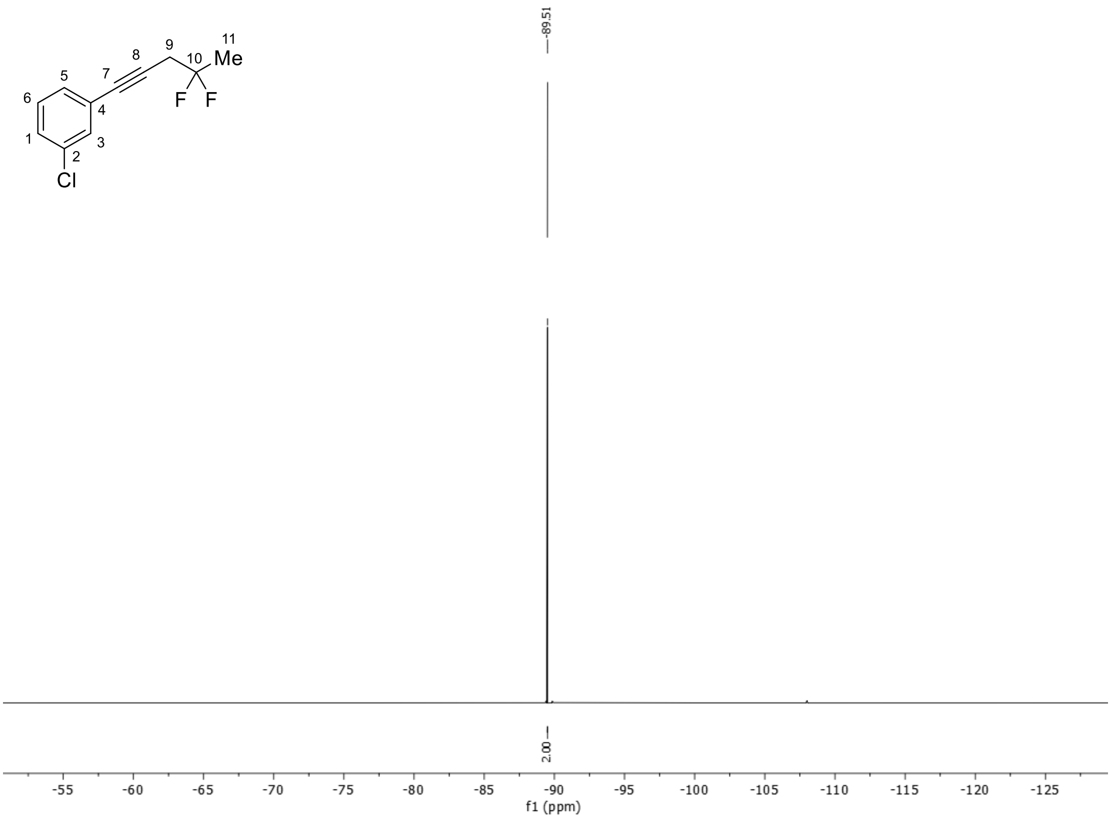

Supplementary Figure 98. ^{19}F{^{1}H} NMR of **5** (470 MHz, 299 K, $CDCl_3$).

1-Bromo-4-(4,4-difluoropent-1-yn-1-yl)benzene (6)

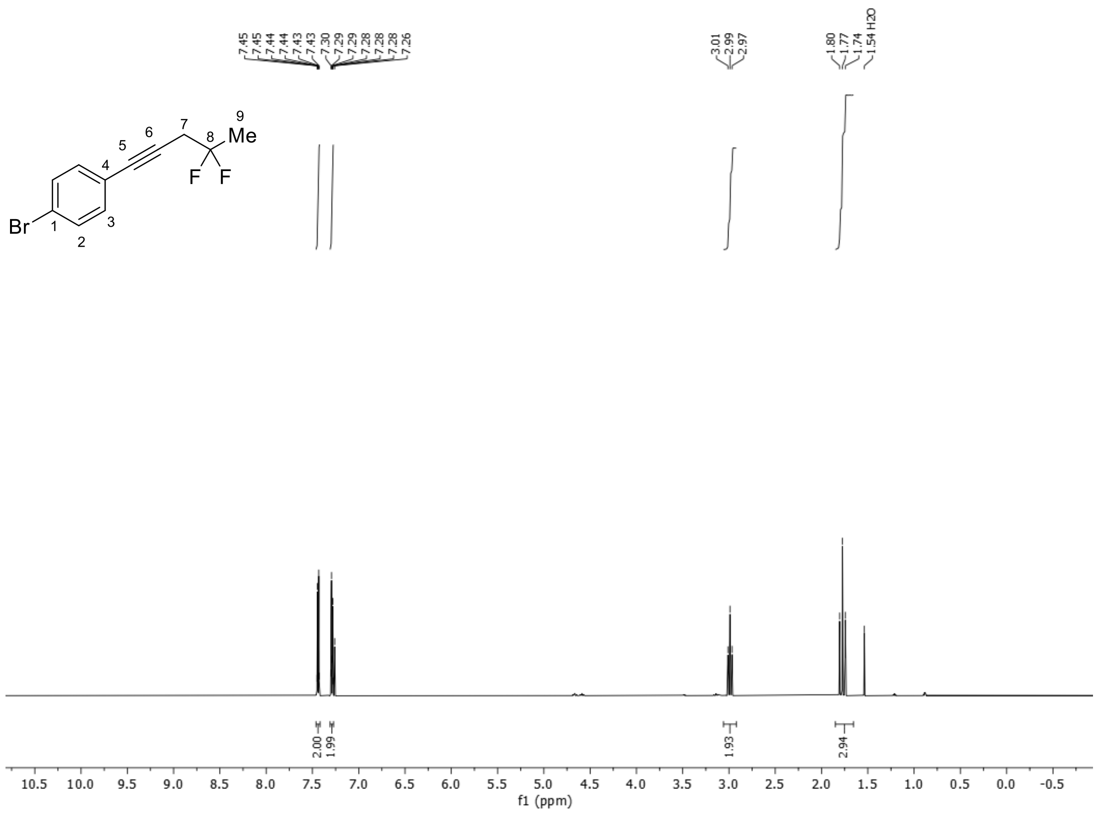

Supplementary Figure 99. ^{1}H NMR of **6** (500 MHz, 299 K, $CDCl_3$).

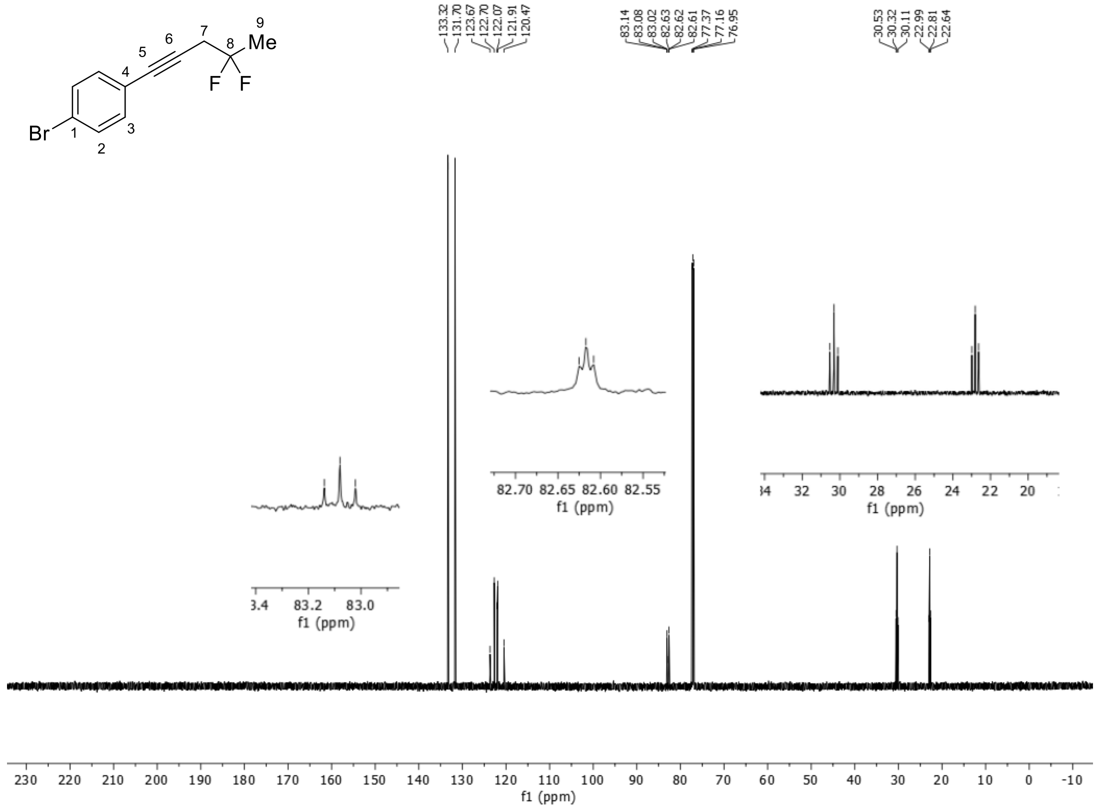

Supplementary Figure 100. $^{13}C\{^{1}H\}$ NMR of **6** (126 MHz, 299 K, $CDCl_3$).

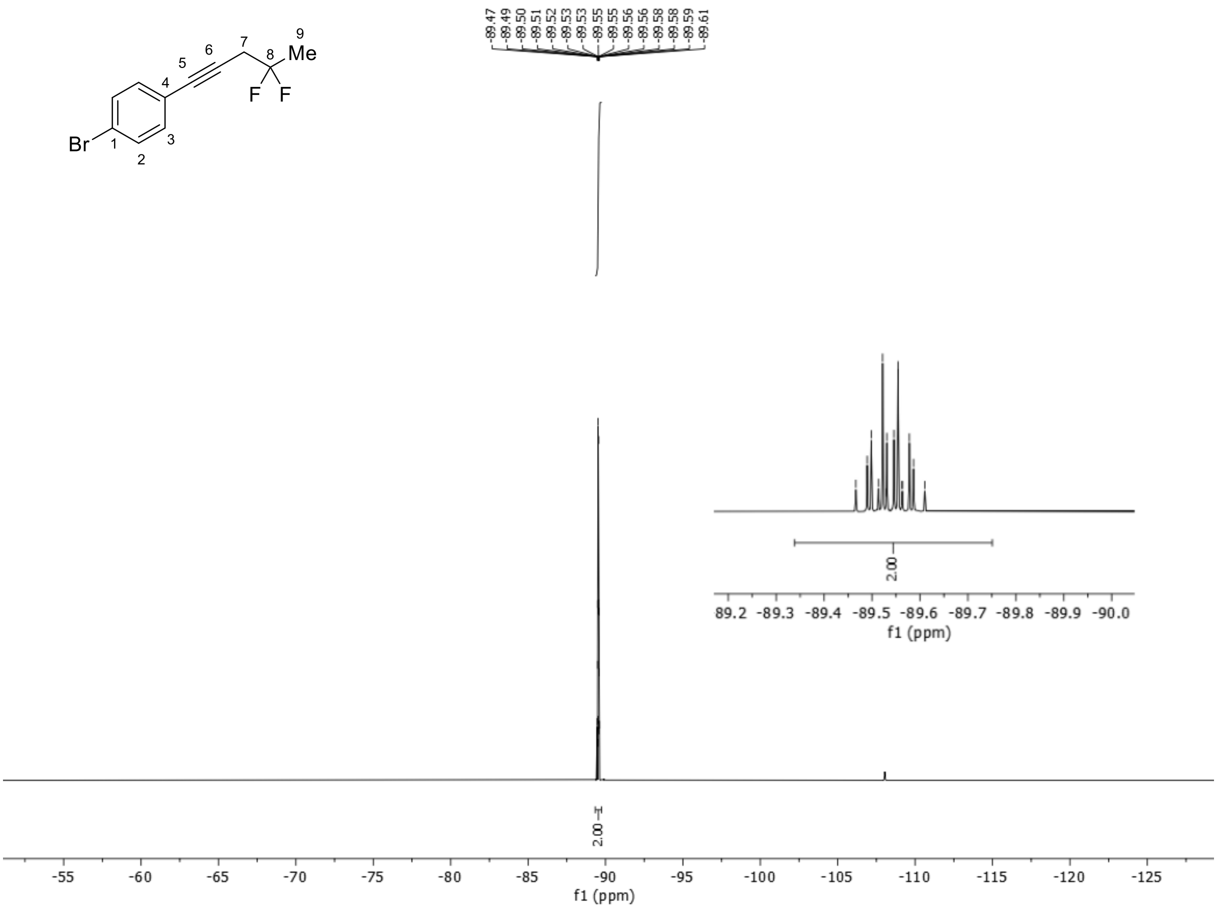

Supplementary Figure 101. ^{19}F NMR of **6** (470 MHz, 299 K, $CDCl_3$).

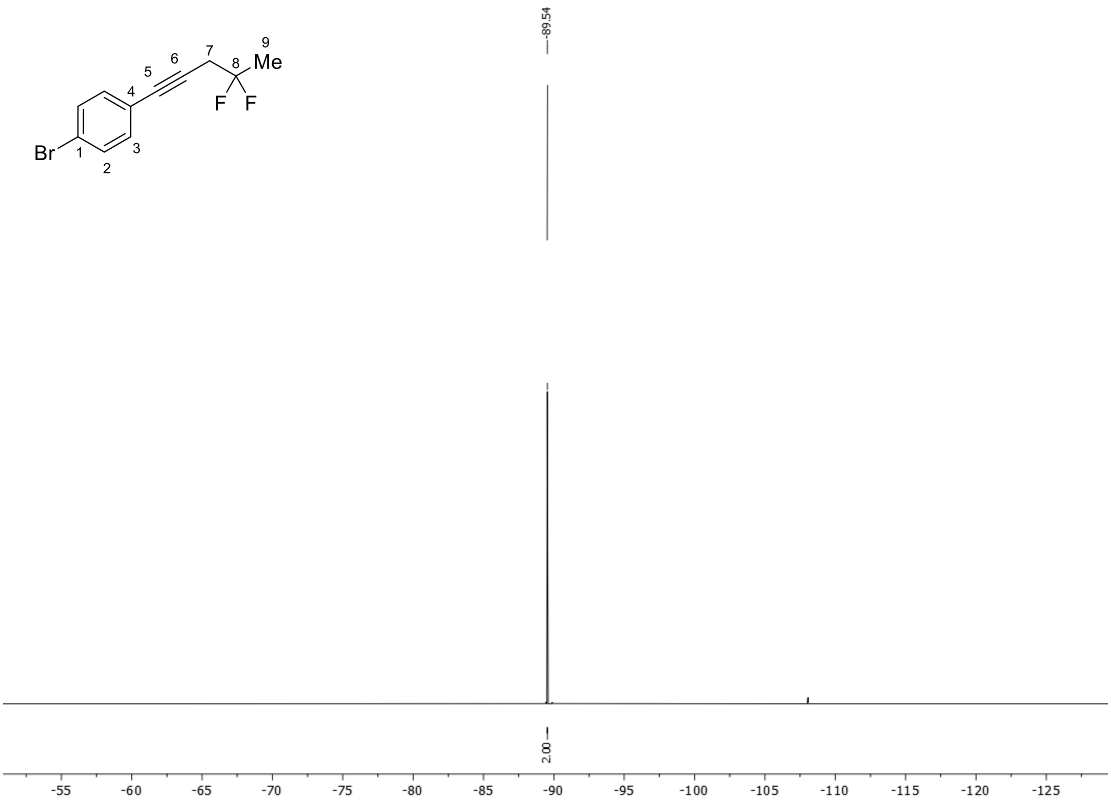

Supplementary Figure 102. $^{19}F\{^1H\}$ NMR of **6** (470 MHz, 299 K, $CDCl_3$).

Methyl 4-(4,4-difluoropent-1-yn-1-yl)benzoate (7)

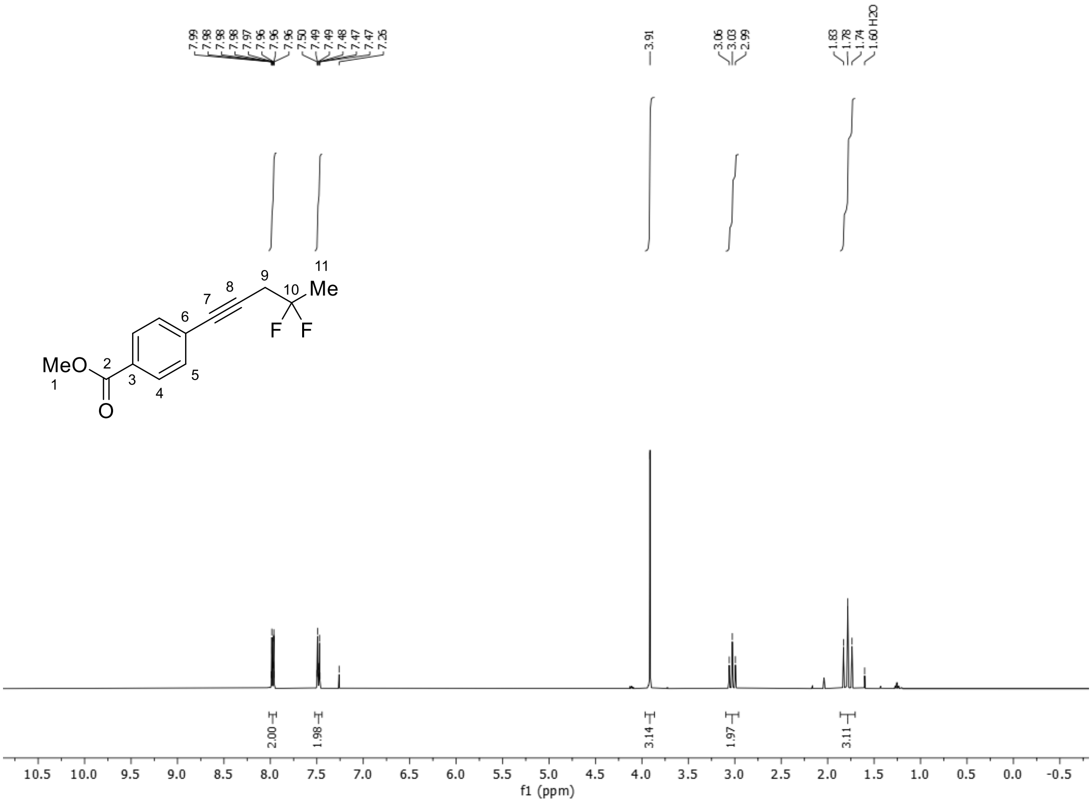

Supplementary Figure 103. ^{1}H NMR of **7** (600 MHz, 299 K, $CDCl_3$).

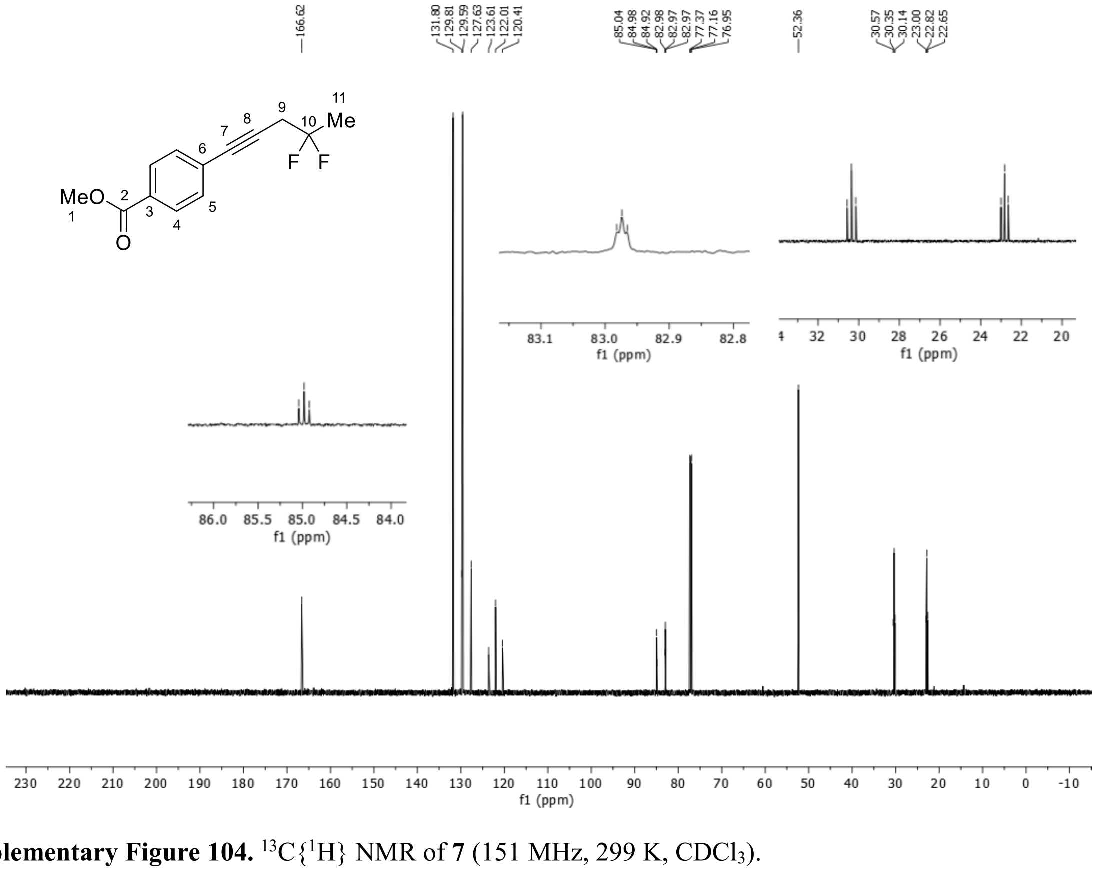

Supplementary Figure 104. $^{13}C\{^1H\}$ NMR of **7** (151 MHz, 299 K, $CDCl_3$).

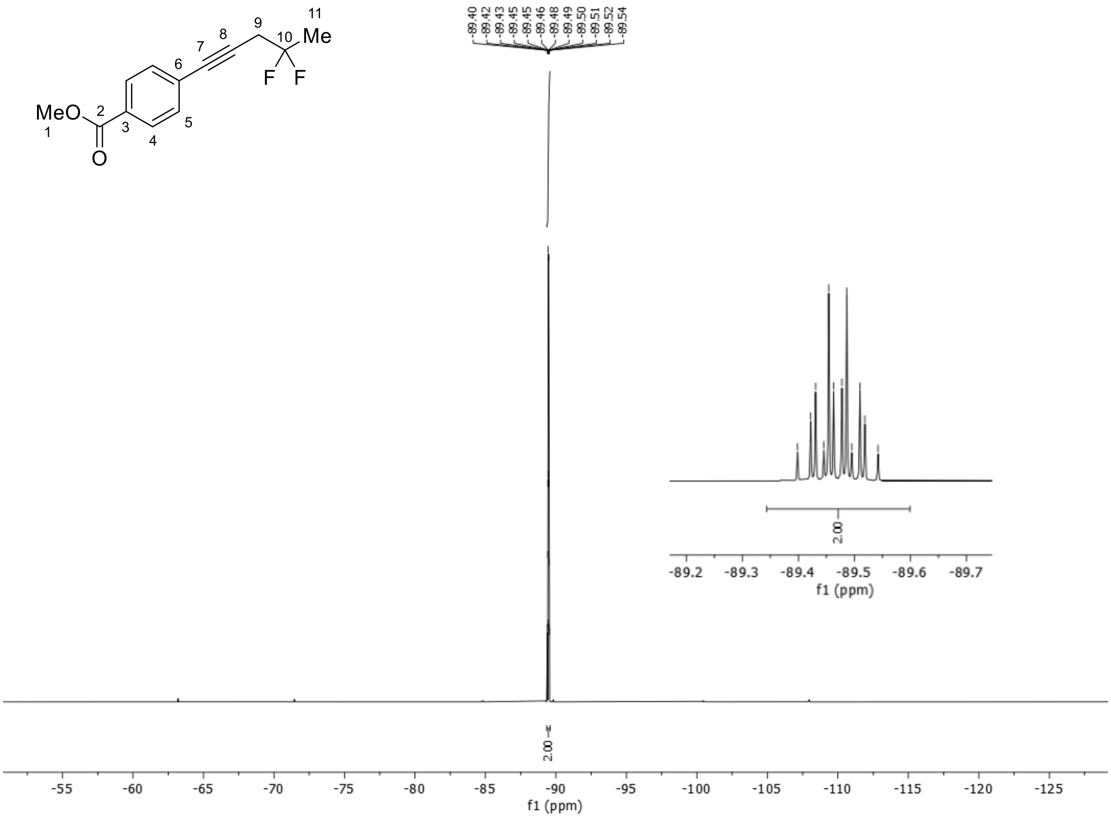

Supplementary Figure 105. ^{19}F NMR of **7** (470 MHz, 299 K, $CDCl_3$).

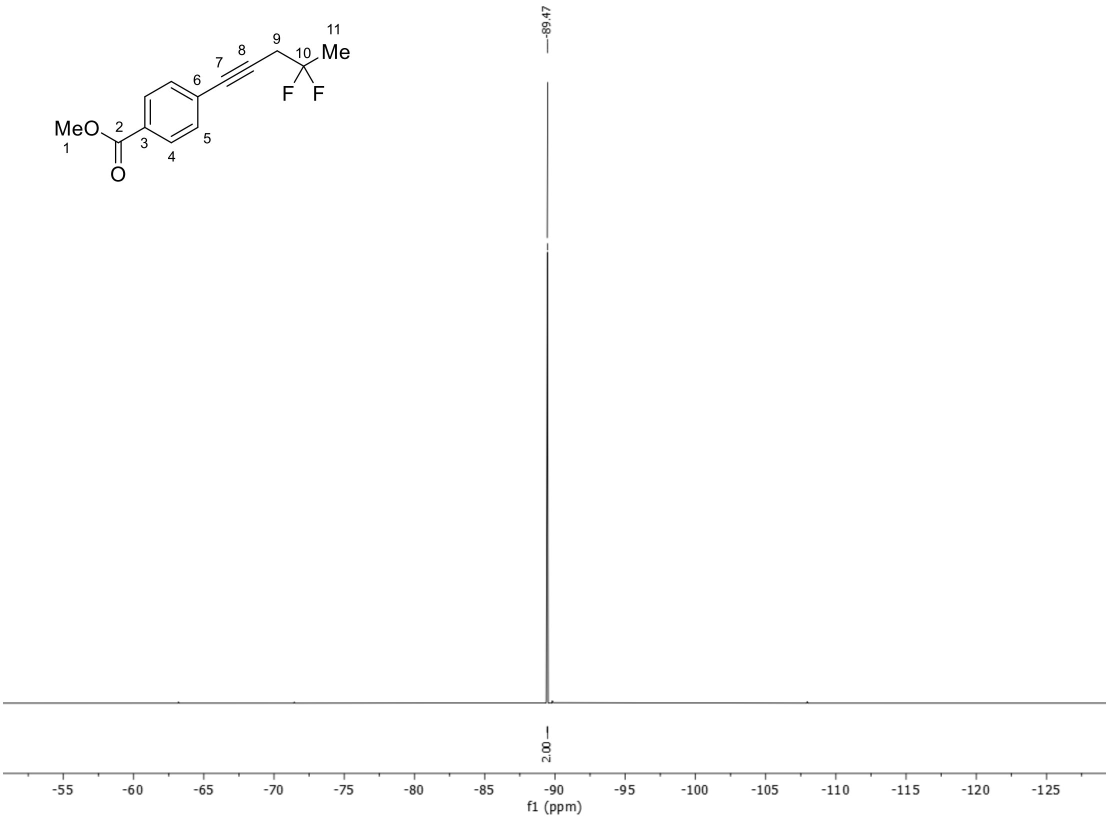

Supplementary Figure 106. $^{19}F\{^1H\}$ NMR of **7** (470 MHz, 299 K, $CDCl_3$).

1-(4,4-Difluoropent-1-yn-1-yl)-4-(methylsulfonyl)benzene (8)

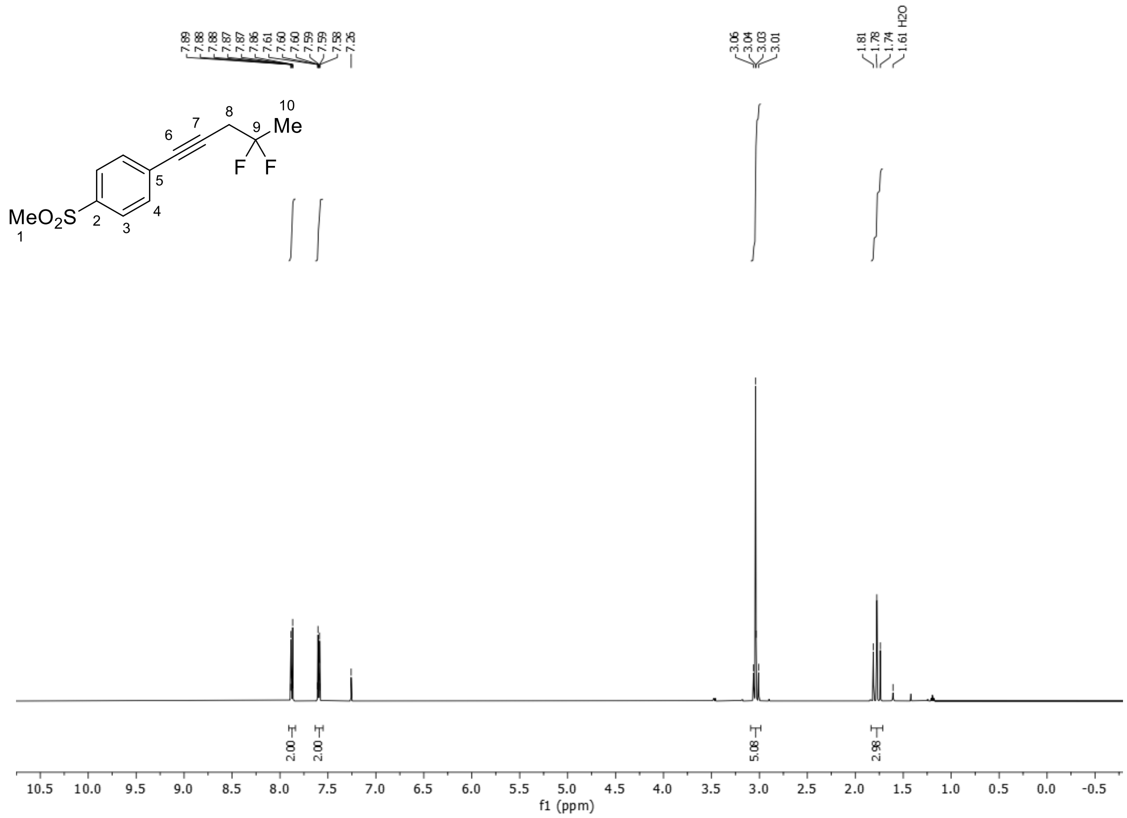

Supplementary Figure 107. ^{1}H NMR of **8** (500 MHz, 299 K, $CDCl_3$).

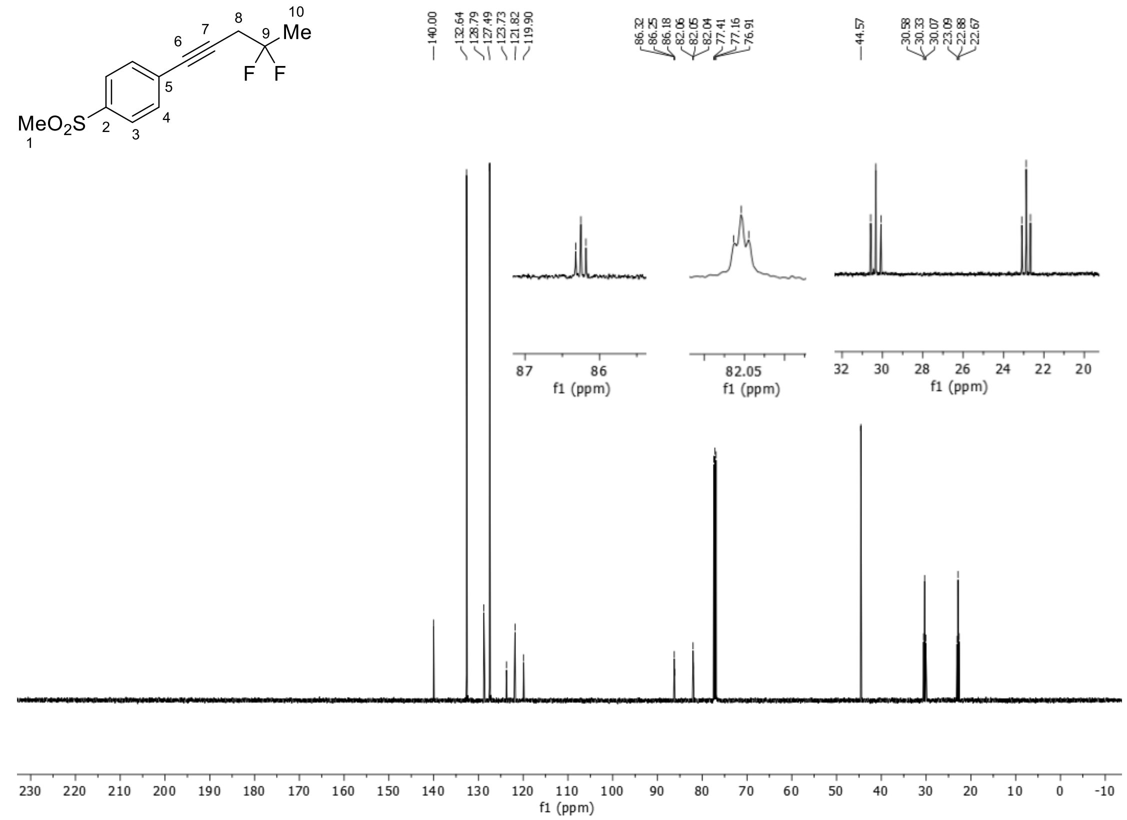

Supplementary Figure 108. $^{13}C\{^{1}H\}$ NMR of **8** (126 MHz, 299 K, $CDCl_3$).

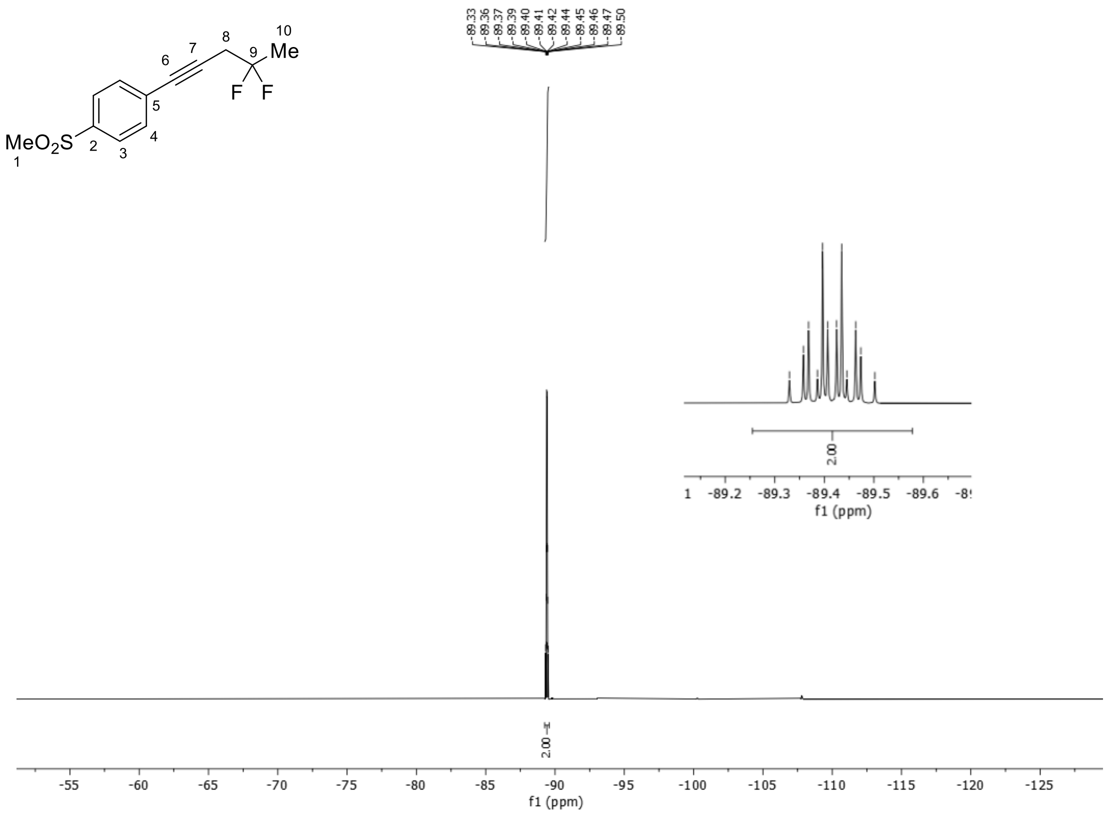

Supplementary Figure 109. ^{19}F NMR of **8** (470 MHz, 299 K, $CDCl_3$).

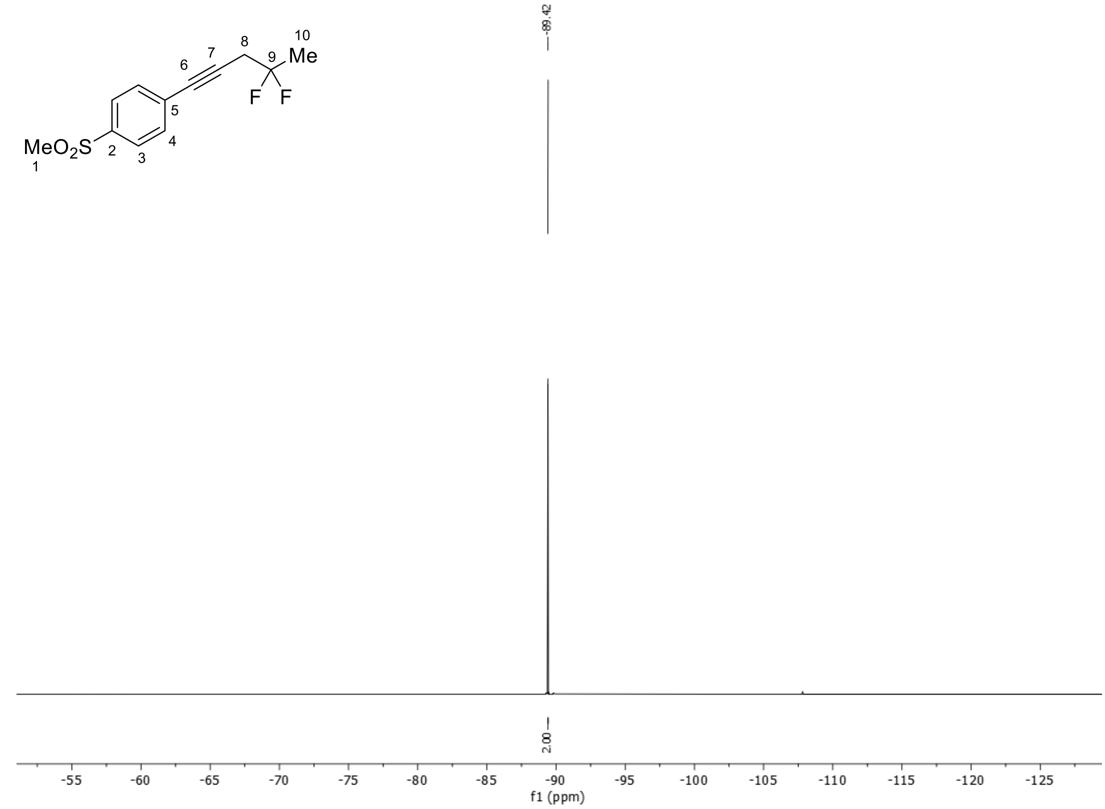

Supplementary Figure 110. $^{19}F\{^{1}H\}$ NMR of **8** (470 MHz, 299 K, $CDCl_3$).

1-(4-(4,4-Difluoropent-1-yn-1-yl)phenyl)-2,2,2-trifluoroethan-1-one (9)

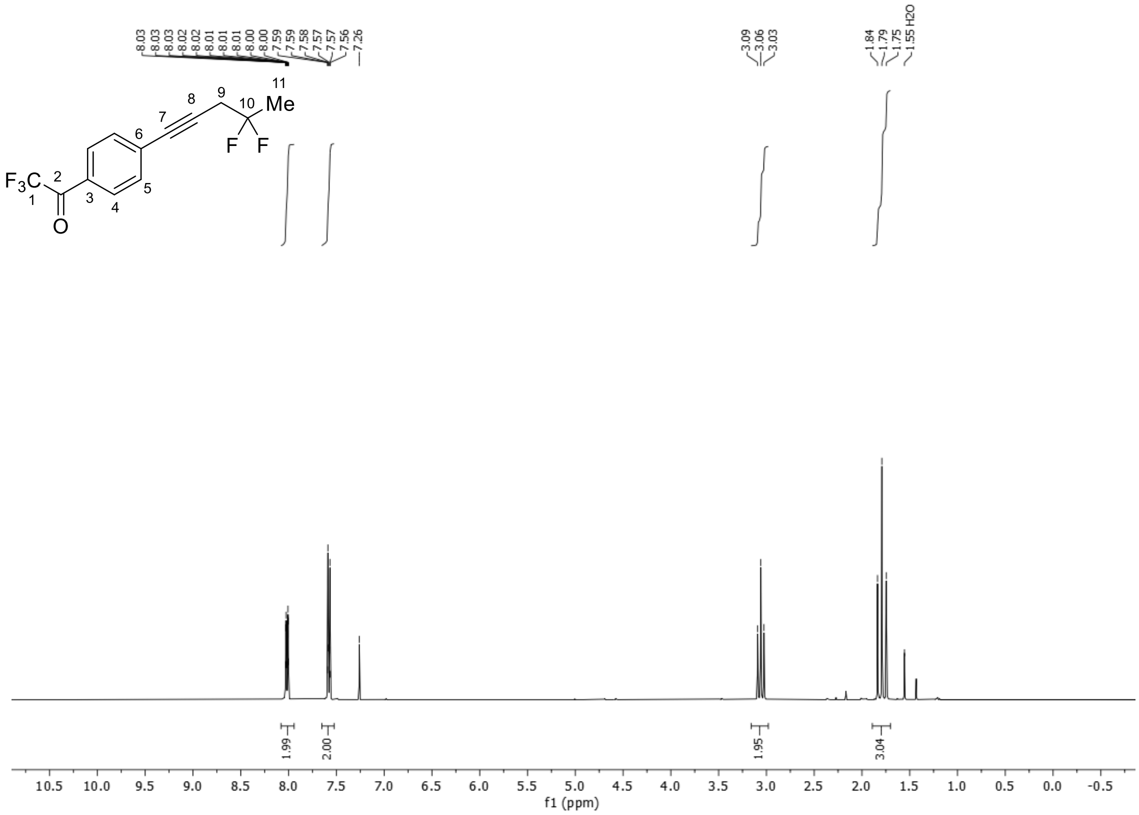

Supplementary Figure 111. ^{1}H NMR of **9** (400 MHz, 299 K, $CDCl_3$).

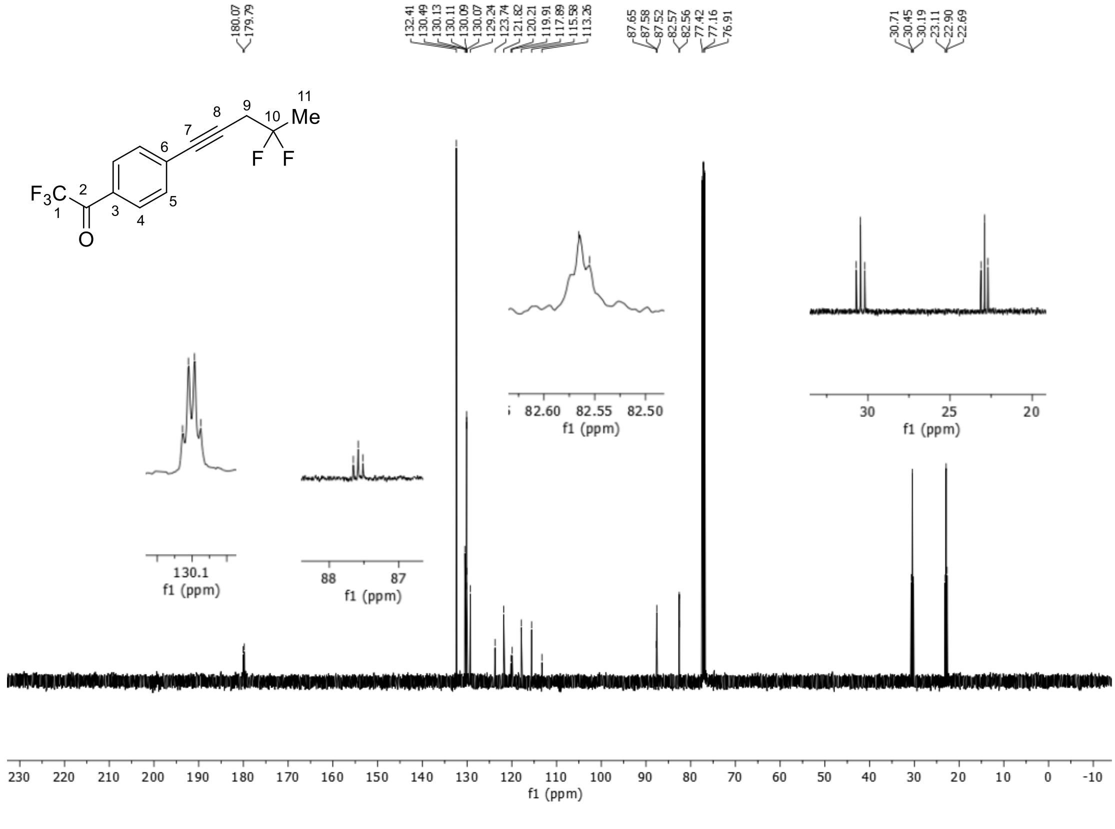

Supplementary Figure 112. $^{13}C\{^{1}H\}$ NMR of **9** (126 MHz, 299 K, $CDCl_3$).

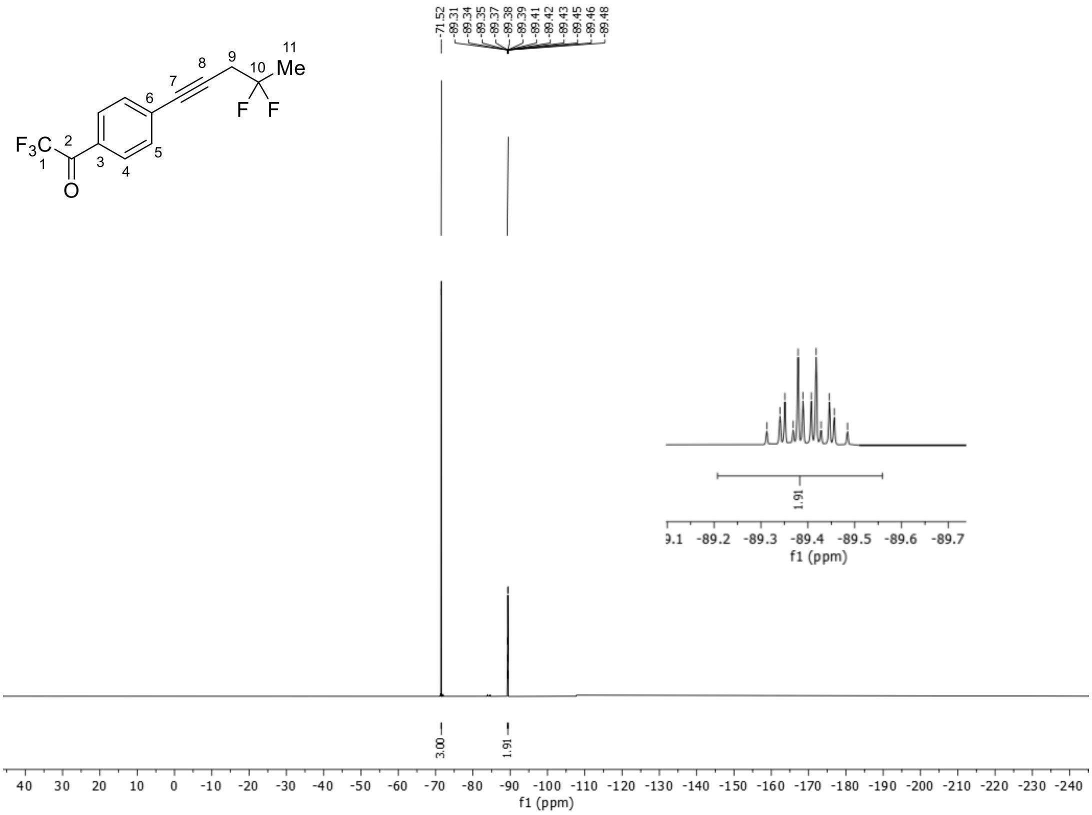

Supplementary Figure 113. ^{19}F NMR of **9** (470 MHz, 299 K, $CDCl_3$).

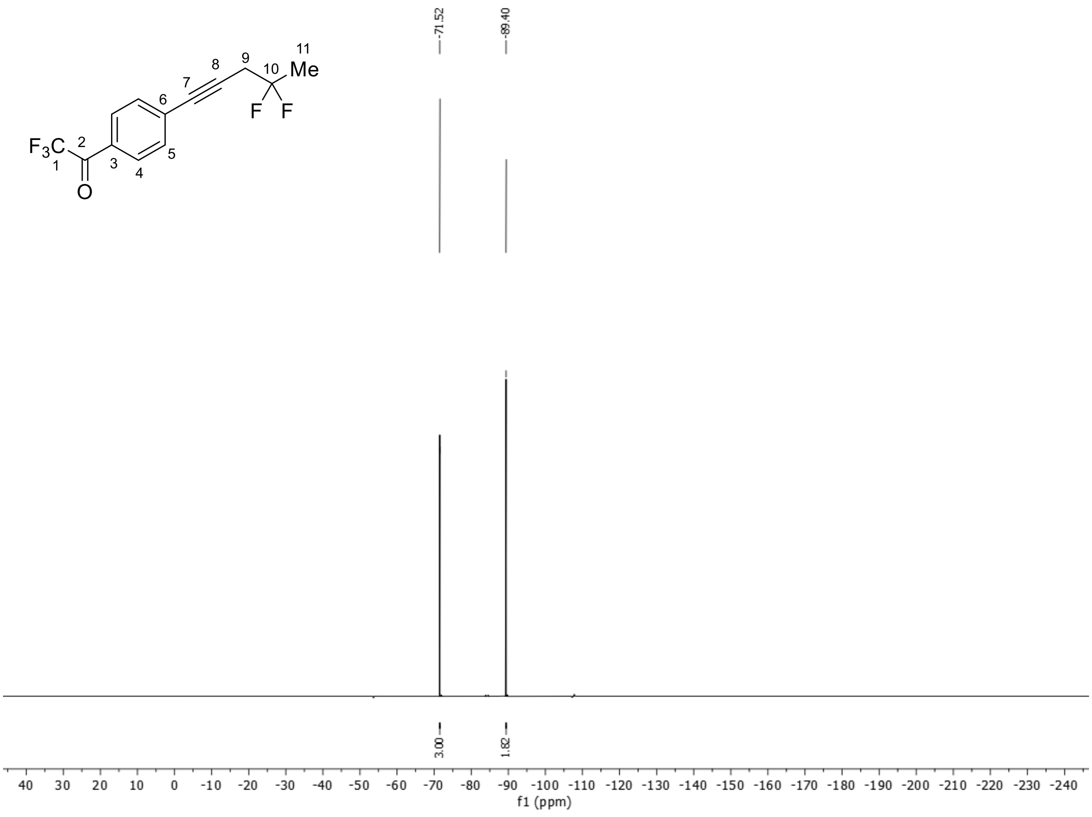

Supplementary Figure 114. ^{19}F{^{1}H} NMR of **9** (470 MHz, 299 K, $CDCl_3$).

1-(4,4-Difluoropent-1-yn-1-yl)-3-methylbenzene (10)

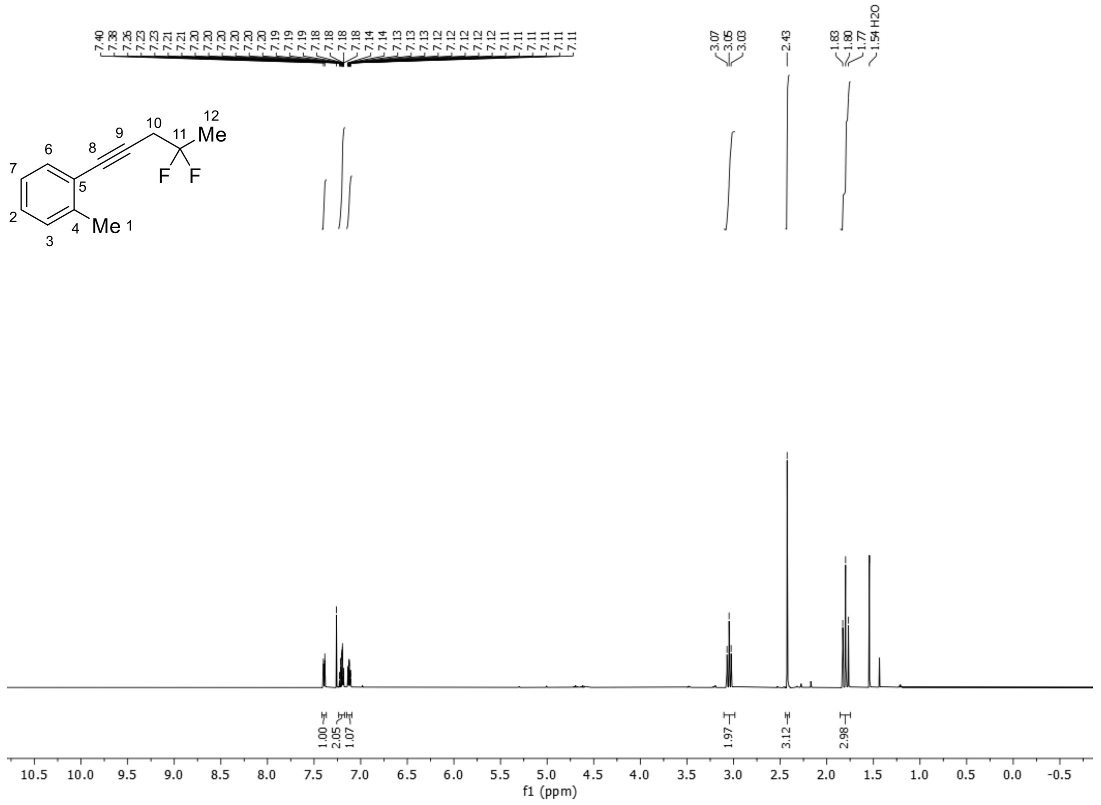

Supplementary Figure 115. ^{1}H NMR of **10** (500 MHz, 299 K, $CDCl_3$).

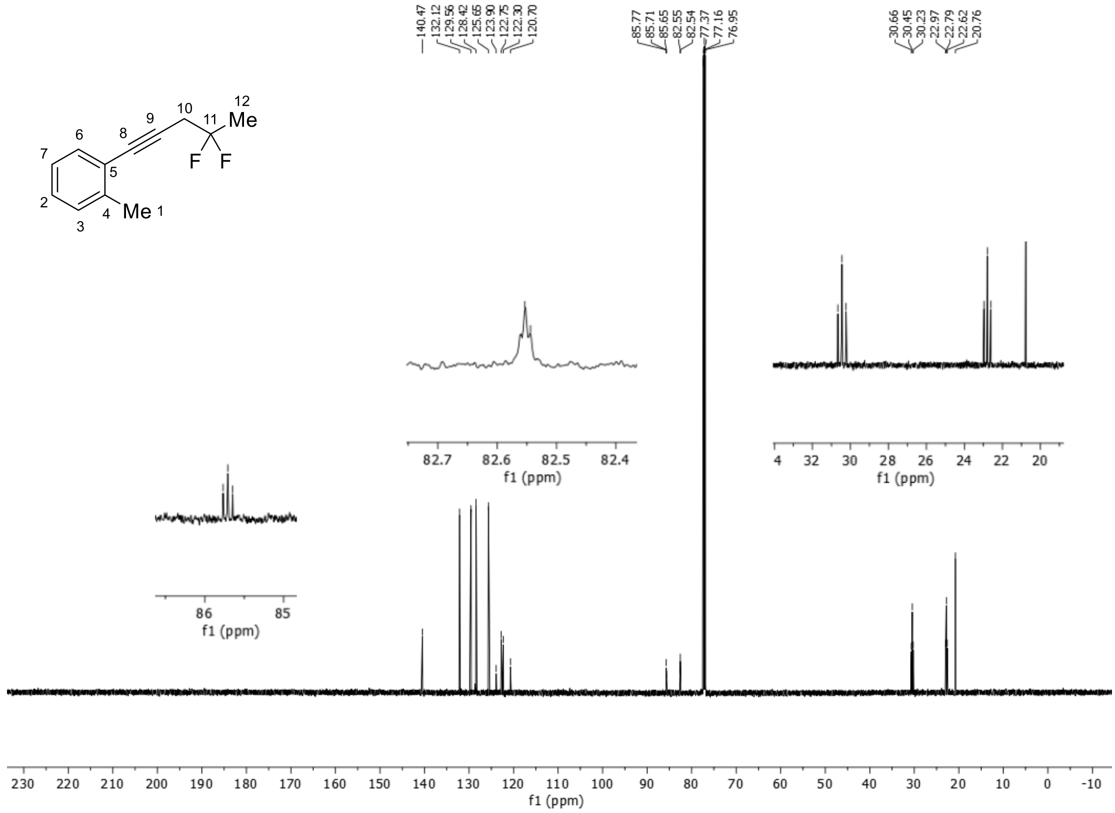

Supplementary Figure 116. $^{13}C\{^{1}H\}$ NMR of **10** (126 MHz, 299 K, $CDCl_3$).

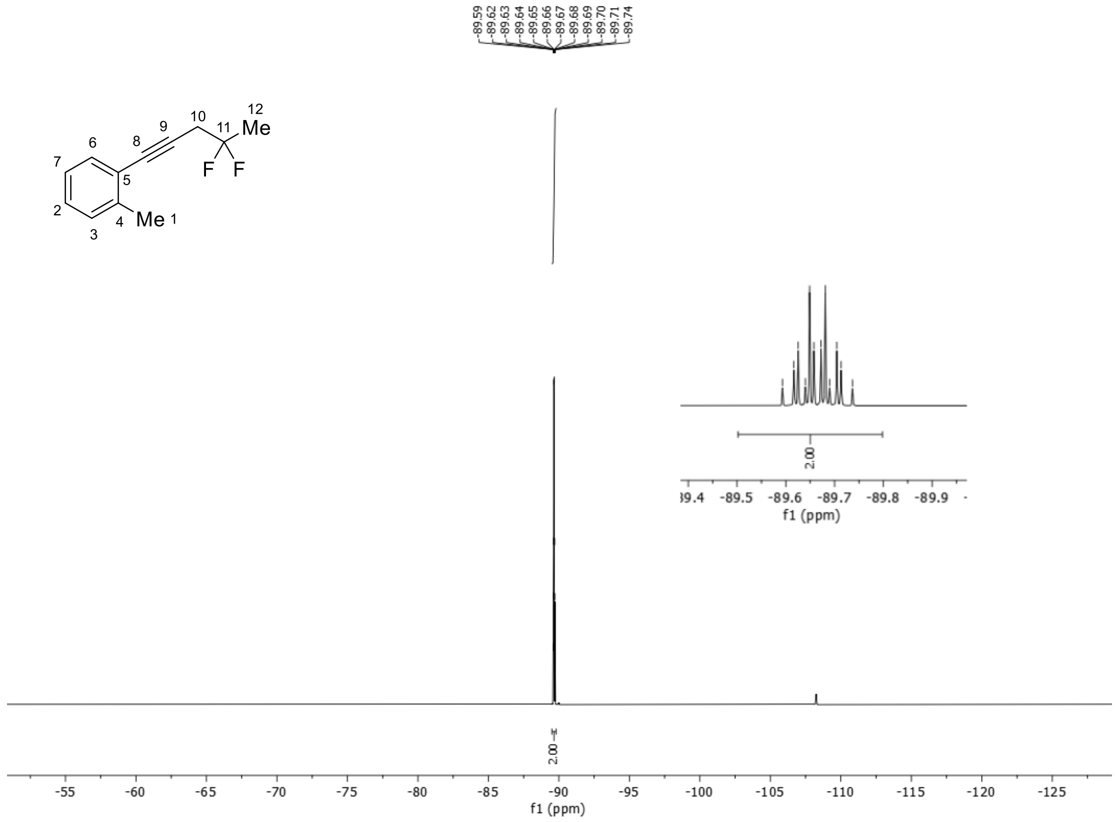

Supplementary Figure 117. ^{19}F NMR of **10** (470 MHz, 299 K, $CDCl_3$).

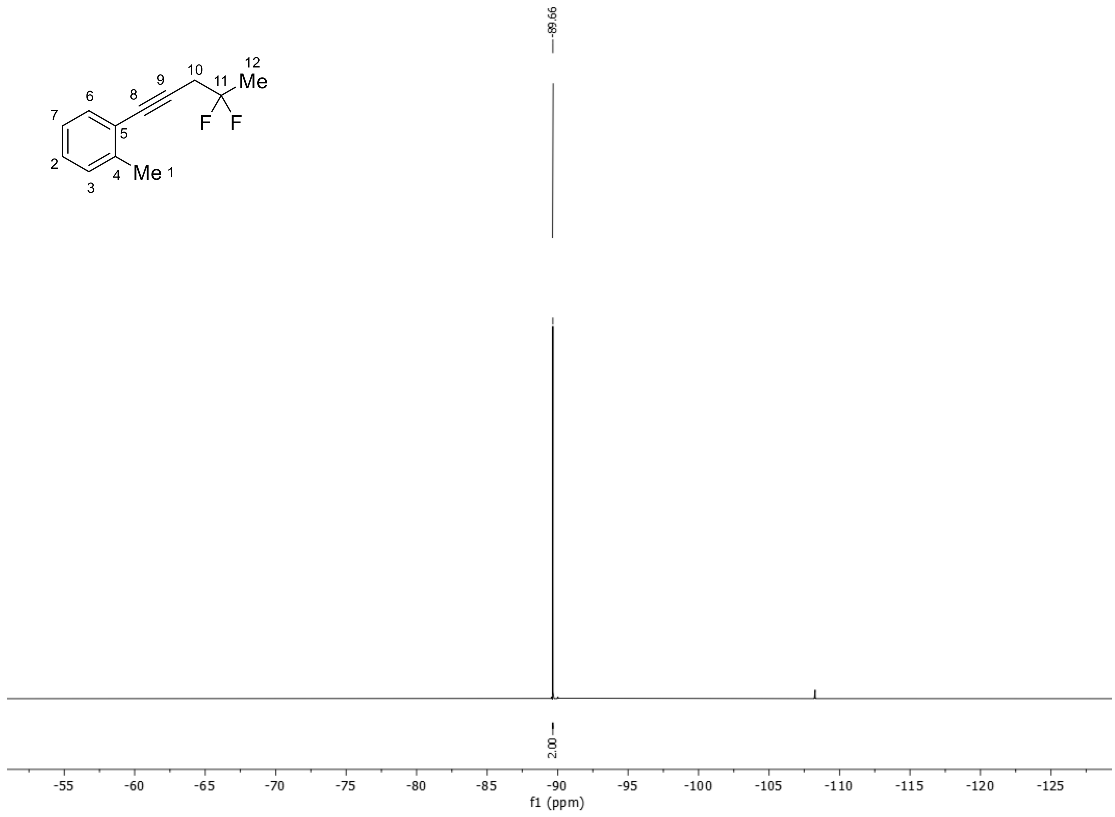

Supplementary Figure 118. ^{19}F{^{1}H} NMR of **10** (470 MHz, 299 K, $CDCl_3$).

1-(3,4-difluoro-3-methylbut-1-yn-1-yl)-3-methylbenzene (10')

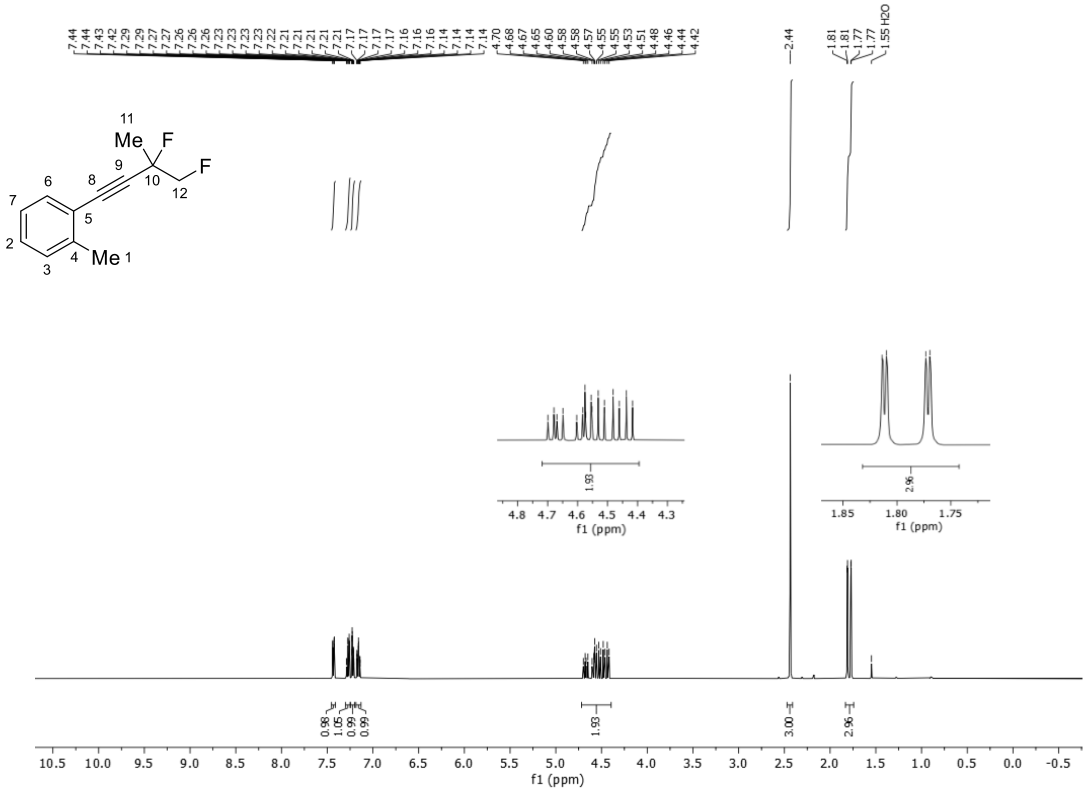

Supplementary Figure 119. ^{1}H NMR of **10'** (500 MHz, 299 K, $CDCl_3$).

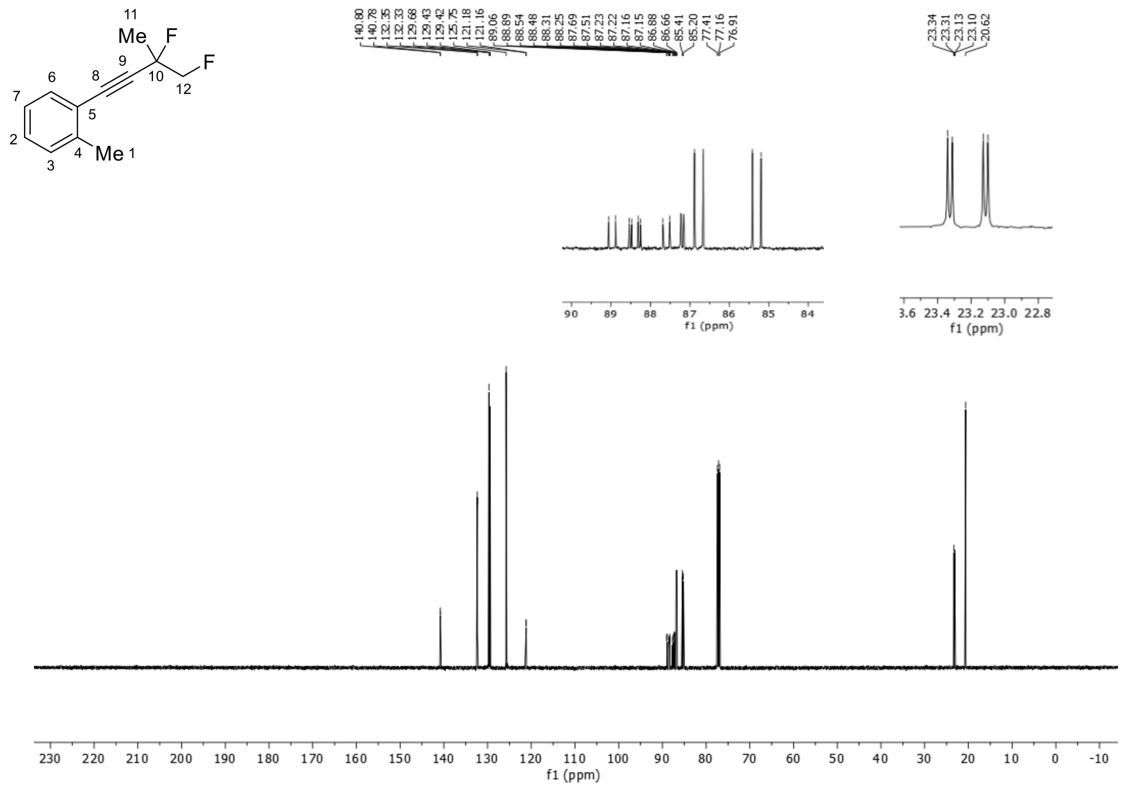

Supplementary Figure 120. $^{13}C\{^{1}H\}$ NMR of **10'** (126 MHz, 299 K, $CDCl_3$).

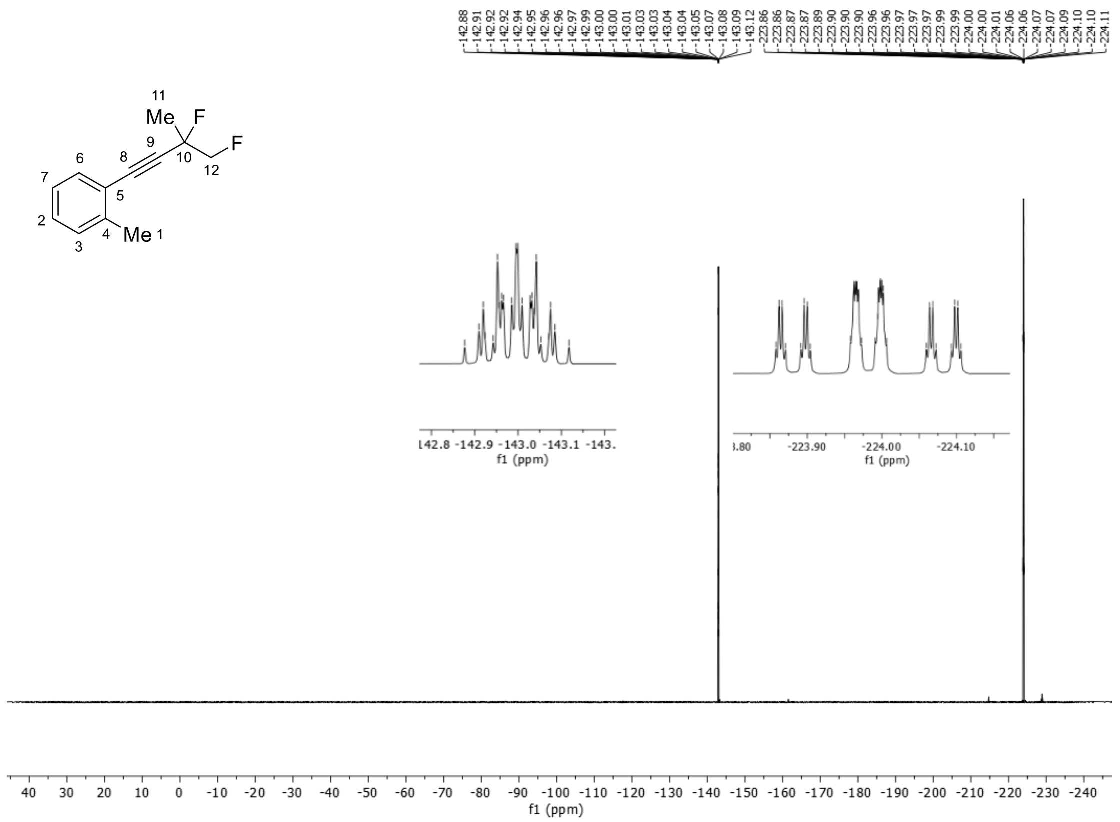

Supplementary Figure 121. ^{19}F NMR of **10'** (470 MHz, 299 K, $CDCl_3$).

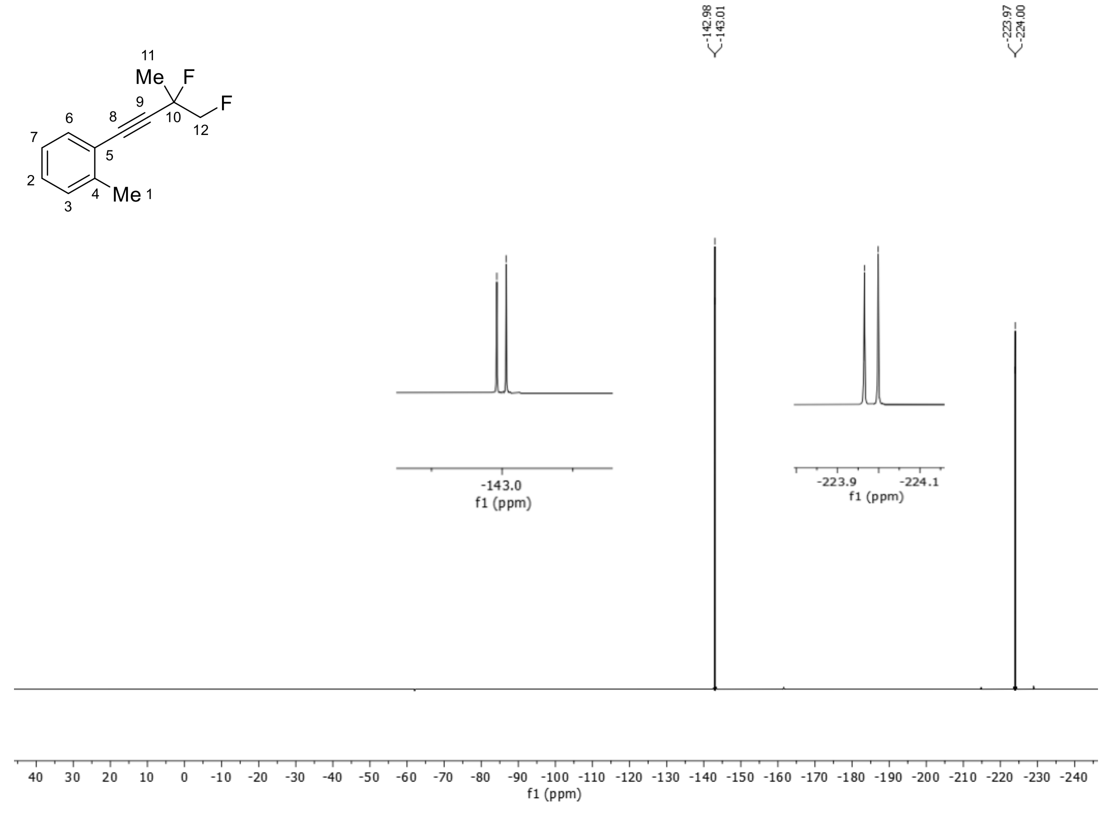

Supplementary Figure 122. $^{19}F\{^1H\}$ NMR of **10'** (470 MHz, 299 K, $CDCl_3$).

3-(4,4-Difluoropent-1-yn-1-yl)pyridine (11)

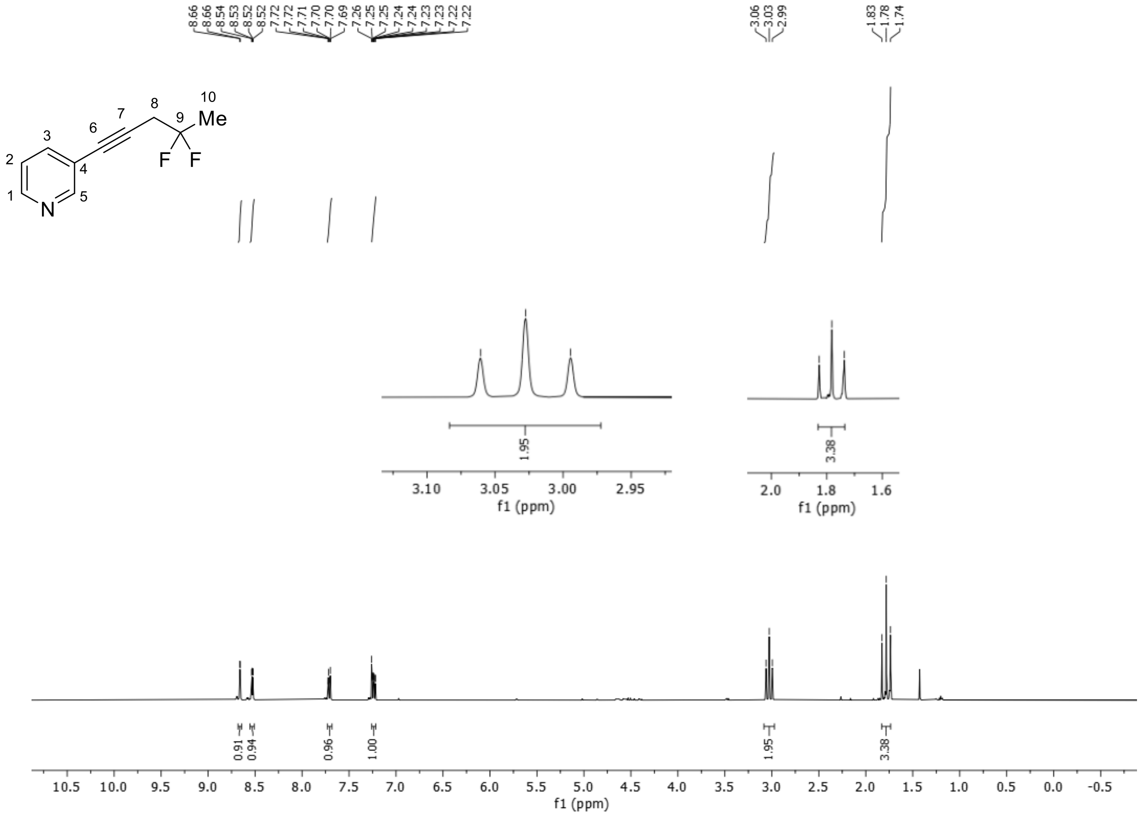

Supplementary Figure 123. ^{1}H NMR of **11** (400 MHz, 299 K, $CDCl_3$).

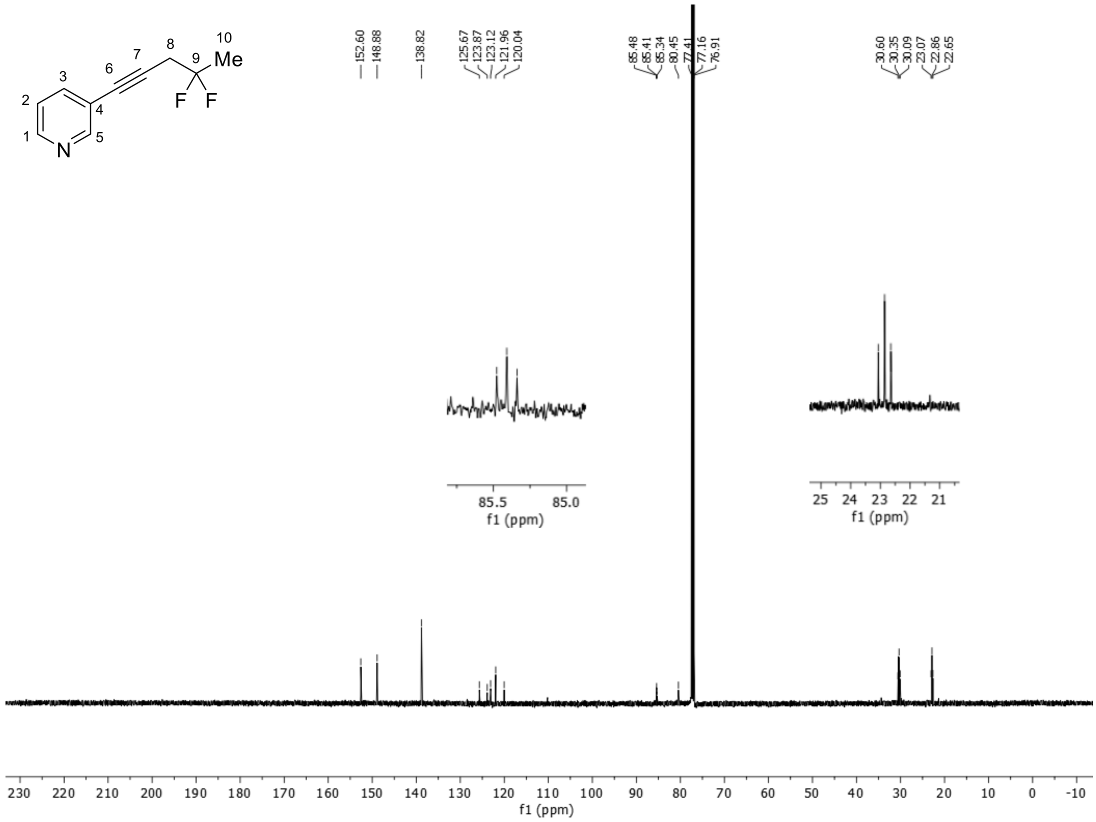

Supplementary Figure 124. $^{13}C\{^{1}H\}$ NMR of **11** (126 MHz, 299 K, $CDCl_3$).

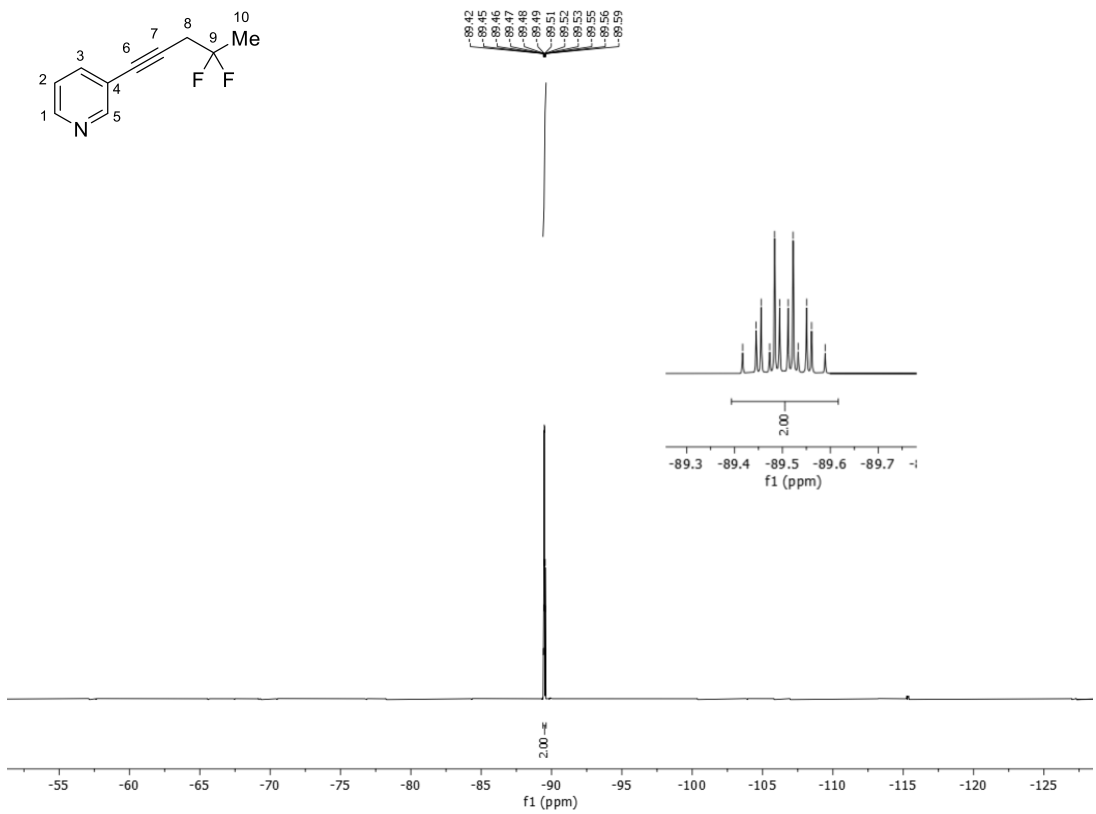

Supplementary Figure 125. ^{19}F NMR of **11** (470 MHz, 299 K, $CDCl_3$).

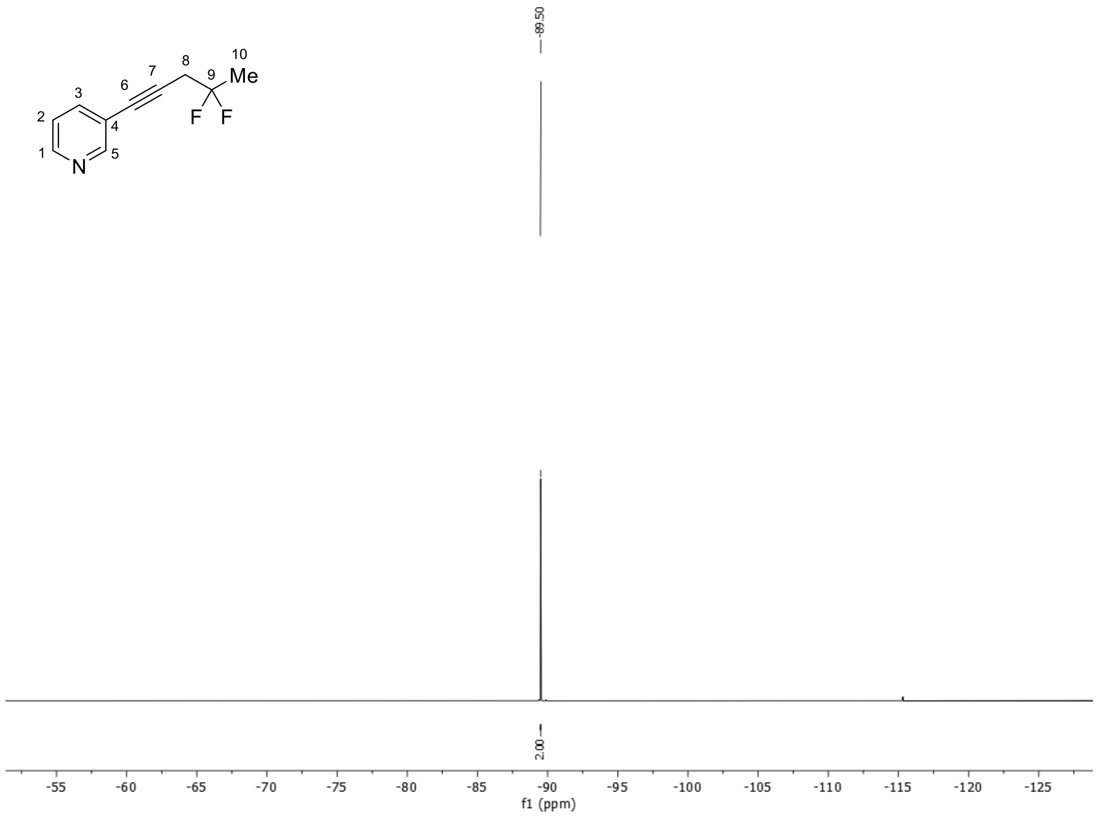

Supplementary Figure 126. ^{19}F{^{1}H} NMR of **11** (470 MHz, 299 K, $CDCl_3$).

6-(4,4-Difluoropent-1-yn-1-yl)quinoline (12)

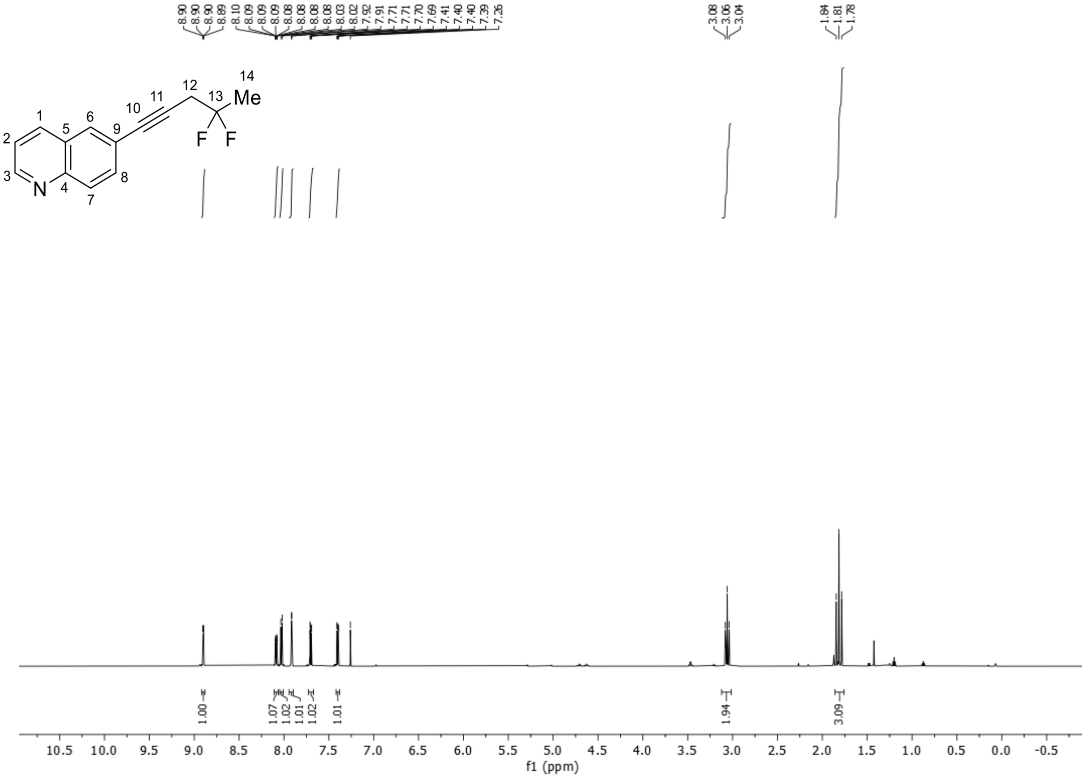

Supplementary Figure 127. ^{1}H NMR of **12** (600 MHz, 299 K, $CDCl_3$).

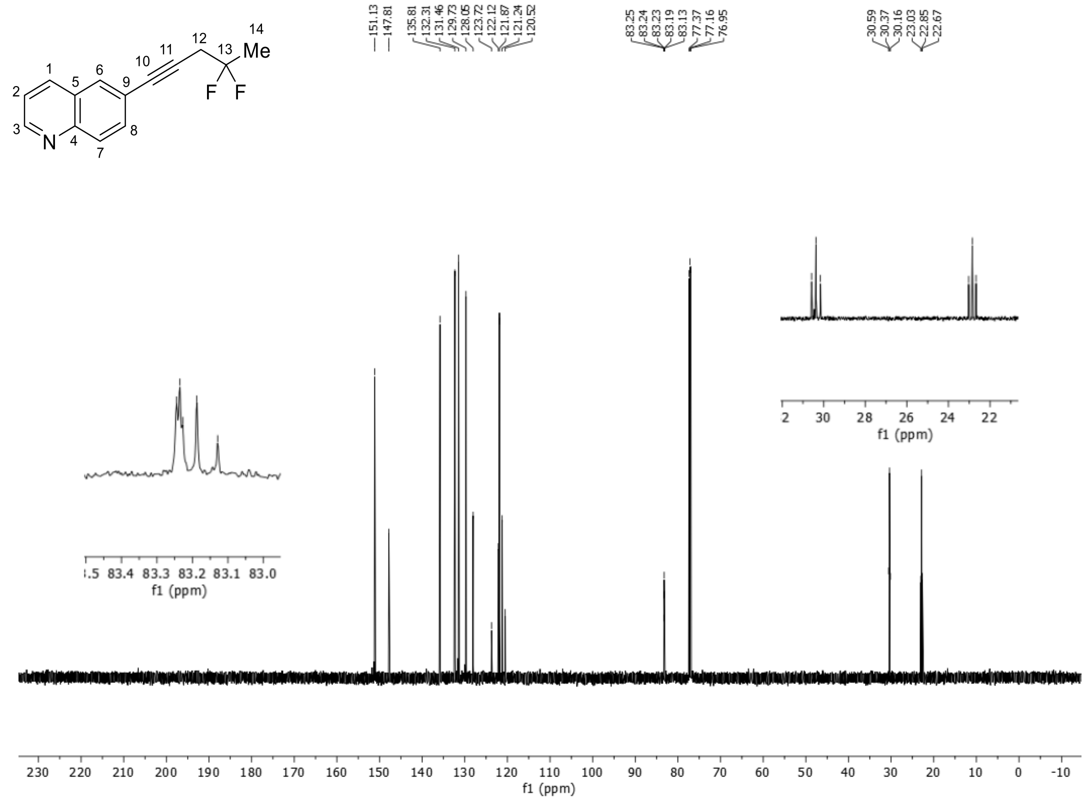

Supplementary Figure 128. $^{13}C\{^{1}H\}$ NMR of **12** (151 MHz, 299 K, $CDCl_3$).

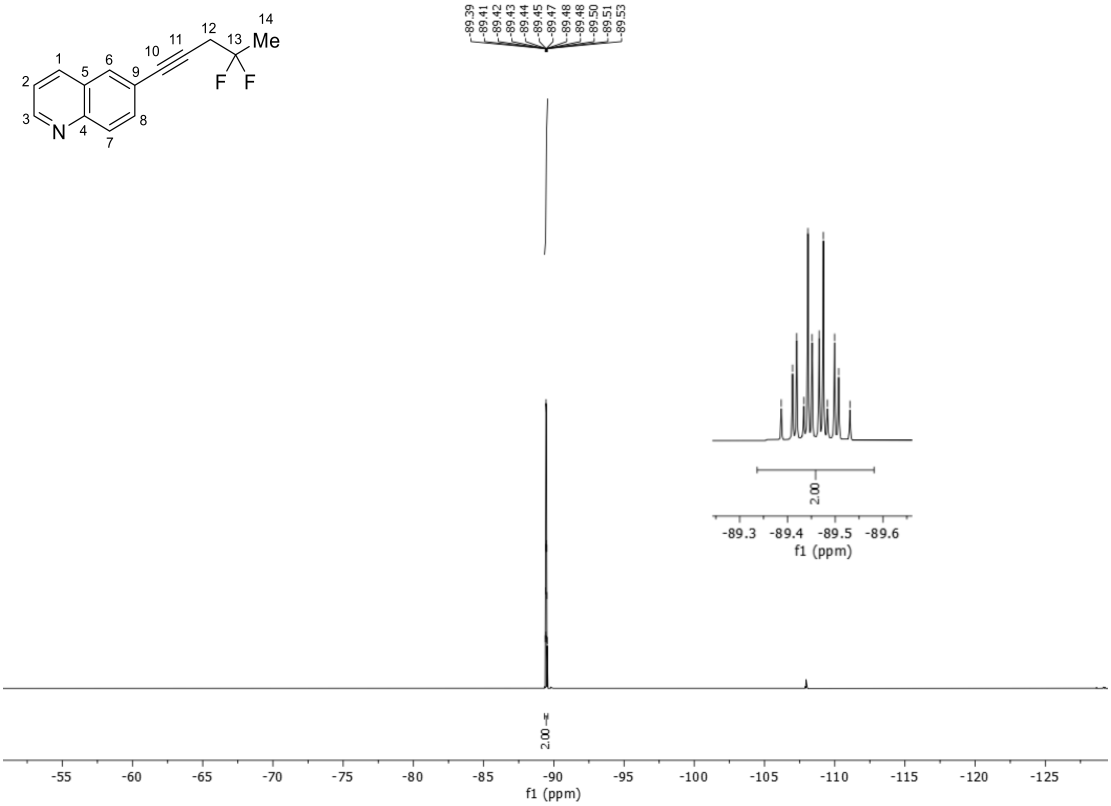

Supplementary Figure 129. ^{19}F NMR of **12** (564 MHz, 299 K, $CDCl_3$).

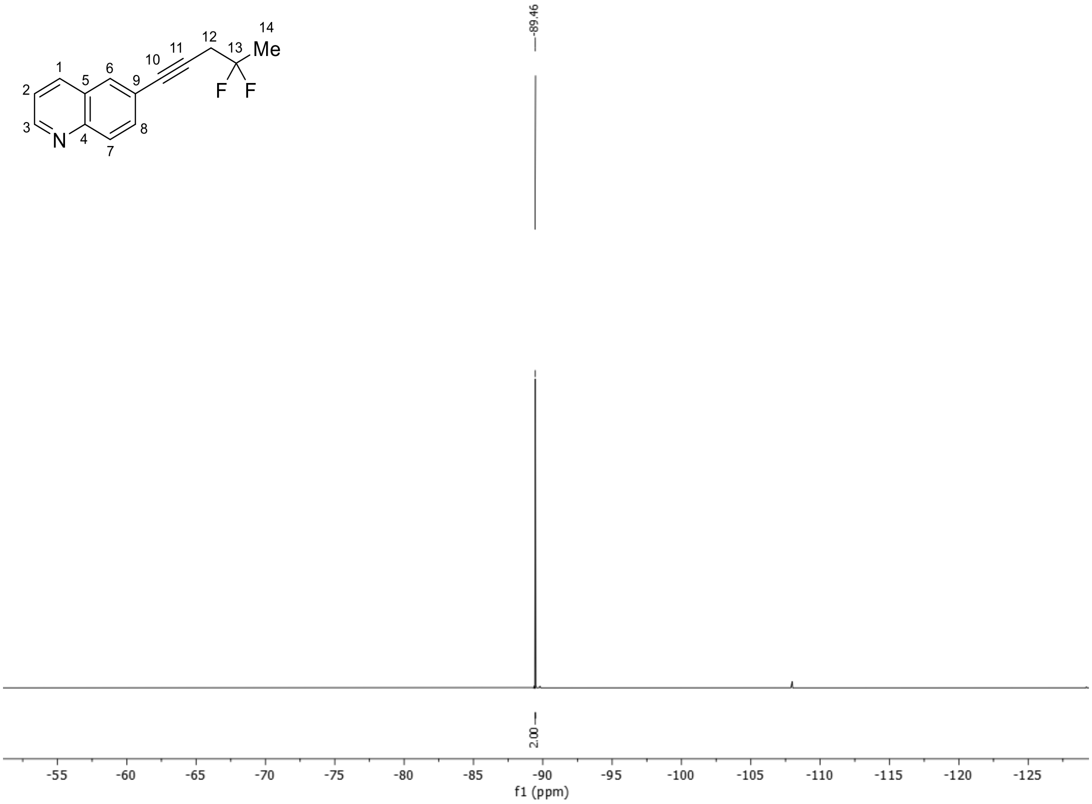

Supplementary Figure 130. ^{19}F{^{1}H} NMR of **12** (564 MHz, 299 K, $CDCl_3$).

(4-(4,4-Difluoropent-1-yn-1-yl)phenyl)(morpholino)methanone (13)

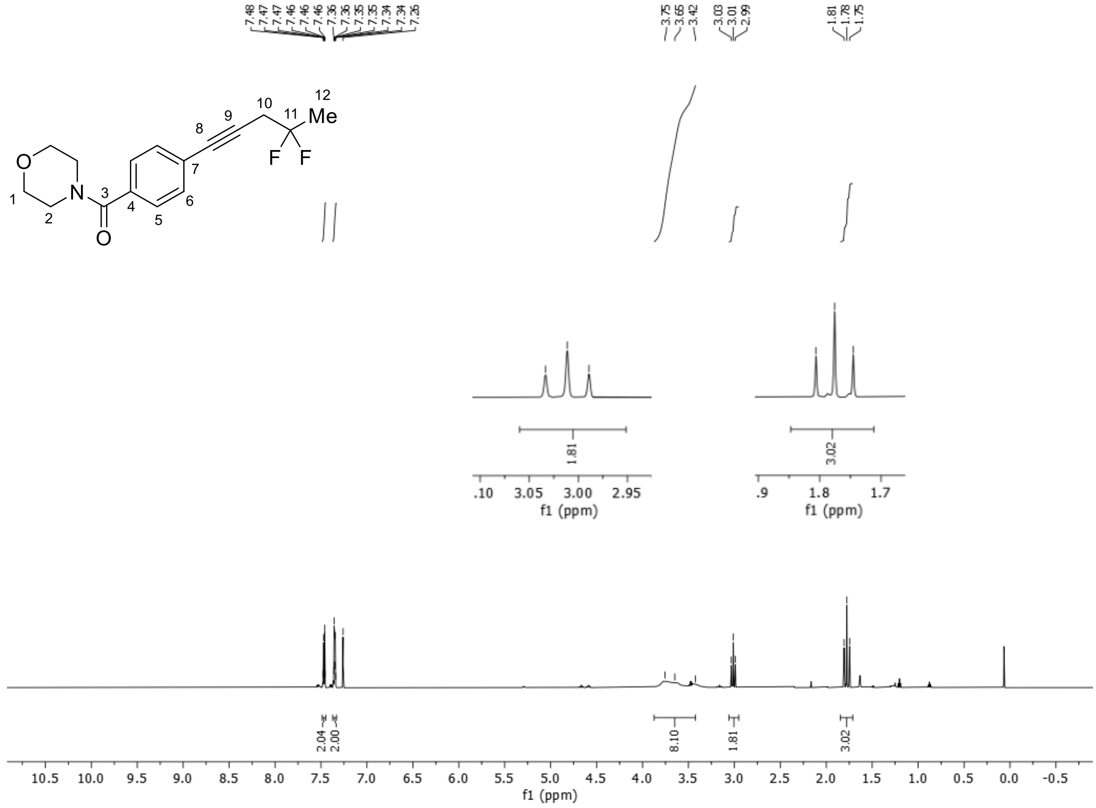

Supplementary Figure 131. ^{1}H NMR of **13** (599 MHz, 299 K, $CDCl_3$).

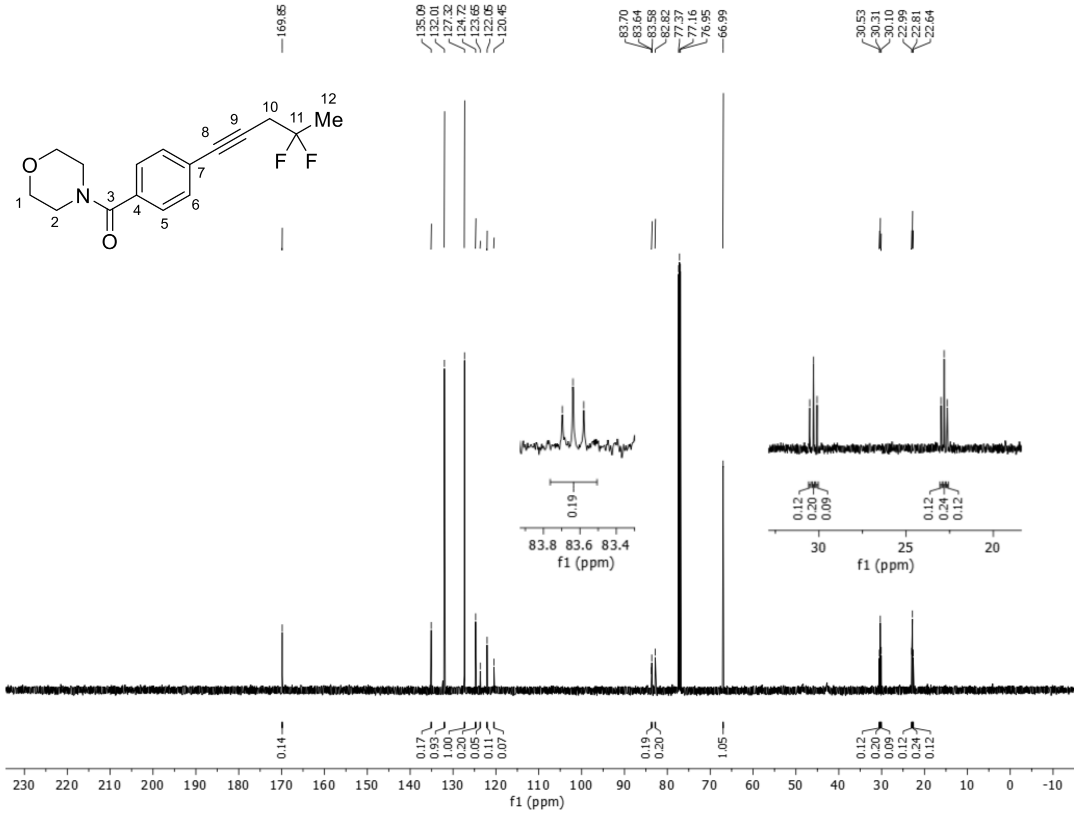

Supplementary Figure 132. $^{13}C\{^{1}H\}$ NMR of **13** (151 MHz, 299 K, $CDCl_3$).

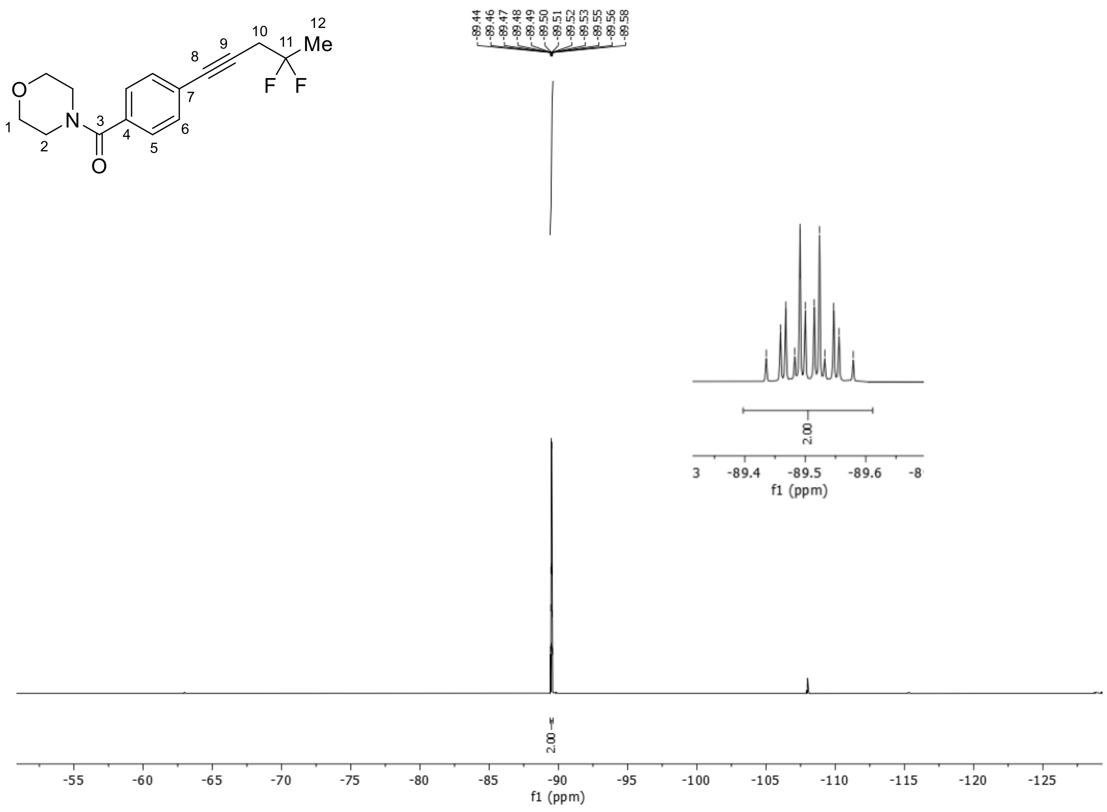

Supplementary Figure 133. ^{19}F NMR of **13** (564 MHz, 299 K, $CDCl_3$).

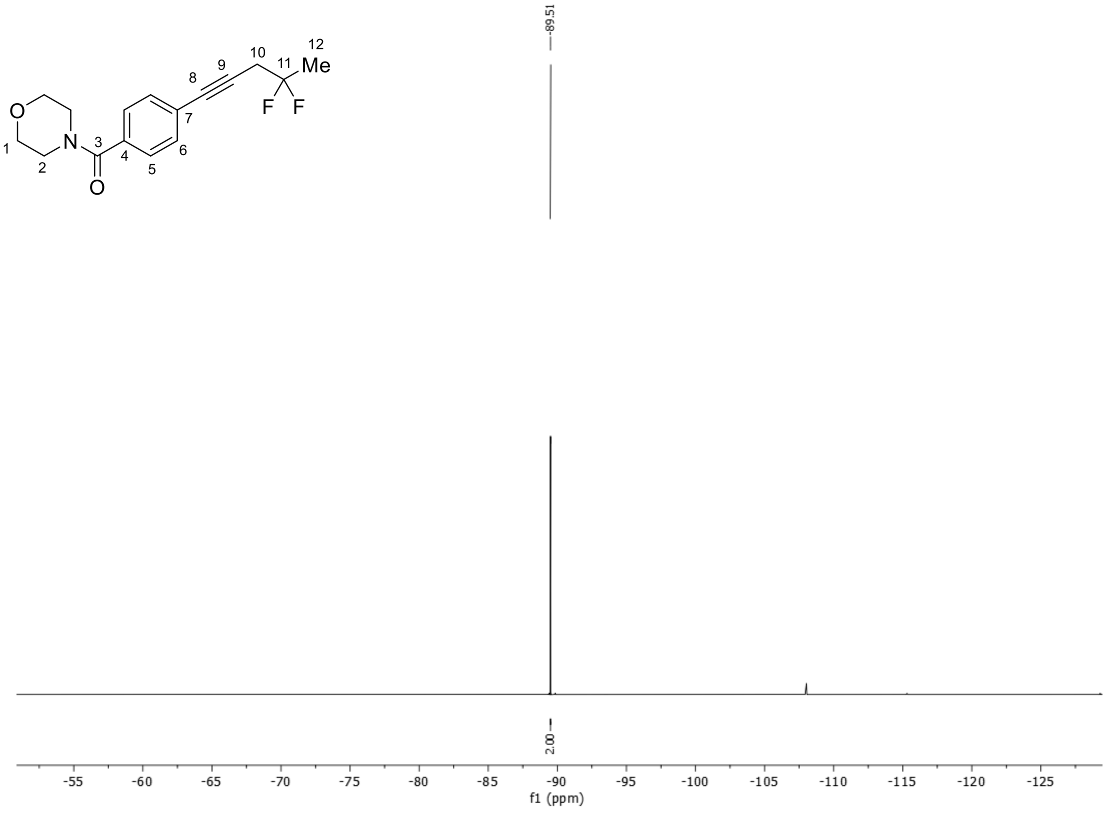

Supplementary Figure 134. $^{19}F\{^1H\}$ NMR of **13** (564 MHz, 299 K, $CDCl_3$).

1-(4,4-Difluorohex-1-yn-1-yl)-4-nitrobenzene (14)

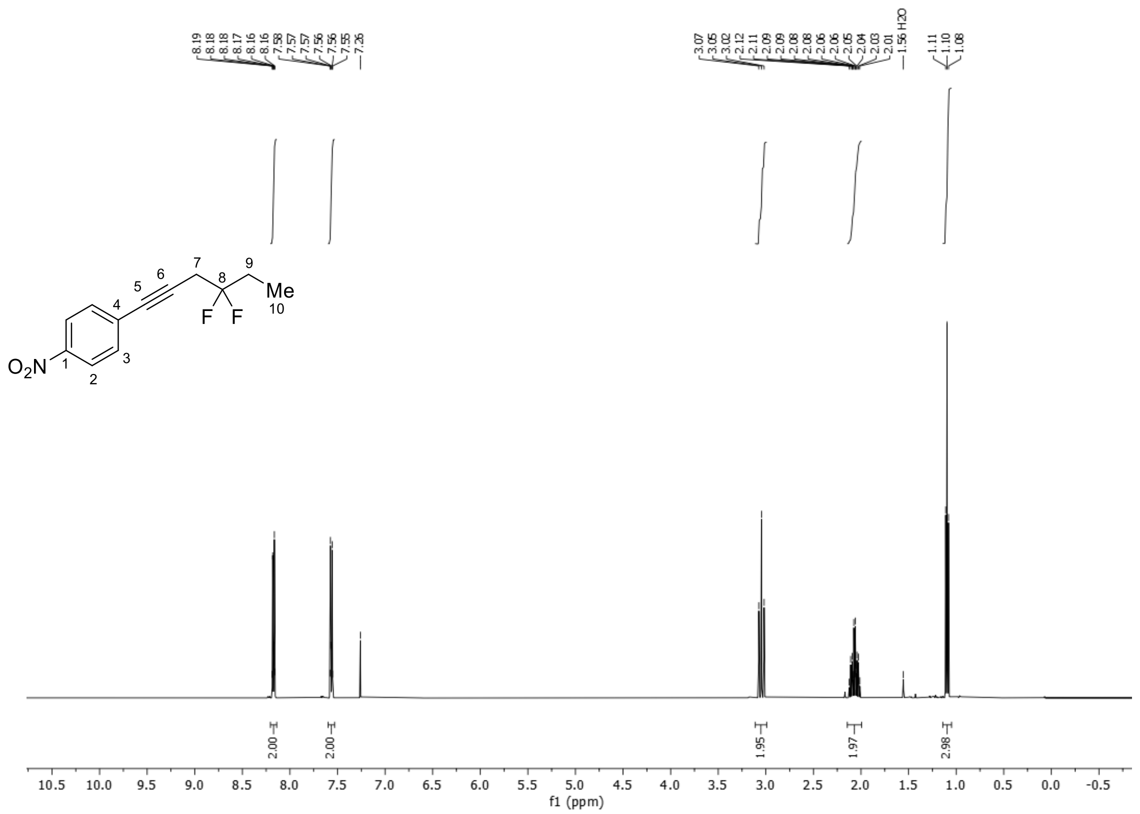

Supplementary Figure 135. ^{1}H NMR of **14** (500 MHz, 299 K, $CDCl_3$).

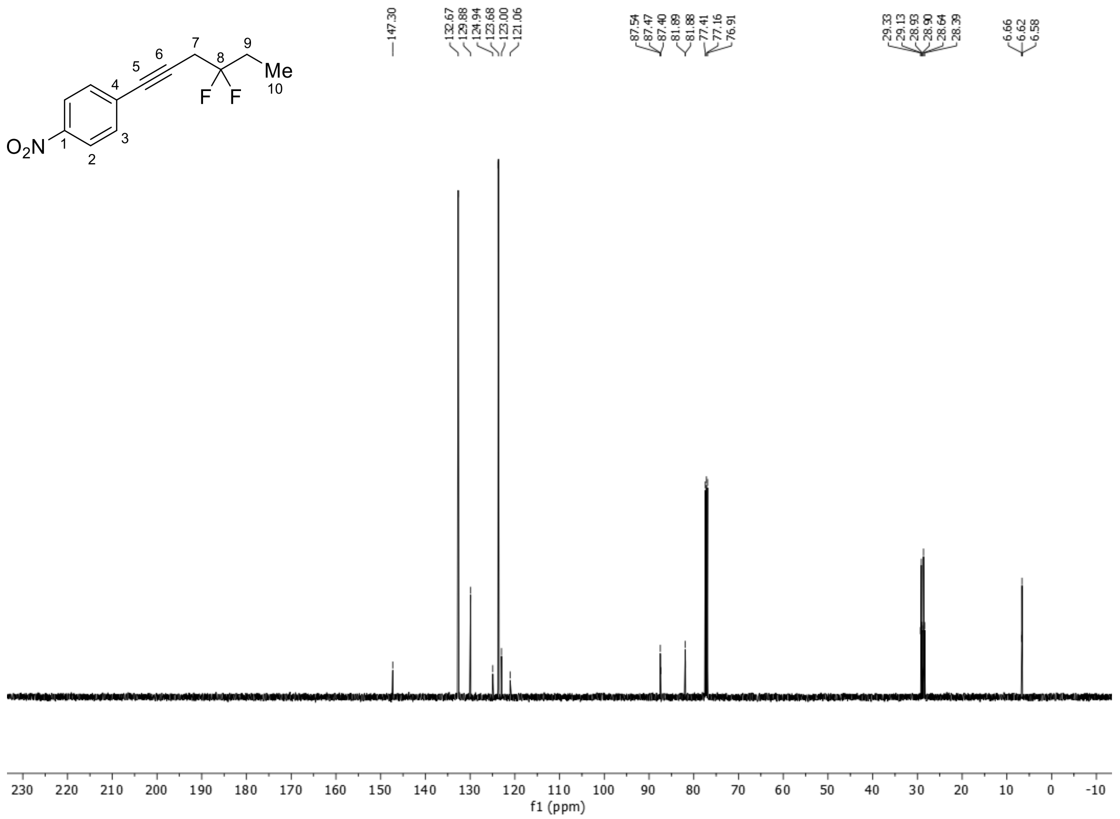

Supplementary Figure 136. $^{13}C\{^{1}H\}$ NMR of **14** (126 MHz, 299 K, $CDCl_3$).

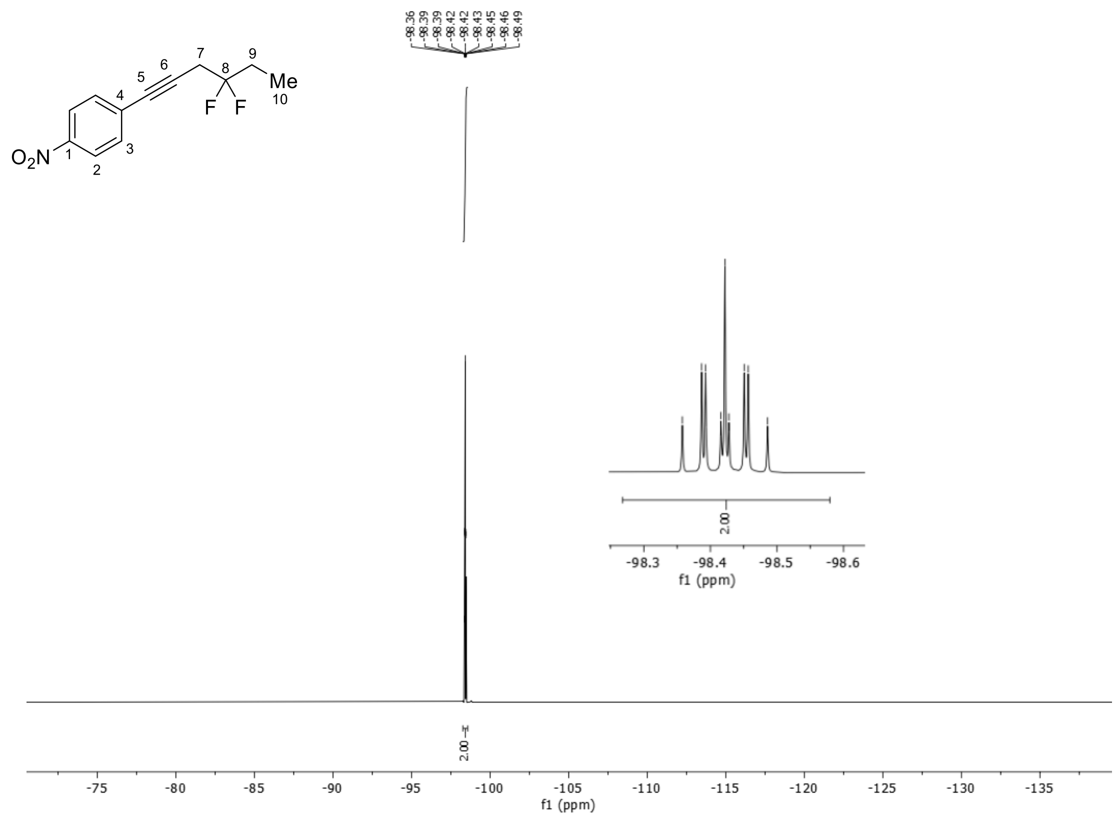

Supplementary Figure 137. ^{19}F NMR of **14** (470 MHz, 299 K, $CDCl_3$).

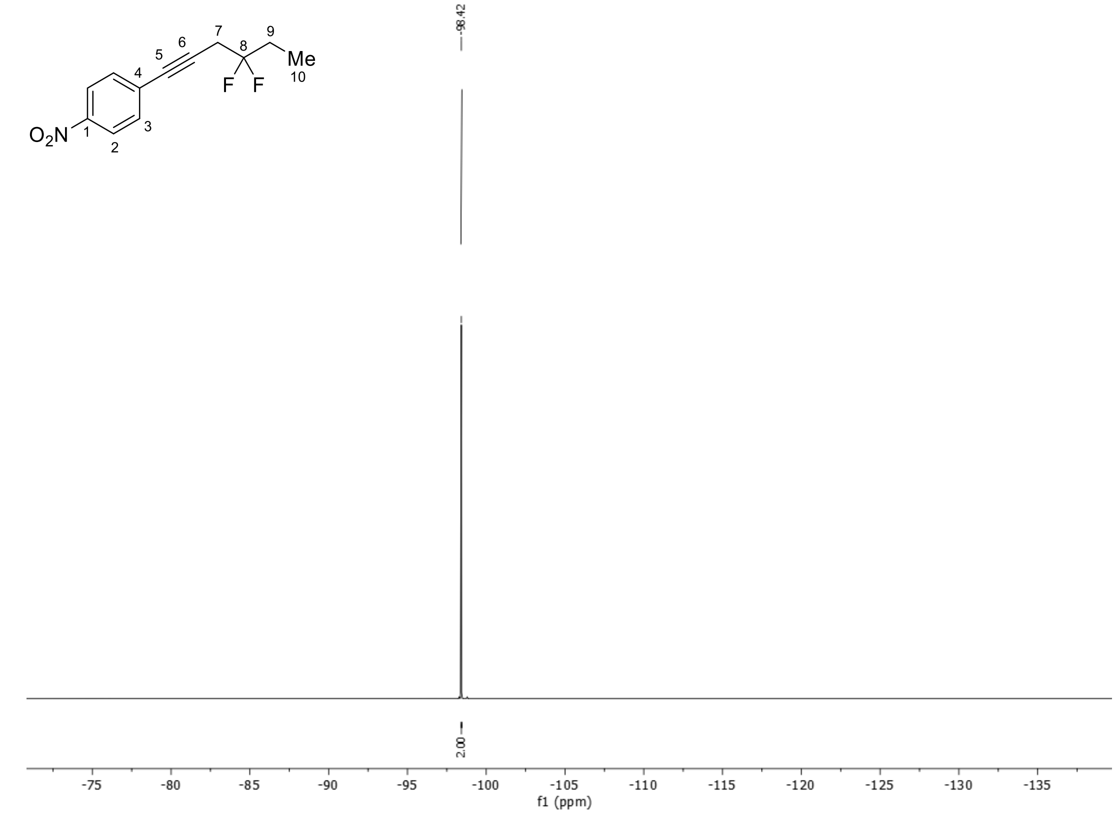

Supplementary Figure 138. $^{19}F\{^1H\}$ NMR of **14** (470 MHz, 299 K, $CDCl_3$).

1-(5-Bromo-4,4-difluoropent-1-yn-1-yl)-4-nitrobenzene (15)

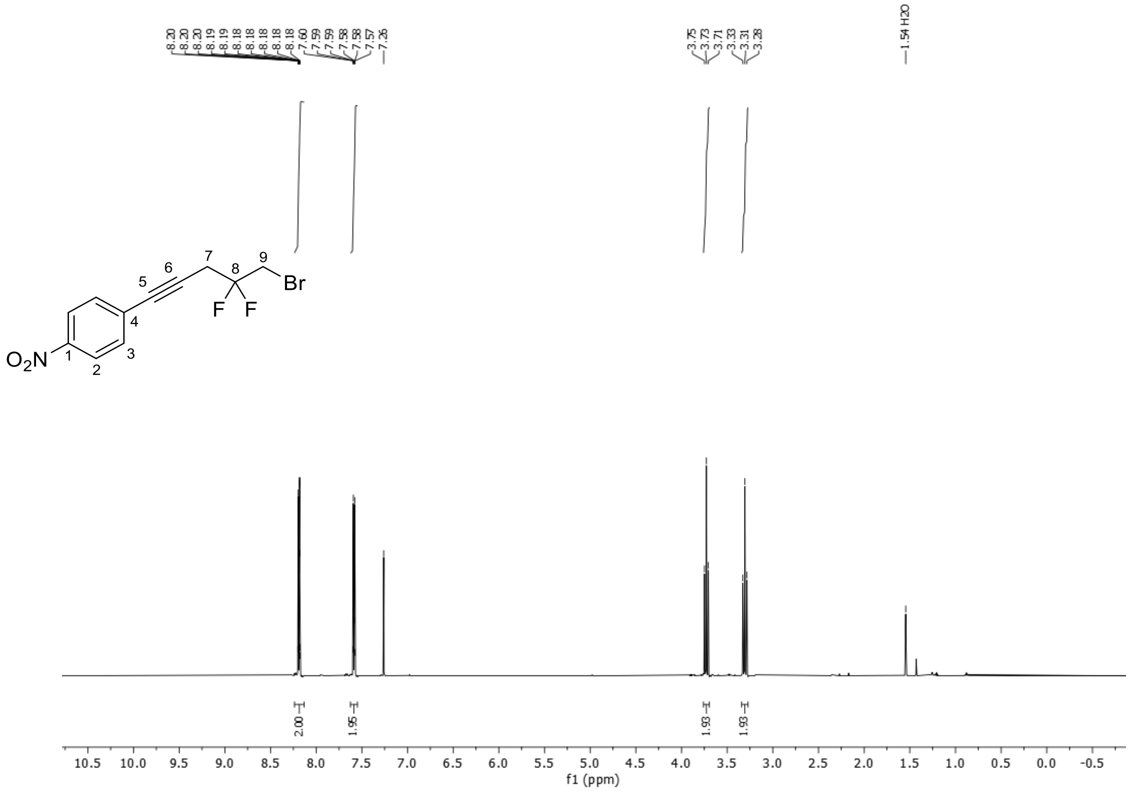

Supplementary Figure 139. ^{1}H NMR of **15** (600 MHz, 299 K, $CDCl_3$).

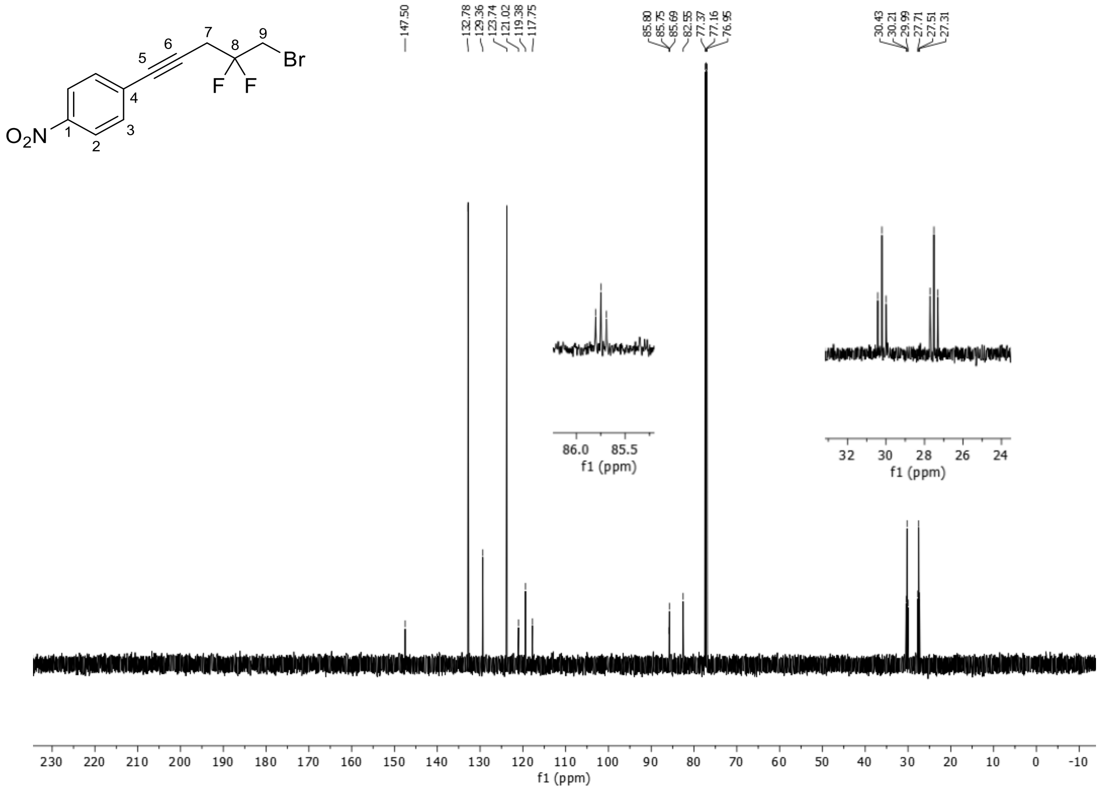

Supplementary Figure 140. $^{13}C\{^{1}H\}$ NMR of **15** (151 MHz, 299 K, $CDCl_3$).

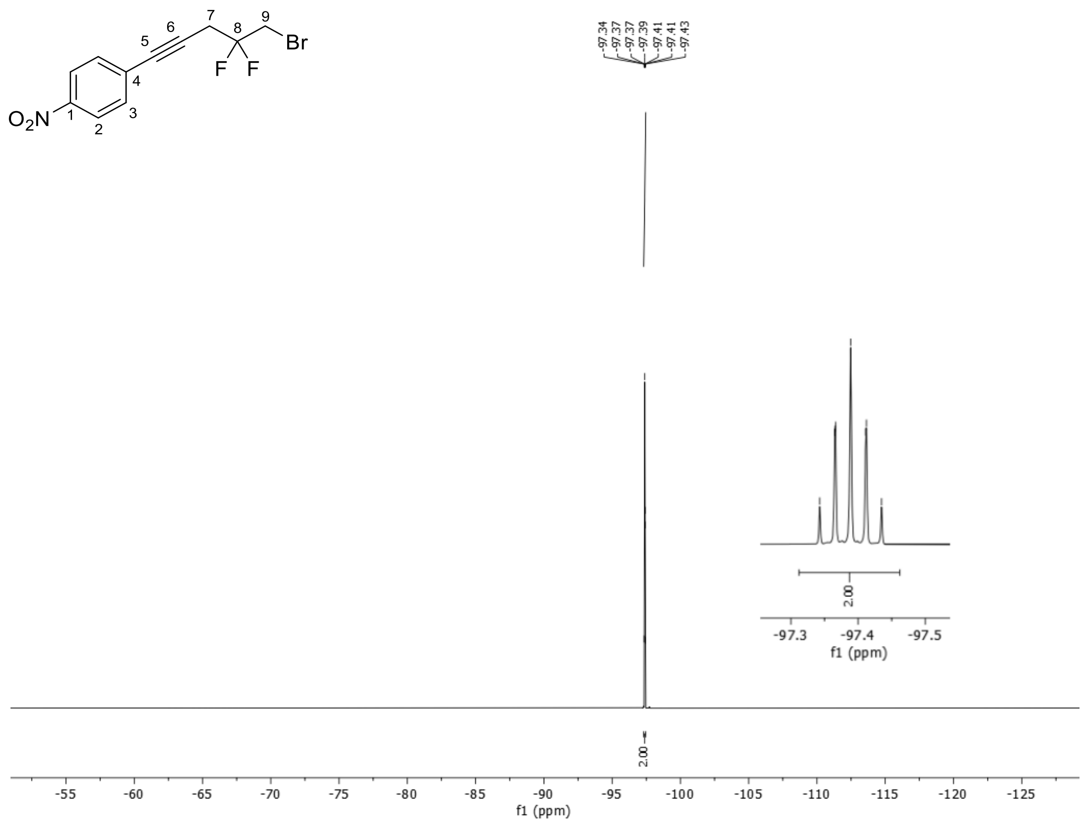

Supplementary Figure 141. ^{19}F NMR of **15** (564 MHz, 299 K, $CDCl_3$).

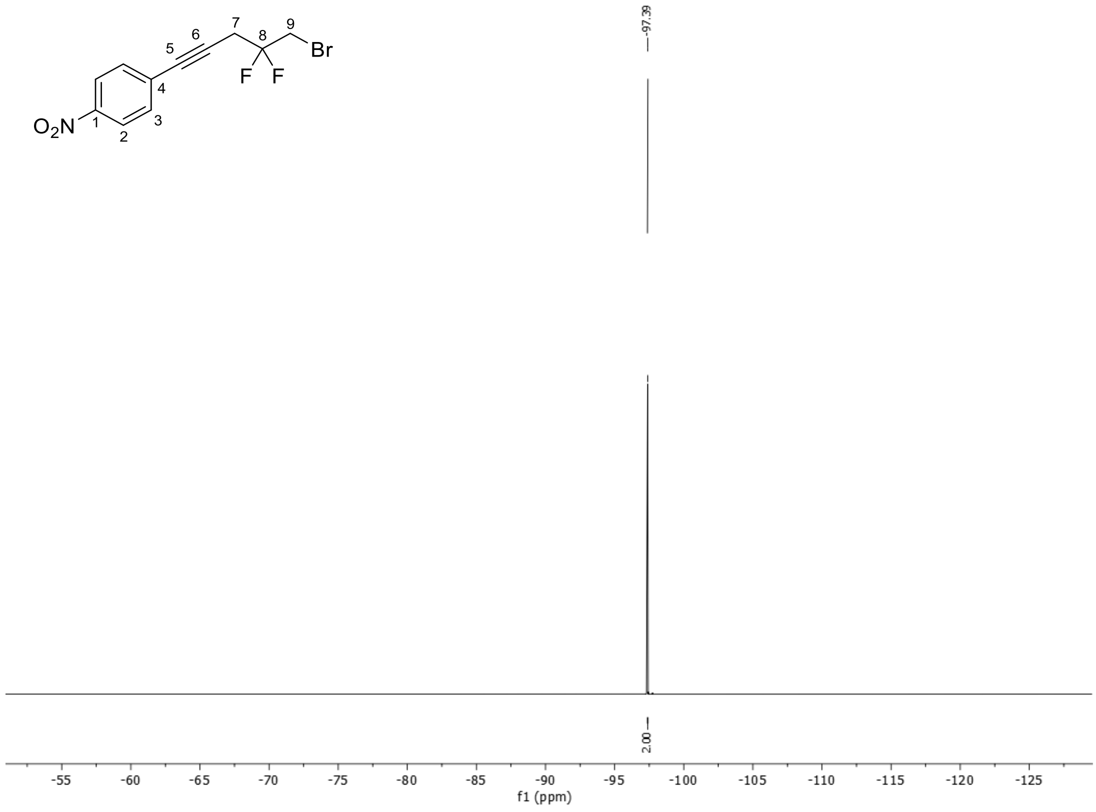

Supplementary Figure 142. $^{19}F\{^{1}H\}$ NMR of **15** (470 MHz, 299 K, $CDCl_3$).

1-(5-Chloro-4,4-difluoropent-1-yn-1-yl)-4-nitrobenzene (16)

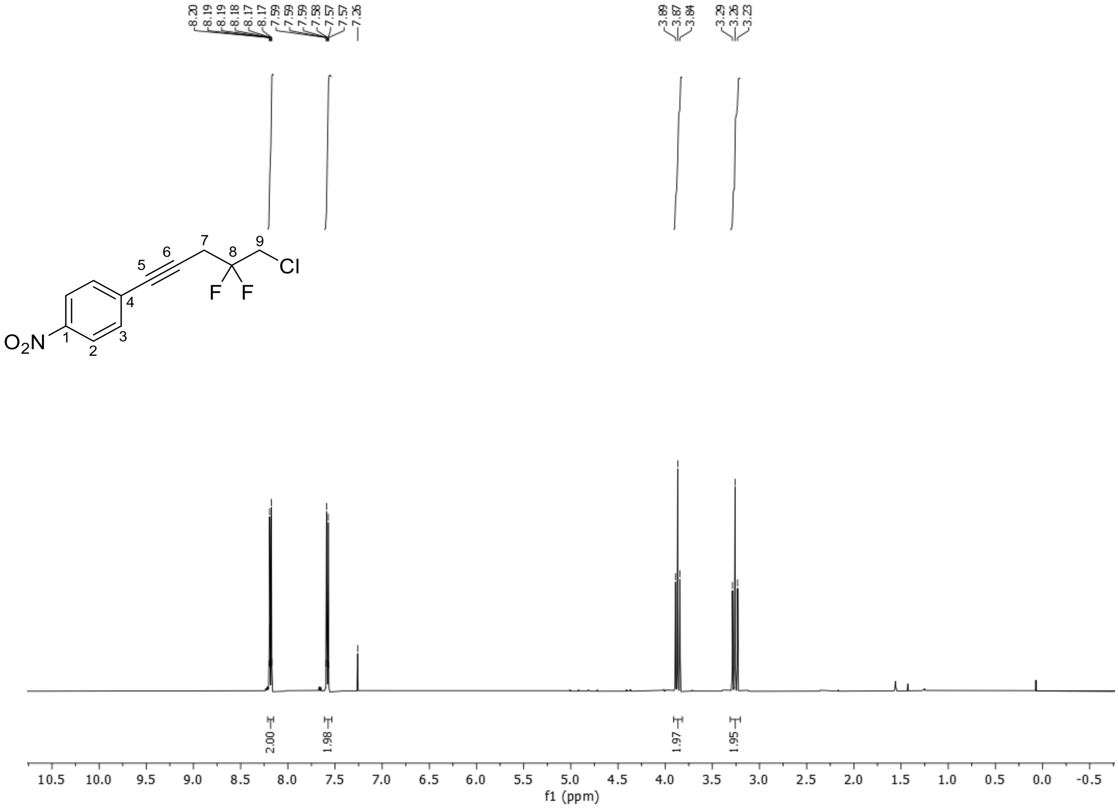

Supplementary Figure 143. ^{1}H NMR of **16** (500 MHz, 299 K, $CDCl_3$).

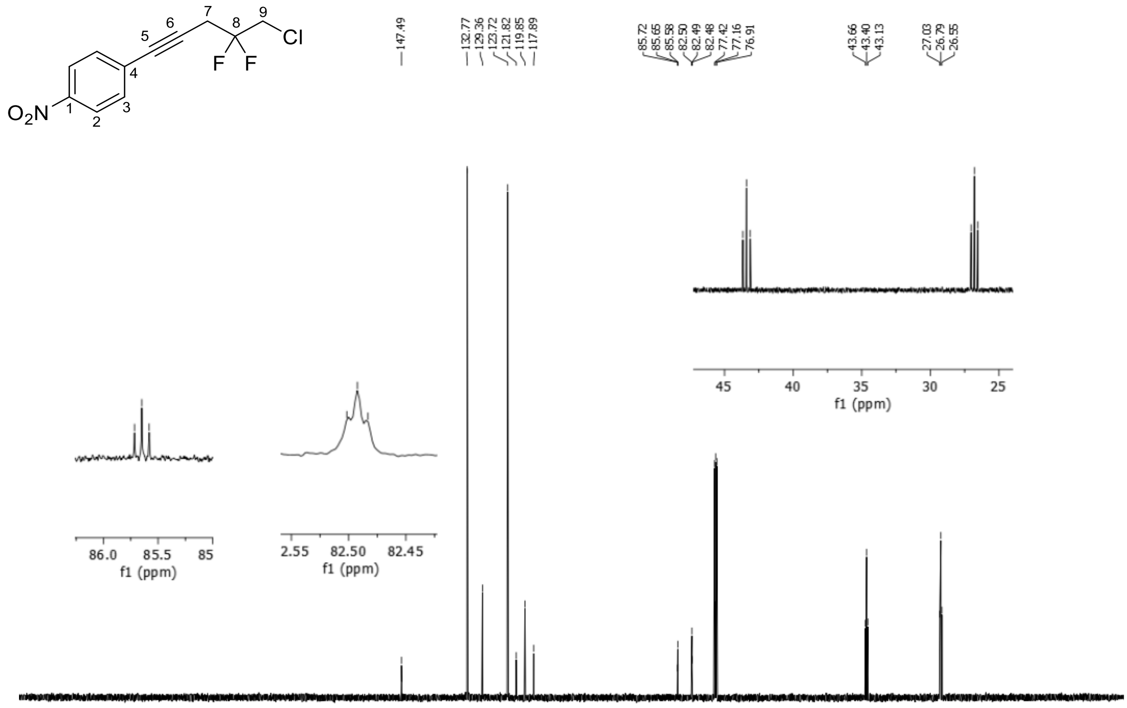

Supplementary Figure 144. $^{13}C\{^{1}H\}$ NMR of **16** (126 MHz, 299 K, $CDCl_3$).

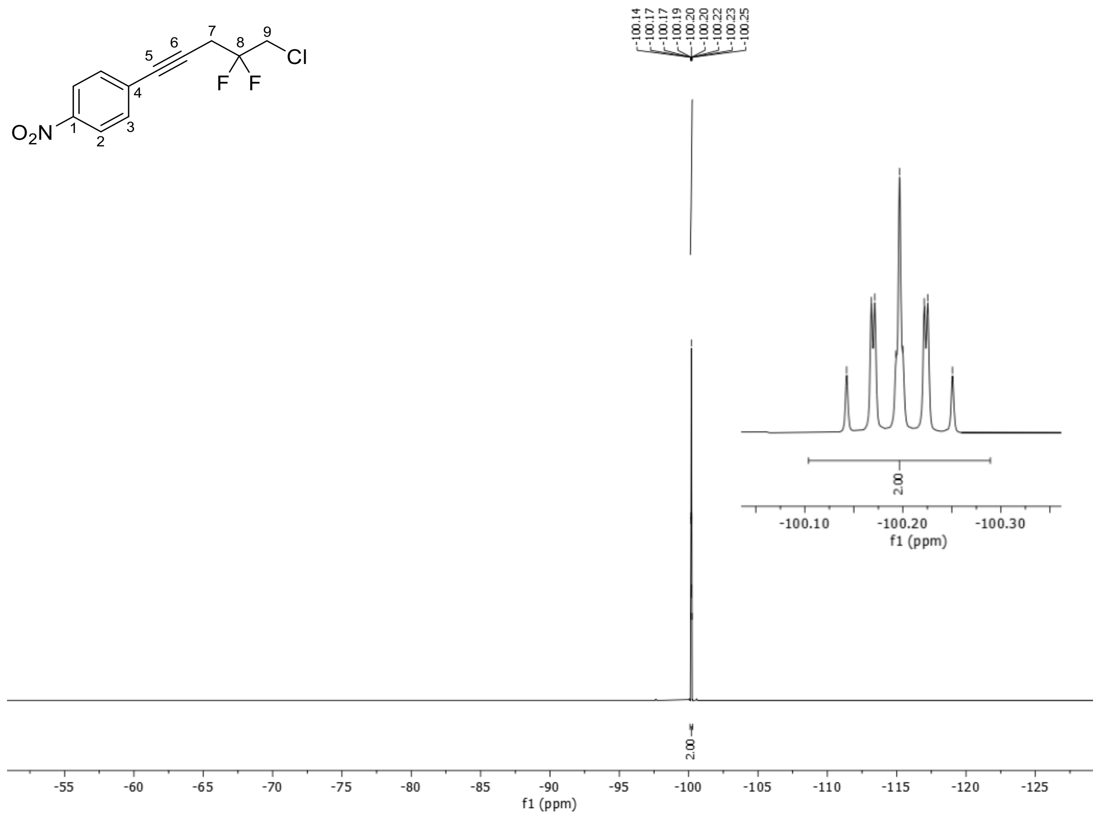

Supplementary Figure 145. ^{19}F NMR of **16** (470 MHz, 299 K, $CDCl_3$).

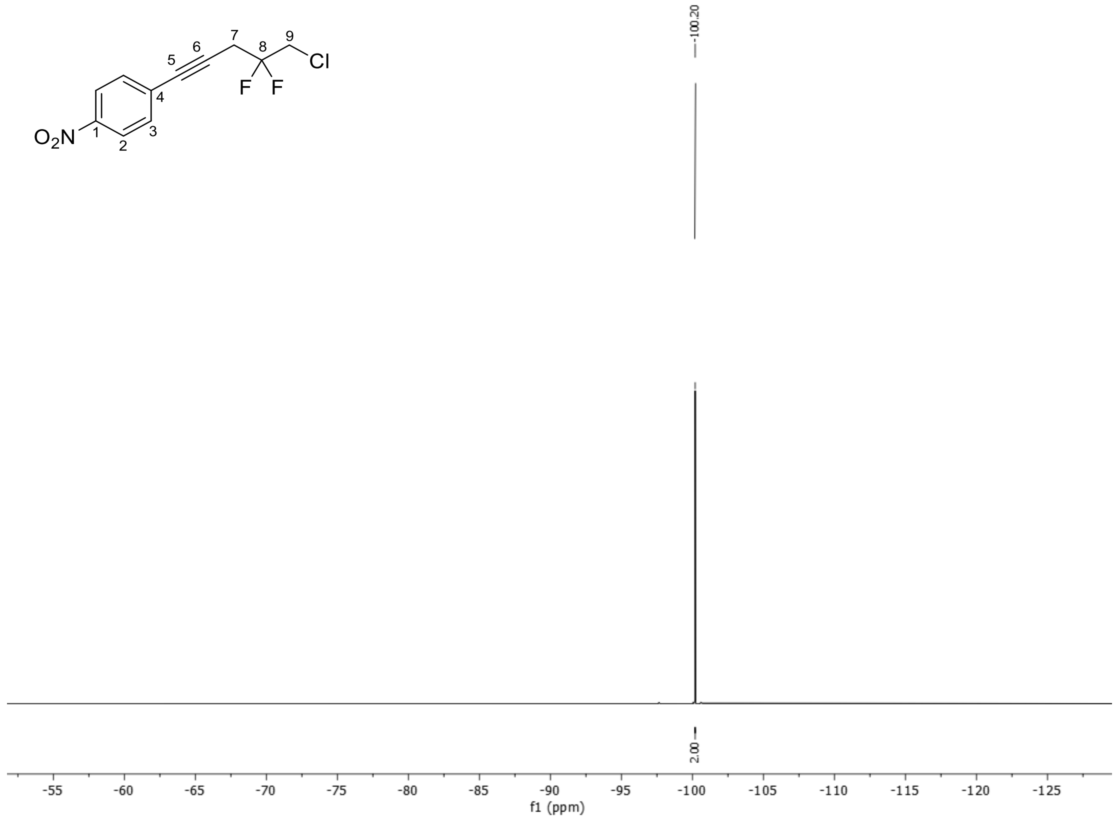

Supplementary Figure 146. $^{19}F\{^1H\}$ NMR of **16** (470 MHz, 299 K, $CDCl_3$).

(1*R*,2*S*,5*R*)-2-Isopropyl-5-methylcyclohexyl 4-(4,4-difluoropent-1-yn-1-yl)benzoate (17)

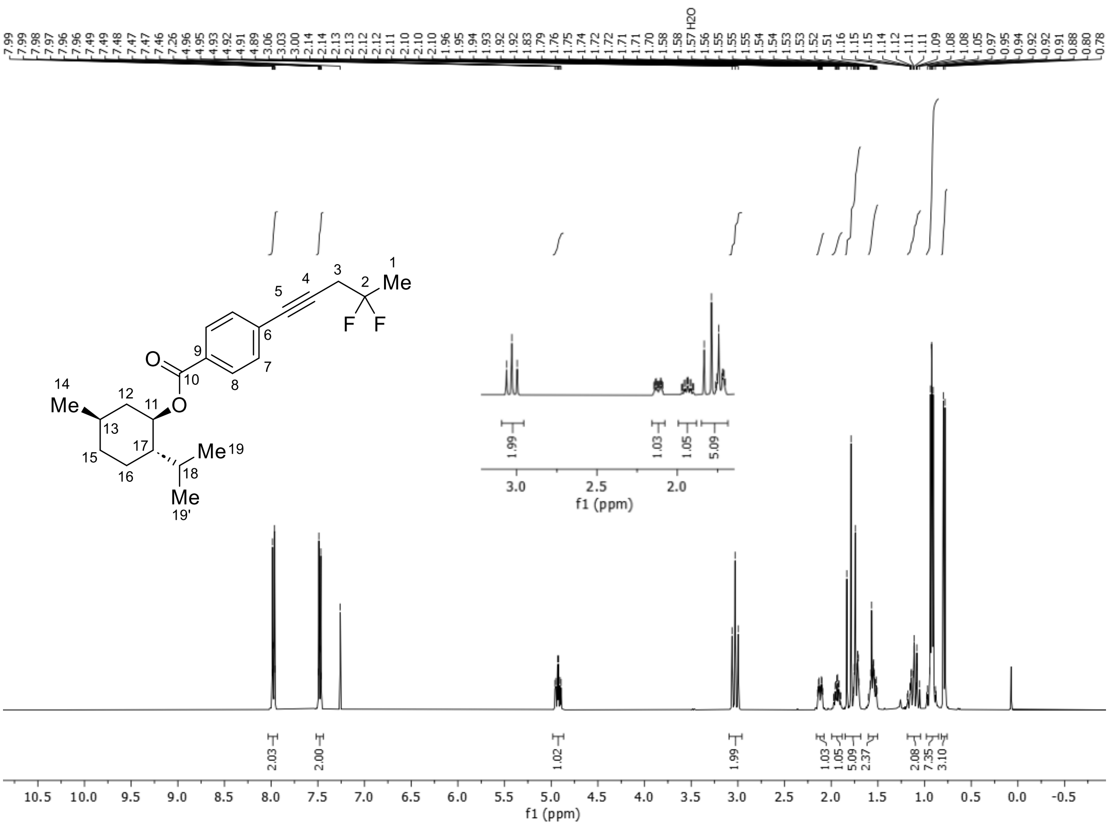

Supplementary Figure 147. ^{1}H NMR of **17** (400 MHz, 299 K, $CDCl_3$).

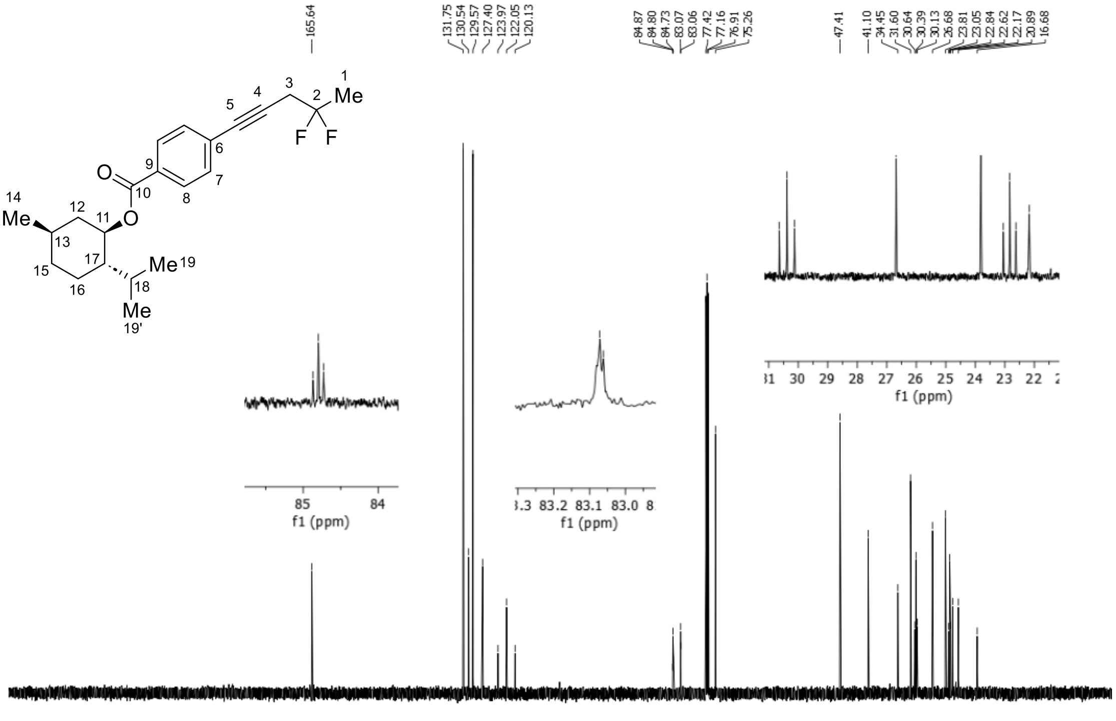

Supplementary Figure 148. $^{13}C\{^1H\}$ NMR of **17** (126 MHz, 299 K, $CDCl_3$).

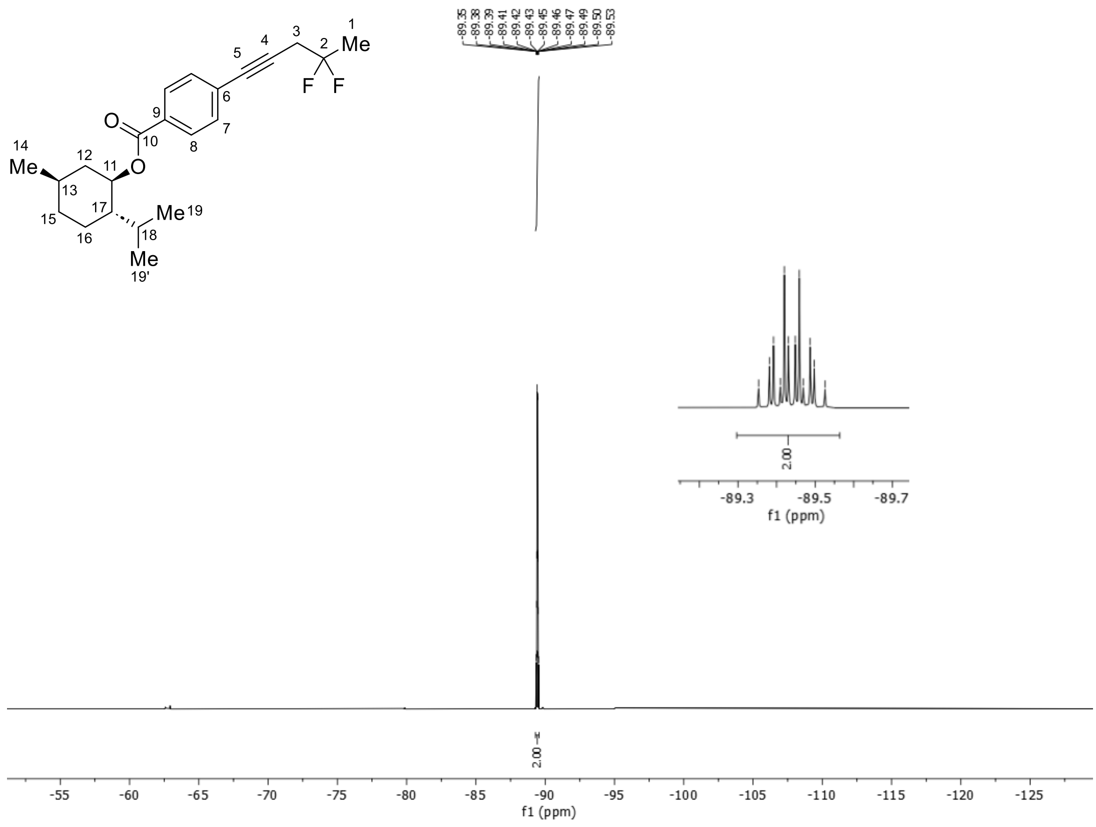

Supplementary Figure 149. ^{19}F NMR of **17** (470 MHz, 299 K, $CDCl_3$).

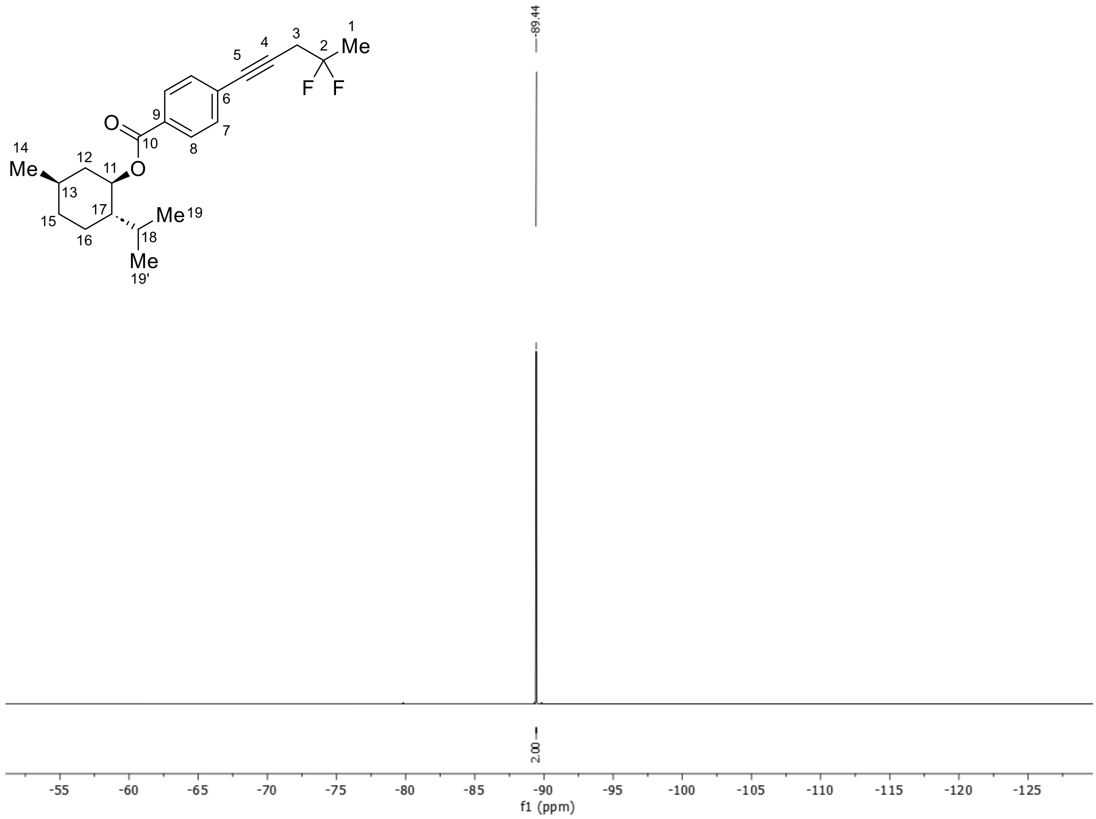

Supplementary Figure 150. ^{19}F{^{1}H} NMR of **17** (470 MHz, 299 K, $CDCl_3$).

(3*S*,5*S*,8*R*,9*S*,10*S*,13*R*,14*S*,17*R*)-10,13-Dimethyl-17-((*R*)-6-methylheptan-2-yl)hexadecahydro-1H-cyclopenta[a]phenanthren-3-yl 4-(4,4-difluoropent-1-yn-1-yl)benzoate (18)

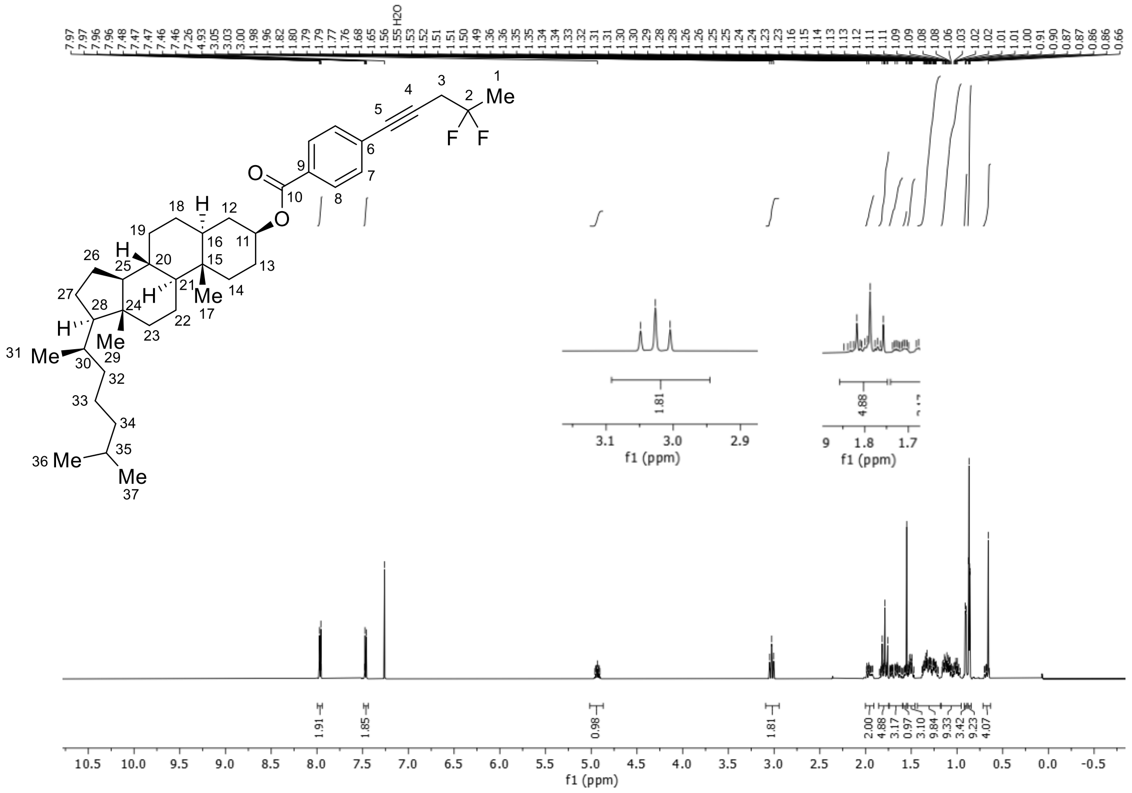

Supplementary Figure 151. ^{1}H NMR of **18** (599 MHz, 299 K, $CDCl_3$).

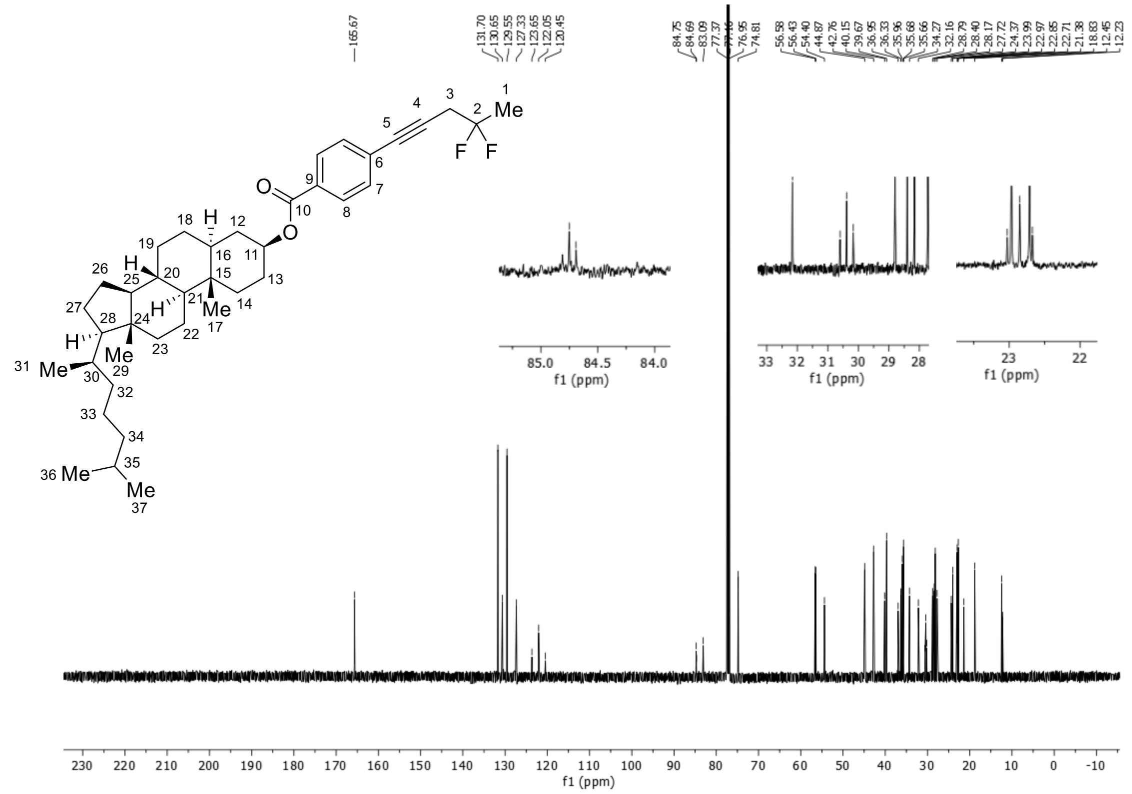

Supplementary Figure 152. $^{13}C\{^{1}H\}$ NMR of **18** (151 MHz, 299 K, $CDCl_3$).

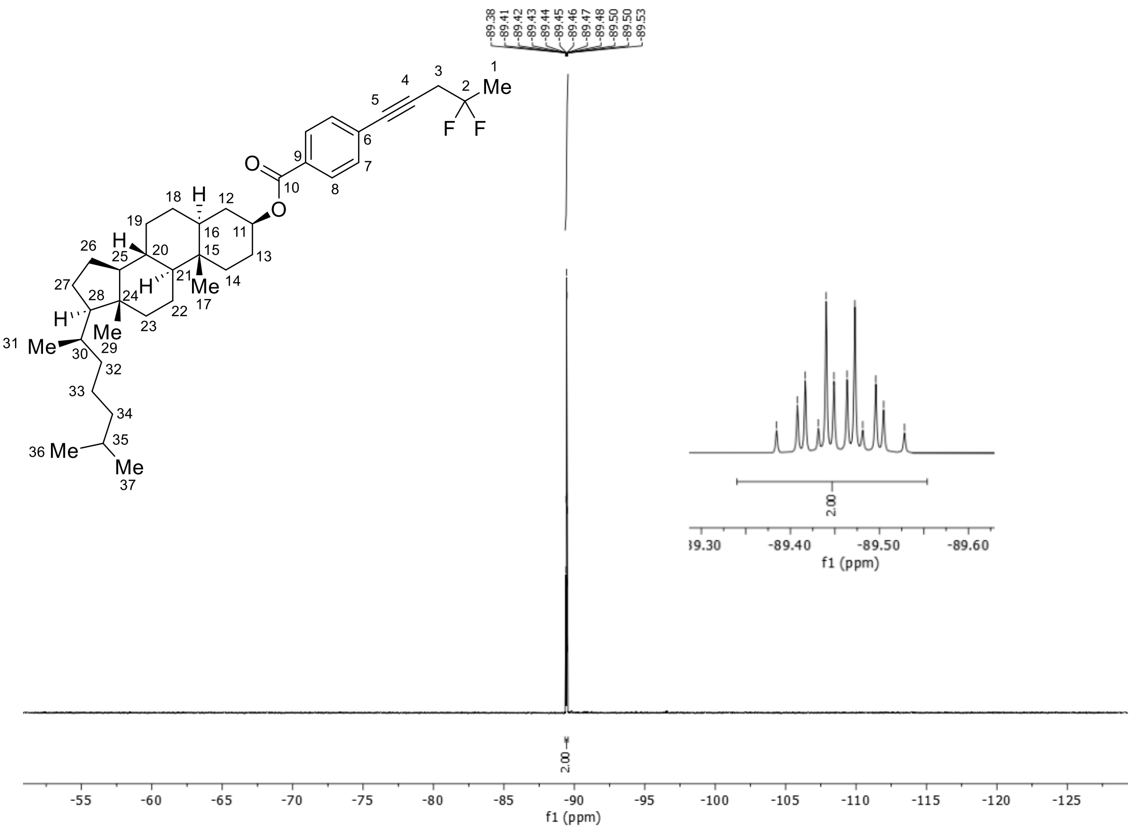

Supplementary Figure 153. ^{19}F NMR of **18** (564 MHz, 299 K, $CDCl_3$).

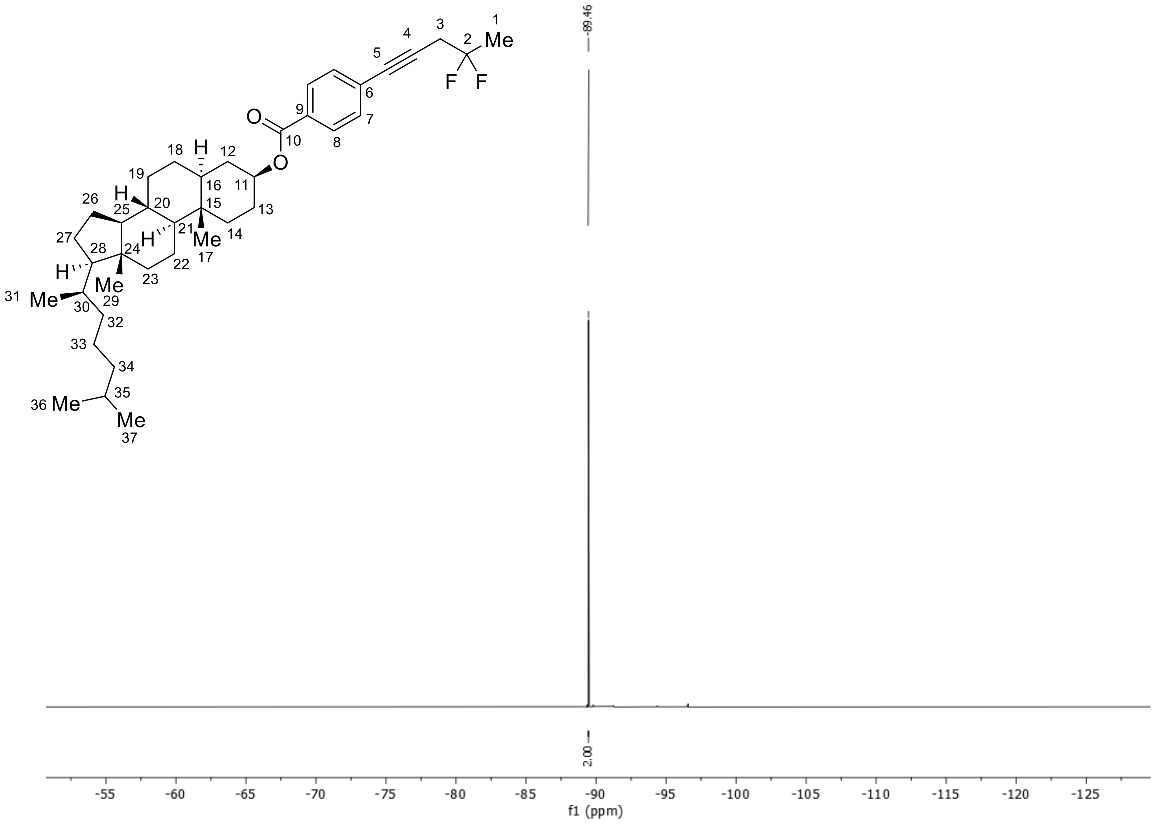

Supplementary Figure 154. ^{19}F{^{1}H} NMR of **18** (564 MHz, 299 K, $CDCl_3$).

(8*R*,9*S*,13*S*)-13-Methyl-17-oxo-7,8,9,11,12,13,14,15,16,17-decahydro-6H-cyclopenta[a]phenanthren-3-yl 4-(4,4-difluoropent-1-yn-1-yl)benzoate (19)

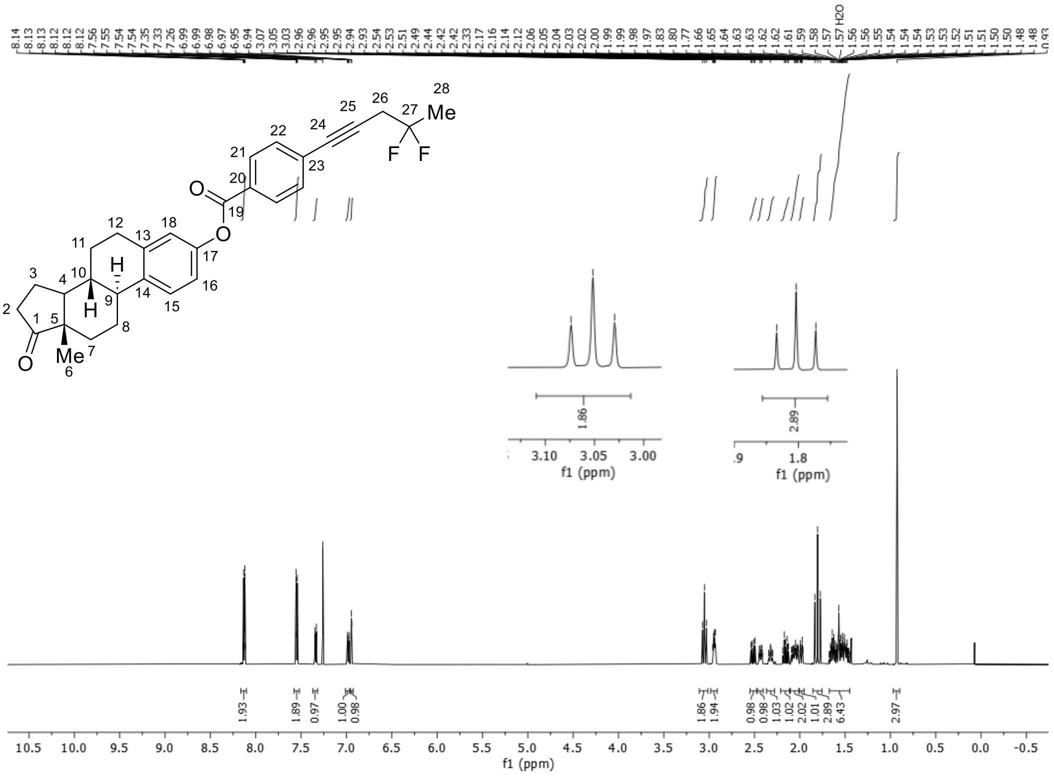

Supplementary Figure 155. ^{1}H NMR of **19** (599 MHz, 299 K, $CDCl_3$).

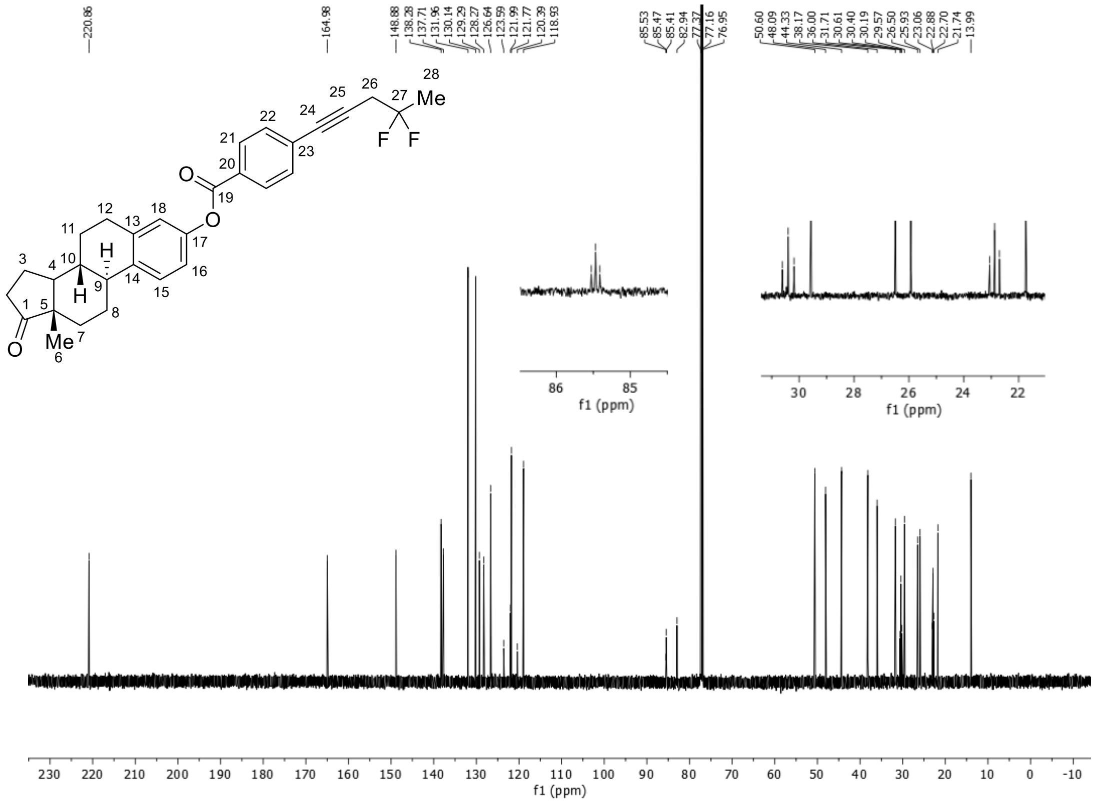

Supplementary Figure 156. $^{13}C\{^{1}H\}$ NMR of **19** (151 MHz, 299 K, $CDCl_3$).

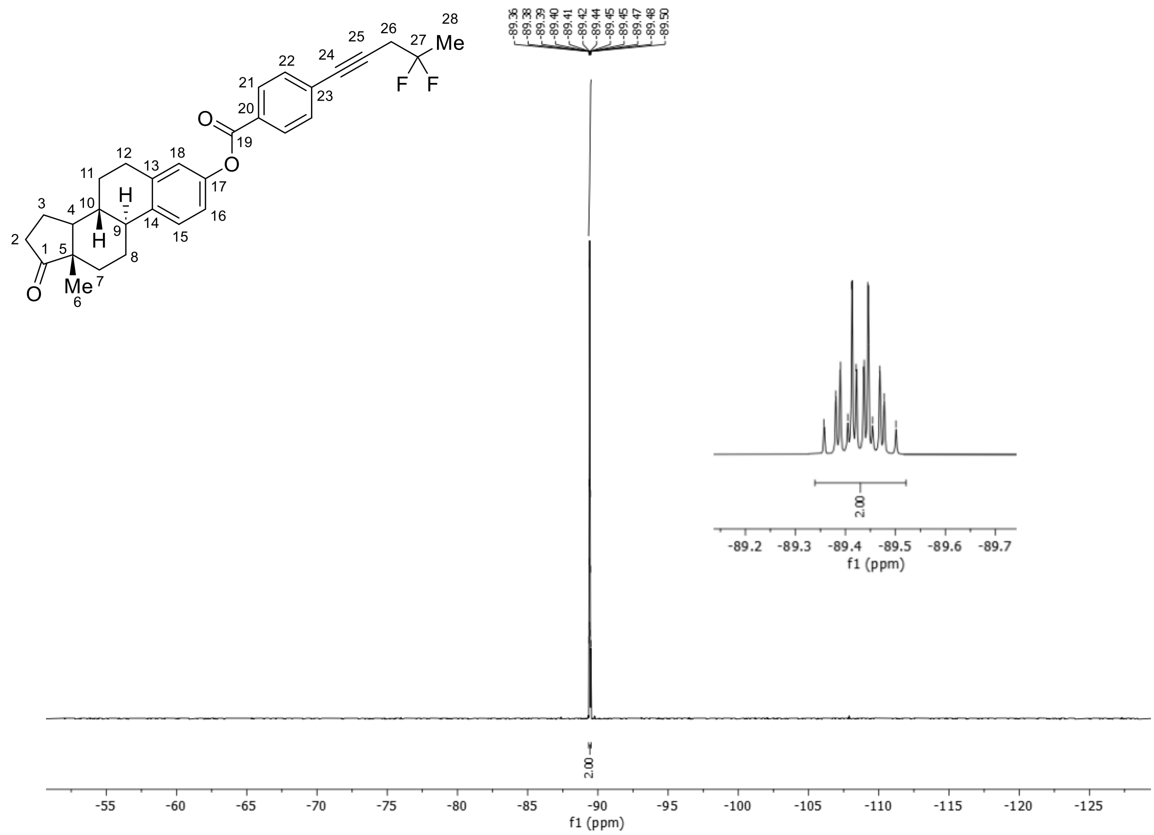

Supplementary Figure 157. ^{19}F NMR of **19** (564 MHz, 299 K, $CDCl_3$).

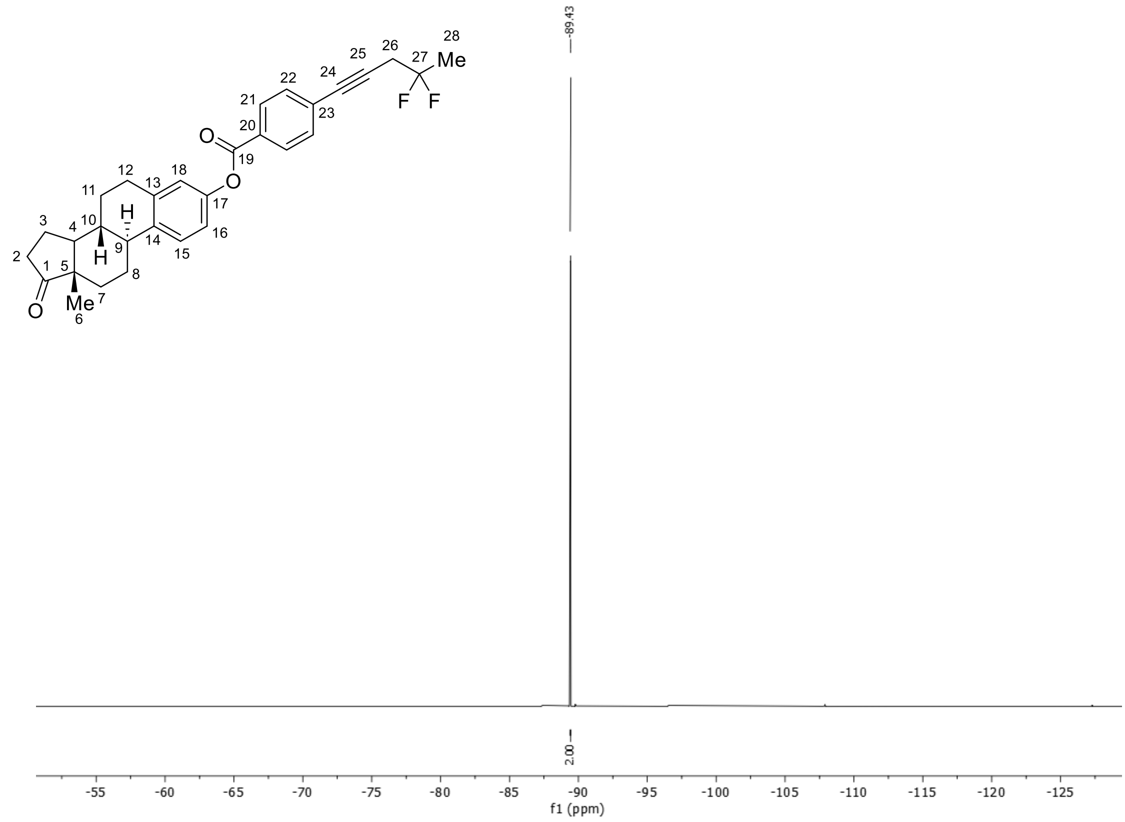

Supplementary Figure 158. ^{19}F{^{1}H} NMR of **19** (564 MHz, 299 K, $CDCl_3$).

2,2-Difluoroheptadec-4-yne (20)

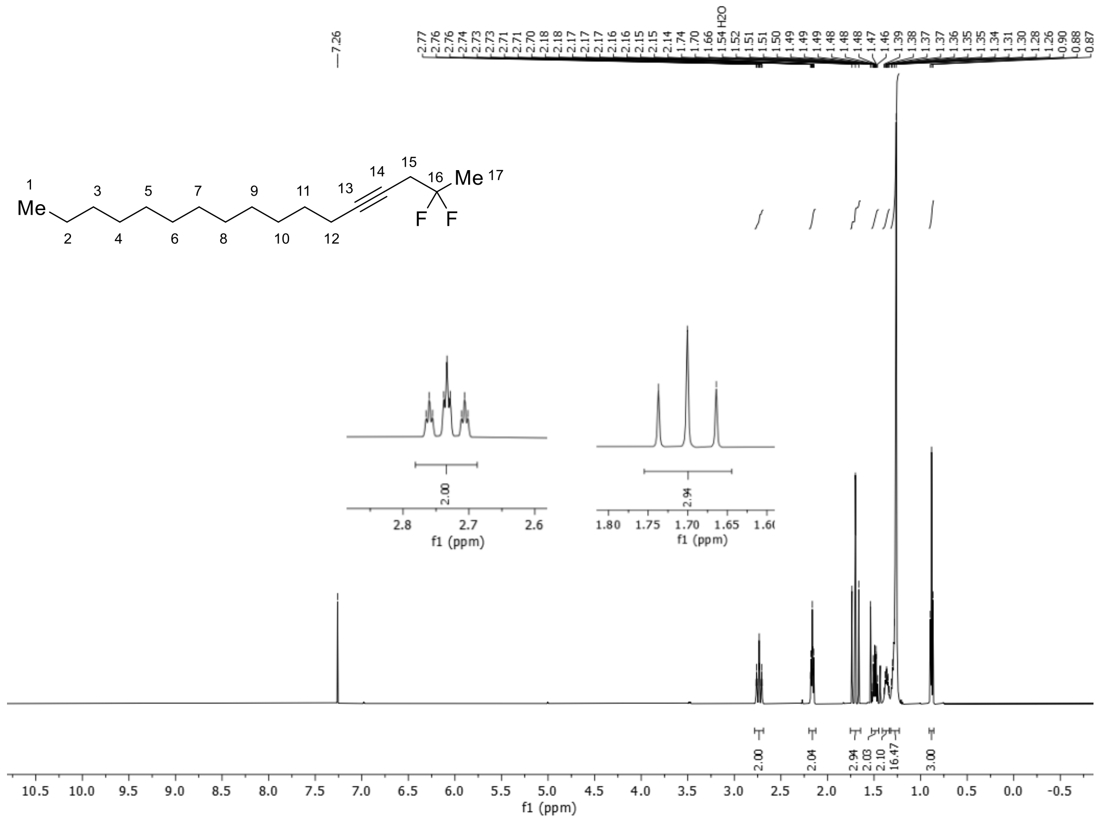

Supplementary Figure 159. ^{1}H NMR of **20** (500 MHz, 299 K, $CDCl_3$).

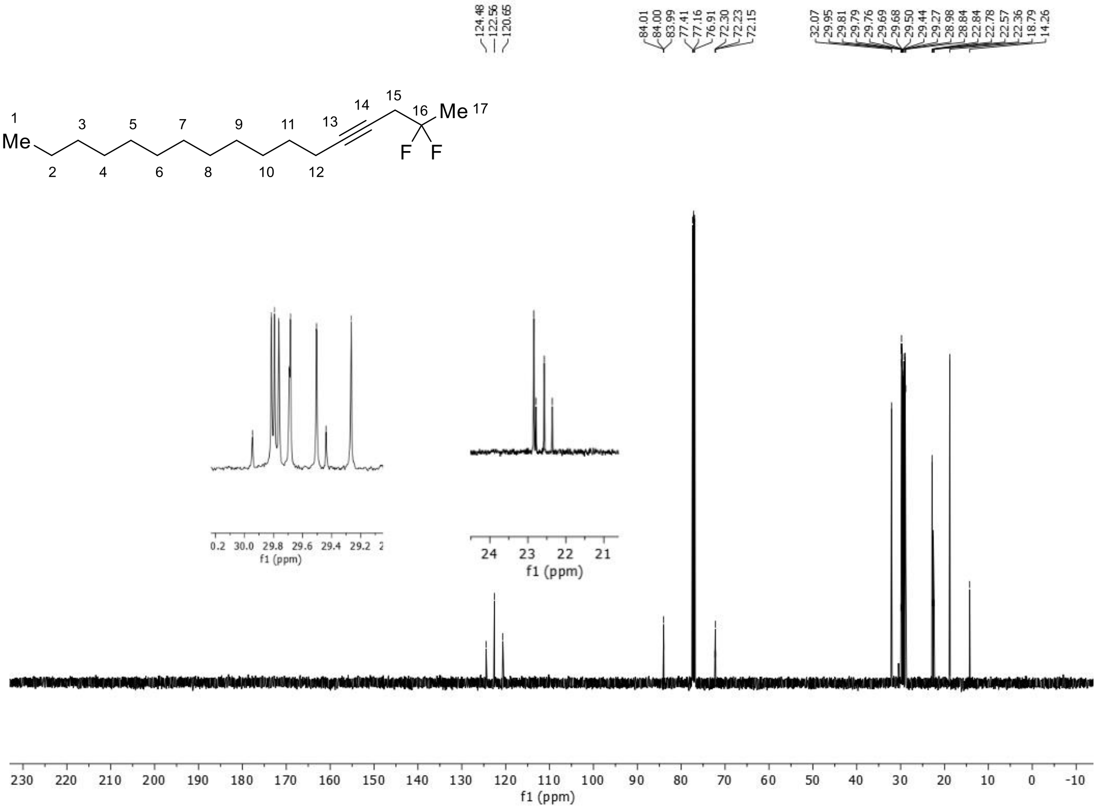

Supplementary Figure 160. $^{13}C\{^{1}H\}$ NMR of **20** (126 MHz, 299 K, $CDCl_3$).

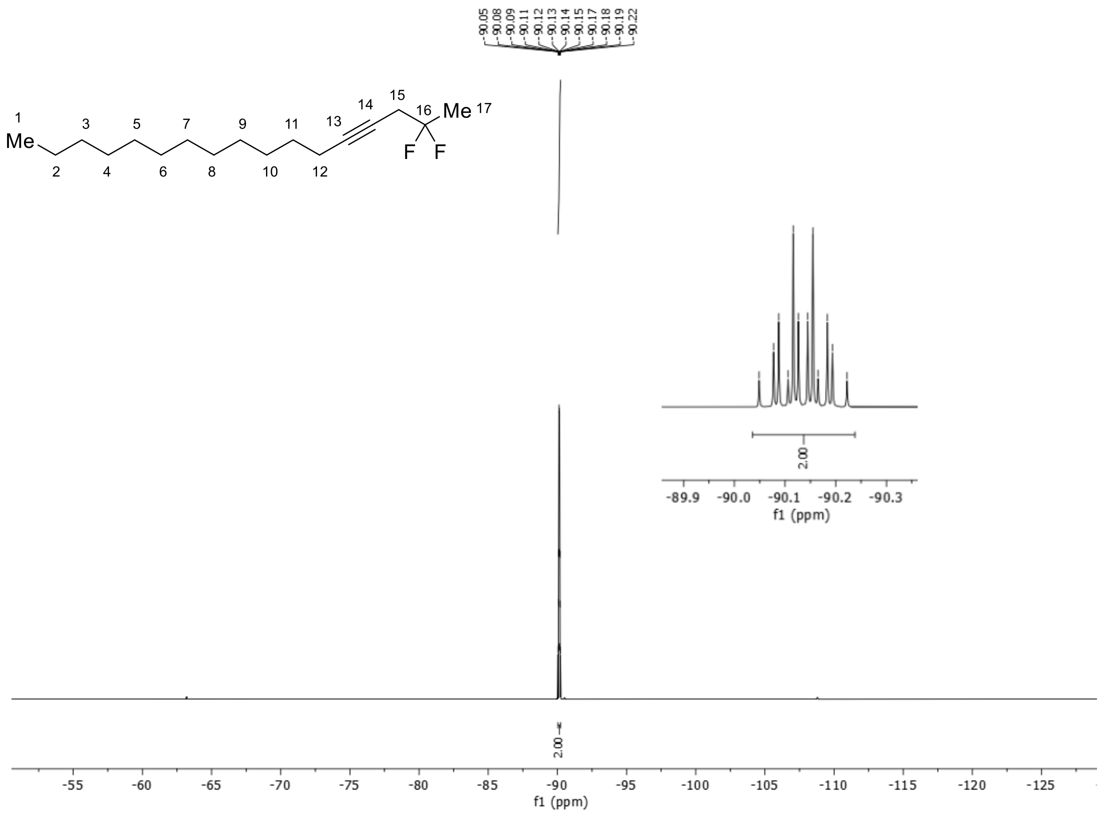

Supplementary Figure 161. ^{19}F NMR of **20** (470 MHz, 299 K, $CDCl_3$).

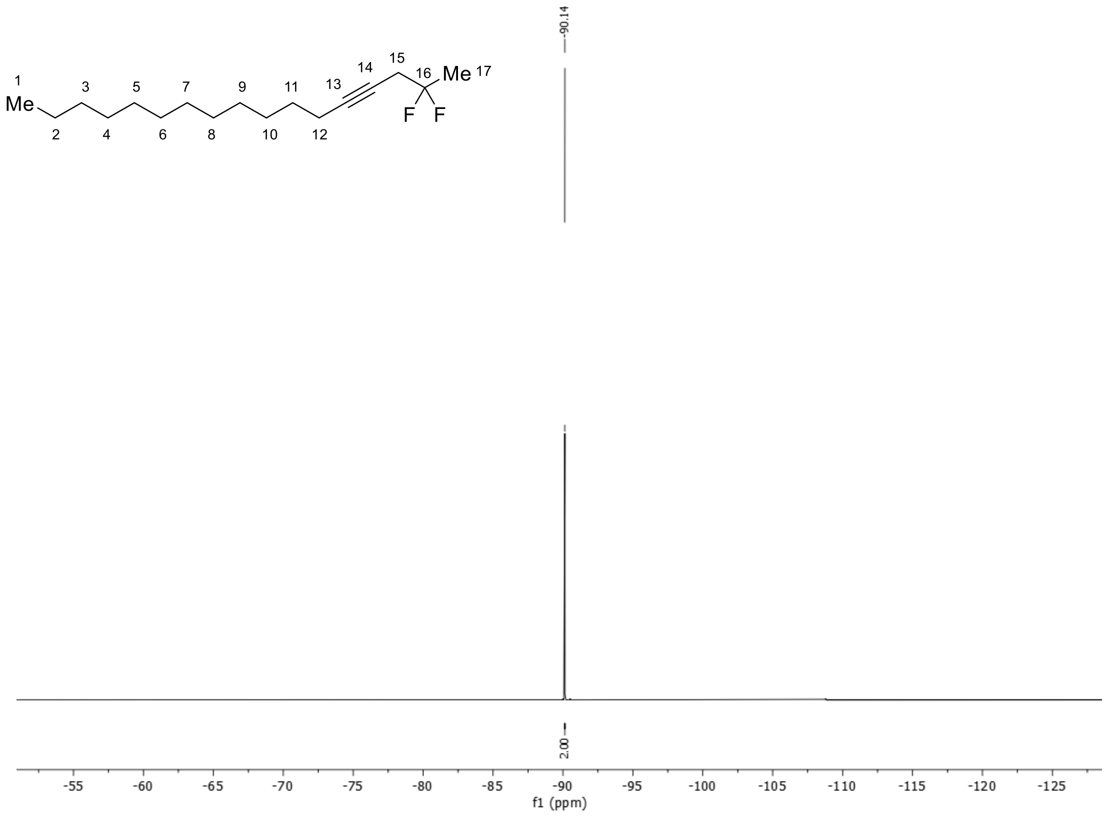

Supplementary Figure 162. $^{19}F\{^1H\}$ NMR of **20** (470 MHz, 299 K, $CDCl_3$).

(5,5-Difluorohex-2-yn-1-yl)cyclohexane (21)

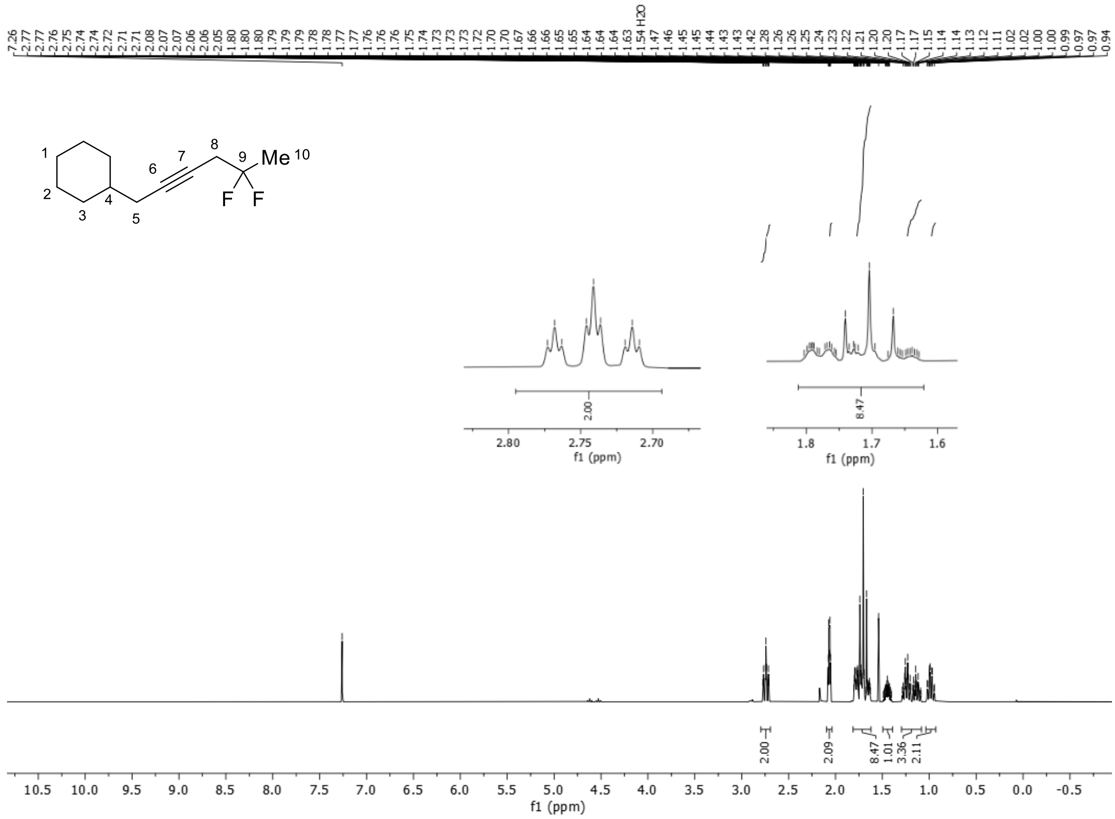

Supplementary Figure 163. ^{1}H NMR of **21** (500 MHz, 299 K, $CDCl_3$).

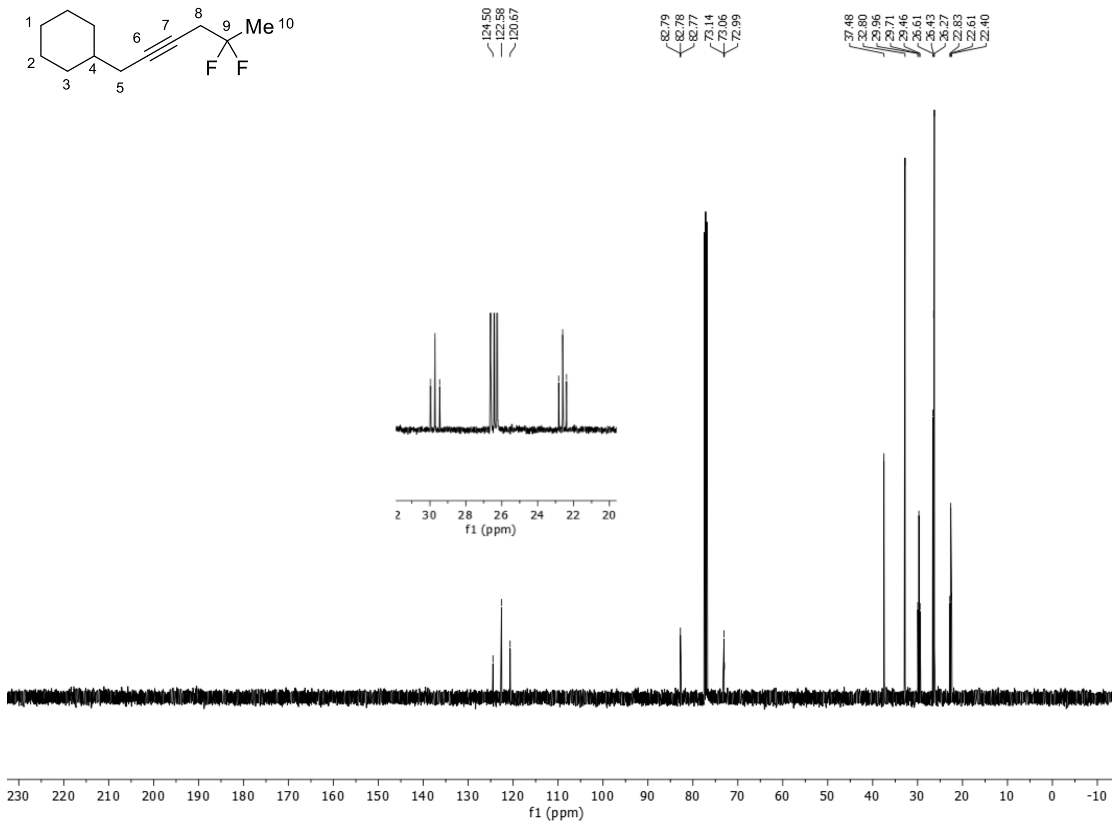

Supplementary Figure 164. $^{13}C\{^{1}H\}$ NMR of **21** (126 MHz, 299 K, $CDCl_3$).

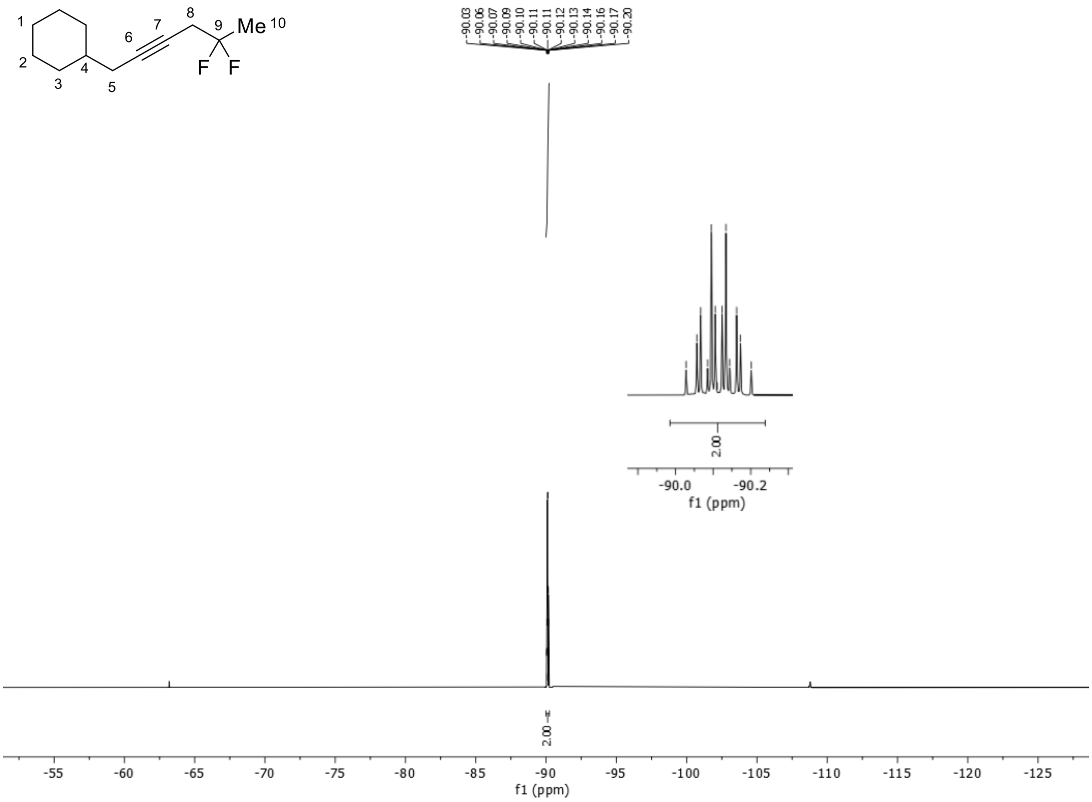

Supplementary Figure 165. ^{19}F NMR of **21** (470 MHz, 299 K, $CDCl_3$).

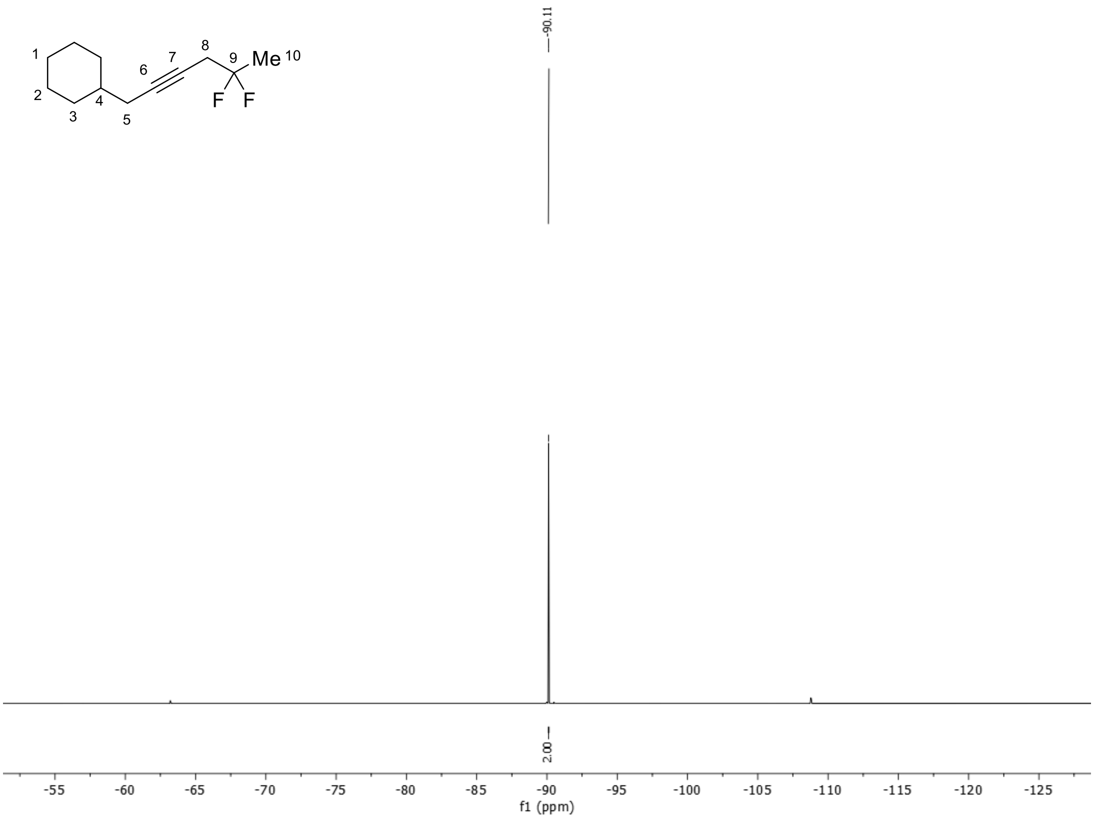

Supplementary Figure 166. $^{19}F\{^1H\}$ NMR of **21** (470 MHz, 299 K, $CDCl_3$).

2-(7,7-Difluorooct-4-yn-1-yl)isoindoline-1,3-dione (22)

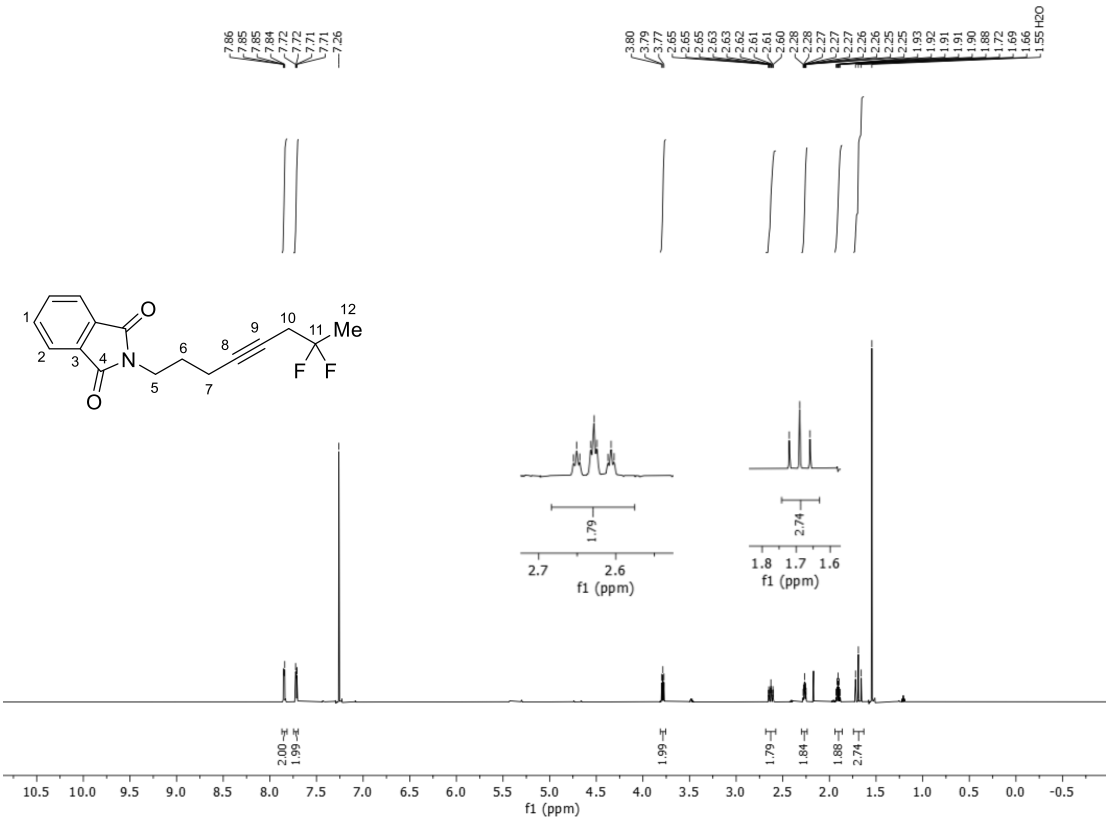

Supplementary Figure 167. ^{1}H NMR of **22** (599 MHz, 299 K, $CDCl_3$).

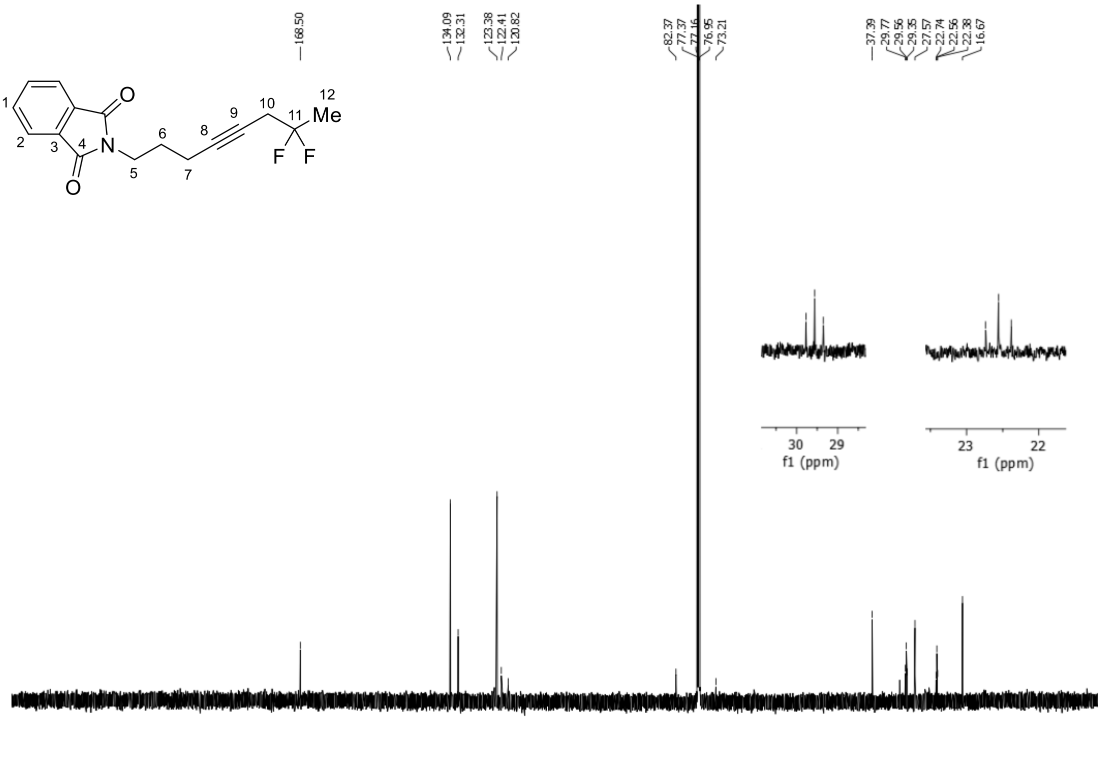

Supplementary Figure 168. $^{13}C\{^{1}H\}$ NMR of **22** (151 MHz, 299 K, $CDCl_3$).

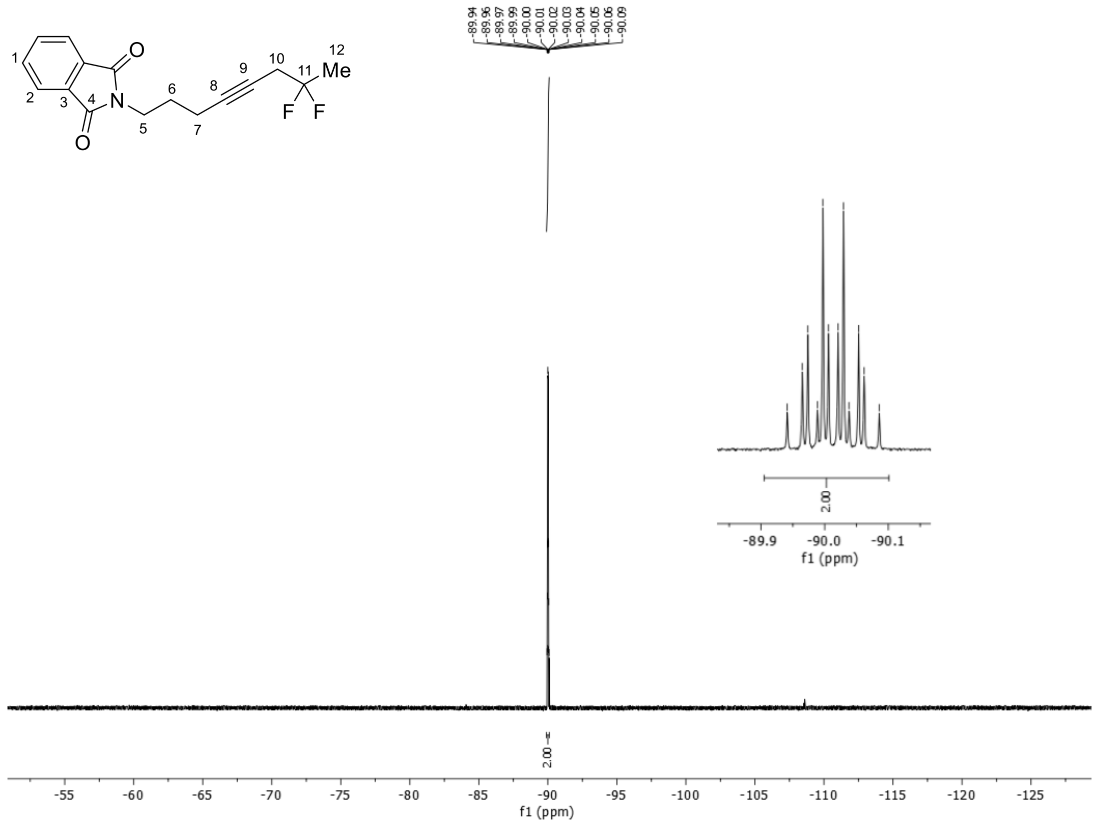

Supplementary Figure 169. ^{19}F NMR of **22** (564 MHz, 299 K, $CDCl_3$).

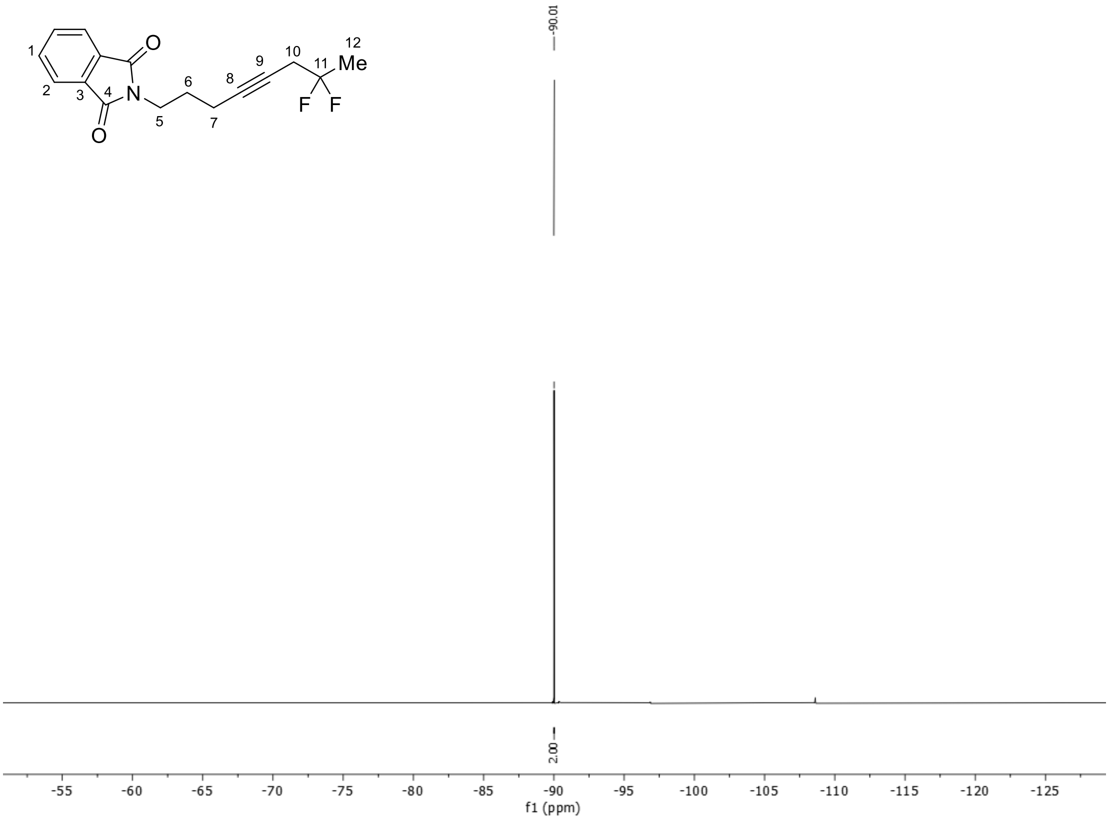

Supplementary Figure 170. $^{19}F\{^{1}H\}$ NMR of **22** (564 MHz, 299 K, $CDCl_3$).

4-(((14,14-Difluoropentadec-11-yn-1-yl)oxy)methyl)-1,2-difluorobenzene (23)

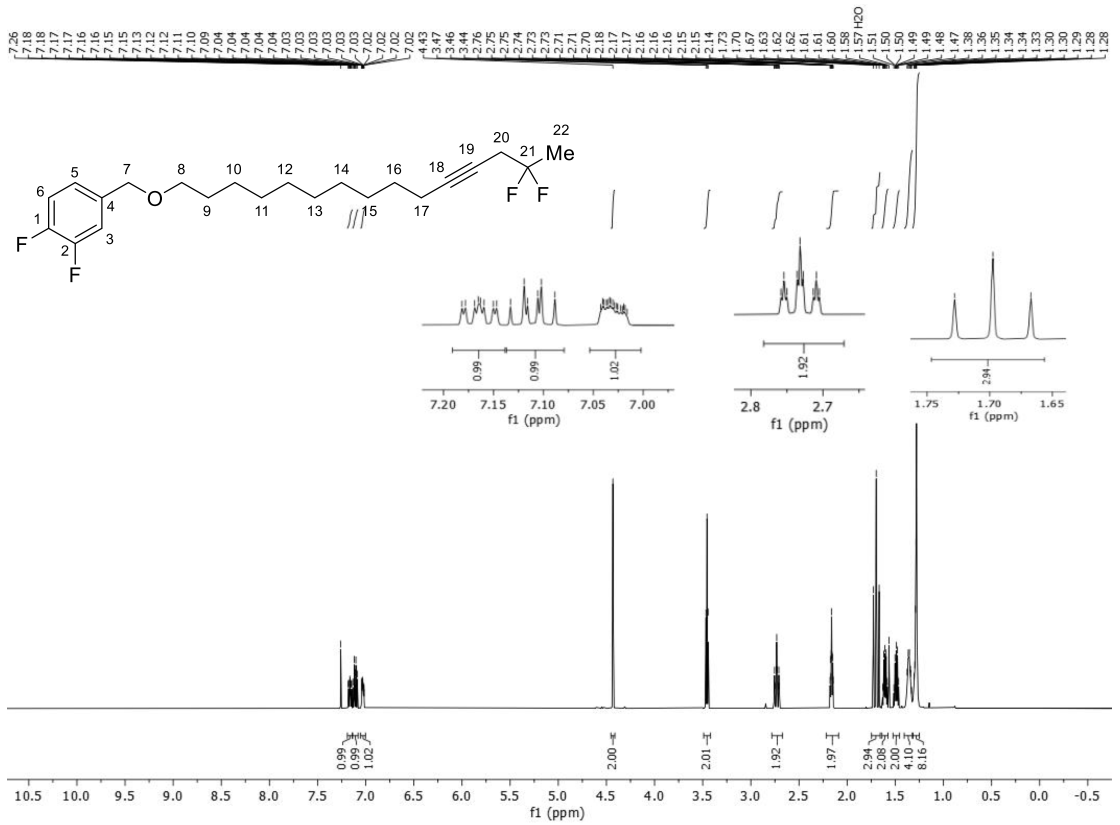

Supplementary Figure 171. ^{1}H NMR of **23** (599 MHz, 299 K, $CDCl_3$).

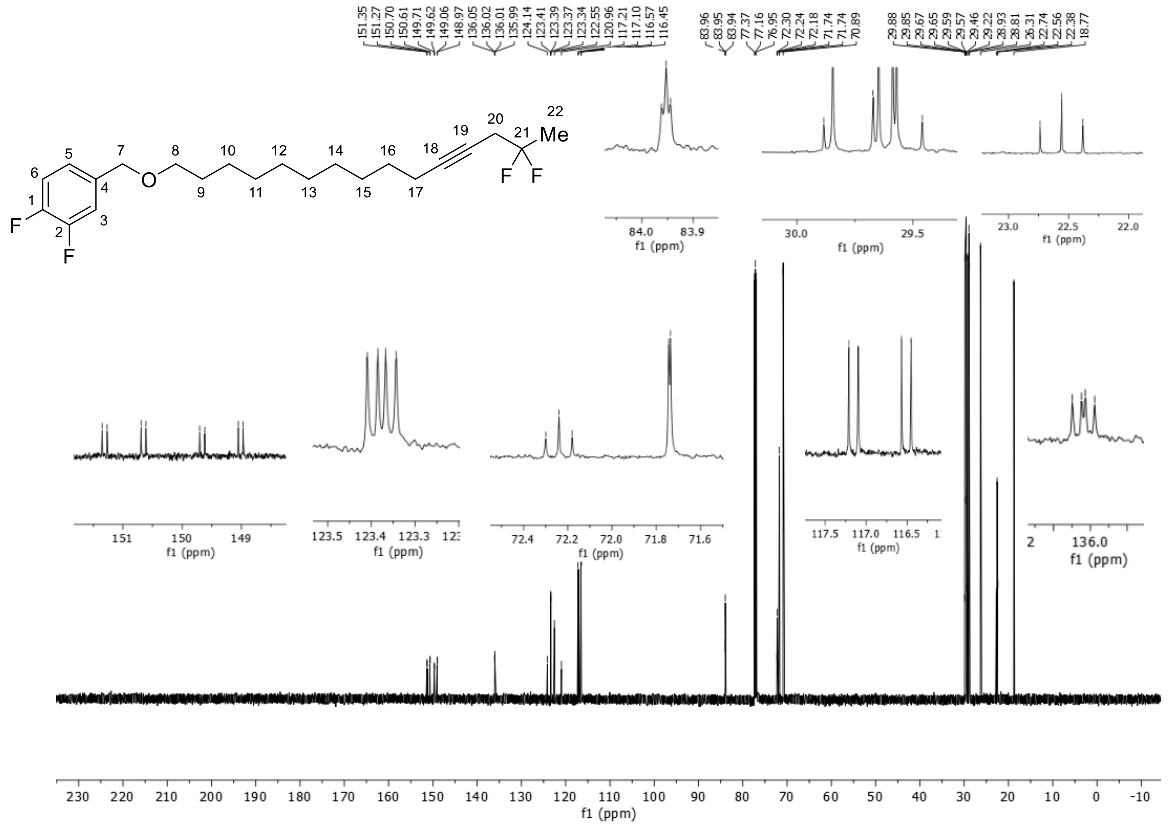

Supplementary Figure 172. $^{13}C\{^{1}H\}$ NMR of **23** (151 MHz, 299 K, $CDCl_3$).

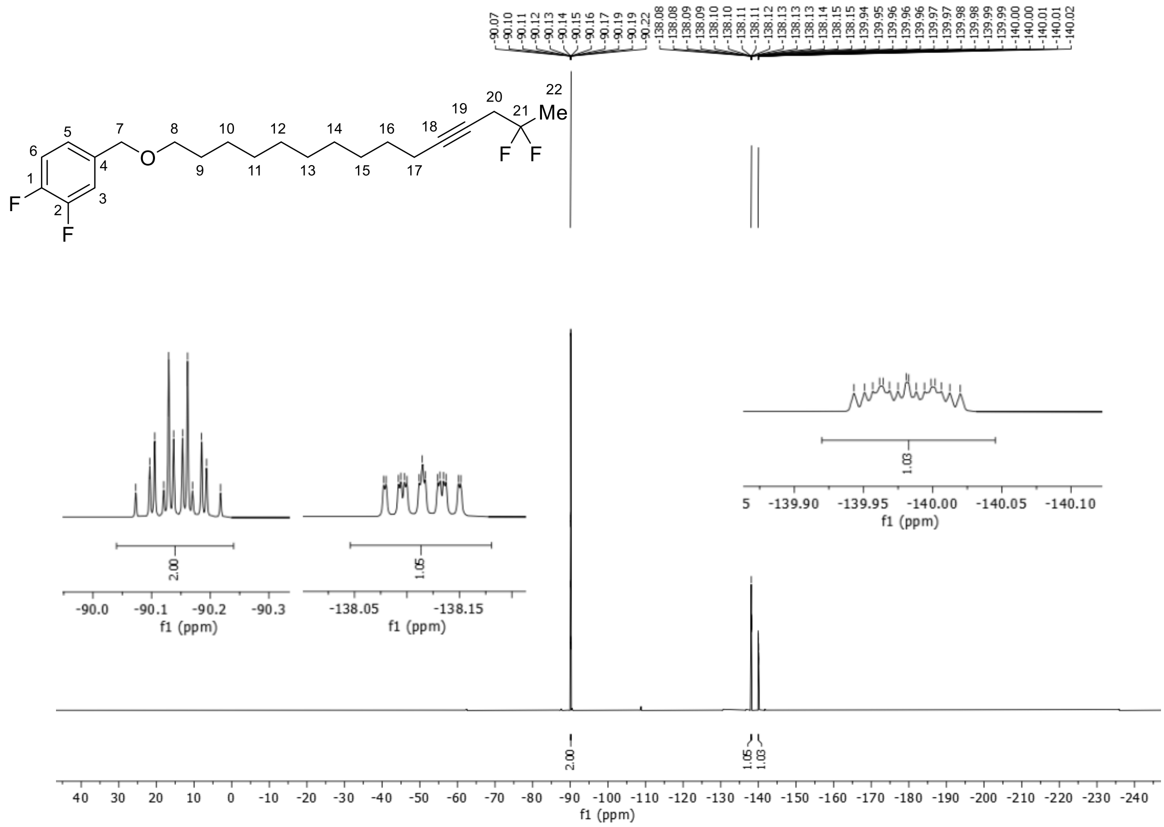

Supplementary Figure 173. ^{19}F NMR of **23** (564 MHz, 299 K, $CDCl_3$).

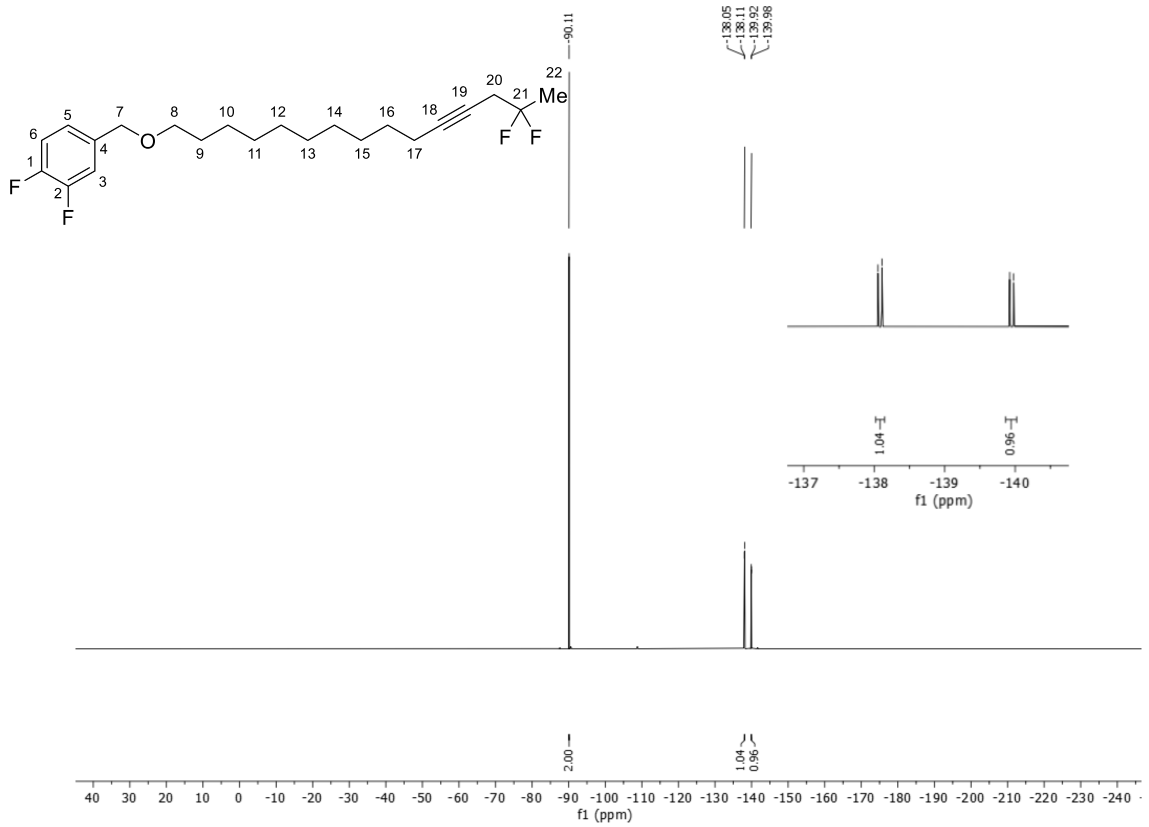

Supplementary Figure 174. $^{19}F\{^1H\}$ NMR of **23** (377 MHz, 299 K, $CDCl_3$).

(5,5-Difluorohex-2-yn-1-yl)benzene (24)

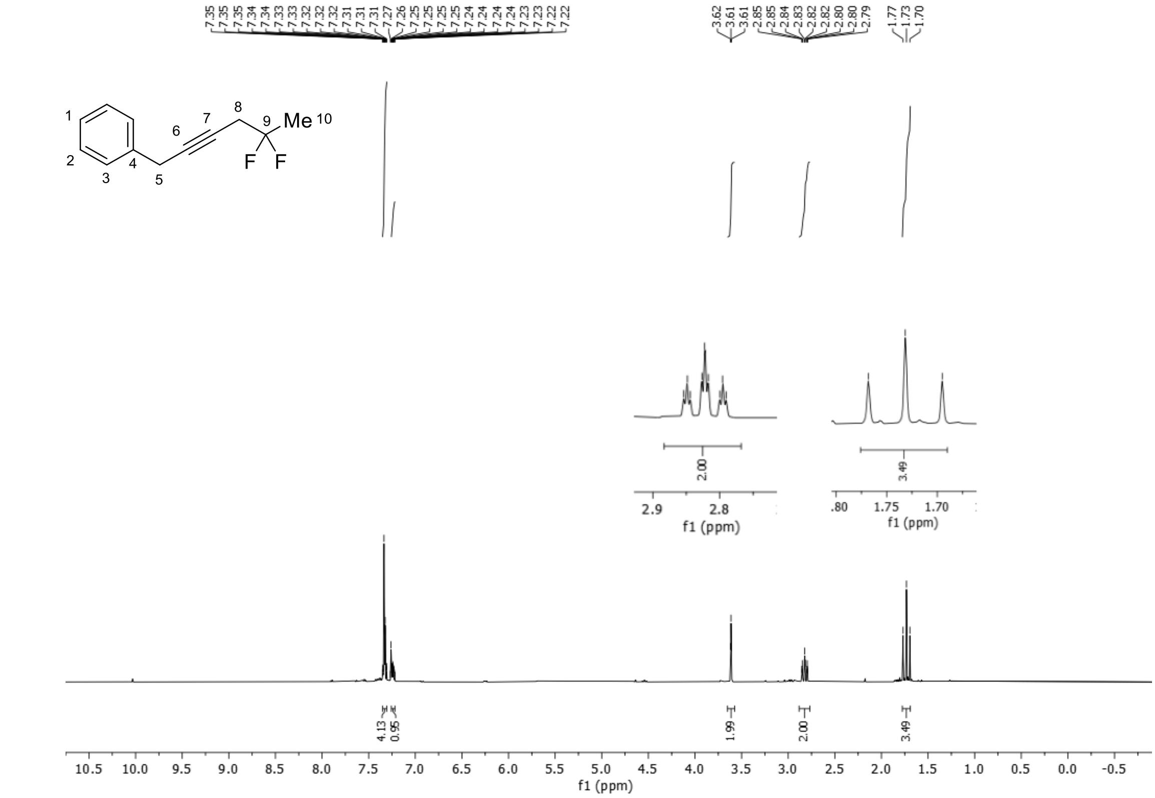

Supplementary Figure 175. ^{1}H NMR of **24** (599 MHz, 299 K, $CDCl_3$).

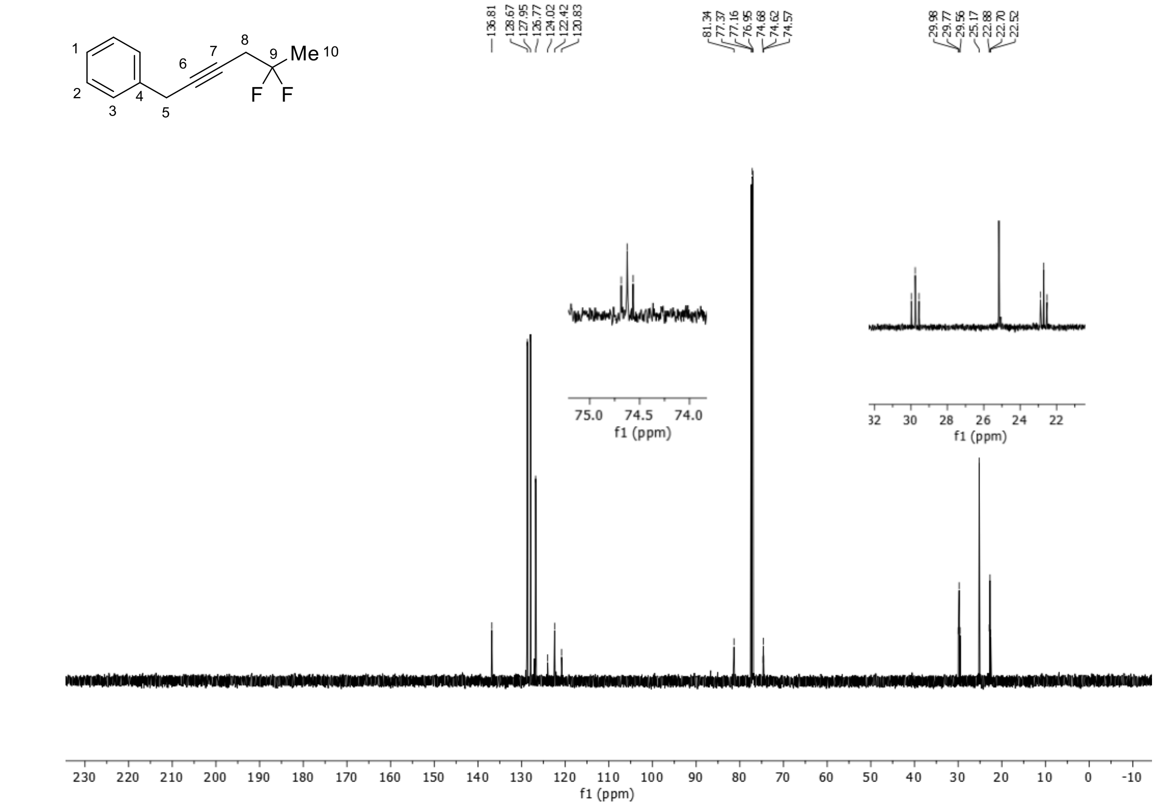

Supplementary Figure 176. $^{13}C\{^{1}H\}$ NMR of **24** (151 MHz, 299 K, $CDCl_3$).

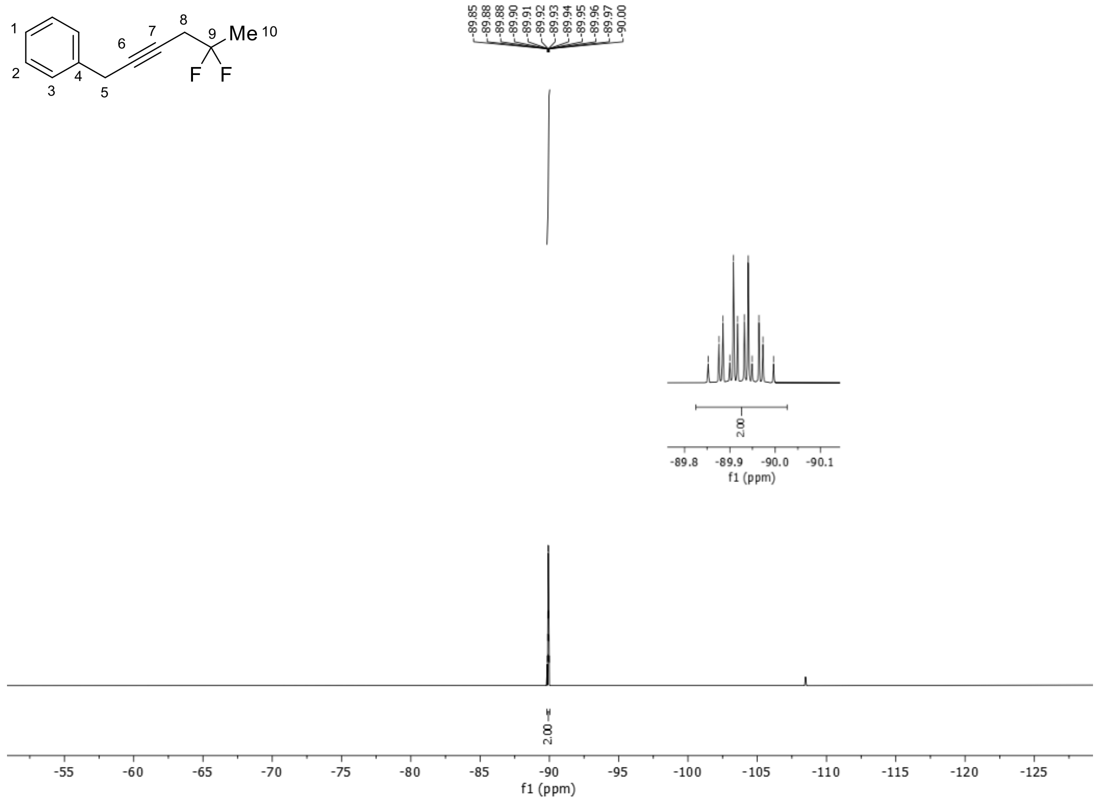

Supplementary Figure 177. ^{19}F NMR of **24** (564 MHz, 299 K, $CDCl_3$).

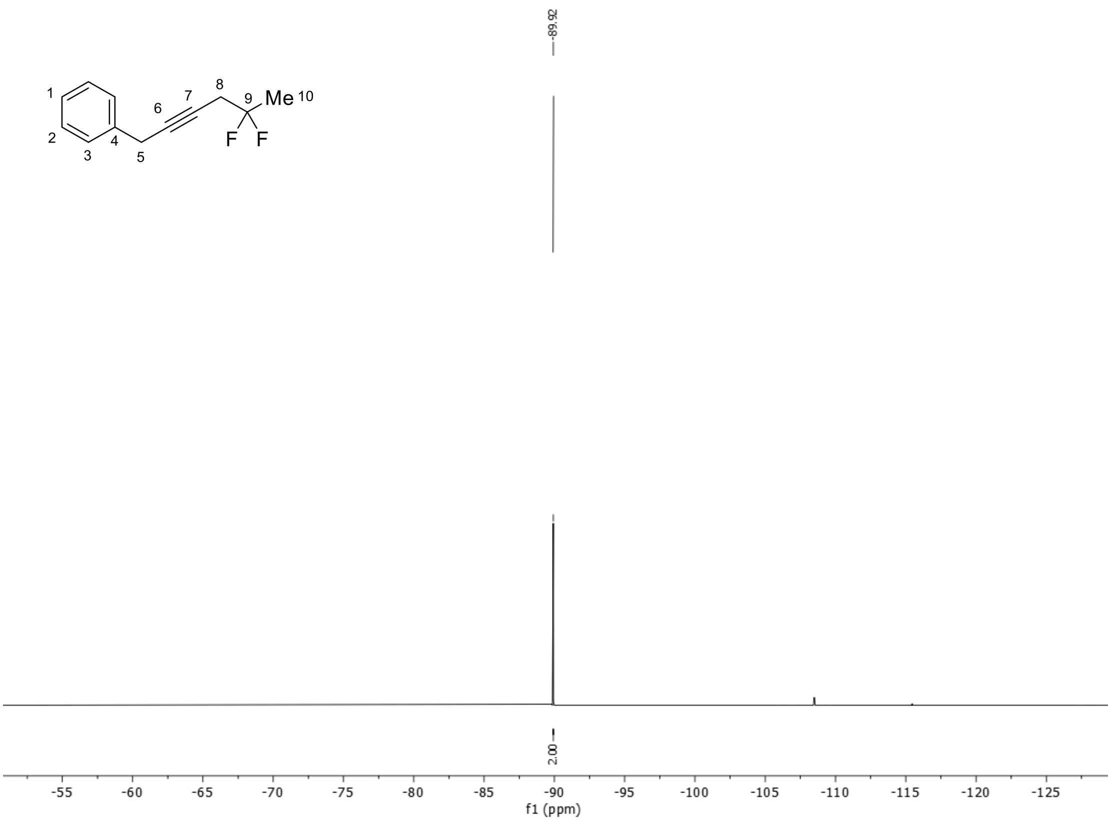

Supplementary Figure 178. $^{19}F\{^1H\}$ NMR of **24** (564 MHz, 299 K, $CDCl_3$).

14,14-Difluoropentadec-11-yn-1-yl (*E*)-3-(4-bromophenyl)acrylate (25)

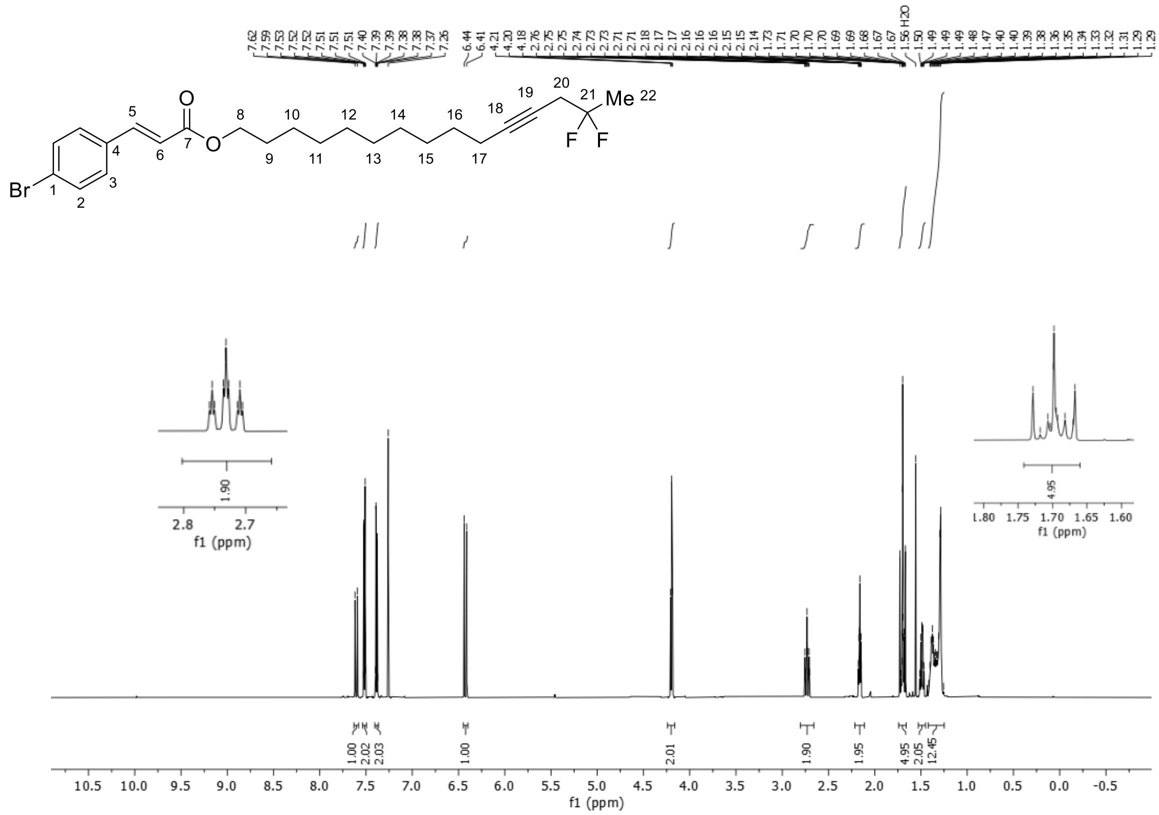

Supplementary Figure 179. ^{1}H NMR of **25** (599 MHz, 299 K, $CDCl_3$).

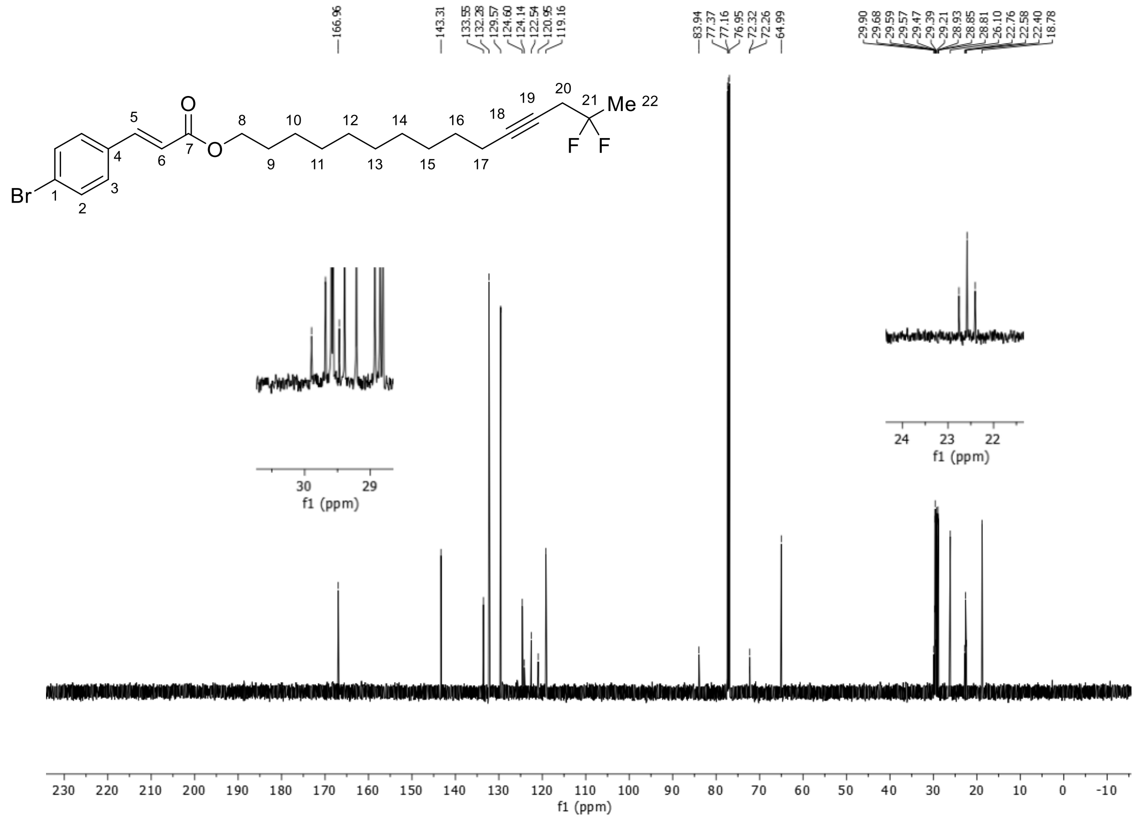

Supplementary Figure 180. $^{13}C\{^{1}H\}$ NMR of **25** (151 MHz, 299 K, $CDCl_3$).

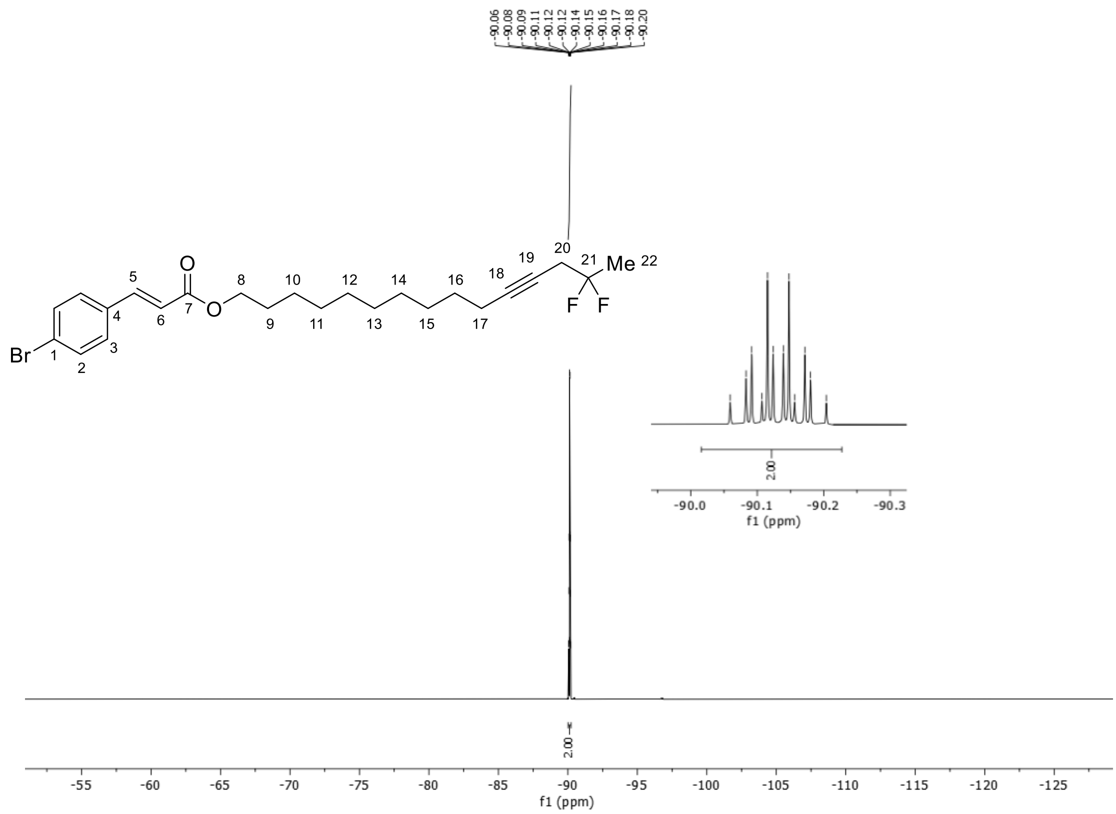

Supplementary Figure 181. ^{19}F NMR of **25** (564 MHz, 299 K, $CDCl_3$).

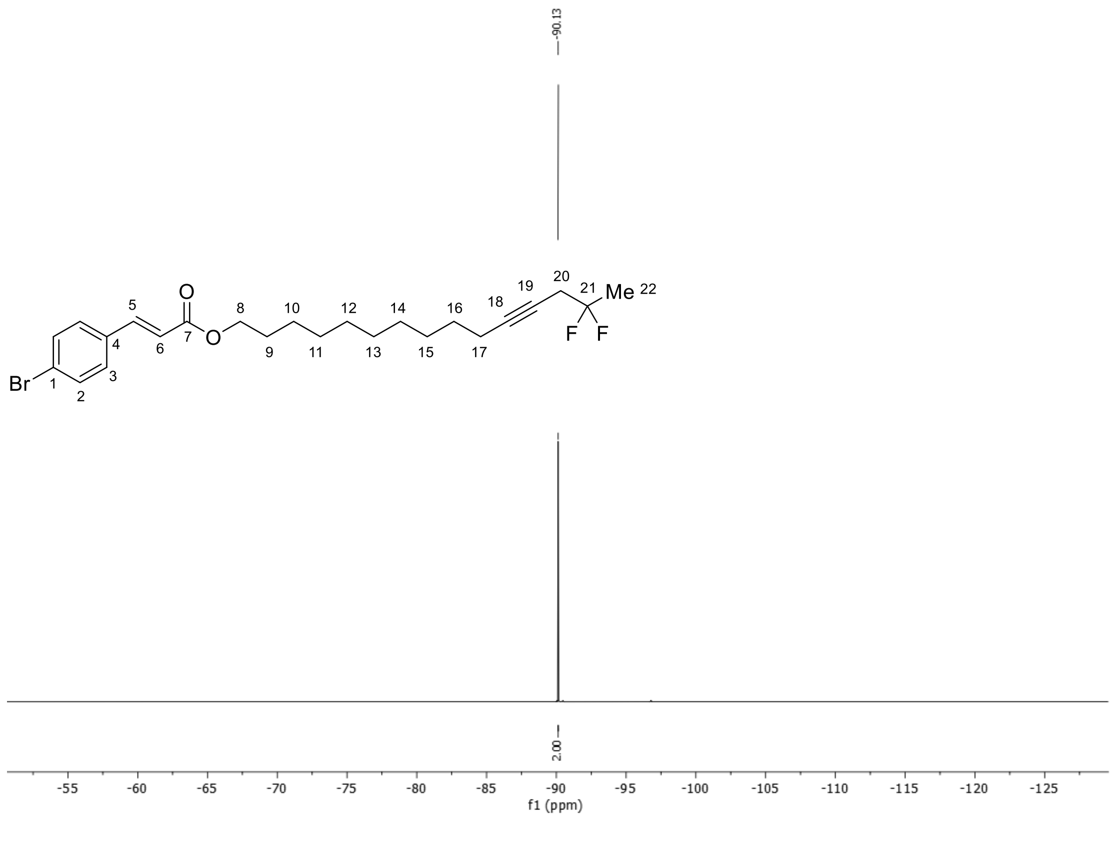

Supplementary Figure 182. ^{19}F{^{1}H} NMR of **25** (564 MHz, 299 K, $CDCl_3$).

14,14-Difluoropentadec-11-yn-1-yl hex-2-ynoate (26)

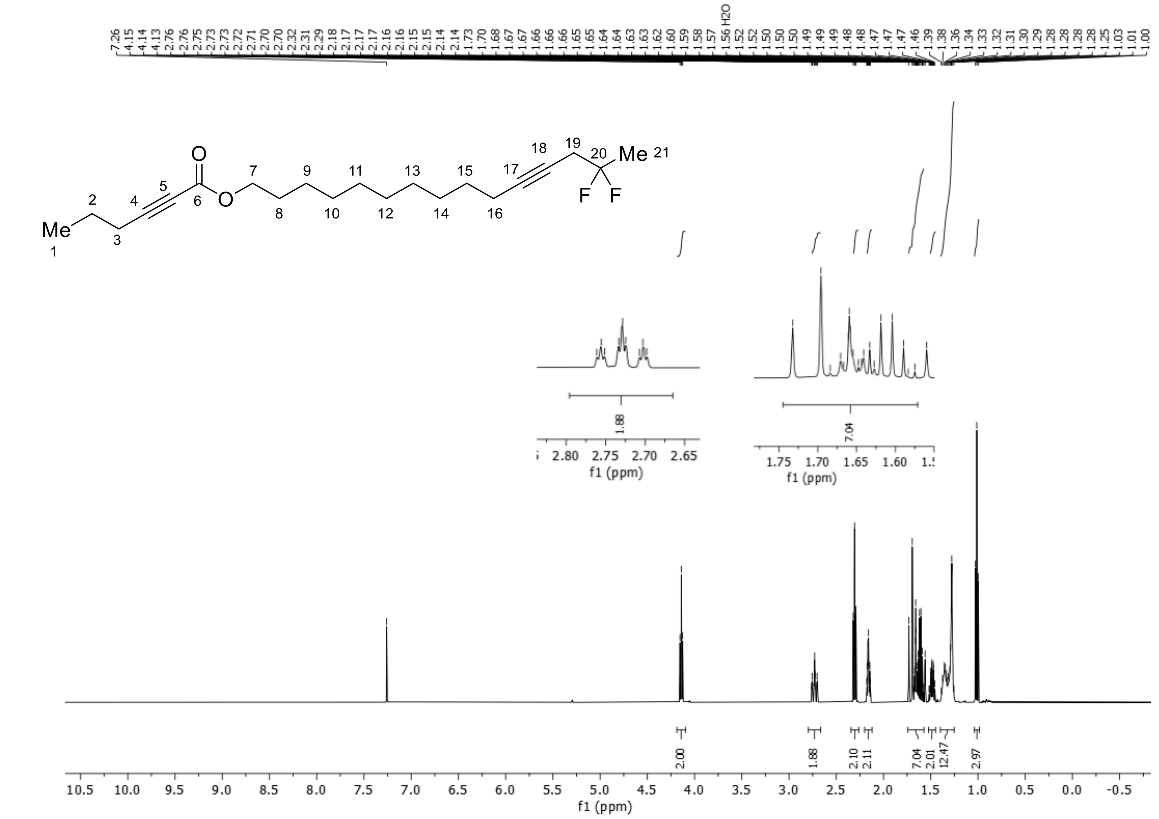

Supplementary Figure 183. ^{1}H NMR of **26** (500 MHz, 299 K, $CDCl_3$).

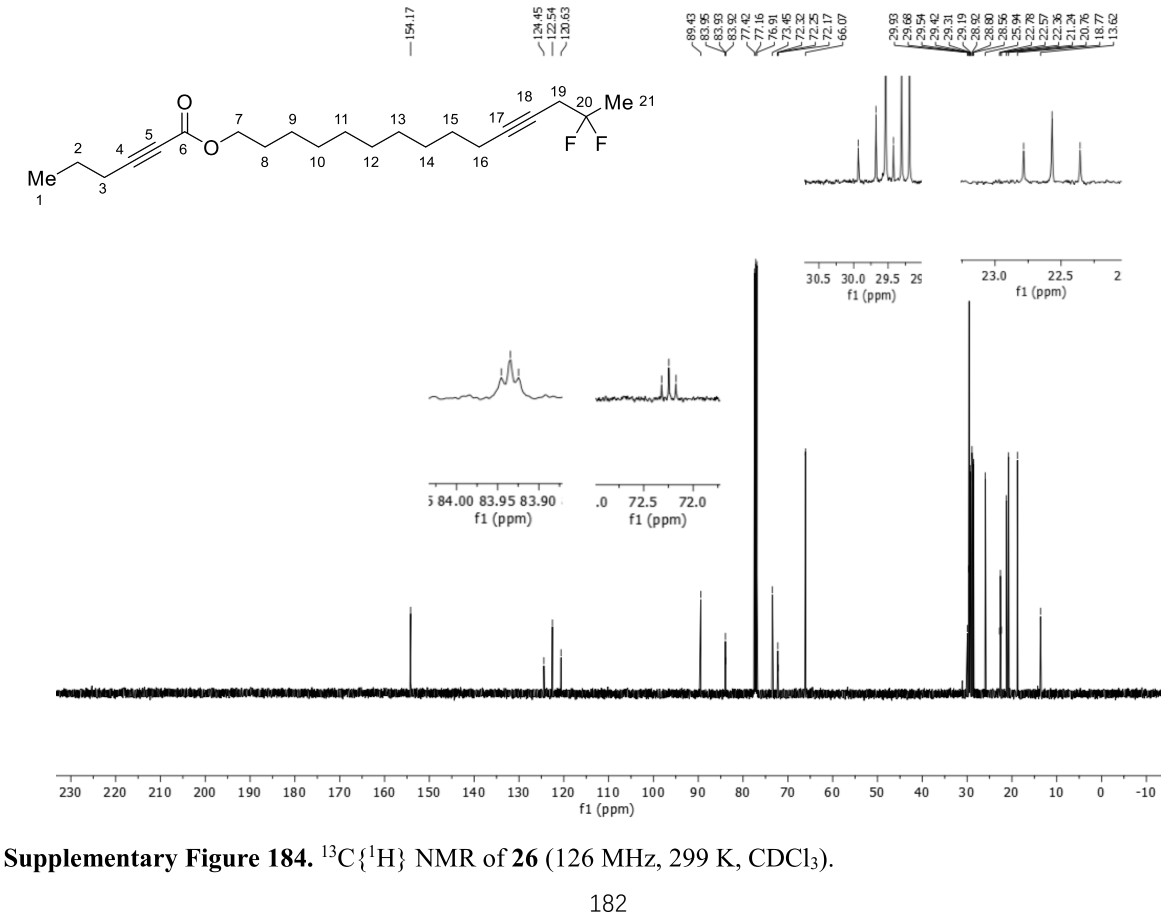

Supplementary Figure 184. $^{13}C\{^{1}H\}$ NMR of **26** (126 MHz, 299 K, $CDCl_3$).

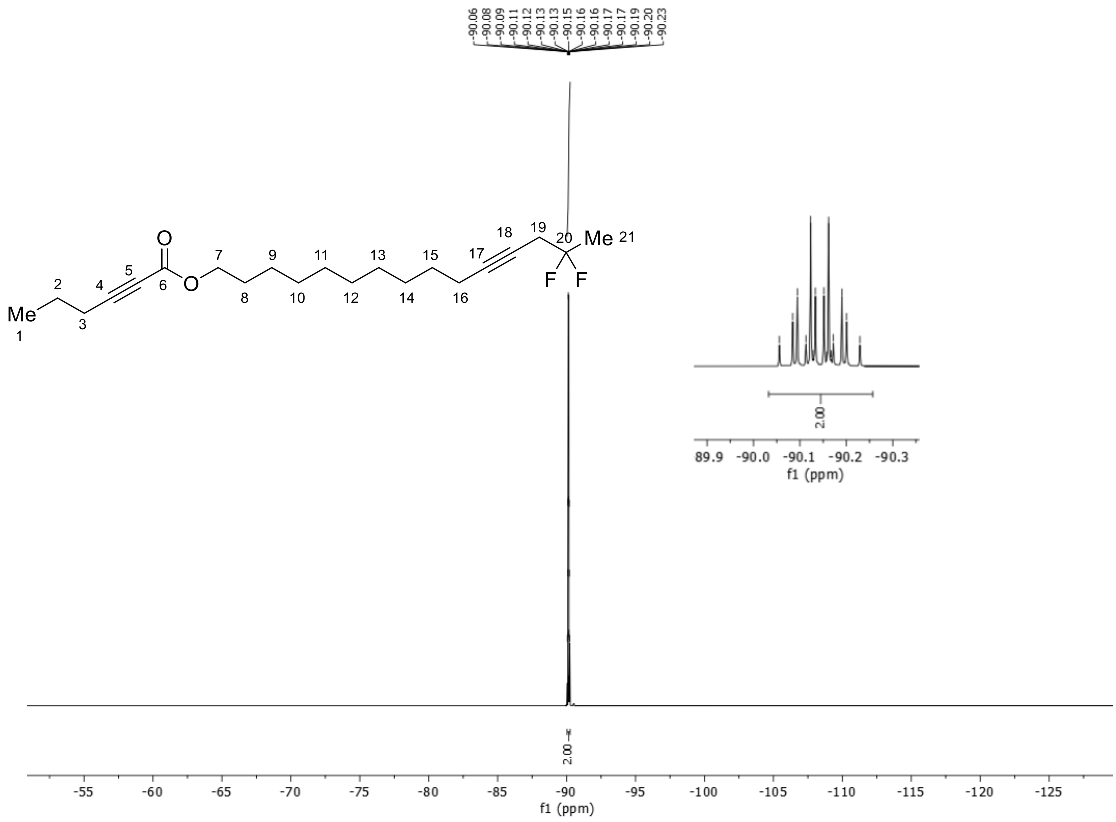

Supplementary Figure 185. ^{19}F NMR of **26** (470 MHz, 299 K, $CDCl_3$).

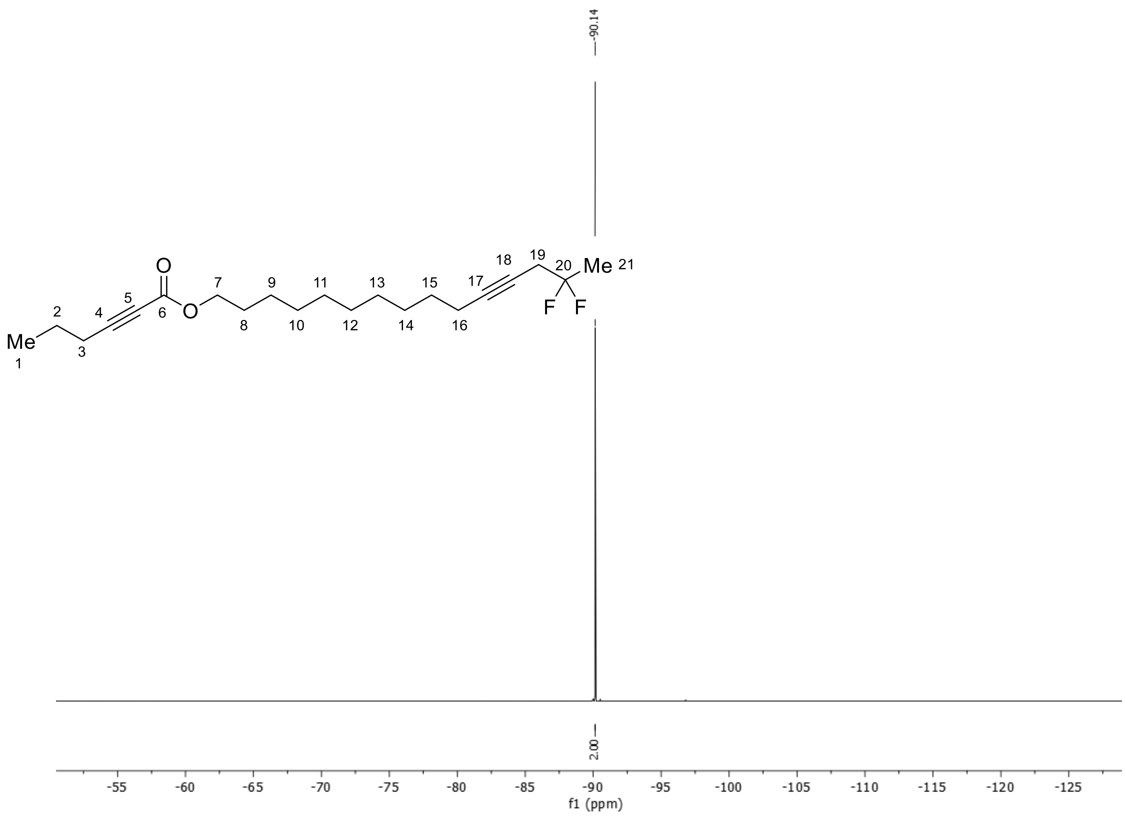

Supplementary Figure 186. $^{19}F\{^{1}H\}$ NMR of **26** (470 MHz, 299 K, $CDCl_3$).

14,14-Difluoropentadec-11-yn-1-yl 4-methylbenzenesulfonate (27)

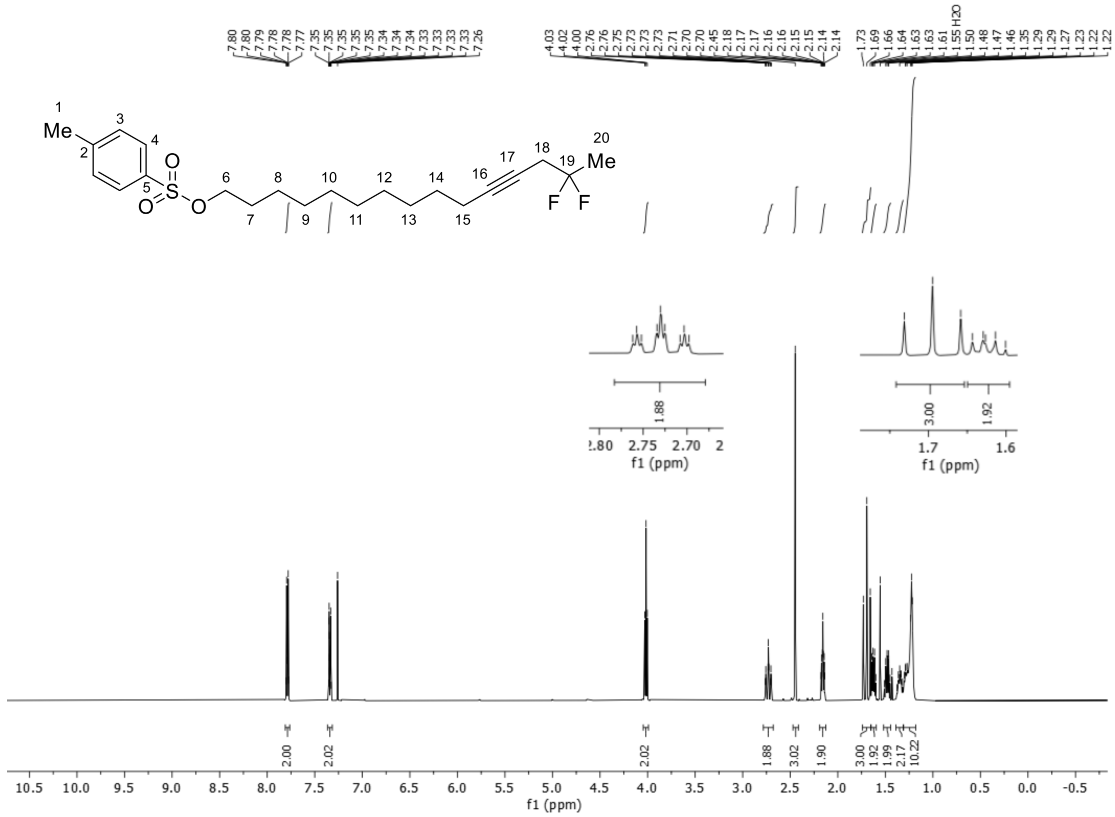

Supplementary Figure 187. ^{1}H NMR of **27** (500 MHz, 299 K, $CDCl_3$).

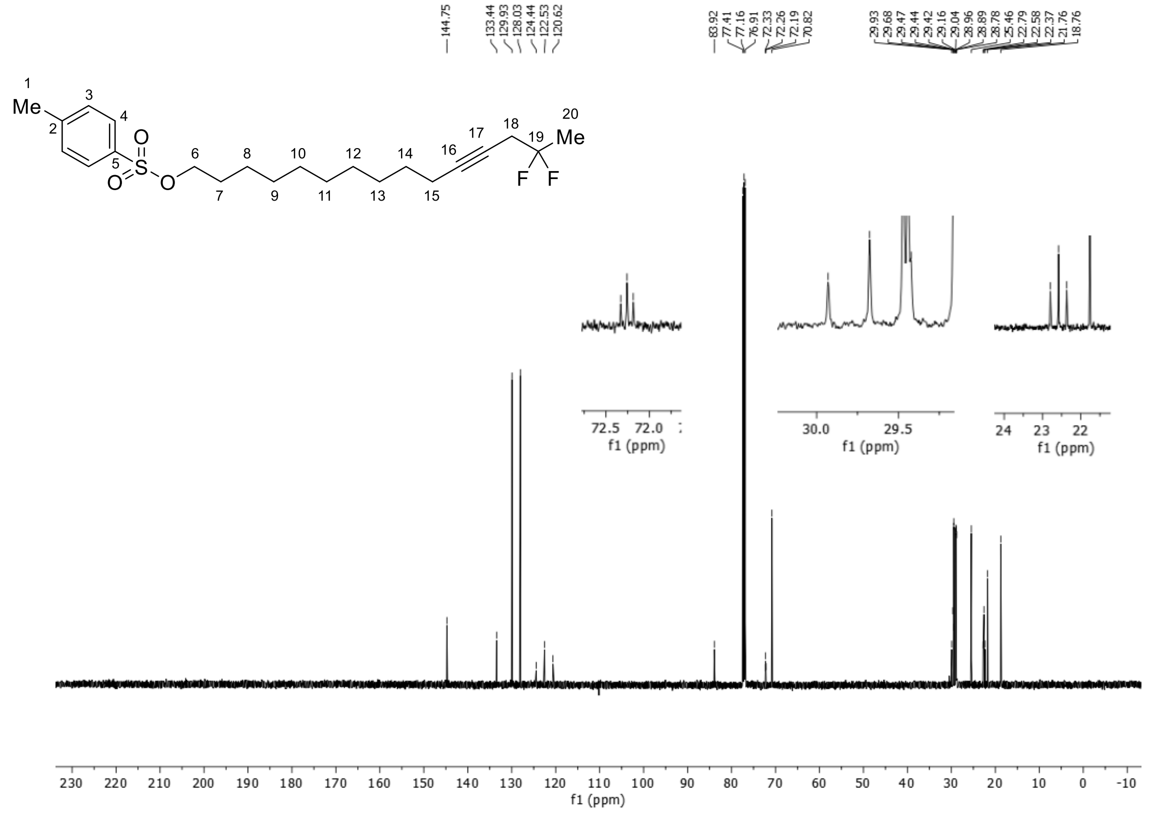

Supplementary Figure 188. $^{13}C\{^{1}H\}$ NMR of **27** (126 MHz, 299 K, $CDCl_3$).

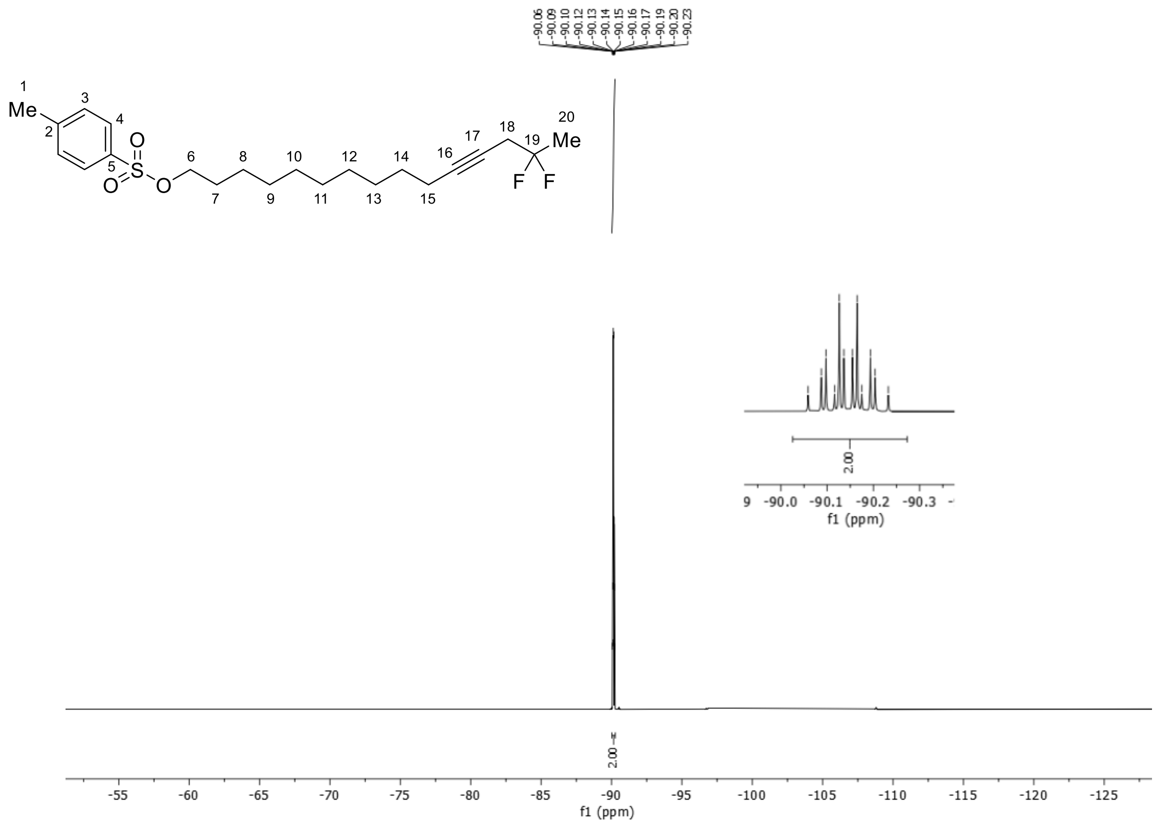

Supplementary Figure 189. ^{19}F NMR of **27** (470 MHz, 299 K, $CDCl_3$).

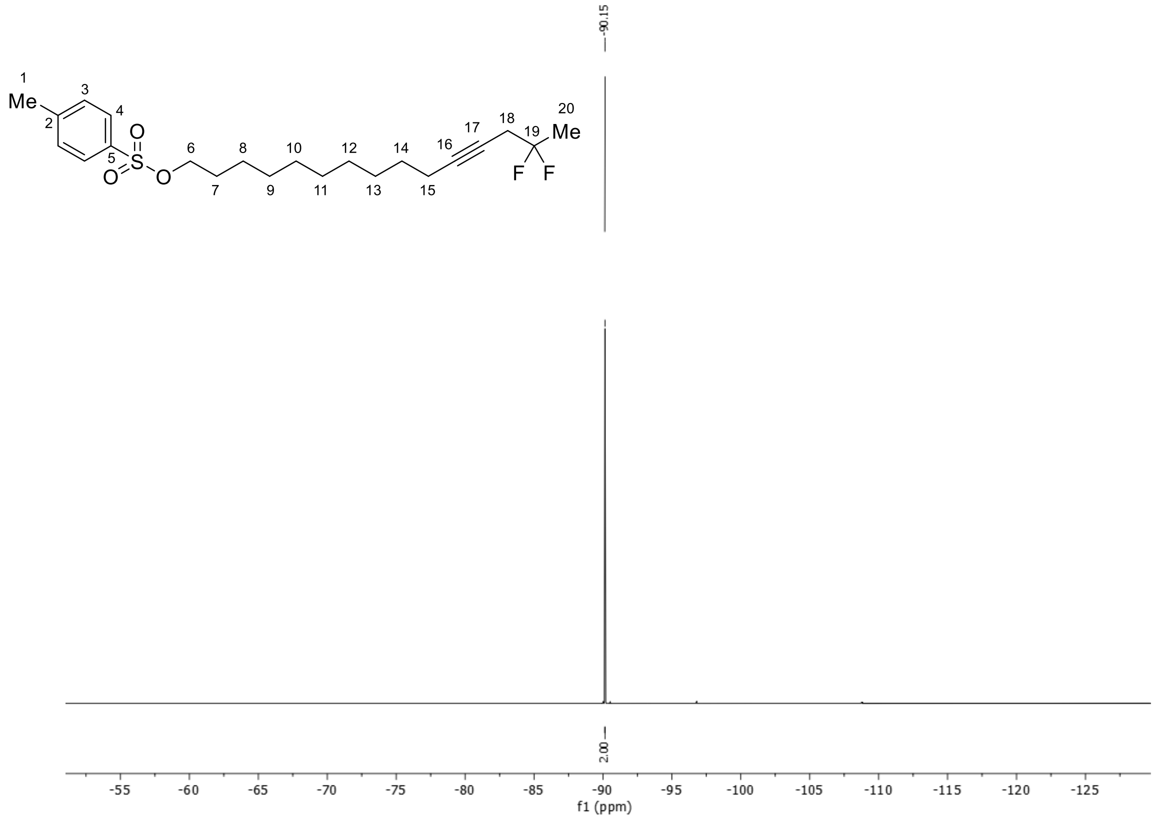

Supplementary Figure 190. ^{19}F{^{1}H} NMR of **27** (470 MHz, 299 K, $CDCl_3$).

14,14-Difluoropentadec-11-yn-1-ol (28)

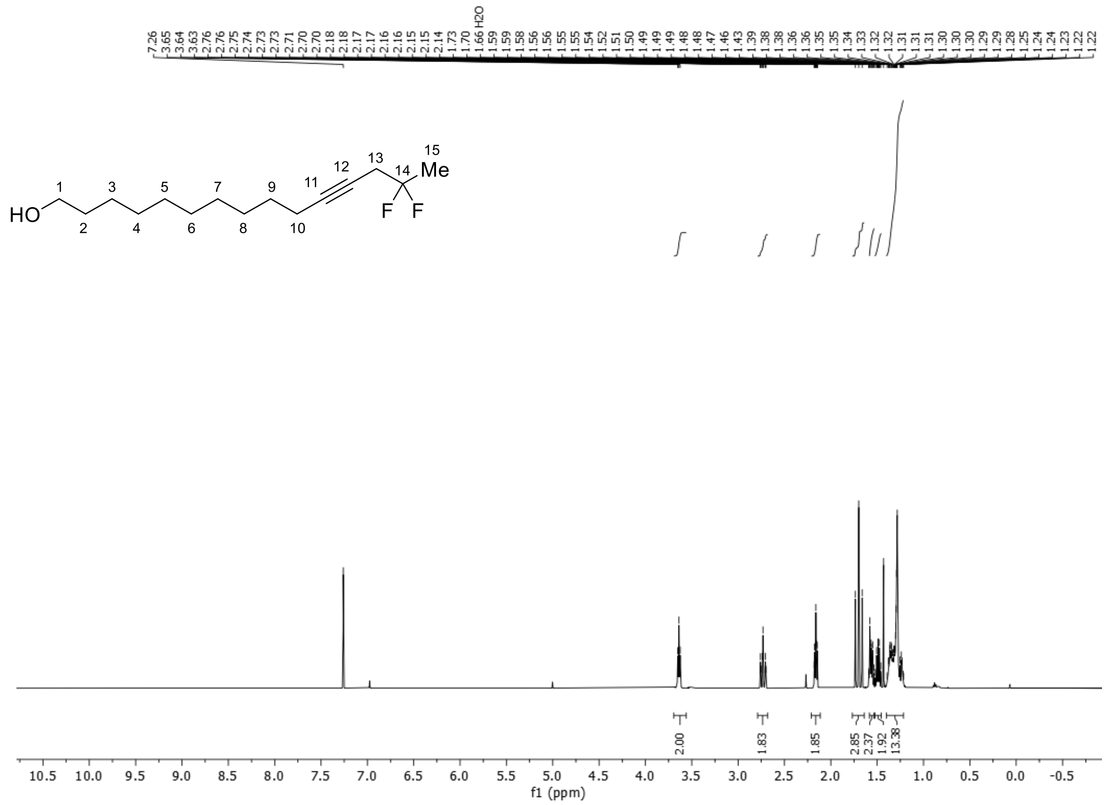

Supplementary Figure 191. ^{1}H NMR of **28** (500 MHz, 299 K, $CDCl_3$).

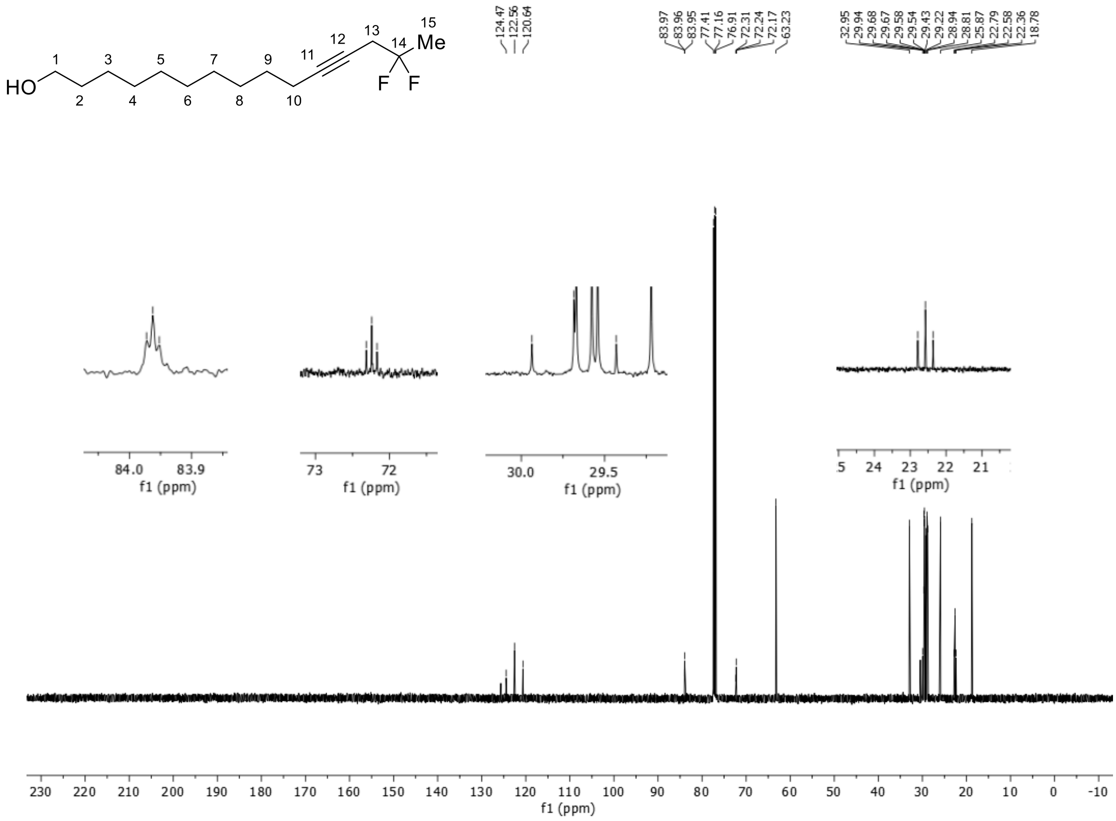

Supplementary Figure 192. $^{13}C\{^{1}H\}$ NMR of **28** (126 MHz, 299 K, $CDCl_3$).

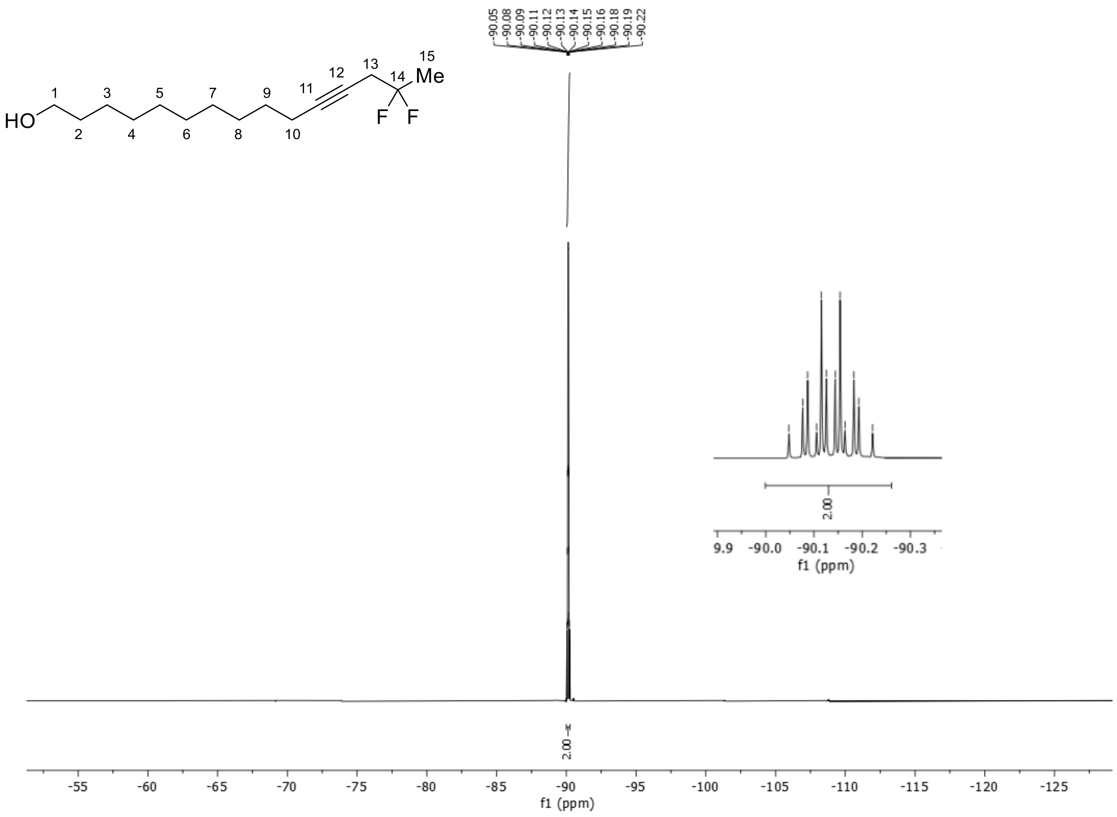

Supplementary Figure 193. ^{19}F NMR of **28** (470 MHz, 299 K, $CDCl_3$).

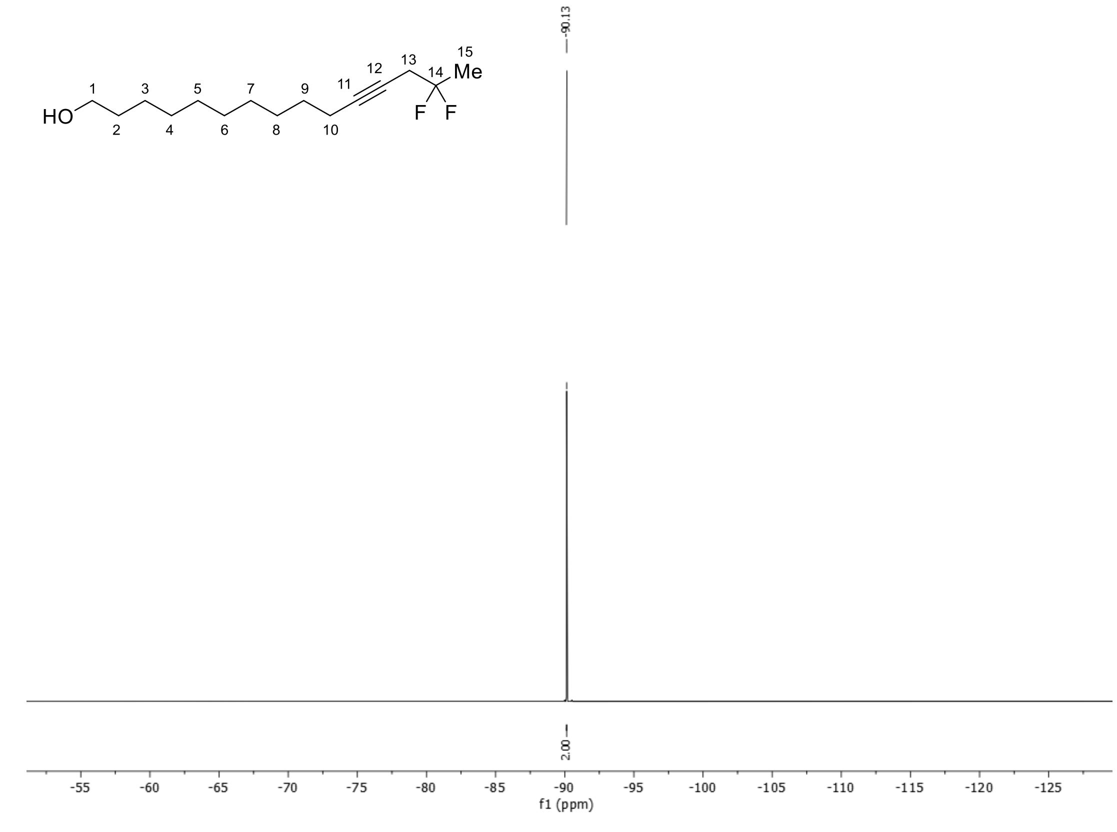

Supplementary Figure 194. ^{19}F{^{1}H} NMR of **28** (470 MHz, 299 K, $CDCl_3$).

3,3-Difluoro-2-methylnonadec-5-yne (29)

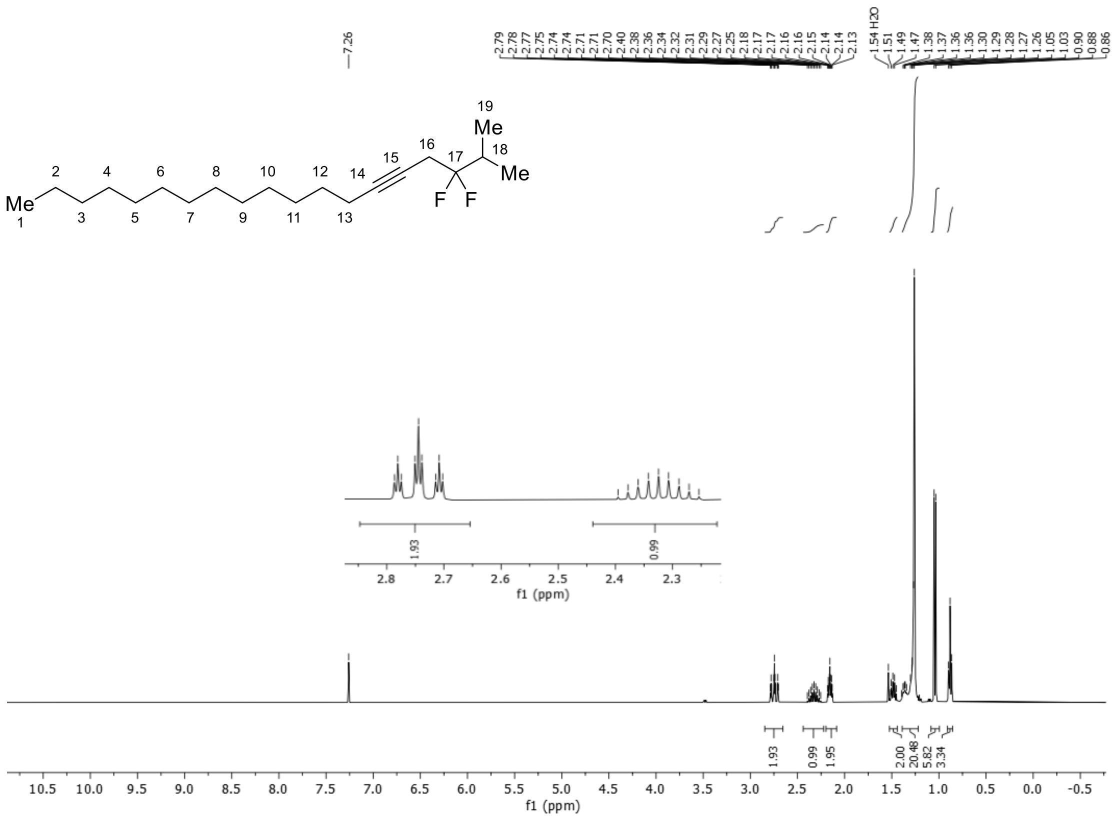

Supplementary Figure 195. ^{1}H NMR of **29** (400 MHz, 299 K, $CDCl_3$).

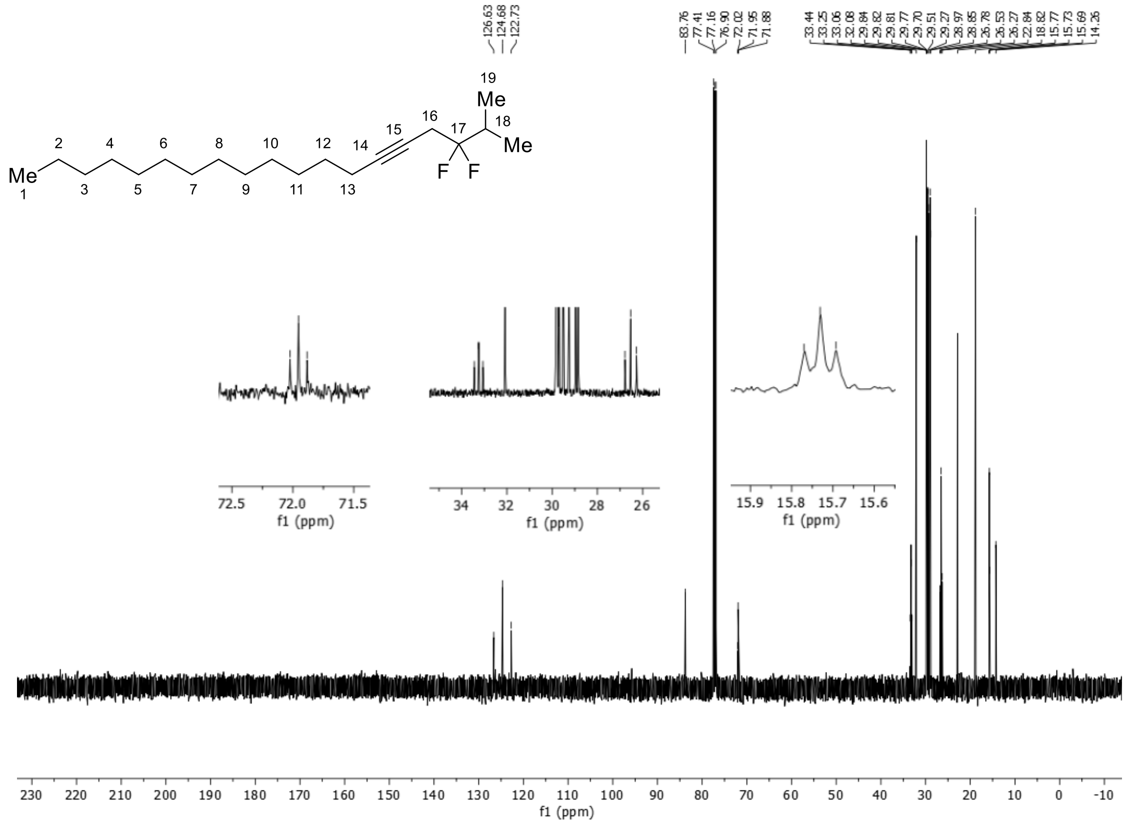

Supplementary Figure 196. $^{13}C\{^{1}H\}$ NMR of **29** (126 MHz, 299 K, $CDCl_3$).

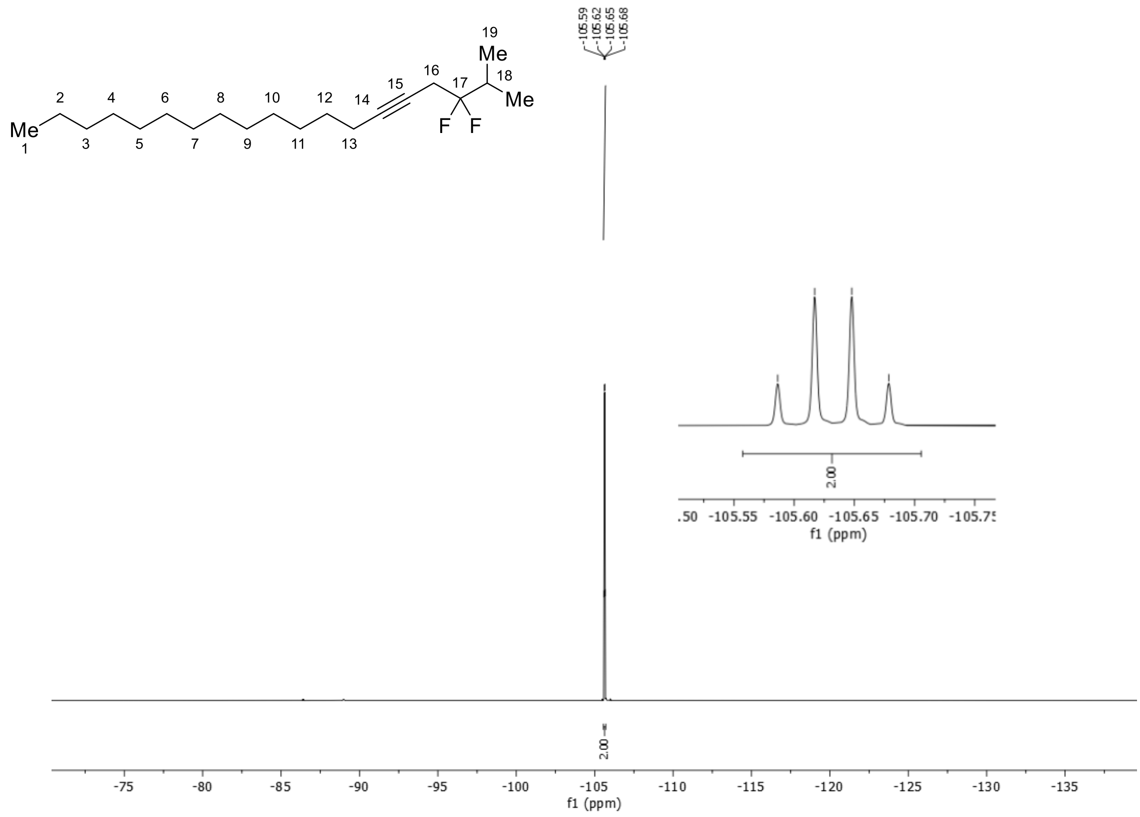

Supplementary Figure 197. ^{19}F NMR of **29** (470 MHz, 299 K, $CDCl_3$).

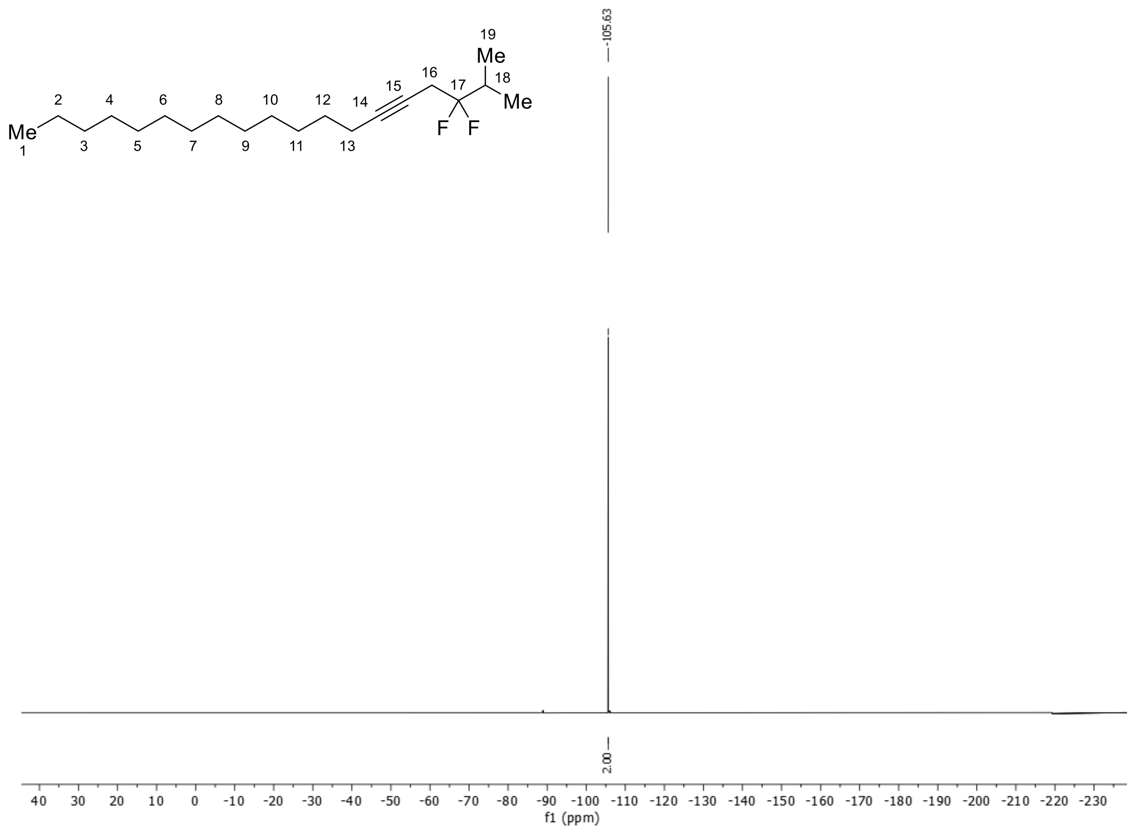

Supplementary Figure 198. $^{19}F\{^1H\}$ NMR of **29** (377 MHz, 299 K, $CDCl_3$).

(1,1-Difluoroheptadec-3-yn-1-yl)cyclohexane (30)

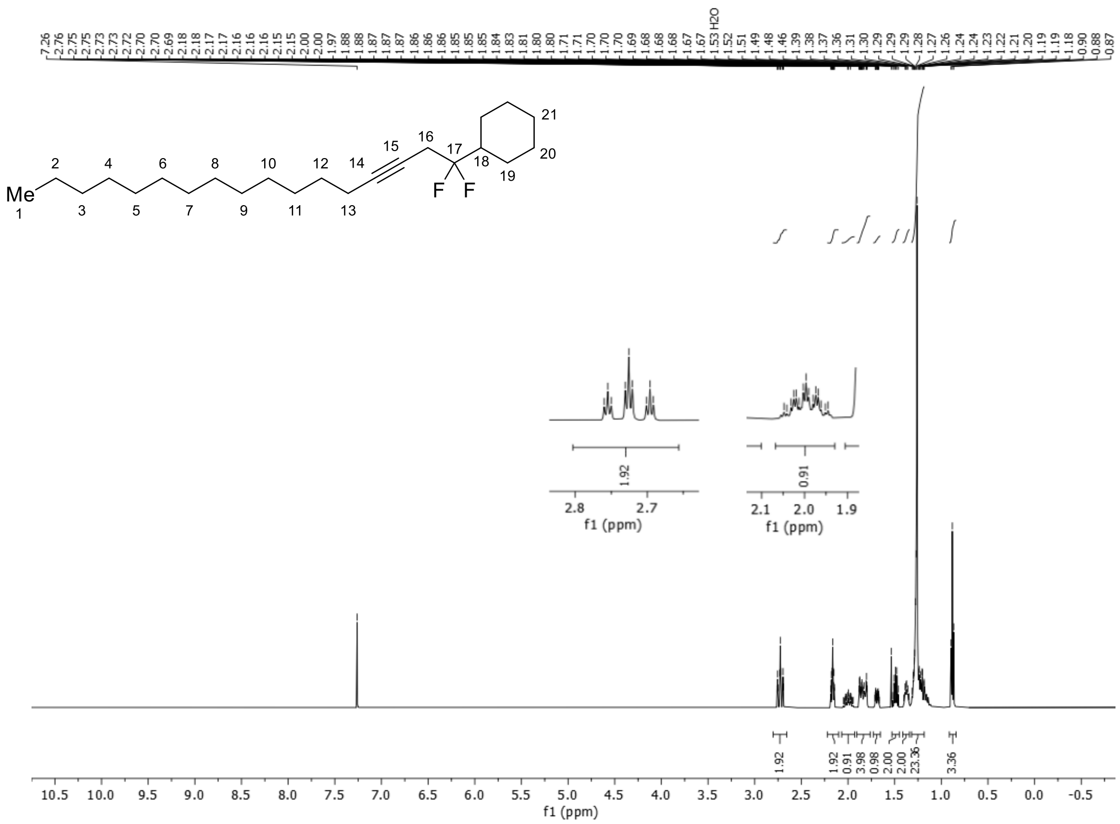

Supplementary Figure 199. ^{1}H NMR of **30** (500 MHz, 299 K, $CDCl_3$).

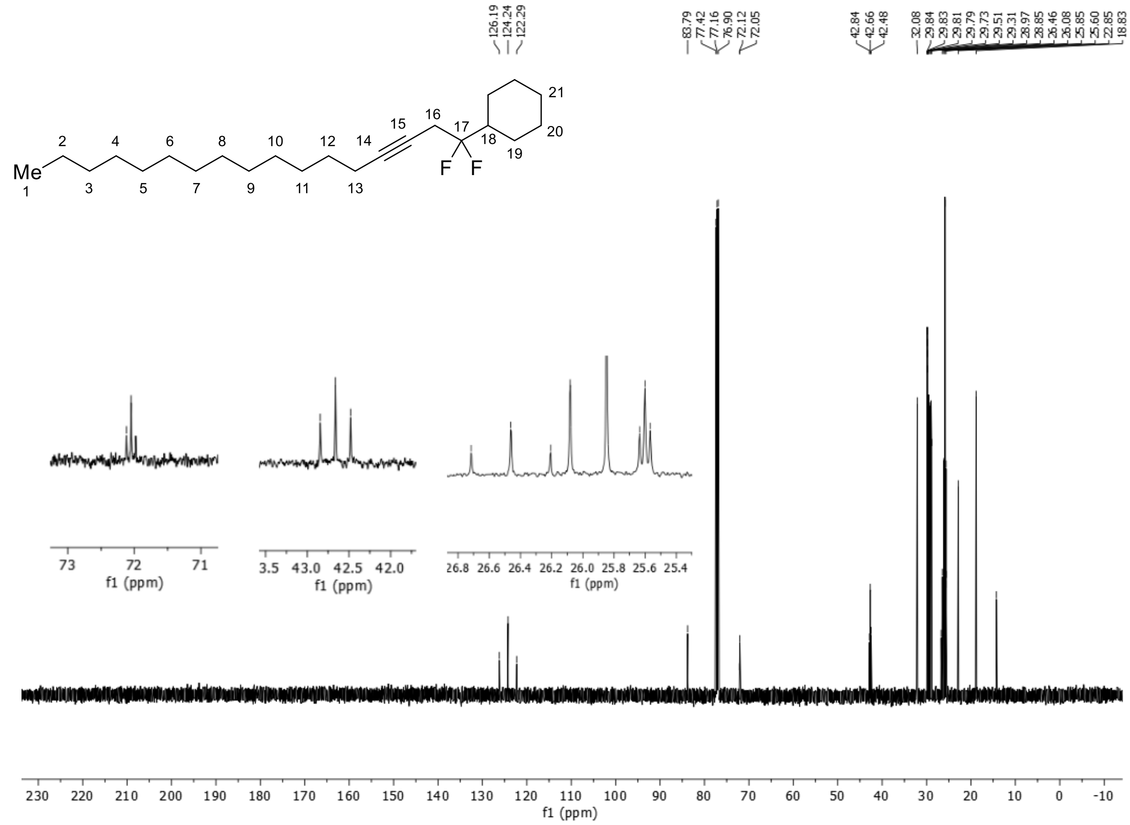

Supplementary Figure 200. $^{13}C\{^{1}H\}$ NMR of **30** (126 MHz, 299 K, $CDCl_3$).

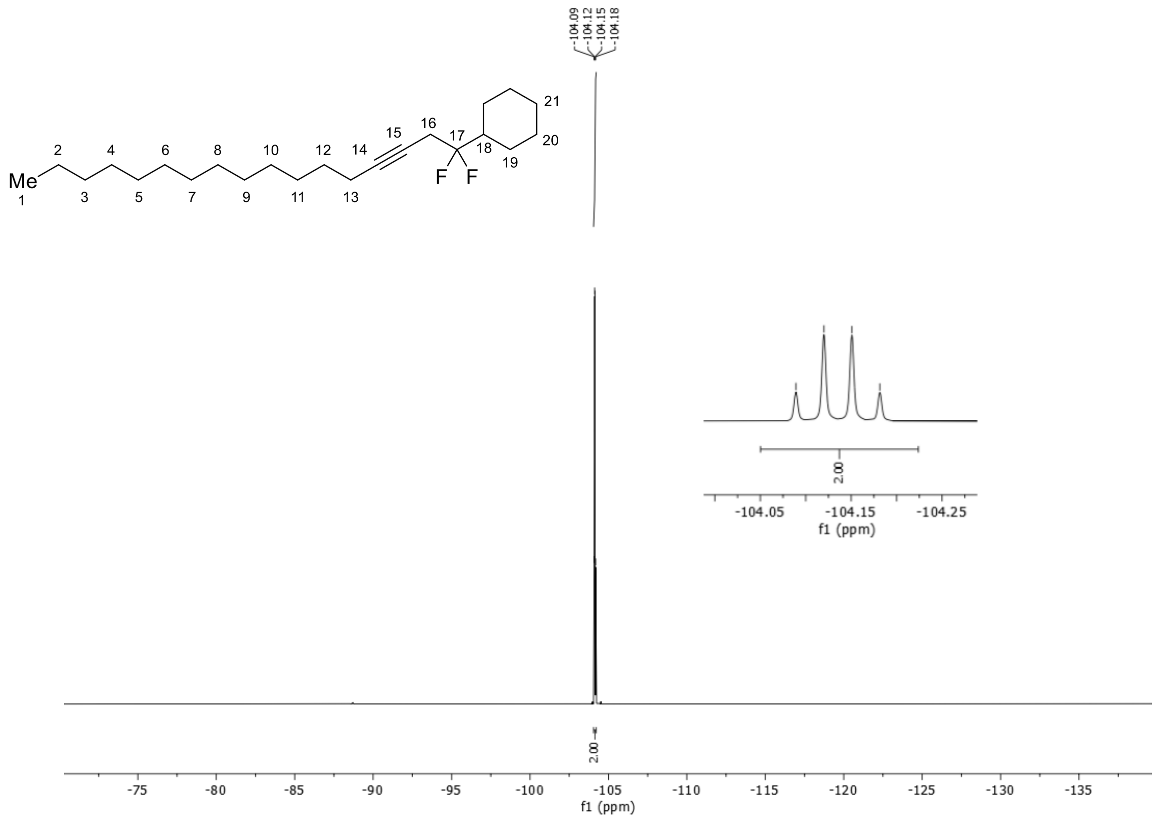

Supplementary Figure 201. ^{19}F NMR of **30** (470 MHz, 299 K, $CDCl_3$).

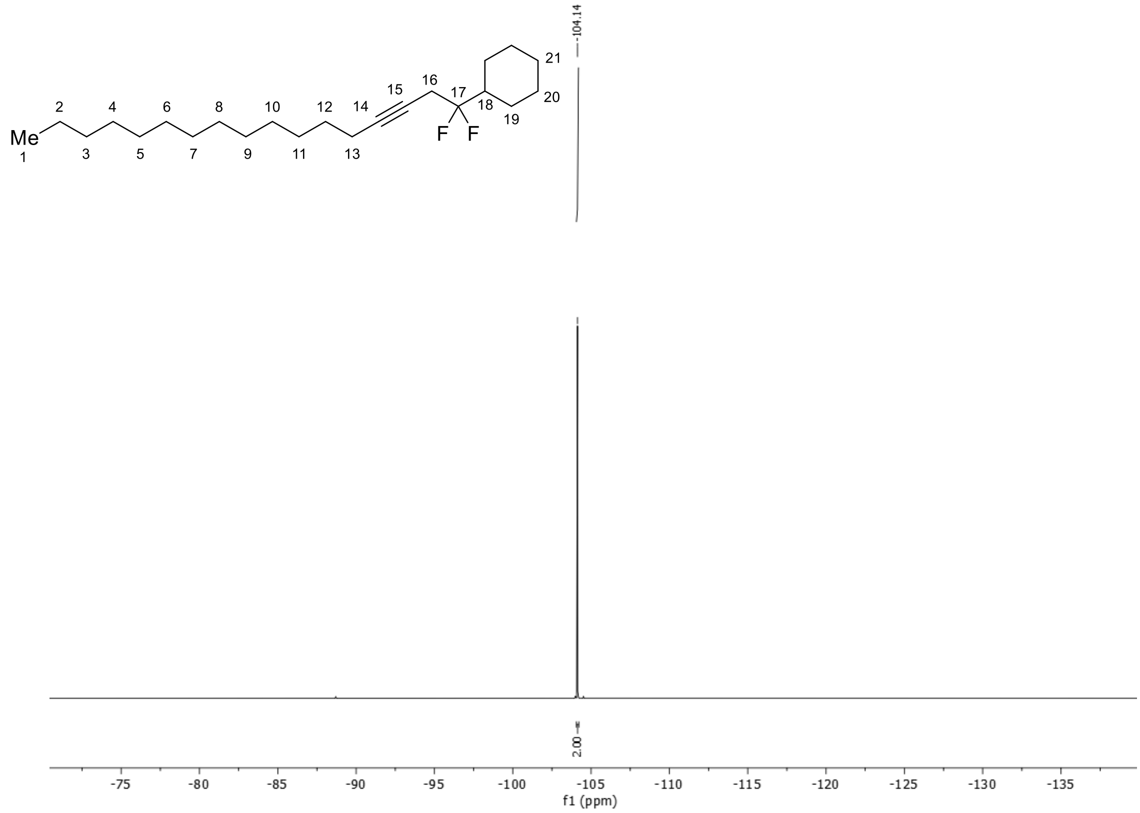

Supplementary Figure 202. ^{19}F{^{1}H} NMR of **30** (377 MHz, 299 K, $CDCl_3$).

1,2,2-Trifluoroheptadec-4-yne (31)

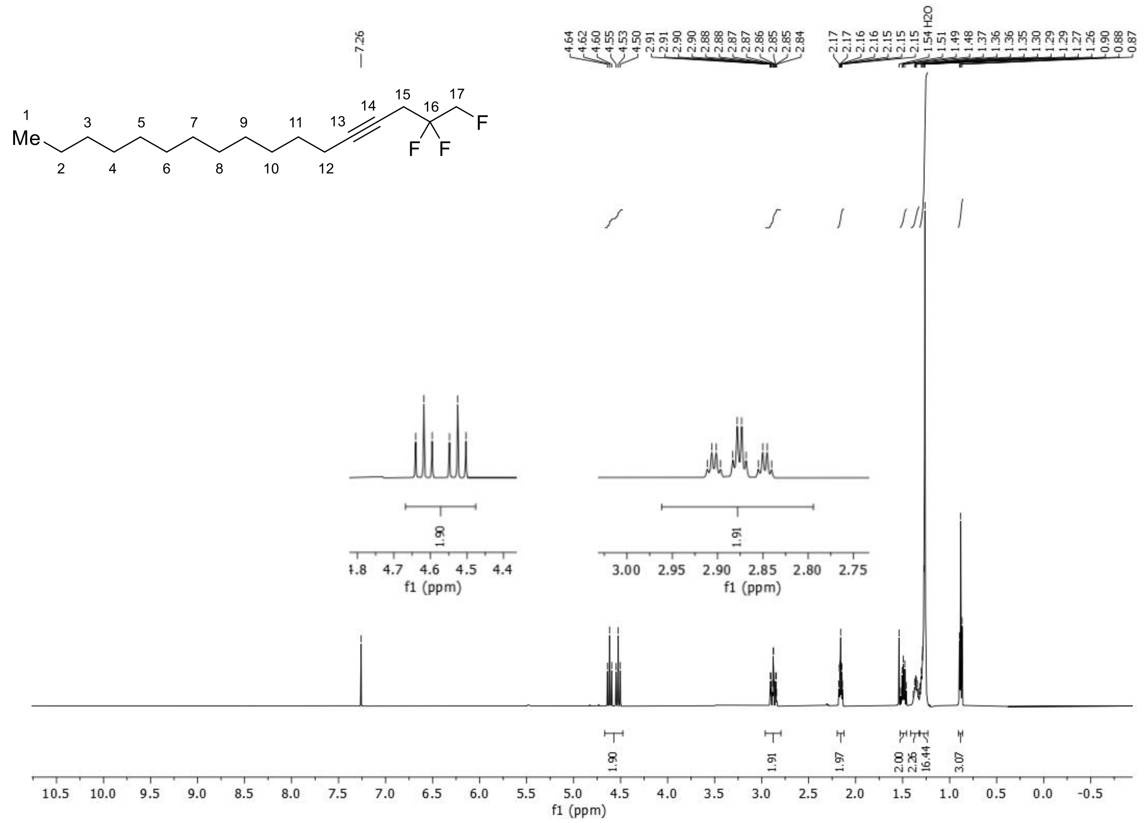

Supplementary Figure 203. ^{1}H NMR of **31** (500 MHz, 299 K, $CDCl_3$).

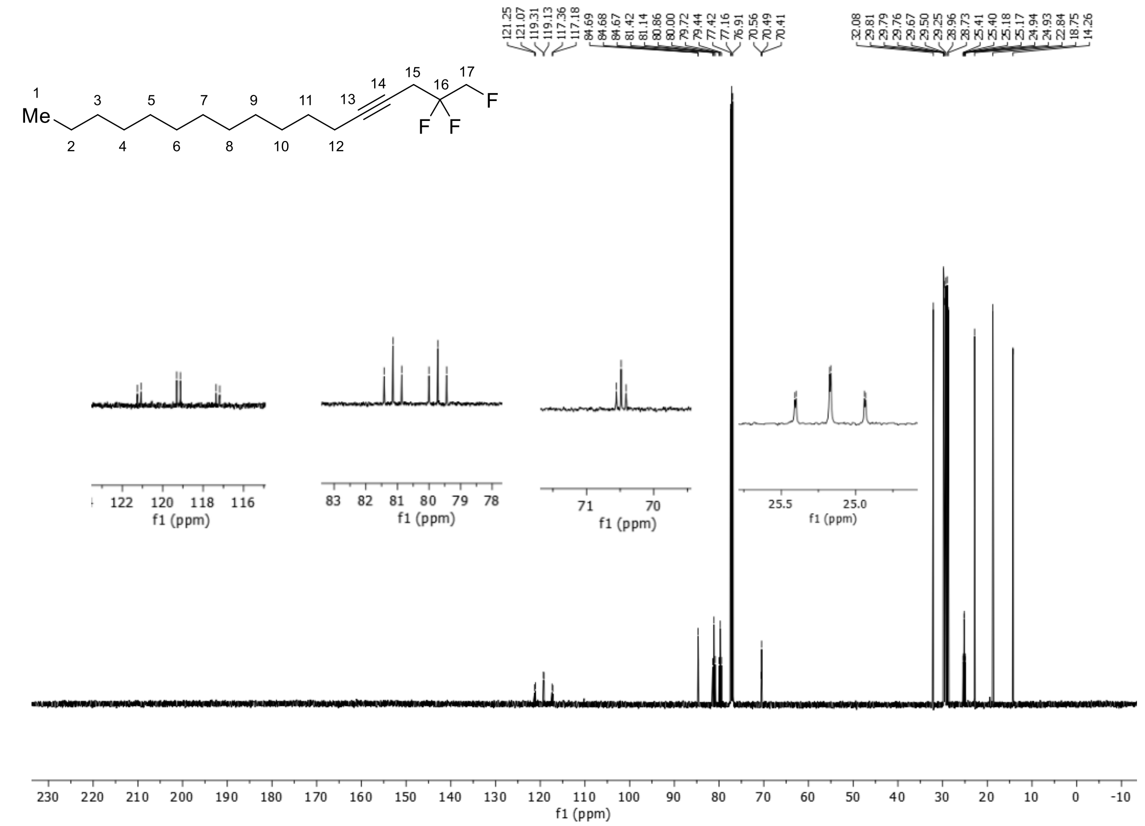

Supplementary Figure 204. $^{13}C\{^{1}H\}$ NMR of **31** (126 MHz, 299 K, $CDCl_3$).

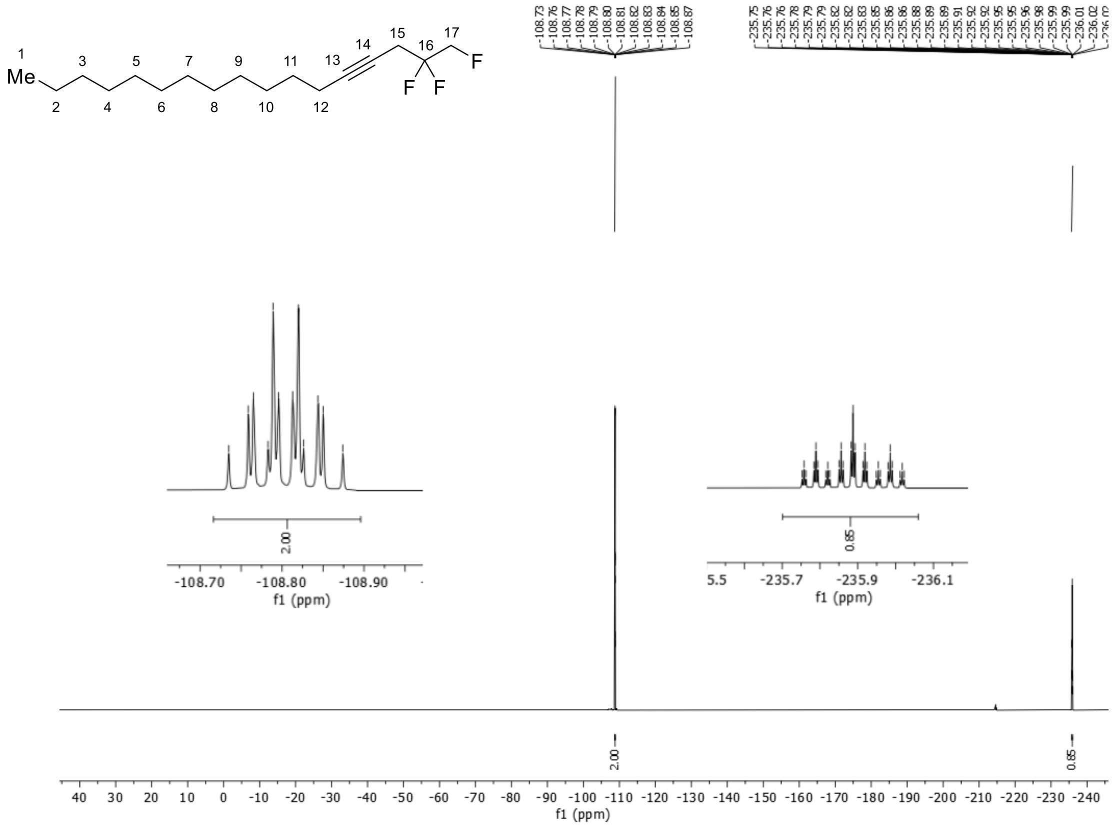

Supplementary Figure 205. ^{19}F NMR of **31** (470 MHz, 299 K, $CDCl_3$).

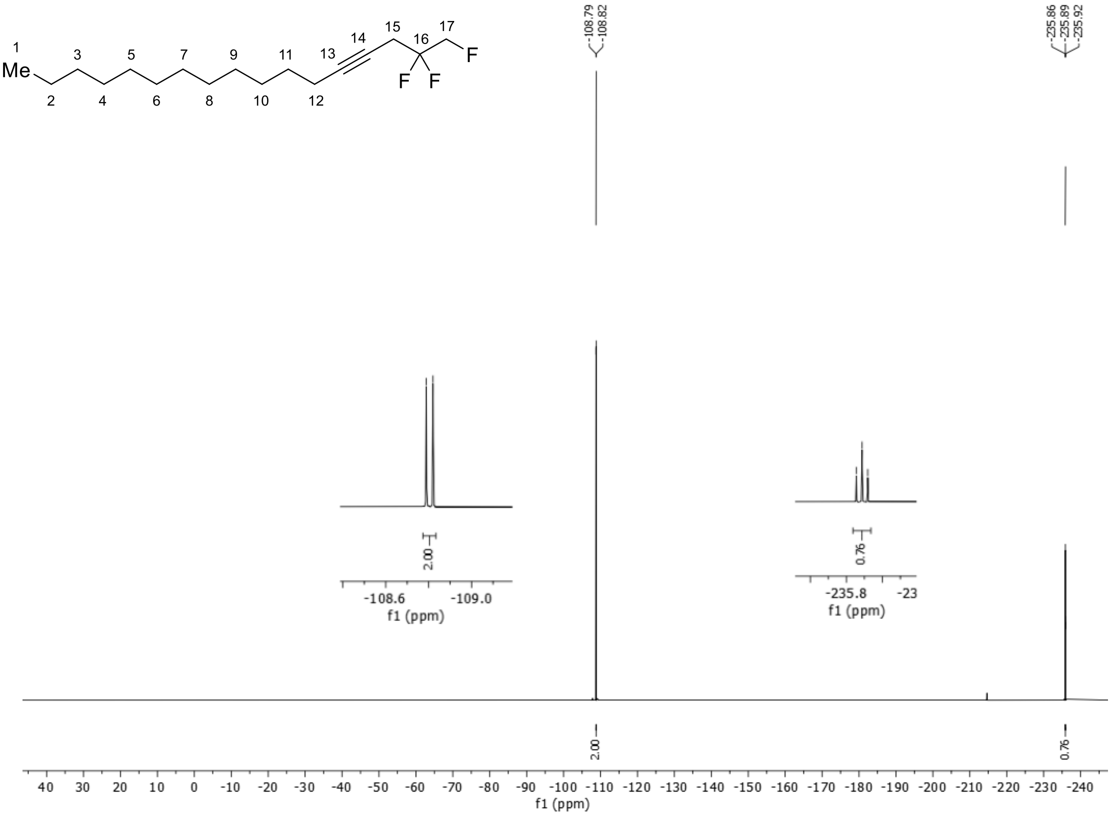

Supplementary Figure 206. $^{19}F\{^1H\}$ NMR of **31** (470 MHz, 299 K, $CDCl_3$).

14,14-Difluoropentadec-11-yn-1-yl-2-(3-cyano-4-isobutoxyphenyl)-4-methylthiazole-5-carboxylate (32)

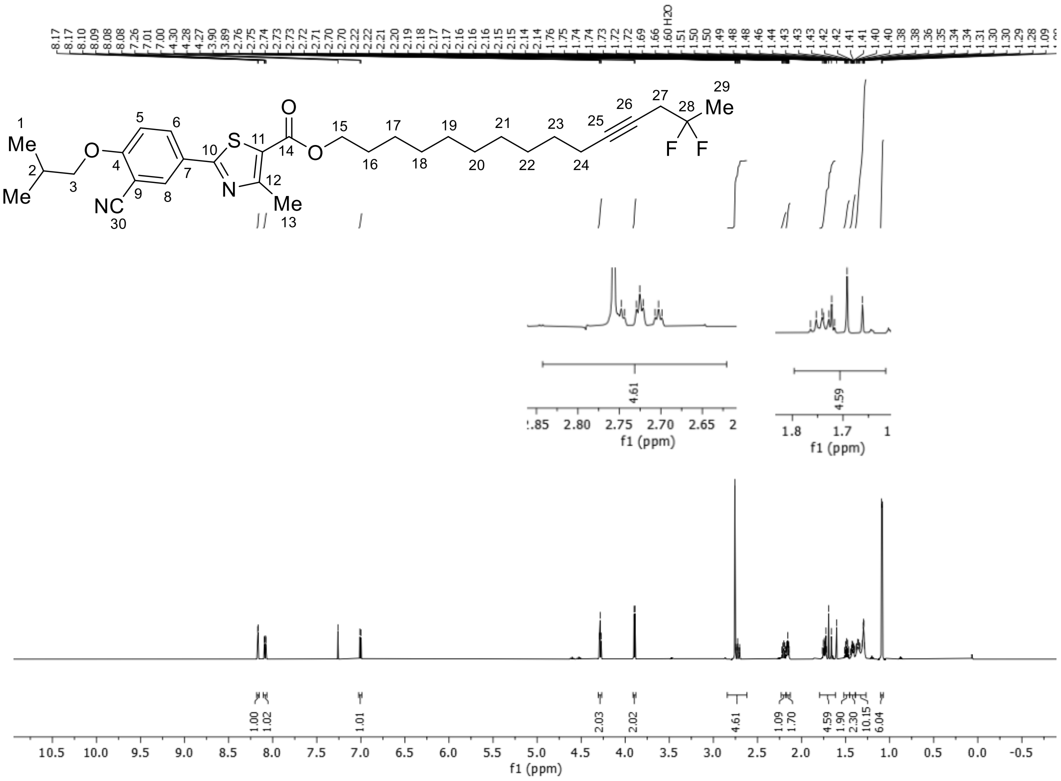

Supplementary Figure 207. ^{1}H NMR of **32** (599 MHz, 299 K, $CDCl_3$).

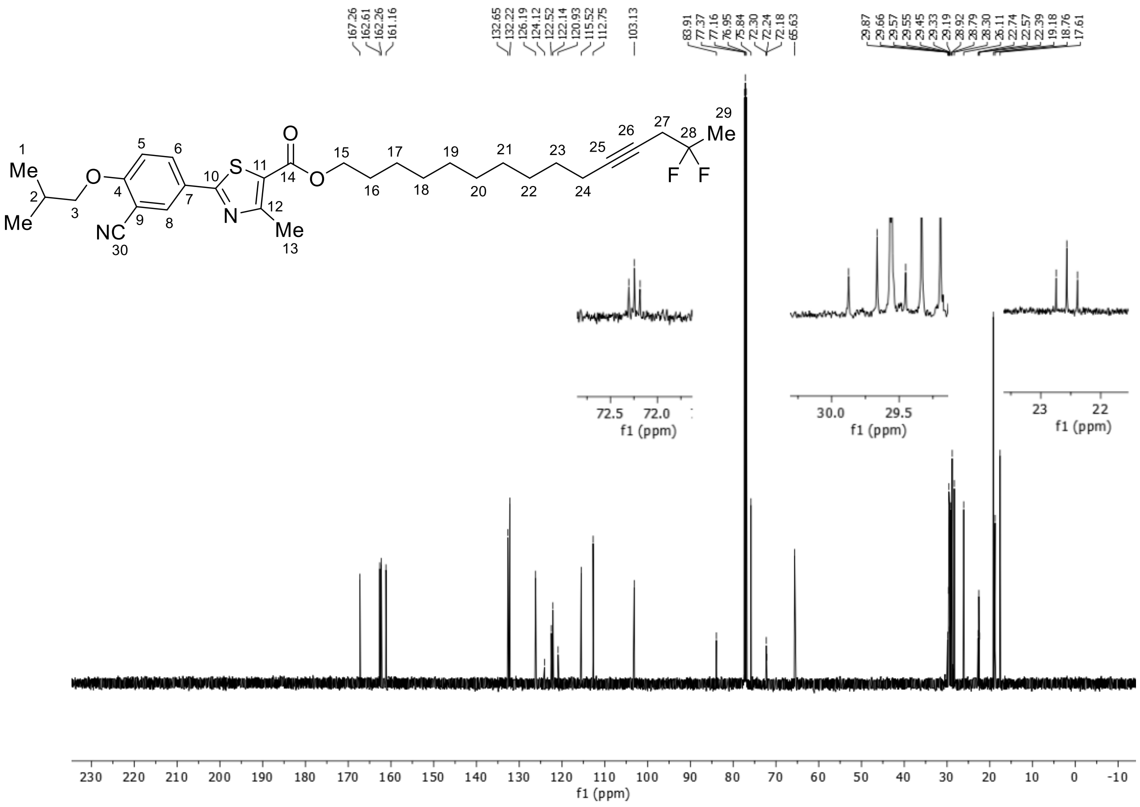

Supplementary Figure 208. $^{13}C\{^{1}H\}$ NMR of **32** (151 MHz, 299 K, $CDCl_3$).

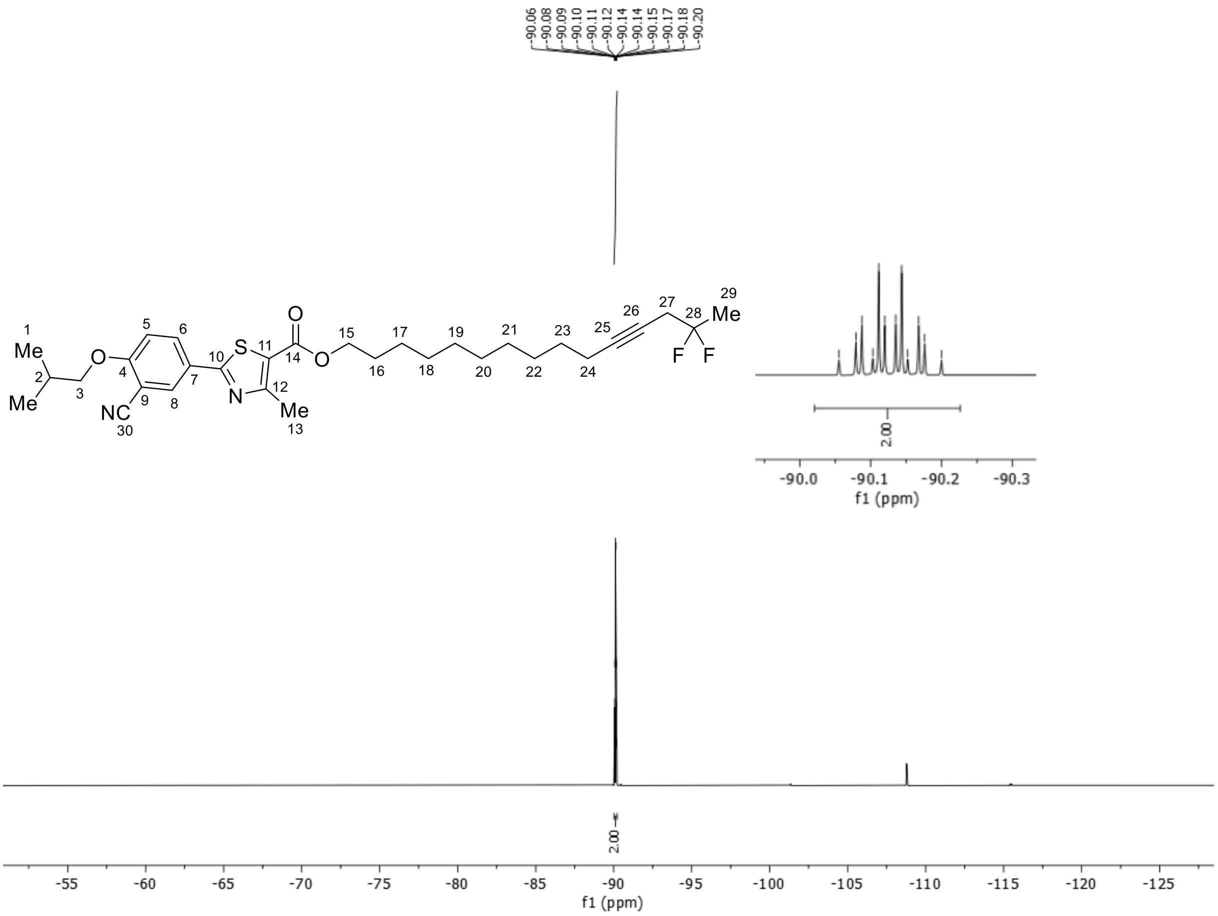

Supplementary Figure 209. ^{19}F NMR of **32** (564 MHz, 299 K, $CDCl_3$).

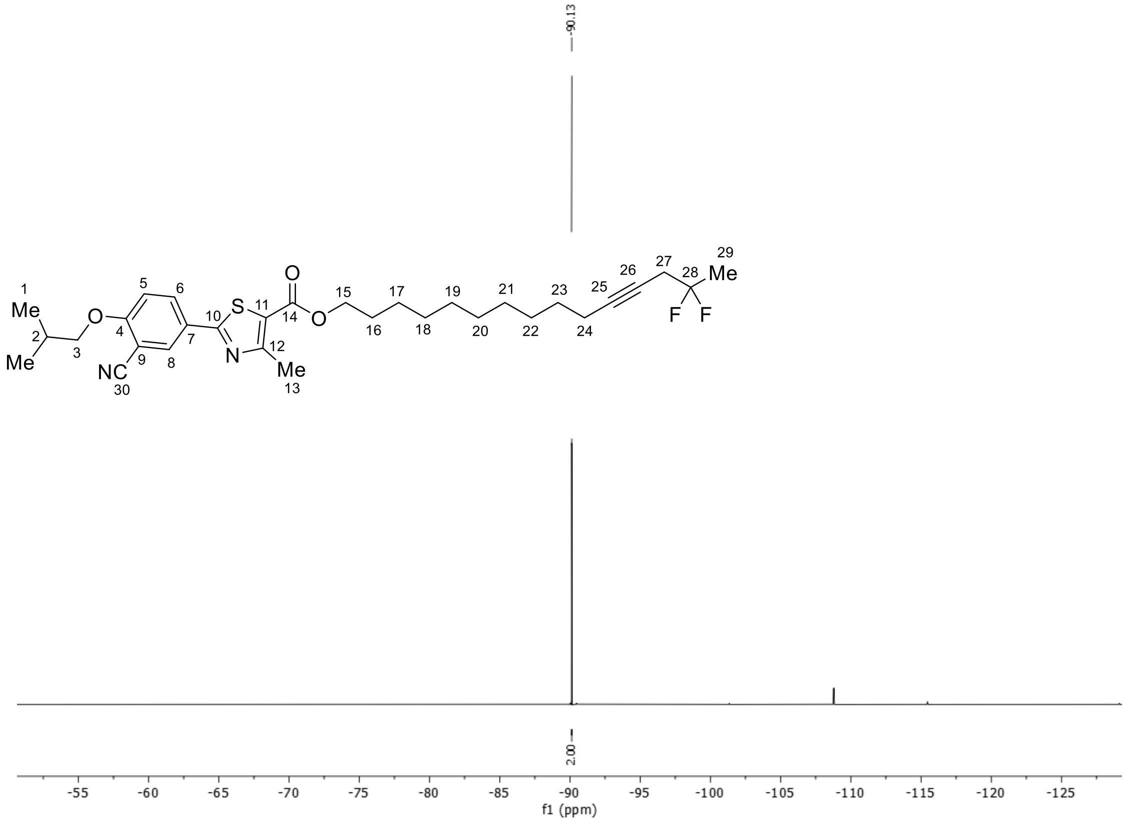

Supplementary Figure 210. ^{19}F{^{1}H} NMR of **32** (564 MHz, 299 K, $CDCl_3$).

(4,4-Difluorodec-1-yn-1-yl)triisopropylsilane (33)

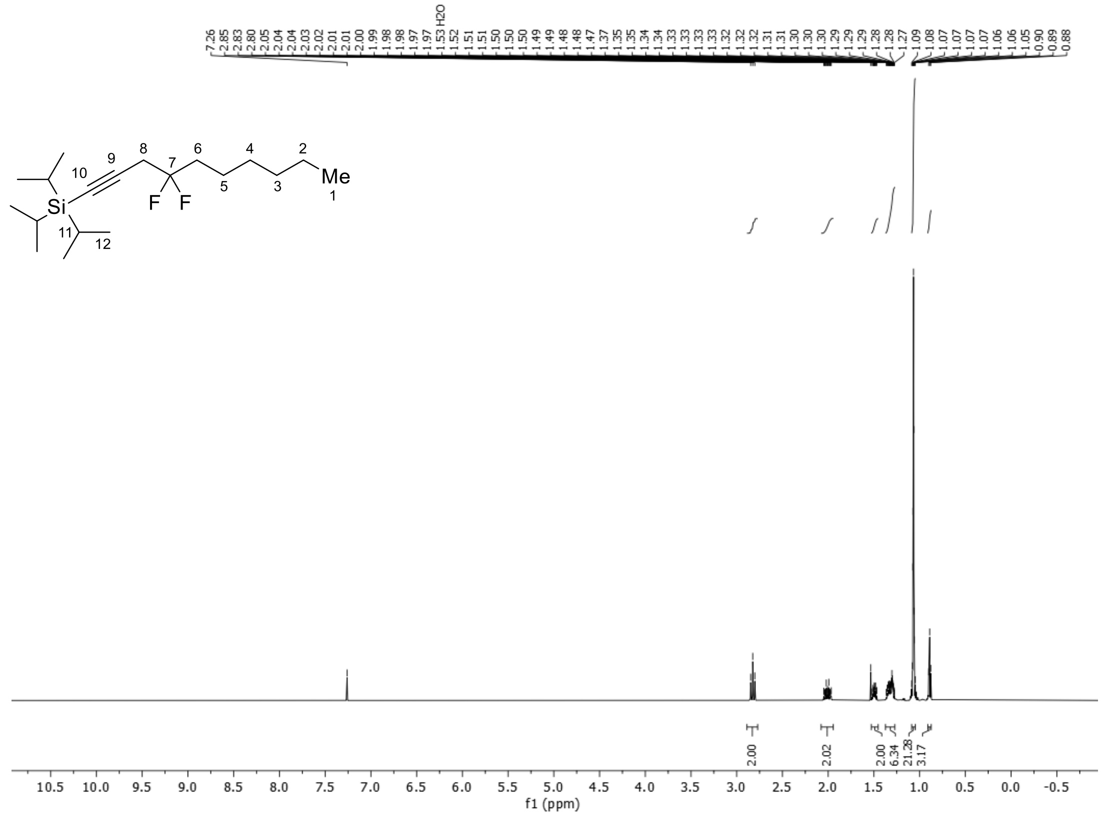

Supplementary Figure 211. ^{1}H NMR of **33** (500 MHz, 299 K, $CDCl_3$).

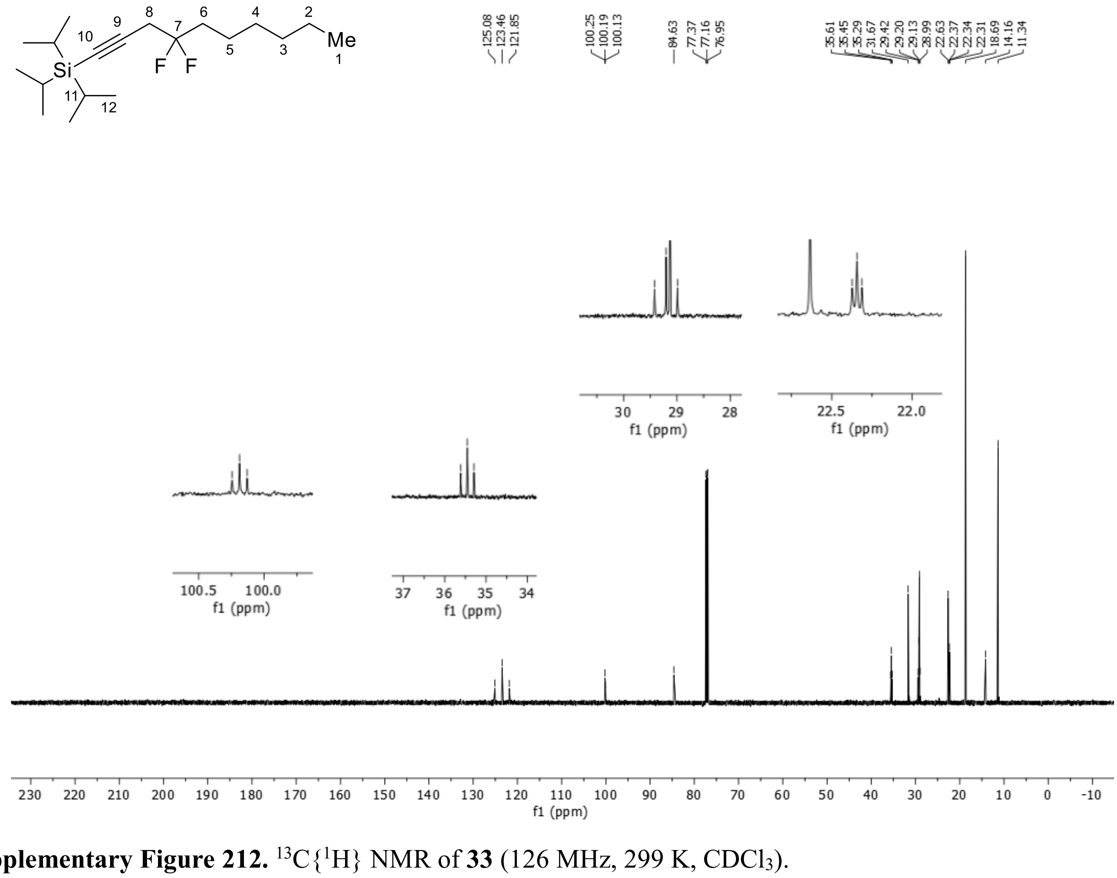

Supplementary Figure 212. $^{13}C\{^{1}H\}$ NMR of **33** (126 MHz, 299 K, $CDCl_3$).

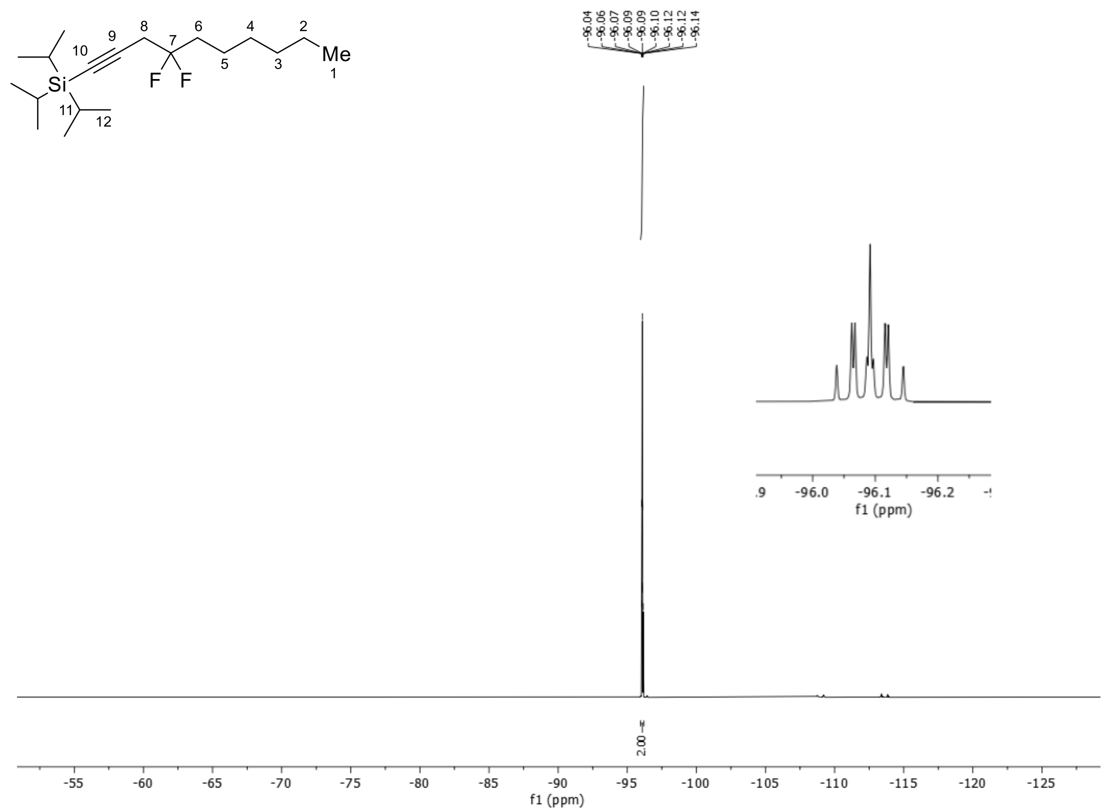

Supplementary Figure 213. ^{19}F NMR of **33** (470 MHz, 299 K, $CDCl_3$).

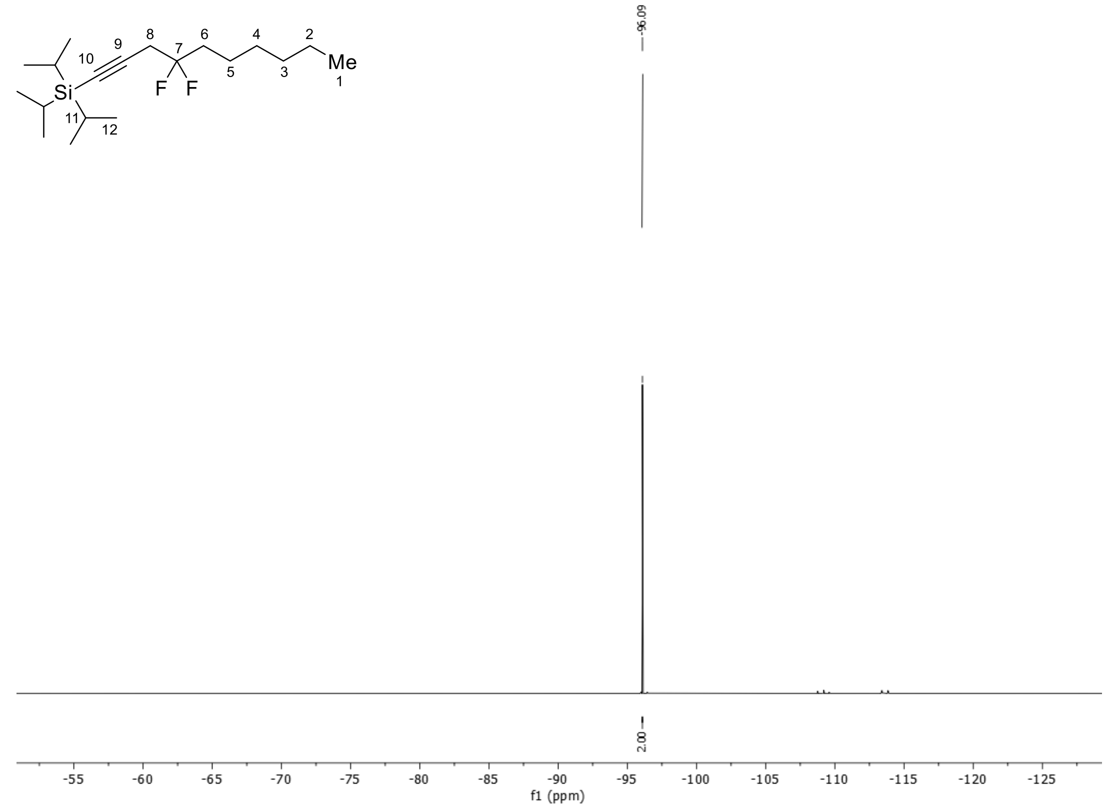

Supplementary Figure 214. $^{19}F\{^1H\}$ NMR of **33** (564 MHz, 299 K, $CDCl_3$).

2,2-Difluoroheptadecane (34)

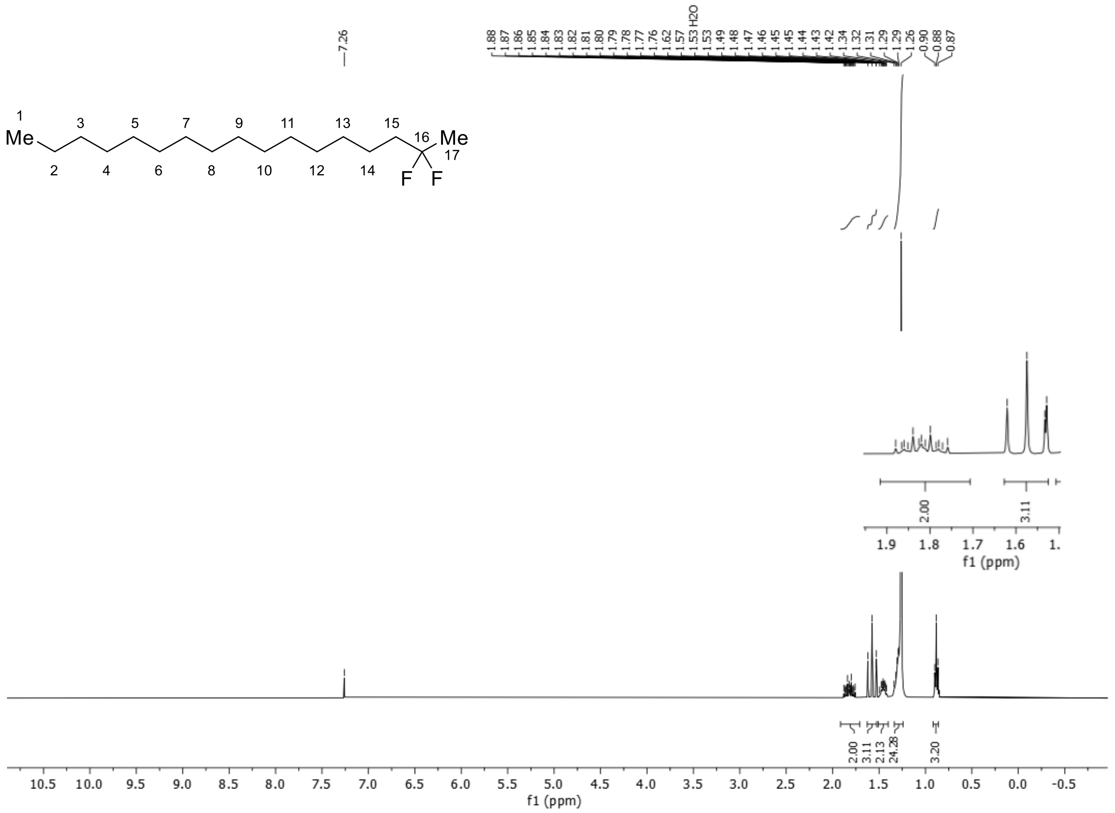

Supplementary Figure 215. ^{1}H NMR of **34** (400 MHz, 299 K, $CDCl_3$).

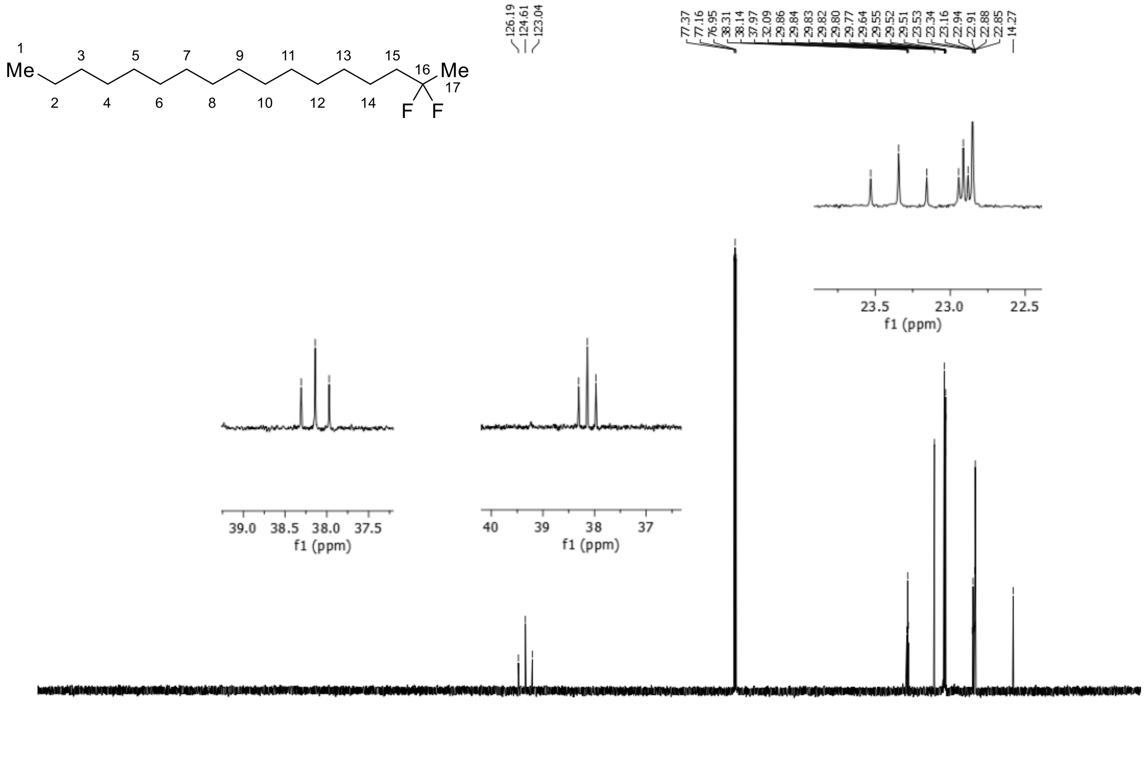

Supplementary Figure 216. $^{13}C\{^{1}H\}$ NMR of **34** (151 MHz, 299 K, $CDCl_3$).

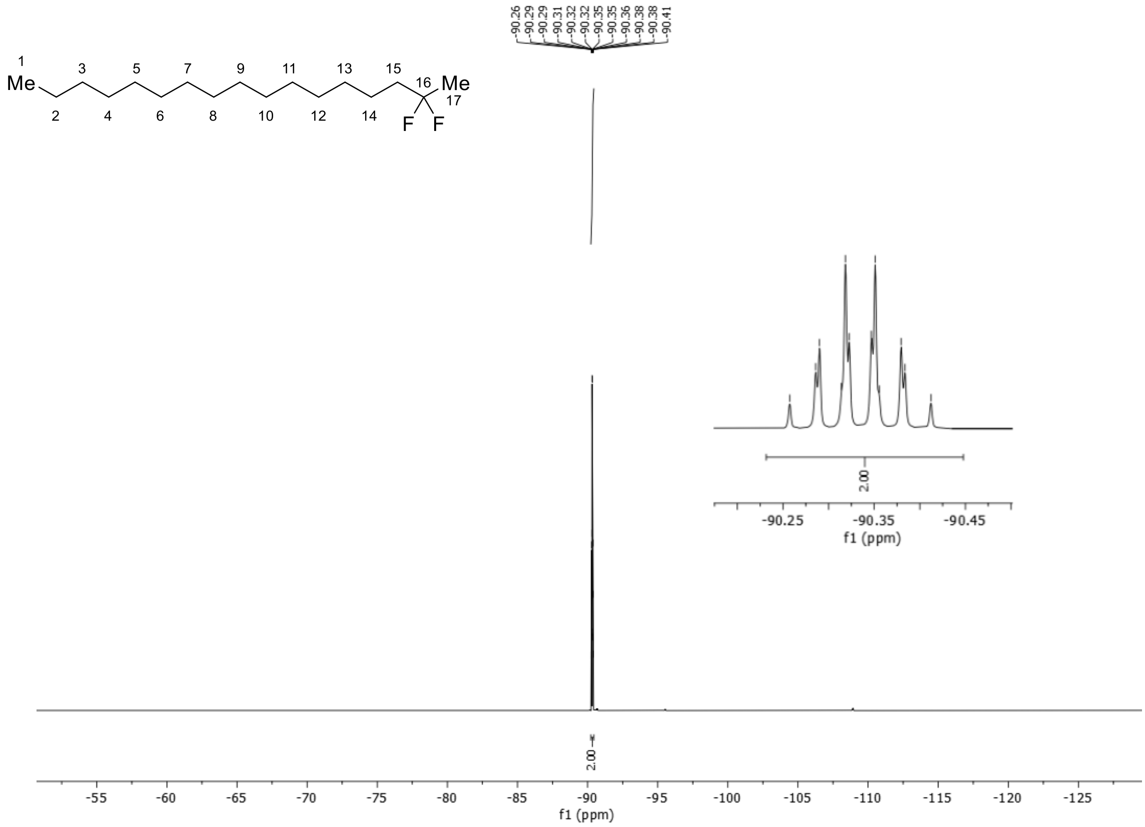

Supplementary Figure 217. ^{19}F NMR of **34** (564 MHz, 299 K, $CDCl_3$).

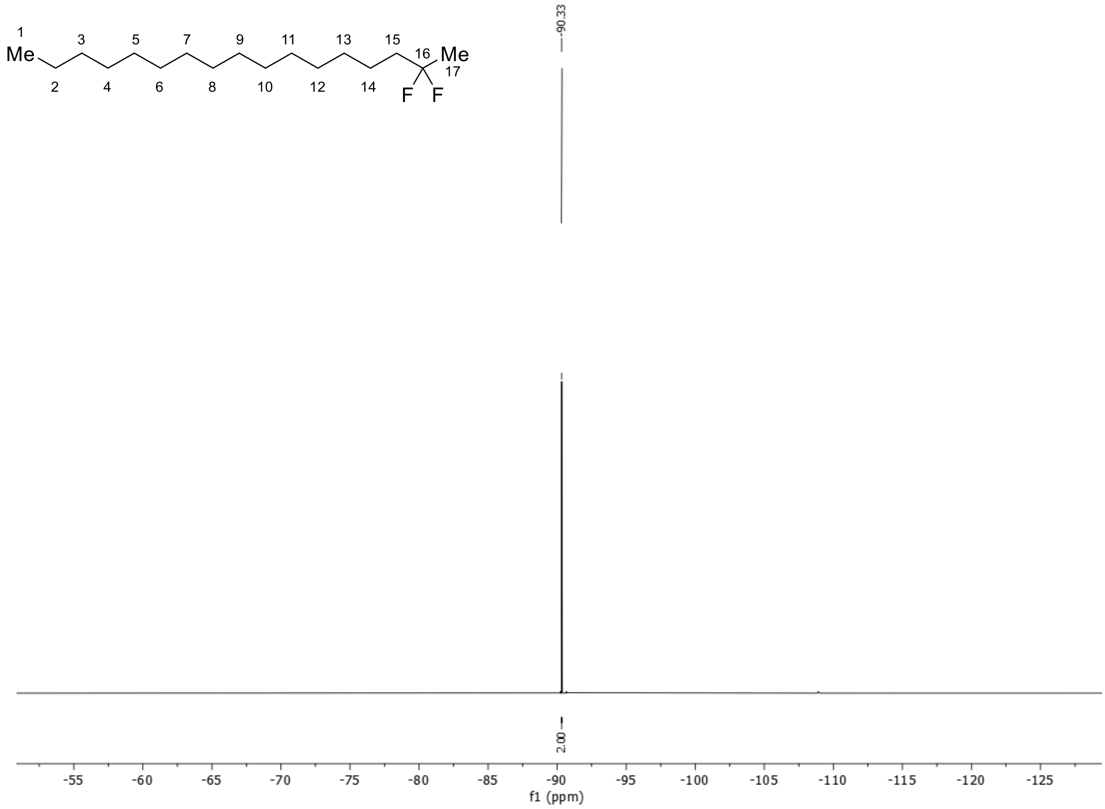

Supplementary Figure 218. ^{19}F{^{1}H} NMR of **34** (564 MHz, 299 K, $CDCl_3$).

(Z)-2,2-Difluoroheptadec-4-ene (35)

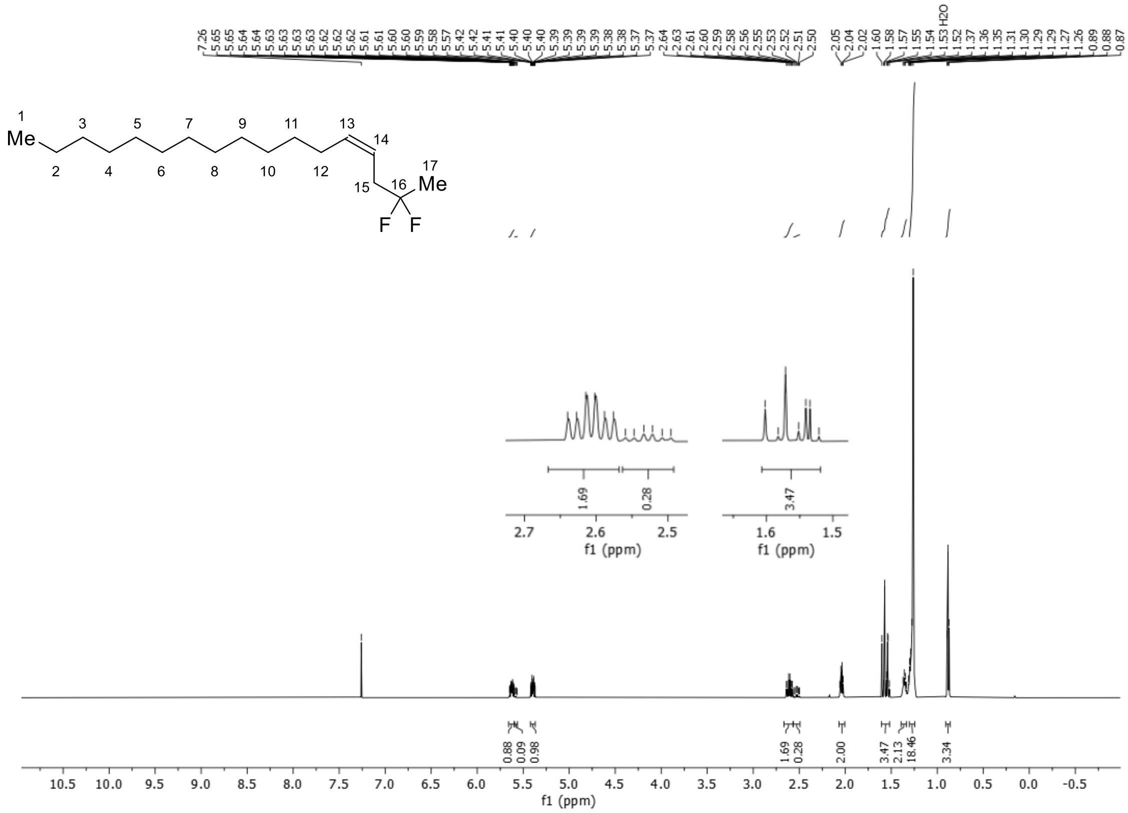

Supplementary Figure 219. ^{1}H NMR of **35** (599 MHz, 299 K, $CDCl_3$).

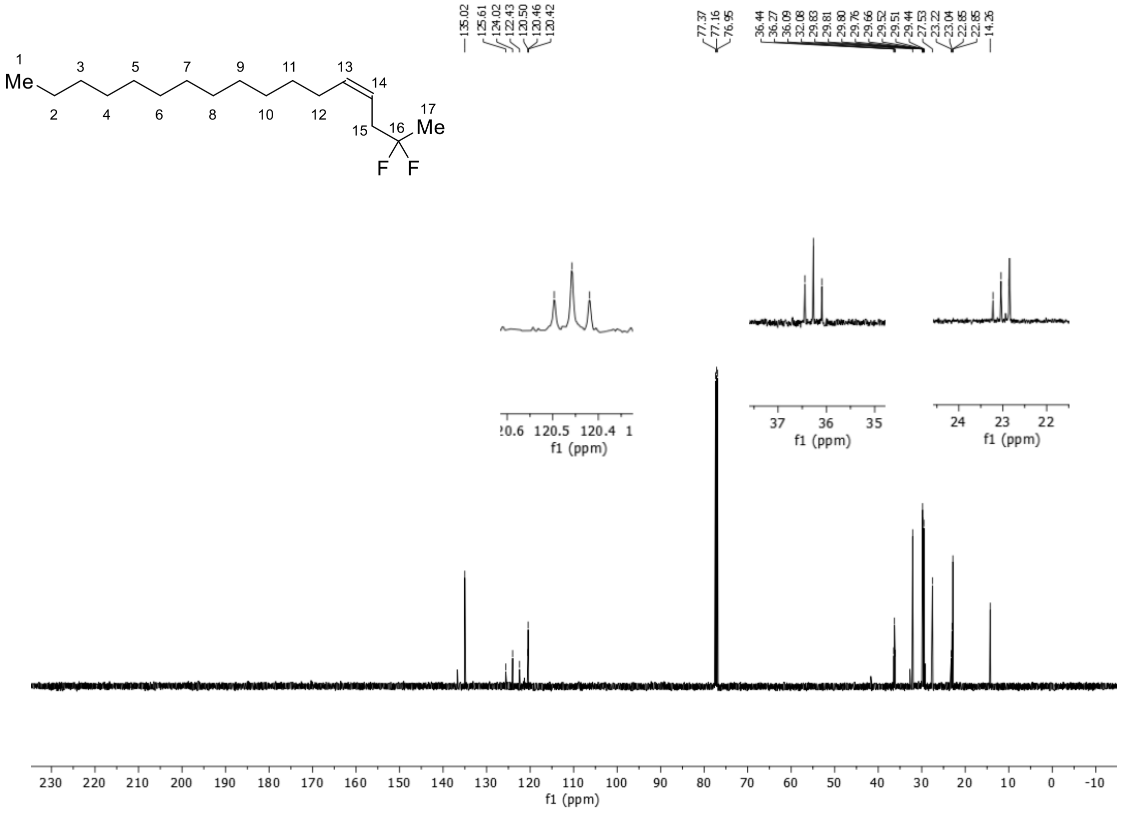

Supplementary Figure 220. $^{13}C\{^{1}H\}$ NMR of **35** (151 MHz, 299 K, $CDCl_3$).

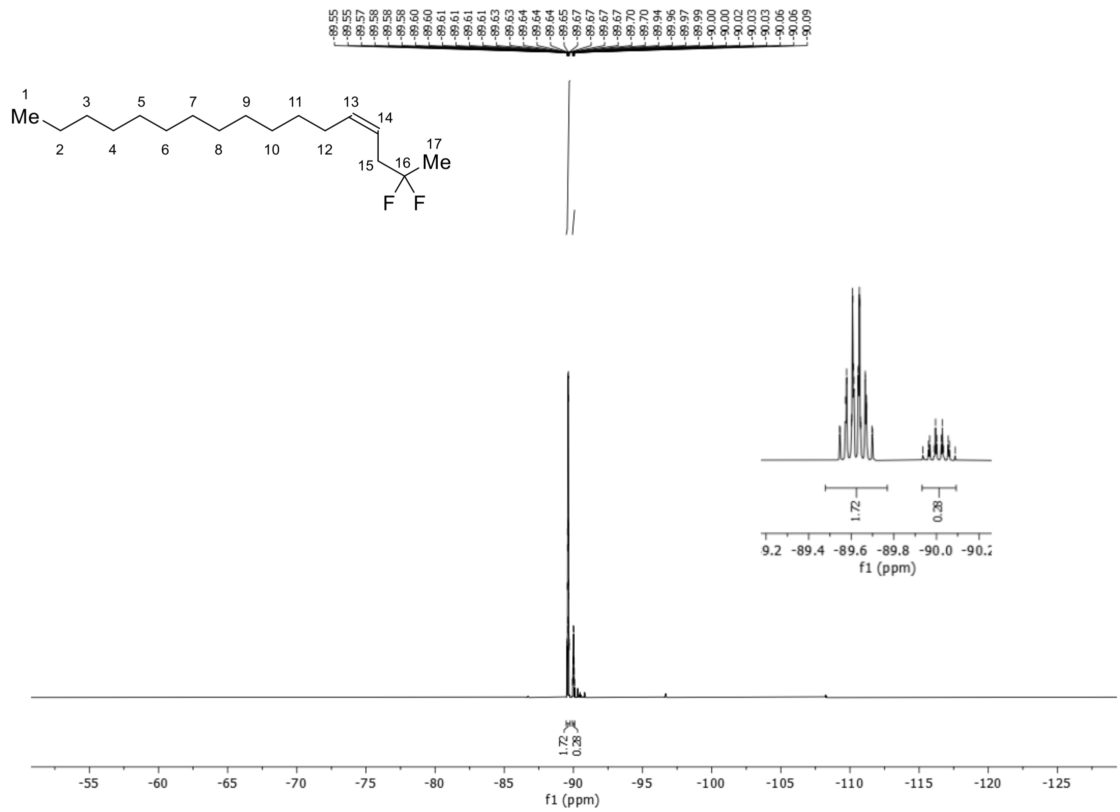

Supplementary Figure 221. ^{19}F NMR of **35** (564 MHz, 299 K, $CDCl_3$).

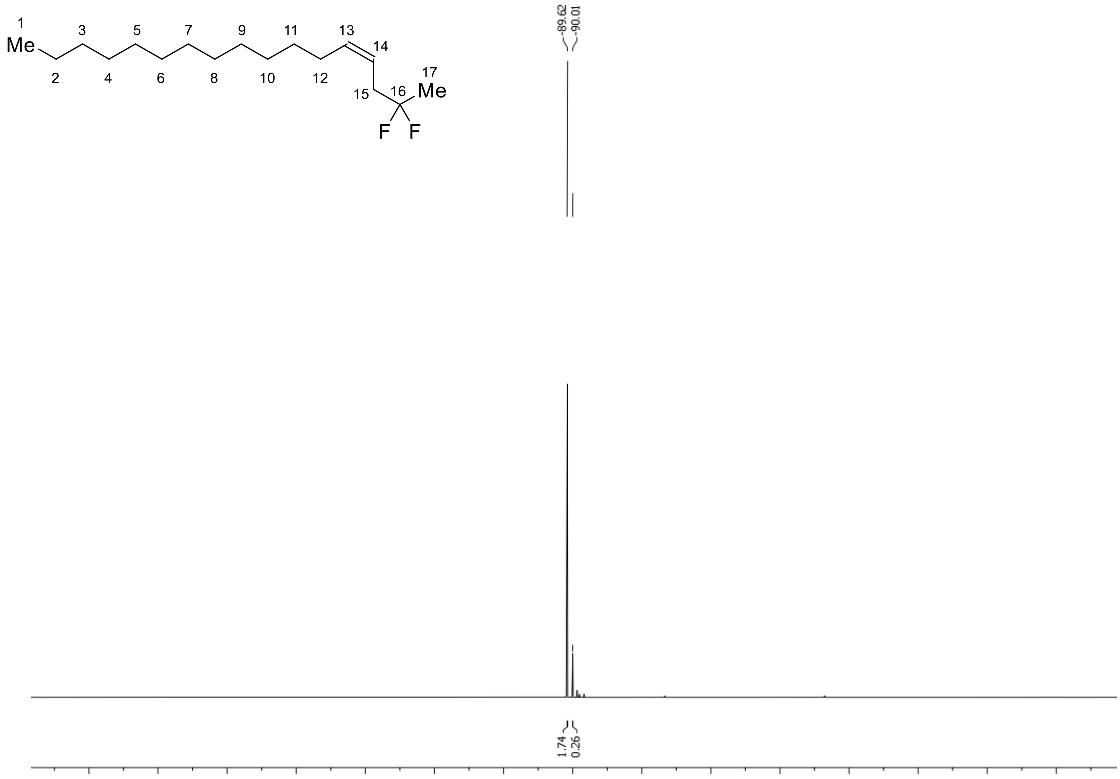

Supplementary Figure 222. $^{19}F\{^1H\}$ NMR of **35** (564 MHz, 299 K, $CDCl_3$).

2-(4-Chlorophenyl)-3-(2,2-difluoropropyl)quinoxaline (36)

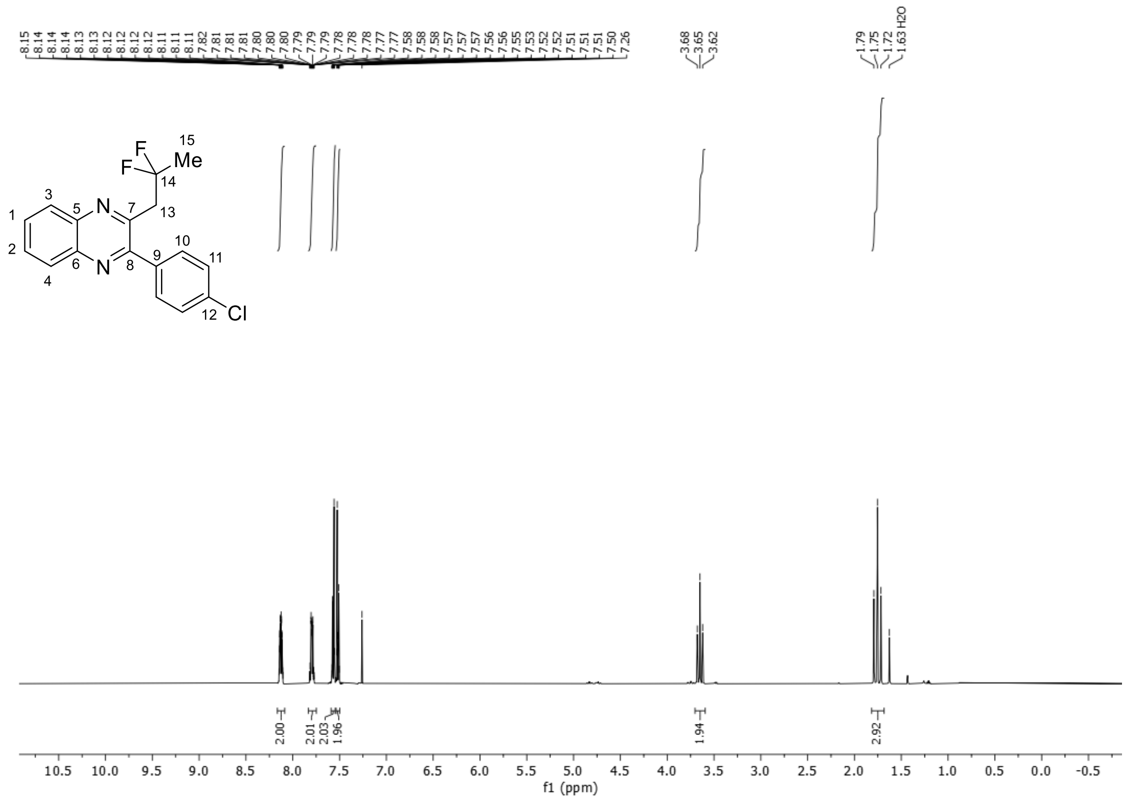

Supplementary Figure 223. ^{1}H NMR of **36** (500 MHz, 299 K, $CDCl_3$).

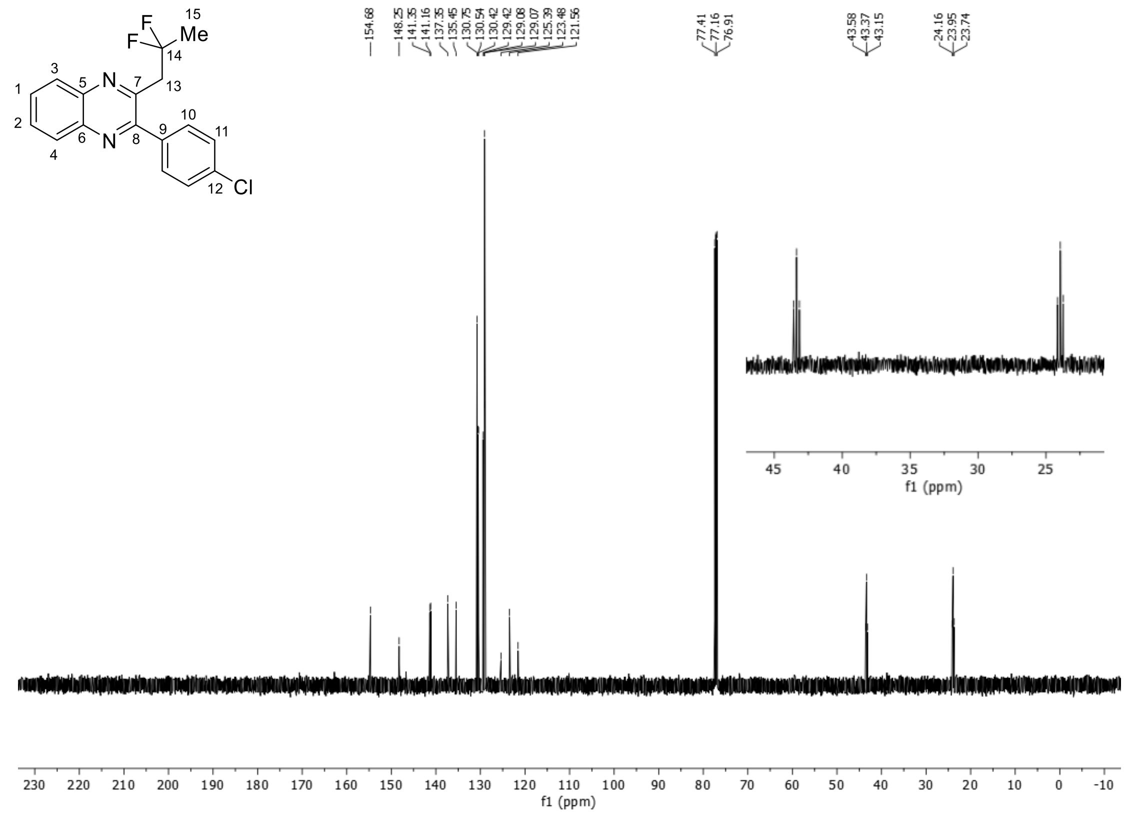

Supplementary Figure 224. $^{13}C\{^{1}H\}$ NMR of **36** (126 MHz, 299 K, $CDCl_3$).

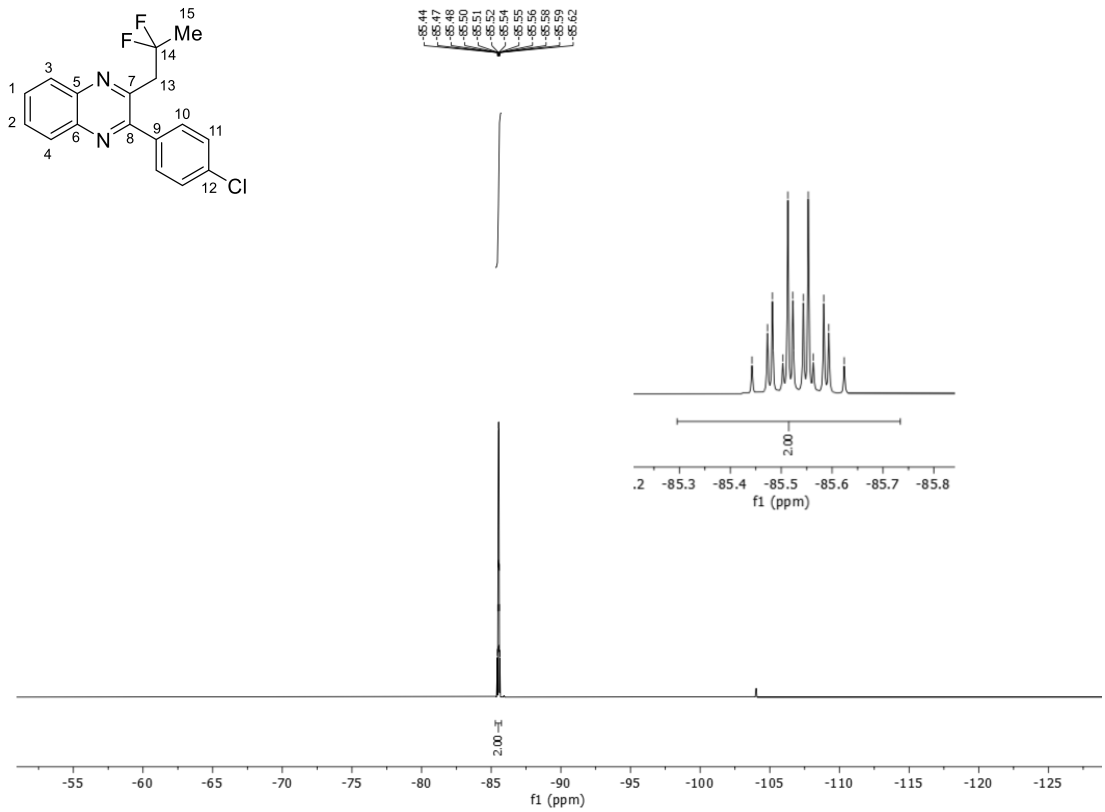

Supplementary Figure 225. ^{19}F NMR of **36** (470 MHz, 299 K, $CDCl_3$).

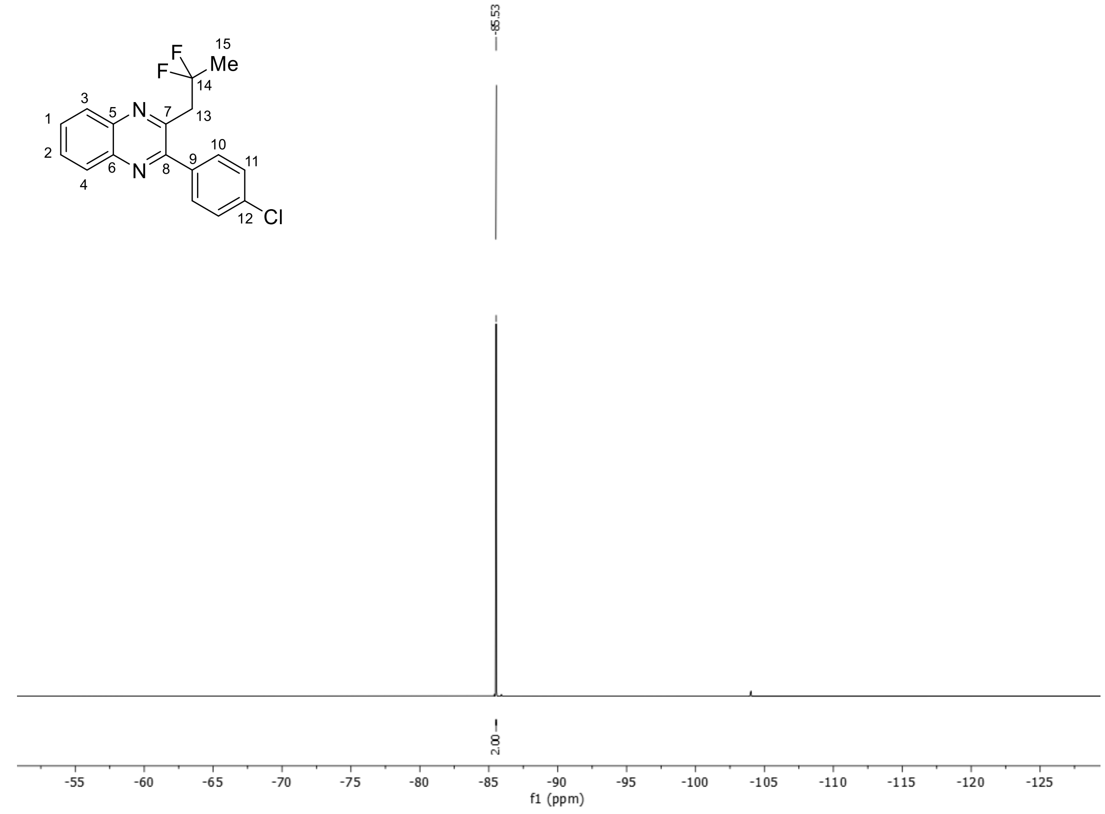

Supplementary Figure 226. $^{19}F\{^1H\}$ NMR of **36** (470 MHz, 299 K, $CDCl_3$).

4,4-Difluorodec-1-yne (37)

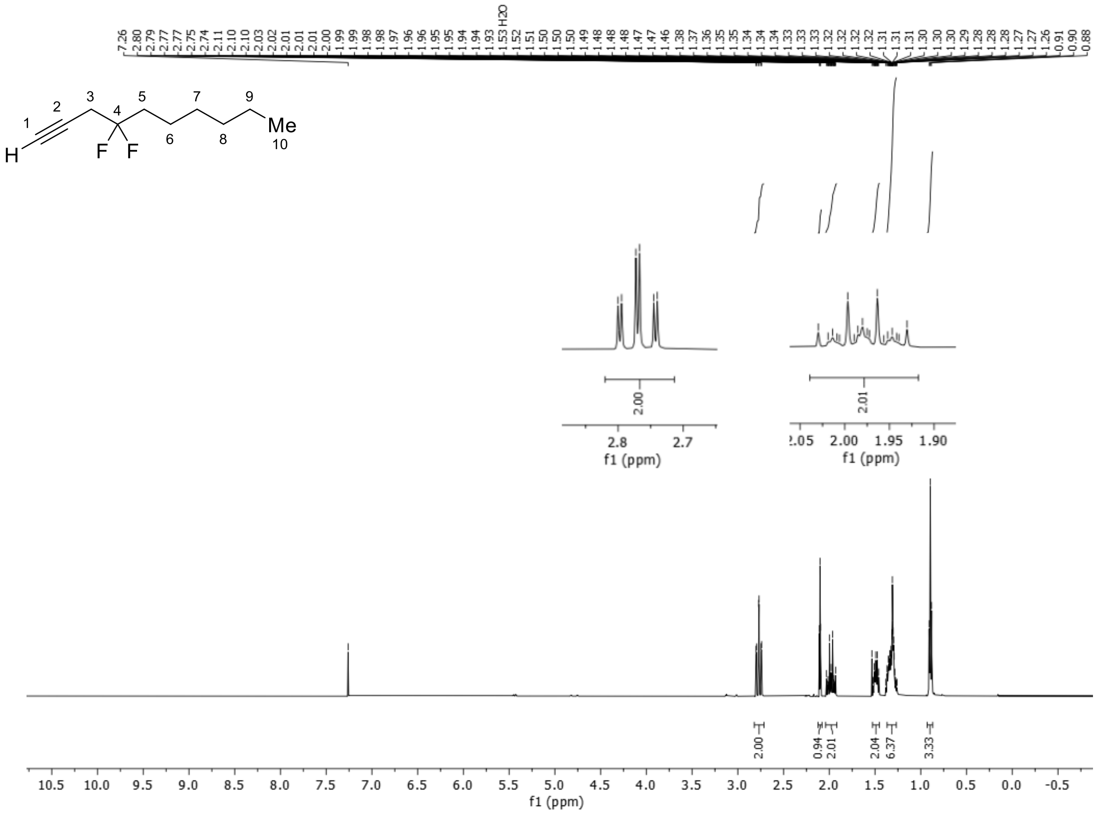

Supplementary Figure 227. ^{1}H NMR of **37** (500 MHz, 299 K, $CDCl_3$).

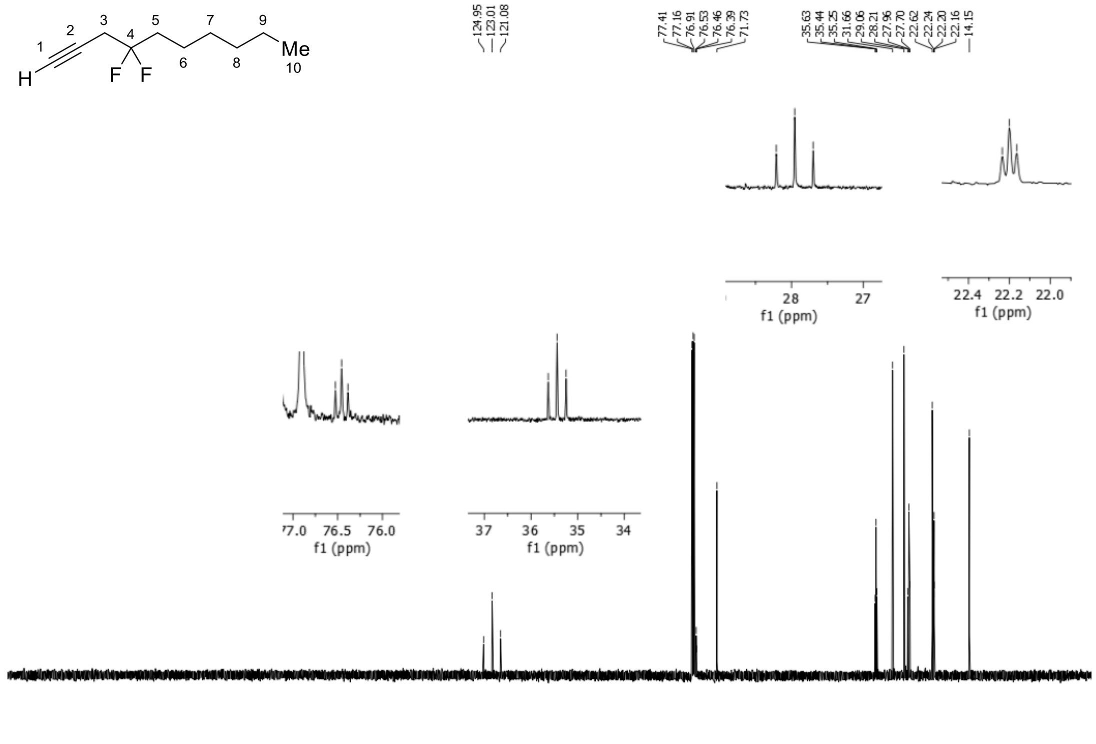

Supplementary Figure 228. $^{13}C\{^{1}H\}$ NMR of **37** (126 MHz, 299 K, $CDCl_3$).

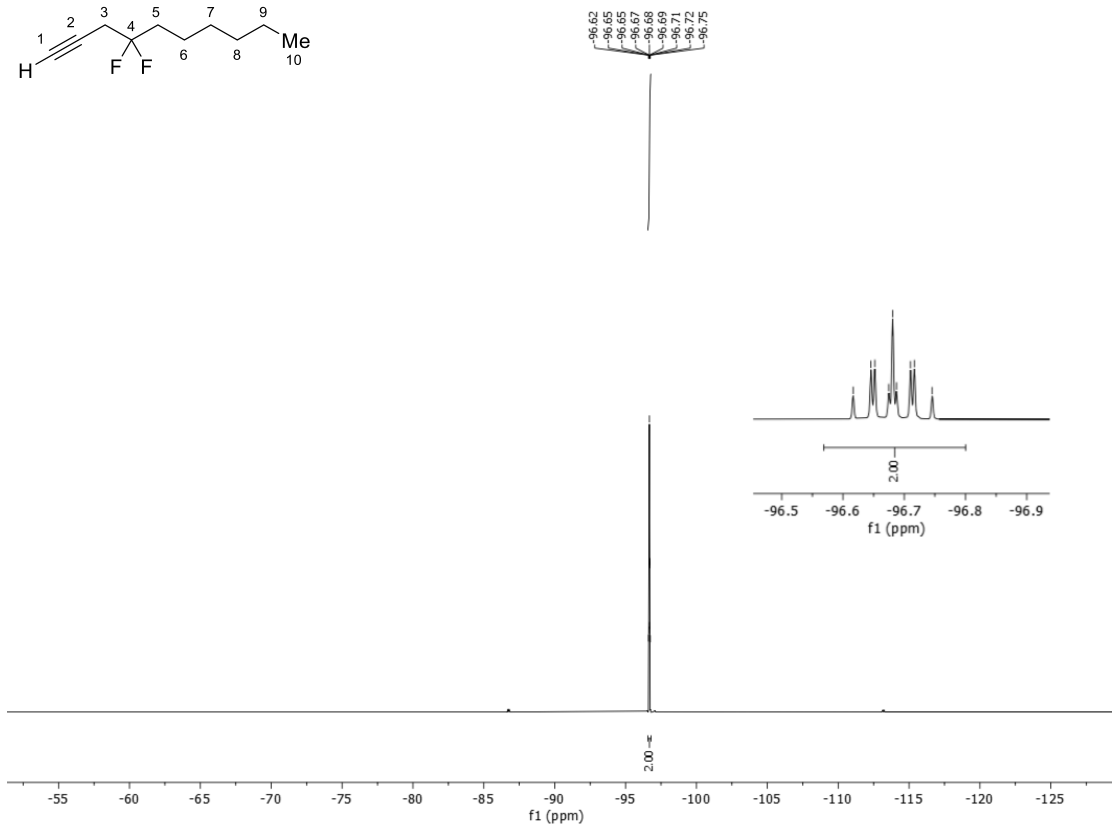

Supplementary Figure 229. ^{19}F NMR of **37** (470 MHz, 299 K, $CDCl_3$).

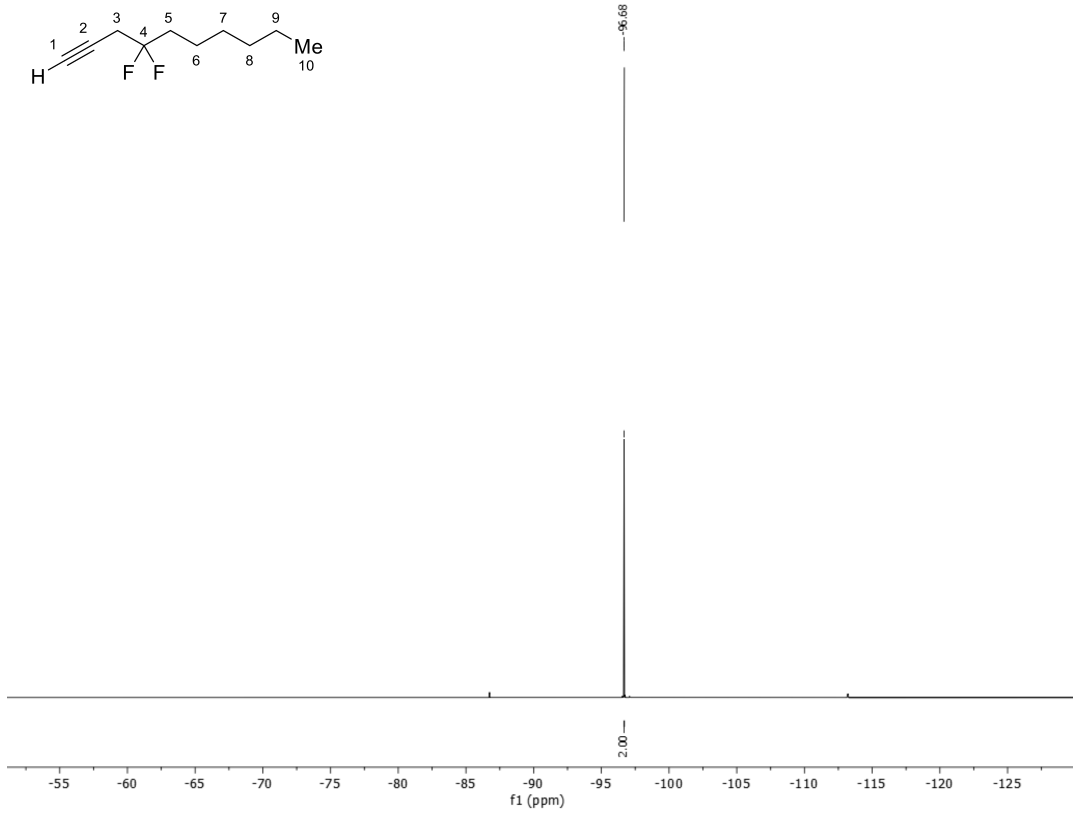

Supplementary Figure 230. $^{19}F\{^1H\}$ NMR of **37** (470 MHz, 299 K, $CDCl_3$).

N-(4-((4-(2,2-difluorooctyl)-1H-1,2,3-triazol-1-yl)sulfonyl)phenyl)acetamide (38)

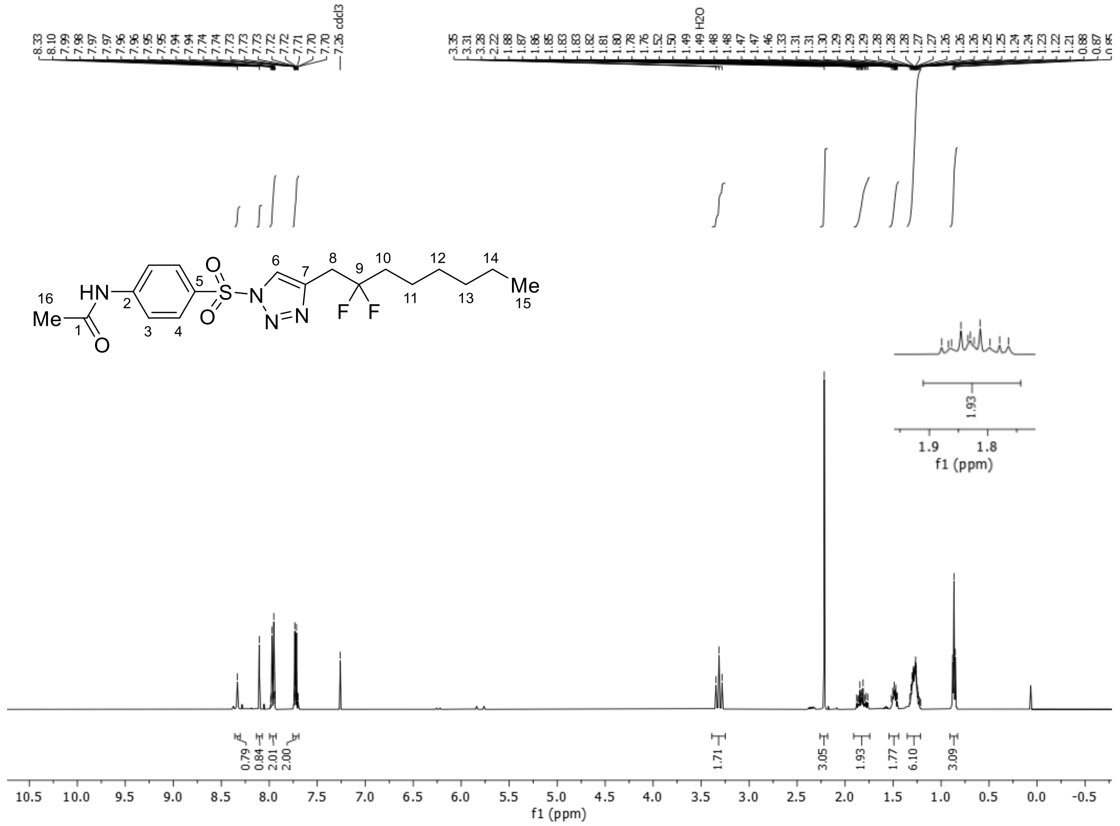

Supplementary Figure 231. ^{1}H NMR of **38** (500 MHz, 299 K, $CDCl_3$).

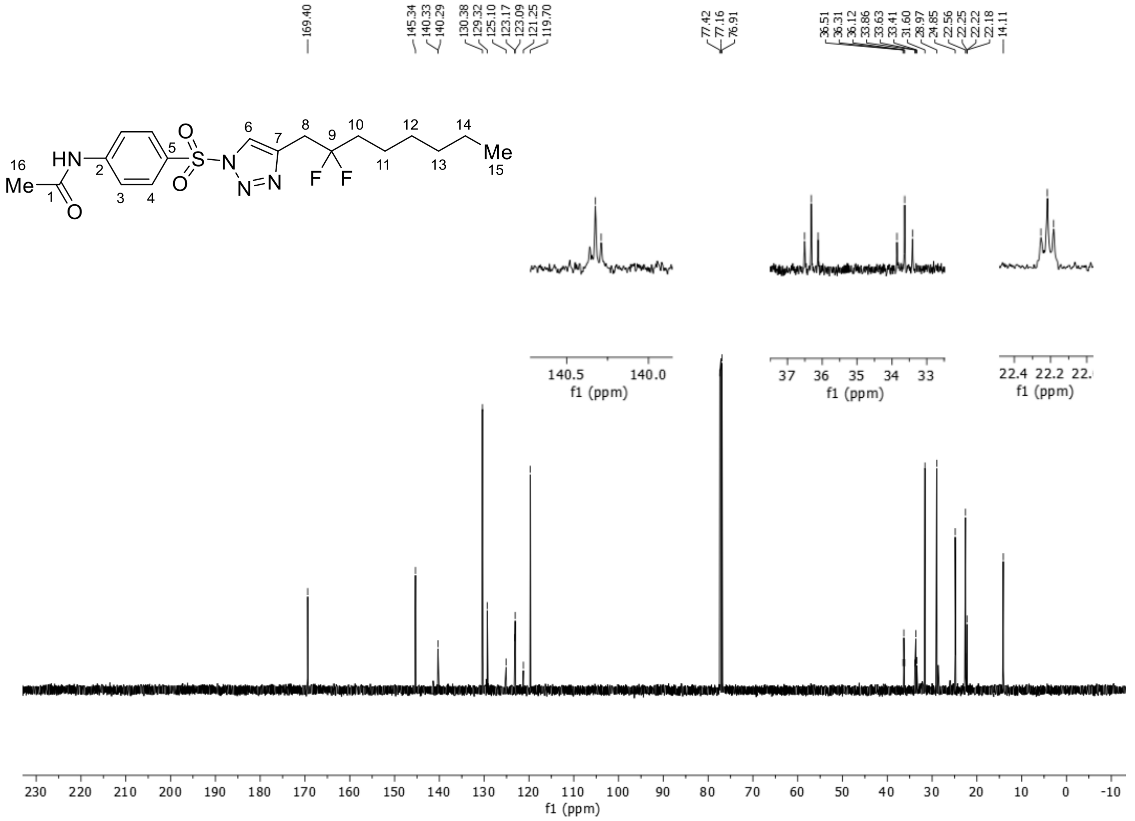

Supplementary Figure 232. $^{13}C\{^{1}H\}$ NMR of **38** (126 MHz, 299 K, $CDCl_3$).

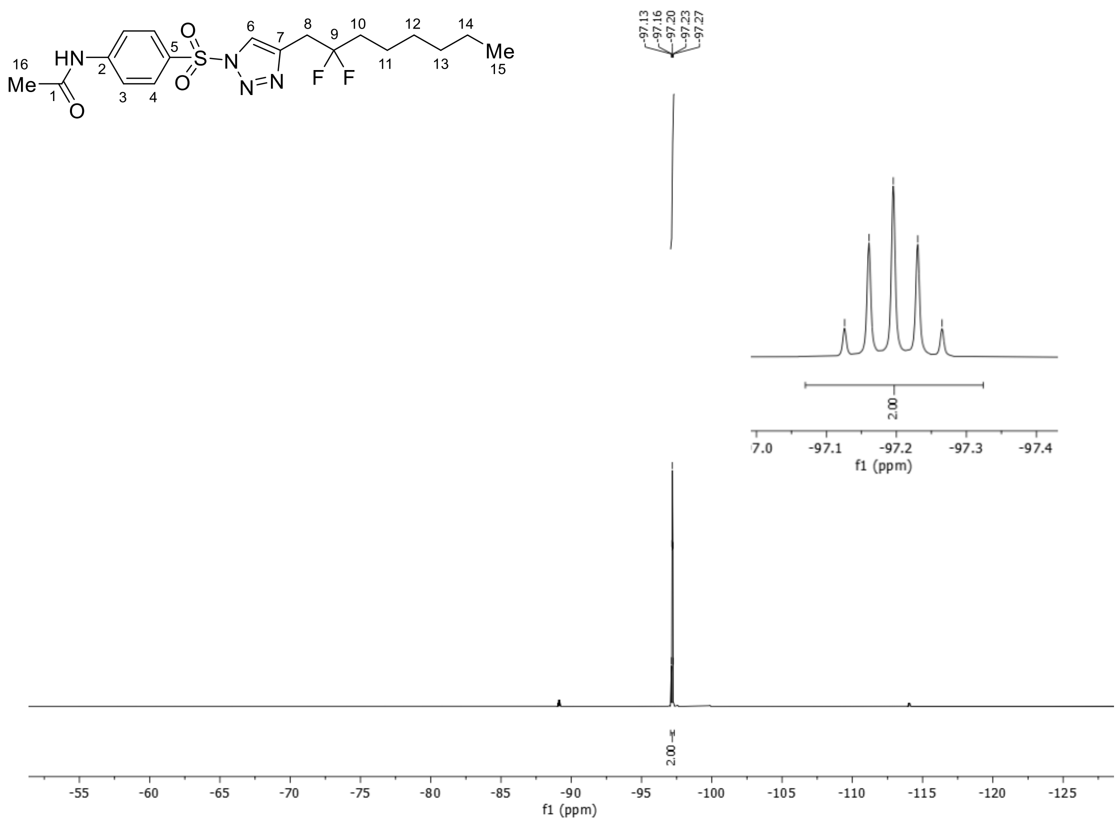

Supplementary Figure 233. ^{19}F NMR of **38** (470 MHz, 299 K, $CDCl_3$).

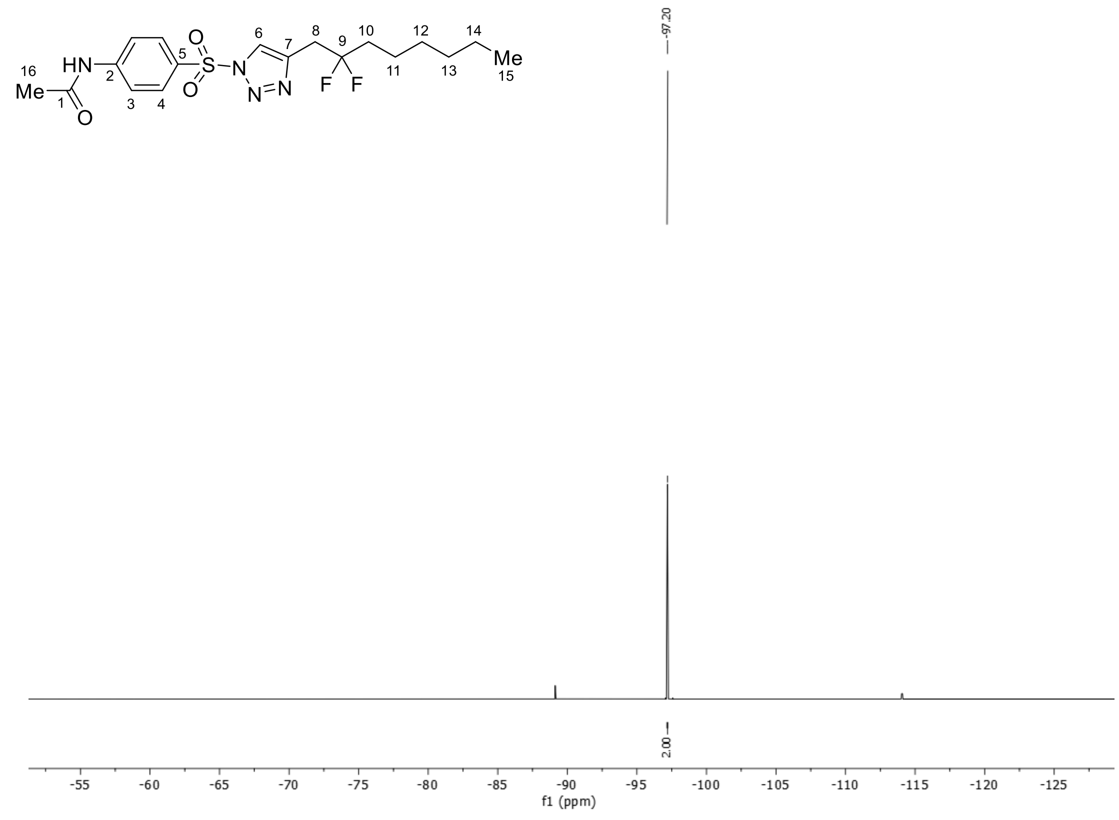

Supplementary Figure 234. $^{19}F\{^1H\}$ NMR of **38** (470 MHz, 299 K, $CDCl_3$).

1-(4,4-Difluorodec-1-yn-1-yl)-4-methoxybenzene (39)

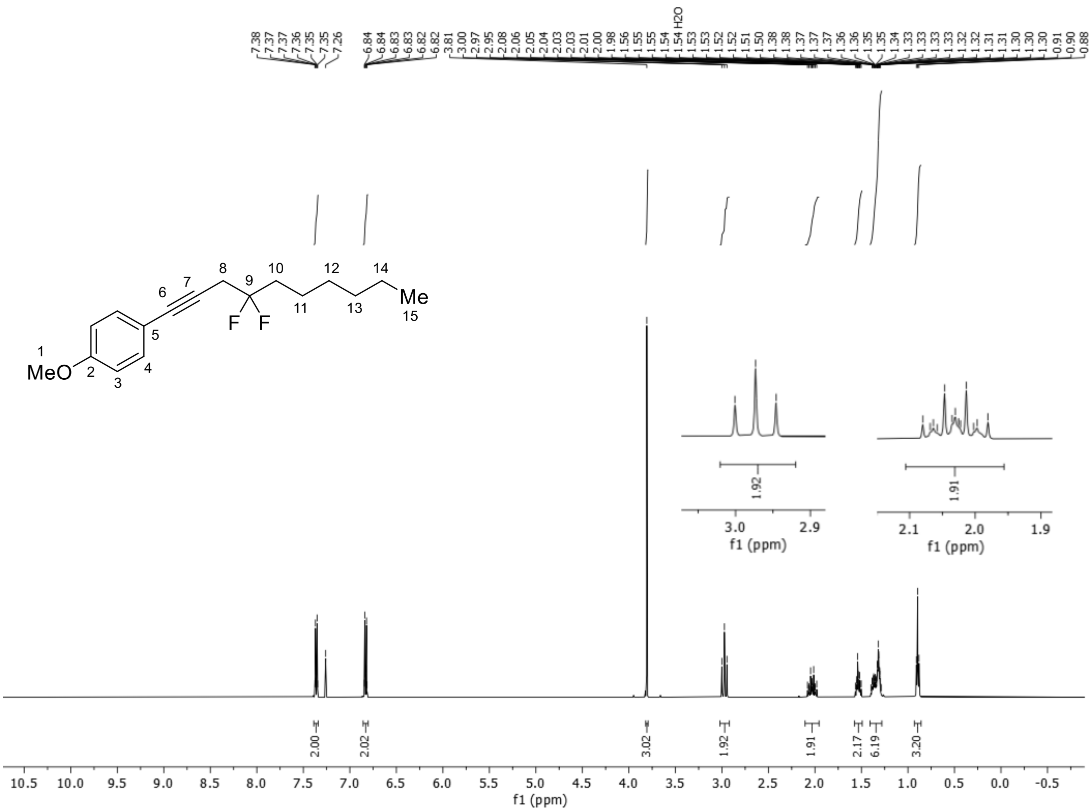

Supplementary Figure 235. ^{1}H NMR of **39** (500 MHz, 299 K, $CDCl_3$).

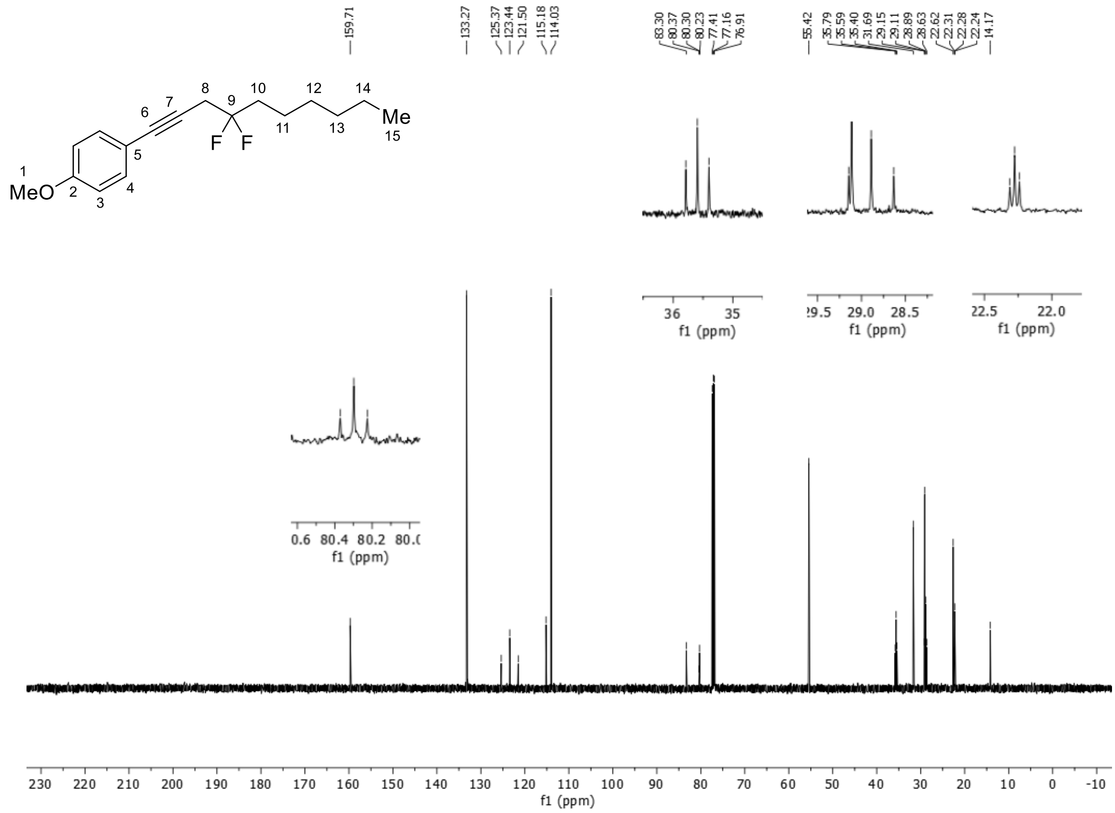

Supplementary Figure 236. $^{13}C\{^{1}H\}$ NMR of **39** (126 MHz, 299 K, $CDCl_3$).

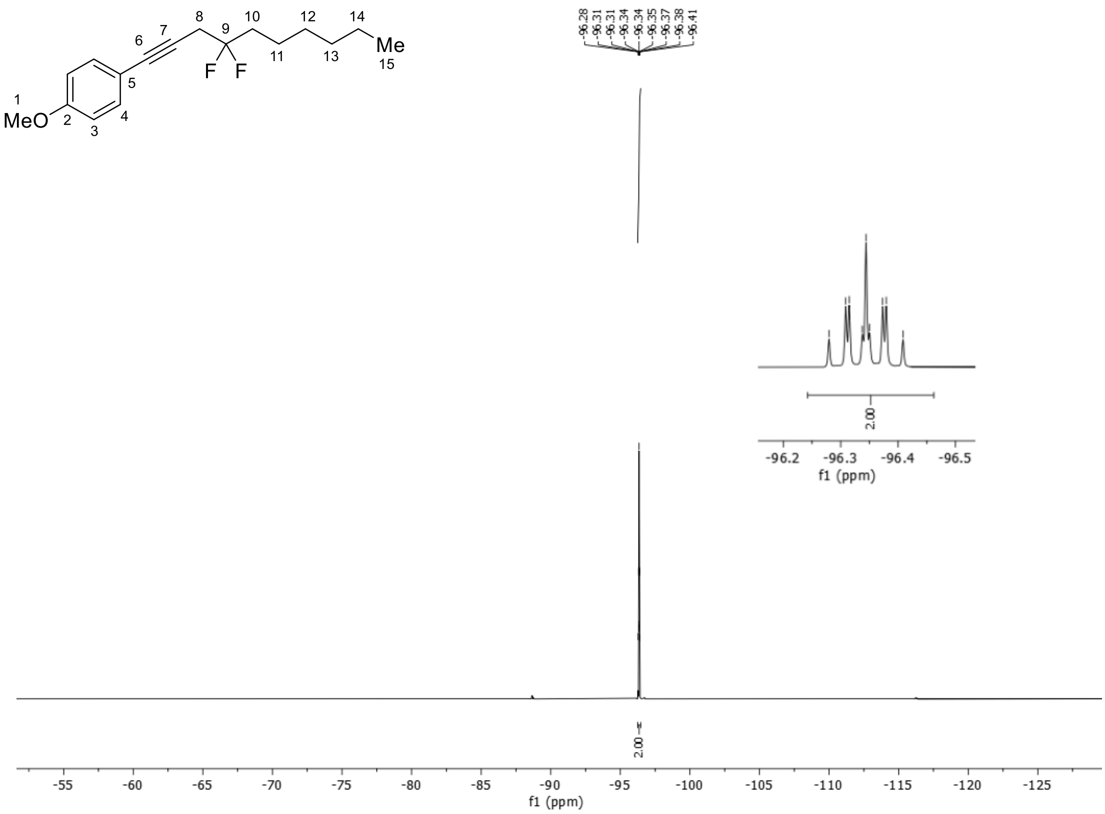

Supplementary Figure 237. ^{19}F NMR of **39** (470 MHz, 299 K, $CDCl_3$).

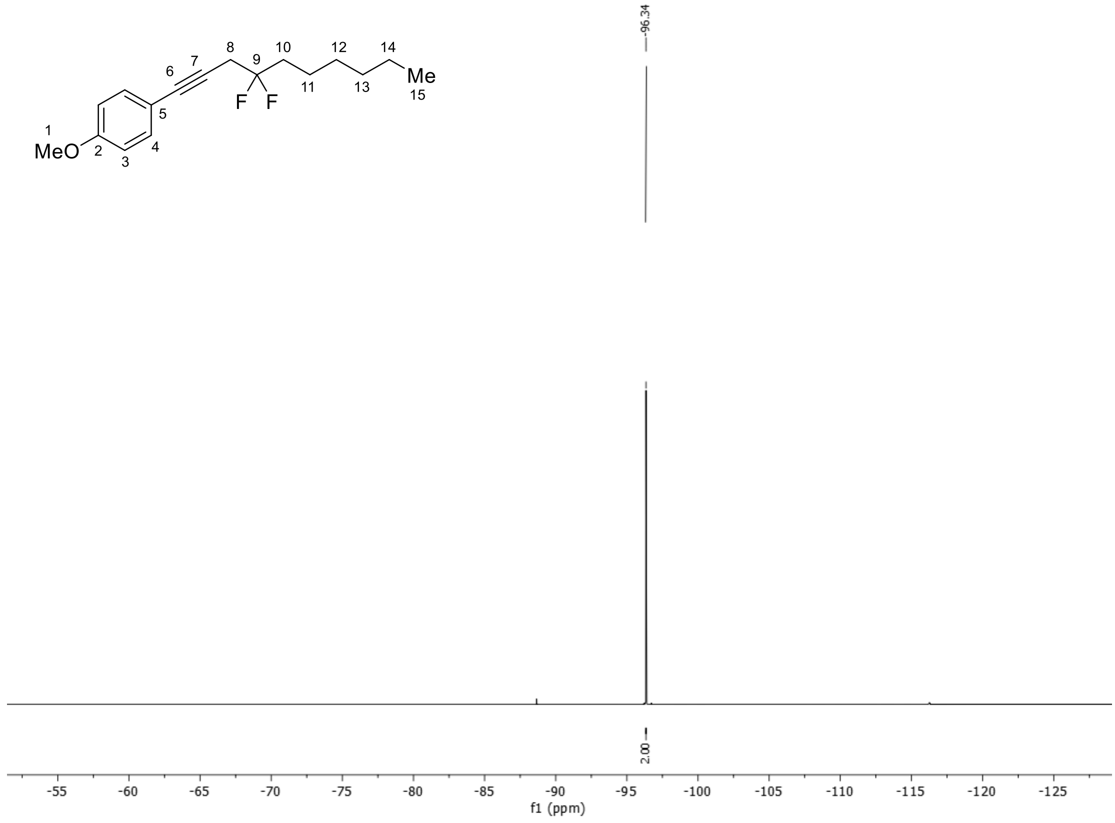

Supplementary Figure 238. $^{19}F\{^1H\}$ NMR of **39** (470 MHz, 299 K, $CDCl_3$).

2-(4-(4,4-Difluoropent-1-yn-1-yl)phenyl)furan (40)

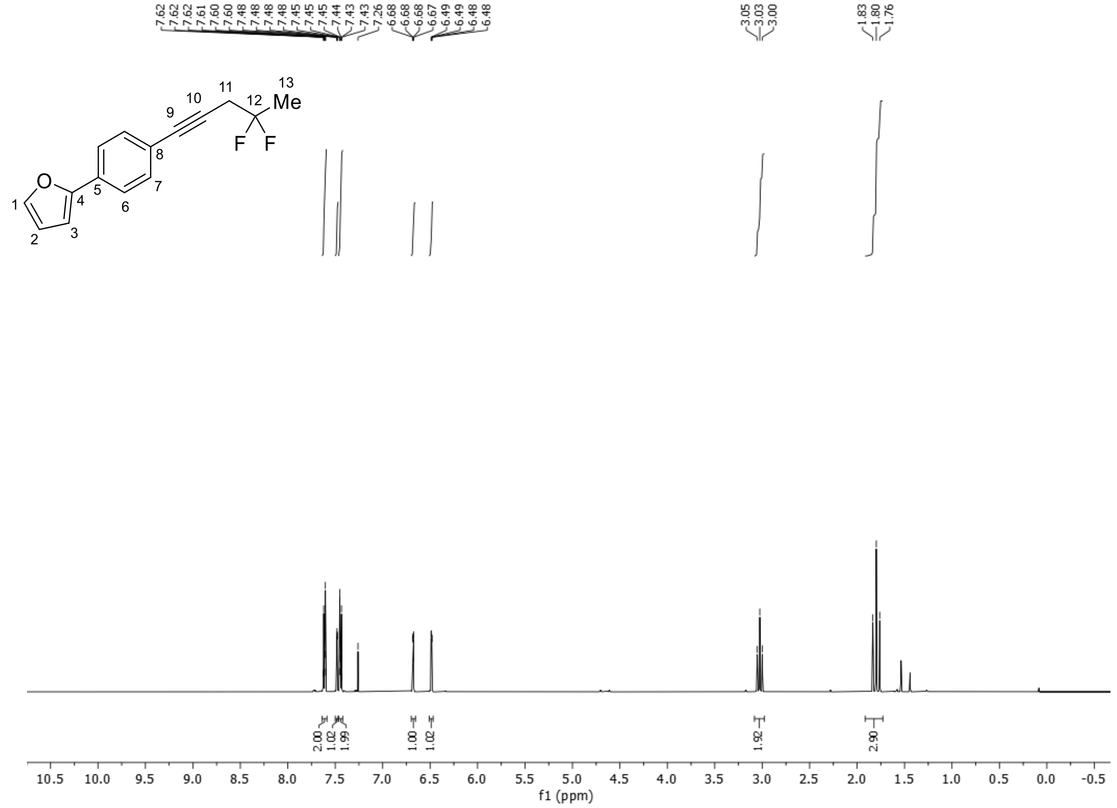

Supplementary Figure 239. ^{1}H NMR of **40** (500 MHz, 299 K, $CDCl_3$).

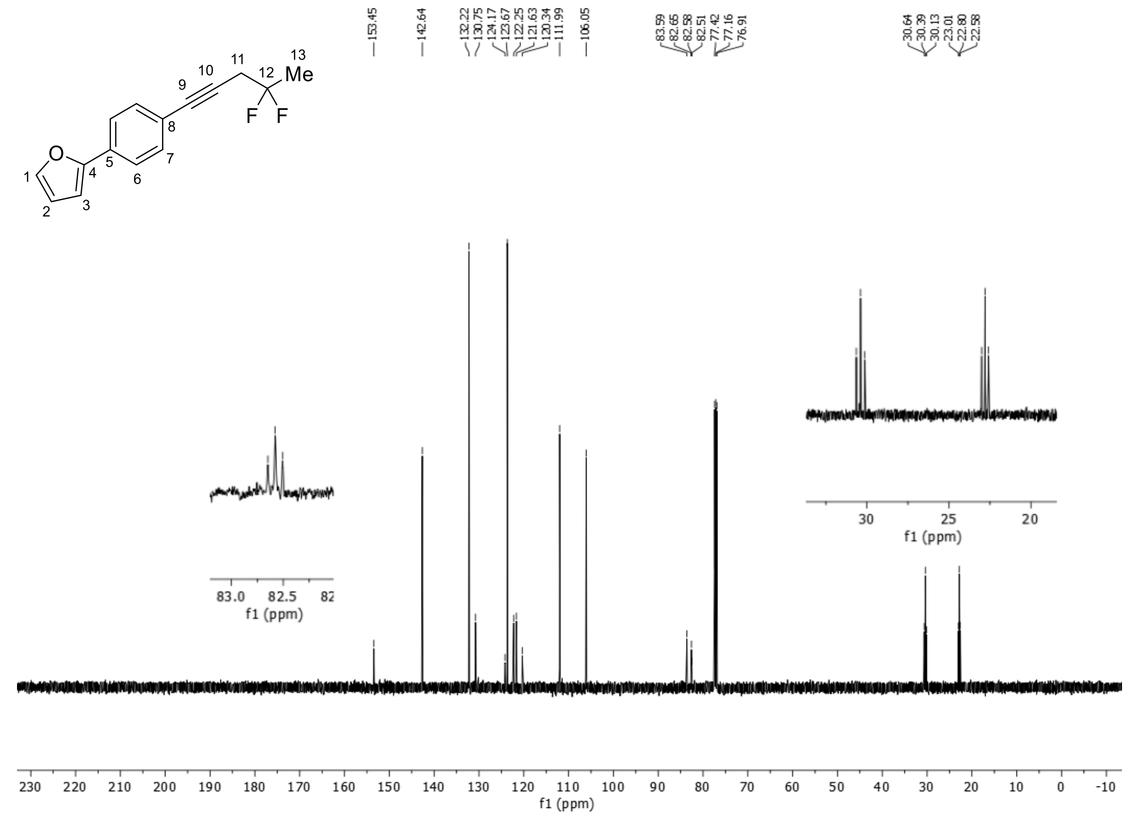

Supplementary Figure 240. $^{13}C\{^{1}H\}$ NMR of **40** (126 MHz, 299 K, $CDCl_3$).

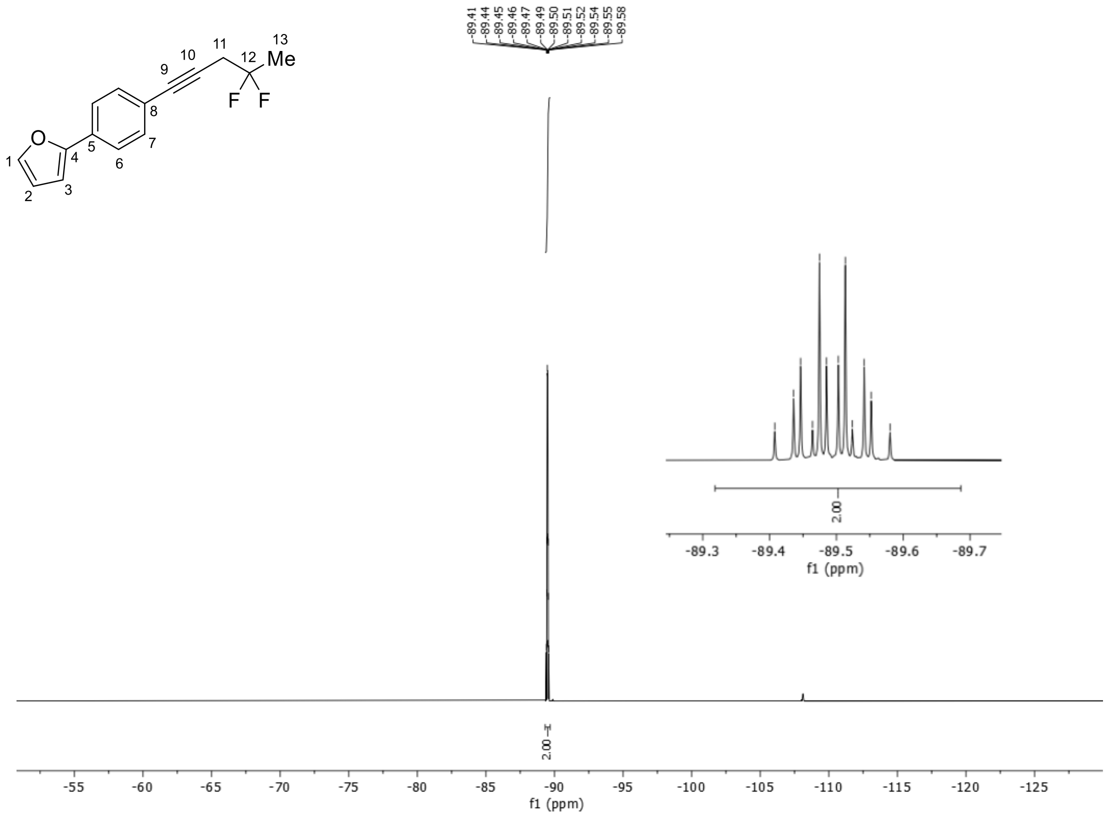

Supplementary Figure 241. ^{19}F NMR of **40** (470 MHz, 299 K, $CDCl_3$).

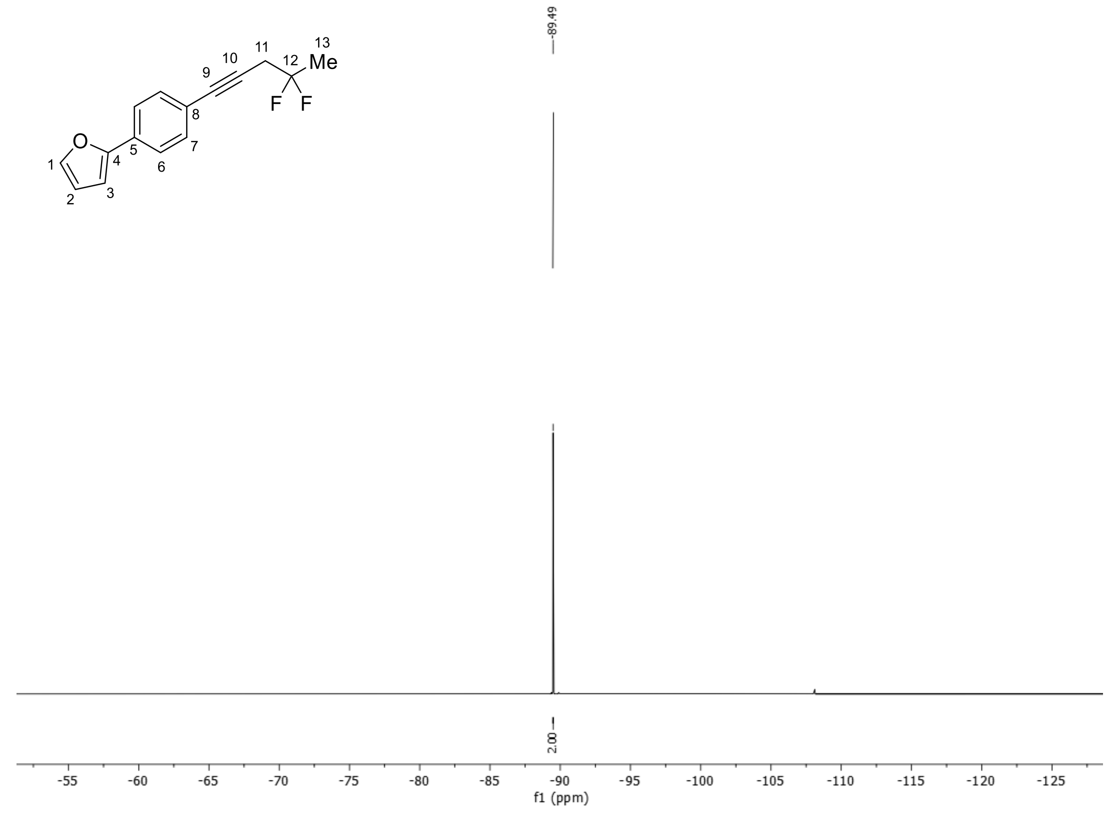

Supplementary Figure 242. $^{19}F\{^{1}H\}$ NMR of **40** (470 MHz, 299 K, $CDCl_3$).

Methyl 7-methylenepentadec-5-ynoate (41)

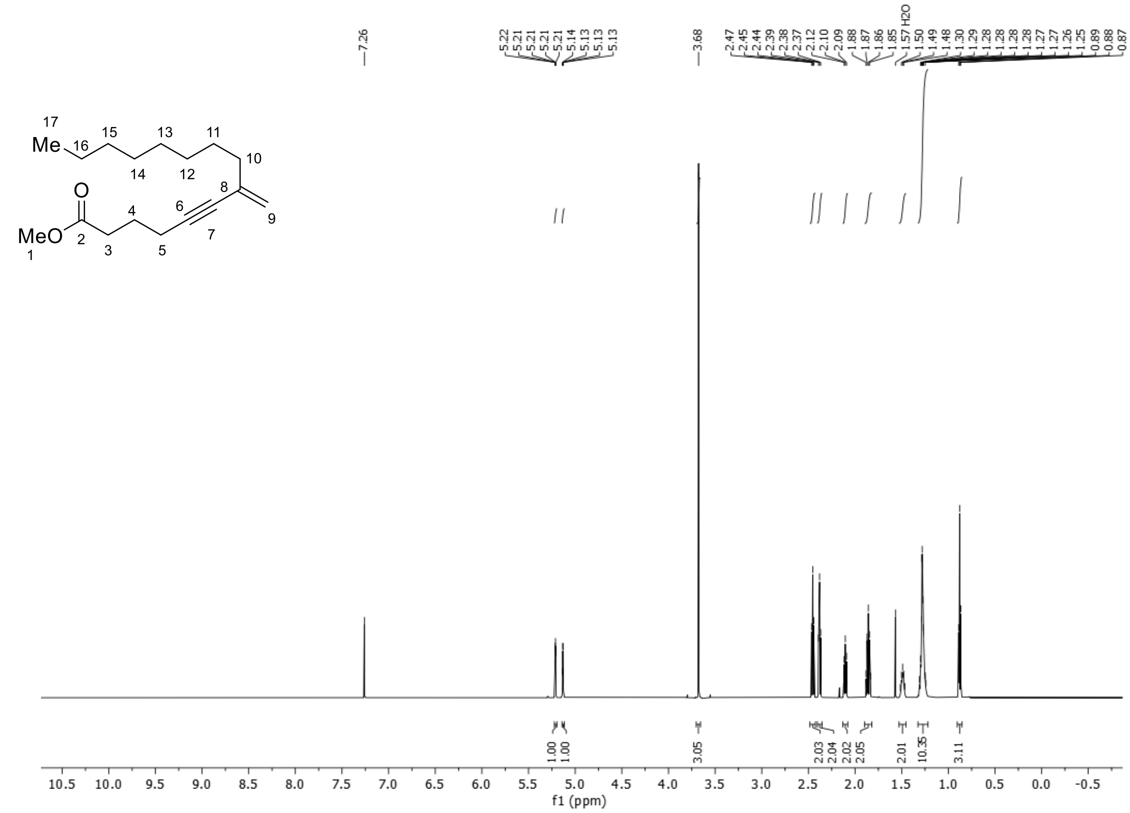

Supplementary Figure 243. ^{1}H NMR of 41 (599 MHz, 299 K, $CDCl_3$).

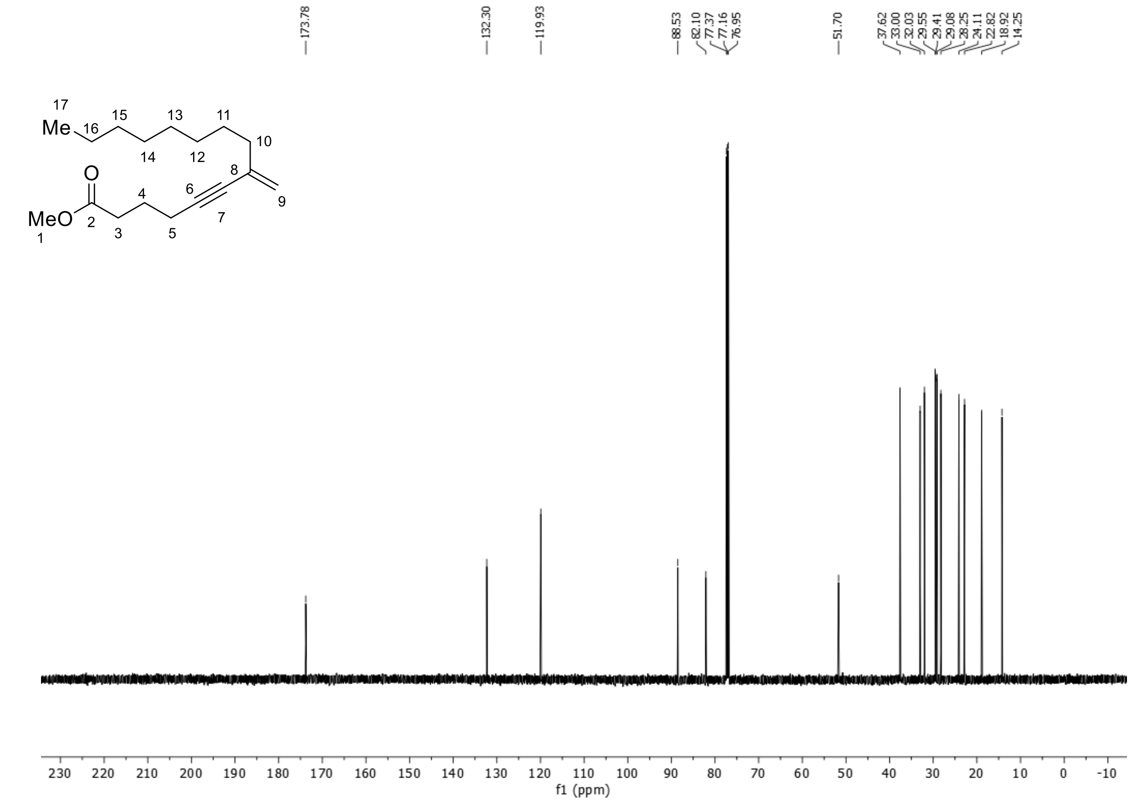

Supplementary Figure 244. $^{13}C\{^1H\}$ NMR of 41 (151 MHz, 299 K, $CDCl_3$).

Methyl 8,8-difluorohexadec-5-ynoate (42)

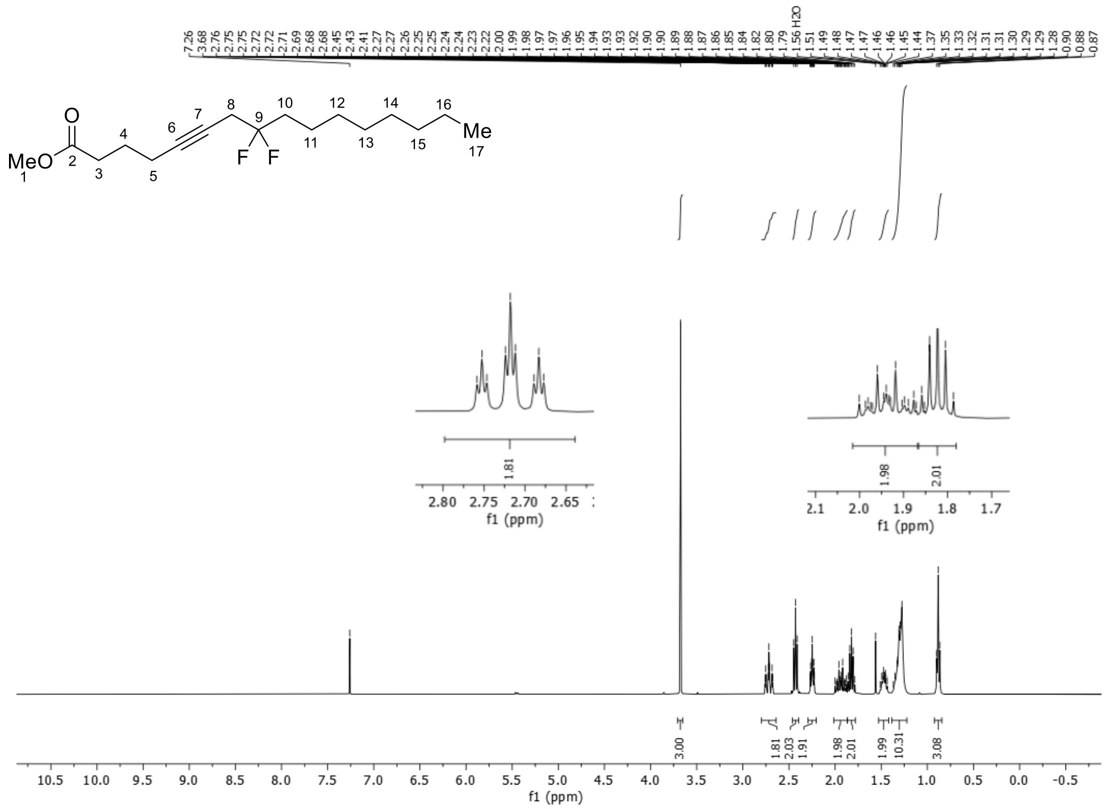

Supplementary Figure 245. ^{1}H NMR of **42** (400 MHz, 299 K, $CDCl_3$).

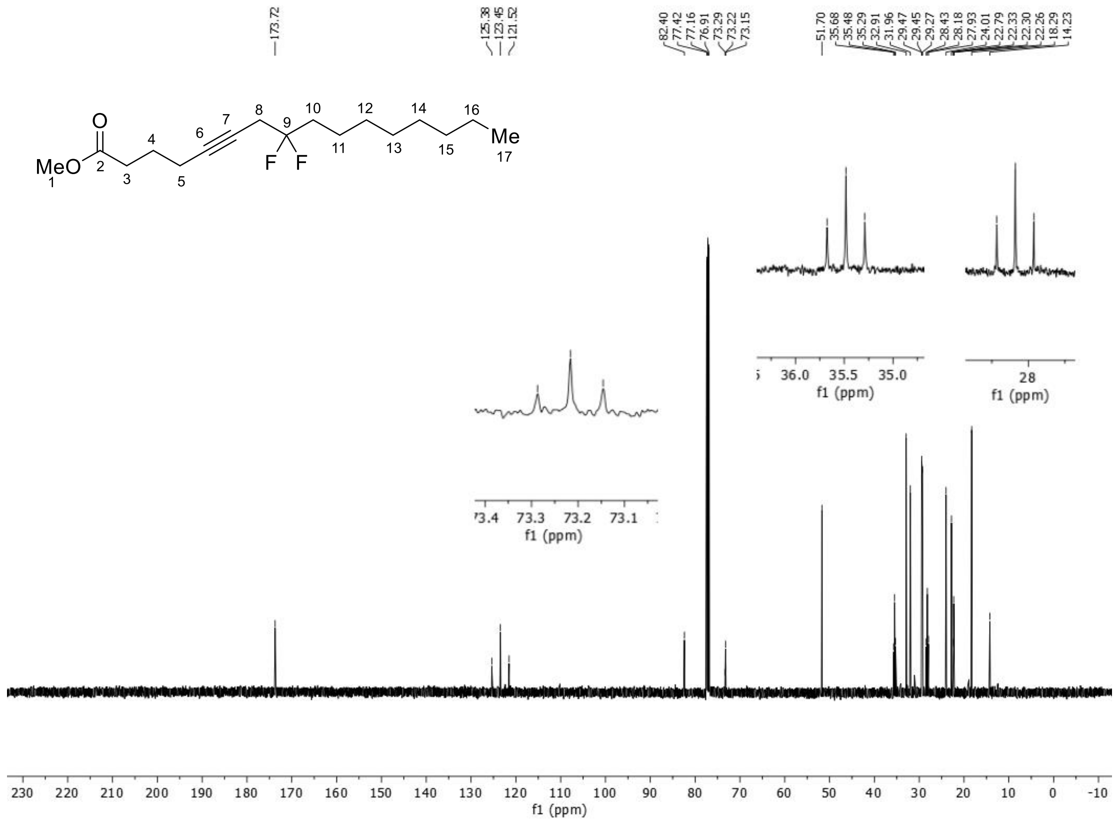

Supplementary Figure 246. $^{13}C\{^{1}H\}$ NMR of **42** (126 MHz, 299 K, $CDCl_3$).

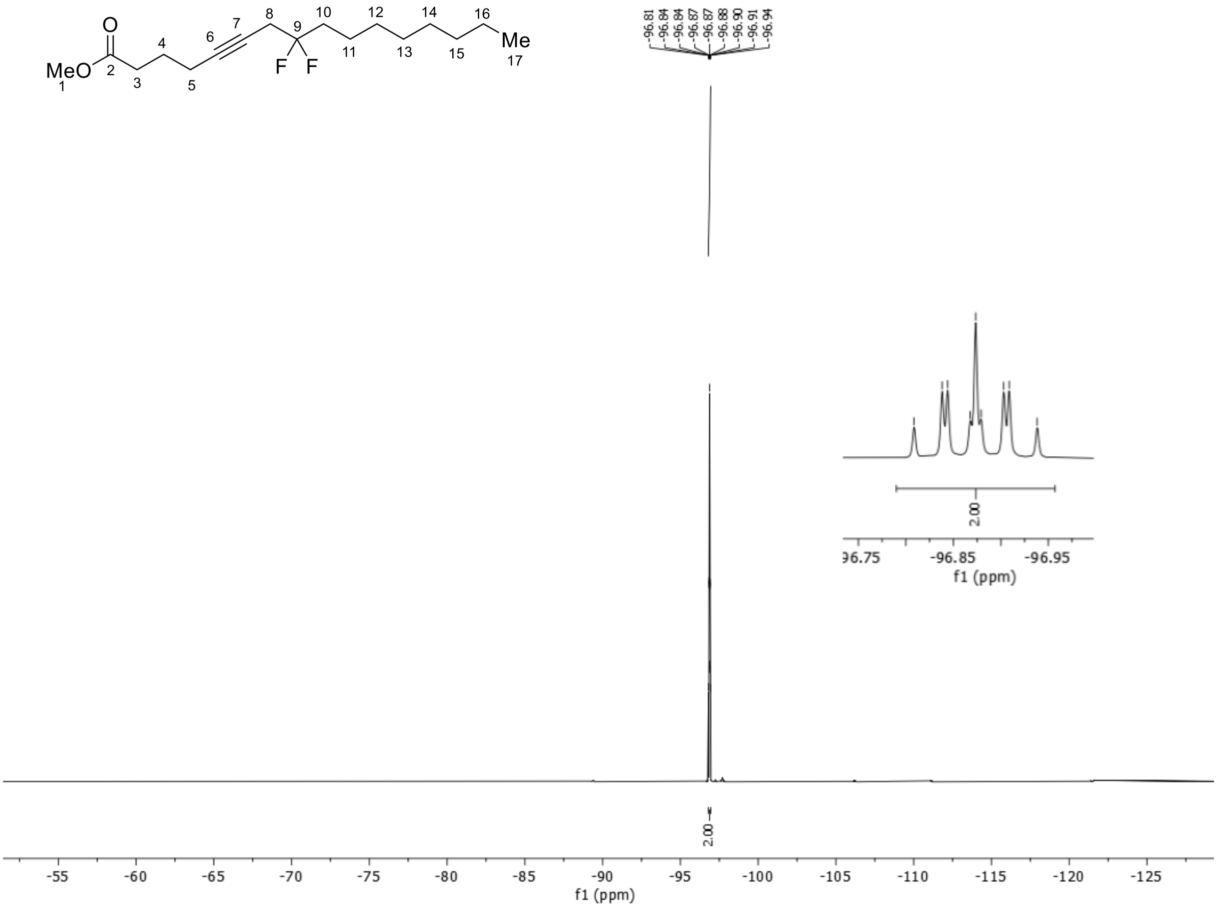

Supplementary Figure 247. ^{19}F NMR of **42** (470 MHz, 299 K, $CDCl_3$).

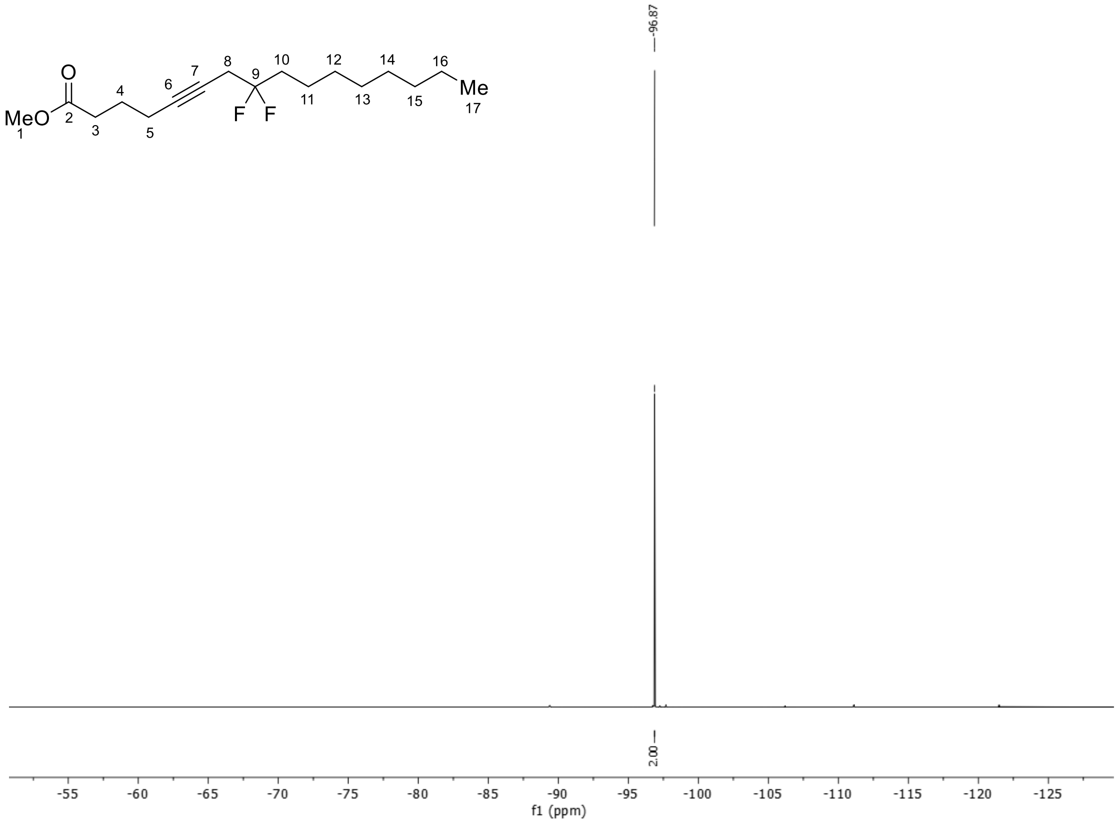

Supplementary Figure 248. $^{19}F\{^1H\}$ NMR of **42** (470 MHz, 299 K, $CDCl_3$).

8,8-Difluorohexadecanoic acid (44)

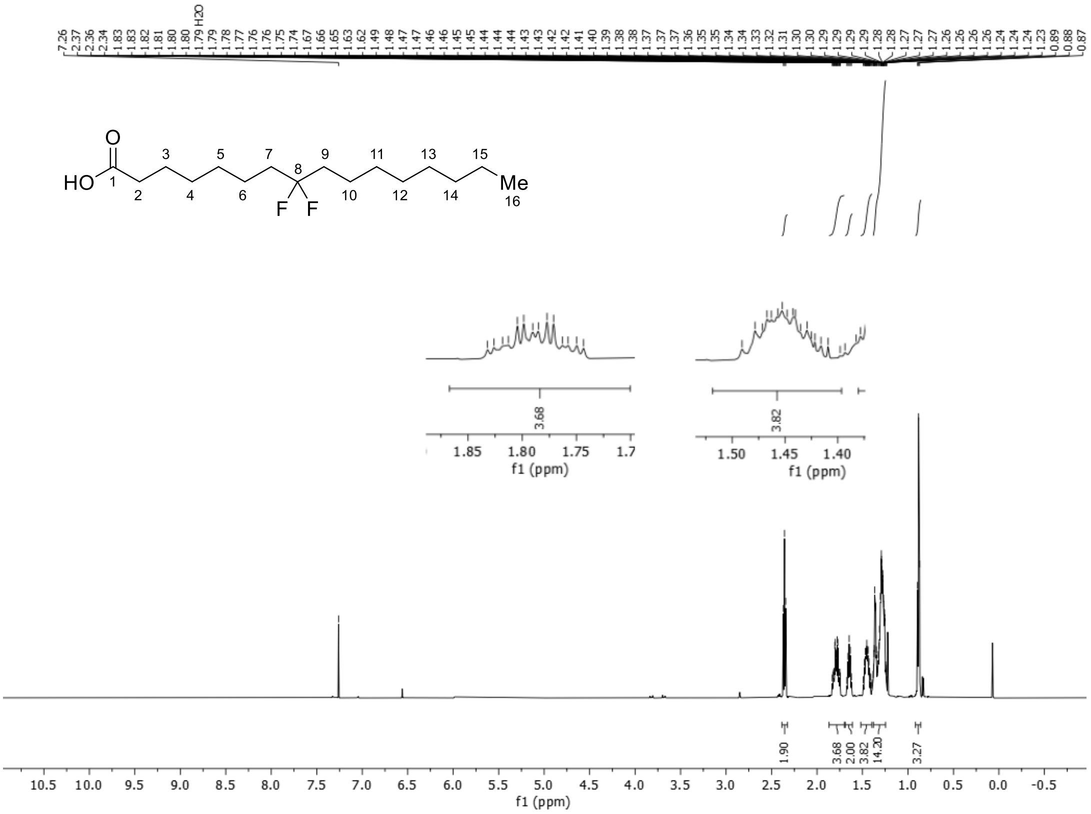

Supplementary Figure 249. ^{1}H NMR of **44** (599 MHz, 299 K, $CDCl_3$).

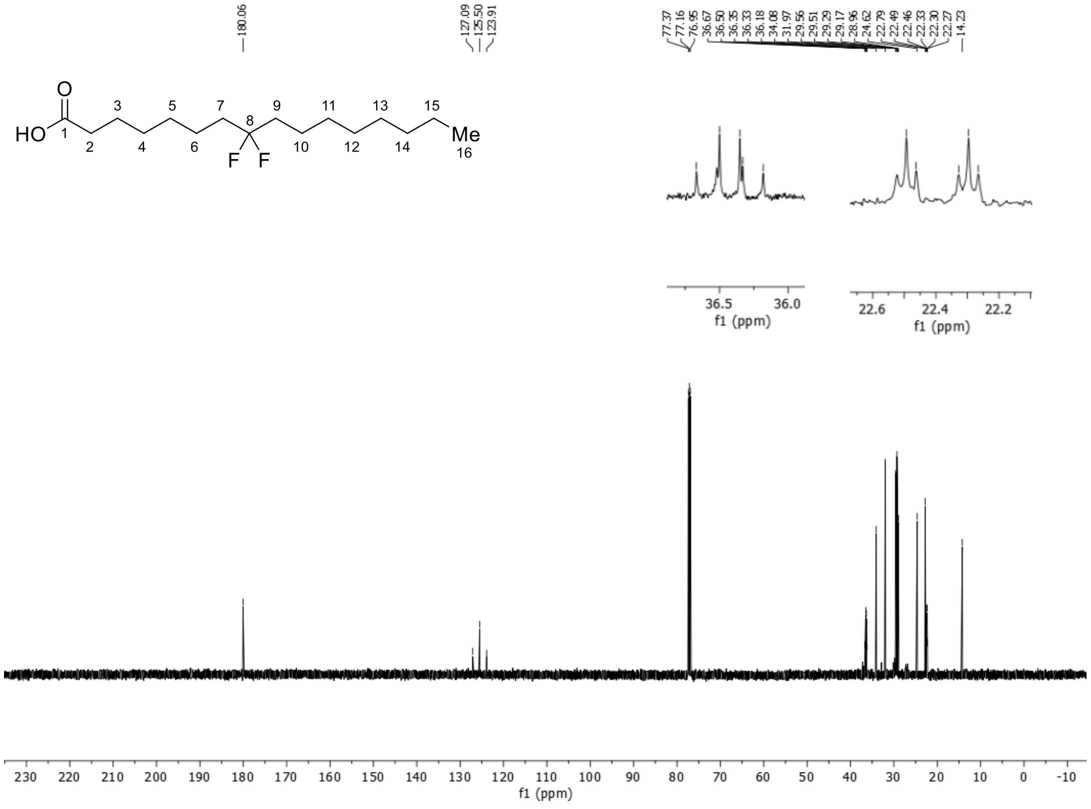

Supplementary Figure 250. $^{13}C\{^{1}H\}$ NMR of **44** (151 MHz, 299 K, $CDCl_3$).

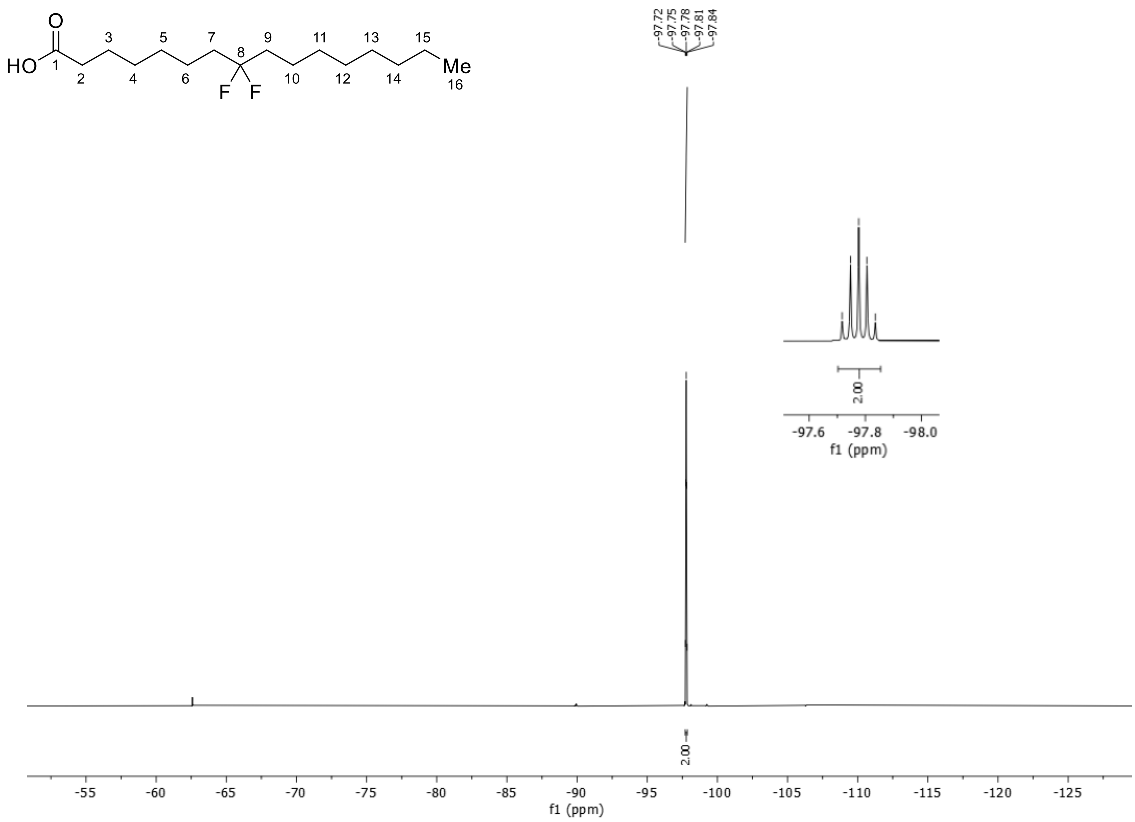

Supplementary Figure 251. ^{19}F NMR of **44** (564 MHz, 299 K, $CDCl_3$).

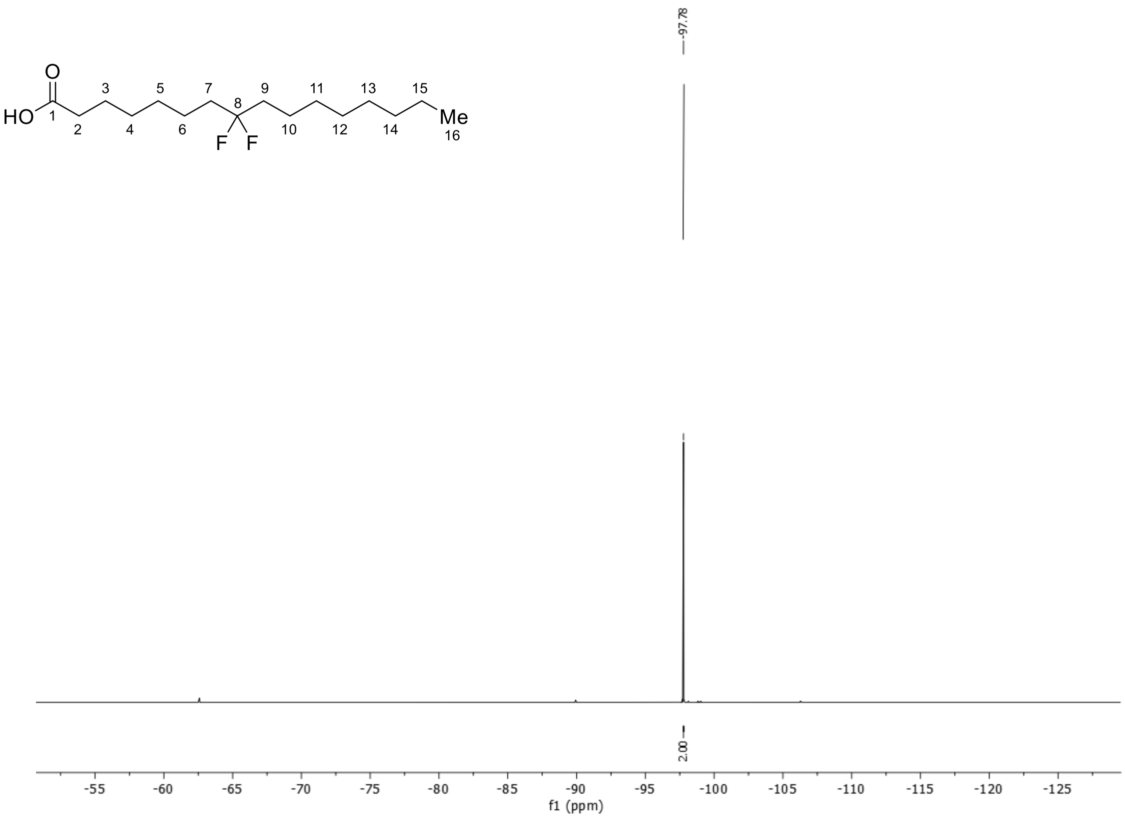

Supplementary Figure 252. ^{19}F{^{1}H} NMR of **44** (564 MHz, 299 K, $CDCl_3$).

1-(But-3-en-1-yn-1-yl)-4-nitrobenzene (45)

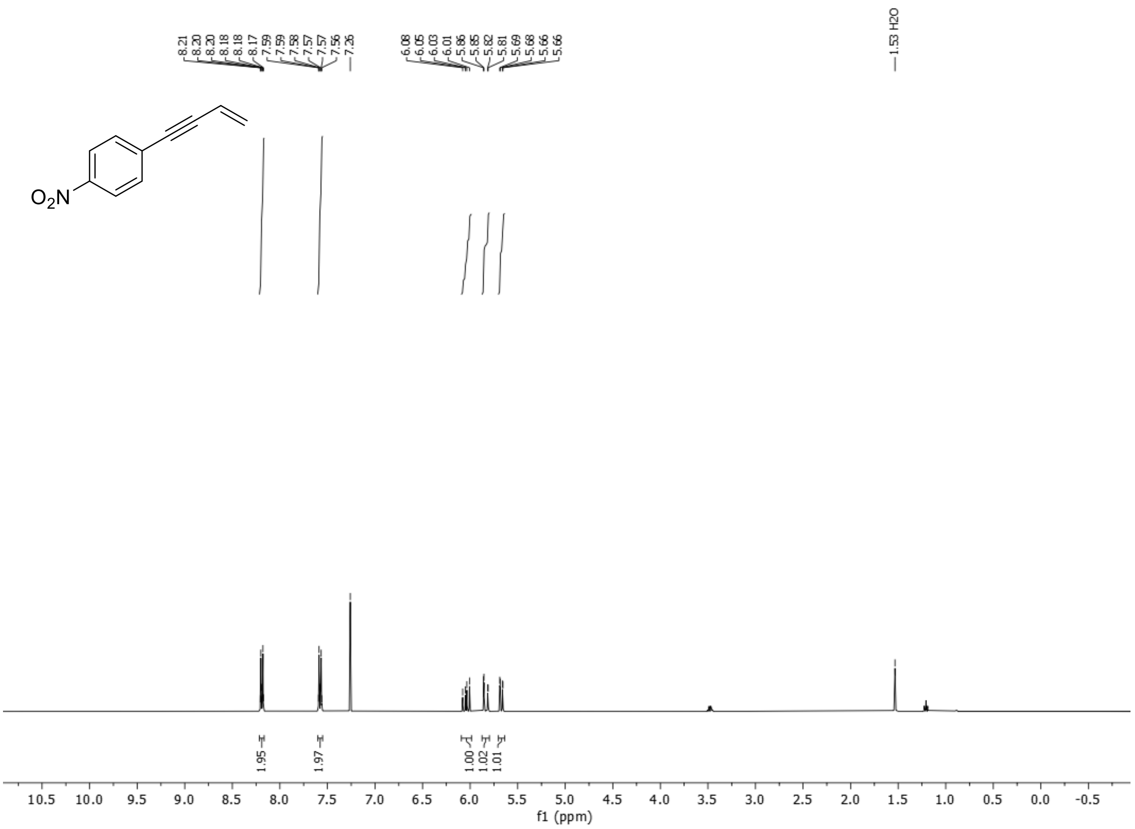

Supplementary Figure 253. ^{1}H NMR of **45** (400 MHz, 299 K, $CDCl_3$).

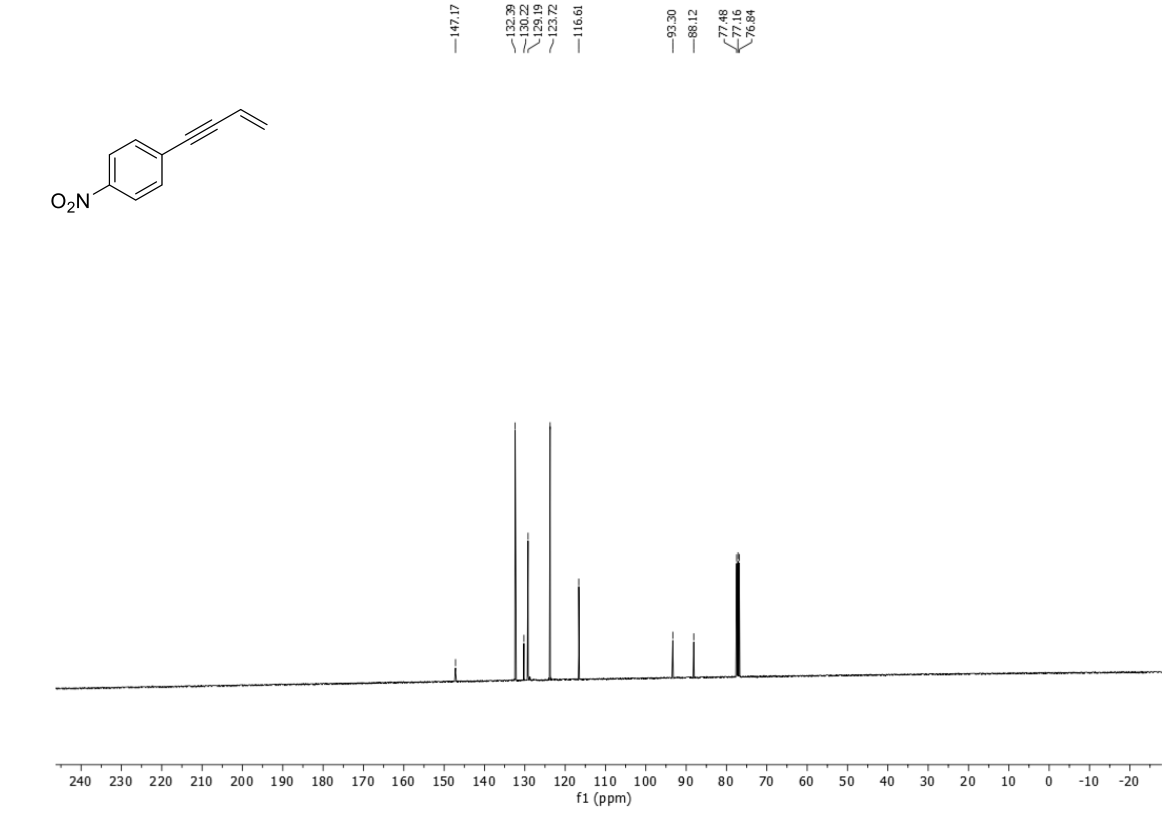

Supplementary Figure 254. $^{13}C\{^{1}H\}$ NMR of **45** (101 MHz, 299 K, $CDCl_3$).

1-(4,4-Difluorobut-1-yn-1-yl)-4-nitrobenzene (46)

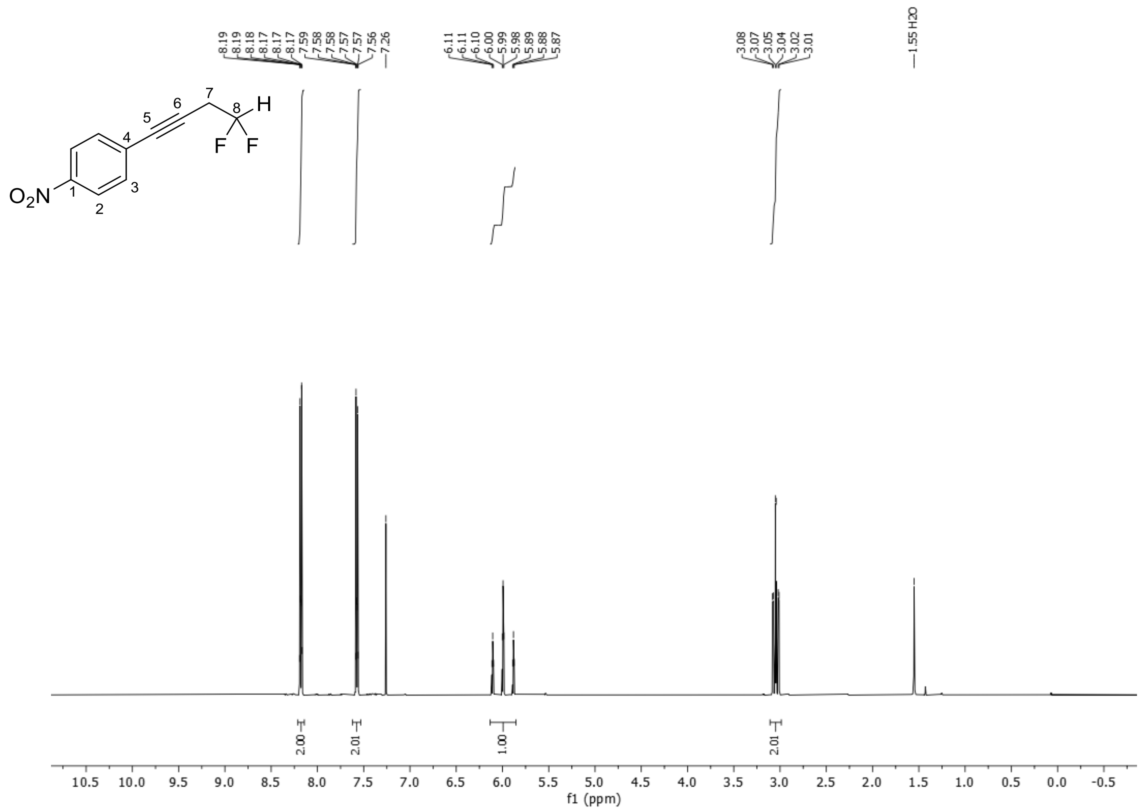

Supplementary Figure 255. ^{1}H NMR of **46** (500 MHz, 299 K, $CDCl_3$).

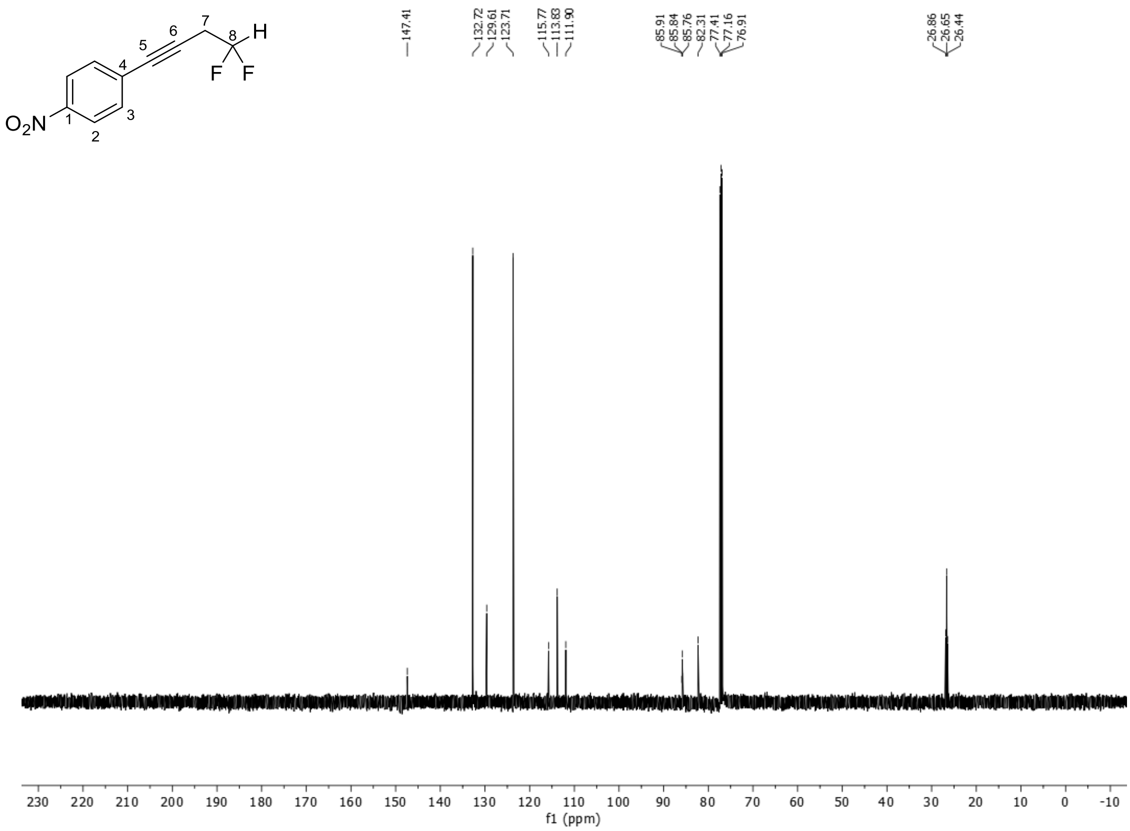

Supplementary Figure 256. $^{13}C\{^{1}H\}$ NMR of **46** (126 MHz, 299 K, $CDCl_3$).

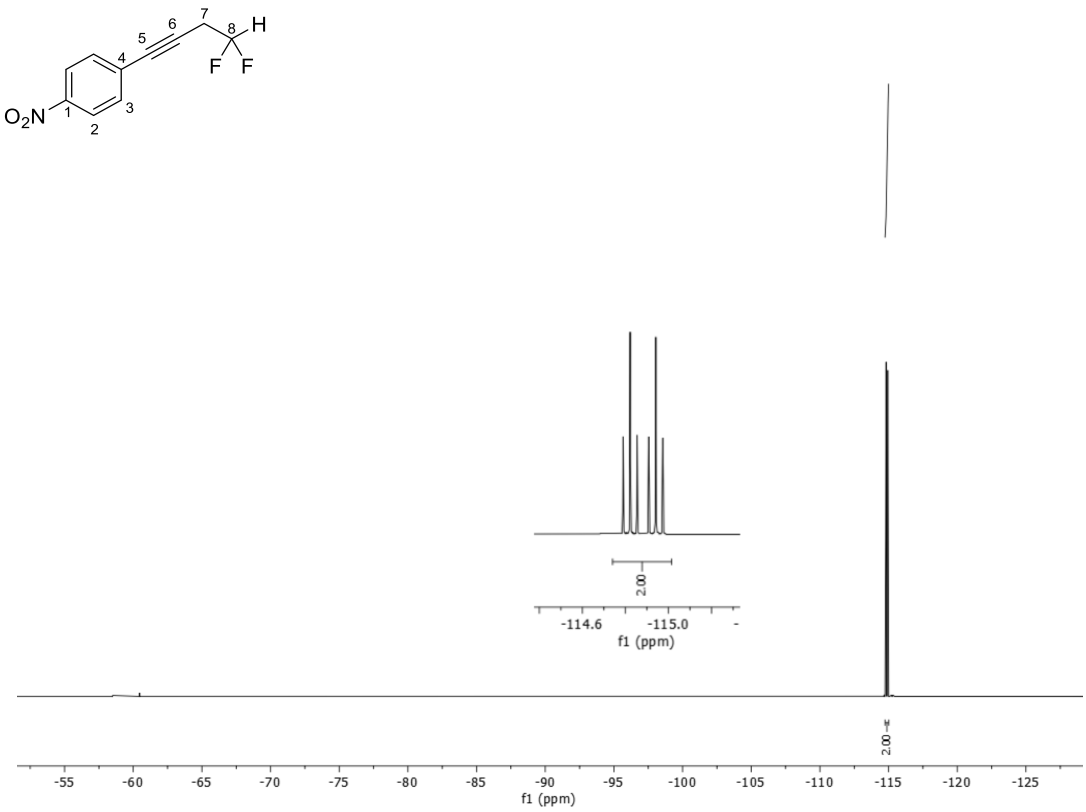

Supplementary Figure 257. ^{19}F NMR of **46** (470 MHz, 299 K, $CDCl_3$).

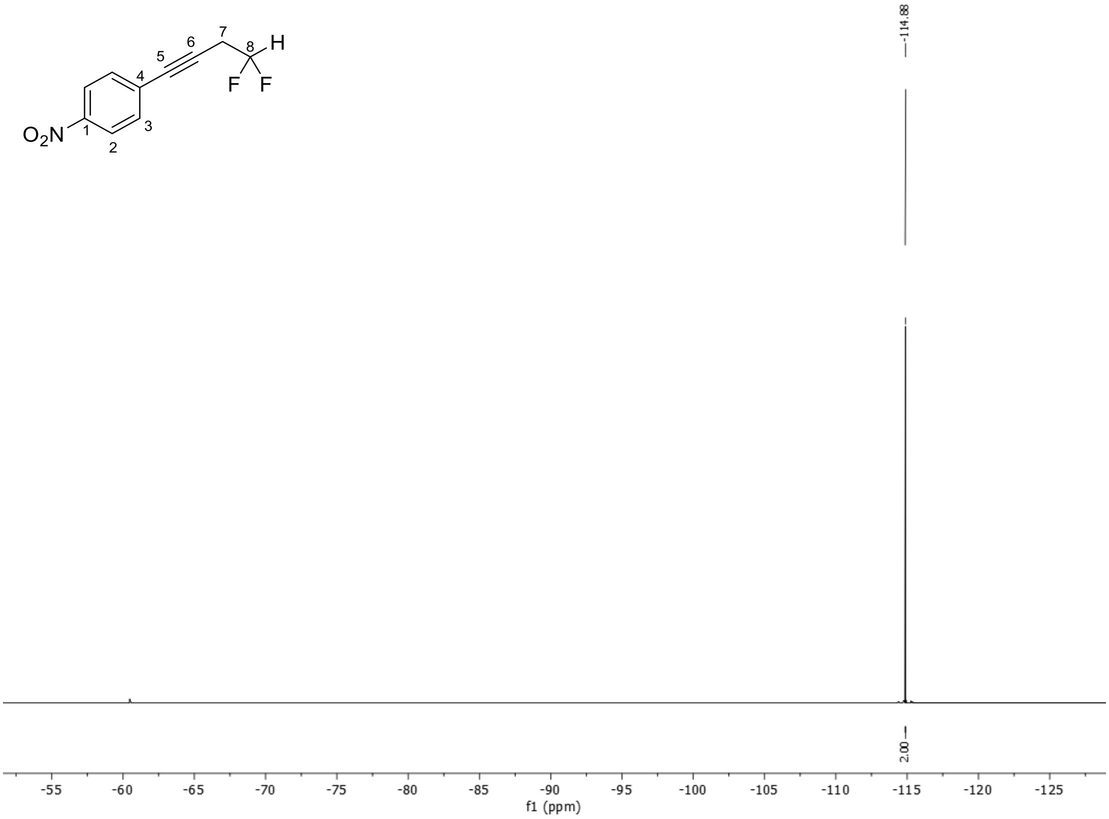

Supplementary Figure 258. $^{19}F\{^1H\}$ NMR of **46** (470 MHz, 299 K, $CDCl_3$).

Heptadec-1-en-3-yne (47)

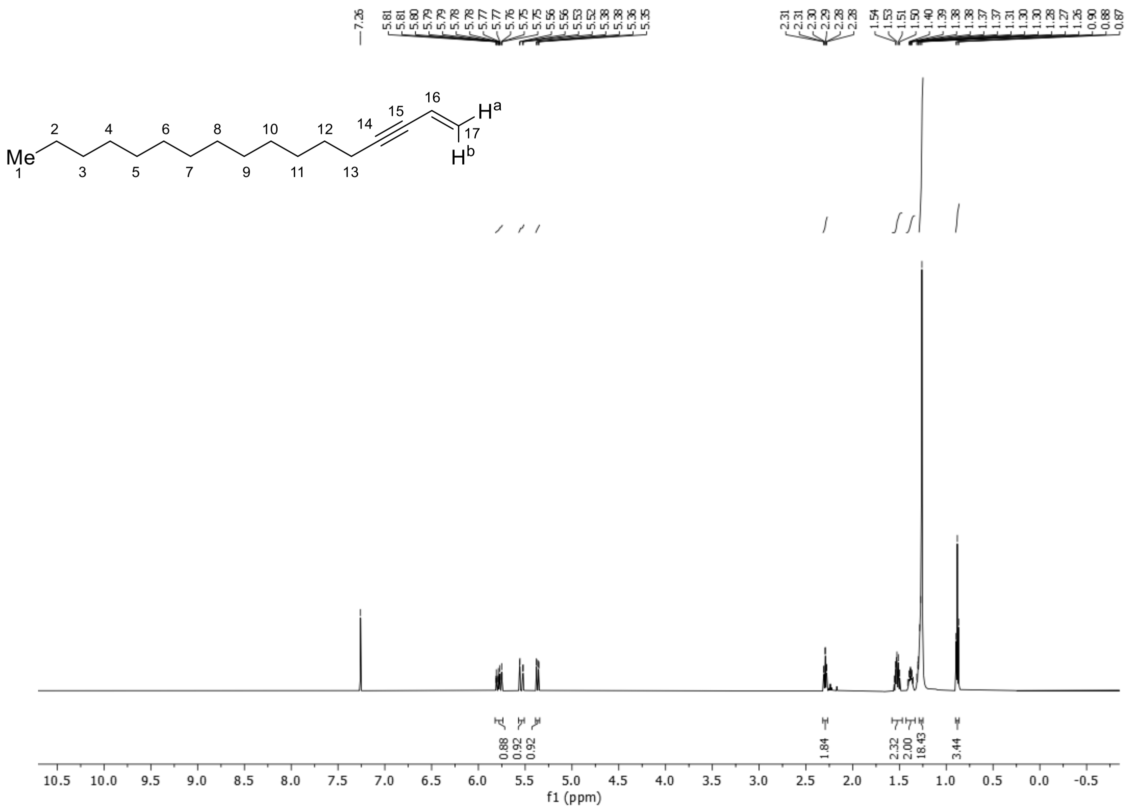

Supplementary Figure 259. ^{1}H NMR of **47** (500 MHz, 299 K, $CDCl_3$).

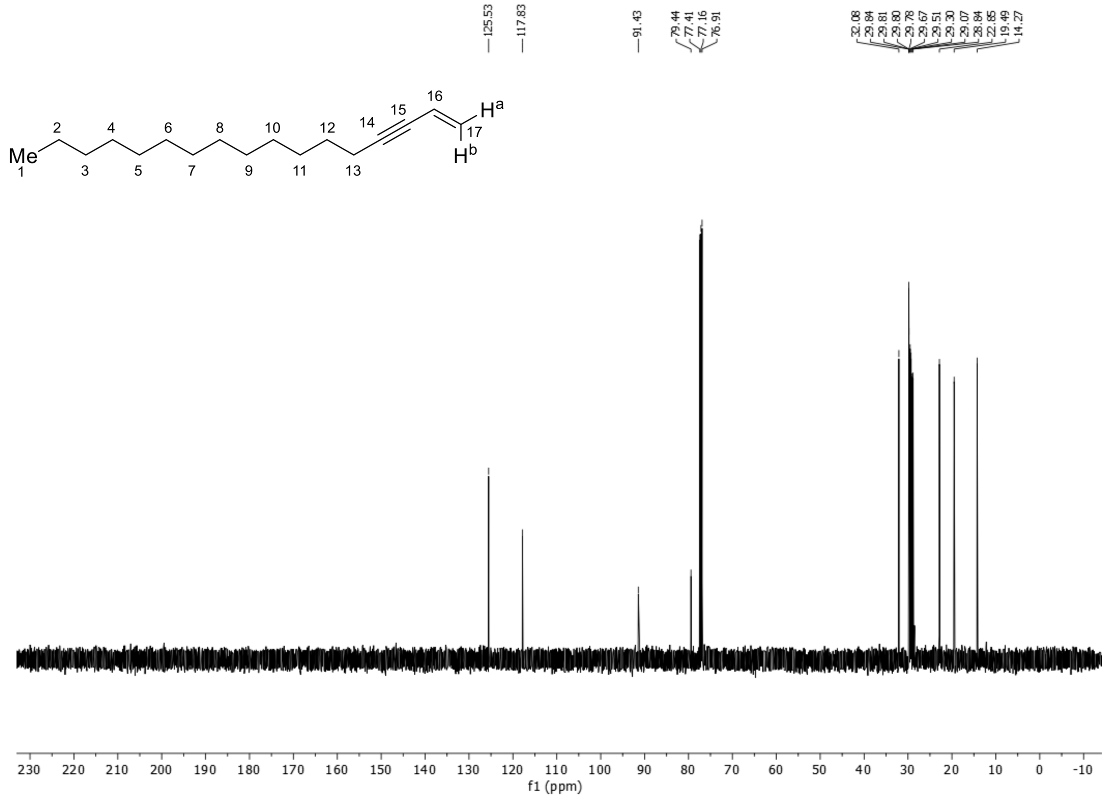

Supplementary Figure 260. $^{13}C\{^{1}H\}$ NMR of **47** (126 MHz, 299 K, $CDCl_3$).

1,1-Difluoroheptadec-3-yne (48)

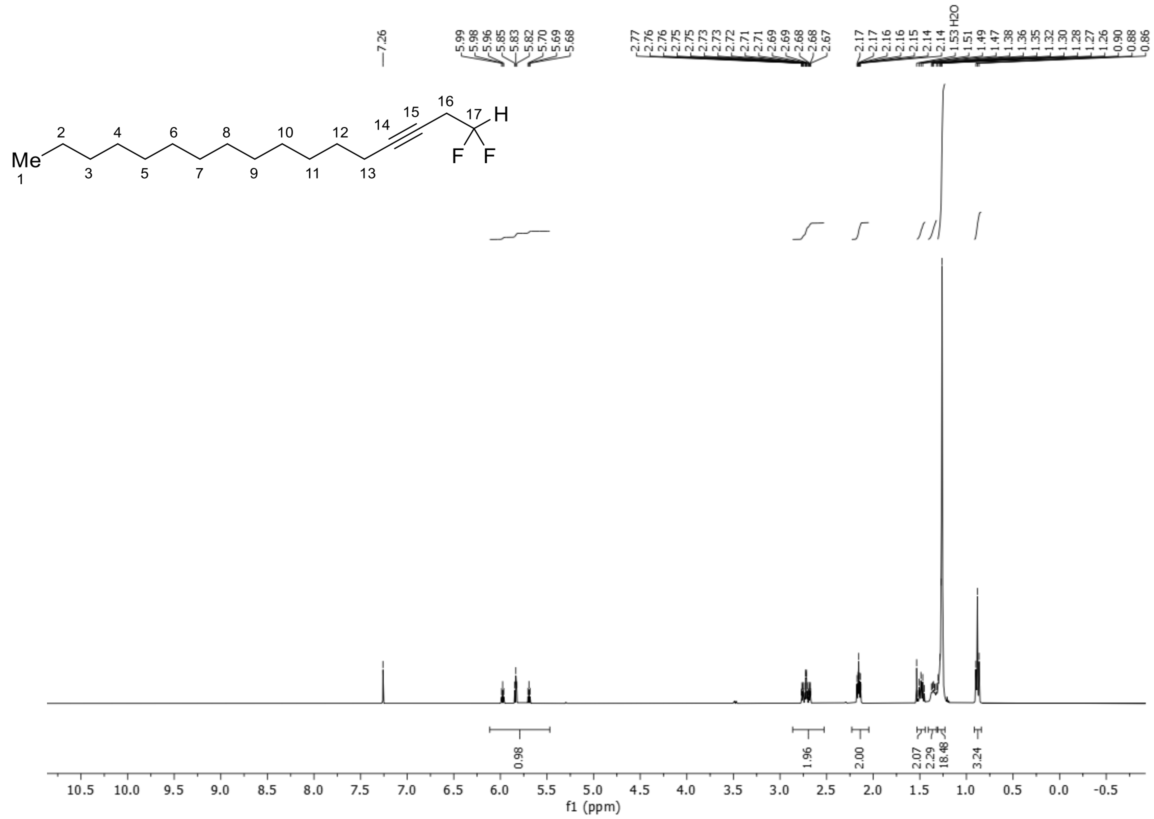

Supplementary Figure 261. ^{1}H NMR of **48** (500 MHz, 299 K, $CDCl_3$).

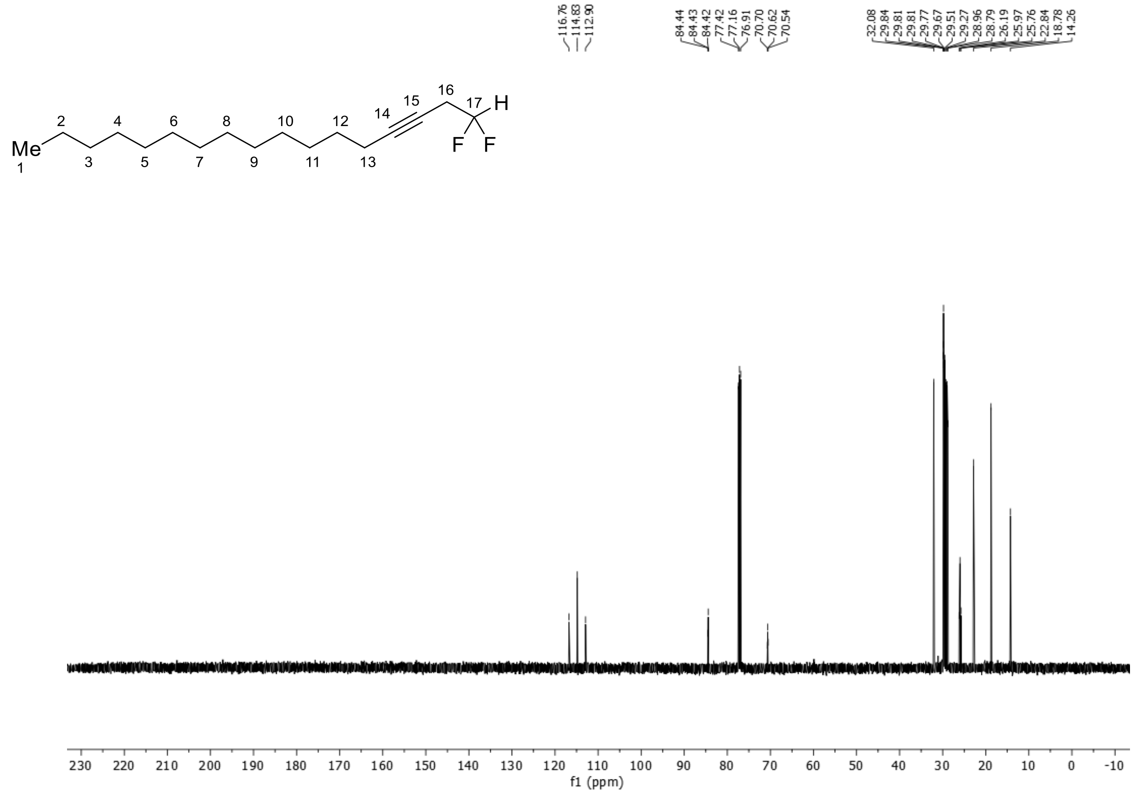

Supplementary Figure 262. $^{13}C\{^{1}H\}$ NMR of **48** (126 MHz, 299 K, $CDCl_3$).

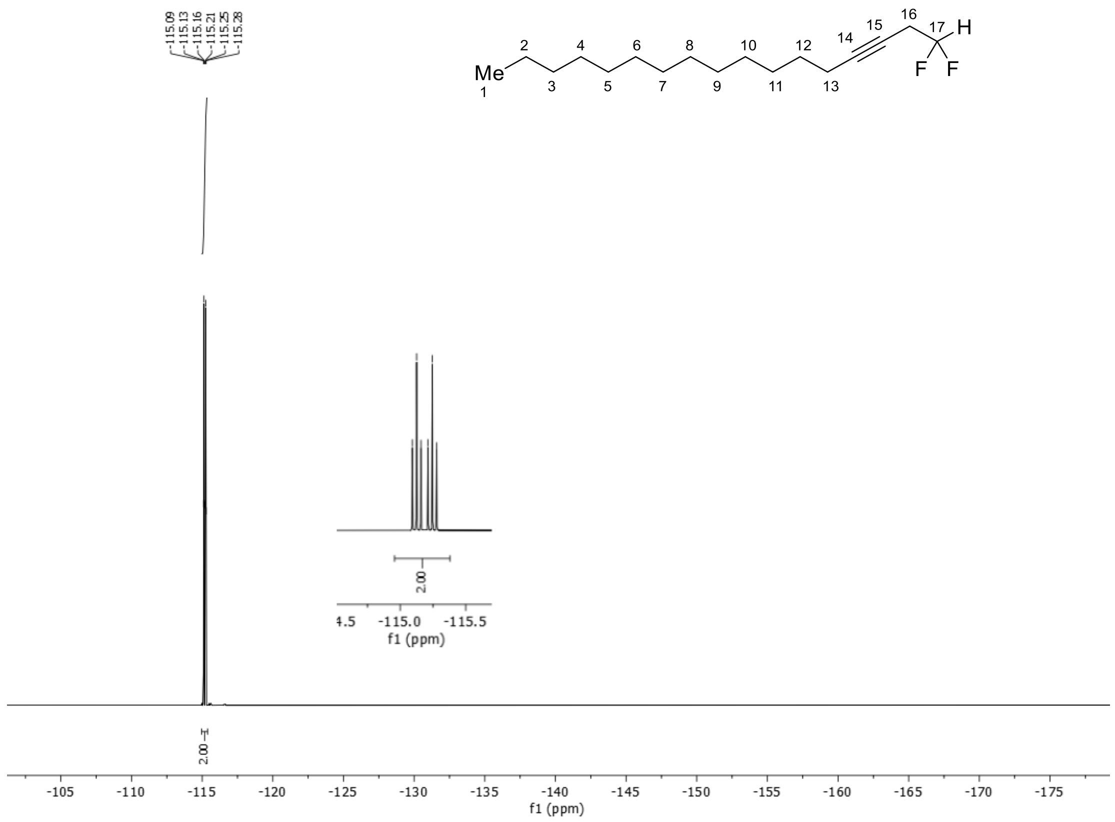

Supplementary Figure 263. ^{19}F NMR of **48** (470 MHz, 299 K, $CDCl_3$).

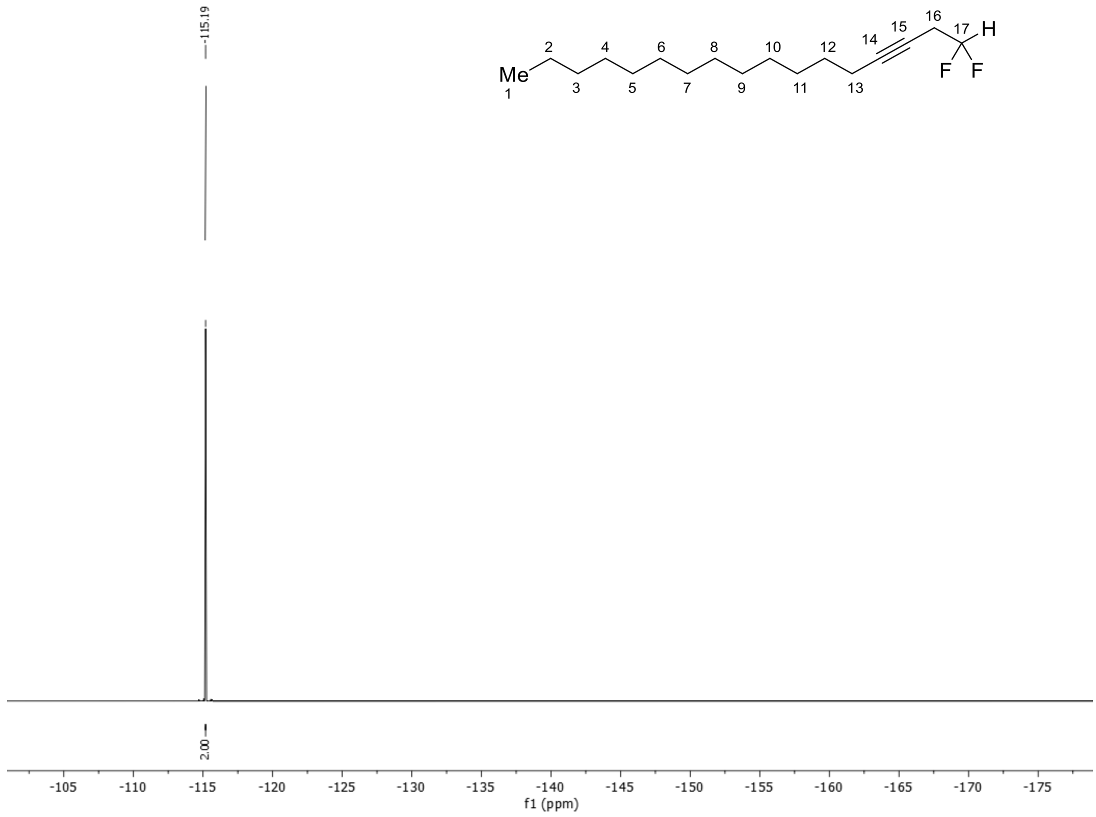

Supplementary Figure 264. $^{19}F\{^1H\}$ NMR of **48** (470 MHz, 299 K, $CDCl_3$).

1-(Dec-1-en-3-yn-2-yl)-4-(trifluoromethyl)benzene (49)

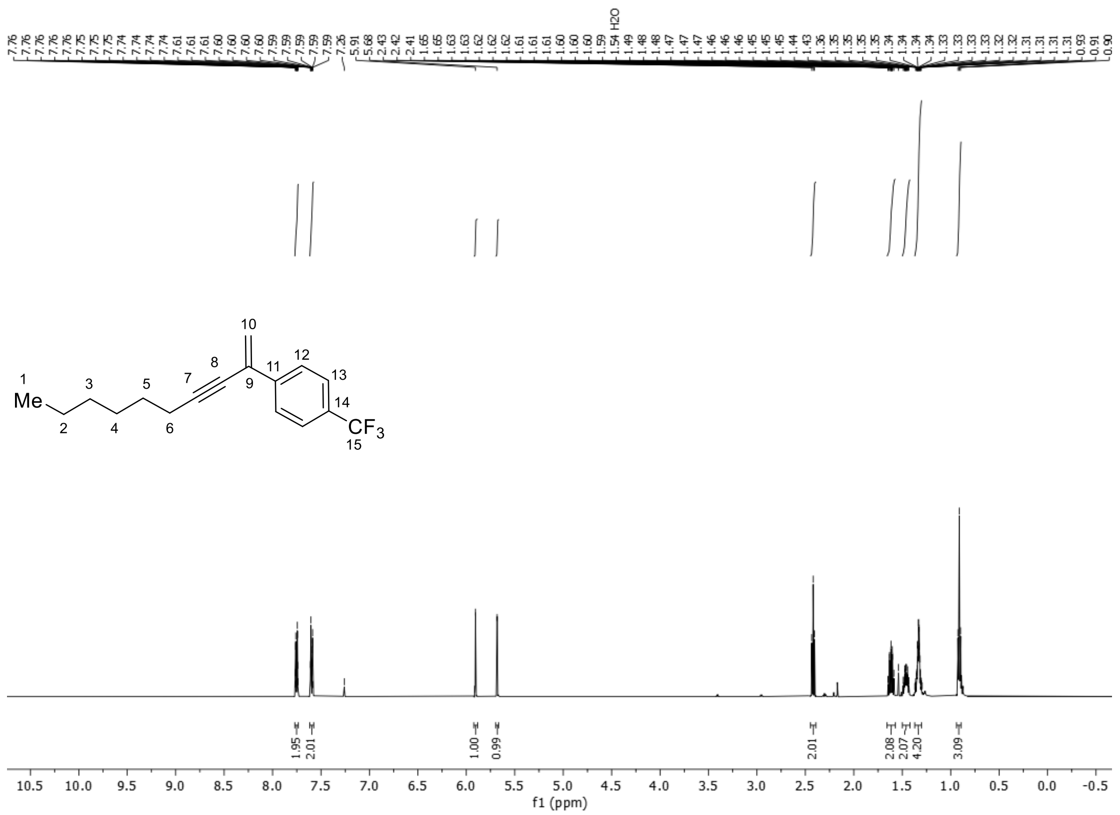

Supplementary Figure 265. ^{1}H NMR of **49** (500 MHz, 299 K, $CDCl_3$).

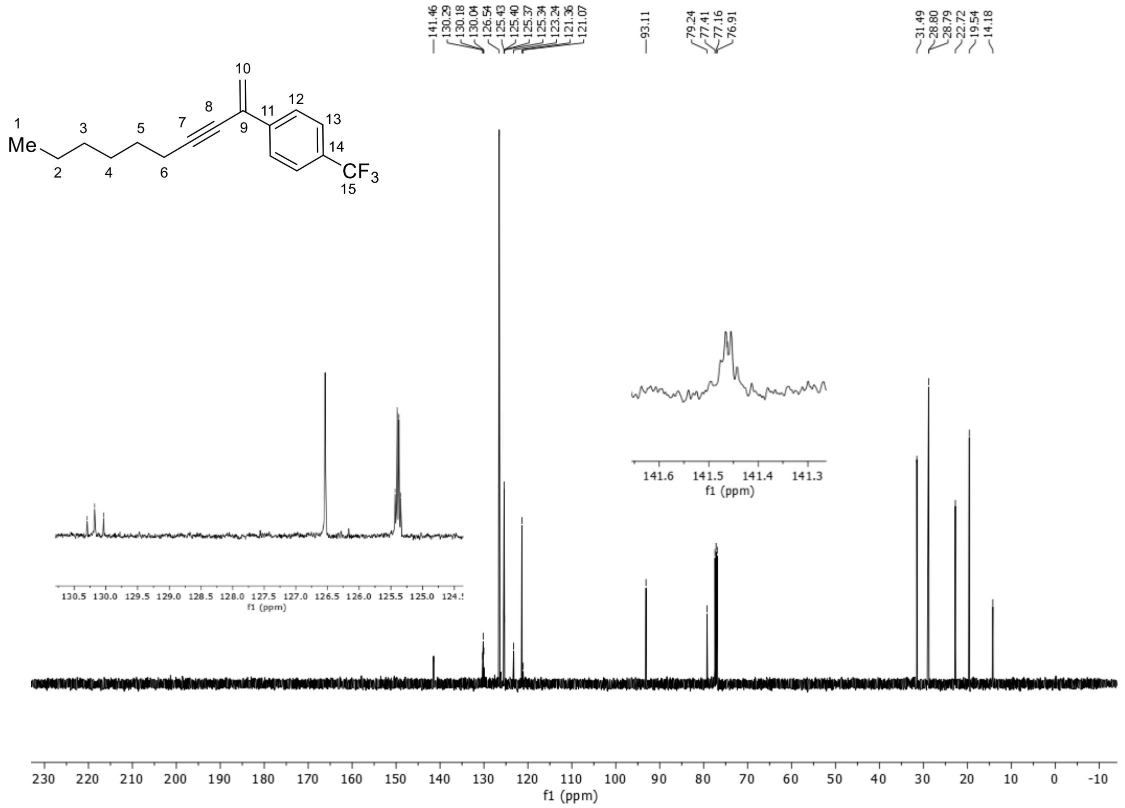

Supplementary Figure 266. $^{13}C\{^{1}H\}$ NMR of **49** (126 MHz, 299 K, $CDCl_3$).

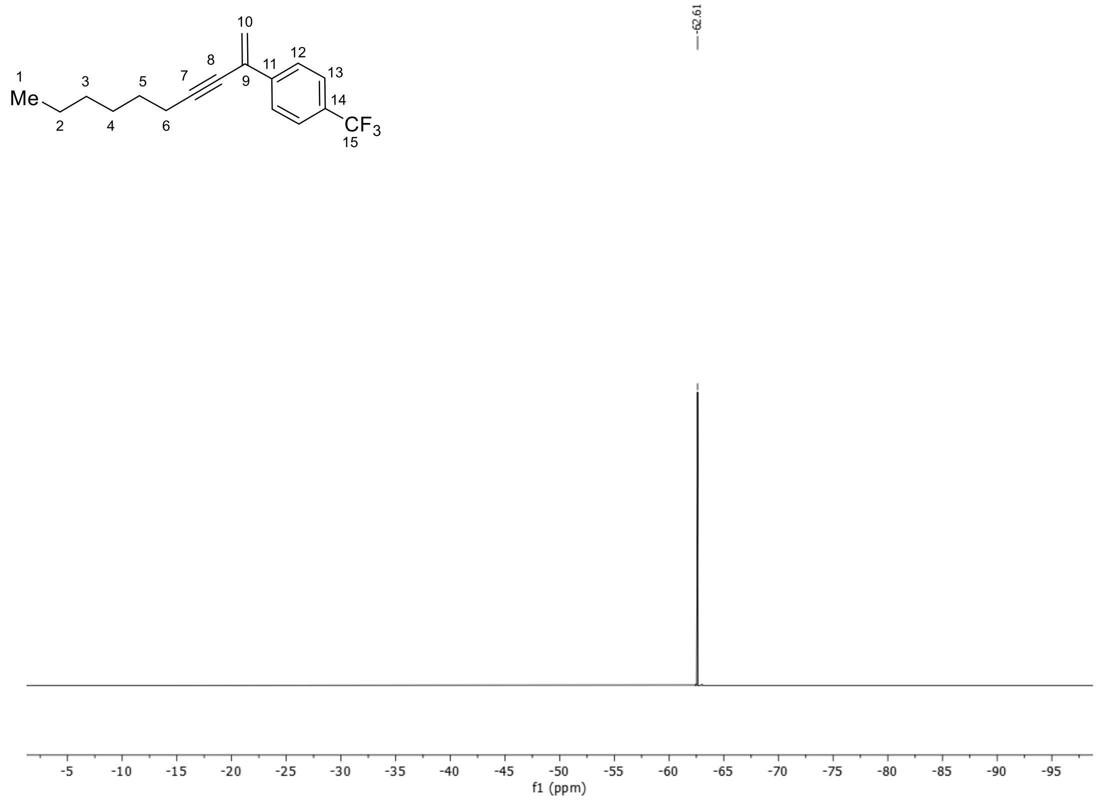

Supplementary Figure 267. ^{19}F NMR of **49** (470 MHz, 299 K, $CDCl_3$).

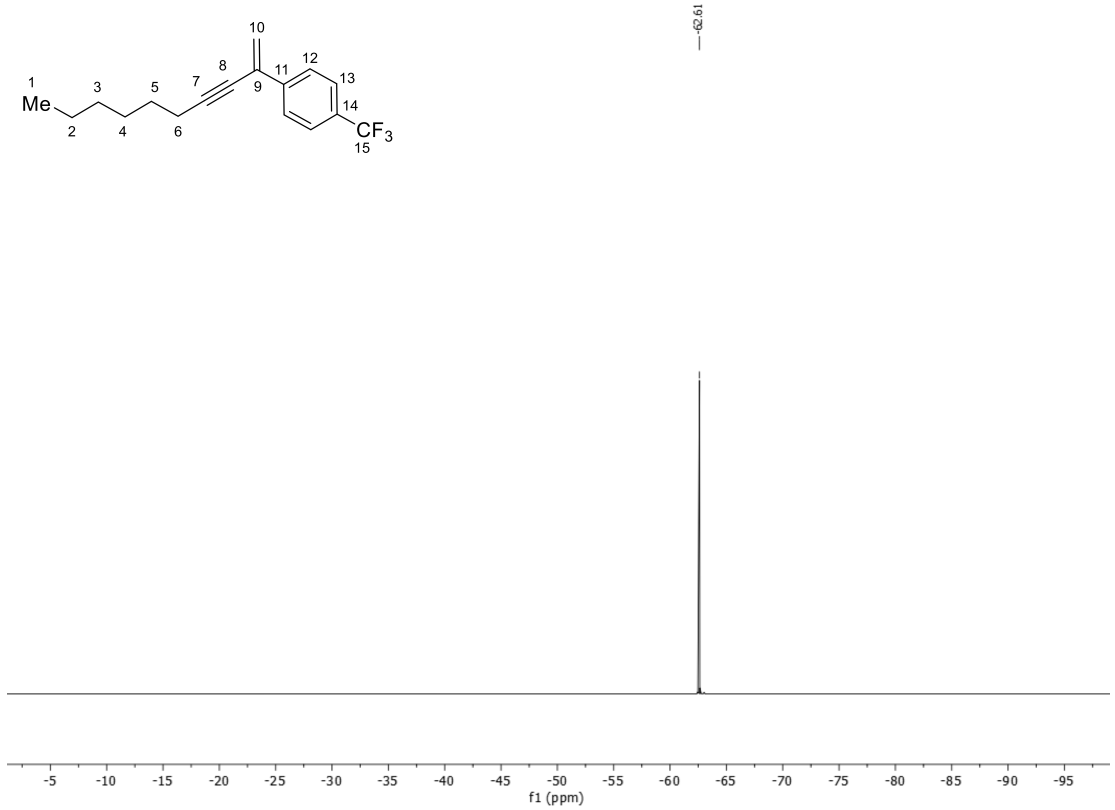

Supplementary Figure 268. $^{19}F\{^1H\}$ NMR of **49** (470 MHz, 299 K, $CDCl_3$).

1-(1,1-Difluorodec-3-yn-1-yl)-4-(trifluoromethyl)benzene (50)

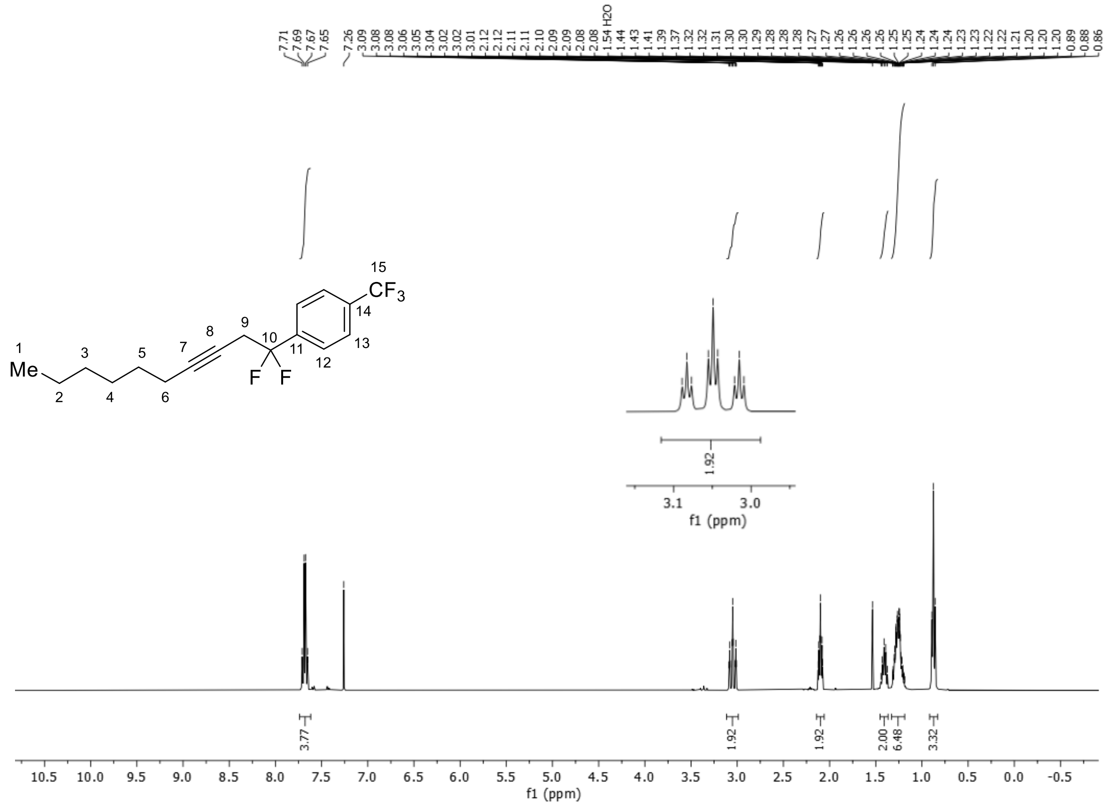

Supplementary Figure 269. ^{1}H NMR of **50** (400 MHz, 299 K, $CDCl_3$).

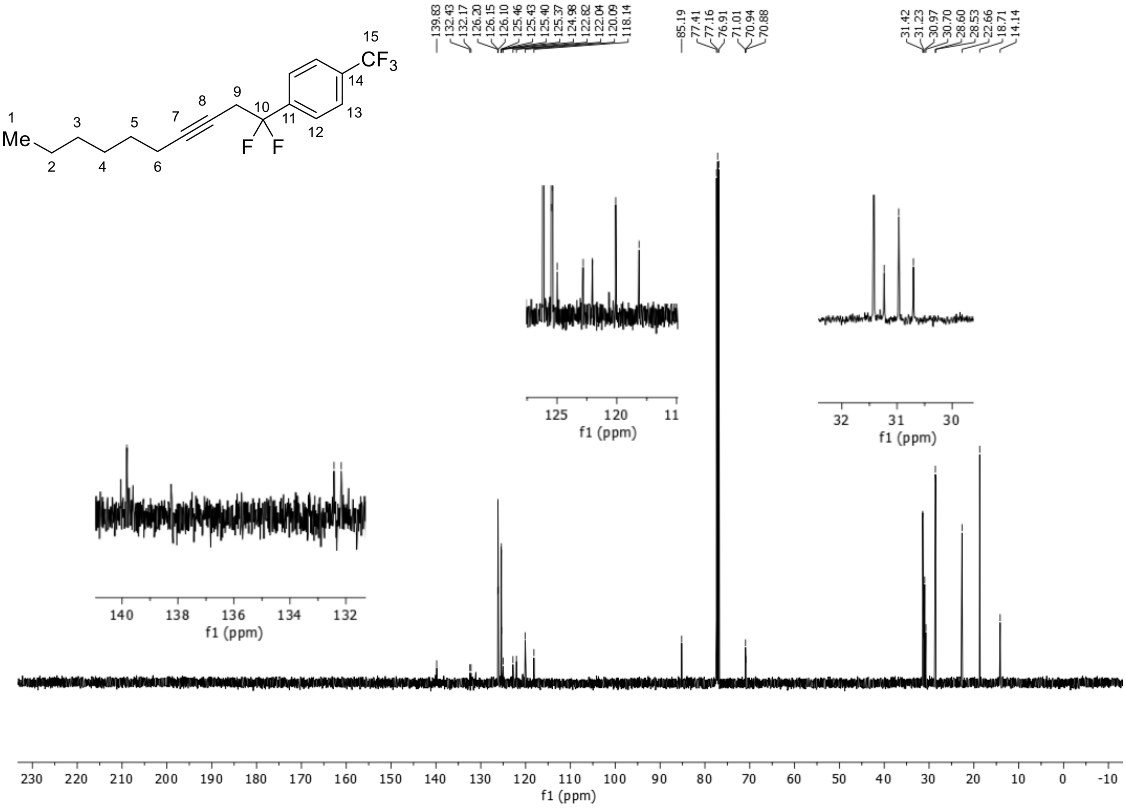

Supplementary Figure 270. $^{13}C\{^{1}H\}$ NMR of **50** (126 MHz, 299 K, $CDCl_3$).

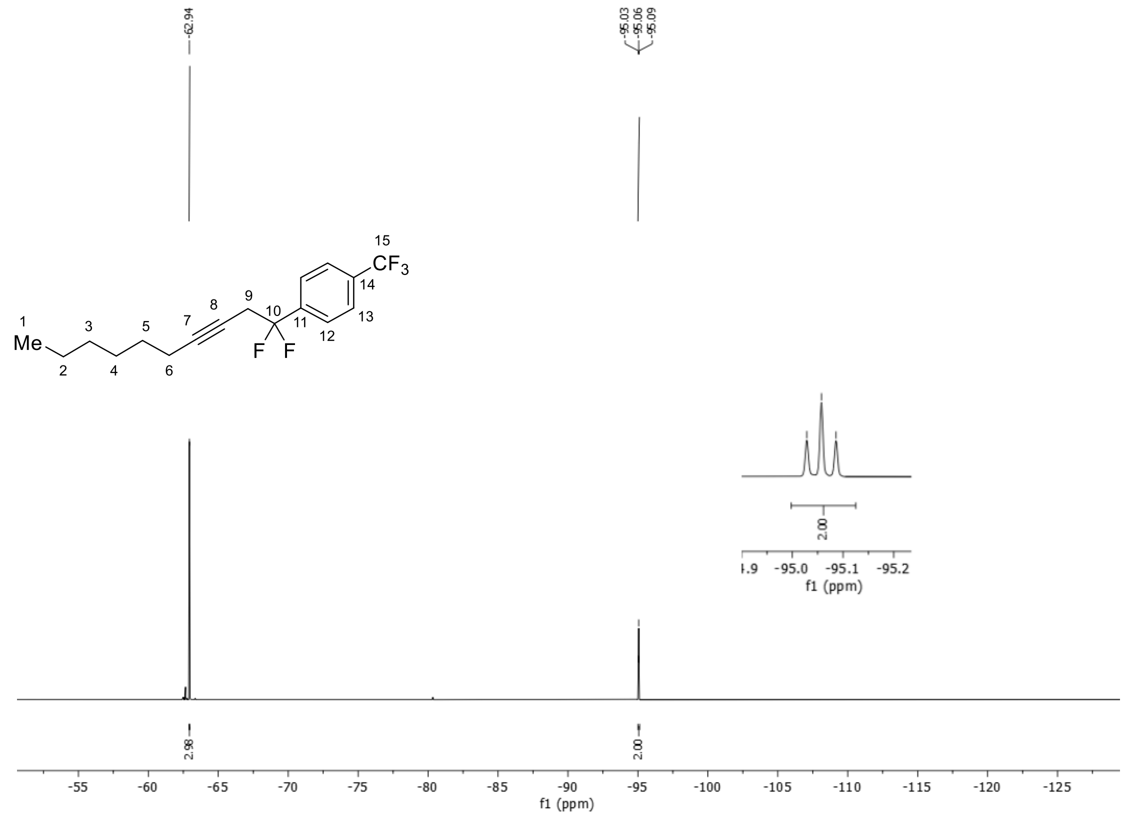

Supplementary Figure 271. ^{19}F NMR of **50** (470 MHz, 299 K, $CDCl_3$).

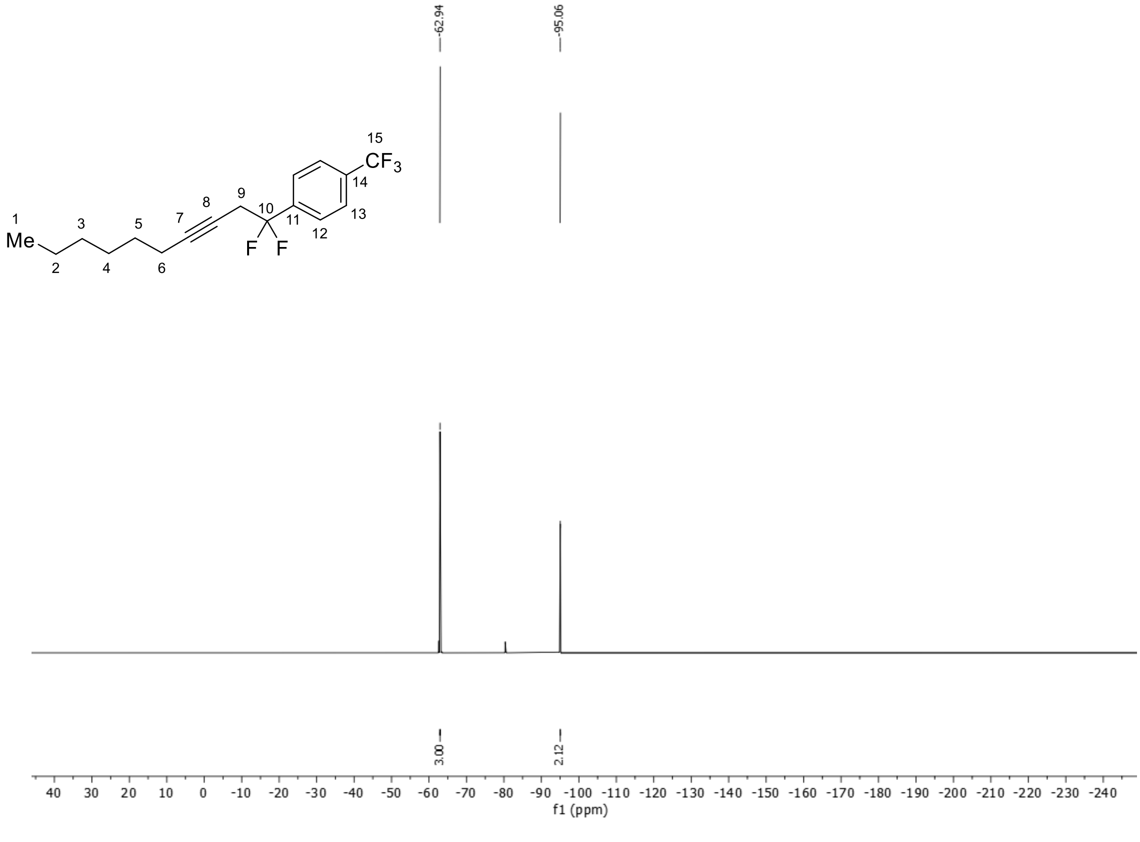

Supplementary Figure 272. ^{19}F{^{1}H} NMR of **50** (377 MHz, 299 K, $CDCl_3$).

(Methyl-d3)triphenylphosphonium bromide (Int 3)

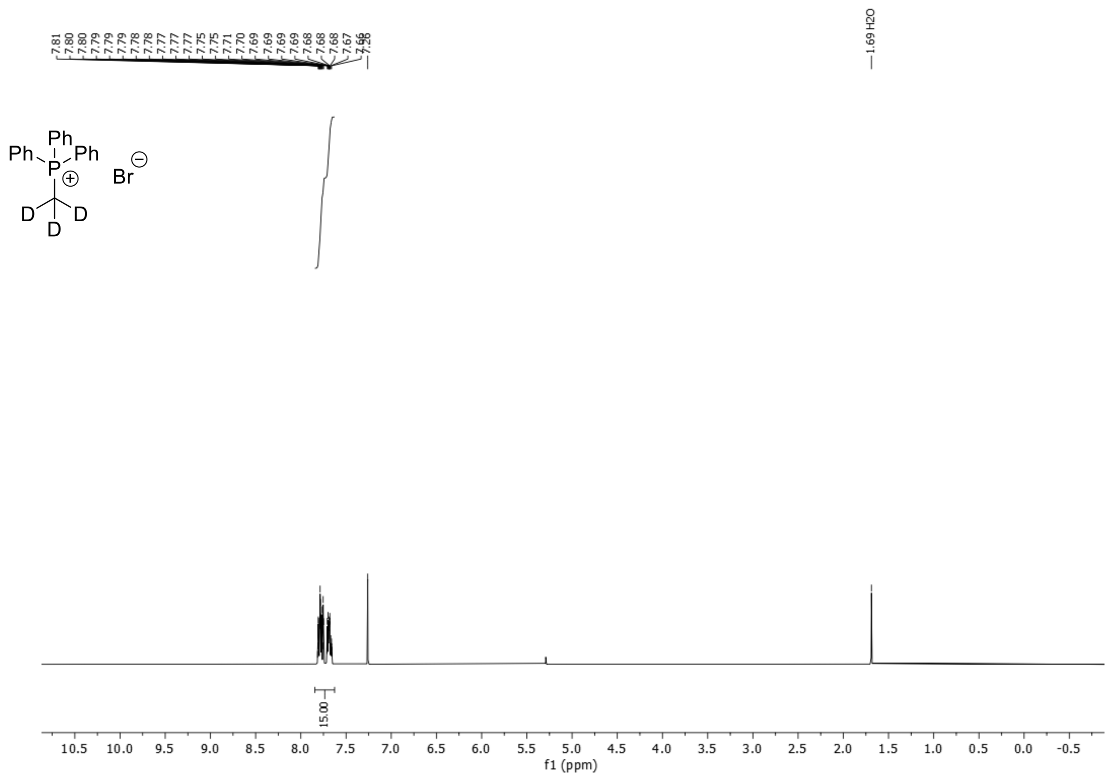

Supplementary Figure 273. ^{1}H NMR of **Int 3** (400 MHz, 299 K, $CDCl_3$).

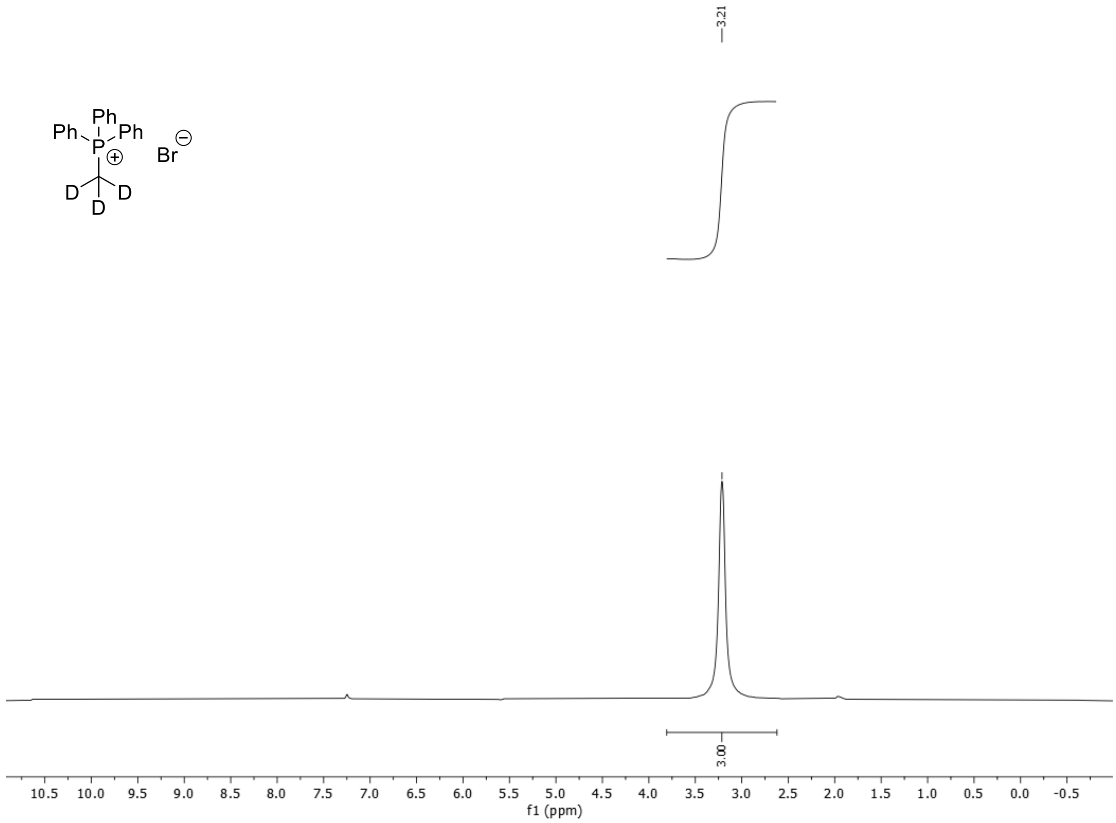

Supplementary Figure 274. ^{2}H NMR of **Int 3** (77 MHz, 299 K, $CHCl_3$).

2-Methyl-3-methyleneoctadec-4-yne (S29-d)

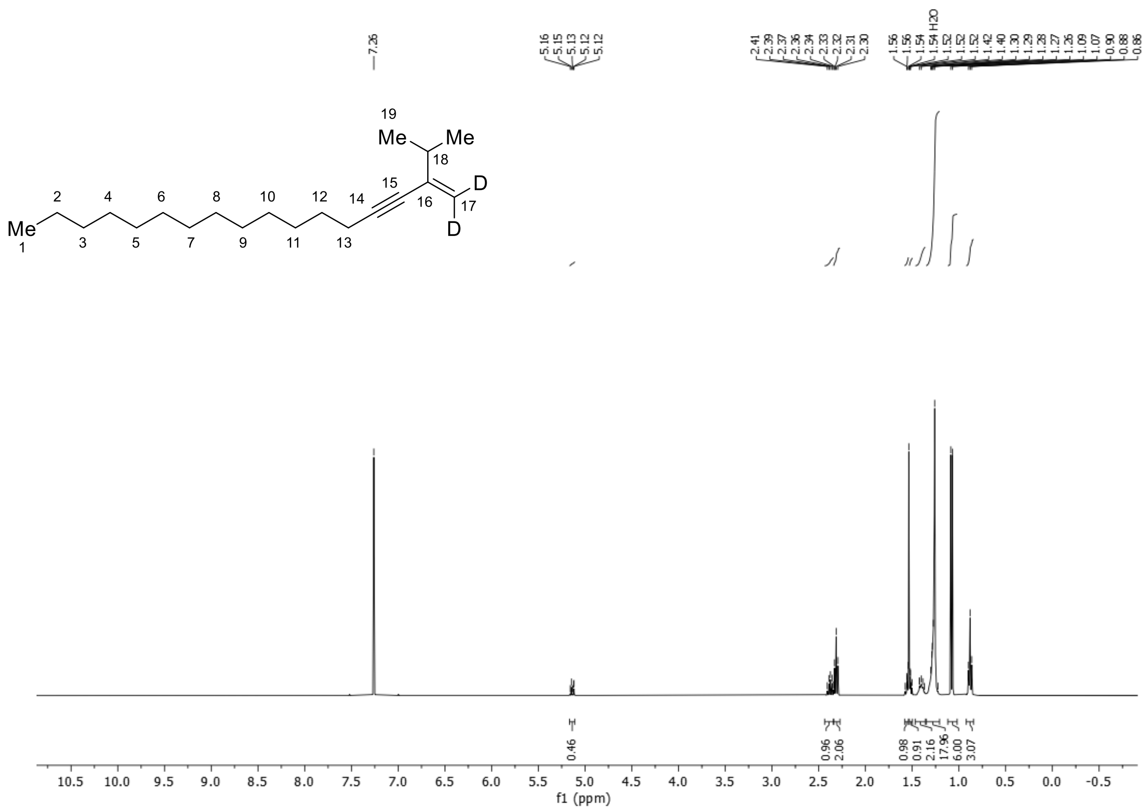

Supplementary Figure 275. ^{1}H NMR of **S29-d** (400 MHz, 299 K, $CDCl_3$).

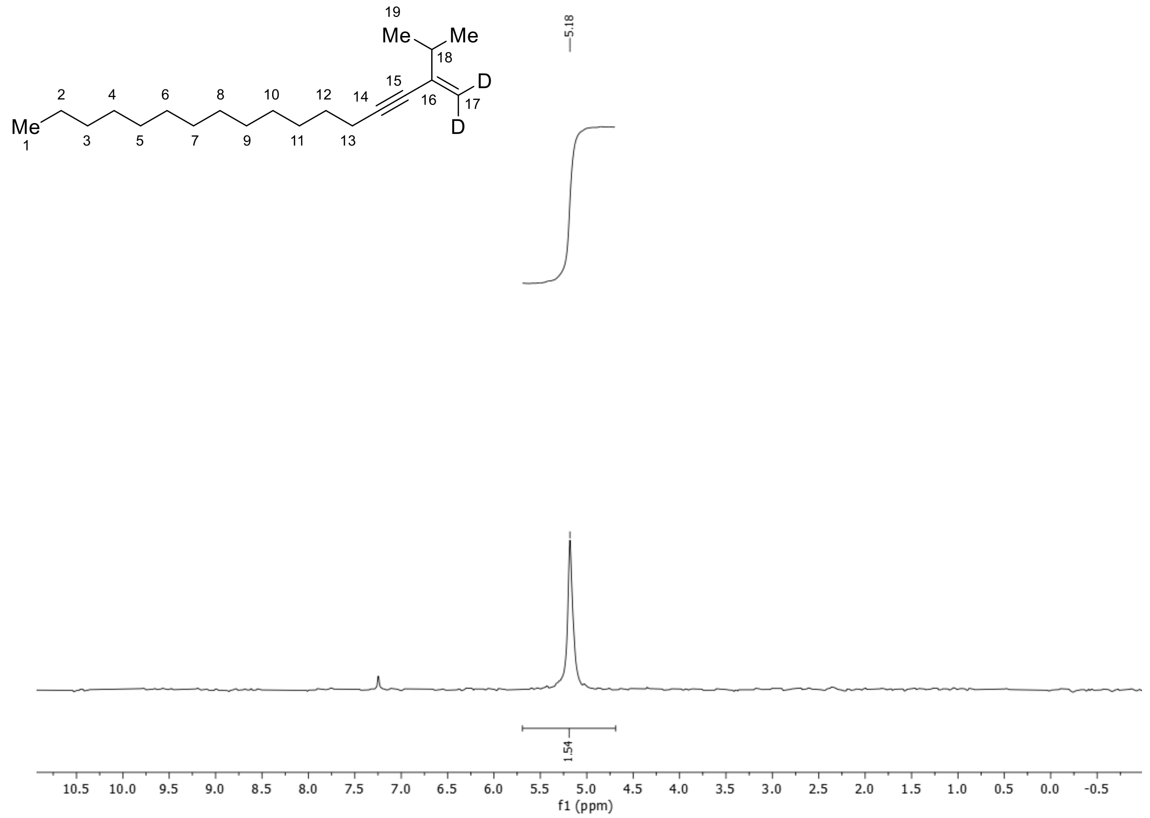

Supplementary Figure 276. ^{2}H NMR of **S29-d** (77 MHz, 299 K, $CHCl_3$).

3,3-Difluoro-2-methylnonadec-5-yne (29-d)

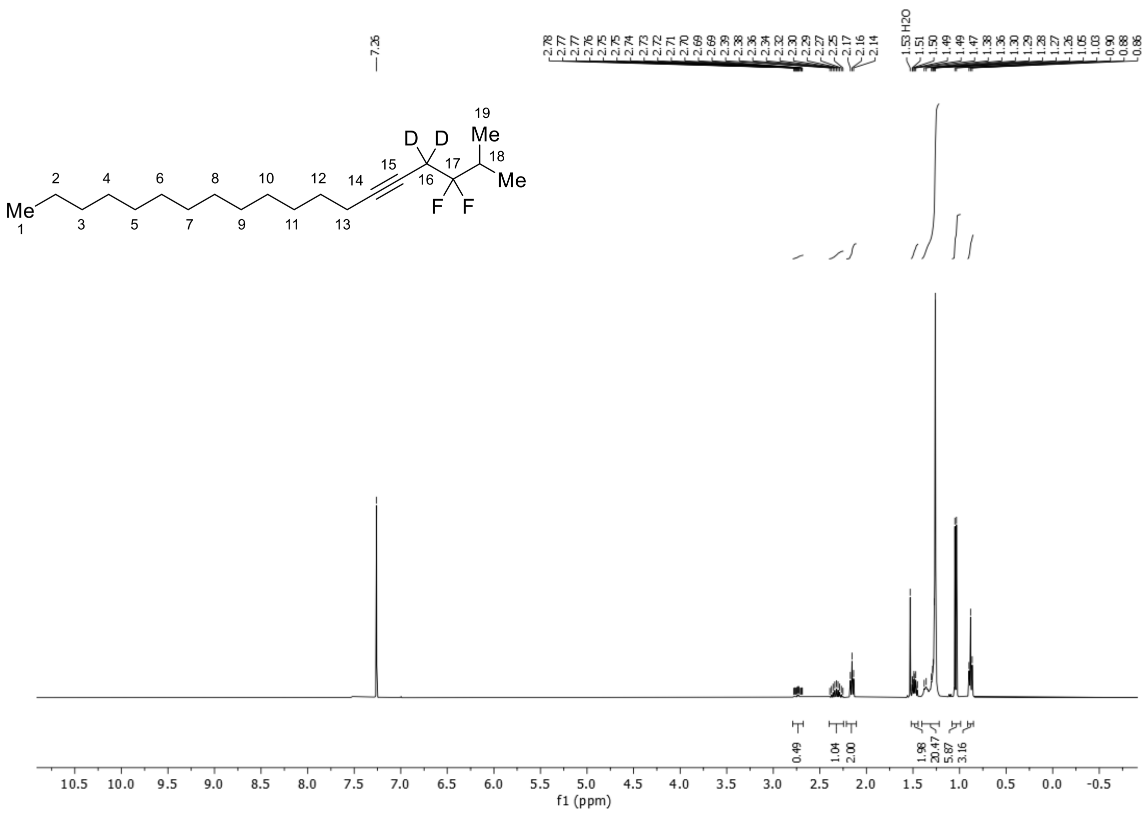

Supplementary Figure 277. ^{1}H NMR of **29-d** (400 MHz, 299 K, $CDCl_3$).

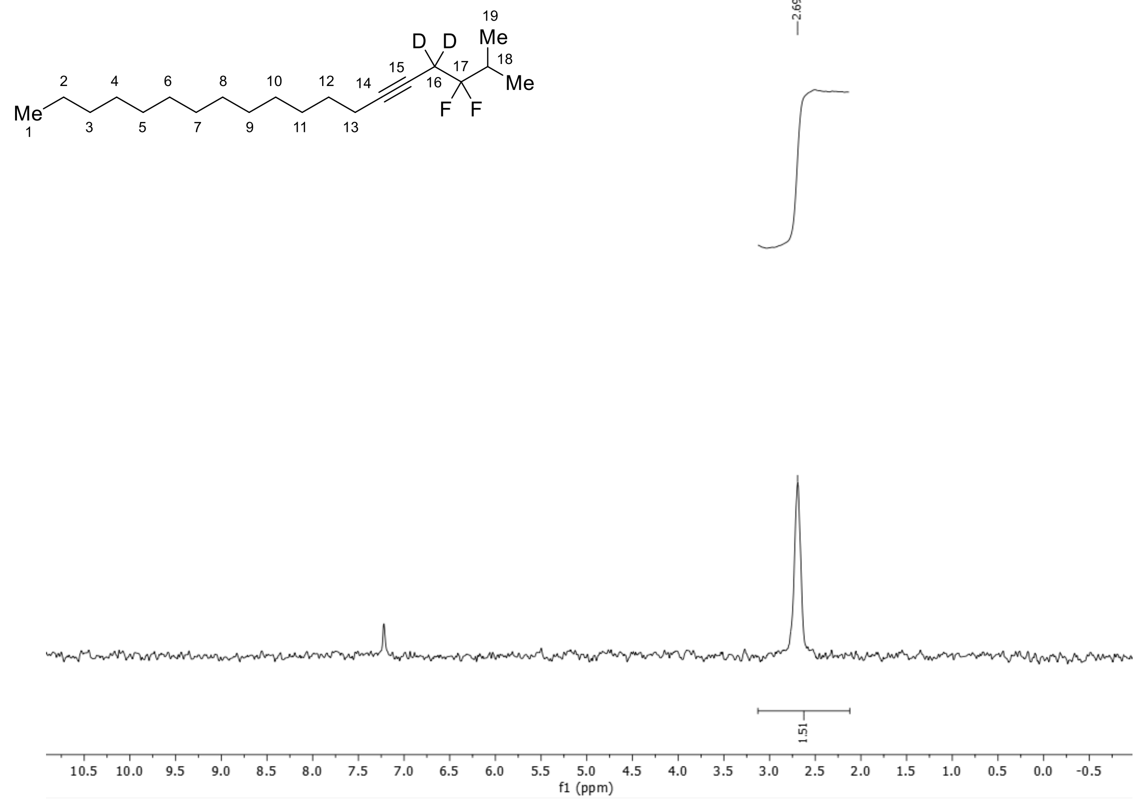

Supplementary Figure 278. ^{2}H NMR of **29-d** (77 MHz, 299 K, $CHCl_3$).

2. Supplementary References

[1] Häfliger, J., Livingstone, K., Daniliuc, C. G. & Gilmour, R. Difluorination of α-(bromomethyl)styrenes via I(I)/I(III) catalysis: facile access to electrophilic linchpins for drug discovery. *Chem. Sci.* **12**, 6148-6152 (2021).

[2] Song, Y. *et al.* Copper-catalyzed radical approach to allenyl iodides. *Chem. Commun.* **55**, 11774-11777 (2019).

[3] Picher, M.-I. & Plietker, B. Fe-Catalyzed Selective Cyclopropanation of Enynes under Photochemical or Thermal Conditions. *Org. Lett.* **22**, 340-344 (2020).

[4] Hou, H. *et al.* Stereo- and Regioselective cis-Hydrophosphorylation of 1,3-Enynes Enabled by the Visible-Light Irradiation of $NiCl_2(PPh_3)_2$. *Org. Lett.* **23**, 2981-2987 (2021).

[5] Wang, X. & Studer, A. Metal-Free Direct C−H Cyanation of Alkenes. *Angew. Chem. Int. Ed.* **57**, 11792-11796 (2018).

[6] Lee, C.-L. *et al.* First total synthesis of antrocamphin A and its analogs as anti-inflammatory and anti-platelet aggregation agents. *Org. Biomol. Chem.* **9**, 70-73 (2011).

[7] Pourjavadi, A. & Marandi, G. B. Selective synthesis of conjugated enynes from α-arylalkynols using LiCl-Acidic Al_2O_3 under solvent-free conditions. *Journal of Chemical Research* **2002**, 378-380 (2022).

[8] Pourjavadi, A. & Marandi, G. B. Preparation of Conjugated Enynes and Arylacetylenic Compounds from Arylalkynols using Alumina in Dry Media. *Journal of Chemical Research* **2002**, 552-555 (2022).

[9] Xu, T., Wu, S., Zhang, Q.-N., Wu, Y., Hu, M. & Li, J.-H. Dual Photoredox/Nickel-Catalyzed 1,4-Sulfonylarylation of 1,3-Enynes with Sulfinate Salts and Aryl Halides: Entry into Tetrasubstituted Allenes. *Org. Lett.* **23**, 8455-8459 (2021).

[10] Hashmi, A. S. K., Häffner, T., Rudolph, M. & Rominger, F. Cyclization of 2-Alkynylallyl Alcohols to Highly Substituted Furans by Gold(I)–Carbene Complexes. *Eur. J. Org. Chem.* **2011**, 667-671 (2011).

[11] Zhang, K.-F. *et al.* Nickel-Catalyzed Carbofluoroalkylation of 1,3-Enynes to Access Structurally Diverse Fluoroalkylated Allenes. *Angew. Chem. Int. Ed.* **58**, 5069-5074 (2019).

[12] Shirakawa, E., Imazaki, Y. & Hayashi, T. Ruthenium-catalyzed transformation of alkenyl triflates to alkenyl halides. *Chem. Commun.* 5088-5090 (2009).

[13] Tsukada, N., Ninomiya, S., Aoyama, Y. & Inoue, Y. Palladium-Catalyzed Selective Cross-Addition of Triisopropylsilylacetylene to Internal and Terminal Unactivated Alkynes. *Org. Lett.* **9**, 2919-2921 (2007).

[14] Cui, X.-Y. *et al.* (Guanidine)copper Complex-Catalyzed Enantioselective Dynamic Kinetic Allylic Alkynylation under Biphasic Condition. *J. Am. Chem. Soc.* **140**, 8448-8455 (2018).

[15] Feng, X.-T., Ren, J.-X., Gao, X., Min, Q.-Q. & Zhang, X. 3,3-Difluoroallyl sulfonium salts: Practical and bench-Stable reagents for highly regioselective gem-difluoroallylations. *Angew. Chem. Int. Ed.* **61**, e202210103 (2022).

[16] Zhang, Y., Yu, B., Gao, B., Zhang, T. & Huang, H. Triple-Bond Insertion Triggers Highly Regioselective 1,4-Aminomethylamination of 1,3-Enynes with Aminals Enabled by Pd-Catalyzed C–N Bond Activation. *Org. Lett.* **21**, 535-539 (2019).

[17] Zhao, Z., Racicot, L. & Murphy, G. K. Fluorinative Rearrangements of Substituted Phenylallenes Mediated by (Difluoroiodo)toluene: Synthesis of α-(Difluoromethyl)styrenes. *Angew. Chem. Int. Ed.* **56**, 11620-11623 (2017).

[18] Bruker AXS (**2021**) *APEX4 Version 2021.4-0*, *SAINT Version 8.40B* and *SADABS Bruker AXS area detector scaling and absorption correction Version 2016/2*, Bruker AXS Inc., Madison, Wisconsin, USA.

[19] Sheldrick, G. M. SHELXT – Integrated space-group and crystal-structure determination. *Acta Cryst.*, **A71**, 3-8 (2015).

[20] Sheldrick, G.M. Crystal structure refinement with SHELXL, *Acta Cryst.*, **C71**, 3-8 (2015).

[21] Bruker AXS (**1998**) *XP – Interactive molecular graphics, Version 5.1*, Bruker AXS Inc., Madison, Wisconsin, USA.

[22] TURBOMOLE V7.6 2021, a development of University of Karlsruhe and Forschungszentrum Karlsruhe GmbH, 1989-2007, TURBOMOLE GmbH, since 2007; available from http://www.turbomole.com

[23] Tao, J., Perdew, J. P., Staroverov, V. N. & Scuseria, G. E. Climbing the Density Functional Ladder: Nonempirical Meta–Generalized Gradient Approximation Designed for Molecules and Solids. *Phys. Rev. Lett.*, **91**, 146401 (2003).

[24] a) Grimme, S., Antony, J., Ehrlich, S. & Krieg, H. A consistent and accurate ab initio parametrization of density functional dispersion correction (DFT-D) for the 94 elements H-Pu. *J. Chem. Phys.* **132**, 154104 (2010).

b) Grimme, S., Ehrlich, S. & Goerigk, L. Effect of the Damping Function in Dispersion Corrected Density Functional Theory. *J. Comput. Chem.* **32**, 1456–1465 (2011).

[25] Weigend, F. & Ahlrichs, R. Balanced basis sets of split valence, triple zeta valence and quadruple zeta valence quality for H to Rn: Design and assessment of accuracy. *Phys. Chem. Chem. Phys.* **7**, 3297–3305 (2005).

[26] Grimme, S. Supramolecular Binding Thermodynamics by Dispersion-Corrected Density Functional Theory. *Chem. Eur. J.* **18**, 9955-9964 (2012).

[27] Zhao, Y. & Truhlar, D. G. Design of Density Functionals That Are Broadly Accurate for Thermochemistry, Thermochemical Kinetics, and Nonbonded Interactions. *J. Phys. Chem. A* **109**, 5656-5667 (2005).

[28] a) Klamt, A. Conductor-like Screening Model for Real Solvents: A New Approach to the Quantitative Calculation of Solvation Phenomena. *J. Phys. Chem.* **99**, 2224-2235 (1995).

b) Eckert, F. & Klamt, A. COSMO*therm*, Version C3.0; COSMO*logic* GmbH & Co. KG, Leverkusen, Germany, **2013**.
